# Differential inflammatory responses of the native left and right ventricle associated with donor heart preservation

**DOI:** 10.14814/phy2.15004

**Published:** 2021-08-26

**Authors:** Ienglam Lei, Wei Huang, Peter A. Ward, Jordan S. Pober, George Tellides, Gorav Ailawadi, Francis D. Pagani, Andrew P. Landstrom, Zhong Wang, Richard M. Mortensen, Marilia Cascalho, Jeffrey Platt, Yuqing Eugene Chen, Hugo Yu Kor Lam, Paul C. Tang

**Affiliations:** ^1^ Department of Cardiac Surgery University of Michigan Frankel Cardiovascular Center Ann Arbor Michigan USA; ^2^ Department of Pathology University of Michigan Medical School Ann Arbor Michigan USA; ^3^ Department of Immunobiology Yale University New Haven Connecticut USA; ^4^ Department of Surgery Yale University New Haven Connecticut USA; ^5^ Department of Pediatrics Duke University Durham North Carolina USA; ^6^ Department of Internal Medicine University of Michigan Frankel Cardiovascular Center Ann Arbor Michigan USA; ^7^ Department of Surgery University of Michigan Medical School Ann Arbor Michigan USA; ^8^ Hypahub Inc San Jose California USA

**Keywords:** contractile function, inflammation, ischemia, myocardial biology, transplantation

## Abstract

**Background:**

Dysfunction and inflammation of hearts subjected to cold ischemic preservation may differ between left and right ventricles, suggesting distinct strategies for amelioration.

**Methods and Results:**

Explanted murine hearts subjected to cold ischemia for 0, 4, or 8 h in preservation solution were assessed for function during 60 min of warm perfusion and then analyzed for cell death and inflammation by immunohistochemistry and western blotting and total RNA sequencing. Increased cold ischemic times led to greater left ventricle (LV) dysfunction compared to right ventricle (RV). The LV experienced greater cell death assessed by TUNEL^+^ cells and cleaved caspase‐3 expression (*n* = 4). While IL‐6 protein levels were upregulated in both LV and RV, IL‐1β, TNFα, IL‐10, and MyD88 were disproportionately increased in the LV. Inflammasome components (NOD‐, LRR‐, and pyrin domain‐containing protein 3 (NLRP3), adaptor molecule apoptosis‐associated speck‐like protein containing a CARD (ASC), cleaved caspase‐1) and products (cleaved IL‐1β and gasdermin D) were also more upregulated in the LV. Pathway analysis of RNA sequencing showed increased signaling related to tumor necrosis factor, interferon, and innate immunity with ex‐vivo ischemia, but no significant differences were found between the LV and RV. Human donor hearts showed comparable inflammatory responses to cold ischemia with greater LV increases of TNFα, IL‐10, and inflammasomes (*n* = 3).

**Conclusions:**

Mouse hearts subjected to cold ischemia showed time‐dependent contractile dysfunction and increased cell death, inflammatory cytokine expression and inflammasome expression that are greater in the LV than RV. However, IL‐6 protein elevations and altered transcriptional profiles were similar in both ventricles. Similar changes are observed in human hearts.

## Graphical abstract

Increasing duration of murine donor heart storage with subsequent ex‐vivo perfusion using clinically utilized histidine‐tryptophan‐ketoglutarate preservation solution induced progressively higher expression of IL‐1β, MyD88, TNFα, IL‐6, MCP1, and inflammasome components. This inflammatory response was disproportionately greater in the left ventricle compared to the right ventricle. We confirm that LV contractility and relaxation were also degraded to a greater degree in the left ventricle compared to the right ventricle following progressively longer periods of preservation. Murine findings corroborated with results from human preserved donor hearts where we also observed selective upregulation of TNFα, MCP1, and inflammasome components (Nlrp3, cleaved IL‐1β‐p17) in the left ventricle compared to the right ventricle during cold preservation.

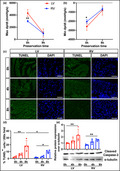

## 1 INTRODUCTION

Severe primary graft dysfunction (PGD) occurs in about 15% of patients following heart transplant and is related, in part, to cold ischemic preservation times. It remains the most common cause of early death with a 30‐day mortality of 30% (Kobashigawa et al., 2014). The precise molecular signaling pathways contributing to PGD are not well understood and targeted therapies remain elusive. The left and right cardiac chambers have distinct embryonic origins. The primary heart field gives rise to the left ventricle (LV), and the secondary heart field is the precursor to right ventricle (RV) (Voelkel et al., 2011). Biventricular changes following reperfusion of hearts that underwent cold storage for differing periods may not be uniform. We hypothesize that the left and right heart may differ in sensitivity to reperfusion injury using current cold preservation techniques.

Inflammatory processes activated during organ preservation with cold ischemia (CIS) likely contribute to impaired contraction (Fattahi & Ward, 2017; Kalbitz, Fattahi, Grailer, et al., 2016). Increased TNFα and IL‐6 expression, two key pro‐inflammatory cytokines, have been identified in human donor hearts that were not transplanted due to poor function (Birks, Burton, et al., 2000; Singh et al., 2019). Elevated serum IL‐6 in particular correlates with the occurrence of PGD in the postoperative period (Plenz et al., 2002). In sepsis, complement 5a and IL‐1β can inhibit cardiomyocyte ATPase expression (Kalbitz, Fattahi, Grailer, et al., 2016; Kalbitz, Fattahi, Herron, et al., 2016). Intensive activation of inflammatory pathways by cytokines may lead to cell death. For example, TNF signaling may induce apoptosis in some cell types and necroptosis in others (Choi et al., 2019). Inflammasome activation not only can contribute to activation and secretion of inflammatory cytokines‐like IL‐1b or IL‐18, but also can induce pyroptosis by cleavage and activation of the pore‐forming protein gasdermin D (Liu et al., 2016). Inflammatory pathways can additionally be activated by sensing of damage associated molecular pattern ligands (e.g., heat shock proteins, HMGB1) released by ischemic cardiac cells that signal through innate immune pathways, often involving the adaptor protein MyD88 (Arslan et al., 2011; Vilahur & Badimon, 2014). Thus, cell death may exacerbate inflammation, amplifying the response.

The pulmonary circulation has a much lower resistance so the adult RV can maintain a cardiac output equal to that of the LV at approximately a fifth of the energy cost (Dell'Italia & Walsh, 1988). Correspondingly, maximal sarcomere shortening in RV myocytes is significantly decreased compared with LV myocytes (Kondo et al., 2006). The adult RV therefore tolerates acute increases in afterload poorly. This may be exerted by increased pulmonary vascular resistance and/or back pressure from poor LV ejection (MacNee, 1994). Increased RV afterload was also reported with longer preservation times due to increased LV stiffness (Schroeder et al., 2018). Here we analyzed the left/right differences in the response of mouse hearts subjected to varying durations of CIS in preservation solution. It models the preservation of human donor hearts where isolated or biventricular dysfunction are a major source of transplantation morbidity and mortality (Kobashigawa et al., 2014).

## 2 METHODS

### 2.1 Experimental animal procedure

All experiments were approved by the Institutional Animal Care and Use Committee of the University of Michigan and were performed in accordance with the recommendations of the American Association for the Accreditation of Laboratory Animal Care. The isolated ex‐vivo perfusion mouse heart model was performed as previously described (DeWitt et al., 2016; Li et al., 2017) with modifications. Briefly, both male and female wild type C57BL/6J mice between 8–10 weeks of age were randomly assigned for the described experiments. Mice were heparinized (100 U, i.p.) 30 min before anesthesia with isoflurane. The heart was then dissected free and excised with care to avoid damage to the ascending aorta. A 30 gauge needle was engaged with the aorta for infusion of histidine‐tryptophan‐ketoglutarate (HTK) solution (7 ml) into the myocardium (HTK preservation solution was a gift from Essential Pharmaceuticals). The heart is then placed immediately on the ex‐vivo apparatus for reperfusion (0 h) or submerged and stored in cold HTK at 4°c for 4 and 8 h. At predesignated time points, murine hearts were retrieved and mounted on the ex‐vivo perfusion apparatus (ADInstruments). The heart was then perfused a constant pressure of 80 mm Hg with Krebs Henseleit (KH) buffer (118 mM NaCl, 25 mM NaHCO_3_, 5.3 mM KCl, 2.0 mM CaCl_2_, 1.2 mM MgSO_4_, 0.5 mM EDTA, 5.5 mM glucose, and 0.5 mM pyruvate, equilibrated with 95% O_2_ and 5% CO_2_, pH 7.4). After 30 min of equilibration, a balloon pressure transducer was inserted into the right ventricle through the right atrium. The balloon pressure transducer was connected to a bridge amplifier (Bridge Amp, ADInstruments, Inc) and recorded using Labchart pro (ADInstruments, Inc). Heart function was determined by calculating the steady state dp/dt for 20 min. After measurement of the RV cavitary pressures, the balloon is removed from the RV and then introduced into the LV through the left atrium of the same heart for evaluation of cavitary pressure. Baseline function was monitored at an end diastolic pressure (EDP) of 5–10 mm Hg by adjusting ballon volume. The balloon for measurement of intracavitary pressure on the small murine hearts are fabricated from water and corn syrup over dried pasta, and then allowed to set as previously described by Miller et al (Miller & Wright, 2011). After a total of 60 min of perfusion, LV and RV tissue was collected for analysis.

### 2.2 Procurement of human donor heart

Approval for this study was obtained from the University of Michigan Institutional Review Board (HUM00131275). Three human donor hearts from deceased brain death donors were procured as per standard clinical protocols from the Gift of Life Michigan (GOLM) organ procurement facility following cardioplegic arrest with 2 L of 4°C histidine‐tryptophan‐ketoglutarate (HTK) solution (HTK preservation solution was a gift from Essential Pharmaceuticals) were infused into the aortic root. The donor heart is then excised for analysis. Myocardial biopsies (~5 mm^3^) of LV and RV free wall were biopsied immediately after cardioplegic arrest (0 h) as well as after 4 hours (4 h) and 8 hours (8 h) of cold storage in a cooler containing ice. The donors had an average age of 55 years, two expired from intracranial hemorrhage, one died from hypoxic brain injury, and one donor had LV hypertrophy.

### 2.3 RNA‐seq

Total RNA was extracted from the LV and RV tissues using Trizol (ThermoFisher) following manufacture's protocol. The total RNA was treated with DNAse Turbo (ThermoFisher) to remove genomic DNA. RNA quality was assessed using Agilent Bioanalyzer Nano RNA Chip (Agilent). Total RNA of 1 μg (RIN > 8) was used to prepare the sequencing library using NEBNext Stranded RNA Kit (New England Biolabs, Ipswich, Massachusetts) with mRNA selection module. The library was sequenced on illumina Nova seq (paired end, 50 base pair) at the Sequencing Core of University of Michigan (Illumina). An average of 40 M reads per sample is obtained. The aforementioned RNA‐seq analysis for the mice ex‐vivo heart perfusion data were also performed on the HypaHub's big data analytics platform for reproducibility. The raw RNA‐seq data were deposit to NCBI GEO with accession number GSE174342.

### 2.4 RNA‐seq data analysis

RNA‐seq data were quantified using Kallisto (Version 0.43.0) with default parameters using the GRCm38 reference. The estimated transcript counts were exported by tximport for Deseq2 analysis. Differential expression genes (DEGs) were then identified using Deseq2 default setting. DEGs were used for GO analysis using panther classification system and GSEA with prerank settings.

### 2.5 Immunostaining and TUNEL staining

The staining studies were performed as previously described (Lei et al., 2020). Briefly, the mice hearts after ex‐vivo perfusion were fixed in zinc fixative solution (BD Biosciences) and embedded with Optimal Cutting Temperature compound at −70°CC and sectioned into 7 μm slides using cryostat. For immunofluorescence, the sections were blocked with 5% bovine serum albumin, and incubated with anti‐Cleaved Caspase‐1 antibody (1:50 dilution, Cell Signaling Technology, Boston Massachusetts) at 4ºC overnight, after three wash of phosphate buffered saline, 2nd antibody conjugate with 594 was applied for 1 h. The sections were mounted with anti‐fade mounting medium for image evaluation. The TUNEL staining procedure was performed according to manufacturer's protocol (Sigma 11684795910). For quantification, sections were obtained both at the basal and toward the apical area of the murine heart. Under 200x magnification, we evaluated three separate high‐powered fields from each of the basal and apical areas for a total of six fields assessed per ventricle. Following enumeration of TUNEL positive cells, it was expressed as a percentage of total number of cells in the high‐powered field. An average percentage of TUNEL positive staining cells was obtained for each ventricle.

### 2.6 Western blot

Proteins were extracted in lysis buffer followed by centrifugation at 4°C for 15 min at 12,000 rpm. Protein concentration was measured by Bradford protein assay and 40 µg of total protein was separated by SDS‐PAGE and then transferred to PVDF membranes. The membranes were blocked with 5% nonfat dry milk for 1 h at room temperature and then incubated with primary antibodies overnight at 4°C. After three washings with tris‐buffered saline and Tween (TBST), the membranes were incubated with secondary antibody in TBST solution for 1 h at room temperature. After three washings, the membranes were scanned and quantified by Odyssey CLx Imaging System (LI‐COR Biosciences). Primary antibodies utilized include α‐tubulin (Cell Signaling Technology, Boston Massachusetts), IL‐1β (ABclonal), cleaved IL‐1β‐p17 (ABclonal), MyD88 (ABclonal), NLRP3 (ABclonal), ASC (ABclonal), cleaved caspase‐1 (p20) (Cell Signaling Technology), cleaved caspase‐3 (ABclonal), TNFα (ABclonal), IL‐6 (ABclonal), IL‐10 (ABclonal), and MCP1 (ABclonal).

### 2.7 Statistical analysis

GraphPad Prism Software (version 8, Graphpad) was used for statistical analysis. Data were expressed as the mean ± SD. Statistical comparisons between two groups were performed by Wilcoxon Rank Sum or Student's *t*‐test, and more than two groups were performed by Kruskal–Wallis test or two‐way ANOVA followed by post hoc Tukey comparison. Groups were considered significantly different at *p* < 0.05.

## 3 RESULTS

### 3.1 The murine left ventricle had poorer functional recovery and greater cell death following ex‐vivo reperfusion that increased with duration of cold ischemic time

**FIGURE 1:**
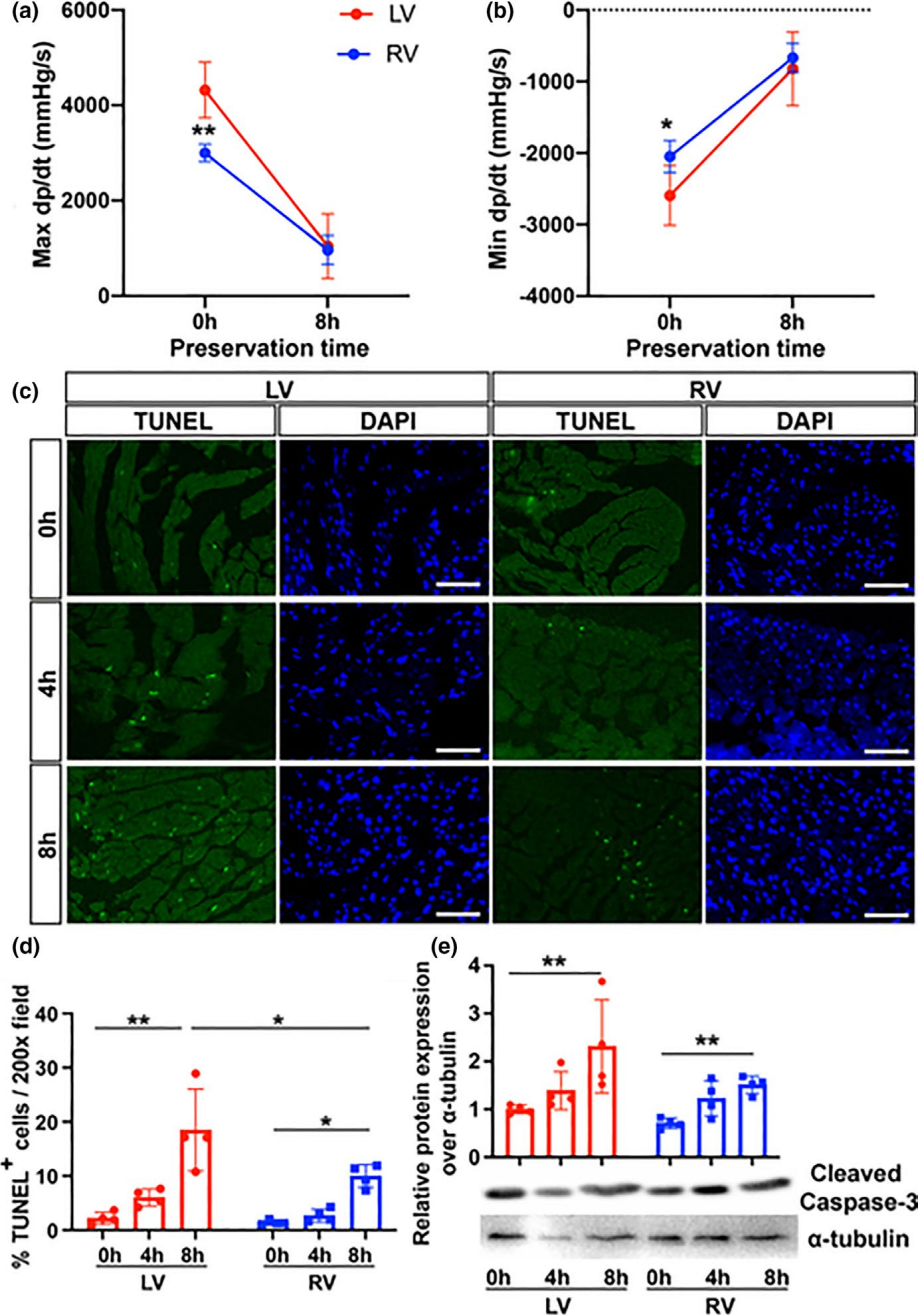
LV show worse function and more cell death during preservation. Murine donor hearts preserved in HTK with subsequent ex‐vivo reperfusion. Balloon transduced intracavitary pressures from the LV (red) and RV (blue) after 0 and 8 h of preservation demonstrating: (a) contractility (maximum dp/dt) and (b) relaxation (minimum dp/dt). n = 10–11 at each time point. (c) Representative images of TUNEL+cells (green) shown at 0, 4, and 8 h preservation in the LV and RV with DAPI (blue) staining. Magnification 200x. Scale bar 50 μm. (d) Quantification of percent TUNEL^+^ cells per average high‐ powered field (n = 4 per time point). (e) Western blot of cleaved caspase‐3 expression after 0, 4, and 8 h of preservation with reperfusion in the LV and RV. **p < 0.001, *p < 0.05, Kruskal–Wallis test or ANOVA with Tukey post hoc. LV, left ventricle; RV, right ventricle

To ensure the LV and RV coronary perfusion were comparable in different assays, we maintained the ex‐vivo cardiac perfusion pressures at 70–80 mm Hg range (Online Figure 1a). Heart function (dp/dt) was calculated based on systolic and diastolic pressures (Online Figure 1b,c). At 0 h, the LV exhibited stronger contraction (4583.8 ± 583.3 vs. 3079.6 ± 227.5 mm Hg/s, *p *< 0.001, Figure 1a) and relaxation (−2718.7 ± 505.8 vs. −2138.9 ± 217.2 mm Hg/s, *p *= 0.027, Figure 1b) compared with the RV as expected. The respective baseline (0 h) LV contractility and relaxation were higher at about 148.8% and 127.1% of the respective RV measurements. After 8 h of CIS however, the difference in LV versus RV contractility (1043.5 ± 678.9 vs. 963.2 ± 304.1 mm Hg/s, *p *= 0.981) and relaxation (−822.6 ± 515.8 vs. −668.8 ± 204.3 mm Hg/s, *p *= 0.793) was no longer present (Figure 1a,b). At 8 h, the contractility and relaxation in the LV were 22.8% and 30.3% of LV baseline while for the RV it was 31.3% and 31.3%, respectively of RV baseline. When examining intracavitary pressures as a ratio of 8 over 0 hours, contractility (Max dp/dt) ratio was lower in the LV compared to RV (Online Figure 2a, 0.211 vs. 0.331, *p *= 0.037) but there was no difference in relaxation (Min dp/dt, Online Figure 2b, 0.28 vs. 0.33, *p *= 0.464). This indicates that the LV had a greater degradation in cardiac function compared with the RV after CIS for 8 h.

**FIGURE 2:**
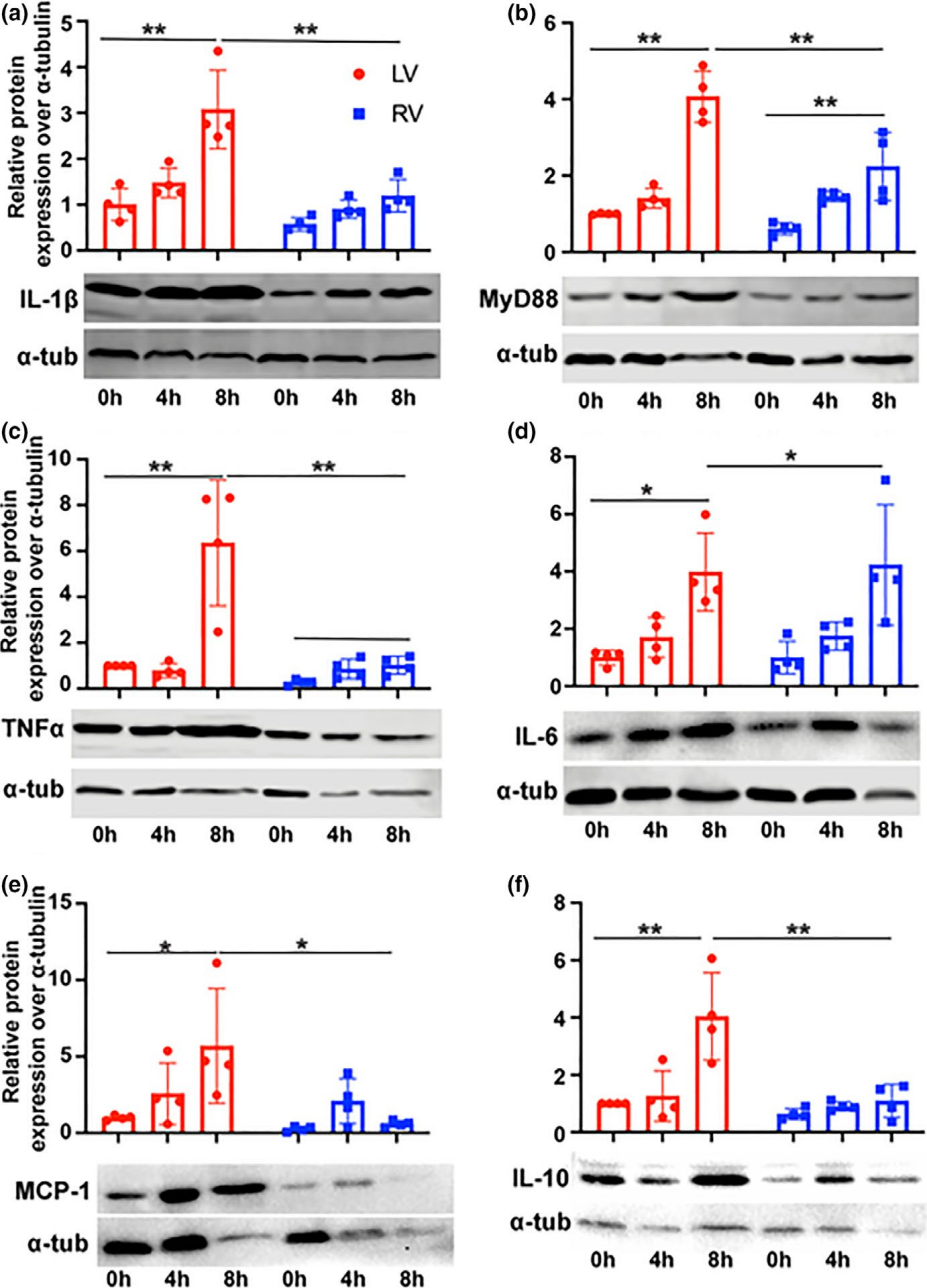
Inflammatory mediator expression is greater in the LV. Western blot analysis of murine LV and RV samples following ex‐vivo perfusion with preservation at 0, 4, and 8 h to determine protein expression of (a) IL1β, (b) MyD88, (c) TNFα, (d) IL‐6, (e) MCP1, and (f) IL‐10. n = 4 per time point. **p < 0.001, *p < 0.05, Kruskal–Wallis test or ANOVA with Tukey post hoc, Relative fold change are normalized to 0 h. LV, left ventricle; RV, right ventricle

TUNEL staining showed that ex‐vivo perfusion after progressively longer periods of CIS at 0, 4, and 8 h showed greater cell death in both the LV and RV. However, cell death was more numerous in the LV (Figure 1c). In the LV (Figure 1d), the average % of TUNEL +* *cells per 200x power field progressively increased from baseline (2.2 ± 1.1%) to 8 h (18.5 ± 7.6%, *p *< 0.001). Similarly, TUNEL^+^ cells increased in the RV from baseline (1.4 ± 0.4%) to 8 h (10.0 ± 2.1%, *p* = 0.020). Percentage of TUNEL^+^ cells was greater in LV compared to RV after 8 h CIS (*p* = 0.021). We also observed that the expression of cell death marker cleaved caspase‐3 expression increased (Figure 1e) with increasing CIS time in the LV (2.3 at 8 h vs. 1.0 at baseline, *p* = 0.009) and RV (1.5 at 8 h vs. 0.7 at baseline, *p* = 0.001). The results suggest that decrease in LV function with increasing CIS‐reperfusion time is associated with greater cell death in the LV compared to the RV.

### 3.2 Increased expression of inflammatory mediator proteins in the murine left ventricle compared with right ventricle after preservation with reperfusion

Next, we asked whether increased CIS time with reperfusion would induce inflammatory protein responses. Results showed that inflammatory protein expression was increased in the LV at 8 h compared with baseline for IL‐1β (3.1 vs. 1.0, *p* < 0.001), MyD88 (4.1 vs. 1.0, *p* < 0.001), TNFα (6.4 vs. 1.0, *p* < 0.001), IL‐6 (4.0 vs. 1.0, *p* = 0.014), MCP1 (5.7 vs. 1, *p* = 0.022), and IL‐10 (4.0 vs. 1, *p* < 0.001, Figure 2a–d). Of these mediators, only MyD88 (2.2 vs. 0.6, *p* = 0.002), and IL‐6 (4.2 vs. 1.0, *p *= 0.007, Figure 2b,d) were increased in the RV following 8 h CIS‐reperfusion. However, RV expression of IL‐1β (1.2 vs. 0.6, *p *= 0.600), TNFα (1.0 vs. 0.3, *p* = 0.937), MCP1 (0.6 vs. 0.3, *p* = 0.999), and IL‐10 (1.1 vs. 0.6, *p* = 0.957) were not significantly elevated at 8 h compared to baseline. Comparing LV and RV at 8 h, respective expression of IL‐1β (3.1 vs. 1.2, *p* < 0.001), MyD88 (4.1 vs. 2.2, *p* < 0.001), TNFα (6.4 vs. 1.0, *p* < 0.001), MCP1 (5.7 vs. 0.6, *p* = 0.011), and IL‐10 (4.0 vs. 1.1, *p* < 0.001) was higher in the LV (Figure 2a–c, 2e‐f). We found no difference in the expression of IL‐6 (4.0 vs. 4.2, *p *= 0.999) at 8 h between the LV and RV (Figure 2d). Our results suggest that inflammatory mediators and innate immune components were preferentially increased with CIS in the LV compared with the RV during CIS‐reperfusion. Accordingly, increased anti‐inflammatory cytokine IL‐10 expression in the LV likely acts to modulate inflammation. However, IL‐6 was equally upregulated in the LV and RV of murine hearts. These results suggest that inflammation responses due to CIS are differentially regulated in the LV and RV.

### 3.3 Inflammasome expression following reperfusion is more prominent in the murine left ventricle

**FIGURE 3:**
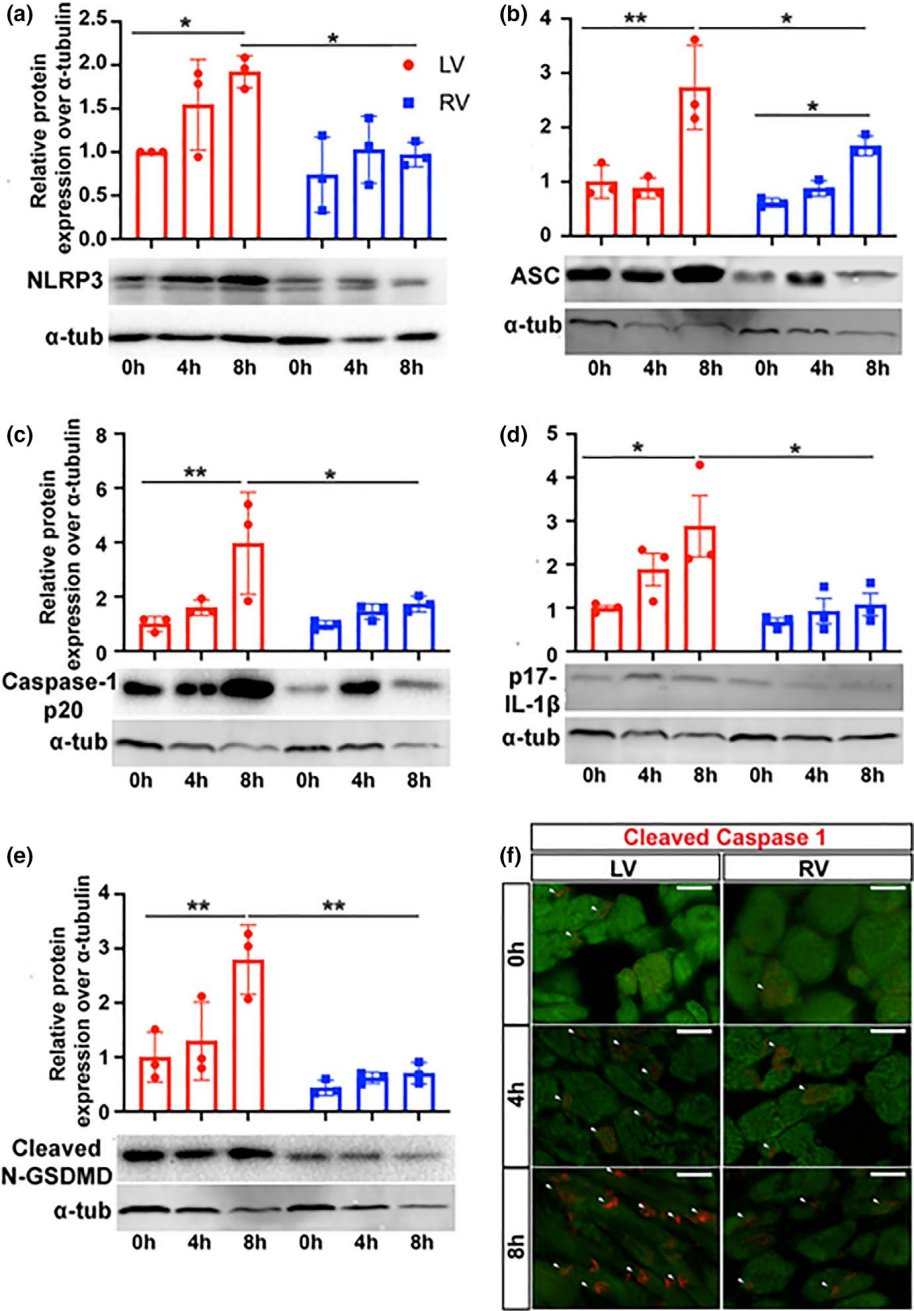
LV exhibit greater inflammasome activation. Western blot analysis of murine LV and RV samples following reperfusion with preservation at 0, 4, and 8 h to determine protein expression of inflammasome components (a) Nlrp3, (b) ASC, (c) cleaved Caspase‐1 p20, (d) p17‐IL‐1β, and (e) cleaved N‐GSDMD. n=3 per time point. **p < 0.001, *p < 0.05, Kruskal–Wallis test or ANOVA with Tukey post hoc, relative fold change are normalized to LV 0 h. (f) Immunohistochemistry for cleaved caspase‐1 (red) and troponin (green) in the LV and RV following preservation for 0, 4, and 8 h with reperfusion. White arrows indicate positive cleaved caspase‐1 staining (red). Magnification 400x. Scale bar 20 μm. LV, left ventricle; RV, right ventricle

Because IL‐1β, TNFα (McGeough et al., 2017), and MyD88 (Chilton et al., 2012) controls the inflammasome, we asked if their increased LV expression was associated with increase inflammasome component expression. Compared with baseline 0 h in the LV, 8 h CIS increased the expression of Nlrp3 (1.9 vs. 1.0, *p* = 0.046), ASC (2.7 vs. 1.0, *p* < 0.001), caspase‐1 p20 (4.0 vs. 1.0, *p* = 0.007), p17‐IL‐1β (2.9 vs. 1.0, *p* = 0.031), and cleaved N terminal‐Gastrodermin D (N‐GSDMD, 2.8 vs. 1.0, *p* = 0.003) (Figure 3a–e). In the RV, while there was an 8 h increase in ASC expression (1.7 vs. 0.6, *p *= 0.037), there was no difference in the expression of Nlrp3 (1.0 vs. 0.7, *p *= 0.950), caspase‐1 p20 (1.7 vs. 1.0, *p *= 0.840), p17‐IL‐1β (1.1 vs. 0.7, *p *= 0.968), and cleaved N‐GSDMD (0.7 vs. 0.4, *p *= 0.974, Figure 3a–e). At 8 h, the LV had greater expression of Nlrp3 (1.9 vs. 1.0, *p *= 0.038), ASC (2.7 vs. 1.6, *p* = 0.032), caspase‐1 p20 (4.0 vs. 1.7, *p* = 0.047), cleaved p17‐IL‐1β (2.9 vs. 1.1, *p* = 0.039) as well as cleaved N‐GSDMD (2.8 vs. 0.7, *p* = 0.001). Immunohistochemistry showed prominent expression of cleaved caspase‐1 (p20) at 8 h in LV cardiomyocytes with much less expression in the RV at the same time point (Figure 3d). Therefore, increased CIS with reperfusion increases expression of inflammasome components in LV cardiomyocytes.

### 3.4 The left ventricle of human hearts also demonstrates preferential inflammatory responses during static cold preservation

**FIGURE 4:**
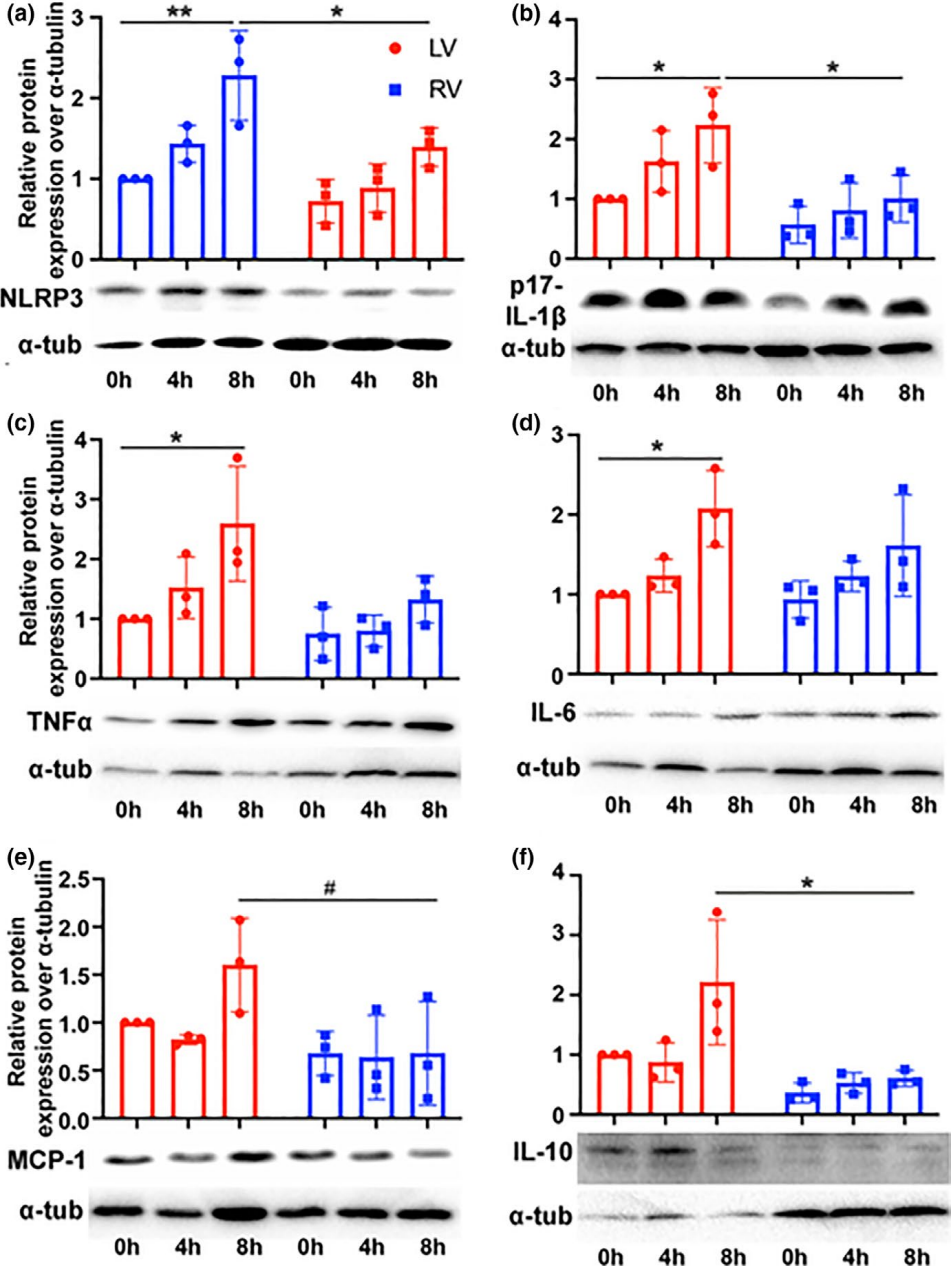
Human donor hearts also show more pronounced inflammatory response in the LV with preservation. Human LV and RV were preserved with cold HTK and biopsied at 0, 4, and 8 h in the same donor heart. Western blot show the LV and RV expression of (a) Nlrp3, (b) IL‐1β, (c) TNFα, (d) IL‐6, (e) MCP1, and (f) IL‐10. **p < 0.001, *p < 0.05, ^#^ *p* < 0.1 Kruskal–Wallis test or ANOVA with Tukey posthoc, LV, left ventricle; RV, right ventricle

To examine whether inflammation changes following CIS‐reperfusion are conserved between human and mice heart with CIS, we examined human donor hearts during CIS storage (Figure 4a‐b). We show greater inflammasome formation at 8 h compared with baseline in the LV with increased expression of NLRP3 (2.3 vs. 1.0, *p* = 0.036) and cleaved p17‐IL‐1β (2.2 vs. 0.1, *p* = 0.041). Other cytokines and chemokines (Figure 4c‐f) in the human LV were also elevated at 8 h compared with baseline such as TNFα (2.6 vs. 1.0, *p* = 0.026), IL‐6 (2.1 vs 1.0, *p* = 0.030), and IL‐10 (2.2 vs. 1.0, *p* = 0.062). In contrast, the RV had relatively unchanged inflammatory mediator expression with no change at 8 h of CIS compared to baseline for NLRP3 (*p* = 0.174), cleaved p17‐IL‐1β (*p* = 0.811), TNFα (*p* = 0.749), IL‐6 (*p* = 0.259), MCP1 (*p* > 0.999), and IL‐10 (*p* = 0.985). Compared with the RV, the human LV had significantly higher expression of NLRP3 (2.3 vs. 1.4, *p* = 0.036), cleaved p17‐IL‐1β (2.2 vs. 1.0, *p* = 0.042), MCP1 (2.2 vs. 0.6, *p* = 0.011), and IL‐10 (1.6 vs. 0.7, *p* = 0.011) after 8 h CIS. There was no difference in TNFα (2.6 vs. 1.3, *p* = 0.091) or IL‐6 (2.1 vs. 1.6, *p* = 0.625) expression in the human LV and RV at 8 h CIS‐reperfusion. The results indicate that like in CIS‐reperfused murine hearts, static CIS of human hearts‐ induced preferential expression of inflammatory mediators in the LV.

### 3.5 Native murine left and right ventricle show progressive immune transcript expression after cold ischemic preservation followed by ex‐vivo reperfusion

**FIGURE 5:**
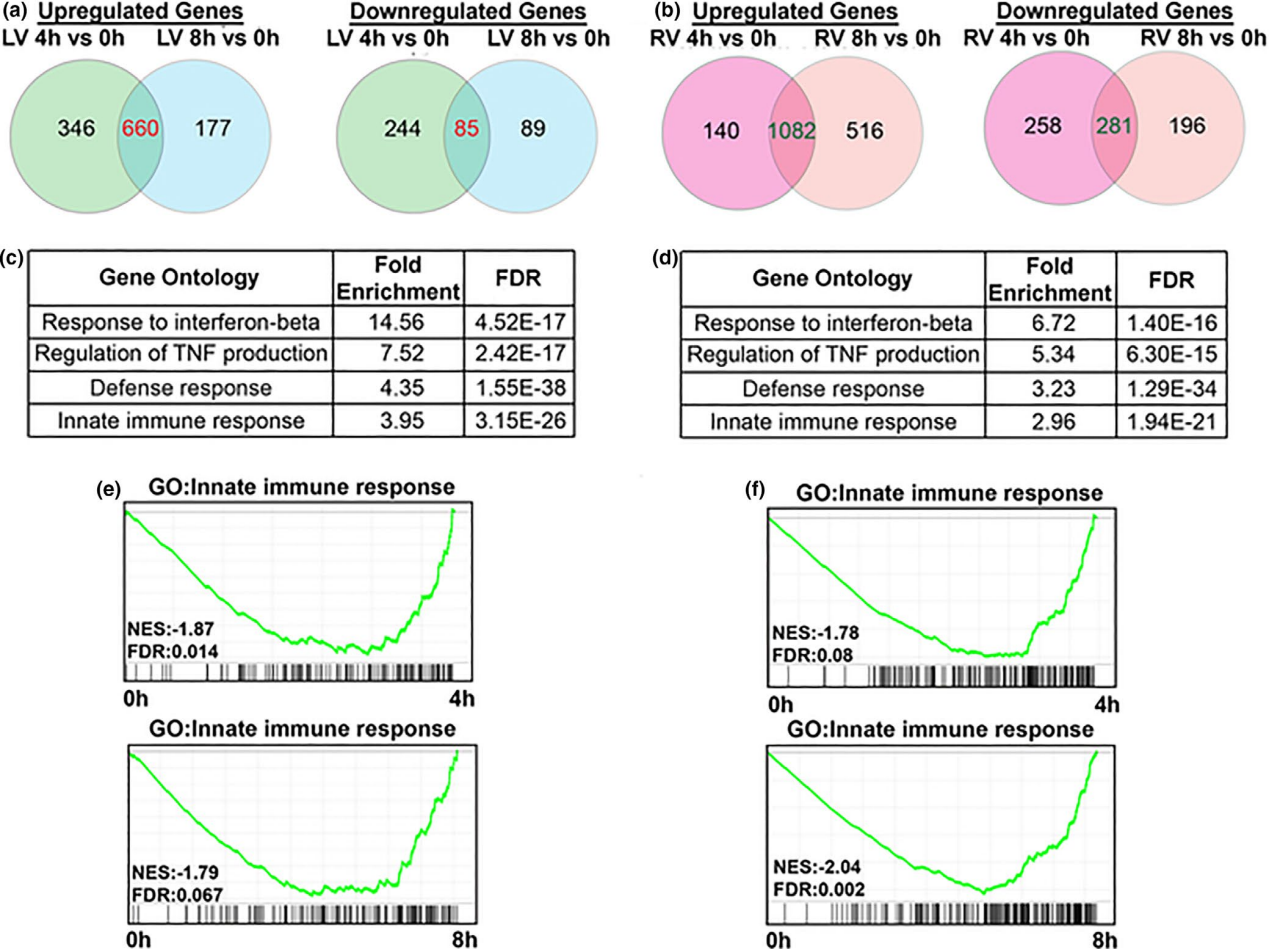
Inflammation response was transcriptionally activated in both murine LV and RV with cold preservation and reperfusion. (a) Venn diagram of upregulated and downregulated genes in LV show common regulated genes after 4 and 8 h storage followed by reperfusion. (b) Venn diagram of upregulated and downregulated genes in RV show common regulated genes after 4 and 8 h storage with reperfusion. GO analysis by panther classification system shows similar inflammation related GO gene sets were enriched in common upregulated genes of LV (c) and RV (d). Representative GSEA plot show that innate immune response gene set was enriched in LV (e) and RV (f) after 4 and 8 h preservation‐reperfusion compared to baseline 0 h. LV, left ventricle; RV, right ventricle; GO, Gene ontology

To further examine the transcriptional response of murine donor hearts to CIS with reperfusion, we performed transcriptomic analysis of LV and RV after reperfusion with different storage times. Differential gene expression (DGE, abs (fold change) >1.5, padj <0.1) was identified when comparing 4 or 8 versus 0 h in both LV and RV using DESeq2 (Figure 5a,b; Online Table S1). We performed Gene ontology (GO) pathway analysis on the common regulated genes in LV and RV, respectively. We found that the CIS‐reperfusion upregulated gene expression in several pathways in both ventricles including: Response of interferon‐beta/gamma, Regulation of TNF production, defense response, and innate immune response (Figure 5c,d). The enrichment of inflammatory responses after cold storage was further confirmed by gene set enrichment analysis (GSEA). This showed that genes related to innate immune response were highly expressed after 4 and 8 h of CIS‐reperfusion in both LV and RV (Figure 5e,f). However, these genes did not show enrichment in LV compared to RV in contrast to our observations at the protein level. This suggests that differential inflammatory responses between the LV and RV may occur via posttranslational modification mechanisms.

## 4 DISCUSSION

While many studies have examined the immunobiology of heart transplantation from the perspective of both acute and chronic rejection, there has been much less attention paid to the detrimental effects of PGD. The critical importance of PGD is apparent when considering it occurs in 2.3% to 28.2% of heart transplants and accounts for 66% of 30‐day mortalities following heart transplantation (Kobashigawa et al., 2014). This study reveals differential and shared time‐dependent inflammatory responses of the native right and left heart to cold storage.

The LV experiences greater inflammatory responses to preservation compared with the RV. This may be in part due to the unique embryonic origins of the left and right ventricle where divergent cellular responses to stress were also identified in other settings. For example, in response to pressure and volume overload, RV has a greater selective increase in expression of cardiac growth factors such as angiotensinogen (AGTN) and preproendothelin‐1 (ppET‐1) (Modesti et al., 2004). Our current study demonstrates a predictable decrease in cardiac contraction and relaxation associated with increased CIS preservation times. While the LV demonstrates a greater magnitude of LV contractile force and diastolic relaxation at baseline, it also experiences a greater decrease in these functions following prolonged preservation compared with the RV. This is accompanied by greater cell death in the LV.

Our analysis shows that with increasing CIS time with reperfusion, greater inflammatory mediator expression occurs in both the LV and RV. Unexpectedly, the LV shows a greater expression of key inflammatory cytokines, chemokines, and innate immune adaptor protein such as IL‐1β, TNFα, MCP1, and MyD88. However, IL‐6 showed similar upregulation in both the LV and RV. Interestingly, anti‐inflammatory cytokine IL‐10 expression was also preferentially increased in the LV with longer CIS time likely as a feedback mechanism for modulating inflammatory responses initiated by CIS‐reperfusion injury.

The inflammatory cytokines we examined are particularly relevant for cardiac PGD. TNFα signaling is recognized to mediate cell death (Wajant et al., 2003), and impair myocardial contractility (Goldhaber et al., 1996). TNFα expression in the donor RV myocardium correlated with the occurrence of PGD (Birks, Owen, et al., 2000). IL‐6 expression in chronic heart failure also correlated with poorer cardiac function and contributes to myocardial injury (Birks, Owen, et al., 2000). Monocyte chemoattractant protein 1 (MCP1) is a potent chemoattractant for monocytes and is upregulated in cardiac allografts early after clinical transplantation (Groot‐Kruseman et al., 2001; Rollins, 1996). MyD88 is a key innate immune adaptor protein (Vilahur & Badimon, 2014) which we show is induced with CIS storage with reperfusion. IL‐10 is an anti‐inflammatory cytokine that has been shown to modulate myocardial reperfusion injury by inhibiting TNFα and NO production, and reduces ischemia‐reperfusion associated inflammation to lessen LV dysfunction (Krishnamurthy et al., 2009; Yang et al., 2000).

Cold preservation increased the expression of inflammasome components preferentially in the LV. Inflammasome components IL‐1β, cleaved IL‐1β‐p17, Nlrp3, ASC, and cleaved caspase‐1 (p20) were increasingly upregulated with longer preservation times mainly in the LV. Correspondingly, we show that pyroptotic cell death mediator cleaved N‐GSDMD is selectively upregulated in LV. Staining confirmed greater expression of cleaved caspase‐1 in troponin positive cardiomyocytes in the LV following prolonged preservation. Indeed, IL‐1β expression and inflammasome formation has been shown to impair myocardial function in cardiomyopathy and sepsis settings (Bracey et al., 2013; Zhang et al., 2014). Dysregulation of intracellular calcium metabolism due to cytokine and complement signaling (Fattahi et al., 2018; Zhang et al., 2005) likely contributes to contractile dysfunction.

RNA sequencing in our study shows that both LV and RV had an inflammatory response (e.g., TNF and interferon pathway signaling) related to innate immune activation that is proportional to the duration of preservation time. While differential inflammatory responses to murine heart preservation was demonstrated at the protein level, we did not see mirrored differences at the transcript level. Identification of posttranslational regulatory processes is needed to better understand the relation of inflammatory transcript to protein expression.

Corroborating murine findings with human preserved heart, we observed that inflammasome components Nlrp3 and cleaved IL‐1β‐p17 were similarly selectively upregulated in the LV during static cold CIS. Expression of TNFα, MCP1, and IL‐10 was also selectively upregulated in the LV with prolonged 8 h CIS compared with the RV. Similar to murine findings, IL‐6 was upregulated to a similar degree in both ventricles with increasing CIS. This finding is in agreement with greater transcriptomic expression of inflammatory signaling pathways in the LV compared with the RV during CIS as outlined in our previous study (Lei et al., 2020). Therefore, our murine model bears significant resemblance to human donor heart cold storage responses. It is also important to recognize that human donor hearts were obtained from a brain death setting and is likely associated with systemic inflammatory responses from death related release of circulating inflammatory mediators induced by the ischemic brain (Barklin, 2009).

Future studies are needed to examine the effects of specific inflammatory mediators on the cardiac CIS‐reperfusion process using targeted genetic models. Furthermore, identification of the cellular source of cytokines and chemokines is needed. Native donor cardiac cellular responses we demonstrate in this study likely includes vascular cells (i.e., vascular smooth muscle cells, endothelial cells), cardiomyocytes, fibroblasts as well as “passenger” leukocytes (Benichou & Thomson, 2009).

Inflammatory responses to CIS‐reperfusion had a magnitude related to CIS duration. Therapeutic strategies to improve donor heart function following transplantation likely needs to be applied as close to the time of procurement as possible. This has the greatest likelihood to minimize injurious inflammatory and cell death responses that have already occurred in the donor heart during CIS and likely to amplify following reperfusion in the recipient. It is important to note that while inflammatory responses and cell death are greater in the LV, the upstream RV will bear the consequences of raised afterload from compromised LV function. Thus, the clinical manifestation of RV failure posttransplantation is likely a combination of both biological responses to CIS‐reperfusion and the unique hemodynamic workloads imposed on the RV.

Our study is limited by functional studies that were isolated to murine heart. Future studies would benefit from examining differences in cardiac functional response to preservation in large animal (e.g., pigs) and human hearts to promote translation of findings to the clinical setting. Furthermore, a working heart ex‐vivo perfusion or heart transplant model will allow greater understanding on how suboptimal LV preservation impacts the downstream RV function since the two ventricles function in a series circuit. Our study does not define the mechanisms by which LV and RV preservation differs. Subsequent studies to address how differing embryonic origins of the two ventricles impact preservation injury through defining discrete molecular pathways would greatly benefit our ability to design corresponding mitigating therapies for cardiac preservation.

In summary, our study demonstrates that donor heart CIS‐reperfusion in native cardiac cells induced expression of IL‐1β, MyD88, TNFα, IL‐6, MCP1, and inflammasome components. Inflammatory responses tend to be greater in the LV compared with the RV. LV contractility and relaxation also degraded to a greater degree. Furthermore, important similarities in cardiac inflammatory responses to preservation exist between human and murine donor hearts. Future molecular therapies targeting inflammation at the time of donor organ procurement may improve transplant graft function.

## CONFLICTS OF INTEREST

The authors have no conflicts of interest. There are no relationships with industry and financial associations within the past 2 years that might pose a conflict of interest in connection with the submitted article.

## AUTHOR CONTRIBUTIONS

I.L. and P.C.T. were involved in conception and design of the research; I.L., and W.H. performed the experiments; I.L., and H.YK.L. analyzed the data; I.L., P.A.W., J.S.P., G.T., G.A., F.D.P., A.P.L., Z.W., R.M.M., M.C., J.P., Y.E.C., and P.C.T. interpreted the results of experiments; I.L., and P.C.T. prepared the figures; I,L. and P.C.T. drafted the manuscript; I.L., P.A.W., J.S.P., G.T., G.A., F.D.P., A.P.L., Z.W., R.M.M., M.C., J.P., Y.E.C., H.Y.K.L., and P.C.T. edited and revised the manuscript; I.L., W.H., P.A.W., J.S.P., G.T., G.A., F.D.P., A.P.L., Z.W., R.M.M., M.C., J.P., Y.E.C., H.Y.K.L., and P.C.T. approved the final version of the manuscript.

## Supporting information



Fig S1‐S2

Table S1
