# Supplementary material for: Differential inflammatory responses of the native left and right ventricle associated with donor heart preservation
Source: Physiol Rep. 2021 Aug 26;9(17):e15004. doi: 10.14814/phy2.15004 (PMC8387788; doi:10.14814/phy2.15004)
Supplement: Supplementary file 1 — Fig S1‐S2 [file PHY2-9-e15004-s002.docx]

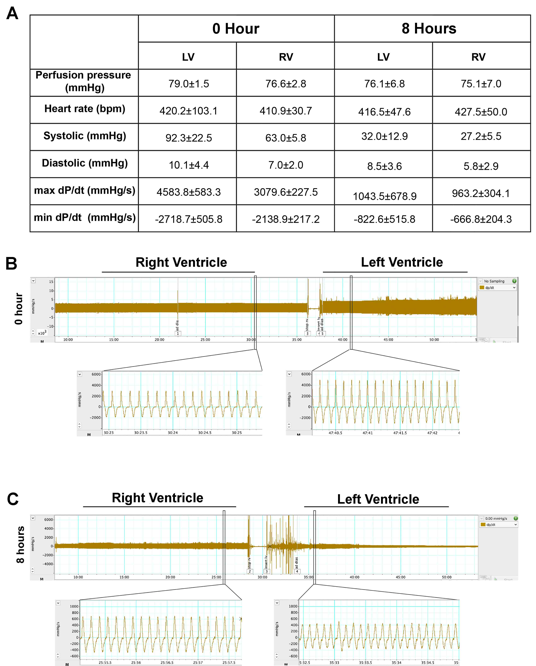
**Supplementary figure 1.** Ex-vivo perfusion hemodynamics are tabulated (a) including perfusion pressure, heart rate, ventricular cavitary pressure in systole and diastole as well as maximum (max) and minimum (min) dp/dt. (b) The ratio of max and min dp/dt in the RV compared to the LV of the same heart at time 0h and 8h are shown. ***P<0.001, Wilcoxon ranked sum test. (c) Representative balloon transduced ventricular cavitary pressure tracings of the RV and LV are presented.

**
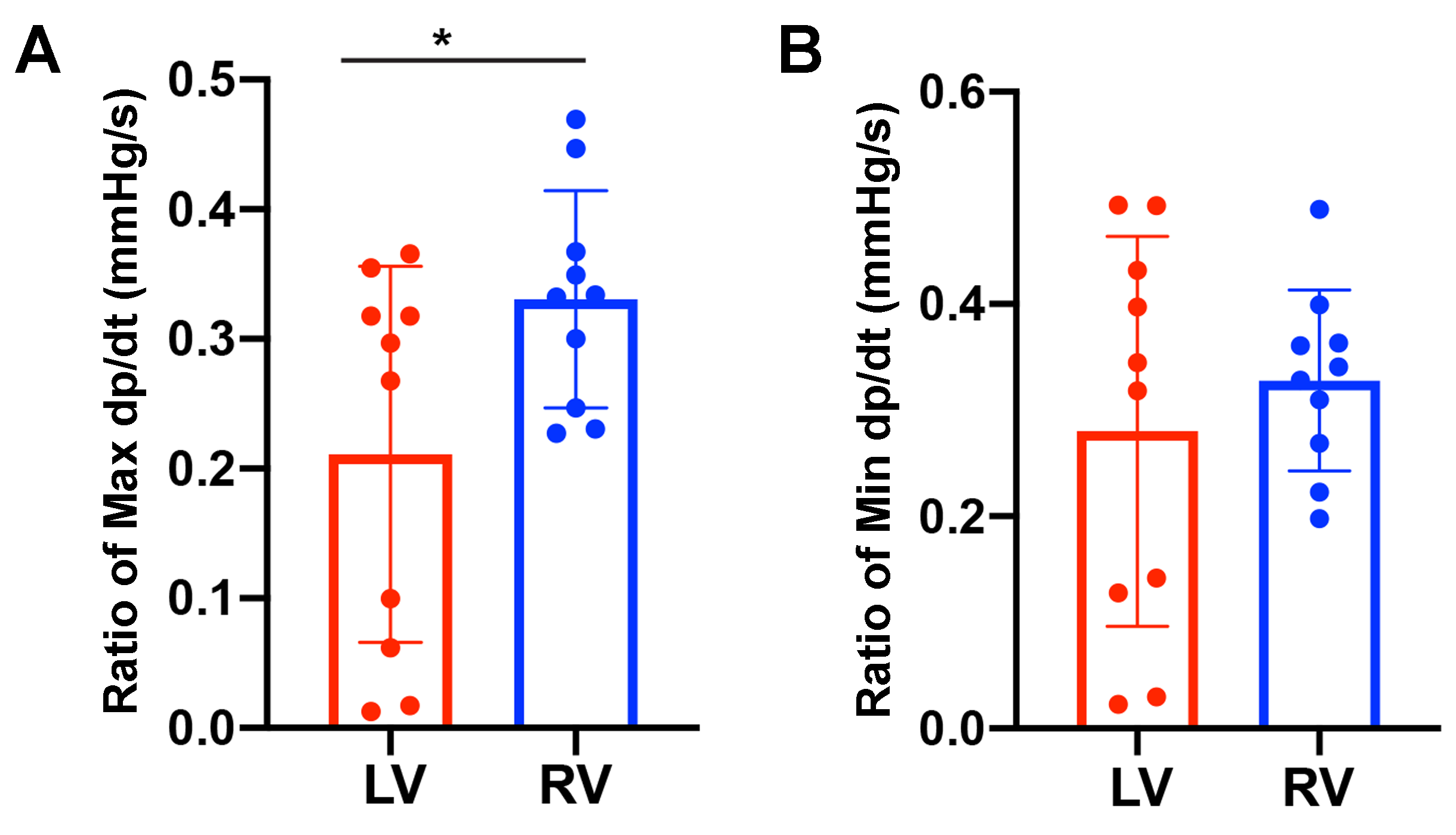
**

**Supplementary figure 2.** Murine donor hearts preserved in HTK with subsequent ex-vivo reperfusion­. The 8 over 0 hour ratio of balloon transduced intracavitary pressures from the LV (red) and RV (blue). n=10-11 at each time point.
