# Supplementary material for: Differential inflammatory responses of the native left and right ventricle associated with donor heart preservation
Source: Physiol Rep. 2021 Aug 26;9(17):e15004. doi: 10.14814/phy2.15004 (PMC8387788; doi:10.14814/phy2.15004)
Supplement: Supplementary file 2 — Table S1 [file PHY2-9-e15004-s001.docx]

LV4h vs LV0h DEGs

|  | baseMean | log2FoldChange | lfcSE | stat | pvalue | padj |
| --- | --- | --- | --- | --- | --- | --- |
| Gm11518 | 100.88 | 24.00 | 4.79 | 5.02 | 5.26E-07 | 1.54E-05 |
| Gm2274 | 33.66 | 8.82 | 1.62 | 5.44 | 5.28E-08 | 1.86E-06 |
| Gm3500 | 13.93 | 7.22 | 1.80 | 4.00 | 6.35E-05 | 1.18E-03 |
| Rpl7-ps9 | 9.43 | 7.21 | 1.86 | 3.89 | 1.02E-04 | 1.79E-03 |
| Gm9892 | 11.51 | 6.56 | 1.81 | 3.63 | 2.81E-04 | 4.35E-03 |
| Gm9522 | 12.18 | 5.86 | 1.90 | 3.09 | 2.02E-03 | 2.28E-02 |
| 1700030C10Rik | 31.50 | 5.31 | 1.20 | 4.43 | 9.64E-06 | 2.20E-04 |
| Rps10 | 310.99 | 4.50 | 1.52 | 2.96 | 3.10E-03 | 3.23E-02 |
| Gm6161 | 15.78 | 3.42 | 1.09 | 3.13 | 1.76E-03 | 2.06E-02 |
| Gm20257 | 17.09 | 3.38 | 1.20 | 2.82 | 4.85E-03 | 4.62E-02 |
| Obscn | 732.97 | 3.37 | 1.20 | 2.80 | 5.15E-03 | 4.84E-02 |
| Pcdhgc4 | 154.54 | 3.26 | 1.20 | 2.71 | 6.66E-03 | 5.88E-02 |
| Tnfaip8l2 | 15.64 | 2.95 | 0.94 | 3.15 | 1.65E-03 | 1.95E-02 |
| Dbp | 1139.57 | 2.89 | 0.18 | 15.89 | 7.28E-57 | 1.98E-53 |
| Per3 | 568.76 | 2.81 | 0.24 | 11.91 | 1.09E-32 | 5.31E-30 |
| Gm42715 | 240.34 | 2.71 | 0.95 | 2.85 | 4.44E-03 | 4.33E-02 |
| Slc22a17 | 60.06 | 2.53 | 0.55 | 4.56 | 5.00E-06 | 1.23E-04 |
| Gpr34 | 31.61 | 2.50 | 0.72 | 3.45 | 5.57E-04 | 7.85E-03 |
| Vstm5 | 20.41 | 2.27 | 0.79 | 2.87 | 4.07E-03 | 4.03E-02 |
| I830077J02Rik | 20.01 | 2.26 | 0.80 | 2.83 | 4.62E-03 | 4.46E-02 |
| Lrrc70 | 23.02 | 2.26 | 0.76 | 2.97 | 2.95E-03 | 3.10E-02 |
| P2ry13 | 24.15 | 2.24 | 0.74 | 3.01 | 2.61E-03 | 2.80E-02 |
| Zfp760 | 49.19 | 2.15 | 0.57 | 3.79 | 1.50E-04 | 2.54E-03 |
| Gm12258 | 32.40 | 2.14 | 0.64 | 3.34 | 8.36E-04 | 1.11E-02 |
| Gm29609 | 43.69 | 2.13 | 0.74 | 2.90 | 3.78E-03 | 3.80E-02 |
| Gm45647 | 20.77 | 2.12 | 0.82 | 2.61 | 9.17E-03 | 7.57E-02 |
| Gm5559 | 21.92 | 2.09 | 0.76 | 2.74 | 6.21E-03 | 5.58E-02 |
| Gm29013 | 1006.06 | 2.08 | 0.61 | 3.40 | 6.79E-04 | 9.29E-03 |
| Egfl7 | 658.47 | 2.08 | 0.25 | 8.40 | 4.57E-17 | 5.06E-15 |
| Zfp273 | 26.67 | 2.08 | 0.78 | 2.65 | 8.09E-03 | 6.88E-02 |
| Rab11fip3 | 72.22 | 2.08 | 0.56 | 3.73 | 1.95E-04 | 3.21E-03 |
| Zfp72 | 64.80 | 2.07 | 0.45 | 4.59 | 4.40E-06 | 1.09E-04 |
| Gstt2 | 187.23 | 2.06 | 0.30 | 6.76 | 1.40E-11 | 8.42E-10 |
| Tedc1 | 24.27 | 1.92 | 0.73 | 2.62 | 8.76E-03 | 7.31E-02 |
| Clec14a | 352.75 | 1.92 | 0.35 | 5.53 | 3.19E-08 | 1.17E-06 |
| Izumo4 | 52.97 | 1.90 | 0.51 | 3.74 | 1.87E-04 | 3.09E-03 |
| Tcf23 | 55.09 | 1.85 | 0.62 | 2.99 | 2.83E-03 | 2.99E-02 |
| Zbtb14 | 82.94 | 1.84 | 0.52 | 3.56 | 3.71E-04 | 5.53E-03 |
| Ephx3 | 26.78 | 1.84 | 0.70 | 2.64 | 8.25E-03 | 6.97E-02 |
| Flt3l | 165.08 | 1.83 | 0.30 | 6.04 | 1.55E-09 | 6.98E-08 |
| Kctd12b | 287.60 | 1.82 | 0.37 | 4.97 | 6.58E-07 | 1.90E-05 |
| Stk38 | 95.09 | 1.81 | 0.41 | 4.42 | 9.90E-06 | 2.25E-04 |
| Olfr78 | 45.30 | 1.80 | 0.50 | 3.59 | 3.27E-04 | 4.98E-03 |
| Taf9 | 37.48 | 1.78 | 0.59 | 3.02 | 2.52E-03 | 2.73E-02 |
| Dynlt1c | 158.28 | 1.75 | 0.31 | 5.70 | 1.17E-08 | 4.60E-07 |
| Eri2 | 62.61 | 1.74 | 0.57 | 3.02 | 2.54E-03 | 2.75E-02 |
| Pla2g4b | 97.48 | 1.73 | 0.43 | 4.02 | 5.87E-05 | 1.10E-03 |
| Zfp108 | 29.38 | 1.72 | 0.67 | 2.56 | 1.06E-02 | 8.43E-02 |
| Spaar | 113.15 | 1.72 | 0.36 | 4.72 | 2.31E-06 | 6.03E-05 |
| Zfp169 | 79.47 | 1.71 | 0.54 | 3.19 | 1.44E-03 | 1.73E-02 |
| Ighm | 104.38 | 1.70 | 0.46 | 3.69 | 2.22E-04 | 3.58E-03 |
| Nemp2 | 60.92 | 1.68 | 0.65 | 2.60 | 9.40E-03 | 7.69E-02 |
| Gpr160 | 39.88 | 1.68 | 0.61 | 2.76 | 5.73E-03 | 5.25E-02 |
| Bcl11b | 36.43 | 1.67 | 0.62 | 2.68 | 7.36E-03 | 6.38E-02 |
| Kif21b | 33.65 | 1.67 | 0.65 | 2.58 | 9.98E-03 | 8.03E-02 |
| Cys1 | 120.48 | 1.64 | 0.40 | 4.14 | 3.43E-05 | 6.87E-04 |
| Pcyox1l | 43.58 | 1.62 | 0.50 | 3.21 | 1.32E-03 | 1.61E-02 |
| Myct1 | 370.54 | 1.61 | 0.29 | 5.61 | 2.00E-08 | 7.66E-07 |
| Grhl2 | 54.52 | 1.60 | 0.48 | 3.34 | 8.28E-04 | 1.10E-02 |
| Efcc1 | 45.49 | 1.60 | 0.55 | 2.88 | 3.99E-03 | 3.97E-02 |
| Naa60 | 60.99 | 1.59 | 0.46 | 3.46 | 5.50E-04 | 7.80E-03 |
| Olfr558 | 96.55 | 1.57 | 0.44 | 3.56 | 3.70E-04 | 5.53E-03 |
| Pdik1l | 89.64 | 1.57 | 0.41 | 3.82 | 1.32E-04 | 2.27E-03 |
| Ryr3 | 40.44 | 1.56 | 0.54 | 2.88 | 4.00E-03 | 3.98E-02 |
| Nr2f2 | 263.83 | 1.54 | 0.29 | 5.39 | 7.16E-08 | 2.45E-06 |
| Cysltr1 | 51.51 | 1.52 | 0.61 | 2.49 | 1.28E-02 | 9.73E-02 |
| Gm42688 | 65.27 | 1.50 | 0.46 | 3.23 | 1.22E-03 | 1.52E-02 |
| Rps6kb2 | 269.44 | 1.50 | 0.25 | 5.97 | 2.32E-09 | 1.02E-07 |
| Hpgd | 118.50 | 1.48 | 0.41 | 3.65 | 2.64E-04 | 4.13E-03 |
| Aqp7 | 454.03 | 1.48 | 0.31 | 4.72 | 2.35E-06 | 6.11E-05 |
| Zfp763 | 58.72 | 1.47 | 0.57 | 2.58 | 9.88E-03 | 7.97E-02 |
| Tbx2 | 211.84 | 1.46 | 0.30 | 4.93 | 8.29E-07 | 2.35E-05 |
| Zfp112 | 100.82 | 1.45 | 0.36 | 4.02 | 5.77E-05 | 1.09E-03 |
| Zfp39 | 66.52 | 1.43 | 0.43 | 3.33 | 8.69E-04 | 1.15E-02 |
| Il2ra | 52.64 | 1.43 | 0.50 | 2.87 | 4.05E-03 | 4.02E-02 |
| Slc25a45 | 87.29 | 1.41 | 0.43 | 3.28 | 1.04E-03 | 1.33E-02 |
| Inka1 | 64.67 | 1.40 | 0.45 | 3.12 | 1.84E-03 | 2.12E-02 |
| Sertad3 | 50.78 | 1.39 | 0.49 | 2.83 | 4.68E-03 | 4.49E-02 |
| Tmem229b | 107.89 | 1.39 | 0.37 | 3.76 | 1.67E-04 | 2.80E-03 |
| Rtl5 | 52.92 | 1.38 | 0.49 | 2.84 | 4.45E-03 | 4.34E-02 |
| Prrt2 | 33.22 | 1.38 | 0.55 | 2.49 | 1.27E-02 | 9.67E-02 |
| Ahr | 198.52 | 1.38 | 0.29 | 4.74 | 2.19E-06 | 5.74E-05 |
| Tor4a | 105.23 | 1.38 | 0.43 | 3.17 | 1.53E-03 | 1.83E-02 |
| Gm2026 | 92.23 | 1.37 | 0.50 | 2.73 | 6.34E-03 | 5.65E-02 |
| Gm6158 | 101.68 | 1.36 | 0.50 | 2.74 | 6.23E-03 | 5.59E-02 |
| Nup210 | 147.85 | 1.35 | 0.45 | 3.01 | 2.62E-03 | 2.81E-02 |
| Adam1a | 88.58 | 1.35 | 0.52 | 2.60 | 9.28E-03 | 7.64E-02 |
| Ccdc8 | 133.09 | 1.34 | 0.36 | 3.70 | 2.12E-04 | 3.45E-03 |
| Pcsk4 | 67.57 | 1.34 | 0.47 | 2.85 | 4.43E-03 | 4.33E-02 |
| Gm48348 | 116.73 | 1.34 | 0.35 | 3.78 | 1.58E-04 | 2.67E-03 |
| Anpep | 316.62 | 1.33 | 0.25 | 5.30 | 1.16E-07 | 3.77E-06 |
| Rfc5 | 60.61 | 1.33 | 0.53 | 2.50 | 1.23E-02 | 9.39E-02 |
| Jrk | 68.32 | 1.33 | 0.42 | 3.17 | 1.50E-03 | 1.80E-02 |
| Calhm2 | 83.34 | 1.33 | 0.47 | 2.85 | 4.31E-03 | 4.23E-02 |
| Zfp606 | 202.07 | 1.33 | 0.30 | 4.43 | 9.34E-06 | 2.14E-04 |
| A430033K04Rik | 77.12 | 1.32 | 0.41 | 3.22 | 1.27E-03 | 1.56E-02 |
| Calcoco1 | 332.20 | 1.32 | 0.39 | 3.40 | 6.62E-04 | 9.08E-03 |
| Ap1g2 | 149.53 | 1.32 | 0.38 | 3.49 | 4.87E-04 | 6.99E-03 |
| Rasl12 | 191.16 | 1.31 | 0.29 | 4.53 | 6.03E-06 | 1.45E-04 |
| Smad9 | 46.89 | 1.30 | 0.51 | 2.53 | 1.15E-02 | 8.99E-02 |
| Iffo1 | 314.90 | 1.29 | 0.32 | 4.05 | 5.09E-05 | 9.75E-04 |
| Zfp867 | 130.81 | 1.29 | 0.36 | 3.54 | 3.93E-04 | 5.80E-03 |
| Zfp709 | 105.86 | 1.28 | 0.34 | 3.72 | 2.00E-04 | 3.28E-03 |
| Stard9 | 105.90 | 1.27 | 0.40 | 3.21 | 1.34E-03 | 1.63E-02 |
| Pqlc3 | 101.46 | 1.27 | 0.36 | 3.55 | 3.92E-04 | 5.79E-03 |
| Dipk2b | 174.47 | 1.27 | 0.32 | 3.97 | 7.17E-05 | 1.31E-03 |
| Zfp974 | 63.37 | 1.27 | 0.50 | 2.51 | 1.21E-02 | 9.32E-02 |
| Zfp280c | 190.67 | 1.26 | 0.39 | 3.19 | 1.41E-03 | 1.71E-02 |
| Hspa12b | 972.96 | 1.26 | 0.22 | 5.81 | 6.25E-09 | 2.57E-07 |
| Klhl42 | 117.54 | 1.25 | 0.47 | 2.64 | 8.28E-03 | 6.99E-02 |
| Tril | 153.60 | 1.25 | 0.35 | 3.61 | 3.11E-04 | 4.77E-03 |
| Sigirr | 149.93 | 1.23 | 0.33 | 3.70 | 2.18E-04 | 3.52E-03 |
| Frs3 | 98.83 | 1.23 | 0.41 | 3.01 | 2.61E-03 | 2.80E-02 |
| Mmp28 | 115.37 | 1.23 | 0.34 | 3.65 | 2.63E-04 | 4.12E-03 |
| Kank3 | 1358.27 | 1.22 | 0.27 | 4.50 | 6.95E-06 | 1.64E-04 |
| 1700030K09Rik | 138.37 | 1.22 | 0.36 | 3.37 | 7.52E-04 | 1.01E-02 |
| Mtcp1 | 59.28 | 1.21 | 0.48 | 2.53 | 1.13E-02 | 8.83E-02 |
| Golga1 | 440.37 | 1.20 | 0.23 | 5.30 | 1.15E-07 | 3.77E-06 |
| Lmod1 | 60.37 | 1.20 | 0.44 | 2.72 | 6.52E-03 | 5.79E-02 |
| Gja5 | 101.91 | 1.20 | 0.34 | 3.56 | 3.70E-04 | 5.53E-03 |
| Ripply3 | 83.33 | 1.20 | 0.43 | 2.76 | 5.78E-03 | 5.29E-02 |
| Gstt3 | 73.13 | 1.20 | 0.43 | 2.76 | 5.74E-03 | 5.26E-02 |
| Gm10443 | 196.17 | 1.20 | 0.34 | 3.50 | 4.65E-04 | 6.73E-03 |
| Adprm | 155.88 | 1.20 | 0.32 | 3.74 | 1.86E-04 | 3.07E-03 |
| D630003M21Rik | 244.02 | 1.19 | 0.33 | 3.56 | 3.70E-04 | 5.53E-03 |
| Asb7 | 185.98 | 1.19 | 0.36 | 3.27 | 1.07E-03 | 1.36E-02 |
| Arrdc1 | 104.55 | 1.18 | 0.38 | 3.13 | 1.77E-03 | 2.06E-02 |
| Asb14 | 435.53 | 1.17 | 0.27 | 4.39 | 1.11E-05 | 2.51E-04 |
| AC149090.1 | 881.21 | 1.17 | 0.22 | 5.31 | 1.07E-07 | 3.53E-06 |
| Cxcl12 | 3927.90 | 1.17 | 0.18 | 6.61 | 3.75E-11 | 2.15E-09 |
| Plscr4 | 290.64 | 1.17 | 0.24 | 4.91 | 9.00E-07 | 2.54E-05 |
| Tef | 1543.13 | 1.16 | 0.19 | 6.09 | 1.14E-09 | 5.26E-08 |
| Hic1 | 267.78 | 1.16 | 0.38 | 3.04 | 2.36E-03 | 2.59E-02 |
| Palm | 922.62 | 1.15 | 0.30 | 3.88 | 1.06E-04 | 1.85E-03 |
| Zfp963 | 61.26 | 1.15 | 0.45 | 2.54 | 1.12E-02 | 8.76E-02 |
| Ptpn18 | 66.66 | 1.14 | 0.43 | 2.68 | 7.40E-03 | 6.40E-02 |
| Fn3krp | 107.74 | 1.14 | 0.38 | 2.97 | 3.00E-03 | 3.14E-02 |
| Cpt1c | 103.16 | 1.13 | 0.35 | 3.24 | 1.19E-03 | 1.49E-02 |
| Thap3 | 196.27 | 1.13 | 0.27 | 4.18 | 2.91E-05 | 5.93E-04 |
| Fibin | 229.24 | 1.13 | 0.41 | 2.77 | 5.62E-03 | 5.19E-02 |
| Shld1 | 547.45 | 1.12 | 0.23 | 4.95 | 7.41E-07 | 2.12E-05 |
| Zfp748 | 75.71 | 1.12 | 0.45 | 2.50 | 1.23E-02 | 9.39E-02 |
| Cyp4f16 | 140.23 | 1.11 | 0.33 | 3.40 | 6.70E-04 | 9.18E-03 |
| Rassf9 | 219.72 | 1.11 | 0.28 | 3.97 | 7.14E-05 | 1.31E-03 |
| Sybu | 148.43 | 1.11 | 0.37 | 3.03 | 2.47E-03 | 2.69E-02 |
| Tesmin | 74.95 | 1.10 | 0.45 | 2.47 | 1.34E-02 | 9.98E-02 |
| Hlf | 492.94 | 1.10 | 0.22 | 4.90 | 9.64E-07 | 2.69E-05 |
| Ctu1 | 58.11 | 1.10 | 0.44 | 2.48 | 1.33E-02 | 9.95E-02 |
| Sertad4 | 84.30 | 1.09 | 0.43 | 2.54 | 1.10E-02 | 8.70E-02 |
| AW146154 | 77.01 | 1.09 | 0.42 | 2.58 | 1.00E-02 | 8.03E-02 |
| Zkscan8 | 244.59 | 1.08 | 0.35 | 3.10 | 1.90E-03 | 2.18E-02 |
| Zfp944 | 129.39 | 1.08 | 0.34 | 3.22 | 1.30E-03 | 1.60E-02 |
| Alox12 | 105.09 | 1.08 | 0.38 | 2.83 | 4.59E-03 | 4.43E-02 |
| Zfp157 | 112.70 | 1.07 | 0.33 | 3.23 | 1.22E-03 | 1.52E-02 |
| Rbak | 127.81 | 1.07 | 0.37 | 2.92 | 3.52E-03 | 3.57E-02 |
| Lysmd1 | 131.60 | 1.06 | 0.32 | 3.37 | 7.62E-04 | 1.02E-02 |
| Gucy1a1 | 434.44 | 1.06 | 0.23 | 4.57 | 4.85E-06 | 1.19E-04 |
| Ocel1 | 265.27 | 1.06 | 0.28 | 3.77 | 1.65E-04 | 2.76E-03 |
| Fbxo9 | 613.06 | 1.05 | 0.19 | 5.53 | 3.27E-08 | 1.20E-06 |
| Sv2a | 66.99 | 1.05 | 0.42 | 2.51 | 1.21E-02 | 9.33E-02 |
| Lysmd4 | 294.81 | 1.05 | 0.27 | 3.93 | 8.64E-05 | 1.56E-03 |
| Rap1gap | 459.35 | 1.04 | 0.36 | 2.90 | 3.70E-03 | 3.73E-02 |
| Fbxl12 | 287.83 | 1.04 | 0.25 | 4.10 | 4.13E-05 | 8.10E-04 |
| Heyl | 558.14 | 1.04 | 0.29 | 3.62 | 2.89E-04 | 4.45E-03 |
| Mfng | 186.81 | 1.04 | 0.29 | 3.61 | 3.09E-04 | 4.75E-03 |
| Il16 | 139.95 | 1.03 | 0.33 | 3.09 | 1.98E-03 | 2.25E-02 |
| Ankzf1 | 480.07 | 1.03 | 0.20 | 5.13 | 2.86E-07 | 8.84E-06 |
| St6galnac2 | 240.67 | 1.03 | 0.28 | 3.70 | 2.15E-04 | 3.49E-03 |
| Ebpl | 117.55 | 1.02 | 0.35 | 2.92 | 3.48E-03 | 3.54E-02 |
| Zfp825 | 183.42 | 1.02 | 0.32 | 3.18 | 1.46E-03 | 1.76E-02 |
| Zfp512b | 538.03 | 1.02 | 0.22 | 4.59 | 4.35E-06 | 1.08E-04 |
| Wdr24 | 318.23 | 1.02 | 0.25 | 4.08 | 4.53E-05 | 8.77E-04 |
| Nek8 | 97.64 | 1.02 | 0.41 | 2.49 | 1.29E-02 | 9.77E-02 |
| Pld4 | 178.20 | 1.02 | 0.29 | 3.51 | 4.46E-04 | 6.49E-03 |
| Gask1b | 854.43 | 1.02 | 0.35 | 2.87 | 4.10E-03 | 4.06E-02 |
| Clcn1 | 298.76 | 1.01 | 0.24 | 4.27 | 1.99E-05 | 4.23E-04 |
| Pik3r2 | 539.54 | 1.01 | 0.19 | 5.23 | 1.68E-07 | 5.41E-06 |
| Zfp799 | 190.71 | 1.00 | 0.33 | 2.99 | 2.75E-03 | 2.92E-02 |
| Slc35c1 | 113.23 | 1.00 | 0.34 | 2.90 | 3.78E-03 | 3.80E-02 |
| Arhgef15 | 1673.43 | 1.00 | 0.20 | 4.91 | 9.02E-07 | 2.54E-05 |
| Map11 | 163.18 | 0.99 | 0.30 | 3.31 | 9.44E-04 | 1.23E-02 |
| Gimap1 | 145.34 | 0.99 | 0.36 | 2.74 | 6.17E-03 | 5.56E-02 |
| Dcaf17 | 170.45 | 0.99 | 0.32 | 3.06 | 2.23E-03 | 2.48E-02 |
| Bok | 100.96 | 0.99 | 0.39 | 2.53 | 1.14E-02 | 8.92E-02 |
| Zfp788 | 221.22 | 0.99 | 0.31 | 3.16 | 1.58E-03 | 1.88E-02 |
| Zfp958 | 119.42 | 0.99 | 0.33 | 2.95 | 3.21E-03 | 3.31E-02 |
| Ston1 | 220.16 | 0.99 | 0.26 | 3.87 | 1.10E-04 | 1.92E-03 |
| Fam219b | 299.96 | 0.98 | 0.29 | 3.39 | 6.92E-04 | 9.43E-03 |
| Ttc30b | 337.68 | 0.98 | 0.31 | 3.21 | 1.32E-03 | 1.61E-02 |
| Pycard | 167.43 | 0.98 | 0.36 | 2.75 | 5.87E-03 | 5.35E-02 |
| Pcdh18 | 120.08 | 0.98 | 0.33 | 2.96 | 3.04E-03 | 3.18E-02 |
| St8sia4 | 348.06 | 0.98 | 0.30 | 3.21 | 1.31E-03 | 1.61E-02 |
| Ajuba | 167.62 | 0.98 | 0.28 | 3.44 | 5.87E-04 | 8.20E-03 |
| Tmem204 | 850.30 | 0.98 | 0.23 | 4.24 | 2.24E-05 | 4.70E-04 |
| Zfp395 | 317.31 | 0.97 | 0.36 | 2.69 | 7.04E-03 | 6.16E-02 |
| Ccdc28b | 166.69 | 0.97 | 0.34 | 2.84 | 4.52E-03 | 4.39E-02 |
| Per2 | 510.23 | 0.97 | 0.34 | 2.89 | 3.84E-03 | 3.84E-02 |
| Eml3 | 314.24 | 0.97 | 0.27 | 3.64 | 2.74E-04 | 4.26E-03 |
| Slx1b | 151.03 | 0.97 | 0.31 | 3.11 | 1.84E-03 | 2.12E-02 |
| H2-T24 | 199.74 | 0.97 | 0.30 | 3.20 | 1.37E-03 | 1.67E-02 |
| Ttf1 | 306.40 | 0.96 | 0.27 | 3.54 | 4.01E-04 | 5.90E-03 |
| Tchp | 196.50 | 0.96 | 0.26 | 3.68 | 2.31E-04 | 3.70E-03 |
| Acbd4 | 328.24 | 0.96 | 0.23 | 4.09 | 4.31E-05 | 8.38E-04 |
| Lims2 | 1564.94 | 0.95 | 0.27 | 3.59 | 3.31E-04 | 5.01E-03 |
| Pdlim2 | 135.82 | 0.95 | 0.34 | 2.78 | 5.44E-03 | 5.06E-02 |
| Stx2 | 408.45 | 0.95 | 0.22 | 4.28 | 1.90E-05 | 4.07E-04 |
| Ky | 351.60 | 0.95 | 0.37 | 2.57 | 1.01E-02 | 8.11E-02 |
| Zfp74 | 156.70 | 0.95 | 0.31 | 3.07 | 2.13E-03 | 2.38E-02 |
| Slc46a3 | 239.17 | 0.95 | 0.29 | 3.29 | 9.89E-04 | 1.27E-02 |
| Pik3ip1 | 1139.41 | 0.94 | 0.20 | 4.82 | 1.44E-06 | 3.90E-05 |
| Grk4 | 102.73 | 0.94 | 0.36 | 2.59 | 9.55E-03 | 7.78E-02 |
| 0610030E20Rik | 343.00 | 0.94 | 0.26 | 3.61 | 3.12E-04 | 4.77E-03 |
| Zfp626 | 166.14 | 0.94 | 0.31 | 3.06 | 2.19E-03 | 2.44E-02 |
| Tspan13 | 1105.60 | 0.94 | 0.22 | 4.31 | 1.64E-05 | 3.56E-04 |
| Nrep | 206.01 | 0.94 | 0.29 | 3.25 | 1.15E-03 | 1.44E-02 |
| Ints6l | 444.18 | 0.94 | 0.25 | 3.71 | 2.03E-04 | 3.32E-03 |
| Eps8l2 | 96.32 | 0.94 | 0.36 | 2.61 | 8.97E-03 | 7.44E-02 |
| Casp2 | 157.03 | 0.93 | 0.31 | 3.00 | 2.66E-03 | 2.85E-02 |
| Trib2 | 599.99 | 0.93 | 0.25 | 3.68 | 2.32E-04 | 3.71E-03 |
| Helq | 114.29 | 0.93 | 0.34 | 2.74 | 6.06E-03 | 5.49E-02 |
| Fam57b | 201.96 | 0.93 | 0.26 | 3.56 | 3.70E-04 | 5.53E-03 |
| Fam13c | 175.60 | 0.93 | 0.29 | 3.18 | 1.47E-03 | 1.77E-02 |
| Zfp612 | 442.83 | 0.93 | 0.22 | 4.14 | 3.47E-05 | 6.93E-04 |
| Gm49396 | 192.19 | 0.92 | 0.34 | 2.68 | 7.39E-03 | 6.40E-02 |
| Thsd1 | 154.56 | 0.92 | 0.36 | 2.57 | 1.02E-02 | 8.14E-02 |
| Fmo1 | 1058.34 | 0.92 | 0.19 | 4.79 | 1.66E-06 | 4.46E-05 |
| Kcnj8 | 958.05 | 0.91 | 0.18 | 5.10 | 3.47E-07 | 1.05E-05 |
| Gsta3 | 156.18 | 0.91 | 0.30 | 3.07 | 2.15E-03 | 2.39E-02 |
| Pth1r | 120.00 | 0.91 | 0.32 | 2.87 | 4.07E-03 | 4.03E-02 |
| Letm2 | 204.09 | 0.90 | 0.33 | 2.73 | 6.32E-03 | 5.65E-02 |
| B3galt2 | 289.76 | 0.90 | 0.29 | 3.07 | 2.11E-03 | 2.36E-02 |
| Zfp507 | 325.51 | 0.90 | 0.35 | 2.54 | 1.10E-02 | 8.72E-02 |
| Dvl2 | 155.34 | 0.90 | 0.28 | 3.20 | 1.37E-03 | 1.67E-02 |
| Tmc6 | 332.37 | 0.89 | 0.31 | 2.84 | 4.45E-03 | 4.34E-02 |
| Rufy2 | 182.38 | 0.89 | 0.30 | 3.01 | 2.65E-03 | 2.84E-02 |
| Mapk14 | 548.10 | 0.88 | 0.26 | 3.41 | 6.49E-04 | 8.93E-03 |
| Slc26a10 | 891.08 | 0.88 | 0.24 | 3.75 | 1.79E-04 | 2.98E-03 |
| Uvssa | 316.11 | 0.88 | 0.26 | 3.35 | 7.94E-04 | 1.06E-02 |
| Sema6c | 161.93 | 0.88 | 0.31 | 2.82 | 4.86E-03 | 4.63E-02 |
| Adamts7 | 795.17 | 0.87 | 0.17 | 5.05 | 4.34E-07 | 1.30E-05 |
| Tcea2 | 117.33 | 0.87 | 0.33 | 2.65 | 8.05E-03 | 6.85E-02 |
| Pi16 | 672.41 | 0.87 | 0.20 | 4.42 | 9.86E-06 | 2.25E-04 |
| Vsig2 | 186.02 | 0.87 | 0.31 | 2.81 | 4.94E-03 | 4.69E-02 |
| Wscd1 | 145.84 | 0.86 | 0.31 | 2.77 | 5.64E-03 | 5.20E-02 |
| Slc2a4rg-ps | 181.67 | 0.86 | 0.32 | 2.73 | 6.43E-03 | 5.72E-02 |
| Nynrin | 139.57 | 0.86 | 0.32 | 2.68 | 7.30E-03 | 6.33E-02 |
| Fat4 | 366.85 | 0.86 | 0.25 | 3.48 | 5.06E-04 | 7.24E-03 |
| Adck2 | 301.20 | 0.85 | 0.32 | 2.65 | 7.93E-03 | 6.77E-02 |
| Snx33 | 195.07 | 0.85 | 0.27 | 3.12 | 1.83E-03 | 2.12E-02 |
| Zfp780b | 156.39 | 0.84 | 0.33 | 2.52 | 1.18E-02 | 9.12E-02 |
| Akr1c14 | 216.68 | 0.84 | 0.27 | 3.06 | 2.24E-03 | 2.48E-02 |
| Ndor1 | 323.26 | 0.84 | 0.27 | 3.08 | 2.04E-03 | 2.30E-02 |
| Krba1 | 952.79 | 0.84 | 0.17 | 4.88 | 1.04E-06 | 2.89E-05 |
| Cnot3 | 631.97 | 0.83 | 0.19 | 4.30 | 1.69E-05 | 3.66E-04 |
| Hdac7 | 1499.93 | 0.83 | 0.23 | 3.69 | 2.23E-04 | 3.58E-03 |
| Pias3 | 288.13 | 0.83 | 0.29 | 2.83 | 4.68E-03 | 4.49E-02 |
| Podn | 425.27 | 0.83 | 0.21 | 3.94 | 8.15E-05 | 1.47E-03 |
| Ap4m1 | 312.45 | 0.83 | 0.32 | 2.55 | 1.06E-02 | 8.46E-02 |
| Cd300lg | 2548.28 | 0.83 | 0.23 | 3.54 | 4.03E-04 | 5.91E-03 |
| Taok2 | 1512.00 | 0.82 | 0.16 | 5.11 | 3.15E-07 | 9.62E-06 |
| Xndc1 | 225.85 | 0.82 | 0.27 | 3.02 | 2.56E-03 | 2.77E-02 |
| Cdk10 | 460.34 | 0.82 | 0.24 | 3.38 | 7.15E-04 | 9.68E-03 |
| Lgals4 | 1405.24 | 0.81 | 0.24 | 3.35 | 8.22E-04 | 1.10E-02 |
| Rtel1 | 301.71 | 0.81 | 0.26 | 3.13 | 1.77E-03 | 2.07E-02 |
| Pdp2 | 783.43 | 0.81 | 0.29 | 2.83 | 4.66E-03 | 4.48E-02 |
| Zfp862-ps | 131.98 | 0.81 | 0.32 | 2.55 | 1.09E-02 | 8.65E-02 |
| Ap3m2 | 128.75 | 0.81 | 0.33 | 2.48 | 1.30E-02 | 9.83E-02 |
| Sfi1 | 353.74 | 0.81 | 0.23 | 3.53 | 4.23E-04 | 6.18E-03 |
| Tubgcp5 | 254.63 | 0.81 | 0.27 | 2.95 | 3.18E-03 | 3.29E-02 |
| Dffb | 166.30 | 0.81 | 0.28 | 2.87 | 4.15E-03 | 4.10E-02 |
| Zfp292 | 616.68 | 0.81 | 0.23 | 3.52 | 4.31E-04 | 6.29E-03 |
| Plp1 | 166.51 | 0.80 | 0.30 | 2.66 | 7.75E-03 | 6.65E-02 |
| Abca8a | 2379.15 | 0.80 | 0.19 | 4.23 | 2.38E-05 | 4.97E-04 |
| Gm5113 | 328.51 | 0.80 | 0.22 | 3.55 | 3.80E-04 | 5.65E-03 |
| Klf15 | 1242.13 | 0.80 | 0.22 | 3.68 | 2.31E-04 | 3.70E-03 |
| Pde7a | 1425.62 | 0.80 | 0.26 | 3.11 | 1.87E-03 | 2.15E-02 |
| Stard8 | 1148.45 | 0.79 | 0.19 | 4.28 | 1.87E-05 | 4.01E-04 |
| Zfp3 | 123.82 | 0.79 | 0.31 | 2.51 | 1.19E-02 | 9.21E-02 |
| Aasdh | 447.48 | 0.78 | 0.24 | 3.32 | 9.02E-04 | 1.18E-02 |
| Cdan1 | 375.88 | 0.78 | 0.24 | 3.24 | 1.19E-03 | 1.49E-02 |
| Zfp866 | 344.81 | 0.78 | 0.24 | 3.23 | 1.24E-03 | 1.53E-02 |
| Vipas39 | 595.10 | 0.78 | 0.19 | 4.05 | 5.10E-05 | 9.76E-04 |
| Id1 | 857.67 | 0.78 | 0.28 | 2.81 | 5.03E-03 | 4.75E-02 |
| Gba2 | 542.79 | 0.77 | 0.20 | 3.78 | 1.58E-04 | 2.67E-03 |
| Usf1 | 513.24 | 0.77 | 0.24 | 3.15 | 1.63E-03 | 1.93E-02 |
| Gatd1 | 186.44 | 0.77 | 0.29 | 2.62 | 8.82E-03 | 7.34E-02 |
| Mcoln1 | 692.37 | 0.76 | 0.18 | 4.18 | 2.91E-05 | 5.93E-04 |
| Dok4 | 404.80 | 0.76 | 0.24 | 3.20 | 1.39E-03 | 1.69E-02 |
| Tada2a | 167.08 | 0.76 | 0.29 | 2.61 | 9.00E-03 | 7.46E-02 |
| Glt8d1 | 530.06 | 0.76 | 0.22 | 3.43 | 5.93E-04 | 8.27E-03 |
| Zfp444 | 197.65 | 0.76 | 0.28 | 2.68 | 7.43E-03 | 6.41E-02 |
| Usp21 | 427.37 | 0.76 | 0.23 | 3.32 | 8.93E-04 | 1.18E-02 |
| Prickle1 | 399.41 | 0.76 | 0.21 | 3.58 | 3.48E-04 | 5.24E-03 |
| Tlcd5 | 245.02 | 0.75 | 0.24 | 3.09 | 2.00E-03 | 2.27E-02 |
| Meox2 | 360.20 | 0.75 | 0.30 | 2.48 | 1.32E-02 | 9.89E-02 |
| Gipc3 | 168.43 | 0.75 | 0.30 | 2.47 | 1.34E-02 | 9.98E-02 |
| Cyb561d1 | 196.61 | 0.75 | 0.29 | 2.60 | 9.43E-03 | 7.71E-02 |
| Ephx1 | 932.03 | 0.75 | 0.28 | 2.69 | 7.18E-03 | 6.26E-02 |
| Fbxl20 | 944.20 | 0.75 | 0.21 | 3.63 | 2.88E-04 | 4.45E-03 |
| Acd | 242.04 | 0.75 | 0.26 | 2.89 | 3.82E-03 | 3.83E-02 |
| Gm10221 | 1238.11 | 0.74 | 0.30 | 2.51 | 1.21E-02 | 9.33E-02 |
| Zfyve19 | 200.08 | 0.74 | 0.29 | 2.58 | 9.97E-03 | 8.03E-02 |
| Kat14 | 679.81 | 0.74 | 0.20 | 3.79 | 1.53E-04 | 2.60E-03 |
| Trim65 | 239.86 | 0.74 | 0.27 | 2.69 | 7.10E-03 | 6.19E-02 |
| Aldh9a1 | 1272.66 | 0.73 | 0.21 | 3.42 | 6.19E-04 | 8.58E-03 |
| Ptp4a3 | 770.10 | 0.73 | 0.27 | 2.74 | 6.10E-03 | 5.51E-02 |
| Kmt5c | 293.38 | 0.73 | 0.29 | 2.53 | 1.15E-02 | 8.96E-02 |
| Abtb1 | 532.74 | 0.73 | 0.19 | 3.80 | 1.46E-04 | 2.49E-03 |
| Wdr6 | 383.78 | 0.73 | 0.21 | 3.44 | 5.85E-04 | 8.19E-03 |
| Dpysl2 | 367.64 | 0.73 | 0.27 | 2.67 | 7.62E-03 | 6.56E-02 |
| Sirt4 | 284.86 | 0.73 | 0.23 | 3.18 | 1.49E-03 | 1.80E-02 |
| Ctc1 | 387.44 | 0.72 | 0.23 | 3.21 | 1.33E-03 | 1.63E-02 |
| Cnpy2 | 503.34 | 0.72 | 0.25 | 2.95 | 3.20E-03 | 3.31E-02 |
| Inpp5k | 779.24 | 0.72 | 0.20 | 3.53 | 4.17E-04 | 6.11E-03 |
| Tia1 | 738.70 | 0.72 | 0.17 | 4.15 | 3.34E-05 | 6.71E-04 |
| Rasgrp2 | 1134.84 | 0.72 | 0.22 | 3.30 | 9.60E-04 | 1.24E-02 |
| Fbxl18 | 287.45 | 0.72 | 0.28 | 2.52 | 1.16E-02 | 9.01E-02 |
| Ifi203-ps | 337.79 | 0.72 | 0.26 | 2.73 | 6.30E-03 | 5.64E-02 |
| Gga3 | 547.18 | 0.71 | 0.26 | 2.74 | 6.24E-03 | 5.59E-02 |
| Cep19 | 208.60 | 0.71 | 0.26 | 2.70 | 7.01E-03 | 6.14E-02 |
| Slc33a1 | 274.07 | 0.71 | 0.23 | 3.05 | 2.32E-03 | 2.56E-02 |
| Ermard | 294.00 | 0.71 | 0.24 | 2.99 | 2.78E-03 | 2.94E-02 |
| Thy1 | 422.12 | 0.71 | 0.25 | 2.81 | 4.96E-03 | 4.70E-02 |
| Gigyf1 | 1189.00 | 0.71 | 0.18 | 3.97 | 7.20E-05 | 1.32E-03 |
| Wdr81 | 391.43 | 0.71 | 0.21 | 3.34 | 8.49E-04 | 1.13E-02 |
| Pnpla6 | 361.26 | 0.71 | 0.24 | 2.94 | 3.27E-03 | 3.36E-02 |
| C1qtnf9 | 1018.33 | 0.71 | 0.18 | 3.86 | 1.14E-04 | 1.98E-03 |
| Ushbp1 | 2131.72 | 0.70 | 0.22 | 3.26 | 1.11E-03 | 1.41E-02 |
| Zfp329 | 204.94 | 0.70 | 0.27 | 2.59 | 9.47E-03 | 7.72E-02 |
| Ubxn4 | 2169.81 | -0.70 | 0.20 | -3.55 | 3.91E-04 | 5.78E-03 |
| Smarca5 | 1944.03 | -0.70 | 0.18 | -3.89 | 9.88E-05 | 1.75E-03 |
| Iffo2 | 368.85 | -0.70 | 0.26 | -2.73 | 6.33E-03 | 5.65E-02 |
| Ms4a6c | 204.77 | -0.71 | 0.28 | -2.53 | 1.13E-02 | 8.83E-02 |
| Mex3c | 593.15 | -0.71 | 0.20 | -3.55 | 3.81E-04 | 5.66E-03 |
| Suco | 595.71 | -0.71 | 0.24 | -3.01 | 2.60E-03 | 2.80E-02 |
| Slc39a1 | 2515.11 | -0.71 | 0.18 | -3.84 | 1.21E-04 | 2.10E-03 |
| Xiap | 1930.81 | -0.71 | 0.23 | -3.13 | 1.75E-03 | 2.05E-02 |
| Tbk1 | 791.92 | -0.71 | 0.19 | -3.66 | 2.51E-04 | 3.95E-03 |
| Osgin2 | 285.38 | -0.71 | 0.24 | -3.00 | 2.66E-03 | 2.85E-02 |
| C3 | 6778.62 | -0.71 | 0.29 | -2.50 | 1.25E-02 | 9.51E-02 |
| Slc23a2 | 903.66 | -0.72 | 0.21 | -3.36 | 7.80E-04 | 1.05E-02 |
| Sh3bp4 | 738.78 | -0.72 | 0.18 | -3.94 | 8.12E-05 | 1.47E-03 |
| Hspa1l | 330.12 | -0.72 | 0.26 | -2.73 | 6.31E-03 | 5.64E-02 |
| Ppp4r2 | 1624.70 | -0.72 | 0.17 | -4.15 | 3.33E-05 | 6.70E-04 |
| Fzd7 | 234.98 | -0.72 | 0.24 | -3.01 | 2.63E-03 | 2.82E-02 |
| Tagln2 | 3030.30 | -0.72 | 0.25 | -2.84 | 4.55E-03 | 4.41E-02 |
| Gm37240 | 182.69 | -0.72 | 0.27 | -2.71 | 6.63E-03 | 5.87E-02 |
| Plekhg2 | 1568.33 | -0.73 | 0.26 | -2.82 | 4.80E-03 | 4.58E-02 |
| Slc16a6 | 261.54 | -0.73 | 0.25 | -2.94 | 3.27E-03 | 3.36E-02 |
| St3gal1 | 1510.69 | -0.73 | 0.17 | -4.21 | 2.54E-05 | 5.26E-04 |
| Kctd6 | 308.29 | -0.73 | 0.25 | -2.94 | 3.33E-03 | 3.42E-02 |
| Cd9 | 1079.36 | -0.73 | 0.25 | -2.92 | 3.47E-03 | 3.54E-02 |
| Tmem120a | 386.23 | -0.73 | 0.23 | -3.11 | 1.89E-03 | 2.16E-02 |
| Agap1 | 426.68 | -0.73 | 0.24 | -3.03 | 2.45E-03 | 2.67E-02 |
| Colq | 338.04 | -0.73 | 0.26 | -2.81 | 4.96E-03 | 4.70E-02 |
| Brd2 | 1821.74 | -0.73 | 0.17 | -4.33 | 1.52E-05 | 3.34E-04 |
| Mlkl | 191.44 | -0.73 | 0.28 | -2.59 | 9.54E-03 | 7.77E-02 |
| Pak6 | 548.73 | -0.73 | 0.29 | -2.54 | 1.11E-02 | 8.73E-02 |
| Plekhg1 | 628.94 | -0.73 | 0.22 | -3.26 | 1.10E-03 | 1.39E-02 |
| Eps8 | 510.27 | -0.74 | 0.28 | -2.60 | 9.21E-03 | 7.59E-02 |
| Ckap4 | 1023.77 | -0.74 | 0.20 | -3.61 | 3.10E-04 | 4.75E-03 |
| Rapgef2 | 1497.95 | -0.74 | 0.21 | -3.55 | 3.88E-04 | 5.75E-03 |
| Nr4a2 | 534.28 | -0.74 | 0.27 | -2.74 | 6.20E-03 | 5.58E-02 |
| Vmp1 | 1726.91 | -0.74 | 0.19 | -3.81 | 1.39E-04 | 2.38E-03 |
| Cblb | 517.70 | -0.74 | 0.23 | -3.26 | 1.10E-03 | 1.40E-02 |
| Shroom3 | 1520.80 | -0.74 | 0.18 | -4.16 | 3.24E-05 | 6.54E-04 |
| Dennd2a | 417.82 | -0.74 | 0.25 | -2.95 | 3.22E-03 | 3.32E-02 |
| Hivep2 | 1552.45 | -0.74 | 0.15 | -5.00 | 5.87E-07 | 1.71E-05 |
| Etv5 | 422.04 | -0.74 | 0.28 | -2.64 | 8.27E-03 | 6.98E-02 |
| Frmd4a | 1102.20 | -0.74 | 0.21 | -3.48 | 5.06E-04 | 7.24E-03 |
| Dusp18 | 2125.92 | -0.74 | 0.26 | -2.86 | 4.24E-03 | 4.17E-02 |
| Zranb1 | 1787.19 | -0.74 | 0.19 | -3.98 | 6.94E-05 | 1.28E-03 |
| Itgav | 852.46 | -0.74 | 0.21 | -3.45 | 5.57E-04 | 7.86E-03 |
| Fam111a | 296.25 | -0.74 | 0.29 | -2.58 | 9.82E-03 | 7.94E-02 |
| Ppp1r9b | 2088.99 | -0.74 | 0.19 | -3.87 | 1.10E-04 | 1.92E-03 |
| Akna | 352.05 | -0.74 | 0.27 | -2.73 | 6.42E-03 | 5.72E-02 |
| Wapl | 1455.93 | -0.74 | 0.15 | -4.88 | 1.07E-06 | 2.98E-05 |
| Tma16 | 338.06 | -0.75 | 0.21 | -3.50 | 4.68E-04 | 6.75E-03 |
| Col4a2 | 15950.87 | -0.75 | 0.16 | -4.71 | 2.50E-06 | 6.47E-05 |
| Gvin1 | 2890.03 | -0.75 | 0.18 | -4.06 | 4.89E-05 | 9.40E-04 |
| Medag | 730.19 | -0.75 | 0.29 | -2.60 | 9.20E-03 | 7.59E-02 |
| Ahsa2 | 1173.53 | -0.75 | 0.17 | -4.50 | 6.93E-06 | 1.64E-04 |
| Srp54b | 1068.57 | -0.75 | 0.17 | -4.48 | 7.45E-06 | 1.75E-04 |
| Fam49a | 354.28 | -0.75 | 0.24 | -3.16 | 1.56E-03 | 1.85E-02 |
| Eif4e | 3849.76 | -0.75 | 0.16 | -4.67 | 3.02E-06 | 7.70E-05 |
| Ggct | 519.68 | -0.75 | 0.24 | -3.12 | 1.83E-03 | 2.12E-02 |
| Cttnbp2nl | 1057.59 | -0.75 | 0.25 | -3.03 | 2.47E-03 | 2.68E-02 |
| Fgd6 | 323.59 | -0.75 | 0.26 | -2.89 | 3.87E-03 | 3.86E-02 |
| Bag3 | 8336.02 | -0.75 | 0.18 | -4.07 | 4.65E-05 | 8.97E-04 |
| Picalm | 6962.61 | -0.75 | 0.19 | -3.96 | 7.63E-05 | 1.39E-03 |
| Prkab2 | 1874.05 | -0.75 | 0.21 | -3.66 | 2.55E-04 | 4.02E-03 |
| Dot1l | 3366.43 | -0.75 | 0.19 | -3.90 | 9.80E-05 | 1.74E-03 |
| Elf4 | 567.03 | -0.75 | 0.27 | -2.81 | 4.97E-03 | 4.70E-02 |
| Nfyb | 469.09 | -0.75 | 0.23 | -3.28 | 1.04E-03 | 1.33E-02 |
| Sdcbp | 3342.64 | -0.76 | 0.17 | -4.33 | 1.48E-05 | 3.27E-04 |
| Fzd1 | 270.45 | -0.76 | 0.26 | -2.93 | 3.34E-03 | 3.43E-02 |
| Rbpj | 1204.02 | -0.76 | 0.15 | -4.94 | 7.90E-07 | 2.25E-05 |
| Ubc | 47549.33 | -0.76 | 0.14 | -5.50 | 3.72E-08 | 1.34E-06 |
| Tgoln1 | 3825.42 | -0.76 | 0.16 | -4.88 | 1.04E-06 | 2.88E-05 |
| Hexim1 | 1029.77 | -0.76 | 0.24 | -3.14 | 1.66E-03 | 1.96E-02 |
| Idi1 | 276.62 | -0.76 | 0.23 | -3.32 | 8.93E-04 | 1.18E-02 |
| Mast4 | 1331.73 | -0.76 | 0.21 | -3.67 | 2.45E-04 | 3.88E-03 |
| Col27a1 | 393.78 | -0.76 | 0.28 | -2.78 | 5.43E-03 | 5.06E-02 |
| Arhgap23 | 1293.89 | -0.77 | 0.23 | -3.30 | 9.77E-04 | 1.26E-02 |
| Actb | 14275.13 | -0.77 | 0.14 | -5.62 | 1.88E-08 | 7.26E-07 |
| Cd53 | 265.73 | -0.77 | 0.31 | -2.51 | 1.19E-02 | 9.21E-02 |
| Tnip2 | 576.89 | -0.77 | 0.25 | -3.12 | 1.81E-03 | 2.11E-02 |
| Tlnrd1 | 905.82 | -0.77 | 0.26 | -2.95 | 3.22E-03 | 3.32E-02 |
| Rai14 | 293.79 | -0.77 | 0.24 | -3.23 | 1.24E-03 | 1.54E-02 |
| Ptbp1 | 1699.41 | -0.77 | 0.20 | -3.95 | 7.91E-05 | 1.44E-03 |
| Sqstm1 | 14609.10 | -0.77 | 0.17 | -4.54 | 5.72E-06 | 1.39E-04 |
| Nrp2 | 2385.23 | -0.77 | 0.22 | -3.47 | 5.18E-04 | 7.39E-03 |
| Epb41l3 | 227.62 | -0.78 | 0.31 | -2.48 | 1.30E-02 | 9.83E-02 |
| Klhl15 | 125.12 | -0.78 | 0.31 | -2.48 | 1.31E-02 | 9.86E-02 |
| Maml1 | 684.40 | -0.78 | 0.17 | -4.46 | 8.33E-06 | 1.93E-04 |
| Sik2 | 480.18 | -0.78 | 0.20 | -3.87 | 1.10E-04 | 1.92E-03 |
| Bcor | 1286.40 | -0.78 | 0.18 | -4.30 | 1.72E-05 | 3.71E-04 |
| Traf3 | 791.92 | -0.78 | 0.22 | -3.53 | 4.18E-04 | 6.11E-03 |
| Dnaja4 | 4823.40 | -0.78 | 0.15 | -5.30 | 1.15E-07 | 3.77E-06 |
| Zkscan5 | 413.09 | -0.78 | 0.27 | -2.92 | 3.46E-03 | 3.53E-02 |
| Akap13 | 3261.02 | -0.78 | 0.14 | -5.74 | 9.32E-09 | 3.73E-07 |
| Ctps | 1636.20 | -0.79 | 0.20 | -3.87 | 1.07E-04 | 1.88E-03 |
| Mamstr | 236.35 | -0.79 | 0.30 | -2.63 | 8.48E-03 | 7.13E-02 |
| Tnfrsf1a | 1522.28 | -0.79 | 0.20 | -3.91 | 9.17E-05 | 1.64E-03 |
| Serpinh1 | 8902.41 | -0.79 | 0.22 | -3.55 | 3.91E-04 | 5.78E-03 |
| Stxbp6 | 158.27 | -0.79 | 0.29 | -2.69 | 7.17E-03 | 6.25E-02 |
| H2-D1 | 9420.07 | -0.79 | 0.22 | -3.63 | 2.87E-04 | 4.43E-03 |
| Mcam | 1782.70 | -0.79 | 0.19 | -4.22 | 2.47E-05 | 5.13E-04 |
| Nfya | 875.93 | -0.79 | 0.17 | -4.67 | 3.00E-06 | 7.67E-05 |
| Zfp335 | 751.39 | -0.79 | 0.18 | -4.50 | 6.74E-06 | 1.61E-04 |
| Chordc1 | 1427.93 | -0.79 | 0.19 | -4.07 | 4.63E-05 | 8.95E-04 |
| Samd4b | 1090.19 | -0.79 | 0.18 | -4.42 | 9.98E-06 | 2.27E-04 |
| Ppan | 317.85 | -0.79 | 0.29 | -2.70 | 6.90E-03 | 6.07E-02 |
| Eif4a-ps4 | 918.12 | -0.80 | 0.26 | -3.07 | 2.17E-03 | 2.41E-02 |
| Foxo1 | 1061.35 | -0.80 | 0.16 | -4.85 | 1.23E-06 | 3.37E-05 |
| Nfatc2 | 620.95 | -0.80 | 0.26 | -3.09 | 2.03E-03 | 2.29E-02 |
| Rnf2 | 568.98 | -0.80 | 0.29 | -2.75 | 6.05E-03 | 5.48E-02 |
| Tfe3 | 663.06 | -0.80 | 0.21 | -3.82 | 1.33E-04 | 2.28E-03 |
| Top1 | 2164.86 | -0.80 | 0.18 | -4.55 | 5.34E-06 | 1.30E-04 |
| Adgrg1 | 2548.58 | -0.80 | 0.20 | -4.10 | 4.17E-05 | 8.17E-04 |
| Foxc1 | 224.40 | -0.80 | 0.29 | -2.75 | 6.04E-03 | 5.48E-02 |
| Tes | 141.65 | -0.80 | 0.32 | -2.50 | 1.26E-02 | 9.55E-02 |
| Snx10 | 861.93 | -0.80 | 0.20 | -4.04 | 5.37E-05 | 1.03E-03 |
| Nup54 | 462.59 | -0.80 | 0.22 | -3.65 | 2.61E-04 | 4.10E-03 |
| Ccdc50 | 1163.79 | -0.80 | 0.19 | -4.28 | 1.85E-05 | 3.99E-04 |
| Scyl2 | 775.85 | -0.81 | 0.20 | -4.03 | 5.69E-05 | 1.08E-03 |
| Nfkbib | 745.27 | -0.81 | 0.19 | -4.15 | 3.29E-05 | 6.62E-04 |
| Hspa8 | 61563.06 | -0.81 | 0.17 | -4.67 | 3.00E-06 | 7.67E-05 |
| Rassf2 | 318.01 | -0.81 | 0.23 | -3.45 | 5.64E-04 | 7.95E-03 |
| Taf7 | 449.38 | -0.81 | 0.25 | -3.26 | 1.11E-03 | 1.41E-02 |
| Cyb561 | 564.01 | -0.81 | 0.27 | -3.02 | 2.53E-03 | 2.74E-02 |
| Crlf2 | 350.67 | -0.81 | 0.29 | -2.82 | 4.76E-03 | 4.55E-02 |
| Dse | 321.04 | -0.81 | 0.30 | -2.70 | 7.04E-03 | 6.15E-02 |
| Ets1 | 2701.09 | -0.81 | 0.23 | -3.47 | 5.21E-04 | 7.42E-03 |
| Col5a3 | 1696.95 | -0.81 | 0.30 | -2.72 | 6.47E-03 | 5.76E-02 |
| Jmjd1c | 2112.43 | -0.82 | 0.21 | -3.82 | 1.31E-04 | 2.26E-03 |
| Arf6 | 929.21 | -0.82 | 0.23 | -3.50 | 4.72E-04 | 6.81E-03 |
| Tjp2 | 956.15 | -0.82 | 0.19 | -4.20 | 2.68E-05 | 5.53E-04 |
| Fam129a | 847.15 | -0.82 | 0.19 | -4.35 | 1.36E-05 | 3.03E-04 |
| Cldnd1 | 1004.54 | -0.82 | 0.21 | -3.91 | 9.05E-05 | 1.62E-03 |
| Bhlhe41 | 890.32 | -0.82 | 0.20 | -4.17 | 3.05E-05 | 6.18E-04 |
| Smox | 794.56 | -0.82 | 0.20 | -4.09 | 4.27E-05 | 8.34E-04 |
| Slc20a1 | 1638.45 | -0.82 | 0.22 | -3.73 | 1.93E-04 | 3.18E-03 |
| Pdlim1 | 2446.12 | -0.82 | 0.20 | -4.13 | 3.66E-05 | 7.29E-04 |
| Anxa1 | 1156.02 | -0.82 | 0.31 | -2.63 | 8.66E-03 | 7.23E-02 |
| Map1b | 533.81 | -0.82 | 0.22 | -3.80 | 1.45E-04 | 2.47E-03 |
| Hcls1 | 453.66 | -0.83 | 0.22 | -3.71 | 2.05E-04 | 3.34E-03 |
| Fcgr2b | 620.26 | -0.83 | 0.31 | -2.64 | 8.32E-03 | 7.01E-02 |
| Nolc1 | 948.38 | -0.83 | 0.21 | -3.97 | 7.11E-05 | 1.31E-03 |
| Vash1 | 560.80 | -0.83 | 0.27 | -3.11 | 1.87E-03 | 2.15E-02 |
| Afmid | 177.66 | -0.83 | 0.28 | -2.94 | 3.30E-03 | 3.39E-02 |
| Cpeb4 | 2536.10 | -0.83 | 0.19 | -4.41 | 1.04E-05 | 2.36E-04 |
| Inpp5d | 327.58 | -0.83 | 0.33 | -2.50 | 1.25E-02 | 9.54E-02 |
| Src | 148.12 | -0.83 | 0.32 | -2.60 | 9.29E-03 | 7.64E-02 |
| Atp13a3 | 1675.09 | -0.83 | 0.15 | -5.48 | 4.14E-08 | 1.49E-06 |
| Hipk2 | 1386.92 | -0.83 | 0.28 | -2.99 | 2.75E-03 | 2.92E-02 |
| N4bp1 | 1331.76 | -0.84 | 0.16 | -5.20 | 1.96E-07 | 6.19E-06 |
| Adnp | 599.60 | -0.84 | 0.21 | -4.03 | 5.56E-05 | 1.06E-03 |
| Xaf1 | 580.23 | -0.84 | 0.27 | -3.16 | 1.59E-03 | 1.89E-02 |
| Mgat4a | 847.12 | -0.85 | 0.27 | -3.08 | 2.06E-03 | 2.31E-02 |
| Alpk1 | 270.70 | -0.85 | 0.29 | -2.92 | 3.50E-03 | 3.56E-02 |
| Atp7a | 195.30 | -0.85 | 0.26 | -3.26 | 1.12E-03 | 1.42E-02 |
| Erbin | 2248.49 | -0.85 | 0.18 | -4.75 | 2.08E-06 | 5.48E-05 |
| Stx6 | 660.71 | -0.85 | 0.19 | -4.47 | 7.75E-06 | 1.81E-04 |
| Slc7a7 | 120.20 | -0.85 | 0.33 | -2.55 | 1.09E-02 | 8.65E-02 |
| Edem1 | 627.74 | -0.85 | 0.20 | -4.35 | 1.39E-05 | 3.08E-04 |
| Zbtb10 | 719.78 | -0.85 | 0.23 | -3.73 | 1.95E-04 | 3.21E-03 |
| Rin3 | 789.22 | -0.85 | 0.23 | -3.70 | 2.15E-04 | 3.49E-03 |
| Rlim | 1157.42 | -0.85 | 0.24 | -3.54 | 3.94E-04 | 5.81E-03 |
| Zc3h12c | 354.26 | -0.85 | 0.28 | -3.09 | 2.01E-03 | 2.28E-02 |
| Tinagl1 | 2571.11 | -0.86 | 0.22 | -3.88 | 1.06E-04 | 1.85E-03 |
| Lbp | 332.53 | -0.86 | 0.23 | -3.72 | 2.01E-04 | 3.30E-03 |
| Diaph1 | 1184.04 | -0.86 | 0.23 | -3.76 | 1.68E-04 | 2.80E-03 |
| Trim30d | 169.68 | -0.86 | 0.31 | -2.79 | 5.31E-03 | 4.96E-02 |
| Med11 | 166.33 | -0.86 | 0.32 | -2.71 | 6.65E-03 | 5.88E-02 |
| Sec24a | 653.94 | -0.86 | 0.20 | -4.27 | 1.94E-05 | 4.16E-04 |
| Ttpal | 775.06 | -0.86 | 0.24 | -3.59 | 3.25E-04 | 4.94E-03 |
| Spry2 | 772.86 | -0.87 | 0.28 | -3.14 | 1.69E-03 | 1.99E-02 |
| Slc41a3 | 1819.41 | -0.87 | 0.23 | -3.70 | 2.18E-04 | 3.52E-03 |
| Nedd9 | 1340.84 | -0.87 | 0.29 | -3.00 | 2.71E-03 | 2.90E-02 |
| Simc1 | 156.70 | -0.87 | 0.34 | -2.55 | 1.09E-02 | 8.65E-02 |
| Agt | 158.22 | -0.87 | 0.28 | -3.11 | 1.89E-03 | 2.17E-02 |
| Klf7 | 1622.29 | -0.87 | 0.21 | -4.10 | 4.08E-05 | 8.04E-04 |
| Tmbim1 | 2295.25 | -0.87 | 0.26 | -3.39 | 6.93E-04 | 9.44E-03 |
| Meox1 | 668.35 | -0.87 | 0.32 | -2.76 | 5.79E-03 | 5.29E-02 |
| Efhd2 | 1004.91 | -0.87 | 0.25 | -3.49 | 4.83E-04 | 6.95E-03 |
| Vav1 | 140.50 | -0.88 | 0.35 | -2.48 | 1.30E-02 | 9.83E-02 |
| Slfn3 | 298.35 | -0.88 | 0.27 | -3.31 | 9.37E-04 | 1.22E-02 |
| Rhoj | 1423.43 | -0.88 | 0.22 | -4.01 | 6.00E-05 | 1.13E-03 |
| Ywhaz | 4071.83 | -0.88 | 0.20 | -4.49 | 7.12E-06 | 1.68E-04 |
| Tor3a | 612.24 | -0.88 | 0.24 | -3.60 | 3.14E-04 | 4.79E-03 |
| Phf11d | 602.66 | -0.88 | 0.24 | -3.60 | 3.14E-04 | 4.79E-03 |
| Rell1 | 607.47 | -0.88 | 0.21 | -4.22 | 2.47E-05 | 5.13E-04 |
| Klf4 | 4510.82 | -0.88 | 0.23 | -3.79 | 1.53E-04 | 2.60E-03 |
| Arih1 | 2854.80 | -0.88 | 0.16 | -5.57 | 2.57E-08 | 9.66E-07 |
| Cited2 | 2691.93 | -0.88 | 0.20 | -4.47 | 7.90E-06 | 1.84E-04 |
| Soga1 | 892.90 | -0.88 | 0.19 | -4.56 | 5.06E-06 | 1.24E-04 |
| Anxa7 | 2916.81 | -0.89 | 0.24 | -3.67 | 2.40E-04 | 3.82E-03 |
| Pogk | 912.43 | -0.89 | 0.19 | -4.56 | 5.02E-06 | 1.23E-04 |
| Oas2 | 764.62 | -0.89 | 0.22 | -4.08 | 4.48E-05 | 8.69E-04 |
| Loxl2 | 1264.22 | -0.89 | 0.21 | -4.24 | 2.23E-05 | 4.70E-04 |
| Marchf3 | 170.33 | -0.89 | 0.29 | -3.11 | 1.84E-03 | 2.13E-02 |
| Nkain1 | 181.18 | -0.89 | 0.32 | -2.76 | 5.74E-03 | 5.26E-02 |
| Slc39a6 | 275.01 | -0.89 | 0.27 | -3.36 | 7.70E-04 | 1.03E-02 |
| Shank1 | 124.33 | -0.89 | 0.36 | -2.50 | 1.25E-02 | 9.54E-02 |
| Rabgef1 | 879.28 | -0.89 | 0.20 | -4.44 | 9.01E-06 | 2.07E-04 |
| Col18a1 | 545.38 | -0.89 | 0.24 | -3.68 | 2.35E-04 | 3.75E-03 |
| Id2 | 297.12 | -0.89 | 0.27 | -3.26 | 1.12E-03 | 1.42E-02 |
| Eif5 | 5049.15 | -0.89 | 0.20 | -4.36 | 1.28E-05 | 2.85E-04 |
| Tle4 | 166.00 | -0.89 | 0.36 | -2.47 | 1.34E-02 | 9.98E-02 |
| Actg1 | 21388.21 | -0.90 | 0.20 | -4.46 | 8.34E-06 | 1.93E-04 |
| Lysmd3 | 533.35 | -0.90 | 0.29 | -3.12 | 1.84E-03 | 2.12E-02 |
| Klf10 | 911.71 | -0.90 | 0.31 | -2.90 | 3.69E-03 | 3.71E-02 |
| Fscn1 | 1480.02 | -0.90 | 0.22 | -4.00 | 6.21E-05 | 1.16E-03 |
| Bbc3 | 163.56 | -0.90 | 0.33 | -2.74 | 6.08E-03 | 5.50E-02 |
| Zfand5 | 471.43 | -0.90 | 0.36 | -2.49 | 1.27E-02 | 9.65E-02 |
| Gnl3 | 886.89 | -0.90 | 0.23 | -3.90 | 9.80E-05 | 1.74E-03 |
| Pdk4 | 23980.65 | -0.90 | 0.29 | -3.07 | 2.12E-03 | 2.37E-02 |
| Nfe2l2 | 2584.42 | -0.90 | 0.19 | -4.81 | 1.50E-06 | 4.05E-05 |
| Tob1 | 2101.51 | -0.90 | 0.21 | -4.32 | 1.53E-05 | 3.35E-04 |
| Camkk2 | 495.42 | -0.90 | 0.21 | -4.27 | 1.96E-05 | 4.20E-04 |
| Zfp217 | 482.34 | -0.91 | 0.23 | -3.91 | 9.37E-05 | 1.67E-03 |
| Foxs1 | 118.02 | -0.91 | 0.32 | -2.80 | 5.04E-03 | 4.75E-02 |
| Clic4 | 17017.39 | -0.91 | 0.14 | -6.46 | 1.03E-10 | 5.54E-09 |
| S1pr2 | 220.24 | -0.91 | 0.33 | -2.79 | 5.30E-03 | 4.95E-02 |
| Rybp | 642.92 | -0.91 | 0.22 | -4.19 | 2.80E-05 | 5.73E-04 |
| Rffl | 259.31 | -0.91 | 0.24 | -3.76 | 1.72E-04 | 2.87E-03 |
| Iqgap1 | 2736.40 | -0.92 | 0.19 | -4.90 | 9.57E-07 | 2.67E-05 |
| Plagl1 | 222.66 | -0.92 | 0.27 | -3.34 | 8.35E-04 | 1.11E-02 |
| Slc4a8 | 173.29 | -0.92 | 0.31 | -2.92 | 3.49E-03 | 3.55E-02 |
| Dusp8 | 2134.95 | -0.92 | 0.18 | -5.05 | 4.41E-07 | 1.31E-05 |
| Slc3a2 | 1476.77 | -0.92 | 0.24 | -3.90 | 9.57E-05 | 1.70E-03 |
| Osgin1 | 237.17 | -0.92 | 0.36 | -2.60 | 9.34E-03 | 7.66E-02 |
| Dusp27 | 1416.23 | -0.93 | 0.27 | -3.48 | 5.07E-04 | 7.25E-03 |
| Tmx4 | 1992.67 | -0.93 | 0.20 | -4.59 | 4.50E-06 | 1.11E-04 |
| Auts2 | 776.89 | -0.93 | 0.18 | -5.15 | 2.57E-07 | 8.04E-06 |
| Cdr2l | 261.71 | -0.93 | 0.32 | -2.89 | 3.90E-03 | 3.89E-02 |
| Btbd8 | 85.59 | -0.94 | 0.36 | -2.63 | 8.49E-03 | 7.14E-02 |
| Nos3 | 2170.12 | -0.94 | 0.28 | -3.33 | 8.73E-04 | 1.16E-02 |
| Spsb1 | 1099.24 | -0.94 | 0.19 | -4.85 | 1.23E-06 | 3.37E-05 |
| Dennd3 | 751.59 | -0.94 | 0.27 | -3.53 | 4.08E-04 | 5.99E-03 |
| Syt12 | 163.79 | -0.94 | 0.29 | -3.19 | 1.42E-03 | 1.72E-02 |
| Cers6 | 153.77 | -0.94 | 0.36 | -2.59 | 9.65E-03 | 7.84E-02 |
| Rap1b | 3096.46 | -0.94 | 0.21 | -4.56 | 5.12E-06 | 1.25E-04 |
| Slc7a8 | 126.63 | -0.94 | 0.35 | -2.68 | 7.42E-03 | 6.41E-02 |
| Fem1b | 1002.70 | -0.94 | 0.18 | -5.37 | 7.75E-08 | 2.63E-06 |
| Myh9 | 7977.30 | -0.94 | 0.17 | -5.48 | 4.28E-08 | 1.53E-06 |
| Clmp | 210.59 | -0.94 | 0.30 | -3.14 | 1.67E-03 | 1.97E-02 |
| Eef1e1 | 302.61 | -0.94 | 0.27 | -3.48 | 4.94E-04 | 7.09E-03 |
| Homer1 | 1618.53 | -0.95 | 0.20 | -4.63 | 3.63E-06 | 9.12E-05 |
| Il10ra | 229.28 | -0.95 | 0.28 | -3.33 | 8.71E-04 | 1.15E-02 |
| Sertad2 | 1042.39 | -0.95 | 0.16 | -5.88 | 4.16E-09 | 1.76E-07 |
| Ncf1 | 266.37 | -0.95 | 0.34 | -2.79 | 5.20E-03 | 4.88E-02 |
| Fam222a | 92.55 | -0.96 | 0.38 | -2.53 | 1.15E-02 | 8.99E-02 |
| Dbn1 | 450.69 | -0.96 | 0.21 | -4.53 | 5.98E-06 | 1.45E-04 |
| Fignl2 | 198.75 | -0.96 | 0.32 | -2.99 | 2.78E-03 | 2.95E-02 |
| Apaf1 | 590.50 | -0.96 | 0.19 | -4.94 | 7.96E-07 | 2.26E-05 |
| Col4a1 | 22465.25 | -0.96 | 0.17 | -5.60 | 2.09E-08 | 7.98E-07 |
| Cnnm4 | 305.39 | -0.96 | 0.25 | -3.81 | 1.38E-04 | 2.36E-03 |
| Ninl | 121.63 | -0.96 | 0.33 | -2.89 | 3.84E-03 | 3.84E-02 |
| Adcy1 | 104.42 | -0.96 | 0.39 | -2.47 | 1.33E-02 | 9.98E-02 |
| Cnn3 | 1813.95 | -0.96 | 0.18 | -5.37 | 7.73E-08 | 2.63E-06 |
| Man1a | 1501.90 | -0.97 | 0.20 | -4.76 | 1.98E-06 | 5.24E-05 |
| Hmga1 | 177.61 | -0.97 | 0.33 | -2.91 | 3.57E-03 | 3.62E-02 |
| Zbtb46 | 958.69 | -0.97 | 0.18 | -5.52 | 3.47E-08 | 1.26E-06 |
| Tubb4b | 7860.63 | -0.97 | 0.17 | -5.82 | 5.96E-09 | 2.46E-07 |
| Ddx21 | 2071.00 | -0.97 | 0.18 | -5.30 | 1.15E-07 | 3.77E-06 |
| Katna1 | 582.09 | -0.97 | 0.24 | -4.05 | 5.13E-05 | 9.80E-04 |
| Wfdc17 | 205.38 | -0.97 | 0.35 | -2.76 | 5.70E-03 | 5.24E-02 |
| Aldh1a2 | 250.56 | -0.97 | 0.32 | -3.02 | 2.55E-03 | 2.76E-02 |
| Tbc1d15 | 948.40 | -0.97 | 0.19 | -5.11 | 3.29E-07 | 1.00E-05 |
| Rps6ka3 | 1920.00 | -0.98 | 0.18 | -5.33 | 9.69E-08 | 3.22E-06 |
| Etv6 | 600.33 | -0.98 | 0.25 | -3.97 | 7.23E-05 | 1.32E-03 |
| Lrrc8d | 360.88 | -0.98 | 0.22 | -4.44 | 9.02E-06 | 2.07E-04 |
| Casp3 | 137.98 | -0.98 | 0.39 | -2.54 | 1.12E-02 | 8.76E-02 |
| Zc3hav1l | 88.69 | -0.98 | 0.38 | -2.60 | 9.21E-03 | 7.59E-02 |
| Pak4 | 293.90 | -0.98 | 0.25 | -3.86 | 1.14E-04 | 1.98E-03 |
| D1Ertd622e | 185.64 | -0.99 | 0.34 | -2.92 | 3.51E-03 | 3.56E-02 |
| Rgs2 | 1156.06 | -0.99 | 0.25 | -4.02 | 5.82E-05 | 1.10E-03 |
| Creb1 | 961.00 | -0.99 | 0.22 | -4.44 | 8.89E-06 | 2.05E-04 |
| Ddx58 | 987.62 | -0.99 | 0.22 | -4.42 | 9.87E-06 | 2.25E-04 |
| Metrnl | 579.49 | -0.99 | 0.30 | -3.29 | 9.87E-04 | 1.27E-02 |
| Mt1 | 6597.47 | -0.99 | 0.20 | -5.09 | 3.59E-07 | 1.08E-05 |
| Pnrc1 | 4310.18 | -1.00 | 0.16 | -6.19 | 6.10E-10 | 2.89E-08 |
| Gbp3 | 1305.65 | -1.00 | 0.22 | -4.50 | 6.92E-06 | 1.64E-04 |
| Rhou | 278.13 | -1.00 | 0.26 | -3.91 | 9.10E-05 | 1.63E-03 |
| Lrrc8c | 1202.01 | -1.00 | 0.18 | -5.44 | 5.22E-08 | 1.84E-06 |
| Zfp800 | 313.35 | -1.00 | 0.24 | -4.17 | 2.99E-05 | 6.07E-04 |
| Acsl4 | 617.27 | -1.00 | 0.24 | -4.10 | 4.17E-05 | 8.17E-04 |
| 1810055G02Rik | 259.91 | -1.00 | 0.26 | -3.89 | 9.97E-05 | 1.76E-03 |
| Lrrc8b | 317.17 | -1.00 | 0.30 | -3.38 | 7.18E-04 | 9.70E-03 |
| Nfatc1 | 605.73 | -1.01 | 0.20 | -5.04 | 4.57E-07 | 1.36E-05 |
| Gabpb1 | 519.57 | -1.01 | 0.22 | -4.66 | 3.10E-06 | 7.88E-05 |
| Nle1 | 356.00 | -1.01 | 0.30 | -3.42 | 6.29E-04 | 8.68E-03 |
| Timd4 | 84.04 | -1.01 | 0.39 | -2.58 | 9.98E-03 | 8.03E-02 |
| Fbn1 | 3920.39 | -1.02 | 0.18 | -5.52 | 3.35E-08 | 1.22E-06 |
| Cdr2 | 508.34 | -1.02 | 0.23 | -4.34 | 1.42E-05 | 3.15E-04 |
| Plekha4 | 200.95 | -1.02 | 0.36 | -2.83 | 4.59E-03 | 4.43E-02 |
| Dtx3l | 1005.01 | -1.02 | 0.24 | -4.23 | 2.30E-05 | 4.81E-04 |
| Ptpn23 | 1235.65 | -1.02 | 0.22 | -4.74 | 2.13E-06 | 5.61E-05 |
| Nlgn2 | 525.75 | -1.02 | 0.20 | -5.23 | 1.72E-07 | 5.51E-06 |
| Lcp1 | 1225.40 | -1.02 | 0.22 | -4.56 | 5.13E-06 | 1.25E-04 |
| Rnf213 | 4453.84 | -1.02 | 0.20 | -5.15 | 2.66E-07 | 8.28E-06 |
| Ero1l | 770.18 | -1.02 | 0.18 | -5.60 | 2.14E-08 | 8.14E-07 |
| Nppb | 9223.86 | -1.03 | 0.30 | -3.41 | 6.42E-04 | 8.83E-03 |
| Ms4a6d | 172.77 | -1.03 | 0.32 | -3.25 | 1.14E-03 | 1.44E-02 |
| Trim30a | 839.03 | -1.03 | 0.26 | -3.99 | 6.49E-05 | 1.21E-03 |
| Ehd1 | 4605.81 | -1.03 | 0.19 | -5.52 | 3.39E-08 | 1.23E-06 |
| Zmynd15 | 212.63 | -1.03 | 0.31 | -3.32 | 8.93E-04 | 1.18E-02 |
| Pdlim7 | 1813.57 | -1.03 | 0.21 | -4.82 | 1.43E-06 | 3.87E-05 |
| Thsd7a | 285.94 | -1.03 | 0.34 | -3.05 | 2.28E-03 | 2.52E-02 |
| Igsf6 | 123.74 | -1.03 | 0.37 | -2.81 | 5.02E-03 | 4.74E-02 |
| Pdlim3 | 492.53 | -1.04 | 0.31 | -3.33 | 8.66E-04 | 1.15E-02 |
| Cdk6 | 528.53 | -1.04 | 0.22 | -4.78 | 1.78E-06 | 4.78E-05 |
| Eif6 | 1262.01 | -1.04 | 0.31 | -3.34 | 8.28E-04 | 1.10E-02 |
| Foxc2 | 95.17 | -1.04 | 0.38 | -2.77 | 5.61E-03 | 5.19E-02 |
| Prkd2 | 1582.82 | -1.04 | 0.28 | -3.77 | 1.63E-04 | 2.74E-03 |
| Stk40 | 2666.96 | -1.04 | 0.19 | -5.54 | 3.03E-08 | 1.12E-06 |
| Ubb-ps | 1384.93 | -1.04 | 0.27 | -3.81 | 1.37E-04 | 2.34E-03 |
| Ncoa7 | 313.50 | -1.04 | 0.38 | -2.72 | 6.56E-03 | 5.81E-02 |
| Rc3h1 | 1463.43 | -1.04 | 0.18 | -5.92 | 3.15E-09 | 1.36E-07 |
| Usp37 | 382.87 | -1.04 | 0.27 | -3.89 | 9.88E-05 | 1.75E-03 |
| Nol10 | 420.45 | -1.04 | 0.29 | -3.59 | 3.33E-04 | 5.04E-03 |
| Ptpn1 | 967.56 | -1.04 | 0.25 | -4.24 | 2.26E-05 | 4.74E-04 |
| Cnksr1 | 394.12 | -1.04 | 0.28 | -3.67 | 2.40E-04 | 3.82E-03 |
| Slc6a17 | 157.17 | -1.05 | 0.32 | -3.23 | 1.24E-03 | 1.54E-02 |
| Gm4070 | 2174.60 | -1.05 | 0.24 | -4.45 | 8.73E-06 | 2.02E-04 |
| Tgtp2 | 1371.61 | -1.05 | 0.30 | -3.44 | 5.72E-04 | 8.05E-03 |
| Sirt1 | 849.62 | -1.05 | 0.20 | -5.37 | 7.73E-08 | 2.63E-06 |
| Myo1g | 186.37 | -1.05 | 0.29 | -3.63 | 2.84E-04 | 4.40E-03 |
| Rab20 | 813.50 | -1.05 | 0.22 | -4.76 | 1.93E-06 | 5.14E-05 |
| Gbp6 | 3267.94 | -1.05 | 0.18 | -5.76 | 8.20E-09 | 3.33E-07 |
| Slc66a2 | 825.90 | -1.05 | 0.26 | -4.09 | 4.28E-05 | 8.34E-04 |
| Abl2 | 1183.00 | -1.05 | 0.19 | -5.57 | 2.52E-08 | 9.48E-07 |
| Nhsl2 | 302.36 | -1.05 | 0.29 | -3.70 | 2.19E-04 | 3.54E-03 |
| Ikbke | 131.46 | -1.06 | 0.32 | -3.27 | 1.06E-03 | 1.35E-02 |
| Flvcr1 | 322.09 | -1.06 | 0.22 | -4.73 | 2.24E-06 | 5.86E-05 |
| Nup98 | 1561.85 | -1.06 | 0.17 | -6.25 | 4.23E-10 | 2.07E-08 |
| Chd1 | 1059.73 | -1.06 | 0.19 | -5.53 | 3.13E-08 | 1.15E-06 |
| Tle3 | 778.73 | -1.06 | 0.17 | -6.10 | 1.07E-09 | 4.95E-08 |
| Rap2b | 299.20 | -1.07 | 0.22 | -4.76 | 1.90E-06 | 5.07E-05 |
| Ipmk | 790.04 | -1.08 | 0.24 | -4.46 | 8.05E-06 | 1.87E-04 |
| Zfp516 | 553.76 | -1.08 | 0.21 | -5.20 | 2.01E-07 | 6.32E-06 |
| Ednrb | 1255.62 | -1.08 | 0.27 | -3.97 | 7.16E-05 | 1.31E-03 |
| Btg2 | 12165.83 | -1.08 | 0.19 | -5.75 | 9.00E-09 | 3.61E-07 |
| Ugcg | 798.87 | -1.09 | 0.27 | -4.09 | 4.28E-05 | 8.34E-04 |
| S100a10 | 1377.10 | -1.09 | 0.25 | -4.36 | 1.31E-05 | 2.91E-04 |
| Spred1 | 1238.95 | -1.09 | 0.20 | -5.52 | 3.30E-08 | 1.21E-06 |
| Ikzf1 | 110.92 | -1.09 | 0.37 | -2.98 | 2.85E-03 | 3.00E-02 |
| Swap70 | 1033.32 | -1.10 | 0.27 | -4.02 | 5.90E-05 | 1.11E-03 |
| Ctla2a | 834.09 | -1.10 | 0.36 | -3.08 | 2.08E-03 | 2.33E-02 |
| Sat1 | 2436.35 | -1.10 | 0.28 | -3.97 | 7.34E-05 | 1.34E-03 |
| Sh2b3 | 1775.95 | -1.10 | 0.25 | -4.48 | 7.52E-06 | 1.76E-04 |
| Btg1 | 1973.34 | -1.10 | 0.25 | -4.38 | 1.17E-05 | 2.62E-04 |
| Gpr4 | 580.27 | -1.10 | 0.27 | -4.14 | 3.46E-05 | 6.93E-04 |
| Rasl11b | 1520.23 | -1.10 | 0.26 | -4.20 | 2.71E-05 | 5.57E-04 |
| Zbtb11 | 616.83 | -1.10 | 0.19 | -5.71 | 1.11E-08 | 4.40E-07 |
| Nupr1 | 285.36 | -1.10 | 0.35 | -3.20 | 1.40E-03 | 1.69E-02 |
| 1110002E22Rik | 3014.36 | -1.11 | 0.18 | -6.19 | 6.08E-10 | 2.89E-08 |
| Malt1 | 97.63 | -1.11 | 0.35 | -3.14 | 1.70E-03 | 2.00E-02 |
| Myo1e | 643.08 | -1.11 | 0.21 | -5.34 | 9.32E-08 | 3.11E-06 |
| Sox7 | 1242.93 | -1.11 | 0.25 | -4.51 | 6.63E-06 | 1.58E-04 |
| Yars | 187.67 | -1.11 | 0.27 | -4.07 | 4.66E-05 | 8.98E-04 |
| Atp10a | 131.17 | -1.11 | 0.33 | -3.35 | 8.07E-04 | 1.08E-02 |
| Cdc42ep4 | 906.31 | -1.12 | 0.31 | -3.55 | 3.83E-04 | 5.69E-03 |
| Herc6 | 570.64 | -1.12 | 0.21 | -5.45 | 5.01E-08 | 1.78E-06 |
| Ankrd23 | 21220.36 | -1.12 | 0.15 | -7.29 | 2.99E-13 | 2.22E-11 |
| Kctd12 | 2064.82 | -1.12 | 0.23 | -4.86 | 1.15E-06 | 3.16E-05 |
| Ptpn12 | 1521.70 | -1.12 | 0.22 | -5.15 | 2.61E-07 | 8.15E-06 |
| Irf2bpl | 2008.07 | -1.13 | 0.19 | -6.08 | 1.19E-09 | 5.42E-08 |
| Slc7a1 | 2028.27 | -1.13 | 0.19 | -5.86 | 4.62E-09 | 1.93E-07 |
| Prkcd | 725.37 | -1.13 | 0.19 | -5.78 | 7.44E-09 | 3.04E-07 |
| Rrad | 16970.63 | -1.13 | 0.27 | -4.21 | 2.51E-05 | 5.21E-04 |
| Jun | 15726.23 | -1.13 | 0.18 | -6.11 | 9.78E-10 | 4.57E-08 |
| Gcc1 | 761.69 | -1.13 | 0.31 | -3.62 | 2.97E-04 | 4.57E-03 |
| Slc2a1 | 678.98 | -1.13 | 0.25 | -4.49 | 7.20E-06 | 1.69E-04 |
| Dnajb4 | 4879.42 | -1.13 | 0.18 | -6.44 | 1.18E-10 | 6.27E-09 |
| Vcan | 818.00 | -1.13 | 0.18 | -6.25 | 4.19E-10 | 2.06E-08 |
| Rhob | 9941.54 | -1.13 | 0.19 | -5.97 | 2.32E-09 | 1.02E-07 |
| Ints6 | 654.78 | -1.13 | 0.24 | -4.72 | 2.37E-06 | 6.16E-05 |
| Flnb | 4214.96 | -1.13 | 0.27 | -4.20 | 2.70E-05 | 5.55E-04 |
| Ptp4a1 | 239.94 | -1.13 | 0.38 | -2.99 | 2.75E-03 | 2.92E-02 |
| Msx1 | 224.49 | -1.13 | 0.37 | -3.07 | 2.15E-03 | 2.39E-02 |
| Spon2 | 219.64 | -1.14 | 0.44 | -2.57 | 1.01E-02 | 8.10E-02 |
| Pprc1 | 813.96 | -1.14 | 0.21 | -5.44 | 5.42E-08 | 1.90E-06 |
| Pou2f2 | 146.89 | -1.14 | 0.32 | -3.58 | 3.44E-04 | 5.19E-03 |
| Arl5c | 76.83 | -1.14 | 0.39 | -2.95 | 3.14E-03 | 3.26E-02 |
| Pard6b | 77.23 | -1.14 | 0.40 | -2.84 | 4.58E-03 | 4.43E-02 |
| Plscr1 | 841.16 | -1.15 | 0.22 | -5.12 | 3.08E-07 | 9.43E-06 |
| Tnfrsf23 | 122.41 | -1.15 | 0.37 | -3.13 | 1.76E-03 | 2.05E-02 |
| Trim25 | 1801.99 | -1.15 | 0.22 | -5.17 | 2.34E-07 | 7.33E-06 |
| Zfp568 | 875.32 | -1.15 | 0.23 | -5.04 | 4.65E-07 | 1.38E-05 |
| Uck2 | 1718.21 | -1.15 | 0.24 | -4.81 | 1.54E-06 | 4.16E-05 |
| Prr5l | 82.72 | -1.16 | 0.38 | -3.07 | 2.17E-03 | 2.42E-02 |
| Syne3 | 292.85 | -1.17 | 0.24 | -4.85 | 1.26E-06 | 3.44E-05 |
| Ifitm3 | 3027.76 | -1.17 | 0.21 | -5.55 | 2.92E-08 | 1.09E-06 |
| Ccn1 | 12655.66 | -1.17 | 0.21 | -5.66 | 1.54E-08 | 5.98E-07 |
| Fbxo33 | 343.04 | -1.17 | 0.25 | -4.69 | 2.72E-06 | 6.99E-05 |
| Lcorl | 176.13 | -1.17 | 0.36 | -3.23 | 1.24E-03 | 1.53E-02 |
| Fhl3 | 289.91 | -1.18 | 0.28 | -4.27 | 1.98E-05 | 4.22E-04 |
| Zfp503 | 108.86 | -1.18 | 0.41 | -2.90 | 3.74E-03 | 3.76E-02 |
| Lcn2 | 161.05 | -1.18 | 0.32 | -3.64 | 2.68E-04 | 4.19E-03 |
| Elf1 | 1028.21 | -1.18 | 0.22 | -5.37 | 7.84E-08 | 2.65E-06 |
| Ralb | 1126.87 | -1.18 | 0.19 | -6.11 | 9.82E-10 | 4.58E-08 |
| Mtcl1 | 201.33 | -1.18 | 0.27 | -4.32 | 1.58E-05 | 3.45E-04 |
| Trpv4 | 372.41 | -1.18 | 0.22 | -5.41 | 6.22E-08 | 2.16E-06 |
| Ldlr | 449.61 | -1.19 | 0.29 | -4.12 | 3.81E-05 | 7.55E-04 |
| Spty2d1 | 799.82 | -1.19 | 0.23 | -5.23 | 1.70E-07 | 5.44E-06 |
| Ptprc | 370.80 | -1.19 | 0.24 | -4.97 | 6.55E-07 | 1.89E-05 |
| Ywhaq | 242.01 | -1.20 | 0.38 | -3.13 | 1.73E-03 | 2.03E-02 |
| Ttc9 | 82.02 | -1.20 | 0.44 | -2.71 | 6.77E-03 | 5.97E-02 |
| Cry1 | 361.34 | -1.20 | 0.26 | -4.64 | 3.40E-06 | 8.59E-05 |
| Pgm2 | 424.57 | -1.21 | 0.38 | -3.17 | 1.50E-03 | 1.80E-02 |
| Ssh1 | 662.91 | -1.21 | 0.26 | -4.64 | 3.55E-06 | 8.92E-05 |
| Slc4a7 | 280.74 | -1.21 | 0.33 | -3.64 | 2.76E-04 | 4.29E-03 |
| Klhl25 | 271.88 | -1.21 | 0.24 | -5.05 | 4.40E-07 | 1.31E-05 |
| Foxp4 | 753.50 | -1.21 | 0.23 | -5.35 | 8.65E-08 | 2.92E-06 |
| Rhog | 598.96 | -1.21 | 0.23 | -5.20 | 1.95E-07 | 6.18E-06 |
| Cflar | 2724.86 | -1.21 | 0.18 | -6.58 | 4.67E-11 | 2.65E-09 |
| Irf5 | 210.38 | -1.21 | 0.28 | -4.37 | 1.24E-05 | 2.78E-04 |
| Irf9 | 1179.93 | -1.21 | 0.23 | -5.22 | 1.77E-07 | 5.65E-06 |
| Fbxo30 | 1236.67 | -1.22 | 0.19 | -6.27 | 3.72E-10 | 1.85E-08 |
| Bcl10 | 983.75 | -1.22 | 0.24 | -5.04 | 4.70E-07 | 1.39E-05 |
| Ptpn2 | 630.26 | -1.22 | 0.23 | -5.35 | 8.68E-08 | 2.92E-06 |
| Nek6 | 194.71 | -1.23 | 0.36 | -3.44 | 5.81E-04 | 8.14E-03 |
| Rnf19b | 1465.23 | -1.23 | 0.21 | -5.74 | 9.48E-09 | 3.77E-07 |
| Oas1b | 215.58 | -1.23 | 0.32 | -3.82 | 1.32E-04 | 2.28E-03 |
| Enah | 5333.10 | -1.23 | 0.20 | -6.31 | 2.84E-10 | 1.43E-08 |
| Plk2 | 2461.06 | -1.23 | 0.18 | -6.98 | 2.89E-12 | 1.90E-10 |
| Plec | 14434.31 | -1.24 | 0.16 | -7.58 | 3.57E-14 | 2.94E-12 |
| Stat2 | 1452.31 | -1.24 | 0.20 | -6.35 | 2.10E-10 | 1.07E-08 |
| Rassf1 | 1362.56 | -1.24 | 0.31 | -3.98 | 6.76E-05 | 1.25E-03 |
| Hivep1 | 1454.44 | -1.24 | 0.16 | -7.79 | 6.55E-15 | 6.02E-13 |
| Noct | 12592.45 | -1.25 | 0.18 | -6.74 | 1.56E-11 | 9.31E-10 |
| Gbp2 | 2630.16 | -1.25 | 0.23 | -5.32 | 1.04E-07 | 3.44E-06 |
| Gramd1a | 2419.33 | -1.25 | 0.16 | -7.98 | 1.47E-15 | 1.45E-13 |
| Pwwp3b | 82.99 | -1.25 | 0.39 | -3.17 | 1.53E-03 | 1.82E-02 |
| Larp6 | 62.58 | -1.25 | 0.45 | -2.77 | 5.57E-03 | 5.16E-02 |
| Bcar1 | 1285.20 | -1.26 | 0.23 | -5.48 | 4.31E-08 | 1.54E-06 |
| Piezo1 | 2207.38 | -1.26 | 0.19 | -6.49 | 8.77E-11 | 4.79E-09 |
| Lpin2 | 435.54 | -1.26 | 0.23 | -5.39 | 7.04E-08 | 2.42E-06 |
| Ankrd33b | 1796.84 | -1.26 | 0.21 | -5.99 | 2.13E-09 | 9.45E-08 |
| Etv3 | 1787.72 | -1.27 | 0.15 | -8.47 | 2.35E-17 | 2.71E-15 |
| Bcl2l11 | 782.86 | -1.27 | 0.19 | -6.80 | 1.07E-11 | 6.54E-10 |
| Irf2bp2 | 2344.28 | -1.28 | 0.27 | -4.71 | 2.49E-06 | 6.45E-05 |
| Frmd6 | 1729.10 | -1.28 | 0.19 | -6.72 | 1.87E-11 | 1.11E-09 |
| Slfn9 | 351.26 | -1.28 | 0.35 | -3.65 | 2.62E-04 | 4.11E-03 |
| Map3k14 | 441.10 | -1.28 | 0.30 | -4.20 | 2.64E-05 | 5.45E-04 |
| Cars | 737.11 | -1.28 | 0.27 | -4.72 | 2.32E-06 | 6.05E-05 |
| Lilrb4a | 1218.34 | -1.28 | 0.32 | -3.96 | 7.47E-05 | 1.36E-03 |
| Ccnl1 | 2794.60 | -1.29 | 0.26 | -4.91 | 9.08E-07 | 2.55E-05 |
| 2010300C02Rik | 56.43 | -1.29 | 0.45 | -2.84 | 4.54E-03 | 4.40E-02 |
| Hspb1 | 17295.71 | -1.29 | 0.17 | -7.53 | 4.97E-14 | 4.07E-12 |
| Lox | 167.70 | -1.29 | 0.37 | -3.45 | 5.67E-04 | 7.97E-03 |
| Edn1 | 378.72 | -1.29 | 0.29 | -4.49 | 7.26E-06 | 1.71E-04 |
| Cbx4 | 499.60 | -1.29 | 0.25 | -5.14 | 2.79E-07 | 8.67E-06 |
| Jund | 9474.51 | -1.29 | 0.25 | -5.13 | 2.89E-07 | 8.92E-06 |
| Mmp3 | 211.26 | -1.29 | 0.35 | -3.70 | 2.19E-04 | 3.54E-03 |
| Slc25a36 | 2165.29 | -1.30 | 0.21 | -6.15 | 7.80E-10 | 3.67E-08 |
| Pdgfa | 1039.49 | -1.30 | 0.22 | -5.90 | 3.66E-09 | 1.56E-07 |
| Gnai3 | 1475.84 | -1.30 | 0.20 | -6.59 | 4.26E-11 | 2.44E-09 |
| Zfp703 | 792.69 | -1.30 | 0.25 | -5.29 | 1.23E-07 | 3.99E-06 |
| Baiap2 | 451.89 | -1.31 | 0.30 | -4.33 | 1.51E-05 | 3.31E-04 |
| Grrp1 | 339.46 | -1.31 | 0.42 | -3.10 | 1.96E-03 | 2.23E-02 |
| Gna13 | 1865.88 | -1.31 | 0.18 | -7.23 | 4.78E-13 | 3.44E-11 |
| Bach1 | 2199.25 | -1.32 | 0.18 | -7.25 | 4.07E-13 | 2.95E-11 |
| Plat | 1320.84 | -1.32 | 0.24 | -5.42 | 5.86E-08 | 2.04E-06 |
| Lacc1 | 324.02 | -1.32 | 0.26 | -5.14 | 2.81E-07 | 8.70E-06 |
| Vasn | 518.07 | -1.32 | 0.34 | -3.87 | 1.10E-04 | 1.92E-03 |
| Ubtd2 | 65.45 | -1.32 | 0.43 | -3.08 | 2.06E-03 | 2.31E-02 |
| Tpm3 | 3608.31 | -1.33 | 0.29 | -4.51 | 6.46E-06 | 1.54E-04 |
| H3f3b | 7202.30 | -1.33 | 0.27 | -4.94 | 7.89E-07 | 2.25E-05 |
| Klf6 | 5442.00 | -1.33 | 0.28 | -4.76 | 1.92E-06 | 5.12E-05 |
| Creb3l1 | 241.92 | -1.33 | 0.28 | -4.78 | 1.77E-06 | 4.74E-05 |
| Ier5 | 4573.36 | -1.33 | 0.20 | -6.77 | 1.33E-11 | 8.01E-10 |
| Sh3bp2 | 198.93 | -1.34 | 0.39 | -3.42 | 6.26E-04 | 8.64E-03 |
| Wsb1 | 1923.28 | -1.34 | 0.29 | -4.66 | 3.24E-06 | 8.20E-05 |
| Dhx58 | 193.18 | -1.34 | 0.35 | -3.83 | 1.28E-04 | 2.22E-03 |
| H2-Q7 | 2704.83 | -1.34 | 0.19 | -7.04 | 1.97E-12 | 1.32E-10 |
| Irf7 | 978.34 | -1.34 | 0.27 | -4.90 | 9.48E-07 | 2.65E-05 |
| Zfp954 | 205.46 | -1.34 | 0.33 | -4.10 | 4.05E-05 | 7.99E-04 |
| Siah2 | 377.74 | -1.35 | 0.27 | -5.01 | 5.35E-07 | 1.57E-05 |
| Ramp3 | 244.90 | -1.35 | 0.31 | -4.34 | 1.43E-05 | 3.17E-04 |
| Epop | 41.88 | -1.35 | 0.54 | -2.51 | 1.20E-02 | 9.23E-02 |
| Fut2 | 51.06 | -1.35 | 0.50 | -2.70 | 6.89E-03 | 6.06E-02 |
| Oasl2 | 1000.96 | -1.35 | 0.29 | -4.61 | 4.02E-06 | 9.99E-05 |
| Oas1a | 300.15 | -1.36 | 0.27 | -4.94 | 7.79E-07 | 2.23E-05 |
| Dyrk2 | 1628.90 | -1.36 | 0.17 | -8.13 | 4.41E-16 | 4.61E-14 |
| Zfand2a | 812.78 | -1.36 | 0.30 | -4.47 | 7.69E-06 | 1.80E-04 |
| Elmsan1 | 1436.78 | -1.36 | 0.20 | -6.86 | 6.75E-12 | 4.21E-10 |
| Sh3bgrl2 | 250.69 | -1.37 | 0.35 | -3.92 | 8.82E-05 | 1.59E-03 |
| Map2k3 | 2775.31 | -1.37 | 0.17 | -8.03 | 9.57E-16 | 9.71E-14 |
| Basp1 | 123.33 | -1.38 | 0.38 | -3.67 | 2.45E-04 | 3.88E-03 |
| Sp140 | 180.19 | -1.38 | 0.31 | -4.44 | 9.07E-06 | 2.08E-04 |
| Ptges | 74.76 | -1.38 | 0.45 | -3.06 | 2.21E-03 | 2.46E-02 |
| Tbc1d9 | 277.82 | -1.38 | 0.27 | -5.13 | 2.95E-07 | 9.07E-06 |
| Pvr | 1736.32 | -1.38 | 0.25 | -5.63 | 1.79E-08 | 6.92E-07 |
| Mmp14 | 814.11 | -1.39 | 0.22 | -6.45 | 1.12E-10 | 6.02E-09 |
| Irgm2 | 1514.95 | -1.39 | 0.26 | -5.27 | 1.40E-07 | 4.51E-06 |
| Fam129b | 1633.09 | -1.39 | 0.28 | -5.04 | 4.65E-07 | 1.38E-05 |
| Dusp4 | 124.06 | -1.39 | 0.42 | -3.31 | 9.20E-04 | 1.20E-02 |
| Saa3 | 434.75 | -1.40 | 0.27 | -5.22 | 1.80E-07 | 5.72E-06 |
| Rtn4rl2 | 159.47 | -1.40 | 0.35 | -3.98 | 6.82E-05 | 1.26E-03 |
| Atp2b4 | 1366.34 | -1.40 | 0.26 | -5.43 | 5.54E-08 | 1.94E-06 |
| Ugdh | 955.76 | -1.40 | 0.34 | -4.11 | 4.03E-05 | 7.96E-04 |
| Taf4b | 239.31 | -1.40 | 0.25 | -5.62 | 1.90E-08 | 7.30E-07 |
| Msr1 | 257.27 | -1.40 | 0.34 | -4.16 | 3.12E-05 | 6.32E-04 |
| H2-K1 | 2317.54 | -1.40 | 0.20 | -6.98 | 2.95E-12 | 1.93E-10 |
| Map3k6 | 1305.06 | -1.40 | 0.23 | -6.02 | 1.76E-09 | 7.87E-08 |
| Phf11b | 62.84 | -1.41 | 0.49 | -2.85 | 4.33E-03 | 4.24E-02 |
| Sesn2 | 241.94 | -1.42 | 0.34 | -4.17 | 3.01E-05 | 6.11E-04 |
| Usp18 | 354.00 | -1.42 | 0.32 | -4.50 | 6.92E-06 | 1.64E-04 |
| Dab2 | 2077.75 | -1.43 | 0.26 | -5.56 | 2.66E-08 | 9.96E-07 |
| Itgb3 | 129.27 | -1.43 | 0.37 | -3.89 | 9.93E-05 | 1.75E-03 |
| Tsc22d2 | 1421.69 | -1.43 | 0.21 | -6.88 | 5.86E-12 | 3.73E-10 |
| Odc1 | 3923.45 | -1.43 | 0.23 | -6.29 | 3.08E-10 | 1.55E-08 |
| Pxdc1 | 989.80 | -1.44 | 0.34 | -4.19 | 2.78E-05 | 5.70E-04 |
| Skil | 1573.70 | -1.44 | 0.18 | -7.98 | 1.52E-15 | 1.49E-13 |
| Fyb | 222.96 | -1.44 | 0.47 | -3.09 | 1.99E-03 | 2.26E-02 |
| Gas7 | 689.26 | -1.45 | 0.22 | -6.49 | 8.68E-11 | 4.76E-09 |
| Zfp57 | 102.25 | -1.45 | 0.45 | -3.21 | 1.33E-03 | 1.62E-02 |
| Neto2 | 48.13 | -1.45 | 0.48 | -3.04 | 2.39E-03 | 2.62E-02 |
| Cmpk2 | 1095.08 | -1.45 | 0.25 | -5.86 | 4.74E-09 | 1.98E-07 |
| Lmna | 7692.54 | -1.45 | 0.30 | -4.84 | 1.27E-06 | 3.46E-05 |
| Hspa2 | 351.21 | -1.46 | 0.42 | -3.46 | 5.37E-04 | 7.61E-03 |
| Zfp36l1 | 5757.86 | -1.46 | 0.23 | -6.37 | 1.84E-10 | 9.50E-09 |
| Ccn2 | 6253.49 | -1.46 | 0.17 | -8.48 | 2.25E-17 | 2.62E-15 |
| Gadd45a | 1097.78 | -1.47 | 0.35 | -4.23 | 2.34E-05 | 4.88E-04 |
| Dpy19l3 | 73.79 | -1.47 | 0.45 | -3.28 | 1.04E-03 | 1.33E-02 |
| Rdh10 | 403.95 | -1.48 | 0.31 | -4.77 | 1.87E-06 | 5.00E-05 |
| H2-Q4 | 4183.97 | -1.48 | 0.23 | -6.57 | 5.18E-11 | 2.91E-09 |
| Rasip1 | 3460.42 | -1.49 | 0.26 | -5.77 | 7.81E-09 | 3.18E-07 |
| Ubald1 | 1433.58 | -1.49 | 0.22 | -6.67 | 2.50E-11 | 1.45E-09 |
| Bhlhe40 | 5543.16 | -1.49 | 0.20 | -7.59 | 3.18E-14 | 2.67E-12 |
| Atpaf2 | 33.98 | -1.49 | 0.60 | -2.48 | 1.30E-02 | 9.83E-02 |
| Nop58 | 1231.43 | -1.49 | 0.27 | -5.48 | 4.21E-08 | 1.51E-06 |
| Zfp593 | 237.49 | -1.49 | 0.37 | -4.06 | 4.88E-05 | 9.39E-04 |
| Kif1a | 43.82 | -1.50 | 0.53 | -2.81 | 5.00E-03 | 4.73E-02 |
| Pakap | 10674.53 | -1.50 | 0.17 | -8.73 | 2.53E-18 | 3.13E-16 |
| Uap1 | 2819.13 | -1.50 | 0.23 | -6.48 | 8.98E-11 | 4.89E-09 |
| Rhoc | 3502.73 | -1.50 | 0.26 | -5.74 | 9.35E-09 | 3.73E-07 |
| Irak3 | 384.81 | -1.50 | 0.25 | -5.95 | 2.76E-09 | 1.21E-07 |
| Myd88 | 1125.03 | -1.50 | 0.26 | -5.76 | 8.64E-09 | 3.49E-07 |
| Pde12 | 827.53 | -1.51 | 0.30 | -5.10 | 3.49E-07 | 1.05E-05 |
| Enc1 | 993.99 | -1.51 | 0.23 | -6.51 | 7.47E-11 | 4.11E-09 |
| Trim16 | 366.22 | -1.51 | 0.31 | -4.80 | 1.62E-06 | 4.36E-05 |
| Dusp6 | 1459.58 | -1.51 | 0.25 | -6.06 | 1.33E-09 | 6.05E-08 |
| Csf3r | 140.42 | -1.51 | 0.48 | -3.15 | 1.64E-03 | 1.94E-02 |
| Gm38431 | 57.53 | -1.52 | 0.60 | -2.54 | 1.11E-02 | 8.76E-02 |
| Arrdc3 | 2359.30 | -1.52 | 0.19 | -7.99 | 1.39E-15 | 1.38E-13 |
| Bcl6 | 1149.58 | -1.52 | 0.16 | -9.63 | 6.22E-22 | 1.07E-19 |
| Crem | 562.12 | -1.53 | 0.36 | -4.26 | 2.03E-05 | 4.32E-04 |
| Tgfb1 | 1467.84 | -1.53 | 0.21 | -7.30 | 2.98E-13 | 2.22E-11 |
| Clec4n | 128.40 | -1.53 | 0.47 | -3.25 | 1.17E-03 | 1.46E-02 |
| Psd4 | 61.13 | -1.54 | 0.46 | -3.31 | 9.30E-04 | 1.22E-02 |
| Jak2 | 2170.22 | -1.54 | 0.23 | -6.76 | 1.40E-11 | 8.42E-10 |
| Sema7a | 3017.30 | -1.54 | 0.16 | -9.44 | 3.75E-21 | 5.67E-19 |
| Fancm | 98.75 | -1.54 | 0.34 | -4.49 | 7.07E-06 | 1.67E-04 |
| Sde2 | 805.69 | -1.54 | 0.29 | -5.35 | 8.85E-08 | 2.97E-06 |
| Rab8b | 882.98 | -1.54 | 0.24 | -6.47 | 1.00E-10 | 5.42E-09 |
| Itga5 | 3766.66 | -1.55 | 0.20 | -7.59 | 3.13E-14 | 2.65E-12 |
| Batf2 | 88.04 | -1.55 | 0.47 | -3.31 | 9.41E-04 | 1.23E-02 |
| Tapbp | 121.76 | -1.55 | 0.43 | -3.59 | 3.30E-04 | 5.01E-03 |
| Tmem88b | 111.58 | -1.55 | 0.47 | -3.32 | 8.94E-04 | 1.18E-02 |
| Relt | 145.98 | -1.55 | 0.31 | -5.08 | 3.68E-07 | 1.11E-05 |
| Sntb2 | 1485.48 | -1.55 | 0.27 | -5.73 | 1.02E-08 | 4.06E-07 |
| Stat3 | 6981.80 | -1.55 | 0.18 | -8.81 | 1.28E-18 | 1.65E-16 |
| Gpr132 | 119.73 | -1.56 | 0.49 | -3.16 | 1.58E-03 | 1.88E-02 |
| Olfr539 | 158.37 | -1.56 | 0.34 | -4.65 | 3.35E-06 | 8.48E-05 |
| Rnd3 | 1135.49 | -1.56 | 0.21 | -7.26 | 3.79E-13 | 2.76E-11 |
| Srxn1 | 1075.40 | -1.56 | 0.32 | -4.85 | 1.22E-06 | 3.36E-05 |
| Tgtp1 | 1081.03 | -1.56 | 0.31 | -5.04 | 4.74E-07 | 1.40E-05 |
| Asb4 | 345.60 | -1.56 | 0.40 | -3.90 | 9.44E-05 | 1.68E-03 |
| Tsku | 164.50 | -1.56 | 0.35 | -4.52 | 6.18E-06 | 1.48E-04 |
| Irgm1 | 1607.42 | -1.57 | 0.27 | -5.90 | 3.71E-09 | 1.58E-07 |
| Zc3hav1 | 1503.08 | -1.57 | 0.21 | -7.61 | 2.85E-14 | 2.47E-12 |
| Lag3 | 59.03 | -1.57 | 0.49 | -3.17 | 1.50E-03 | 1.80E-02 |
| Slc10a6 | 388.52 | -1.57 | 0.27 | -5.90 | 3.59E-09 | 1.54E-07 |
| Adgrd1 | 449.46 | -1.58 | 0.32 | -4.98 | 6.32E-07 | 1.83E-05 |
| Rela | 2553.97 | -1.58 | 0.25 | -6.21 | 5.21E-10 | 2.50E-08 |
| Cd80 | 40.83 | -1.58 | 0.59 | -2.66 | 7.84E-03 | 6.71E-02 |
| Parp14 | 3660.57 | -1.58 | 0.24 | -6.70 | 2.06E-11 | 1.21E-09 |
| Ccrl2 | 962.20 | -1.58 | 0.25 | -6.32 | 2.63E-10 | 1.34E-08 |
| Midn | 4694.19 | -1.58 | 0.25 | -6.36 | 2.07E-10 | 1.06E-08 |
| Tap1 | 1310.55 | -1.59 | 0.22 | -7.08 | 1.41E-12 | 9.56E-11 |
| Alkal2 | 47.10 | -1.59 | 0.59 | -2.68 | 7.28E-03 | 6.32E-02 |
| Cebpb | 3211.66 | -1.60 | 0.21 | -7.60 | 2.91E-14 | 2.51E-12 |
| Helz2 | 4802.12 | -1.60 | 0.24 | -6.62 | 3.57E-11 | 2.07E-09 |
| Dcun1d3 | 468.42 | -1.61 | 0.25 | -6.43 | 1.30E-10 | 6.83E-09 |
| Igtp | 1109.19 | -1.61 | 0.33 | -4.92 | 8.56E-07 | 2.42E-05 |
| Isg20 | 218.71 | -1.61 | 0.32 | -5.05 | 4.39E-07 | 1.31E-05 |
| Sema3f | 1303.50 | -1.61 | 0.20 | -7.87 | 3.50E-15 | 3.28E-13 |
| Usp27x | 66.45 | -1.61 | 0.57 | -2.81 | 4.92E-03 | 4.68E-02 |
| Flnc | 23072.28 | -1.62 | 0.20 | -8.25 | 1.62E-16 | 1.77E-14 |
| Fzd5 | 367.25 | -1.62 | 0.28 | -5.76 | 8.65E-09 | 3.49E-07 |
| Cytip | 39.36 | -1.62 | 0.65 | -2.48 | 1.31E-02 | 9.84E-02 |
| Ptafr | 334.48 | -1.62 | 0.36 | -4.52 | 6.07E-06 | 1.46E-04 |
| Mpp2 | 290.22 | -1.63 | 0.26 | -6.24 | 4.43E-10 | 2.16E-08 |
| Spi1 | 249.24 | -1.63 | 0.31 | -5.35 | 8.98E-08 | 3.01E-06 |
| Nuak1 | 1407.98 | -1.63 | 0.21 | -7.91 | 2.63E-15 | 2.52E-13 |
| Icosl | 785.51 | -1.64 | 0.30 | -5.54 | 2.94E-08 | 1.09E-06 |
| Lilr4b | 1094.36 | -1.64 | 0.29 | -5.61 | 2.02E-08 | 7.74E-07 |
| Txnrd1 | 2922.73 | -1.64 | 0.23 | -7.03 | 2.08E-12 | 1.38E-10 |
| Ntrk2 | 41.33 | -1.64 | 0.59 | -2.79 | 5.19E-03 | 4.88E-02 |
| Zfp729a | 1923.86 | -1.64 | 0.19 | -8.60 | 8.17E-18 | 9.82E-16 |
| Nfkb1 | 3050.20 | -1.64 | 0.18 | -9.07 | 1.18E-19 | 1.63E-17 |
| Snx20 | 74.57 | -1.64 | 0.50 | -3.32 | 8.98E-04 | 1.18E-02 |
| Zswim4 | 803.33 | -1.65 | 0.27 | -6.14 | 8.09E-10 | 3.79E-08 |
| H2-Q10 | 430.31 | -1.65 | 0.26 | -6.28 | 3.37E-10 | 1.69E-08 |
| Adamts1 | 6798.60 | -1.65 | 0.21 | -7.74 | 1.01E-14 | 9.18E-13 |
| Crtc2 | 1431.03 | -1.65 | 0.19 | -8.60 | 8.23E-18 | 9.82E-16 |
| Ncf4 | 75.00 | -1.65 | 0.44 | -3.78 | 1.54E-04 | 2.61E-03 |
| Sash1 | 2309.97 | -1.65 | 0.17 | -9.71 | 2.65E-22 | 4.88E-20 |
| Ngfr | 40.77 | -1.65 | 0.56 | -2.95 | 3.16E-03 | 3.27E-02 |
| Lmcd1 | 1874.99 | -1.66 | 0.25 | -6.70 | 2.09E-11 | 1.23E-09 |
| Otud1 | 3931.37 | -1.66 | 0.27 | -6.24 | 4.44E-10 | 2.16E-08 |
| Aacs | 235.87 | -1.67 | 0.27 | -6.19 | 6.19E-10 | 2.92E-08 |
| Ell2 | 874.07 | -1.68 | 0.20 | -8.42 | 3.73E-17 | 4.19E-15 |
| Ccl9 | 708.24 | -1.68 | 0.23 | -7.15 | 8.59E-13 | 6.02E-11 |
| H2-Q5 | 699.09 | -1.68 | 0.23 | -7.27 | 3.52E-13 | 2.59E-11 |
| Sh2b2 | 74.51 | -1.69 | 0.44 | -3.86 | 1.16E-04 | 2.01E-03 |
| Izumo1 | 56.93 | -1.69 | 0.49 | -3.42 | 6.24E-04 | 8.63E-03 |
| Ptprj | 660.81 | -1.69 | 0.24 | -6.94 | 3.87E-12 | 2.51E-10 |
| Egr1 | 23008.03 | -1.69 | 0.54 | -3.14 | 1.67E-03 | 1.97E-02 |
| Igf2bp2 | 236.82 | -1.70 | 0.31 | -5.45 | 5.01E-08 | 1.78E-06 |
| Ripk1 | 981.78 | -1.70 | 0.32 | -5.34 | 9.24E-08 | 3.09E-06 |
| Il4i1 | 44.90 | -1.70 | 0.57 | -2.99 | 2.76E-03 | 2.93E-02 |
| Csf2rb | 176.14 | -1.70 | 0.34 | -4.95 | 7.37E-07 | 2.12E-05 |
| Plekho2 | 1685.74 | -1.70 | 0.30 | -5.67 | 1.39E-08 | 5.43E-07 |
| Foxf1 | 40.92 | -1.71 | 0.57 | -3.00 | 2.74E-03 | 2.92E-02 |
| Eda2r | 242.97 | -1.71 | 0.37 | -4.68 | 2.84E-06 | 7.28E-05 |
| Il1r1 | 1125.25 | -1.71 | 0.29 | -6.00 | 2.02E-09 | 8.98E-08 |
| Gm18853 | 287.16 | -1.72 | 0.47 | -3.68 | 2.36E-04 | 3.76E-03 |
| Baz1a | 653.17 | -1.72 | 0.20 | -8.74 | 2.38E-18 | 2.98E-16 |
| Adamts9 | 2395.26 | -1.72 | 0.23 | -7.42 | 1.18E-13 | 9.37E-12 |
| Rnf24 | 390.06 | -1.73 | 0.23 | -7.39 | 1.48E-13 | 1.16E-11 |
| Rab32 | 101.98 | -1.73 | 0.37 | -4.63 | 3.72E-06 | 9.31E-05 |
| Hp | 44.80 | -1.73 | 0.63 | -2.75 | 5.88E-03 | 5.35E-02 |
| Eno2 | 71.59 | -1.74 | 0.48 | -3.58 | 3.41E-04 | 5.16E-03 |
| Kcne4 | 333.58 | -1.74 | 0.29 | -5.91 | 3.42E-09 | 1.47E-07 |
| Oas1g | 112.66 | -1.74 | 0.43 | -4.09 | 4.30E-05 | 8.38E-04 |
| Mafk | 3398.42 | -1.75 | 0.20 | -8.86 | 7.77E-19 | 1.02E-16 |
| Mapk6 | 1813.56 | -1.75 | 0.17 | -10.20 | 1.95E-24 | 4.58E-22 |
| Ier3 | 4417.46 | -1.75 | 0.25 | -7.05 | 1.80E-12 | 1.21E-10 |
| Phlda1 | 2734.93 | -1.75 | 0.21 | -8.46 | 2.68E-17 | 3.07E-15 |
| Slc2a3 | 127.48 | -1.76 | 0.41 | -4.33 | 1.51E-05 | 3.32E-04 |
| Eya2 | 66.91 | -1.76 | 0.52 | -3.38 | 7.35E-04 | 9.91E-03 |
| Mycbpap | 30.29 | -1.76 | 0.70 | -2.53 | 1.15E-02 | 8.97E-02 |
| Adam8 | 61.56 | -1.76 | 0.58 | -3.03 | 2.44E-03 | 2.66E-02 |
| Exoc3l4 | 201.50 | -1.76 | 0.33 | -5.42 | 5.84E-08 | 2.04E-06 |
| Ppp1r18 | 1738.47 | -1.76 | 0.20 | -8.96 | 3.31E-19 | 4.46E-17 |
| Slc1a1 | 284.31 | -1.76 | 0.25 | -7.18 | 6.89E-13 | 4.91E-11 |
| Zfp36 | 14734.93 | -1.77 | 0.26 | -6.90 | 5.16E-12 | 3.29E-10 |
| Mob3c | 546.01 | -1.77 | 0.33 | -5.39 | 7.05E-08 | 2.42E-06 |
| Ypel2 | 976.75 | -1.78 | 0.20 | -8.88 | 6.90E-19 | 9.12E-17 |
| Tnfrsf1b | 765.25 | -1.78 | 0.22 | -8.19 | 2.55E-16 | 2.72E-14 |
| Ada | 45.44 | -1.79 | 0.64 | -2.81 | 4.94E-03 | 4.69E-02 |
| Lrrc32 | 1475.90 | -1.79 | 0.28 | -6.46 | 1.03E-10 | 5.54E-09 |
| Agpat4 | 256.61 | -1.79 | 0.38 | -4.67 | 3.04E-06 | 7.73E-05 |
| Jdp2 | 585.90 | -1.79 | 0.21 | -8.41 | 4.24E-17 | 4.73E-15 |
| Peg10 | 96.54 | -1.79 | 0.43 | -4.13 | 3.68E-05 | 7.30E-04 |
| Il1b | 842.71 | -1.79 | 0.34 | -5.25 | 1.53E-07 | 4.94E-06 |
| Fam124b | 42.85 | -1.80 | 0.63 | -2.86 | 4.19E-03 | 4.13E-02 |
| Fos | 16851.65 | -1.80 | 0.19 | -9.33 | 1.09E-20 | 1.59E-18 |
| H2-Q6 | 2540.80 | -1.80 | 0.17 | -10.63 | 2.27E-26 | 6.56E-24 |
| Gch1 | 722.86 | -1.80 | 0.25 | -7.32 | 2.57E-13 | 1.94E-11 |
| Smad7 | 998.99 | -1.81 | 0.19 | -9.65 | 5.11E-22 | 9.02E-20 |
| Tnip3 | 331.06 | -1.81 | 0.30 | -6.03 | 1.61E-09 | 7.23E-08 |
| Ccl8 | 43.64 | -1.82 | 0.72 | -2.51 | 1.21E-02 | 9.32E-02 |
| Mxd1 | 471.26 | -1.82 | 0.28 | -6.59 | 4.38E-11 | 2.49E-09 |
| 2200002D01Rik | 44.70 | -1.83 | 0.59 | -3.09 | 2.00E-03 | 2.27E-02 |
| Dusp10 | 377.84 | -1.83 | 0.21 | -8.81 | 1.25E-18 | 1.62E-16 |
| Wnt10b | 22.35 | -1.83 | 0.70 | -2.63 | 8.58E-03 | 7.19E-02 |
| Mthfd2 | 198.30 | -1.84 | 0.43 | -4.29 | 1.82E-05 | 3.92E-04 |
| Ralgds | 1370.76 | -1.84 | 0.18 | -10.48 | 1.03E-25 | 2.74E-23 |
| Trib1 | 1800.73 | -1.84 | 0.23 | -7.99 | 1.32E-15 | 1.33E-13 |
| Cd86 | 250.65 | -1.84 | 0.30 | -6.20 | 5.58E-10 | 2.67E-08 |
| Cybb | 1049.66 | -1.84 | 0.31 | -6.02 | 1.78E-09 | 7.93E-08 |
| Creb5 | 780.11 | -1.84 | 0.24 | -7.59 | 3.12E-14 | 2.65E-12 |
| Cgas | 175.30 | -1.84 | 0.36 | -5.10 | 3.46E-07 | 1.05E-05 |
| Emp1 | 4833.34 | -1.84 | 0.54 | -3.42 | 6.37E-04 | 8.79E-03 |
| Ifit3b | 450.44 | -1.84 | 0.31 | -5.97 | 2.33E-09 | 1.02E-07 |
| Gm15542 | 297.03 | -1.85 | 0.35 | -5.23 | 1.74E-07 | 5.56E-06 |
| Rnf122 | 449.95 | -1.85 | 0.35 | -5.29 | 1.21E-07 | 3.93E-06 |
| Hdx | 65.57 | -1.86 | 0.45 | -4.10 | 4.09E-05 | 8.04E-04 |
| Upp1 | 314.52 | -1.86 | 0.27 | -6.85 | 7.63E-12 | 4.72E-10 |
| Gclc | 858.95 | -1.86 | 0.19 | -9.87 | 5.47E-23 | 1.06E-20 |
| Egfr | 531.89 | -1.87 | 0.27 | -6.93 | 4.21E-12 | 2.72E-10 |
| Pnp | 1317.17 | -1.87 | 0.27 | -6.88 | 5.90E-12 | 3.73E-10 |
| Ppard | 59.76 | -1.87 | 0.55 | -3.41 | 6.55E-04 | 9.00E-03 |
| Pilra | 43.59 | -1.87 | 0.57 | -3.31 | 9.32E-04 | 1.22E-02 |
| C5ar1 | 509.03 | -1.88 | 0.28 | -6.83 | 8.30E-12 | 5.11E-10 |
| Gm15459 | 35.70 | -1.88 | 0.71 | -2.64 | 8.22E-03 | 6.95E-02 |
| Cd44 | 727.60 | -1.88 | 0.25 | -7.58 | 3.48E-14 | 2.89E-12 |
| Sh3pxd2b | 673.47 | -1.88 | 0.19 | -9.92 | 3.44E-23 | 6.87E-21 |
| Il33 | 284.84 | -1.88 | 0.75 | -2.52 | 1.19E-02 | 9.19E-02 |
| Micall2 | 239.68 | -1.89 | 0.25 | -7.47 | 7.91E-14 | 6.37E-12 |
| Rhbdf2 | 1415.19 | -1.89 | 0.18 | -10.21 | 1.70E-24 | 4.06E-22 |
| Adgrg6 | 72.61 | -1.90 | 0.43 | -4.39 | 1.13E-05 | 2.55E-04 |
| Akap12 | 2816.25 | -1.90 | 0.23 | -8.27 | 1.39E-16 | 1.53E-14 |
| Lratd1 | 56.97 | -1.90 | 0.53 | -3.60 | 3.13E-04 | 4.78E-03 |
| Epha2 | 929.44 | -1.91 | 0.25 | -7.65 | 1.96E-14 | 1.72E-12 |
| Fam107b | 318.37 | -1.91 | 0.32 | -6.06 | 1.36E-09 | 6.16E-08 |
| Tnfrsf10b | 703.66 | -1.92 | 0.32 | -5.93 | 3.04E-09 | 1.33E-07 |
| Spdl1 | 26.45 | -1.92 | 0.73 | -2.63 | 8.61E-03 | 7.21E-02 |
| Sertad1 | 1052.66 | -1.92 | 0.59 | -3.27 | 1.07E-03 | 1.36E-02 |
| Rsad2 | 3481.66 | -1.92 | 0.26 | -7.32 | 2.52E-13 | 1.91E-11 |
| Pde4b | 3854.32 | -1.92 | 0.25 | -7.58 | 3.41E-14 | 2.85E-12 |
| Dennd4a | 1010.07 | -1.93 | 0.19 | -9.90 | 4.12E-23 | 8.12E-21 |
| Nts | 194.23 | -1.93 | 0.35 | -5.56 | 2.69E-08 | 1.00E-06 |
| Azin1 | 3923.31 | -1.94 | 0.22 | -9.04 | 1.63E-19 | 2.24E-17 |
| Peli1 | 1125.60 | -1.94 | 0.22 | -8.71 | 2.91E-18 | 3.57E-16 |
| Adm | 576.58 | -1.94 | 0.19 | -10.17 | 2.70E-24 | 6.02E-22 |
| Ifi47 | 454.00 | -1.95 | 0.29 | -6.62 | 3.70E-11 | 2.13E-09 |
| Serpine1 | 7869.17 | -1.95 | 0.16 | -11.91 | 1.04E-32 | 5.24E-30 |
| Lhfpl2 | 361.98 | -1.95 | 0.22 | -8.77 | 1.72E-18 | 2.19E-16 |
| Rab11fip1 | 70.40 | -1.95 | 0.46 | -4.24 | 2.22E-05 | 4.69E-04 |
| Rbm47 | 46.49 | -1.95 | 0.69 | -2.81 | 4.89E-03 | 4.66E-02 |
| Irak2 | 876.26 | -1.96 | 0.25 | -7.95 | 1.93E-15 | 1.87E-13 |
| Trex1 | 715.24 | -1.96 | 0.28 | -7.07 | 1.50E-12 | 1.02E-10 |
| Cx3cl1 | 729.68 | -1.96 | 0.26 | -7.61 | 2.77E-14 | 2.42E-12 |
| Gm9973 | 52.39 | -1.96 | 0.73 | -2.71 | 6.82E-03 | 6.01E-02 |
| Rtp4 | 629.11 | -1.97 | 0.28 | -6.92 | 4.42E-12 | 2.84E-10 |
| Arid5b | 1998.86 | -1.97 | 0.26 | -7.47 | 7.99E-14 | 6.39E-12 |
| Orai2 | 122.22 | -1.97 | 0.39 | -5.11 | 3.15E-07 | 9.62E-06 |
| Nfkbid | 273.04 | -1.98 | 0.76 | -2.60 | 9.40E-03 | 7.69E-02 |
| Gm42417 | 107.10 | -1.98 | 0.54 | -3.63 | 2.81E-04 | 4.35E-03 |
| Errfi1 | 3216.23 | -1.99 | 0.23 | -8.52 | 1.60E-17 | 1.87E-15 |
| Arntl | 479.45 | -2.00 | 0.27 | -7.41 | 1.28E-13 | 1.01E-11 |
| Cfb | 173.51 | -2.00 | 0.35 | -5.70 | 1.18E-08 | 4.63E-07 |
| Nr4a3 | 3817.12 | -2.00 | 0.20 | -10.16 | 3.12E-24 | 6.84E-22 |
| Slamf7 | 121.23 | -2.01 | 0.43 | -4.62 | 3.87E-06 | 9.64E-05 |
| Slc5a3 | 282.75 | -2.01 | 0.25 | -7.92 | 2.36E-15 | 2.28E-13 |
| Gbp5 | 2623.48 | -2.01 | 0.27 | -7.36 | 1.82E-13 | 1.41E-11 |
| Oas3 | 56.28 | -2.01 | 0.61 | -3.30 | 9.51E-04 | 1.23E-02 |
| Xirp1 | 18173.22 | -2.01 | 0.20 | -10.32 | 6.00E-25 | 1.48E-22 |
| Eif1a | 512.80 | -2.01 | 0.28 | -7.13 | 9.68E-13 | 6.72E-11 |
| Junb | 14852.73 | -2.02 | 0.28 | -7.27 | 3.58E-13 | 2.62E-11 |
| Slc41a2 | 109.56 | -2.02 | 0.40 | -5.00 | 5.71E-07 | 1.66E-05 |
| Panx1 | 161.57 | -2.02 | 0.31 | -6.56 | 5.52E-11 | 3.09E-09 |
| Tnip1 | 3098.02 | -2.03 | 0.16 | -12.68 | 7.21E-37 | 4.67E-34 |
| Rasgef1a | 28.55 | -2.04 | 0.66 | -3.12 | 1.84E-03 | 2.12E-02 |
| Rasd1 | 442.18 | -2.04 | 0.21 | -9.87 | 5.60E-23 | 1.07E-20 |
| Gm614 | 46.22 | -2.05 | 0.66 | -3.10 | 1.96E-03 | 2.24E-02 |
| Wt1 | 596.03 | -2.05 | 0.70 | -2.92 | 3.45E-03 | 3.52E-02 |
| Bcr | 979.06 | -2.05 | 0.35 | -5.79 | 7.05E-09 | 2.89E-07 |
| Arid5a | 3397.13 | -2.05 | 0.59 | -3.49 | 4.83E-04 | 6.95E-03 |
| Ppp1r15a | 5085.85 | -2.05 | 0.22 | -9.45 | 3.25E-21 | 4.96E-19 |
| Ifi207 | 1356.65 | -2.06 | 0.31 | -6.57 | 5.06E-11 | 2.85E-09 |
| Trim30b | 33.58 | -2.06 | 0.66 | -3.14 | 1.71E-03 | 2.02E-02 |
| Adgrg3 | 147.25 | -2.06 | 0.45 | -4.56 | 5.16E-06 | 1.26E-04 |
| Hoxa5 | 43.29 | -2.06 | 0.61 | -3.40 | 6.85E-04 | 9.36E-03 |
| Slc25a25 | 1507.56 | -2.06 | 0.33 | -6.27 | 3.57E-10 | 1.78E-08 |
| Inhbb | 218.58 | -2.08 | 0.39 | -5.30 | 1.17E-07 | 3.80E-06 |
| Inhba | 292.56 | -2.09 | 0.44 | -4.76 | 1.97E-06 | 5.22E-05 |
| Klhl40 | 1357.98 | -2.10 | 0.29 | -7.35 | 2.03E-13 | 1.56E-11 |
| Ifi206 | 56.19 | -2.10 | 0.51 | -4.13 | 3.63E-05 | 7.24E-04 |
| Hilpda | 282.26 | -2.10 | 0.46 | -4.61 | 4.10E-06 | 1.02E-04 |
| Fgl2 | 3691.36 | -2.11 | 0.26 | -8.11 | 5.15E-16 | 5.34E-14 |
| Fosl2 | 6752.69 | -2.12 | 0.24 | -8.65 | 5.02E-18 | 6.09E-16 |
| Hsp90aa1 | 13914.91 | -2.12 | 0.23 | -9.39 | 5.79E-21 | 8.56E-19 |
| Kif11 | 125.31 | -2.13 | 0.39 | -5.49 | 4.07E-08 | 1.47E-06 |
| Sowahc | 434.00 | -2.13 | 0.26 | -8.24 | 1.77E-16 | 1.91E-14 |
| Gpr68 | 26.57 | -2.13 | 0.70 | -3.05 | 2.25E-03 | 2.50E-02 |
| Sema4c | 2342.81 | -2.14 | 0.22 | -9.70 | 2.91E-22 | 5.27E-20 |
| Tent5c | 97.42 | -2.14 | 0.51 | -4.24 | 2.22E-05 | 4.69E-04 |
| Ifit3 | 1802.23 | -2.14 | 0.32 | -6.74 | 1.61E-11 | 9.57E-10 |
| Ifi204 | 1012.55 | -2.15 | 0.35 | -6.19 | 5.97E-10 | 2.85E-08 |
| Gm6377 | 230.26 | -2.15 | 0.49 | -4.39 | 1.14E-05 | 2.57E-04 |
| Ptgdr | 15.55 | -2.16 | 0.86 | -2.50 | 1.23E-02 | 9.39E-02 |
| Ndrg1 | 39.35 | -2.16 | 0.54 | -4.01 | 6.15E-05 | 1.15E-03 |
| Serpina3i | 24.87 | -2.16 | 0.86 | -2.51 | 1.20E-02 | 9.24E-02 |
| Tgif1 | 476.86 | -2.18 | 0.32 | -6.88 | 5.91E-12 | 3.73E-10 |
| Il2rg | 1000.79 | -2.19 | 0.27 | -8.14 | 3.96E-16 | 4.18E-14 |
| Fosb | 16107.20 | -2.21 | 0.56 | -3.92 | 8.84E-05 | 1.59E-03 |
| Nuak2 | 533.10 | -2.21 | 0.30 | -7.50 | 6.56E-14 | 5.31E-12 |
| Il4ra | 2208.10 | -2.21 | 0.28 | -8.00 | 1.22E-15 | 1.23E-13 |
| Atf3 | 14101.49 | -2.22 | 0.22 | -10.27 | 9.53E-25 | 2.32E-22 |
| Nfkbiz | 4582.44 | -2.22 | 0.62 | -3.56 | 3.78E-04 | 5.63E-03 |
| Cxcl16 | 1101.43 | -2.23 | 0.24 | -9.48 | 2.49E-21 | 3.89E-19 |
| Dll1 | 496.19 | -2.23 | 0.36 | -6.26 | 3.86E-10 | 1.91E-08 |
| Slfn8 | 435.56 | -2.23 | 0.33 | -6.69 | 2.18E-11 | 1.28E-09 |
| Col12a1 | 93.37 | -2.24 | 0.48 | -4.64 | 3.41E-06 | 8.60E-05 |
| Arrdc4 | 1401.01 | -2.24 | 0.23 | -9.66 | 4.46E-22 | 7.98E-20 |
| Osmr | 2424.71 | -2.24 | 0.26 | -8.76 | 1.89E-18 | 2.38E-16 |
| Zbp1 | 139.78 | -2.24 | 0.38 | -5.89 | 3.91E-09 | 1.66E-07 |
| Ripk2 | 725.55 | -2.25 | 0.31 | -7.17 | 7.61E-13 | 5.39E-11 |
| Gm49392 | 53.45 | -2.25 | 0.54 | -4.18 | 2.86E-05 | 5.83E-04 |
| Slc15a3 | 698.67 | -2.25 | 0.24 | -9.56 | 1.22E-21 | 1.98E-19 |
| Ccno | 55.31 | -2.25 | 0.64 | -3.51 | 4.52E-04 | 6.56E-03 |
| Tlr2 | 992.61 | -2.26 | 0.36 | -6.29 | 3.27E-10 | 1.64E-08 |
| Ifi209 | 132.93 | -2.26 | 0.38 | -5.92 | 3.15E-09 | 1.36E-07 |
| Slc16a13 | 363.86 | -2.26 | 0.36 | -6.23 | 4.53E-10 | 2.19E-08 |
| Tnfaip8l1 | 165.04 | -2.27 | 0.35 | -6.44 | 1.23E-10 | 6.52E-09 |
| Cd40 | 437.52 | -2.27 | 0.31 | -7.28 | 3.35E-13 | 2.48E-11 |
| Pdpn | 331.92 | -2.28 | 0.36 | -6.36 | 1.99E-10 | 1.03E-08 |
| Pou3f1 | 29.83 | -2.28 | 0.64 | -3.57 | 3.54E-04 | 5.32E-03 |
| Lonrf3 | 74.11 | -2.28 | 0.54 | -4.23 | 2.33E-05 | 4.86E-04 |
| Iigp1 | 264.26 | -2.28 | 0.39 | -5.82 | 5.89E-09 | 2.44E-07 |
| Ubash3b | 250.18 | -2.29 | 0.89 | -2.57 | 1.02E-02 | 8.12E-02 |
| Irf8 | 774.23 | -2.30 | 0.34 | -6.78 | 1.17E-11 | 7.11E-10 |
| Rgs16 | 858.69 | -2.31 | 0.62 | -3.71 | 2.08E-04 | 3.40E-03 |
| Slc7a5 | 377.16 | -2.32 | 0.41 | -5.68 | 1.37E-08 | 5.39E-07 |
| Slc16a3 | 234.51 | -2.32 | 0.40 | -5.75 | 8.95E-09 | 3.60E-07 |
| Slc7a2 | 390.72 | -2.33 | 0.36 | -6.55 | 5.73E-11 | 3.20E-09 |
| Frat2 | 287.91 | -2.33 | 0.38 | -6.09 | 1.14E-09 | 5.25E-08 |
| Ddit4 | 2096.99 | -2.33 | 0.54 | -4.32 | 1.55E-05 | 3.39E-04 |
| Cxcl9 | 1770.01 | -2.33 | 0.31 | -7.44 | 1.03E-13 | 8.19E-12 |
| Tchh | 43.53 | -2.34 | 0.71 | -3.29 | 9.93E-04 | 1.28E-02 |
| Eid3 | 39.82 | -2.34 | 0.64 | -3.68 | 2.34E-04 | 3.74E-03 |
| Bmp2 | 192.57 | -2.35 | 0.33 | -7.13 | 9.95E-13 | 6.87E-11 |
| Lcp2 | 385.11 | -2.35 | 0.27 | -8.57 | 1.01E-17 | 1.19E-15 |
| Ccl11 | 206.00 | -2.36 | 0.37 | -6.41 | 1.47E-10 | 7.68E-09 |
| Il17ra | 681.40 | -2.37 | 0.27 | -8.89 | 6.38E-19 | 8.51E-17 |
| Itpkc | 703.51 | -2.38 | 0.28 | -8.45 | 2.92E-17 | 3.31E-15 |
| Slfn2 | 1032.14 | -2.38 | 0.24 | -9.81 | 1.05E-22 | 1.99E-20 |
| Gm49342 | 295.02 | -2.38 | 0.30 | -7.90 | 2.78E-15 | 2.64E-13 |
| Relb | 1177.76 | -2.39 | 0.22 | -10.73 | 7.19E-27 | 2.17E-24 |
| Kdm6b | 5461.91 | -2.39 | 0.25 | -9.59 | 8.52E-22 | 1.45E-19 |
| Plek | 1590.09 | -2.39 | 0.31 | -7.77 | 8.08E-15 | 7.37E-13 |
| Tifa | 523.66 | -2.39 | 0.37 | -6.42 | 1.35E-10 | 7.09E-09 |
| Cebpd | 2735.69 | -2.39 | 0.23 | -10.18 | 2.40E-24 | 5.45E-22 |
| Tfpi2 | 241.58 | -2.39 | 0.48 | -5.01 | 5.37E-07 | 1.57E-05 |
| Gm45799 | 43.34 | -2.39 | 0.71 | -3.38 | 7.18E-04 | 9.70E-03 |
| Rel | 1536.61 | -2.40 | 0.23 | -10.44 | 1.69E-25 | 4.35E-23 |
| Dnaja1 | 6435.66 | -2.40 | 0.21 | -11.52 | 9.91E-31 | 3.85E-28 |
| Irs2 | 4712.18 | -2.40 | 0.23 | -10.36 | 3.65E-25 | 9.18E-23 |
| Rasgef1b | 1271.90 | -2.41 | 0.21 | -11.44 | 2.55E-30 | 9.63E-28 |
| Mt2 | 4403.46 | -2.41 | 0.50 | -4.86 | 1.16E-06 | 3.18E-05 |
| Gm11843 | 20.48 | -2.41 | 0.92 | -2.63 | 8.63E-03 | 7.22E-02 |
| Hspa13 | 30.86 | -2.43 | 0.77 | -3.16 | 1.60E-03 | 1.90E-02 |
| Litaf | 2269.00 | -2.44 | 0.23 | -10.45 | 1.47E-25 | 3.85E-23 |
| Nfkbia | 7170.52 | -2.45 | 0.23 | -10.51 | 7.53E-26 | 2.14E-23 |
| Gdf15 | 218.19 | -2.45 | 0.36 | -6.85 | 7.46E-12 | 4.63E-10 |
| Ifih1 | 1365.79 | -2.45 | 0.26 | -9.63 | 6.23E-22 | 1.07E-19 |
| Ngf | 744.89 | -2.46 | 0.32 | -7.68 | 1.62E-14 | 1.44E-12 |
| Ccl12 | 178.42 | -2.48 | 0.50 | -4.98 | 6.36E-07 | 1.84E-05 |
| Slfn10-ps | 21.32 | -2.49 | 0.87 | -2.85 | 4.34E-03 | 4.25E-02 |
| Hck | 167.04 | -2.49 | 0.50 | -4.97 | 6.63E-07 | 1.91E-05 |
| Dnajb1 | 4948.41 | -2.50 | 0.22 | -11.15 | 6.94E-29 | 2.30E-26 |
| Birc3 | 2089.86 | -2.50 | 0.32 | -7.84 | 4.59E-15 | 4.28E-13 |
| Hsph1 | 5986.94 | -2.50 | 0.20 | -12.48 | 9.63E-36 | 5.46E-33 |
| Zc3h12a | 1302.50 | -2.52 | 0.57 | -4.45 | 8.56E-06 | 1.98E-04 |
| Ackr1 | 17.49 | -2.54 | 0.92 | -2.76 | 5.70E-03 | 5.24E-02 |
| Tfec | 52.35 | -2.54 | 0.59 | -4.33 | 1.48E-05 | 3.27E-04 |
| Pgf | 548.77 | -2.55 | 0.32 | -7.87 | 3.47E-15 | 3.28E-13 |
| Mx2 | 767.86 | -2.55 | 0.35 | -7.20 | 5.82E-13 | 4.17E-11 |
| Gfpt2 | 2728.39 | -2.55 | 0.24 | -10.49 | 9.41E-26 | 2.56E-23 |
| Cd69 | 31.05 | -2.57 | 0.97 | -2.65 | 8.05E-03 | 6.85E-02 |
| Rgs1 | 85.72 | -2.58 | 0.47 | -5.49 | 4.06E-08 | 1.46E-06 |
| Runx1 | 245.98 | -2.58 | 0.27 | -9.50 | 2.02E-21 | 3.23E-19 |
| Dusp2 | 217.42 | -2.58 | 0.36 | -7.14 | 9.43E-13 | 6.58E-11 |
| Ifrd1 | 5845.78 | -2.59 | 0.26 | -9.92 | 3.44E-23 | 6.87E-21 |
| Casp4 | 989.08 | -2.61 | 0.61 | -4.27 | 1.99E-05 | 4.23E-04 |
| Gm5970 | 28.86 | -2.61 | 0.81 | -3.21 | 1.33E-03 | 1.62E-02 |
| Dusp5 | 1101.95 | -2.61 | 0.29 | -9.08 | 1.11E-19 | 1.56E-17 |
| Gm45551 | 1789.66 | -2.62 | 0.19 | -13.63 | 2.63E-42 | 2.38E-39 |
| Nfil3 | 1300.51 | -2.62 | 0.26 | -10.15 | 3.37E-24 | 7.29E-22 |
| Syt5 | 29.78 | -2.63 | 0.70 | -3.77 | 1.65E-04 | 2.76E-03 |
| AC109138.2 | 2374.72 | -2.63 | 0.16 | -16.19 | 6.43E-59 | 2.19E-55 |
| Stc1 | 411.08 | -2.64 | 0.28 | -9.47 | 2.71E-21 | 4.19E-19 |
| Arl13b | 1182.68 | -2.64 | 0.20 | -12.94 | 2.68E-38 | 1.92E-35 |
| Cfap69 | 120.16 | -2.64 | 0.56 | -4.70 | 2.56E-06 | 6.59E-05 |
| Bcl2a1b | 219.24 | -2.65 | 0.37 | -7.10 | 1.26E-12 | 8.65E-11 |
| Sdc4 | 2583.33 | -2.66 | 0.23 | -11.70 | 1.21E-31 | 4.97E-29 |
| Tnfaip2 | 6930.34 | -2.67 | 0.19 | -14.25 | 4.24E-46 | 4.81E-43 |
| Klf5 | 35.19 | -2.67 | 0.61 | -4.36 | 1.29E-05 | 2.88E-04 |
| Traf1 | 217.00 | -2.67 | 0.39 | -6.86 | 6.69E-12 | 4.20E-10 |
| Irf1 | 7395.79 | -2.68 | 0.28 | -9.57 | 1.03E-21 | 1.69E-19 |
| Hamp | 20.05 | -2.68 | 0.81 | -3.31 | 9.35E-04 | 1.22E-02 |
| Socs3 | 8192.75 | -2.69 | 0.50 | -5.40 | 6.52E-08 | 2.25E-06 |
| Tmem132e | 32.97 | -2.69 | 0.66 | -4.08 | 4.49E-05 | 8.71E-04 |
| Ccl19 | 77.21 | -2.70 | 0.46 | -5.82 | 5.77E-09 | 2.39E-07 |
| Ptpre | 736.80 | -2.73 | 0.27 | -9.94 | 2.85E-23 | 5.87E-21 |
| Tpbg | 57.47 | -2.74 | 0.56 | -4.91 | 9.28E-07 | 2.60E-05 |
| Asprv1 | 14.37 | -2.76 | 1.11 | -2.49 | 1.29E-02 | 9.77E-02 |
| Ifi211 | 1286.83 | -2.76 | 0.58 | -4.75 | 1.99E-06 | 5.26E-05 |
| B4galt5 | 1285.02 | -2.77 | 0.21 | -13.01 | 1.13E-38 | 8.52E-36 |
| Vcam1 | 5525.57 | -2.78 | 0.24 | -11.72 | 1.02E-31 | 4.34E-29 |
| Gem | 1714.27 | -2.78 | 0.24 | -11.81 | 3.66E-32 | 1.66E-29 |
| Sec1 | 23.48 | -2.80 | 0.92 | -3.04 | 2.35E-03 | 2.58E-02 |
| Marcksl1 | 2146.77 | -2.80 | 0.30 | -9.30 | 1.42E-20 | 2.06E-18 |
| Nlrp3 | 572.02 | -2.81 | 0.23 | -12.32 | 6.87E-35 | 3.74E-32 |
| Csf1 | 9083.75 | -2.81 | 0.23 | -12.05 | 1.99E-33 | 1.04E-30 |
| Asns | 219.97 | -2.82 | 0.48 | -5.91 | 3.35E-09 | 1.45E-07 |
| Slfn1 | 30.95 | -2.82 | 0.75 | -3.75 | 1.80E-04 | 2.99E-03 |
| Plau | 906.24 | -2.84 | 0.66 | -4.32 | 1.59E-05 | 3.46E-04 |
| Gadd45g | 3765.76 | -2.85 | 0.20 | -14.43 | 3.30E-47 | 4.08E-44 |
| Tnc | 256.17 | -2.86 | 0.88 | -3.23 | 1.22E-03 | 1.52E-02 |
| Adamts8 | 177.35 | -2.86 | 0.40 | -7.13 | 1.02E-12 | 7.00E-11 |
| Gm49339 | 301.36 | -2.87 | 0.29 | -9.79 | 1.21E-22 | 2.26E-20 |
| Hmox1 | 1517.58 | -2.88 | 0.57 | -5.04 | 4.75E-07 | 1.40E-05 |
| Pik3r5 | 483.91 | -2.88 | 0.22 | -12.91 | 3.79E-38 | 2.58E-35 |
| Myc | 1523.25 | -2.89 | 0.26 | -10.92 | 8.79E-28 | 2.78E-25 |
| Has2 | 271.07 | -2.89 | 0.35 | -8.21 | 2.17E-16 | 2.33E-14 |
| Olfr1033 | 3661.24 | -2.92 | 0.23 | -12.56 | 3.46E-36 | 2.14E-33 |
| Adora2b | 107.67 | -2.92 | 0.40 | -7.33 | 2.38E-13 | 1.82E-11 |
| Mx1 | 279.47 | -2.93 | 0.80 | -3.66 | 2.54E-04 | 4.00E-03 |
| Fjx1 | 104.27 | -2.94 | 0.52 | -5.66 | 1.52E-08 | 5.93E-07 |
| Car13 | 133.80 | -2.95 | 0.38 | -7.72 | 1.12E-14 | 1.01E-12 |
| Ifi205 | 2309.32 | -2.96 | 0.58 | -5.09 | 3.64E-07 | 1.10E-05 |
| Cxcl11 | 64.40 | -2.96 | 0.64 | -4.62 | 3.86E-06 | 9.63E-05 |
| Nptx1 | 23.52 | -2.97 | 0.83 | -3.56 | 3.71E-04 | 5.53E-03 |
| Tgif2 | 219.76 | -2.97 | 0.47 | -6.37 | 1.83E-10 | 9.50E-09 |
| Arc | 810.75 | -2.99 | 0.25 | -11.78 | 5.23E-32 | 2.29E-29 |
| Mmp13 | 607.11 | -3.00 | 0.37 | -8.10 | 5.67E-16 | 5.80E-14 |
| Bcl2a1d | 93.87 | -3.02 | 0.48 | -6.31 | 2.80E-10 | 1.42E-08 |
| Batf | 123.74 | -3.02 | 0.40 | -7.60 | 3.06E-14 | 2.62E-12 |
| Bcl3 | 1281.70 | -3.03 | 0.29 | -10.49 | 9.26E-26 | 2.56E-23 |
| Tnf | 1389.17 | -3.03 | 0.72 | -4.23 | 2.29E-05 | 4.81E-04 |
| Slc2a6 | 147.05 | -3.06 | 0.47 | -6.44 | 1.19E-10 | 6.34E-09 |
| Ifit1 | 2680.58 | -3.09 | 0.59 | -5.19 | 2.10E-07 | 6.60E-06 |
| Hcar2 | 34.22 | -3.10 | 0.83 | -3.74 | 1.82E-04 | 3.01E-03 |
| Thbs1 | 13182.74 | -3.11 | 0.29 | -10.90 | 1.17E-27 | 3.62E-25 |
| Sbno2 | 5602.25 | -3.12 | 0.51 | -6.08 | 1.18E-09 | 5.40E-08 |
| Gadd45b | 3835.45 | -3.13 | 0.26 | -11.85 | 2.10E-32 | 9.86E-30 |
| Fam83a | 24.31 | -3.14 | 0.95 | -3.32 | 9.12E-04 | 1.19E-02 |
| Nrip3 | 22.79 | -3.15 | 0.76 | -4.13 | 3.69E-05 | 7.31E-04 |
| Gm13889 | 1248.15 | -3.15 | 0.33 | -9.58 | 9.30E-22 | 1.54E-19 |
| Pfkfb3 | 4469.70 | -3.16 | 0.20 | -15.82 | 2.39E-56 | 5.41E-53 |
| Cyp1b1 | 1126.86 | -3.16 | 0.30 | -10.63 | 2.09E-26 | 6.19E-24 |
| Ereg | 49.94 | -3.18 | 0.59 | -5.40 | 6.84E-08 | 2.35E-06 |
| Gprc5a | 144.96 | -3.18 | 0.52 | -6.07 | 1.29E-09 | 5.86E-08 |
| Csrnp1 | 3618.52 | -3.18 | 0.27 | -11.68 | 1.59E-31 | 6.38E-29 |
| Il11 | 32.16 | -3.21 | 0.80 | -4.02 | 5.84E-05 | 1.10E-03 |
| Ripk3 | 401.24 | -3.23 | 0.87 | -3.72 | 1.99E-04 | 3.28E-03 |
| Clec4d | 93.03 | -3.24 | 0.53 | -6.11 | 1.02E-09 | 4.72E-08 |
| Hspa1a | 20241.84 | -3.26 | 0.81 | -4.04 | 5.45E-05 | 1.04E-03 |
| Hspa1b | 17929.57 | -3.26 | 0.92 | -3.55 | 3.84E-04 | 5.69E-03 |
| Gm49378 | 21.37 | -3.27 | 1.10 | -2.97 | 2.95E-03 | 3.10E-02 |
| Ccl7 | 2435.30 | -3.29 | 0.59 | -5.58 | 2.42E-08 | 9.15E-07 |
| Samsn1 | 145.33 | -3.30 | 0.45 | -7.36 | 1.80E-13 | 1.40E-11 |
| Tiparp | 3376.61 | -3.30 | 0.30 | -11.15 | 7.54E-29 | 2.44E-26 |
| Sphk1 | 336.98 | -3.31 | 0.77 | -4.31 | 1.65E-05 | 3.58E-04 |
| Serpina3f | 195.73 | -3.35 | 0.48 | -6.98 | 3.03E-12 | 1.97E-10 |
| Acp5 | 89.20 | -3.36 | 0.45 | -7.52 | 5.55E-14 | 4.52E-12 |
| Gm12250 | 163.23 | -3.37 | 0.46 | -7.30 | 2.82E-13 | 2.12E-11 |
| Gm8818 | 16.22 | -3.41 | 1.07 | -3.17 | 1.52E-03 | 1.82E-02 |
| Nod2 | 840.02 | -3.42 | 0.23 | -14.86 | 6.44E-50 | 1.10E-46 |
| Stk36 | 34.91 | -3.43 | 0.59 | -5.84 | 5.09E-09 | 2.12E-07 |
| Loxl4 | 213.52 | -3.45 | 0.49 | -7.00 | 2.48E-12 | 1.64E-10 |
| Rnd1 | 5005.15 | -3.46 | 0.64 | -5.41 | 6.26E-08 | 2.17E-06 |
| Neurl3 | 4240.04 | -3.47 | 0.65 | -5.31 | 1.12E-07 | 3.69E-06 |
| Oasl1 | 936.49 | -3.47 | 0.31 | -11.29 | 1.51E-29 | 5.28E-27 |
| Fpr2 | 40.05 | -3.48 | 0.83 | -4.21 | 2.57E-05 | 5.31E-04 |
| Cxcl10 | 3485.10 | -3.48 | 0.82 | -4.24 | 2.21E-05 | 4.68E-04 |
| Ccl3 | 952.98 | -3.51 | 0.34 | -10.18 | 2.33E-24 | 5.37E-22 |
| Prr7 | 210.43 | -3.54 | 0.37 | -9.49 | 2.32E-21 | 3.67E-19 |
| Ccl22 | 47.23 | -3.55 | 0.73 | -4.87 | 1.12E-06 | 3.10E-05 |
| Tnfaip3 | 7815.14 | -3.55 | 0.64 | -5.54 | 2.95E-08 | 1.09E-06 |
| Ikzf4 | 259.05 | -3.56 | 0.81 | -4.41 | 1.04E-05 | 2.36E-04 |
| Ptgs2 | 2466.02 | -3.57 | 0.24 | -14.87 | 5.33E-50 | 1.04E-46 |
| Cxcl2 | 3766.85 | -3.58 | 0.32 | -11.34 | 8.31E-30 | 2.97E-27 |
| Clec4e | 201.65 | -3.63 | 0.32 | -11.20 | 3.97E-29 | 1.35E-26 |
| Procr | 325.20 | -3.67 | 0.37 | -10.02 | 1.30E-23 | 2.71E-21 |
| Sgms2 | 207.06 | -3.68 | 0.47 | -7.83 | 4.69E-15 | 4.34E-13 |
| Bcl2a1a | 41.70 | -3.70 | 0.66 | -5.58 | 2.45E-08 | 9.25E-07 |
| Timp1 | 512.89 | -3.77 | 0.41 | -9.25 | 2.35E-20 | 3.37E-18 |
| Tnfaip6 | 1216.38 | -3.77 | 0.27 | -14.09 | 4.26E-45 | 4.14E-42 |
| Cxcl1 | 9827.68 | -3.78 | 0.26 | -14.59 | 3.36E-48 | 4.57E-45 |
| Bdkrb2 | 142.35 | -3.80 | 0.40 | -9.41 | 5.05E-21 | 7.55E-19 |
| Olr1 | 171.36 | -3.82 | 0.42 | -9.09 | 1.02E-19 | 1.45E-17 |
| Trim30c | 62.55 | -3.88 | 0.62 | -6.25 | 4.11E-10 | 2.02E-08 |
| Lipg | 98.38 | -3.89 | 0.66 | -5.88 | 4.17E-09 | 1.76E-07 |
| Olfr961 | 23.15 | -3.89 | 0.93 | -4.19 | 2.83E-05 | 5.79E-04 |
| Il1a | 314.24 | -3.90 | 0.41 | -9.59 | 9.13E-22 | 1.53E-19 |
| Icam1 | 23418.22 | -3.91 | 0.57 | -6.82 | 8.88E-12 | 5.44E-10 |
| Vgf | 15.19 | -3.96 | 1.21 | -3.29 | 1.01E-03 | 1.29E-02 |
| Ccl2 | 5754.16 | -4.07 | 0.31 | -13.32 | 1.87E-40 | 1.59E-37 |
| Plaur | 2089.53 | -4.07 | 0.64 | -6.39 | 1.71E-10 | 8.93E-09 |
| Stx11 | 1131.27 | -4.10 | 0.57 | -7.16 | 7.89E-13 | 5.56E-11 |
| Areg | 46.23 | -4.12 | 0.70 | -5.86 | 4.61E-09 | 1.93E-07 |
| Ccl4 | 1204.39 | -4.15 | 0.32 | -13.11 | 2.92E-39 | 2.34E-36 |
| Sprr1a | 24.33 | -4.22 | 0.82 | -5.13 | 2.95E-07 | 9.07E-06 |
| Il10 | 26.49 | -4.24 | 0.89 | -4.75 | 2.07E-06 | 5.45E-05 |
| Serpinb2 | 33.83 | -4.27 | 0.95 | -4.51 | 6.34E-06 | 1.52E-04 |
| Egr4 | 66.48 | -4.33 | 0.57 | -7.66 | 1.89E-14 | 1.67E-12 |
| Gm5796 | 24.68 | -4.40 | 1.27 | -3.47 | 5.23E-04 | 7.44E-03 |
| Edn2 | 28.40 | -4.41 | 1.02 | -4.33 | 1.47E-05 | 3.24E-04 |
| Pcdh10 | 16.30 | -4.44 | 1.01 | -4.39 | 1.12E-05 | 2.53E-04 |
| Gm8752 | 23.54 | -4.45 | 1.11 | -4.00 | 6.25E-05 | 1.17E-03 |
| Cxcl5 | 734.12 | -4.53 | 0.87 | -5.22 | 1.80E-07 | 5.72E-06 |
| Selp | 2632.62 | -4.55 | 0.26 | -17.30 | 4.95E-67 | 2.25E-63 |
| Cyp1a1 | 1556.61 | -4.57 | 1.14 | -4.02 | 5.76E-05 | 1.09E-03 |
| Calhm6 | 51.56 | -4.68 | 0.86 | -5.45 | 5.10E-08 | 1.80E-06 |
| Acod1 | 311.44 | -4.74 | 0.81 | -5.87 | 4.26E-09 | 1.79E-07 |
| Il6 | 3330.75 | -4.76 | 0.53 | -9.01 | 2.14E-19 | 2.91E-17 |
| Lif | 512.78 | -4.81 | 0.34 | -14.20 | 9.69E-46 | 1.01E-42 |
| Il1rn | 506.42 | -4.83 | 0.33 | -14.65 | 1.30E-48 | 1.96E-45 |
| S100a8 | 21.75 | -4.84 | 1.75 | -2.76 | 5.74E-03 | 5.26E-02 |
| Sele | 6058.28 | -4.86 | 0.26 | -18.50 | 1.92E-76 | 2.62E-72 |
| Csf2 | 89.48 | -4.88 | 0.75 | -6.52 | 6.98E-11 | 3.86E-09 |
| Ccl5 | 282.32 | -4.92 | 0.39 | -12.53 | 5.07E-36 | 3.00E-33 |
| Sh2d5 | 93.66 | -4.99 | 0.71 | -7.01 | 2.37E-12 | 1.57E-10 |
| Csf3 | 222.38 | -5.04 | 0.44 | -11.35 | 7.58E-30 | 2.79E-27 |
| A730049H05Rik | 56.28 | -5.08 | 0.78 | -6.53 | 6.49E-11 | 3.61E-09 |
| Nipal1 | 10.98 | -5.13 | 1.70 | -3.03 | 2.48E-03 | 2.70E-02 |
| Mcoln2 | 163.25 | -5.16 | 0.64 | -8.11 | 5.25E-16 | 5.41E-14 |
| Foxj1 | 32.95 | -5.17 | 0.91 | -5.65 | 1.57E-08 | 6.07E-07 |
| Fgf23 | 49.83 | -5.37 | 0.86 | -6.23 | 4.68E-10 | 2.26E-08 |
| Cxcl3 | 117.35 | -5.39 | 1.11 | -4.87 | 1.11E-06 | 3.07E-05 |
| Adamts4 | 6764.22 | -5.39 | 0.53 | -10.12 | 4.69E-24 | 9.98E-22 |
| Ptx3 | 5910.66 | -5.42 | 0.30 | -17.90 | 1.27E-71 | 8.61E-68 |
| Il12b | 12.55 | -5.56 | 1.68 | -3.30 | 9.61E-04 | 1.24E-02 |
| Ifnb1 | 65.44 | -5.72 | 0.74 | -7.71 | 1.23E-14 | 1.10E-12 |
| Mab21l3 | 14.33 | -5.92 | 1.62 | -3.66 | 2.48E-04 | 3.91E-03 |
| Prss46 | 20.69 | -6.01 | 1.28 | -4.71 | 2.53E-06 | 6.52E-05 |
| Acat3 | 9.74 | -6.07 | 1.88 | -3.22 | 1.27E-03 | 1.57E-02 |
| Gm10240 | 30.09 | -6.42 | 1.65 | -3.90 | 9.55E-05 | 1.70E-03 |
| Gm13370 | 30.09 | -6.42 | 1.65 | -3.90 | 9.55E-05 | 1.70E-03 |
| Kctd20 | 10.26 | -6.75 | 1.77 | -3.82 | 1.31E-04 | 2.27E-03 |
| Alas2 | 15.84 | -6.92 | 1.96 | -3.52 | 4.28E-04 | 6.25E-03 |
| Cbx3-ps6 | 25.01 | -8.33 | 1.80 | -4.63 | 3.70E-06 | 9.26E-05 |
| Pgam1-ps2 | 22.31 | -8.65 | 1.62 | -5.33 | 9.69E-08 | 3.22E-06 |
| Gm50241 | 76.90 | -20.52 | 4.79 | -4.29 | 1.82E-05 | 3.92E-04 |

LV8h vs LV0h DEGs

|  | baseMean | log2FoldChange | lfcSE | stat | pvalue | padj |
| --- | --- | --- | --- | --- | --- | --- |
| Gm11518 | 100.88 | 23.98 | 4.79 | 5.01 | 5.41E-07 | 1.93E-05 |
| Gm5537 | 156.08 | 23.62 | 4.79 | 4.94 | 7.97E-07 | 2.78E-05 |
| Gm28661 | 37791.50 | 8.27 | 2.25 | 3.68 | 2.37E-04 | 4.97E-03 |
| Rpl7-ps9 | 9.43 | 7.18 | 1.86 | 3.87 | 1.10E-04 | 2.56E-03 |
| Nfam1 | 7.07 | 6.76 | 1.99 | 3.40 | 6.69E-04 | 1.20E-02 |
| Ttll1 | 31.31 | 5.19 | 1.38 | 3.77 | 1.63E-04 | 3.61E-03 |
| Gm10359 | 3681.05 | 4.48 | 0.19 | 23.55 | 1.22E-122 | 8.51E-119 |
| Gm12671 | 3681.05 | 4.48 | 0.19 | 23.55 | 1.22E-122 | 8.51E-119 |
| 1700030C10Rik | 31.50 | 4.18 | 1.12 | 3.73 | 1.93E-04 | 4.18E-03 |
| Gm21293 | 13.83 | 4.01 | 1.28 | 3.13 | 1.76E-03 | 2.69E-02 |
| Lrrc70 | 23.02 | 3.07 | 0.81 | 3.78 | 1.55E-04 | 3.46E-03 |
| Gpr34 | 31.61 | 3.01 | 0.74 | 4.04 | 5.39E-05 | 1.35E-03 |
| Zfp407 | 13.55 | 2.99 | 1.02 | 2.94 | 3.27E-03 | 4.42E-02 |
| Dbp | 1139.57 | 2.73 | 0.18 | 15.04 | 4.08E-51 | 4.76E-48 |
| Gm2274 | 33.66 | 2.57 | 0.79 | 3.26 | 1.11E-03 | 1.84E-02 |
| Per3 | 568.76 | 2.34 | 0.23 | 10.00 | 1.49E-23 | 3.11E-21 |
| Irf4 | 43.37 | 2.25 | 0.74 | 3.03 | 2.47E-03 | 3.56E-02 |
| Ddit4l | 32.39 | 2.19 | 0.76 | 2.87 | 4.05E-03 | 5.24E-02 |
| Gpr183 | 29.27 | 2.13 | 0.77 | 2.78 | 5.42E-03 | 6.61E-02 |
| Slc22a17 | 60.06 | 1.98 | 0.54 | 3.68 | 2.34E-04 | 4.92E-03 |
| Sertad4 | 84.30 | 1.88 | 0.44 | 4.27 | 1.93E-05 | 5.46E-04 |
| Gpr160 | 39.88 | 1.85 | 0.60 | 3.09 | 2.02E-03 | 3.00E-02 |
| Gstt2 | 187.23 | 1.85 | 0.30 | 6.10 | 1.07E-09 | 5.55E-08 |
| Gm20431 | 91.98 | 1.72 | 0.64 | 2.67 | 7.53E-03 | 8.51E-02 |
| Kctd12b | 287.60 | 1.65 | 0.37 | 4.51 | 6.44E-06 | 1.98E-04 |
| Gm29013 | 1006.06 | 1.65 | 0.61 | 2.69 | 7.16E-03 | 8.24E-02 |
| Hpgd | 118.50 | 1.62 | 0.41 | 3.97 | 7.18E-05 | 1.74E-03 |
| Gm42688 | 65.27 | 1.61 | 0.47 | 3.46 | 5.43E-04 | 1.01E-02 |
| Per2 | 510.23 | 1.59 | 0.34 | 4.71 | 2.49E-06 | 8.23E-05 |
| Clec14a | 352.75 | 1.55 | 0.34 | 4.50 | 6.81E-06 | 2.09E-04 |
| Egfl7 | 658.47 | 1.52 | 0.24 | 6.21 | 5.27E-10 | 2.81E-08 |
| Pik3cg | 44.70 | 1.51 | 0.56 | 2.71 | 6.72E-03 | 7.87E-02 |
| Glyctk | 50.89 | 1.50 | 0.49 | 3.07 | 2.13E-03 | 3.13E-02 |
| Zfp760 | 49.19 | 1.46 | 0.55 | 2.65 | 7.94E-03 | 8.84E-02 |
| Msc | 61.19 | 1.42 | 0.55 | 2.61 | 9.11E-03 | 9.75E-02 |
| Hhex | 53.29 | 1.42 | 0.50 | 2.85 | 4.30E-03 | 5.51E-02 |
| Myct1 | 370.54 | 1.41 | 0.29 | 4.94 | 7.85E-07 | 2.75E-05 |
| Ush1c | 176.72 | 1.38 | 0.29 | 4.83 | 1.40E-06 | 4.71E-05 |
| Ddc | 72.56 | 1.38 | 0.46 | 3.02 | 2.52E-03 | 3.59E-02 |
| Olfr78 | 45.30 | 1.36 | 0.49 | 2.79 | 5.26E-03 | 6.46E-02 |
| Meox2 | 360.20 | 1.34 | 0.30 | 4.41 | 1.03E-05 | 3.04E-04 |
| Ahr | 198.52 | 1.33 | 0.29 | 4.57 | 4.80E-06 | 1.53E-04 |
| Ky | 351.60 | 1.33 | 0.37 | 3.61 | 3.01E-04 | 6.11E-03 |
| Lepr | 120.06 | 1.31 | 0.38 | 3.45 | 5.55E-04 | 1.02E-02 |
| Carmil3 | 101.45 | 1.30 | 0.37 | 3.55 | 3.85E-04 | 7.48E-03 |
| Sema6c | 161.93 | 1.29 | 0.31 | 4.10 | 4.07E-05 | 1.06E-03 |
| Hey1 | 206.25 | 1.28 | 0.37 | 3.43 | 6.09E-04 | 1.11E-02 |
| Lhx6 | 110.69 | 1.25 | 0.48 | 2.61 | 9.18E-03 | 9.79E-02 |
| Ciart | 146.27 | 1.25 | 0.33 | 3.83 | 1.30E-04 | 2.99E-03 |
| Nr1d1 | 3183.16 | 1.25 | 0.16 | 7.91 | 2.65E-15 | 2.59E-13 |
| Tef | 1543.13 | 1.23 | 0.19 | 6.43 | 1.27E-10 | 7.24E-09 |
| Rassf9 | 219.72 | 1.22 | 0.28 | 4.34 | 1.41E-05 | 4.07E-04 |
| Sv2a | 66.99 | 1.22 | 0.42 | 2.89 | 3.87E-03 | 5.05E-02 |
| Hic1 | 267.78 | 1.20 | 0.38 | 3.15 | 1.64E-03 | 2.56E-02 |
| Efnb3 | 1490.68 | 1.20 | 0.28 | 4.26 | 2.04E-05 | 5.74E-04 |
| Cog3 | 62.37 | 1.19 | 0.43 | 2.79 | 5.34E-03 | 6.54E-02 |
| Thsd1 | 154.56 | 1.18 | 0.36 | 3.28 | 1.05E-03 | 1.76E-02 |
| Engase | 116.47 | 1.18 | 0.34 | 3.51 | 4.41E-04 | 8.47E-03 |
| Inka1 | 64.67 | 1.18 | 0.45 | 2.64 | 8.33E-03 | 9.13E-02 |
| Nr1d2 | 2892.61 | 1.17 | 0.16 | 7.31 | 2.57E-13 | 1.95E-11 |
| Gm47985 | 64.05 | 1.17 | 0.43 | 2.70 | 6.97E-03 | 8.08E-02 |
| Spaar | 113.15 | 1.14 | 0.36 | 3.21 | 1.33E-03 | 2.14E-02 |
| Naa60 | 60.99 | 1.14 | 0.44 | 2.60 | 9.41E-03 | 9.92E-02 |
| Zfp395 | 317.31 | 1.14 | 0.36 | 3.14 | 1.66E-03 | 2.58E-02 |
| Anpep | 316.62 | 1.14 | 0.25 | 4.52 | 6.15E-06 | 1.90E-04 |
| Cxcr4 | 126.67 | 1.13 | 0.40 | 2.83 | 4.62E-03 | 5.81E-02 |
| Ide | 2963.76 | 1.12 | 0.16 | 6.89 | 5.59E-12 | 3.64E-10 |
| A430033K04Rik | 77.12 | 1.11 | 0.41 | 2.73 | 6.36E-03 | 7.49E-02 |
| Alox12 | 105.09 | 1.11 | 0.38 | 2.92 | 3.52E-03 | 4.69E-02 |
| Flt3l | 165.08 | 1.11 | 0.29 | 3.80 | 1.45E-04 | 3.27E-03 |
| Dynlt1c | 158.28 | 1.11 | 0.30 | 3.71 | 2.10E-04 | 4.52E-03 |
| Nr2f2 | 263.83 | 1.11 | 0.28 | 3.92 | 9.00E-05 | 2.14E-03 |
| Stk38 | 95.09 | 1.10 | 0.41 | 2.72 | 6.57E-03 | 7.73E-02 |
| Hlf | 492.94 | 1.10 | 0.22 | 4.90 | 9.65E-07 | 3.34E-05 |
| Aqp7 | 454.03 | 1.09 | 0.31 | 3.48 | 4.95E-04 | 9.32E-03 |
| Tchp | 196.50 | 1.08 | 0.26 | 4.12 | 3.84E-05 | 1.01E-03 |
| Nr4a2 | 534.28 | 1.06 | 0.27 | 3.89 | 9.92E-05 | 2.34E-03 |
| Lamb1 | 228.58 | 1.06 | 0.35 | 3.01 | 2.62E-03 | 3.71E-02 |
| Clcn1 | 298.76 | 1.05 | 0.24 | 4.42 | 9.72E-06 | 2.89E-04 |
| Prr29 | 87.84 | 1.05 | 0.38 | 2.73 | 6.25E-03 | 7.39E-02 |
| Tnfrsf25 | 106.43 | 1.04 | 0.39 | 2.68 | 7.38E-03 | 8.41E-02 |
| Calcoco1 | 332.20 | 1.03 | 0.39 | 2.66 | 7.75E-03 | 8.68E-02 |
| Heyl | 558.14 | 1.03 | 0.29 | 3.58 | 3.47E-04 | 6.91E-03 |
| Slc27a1 | 5019.64 | 1.02 | 0.14 | 7.12 | 1.09E-12 | 7.68E-11 |
| Dipk2b | 174.47 | 1.02 | 0.32 | 3.21 | 1.33E-03 | 2.14E-02 |
| Nynrin | 139.57 | 1.02 | 0.32 | 3.20 | 1.38E-03 | 2.21E-02 |
| G0s2 | 796.37 | 1.01 | 0.32 | 3.13 | 1.74E-03 | 2.67E-02 |
| Arrdc1 | 104.55 | 1.00 | 0.38 | 2.64 | 8.27E-03 | 9.10E-02 |
| Mmp28 | 115.37 | 0.98 | 0.33 | 2.95 | 3.22E-03 | 4.36E-02 |
| Pcdh18 | 120.08 | 0.98 | 0.33 | 2.98 | 2.86E-03 | 3.96E-02 |
| Wnk4 | 502.30 | 0.98 | 0.27 | 3.69 | 2.23E-04 | 4.74E-03 |
| Dpysl4 | 201.89 | 0.98 | 0.31 | 3.14 | 1.71E-03 | 2.64E-02 |
| Otud1 | 3931.37 | 0.98 | 0.27 | 3.64 | 2.71E-04 | 5.60E-03 |
| Ccdc8 | 133.09 | 0.97 | 0.36 | 2.69 | 7.08E-03 | 8.16E-02 |
| Car11 | 95.21 | 0.97 | 0.36 | 2.68 | 7.46E-03 | 8.46E-02 |
| Tppp | 857.36 | 0.97 | 0.24 | 4.03 | 5.63E-05 | 1.39E-03 |
| Pdp2 | 783.43 | 0.96 | 0.29 | 3.35 | 8.05E-04 | 1.41E-02 |
| Slc6a6 | 3219.37 | 0.96 | 0.25 | 3.76 | 1.71E-04 | 3.76E-03 |
| Pqlc3 | 101.46 | 0.95 | 0.35 | 2.70 | 6.91E-03 | 8.02E-02 |
| D630003M21Rik | 244.02 | 0.95 | 0.33 | 2.86 | 4.20E-03 | 5.42E-02 |
| Gpatch3 | 98.77 | 0.95 | 0.35 | 2.70 | 6.85E-03 | 7.98E-02 |
| Zfp507 | 325.51 | 0.95 | 0.35 | 2.67 | 7.49E-03 | 8.49E-02 |
| Klhl33 | 776.25 | 0.95 | 0.25 | 3.83 | 1.27E-04 | 2.91E-03 |
| Dixdc1 | 220.69 | 0.94 | 0.35 | 2.65 | 8.00E-03 | 8.87E-02 |
| Rufy2 | 182.38 | 0.94 | 0.30 | 3.16 | 1.58E-03 | 2.48E-02 |
| Adamts7 | 795.17 | 0.93 | 0.17 | 5.38 | 7.35E-08 | 2.97E-06 |
| Ajuba | 167.62 | 0.92 | 0.28 | 3.26 | 1.10E-03 | 1.83E-02 |
| Acad10 | 218.03 | 0.92 | 0.29 | 3.13 | 1.75E-03 | 2.68E-02 |
| Kdm6a | 572.96 | 0.92 | 0.31 | 2.98 | 2.89E-03 | 4.00E-02 |
| Cacna1g | 559.86 | 0.92 | 0.31 | 2.95 | 3.20E-03 | 4.35E-02 |
| Ints6l | 444.18 | 0.91 | 0.25 | 3.62 | 2.99E-04 | 6.10E-03 |
| Pm20d1 | 313.70 | 0.91 | 0.35 | 2.61 | 9.11E-03 | 9.75E-02 |
| Snx30 | 211.06 | 0.90 | 0.26 | 3.45 | 5.53E-04 | 1.02E-02 |
| Fbxl12 | 287.83 | 0.90 | 0.25 | 3.58 | 3.43E-04 | 6.85E-03 |
| Scrn1 | 254.81 | 0.90 | 0.26 | 3.41 | 6.45E-04 | 1.17E-02 |
| Hmgcs2 | 386.45 | 0.89 | 0.26 | 3.49 | 4.81E-04 | 9.08E-03 |
| Thap3 | 196.27 | 0.89 | 0.27 | 3.32 | 9.05E-04 | 1.56E-02 |
| Crocc | 369.07 | 0.89 | 0.30 | 2.92 | 3.52E-03 | 4.69E-02 |
| Podn | 425.27 | 0.88 | 0.21 | 4.18 | 2.94E-05 | 7.97E-04 |
| Stard8 | 1148.45 | 0.87 | 0.19 | 4.71 | 2.53E-06 | 8.31E-05 |
| Sox4 | 725.49 | 0.87 | 0.27 | 3.27 | 1.09E-03 | 1.81E-02 |
| Celsr2 | 218.46 | 0.87 | 0.28 | 3.07 | 2.11E-03 | 3.11E-02 |
| Sorcs2 | 432.74 | 0.87 | 0.24 | 3.58 | 3.48E-04 | 6.92E-03 |
| Pld4 | 178.20 | 0.86 | 0.29 | 3.00 | 2.74E-03 | 3.83E-02 |
| Hspa12b | 972.96 | 0.86 | 0.22 | 4.02 | 5.92E-05 | 1.46E-03 |
| Prickle1 | 399.41 | 0.86 | 0.21 | 4.05 | 5.19E-05 | 1.32E-03 |
| Tcap | 27533.19 | 0.86 | 0.13 | 6.73 | 1.76E-11 | 1.10E-09 |
| Pde7a | 1425.62 | 0.86 | 0.26 | 3.36 | 7.90E-04 | 1.39E-02 |
| Dact1 | 204.99 | 0.85 | 0.28 | 3.07 | 2.14E-03 | 3.13E-02 |
| Dhtkd1 | 305.41 | 0.85 | 0.26 | 3.26 | 1.10E-03 | 1.83E-02 |
| Madd | 320.21 | 0.85 | 0.22 | 3.93 | 8.63E-05 | 2.06E-03 |
| Rnf207 | 2218.76 | 0.85 | 0.17 | 4.92 | 8.78E-07 | 3.06E-05 |
| Smtnl2 | 257.66 | 0.85 | 0.28 | 3.00 | 2.72E-03 | 3.80E-02 |
| Trib2 | 599.99 | 0.84 | 0.25 | 3.35 | 8.09E-04 | 1.42E-02 |
| Art5 | 402.87 | 0.84 | 0.26 | 3.29 | 1.01E-03 | 1.71E-02 |
| Plscr4 | 290.64 | 0.84 | 0.24 | 3.57 | 3.59E-04 | 7.09E-03 |
| Cavin2 | 3748.14 | 0.84 | 0.22 | 3.82 | 1.36E-04 | 3.10E-03 |
| Adprm | 155.88 | 0.83 | 0.32 | 2.63 | 8.43E-03 | 9.20E-02 |
| St8sia4 | 348.06 | 0.82 | 0.30 | 2.70 | 6.88E-03 | 7.99E-02 |
| Brat1 | 210.73 | 0.81 | 0.27 | 2.97 | 2.95E-03 | 4.06E-02 |
| Aldh9a1 | 1272.66 | 0.81 | 0.21 | 3.77 | 1.67E-04 | 3.67E-03 |
| Afdn | 2277.67 | 0.81 | 0.24 | 3.40 | 6.65E-04 | 1.20E-02 |
| Lgals4 | 1405.24 | 0.81 | 0.24 | 3.31 | 9.27E-04 | 1.59E-02 |
| Pfas | 578.05 | 0.80 | 0.21 | 3.77 | 1.65E-04 | 3.65E-03 |
| Prdm15 | 195.52 | 0.80 | 0.28 | 2.85 | 4.32E-03 | 5.52E-02 |
| Fbxo10 | 348.93 | 0.80 | 0.24 | 3.38 | 7.17E-04 | 1.28E-02 |
| Plekhh3 | 407.55 | 0.78 | 0.28 | 2.73 | 6.34E-03 | 7.47E-02 |
| Dll4 | 995.06 | 0.77 | 0.30 | 2.60 | 9.24E-03 | 9.81E-02 |
| Thy1 | 422.12 | 0.77 | 0.25 | 3.05 | 2.29E-03 | 3.33E-02 |
| Dvl2 | 155.34 | 0.77 | 0.28 | 2.75 | 5.94E-03 | 7.11E-02 |
| Plxnb1 | 1806.89 | 0.77 | 0.20 | 3.78 | 1.57E-04 | 3.49E-03 |
| Zfp292 | 616.68 | 0.75 | 0.23 | 3.30 | 9.67E-04 | 1.64E-02 |
| Abhd18 | 1572.72 | 0.75 | 0.23 | 3.21 | 1.32E-03 | 2.13E-02 |
| Fam214a | 953.64 | 0.75 | 0.19 | 3.96 | 7.56E-05 | 1.83E-03 |
| S100pbp | 289.80 | 0.75 | 0.24 | 3.18 | 1.45E-03 | 2.30E-02 |
| Dgke | 670.63 | 0.75 | 0.23 | 3.21 | 1.33E-03 | 2.14E-02 |
| Carns1 | 1152.18 | 0.75 | 0.15 | 4.85 | 1.25E-06 | 4.29E-05 |
| Fam57b | 201.96 | 0.75 | 0.26 | 2.90 | 3.71E-03 | 4.88E-02 |
| Klhl22 | 303.96 | 0.75 | 0.28 | 2.68 | 7.29E-03 | 8.36E-02 |
| Uvssa | 316.11 | 0.74 | 0.26 | 2.84 | 4.55E-03 | 5.75E-02 |
| Mdm4 | 1351.37 | 0.73 | 0.20 | 3.60 | 3.24E-04 | 6.51E-03 |
| Dlg3 | 178.19 | 0.73 | 0.26 | 2.78 | 5.49E-03 | 6.70E-02 |
| Gpcpd1 | 6772.25 | 0.72 | 0.25 | 2.91 | 3.57E-03 | 4.73E-02 |
| Ston1 | 220.16 | 0.72 | 0.25 | 2.85 | 4.41E-03 | 5.61E-02 |
| Krba1 | 952.79 | 0.72 | 0.17 | 4.21 | 2.52E-05 | 6.94E-04 |
| Spice1 | 214.07 | 0.72 | 0.25 | 2.84 | 4.50E-03 | 5.69E-02 |
| Cnnm3 | 406.06 | 0.72 | 0.25 | 2.85 | 4.33E-03 | 5.53E-02 |
| Mknk1 | 537.56 | 0.71 | 0.22 | 3.21 | 1.32E-03 | 2.13E-02 |
| Dpysl2 | 367.64 | 0.71 | 0.27 | 2.61 | 8.99E-03 | 9.68E-02 |
| Rps6kb2 | 269.44 | 0.71 | 0.24 | 2.91 | 3.60E-03 | 4.75E-02 |
| Slc25a42 | 1755.50 | 0.71 | 0.19 | 3.77 | 1.65E-04 | 3.65E-03 |
| Pcmtd2 | 1394.30 | 0.71 | 0.21 | 3.30 | 9.70E-04 | 1.64E-02 |
| Bicra | 411.84 | 0.71 | 0.24 | 2.91 | 3.58E-03 | 4.74E-02 |
| Atg14 | 310.26 | 0.70 | 0.23 | 3.02 | 2.50E-03 | 3.58E-02 |
| Soga1 | 892.90 | -0.70 | 0.19 | -3.61 | 3.07E-04 | 6.21E-03 |
| Uck2 | 1718.21 | -0.70 | 0.24 | -2.92 | 3.46E-03 | 4.62E-02 |
| Zswim4 | 803.33 | -0.70 | 0.27 | -2.61 | 9.14E-03 | 9.77E-02 |
| Tbk1 | 791.92 | -0.70 | 0.19 | -3.62 | 2.93E-04 | 6.00E-03 |
| Tnip2 | 576.89 | -0.71 | 0.25 | -2.86 | 4.21E-03 | 5.42E-02 |
| Atf3 | 14101.49 | -0.71 | 0.22 | -3.27 | 1.07E-03 | 1.79E-02 |
| Ctps | 1636.20 | -0.71 | 0.20 | -3.48 | 4.93E-04 | 9.30E-03 |
| Birc2 | 990.53 | -0.71 | 0.19 | -3.80 | 1.45E-04 | 3.26E-03 |
| Bcar1 | 1285.20 | -0.71 | 0.23 | -3.09 | 1.99E-03 | 2.96E-02 |
| Lpin2 | 435.54 | -0.71 | 0.24 | -3.03 | 2.48E-03 | 3.56E-02 |
| Slc30a4 | 463.03 | -0.72 | 0.22 | -3.31 | 9.44E-04 | 1.61E-02 |
| S1pr1 | 2639.02 | -0.72 | 0.16 | -4.46 | 8.09E-06 | 2.45E-04 |
| Ankrd23 | 21220.36 | -0.72 | 0.15 | -4.68 | 2.92E-06 | 9.57E-05 |
| Yars | 187.67 | -0.72 | 0.28 | -2.61 | 9.01E-03 | 9.68E-02 |
| Sdcbp | 3342.64 | -0.72 | 0.17 | -4.14 | 3.48E-05 | 9.28E-04 |
| Parp12 | 900.43 | -0.72 | 0.17 | -4.33 | 1.49E-05 | 4.30E-04 |
| Nlgn2 | 525.75 | -0.73 | 0.20 | -3.69 | 2.21E-04 | 4.72E-03 |
| Picalm | 6962.61 | -0.73 | 0.19 | -3.82 | 1.36E-04 | 3.10E-03 |
| Itga5 | 3766.66 | -0.73 | 0.20 | -3.56 | 3.66E-04 | 7.19E-03 |
| Phlda1 | 2734.93 | -0.73 | 0.21 | -3.50 | 4.63E-04 | 8.81E-03 |
| Gnl3 | 886.89 | -0.73 | 0.23 | -3.15 | 1.61E-03 | 2.52E-02 |
| Nostrin | 287.08 | -0.73 | 0.26 | -2.85 | 4.34E-03 | 5.53E-02 |
| Svbp | 319.29 | -0.73 | 0.25 | -2.91 | 3.59E-03 | 4.75E-02 |
| Trim12a | 442.60 | -0.73 | 0.21 | -3.50 | 4.70E-04 | 8.92E-03 |
| Ckap4 | 1023.77 | -0.74 | 0.20 | -3.61 | 3.07E-04 | 6.21E-03 |
| Cp | 3448.00 | -0.74 | 0.20 | -3.64 | 2.74E-04 | 5.64E-03 |
| Pabpc1 | 3919.05 | -0.74 | 0.21 | -3.50 | 4.74E-04 | 8.96E-03 |
| Serpinh1 | 8902.41 | -0.74 | 0.22 | -3.34 | 8.45E-04 | 1.46E-02 |
| Jade2 | 534.66 | -0.74 | 0.18 | -4.04 | 5.41E-05 | 1.35E-03 |
| Cdr2 | 508.34 | -0.74 | 0.24 | -3.16 | 1.60E-03 | 2.51E-02 |
| Tbc1d9 | 277.82 | -0.74 | 0.27 | -2.73 | 6.31E-03 | 7.44E-02 |
| F2r | 1366.35 | -0.75 | 0.20 | -3.68 | 2.31E-04 | 4.86E-03 |
| Man1a | 1501.90 | -0.75 | 0.20 | -3.70 | 2.17E-04 | 4.64E-03 |
| Plxna2 | 2616.46 | -0.75 | 0.14 | -5.23 | 1.73E-07 | 6.66E-06 |
| Sav1 | 960.57 | -0.75 | 0.17 | -4.43 | 9.63E-06 | 2.87E-04 |
| Medag | 730.19 | -0.76 | 0.29 | -2.65 | 8.00E-03 | 8.87E-02 |
| Abl2 | 1183.00 | -0.76 | 0.19 | -4.01 | 5.96E-05 | 1.47E-03 |
| Cald1 | 2320.98 | -0.76 | 0.20 | -3.87 | 1.08E-04 | 2.53E-03 |
| Csf2ra | 370.60 | -0.76 | 0.21 | -3.60 | 3.17E-04 | 6.40E-03 |
| Fndc3a | 868.91 | -0.76 | 0.24 | -3.24 | 1.21E-03 | 1.99E-02 |
| Ehd1 | 4605.81 | -0.77 | 0.19 | -4.11 | 3.97E-05 | 1.04E-03 |
| Ssh1 | 662.91 | -0.77 | 0.26 | -2.94 | 3.26E-03 | 4.40E-02 |
| Cbx4 | 499.60 | -0.77 | 0.25 | -3.03 | 2.49E-03 | 3.56E-02 |
| Tmem106a | 202.10 | -0.77 | 0.27 | -2.89 | 3.84E-03 | 5.03E-02 |
| Dph5 | 192.99 | -0.77 | 0.28 | -2.77 | 5.53E-03 | 6.73E-02 |
| Ddx21 | 2071.00 | -0.77 | 0.18 | -4.23 | 2.34E-05 | 6.50E-04 |
| Syne3 | 292.85 | -0.77 | 0.24 | -3.19 | 1.40E-03 | 2.24E-02 |
| Cxcl14 | 608.63 | -0.77 | 0.22 | -3.55 | 3.88E-04 | 7.52E-03 |
| Stx6 | 660.71 | -0.77 | 0.19 | -4.07 | 4.66E-05 | 1.19E-03 |
| Efhd2 | 1004.91 | -0.78 | 0.25 | -3.09 | 2.00E-03 | 2.97E-02 |
| Fstl1 | 3634.61 | -0.78 | 0.19 | -4.06 | 4.94E-05 | 1.26E-03 |
| Mgat4a | 847.12 | -0.78 | 0.27 | -2.83 | 4.60E-03 | 5.80E-02 |
| Ppan | 317.85 | -0.78 | 0.29 | -2.65 | 8.08E-03 | 8.94E-02 |
| Rap1b | 3096.46 | -0.79 | 0.21 | -3.81 | 1.41E-04 | 3.20E-03 |
| St3gal1 | 1510.69 | -0.79 | 0.17 | -4.58 | 4.59E-06 | 1.48E-04 |
| Mapk4 | 223.86 | -0.79 | 0.27 | -2.98 | 2.88E-03 | 3.98E-02 |
| Itgbl1 | 387.26 | -0.79 | 0.26 | -3.08 | 2.10E-03 | 3.09E-02 |
| Ogfr | 1459.48 | -0.80 | 0.20 | -3.90 | 9.79E-05 | 2.32E-03 |
| Nrp2 | 2385.23 | -0.80 | 0.22 | -3.59 | 3.28E-04 | 6.57E-03 |
| Slit3 | 419.85 | -0.80 | 0.26 | -3.06 | 2.25E-03 | 3.27E-02 |
| Rnf31 | 1160.59 | -0.80 | 0.24 | -3.34 | 8.23E-04 | 1.43E-02 |
| Spty2d1 | 799.82 | -0.80 | 0.23 | -3.52 | 4.26E-04 | 8.23E-03 |
| Btg1 | 1973.34 | -0.80 | 0.25 | -3.20 | 1.35E-03 | 2.17E-02 |
| Clec2d | 677.37 | -0.81 | 0.23 | -3.46 | 5.38E-04 | 1.00E-02 |
| Metrnl | 579.49 | -0.81 | 0.30 | -2.68 | 7.45E-03 | 8.46E-02 |
| Ptpn12 | 1521.70 | -0.81 | 0.22 | -3.69 | 2.22E-04 | 4.72E-03 |
| Azin1 | 3923.31 | -0.81 | 0.22 | -3.77 | 1.66E-04 | 3.66E-03 |
| Cfp | 348.10 | -0.81 | 0.29 | -2.77 | 5.59E-03 | 6.78E-02 |
| Epb41l1 | 156.44 | -0.81 | 0.29 | -2.83 | 4.68E-03 | 5.88E-02 |
| Pmepa1 | 1019.67 | -0.81 | 0.20 | -3.98 | 6.76E-05 | 1.65E-03 |
| Relt | 145.98 | -0.81 | 0.31 | -2.64 | 8.33E-03 | 9.13E-02 |
| Plxnd1 | 5146.67 | -0.82 | 0.29 | -2.83 | 4.72E-03 | 5.91E-02 |
| Slc39a6 | 275.01 | -0.82 | 0.27 | -3.07 | 2.12E-03 | 3.11E-02 |
| Iffo2 | 368.85 | -0.82 | 0.26 | -3.17 | 1.52E-03 | 2.40E-02 |
| Myh7 | 3269.60 | -0.82 | 0.23 | -3.51 | 4.56E-04 | 8.73E-03 |
| Zfp703 | 792.69 | -0.82 | 0.25 | -3.31 | 9.37E-04 | 1.60E-02 |
| Lrrc8d | 360.88 | -0.82 | 0.22 | -3.68 | 2.33E-04 | 4.89E-03 |
| Tnfrsf1a | 1522.28 | -0.82 | 0.20 | -4.07 | 4.73E-05 | 1.21E-03 |
| Rps6ka3 | 1920.00 | -0.82 | 0.18 | -4.47 | 7.71E-06 | 2.34E-04 |
| Tulp1 | 262.22 | -0.82 | 0.25 | -3.34 | 8.51E-04 | 1.47E-02 |
| Swap70 | 1033.32 | -0.82 | 0.27 | -3.00 | 2.68E-03 | 3.76E-02 |
| Col5a2 | 1551.23 | -0.82 | 0.25 | -3.32 | 9.00E-04 | 1.55E-02 |
| Ifi35 | 595.10 | -0.82 | 0.23 | -3.56 | 3.68E-04 | 7.19E-03 |
| Manf | 1162.75 | -0.83 | 0.26 | -3.17 | 1.52E-03 | 2.40E-02 |
| Slc66a2 | 825.90 | -0.83 | 0.26 | -3.21 | 1.33E-03 | 2.14E-02 |
| Ednrb | 1255.62 | -0.83 | 0.27 | -3.04 | 2.36E-03 | 3.42E-02 |
| Zfp36l1 | 5757.86 | -0.83 | 0.23 | -3.62 | 2.97E-04 | 6.08E-03 |
| Cd302 | 278.16 | -0.83 | 0.28 | -2.96 | 3.07E-03 | 4.20E-02 |
| Taf7 | 449.38 | -0.83 | 0.25 | -3.35 | 8.22E-04 | 1.43E-02 |
| Ncf2 | 189.96 | -0.83 | 0.26 | -3.13 | 1.74E-03 | 2.67E-02 |
| Tank | 578.20 | -0.83 | 0.22 | -3.79 | 1.51E-04 | 3.39E-03 |
| Clmp | 210.59 | -0.83 | 0.30 | -2.76 | 5.72E-03 | 6.92E-02 |
| Ly6a | 2876.83 | -0.83 | 0.23 | -3.63 | 2.82E-04 | 5.80E-03 |
| Rhoj | 1423.43 | -0.84 | 0.22 | -3.82 | 1.34E-04 | 3.06E-03 |
| Rassf1 | 1362.56 | -0.84 | 0.31 | -2.69 | 7.19E-03 | 8.26E-02 |
| Pprc1 | 813.96 | -0.84 | 0.21 | -3.99 | 6.54E-05 | 1.60E-03 |
| Cd9 | 1079.36 | -0.84 | 0.25 | -3.37 | 7.39E-04 | 1.31E-02 |
| Acsl4 | 617.27 | -0.84 | 0.25 | -3.42 | 6.15E-04 | 1.12E-02 |
| P2ry6 | 108.80 | -0.84 | 0.32 | -2.60 | 9.24E-03 | 9.81E-02 |
| Cdh11 | 194.91 | -0.84 | 0.26 | -3.20 | 1.37E-03 | 2.19E-02 |
| Myo1e | 643.08 | -0.84 | 0.21 | -4.04 | 5.31E-05 | 1.34E-03 |
| Foxs1 | 118.02 | -0.84 | 0.32 | -2.60 | 9.35E-03 | 9.87E-02 |
| Ltbp2 | 183.18 | -0.84 | 0.31 | -2.75 | 5.93E-03 | 7.10E-02 |
| Postn | 1675.64 | -0.84 | 0.25 | -3.34 | 8.24E-04 | 1.43E-02 |
| Col4a2 | 15950.87 | -0.84 | 0.16 | -5.32 | 1.02E-07 | 4.02E-06 |
| Myh9 | 7977.30 | -0.84 | 0.17 | -4.90 | 9.62E-07 | 3.34E-05 |
| Gda | 718.12 | -0.84 | 0.27 | -3.12 | 1.83E-03 | 2.76E-02 |
| Mxd1 | 471.26 | -0.85 | 0.28 | -3.04 | 2.35E-03 | 3.41E-02 |
| Elf1 | 1028.21 | -0.85 | 0.22 | -3.86 | 1.15E-04 | 2.66E-03 |
| Bcl10 | 983.75 | -0.85 | 0.24 | -3.50 | 4.58E-04 | 8.74E-03 |
| Rhob | 9941.54 | -0.85 | 0.19 | -4.48 | 7.36E-06 | 2.24E-04 |
| Cars | 737.11 | -0.85 | 0.27 | -3.12 | 1.78E-03 | 2.71E-02 |
| Cachd1 | 171.91 | -0.85 | 0.32 | -2.68 | 7.40E-03 | 8.42E-02 |
| Stk40 | 2666.96 | -0.85 | 0.19 | -4.54 | 5.59E-06 | 1.76E-04 |
| H2ac19 | 436.65 | -0.86 | 0.29 | -2.91 | 3.62E-03 | 4.77E-02 |
| C3 | 6778.62 | -0.86 | 0.29 | -3.00 | 2.71E-03 | 3.80E-02 |
| Man2a1 | 1430.07 | -0.86 | 0.18 | -4.83 | 1.37E-06 | 4.64E-05 |
| Tmsb10 | 1147.19 | -0.86 | 0.22 | -3.94 | 8.31E-05 | 1.99E-03 |
| Pogk | 912.43 | -0.86 | 0.19 | -4.45 | 8.65E-06 | 2.59E-04 |
| Pdlim7 | 1813.57 | -0.86 | 0.21 | -4.04 | 5.40E-05 | 1.35E-03 |
| Slc16a6 | 261.54 | -0.87 | 0.24 | -3.55 | 3.91E-04 | 7.58E-03 |
| Id2 | 297.12 | -0.87 | 0.27 | -3.17 | 1.54E-03 | 2.42E-02 |
| Tm4sf1 | 2608.29 | -0.87 | 0.30 | -2.88 | 3.99E-03 | 5.19E-02 |
| Hivep1 | 1454.44 | -0.87 | 0.16 | -5.44 | 5.31E-08 | 2.17E-06 |
| B3gnt2 | 465.29 | -0.87 | 0.28 | -3.13 | 1.76E-03 | 2.69E-02 |
| Zc3h12c | 354.26 | -0.87 | 0.28 | -3.15 | 1.63E-03 | 2.55E-02 |
| S100a10 | 1377.10 | -0.87 | 0.25 | -3.48 | 4.97E-04 | 9.33E-03 |
| Adar | 921.46 | -0.87 | 0.26 | -3.35 | 8.05E-04 | 1.41E-02 |
| Dram1 | 279.61 | -0.88 | 0.28 | -3.14 | 1.70E-03 | 2.63E-02 |
| Cdk6 | 528.53 | -0.88 | 0.22 | -4.04 | 5.33E-05 | 1.34E-03 |
| Anxa1 | 1156.02 | -0.88 | 0.31 | -2.80 | 5.07E-03 | 6.28E-02 |
| Slc5a3 | 282.75 | -0.88 | 0.26 | -3.40 | 6.80E-04 | 1.22E-02 |
| D1Ertd622e | 185.64 | -0.88 | 0.34 | -2.61 | 9.15E-03 | 9.77E-02 |
| Arl13b | 1182.68 | -0.88 | 0.21 | -4.28 | 1.87E-05 | 5.30E-04 |
| Ampd2 | 397.16 | -0.88 | 0.26 | -3.37 | 7.48E-04 | 1.33E-02 |
| Spsb4 | 218.54 | -0.89 | 0.32 | -2.76 | 5.78E-03 | 6.97E-02 |
| Midn | 4694.19 | -0.89 | 0.25 | -3.56 | 3.67E-04 | 7.19E-03 |
| Crlf2 | 350.67 | -0.89 | 0.29 | -3.10 | 1.95E-03 | 2.91E-02 |
| Prkcd | 725.37 | -0.89 | 0.20 | -4.55 | 5.28E-06 | 1.67E-04 |
| Nhsl2 | 302.36 | -0.89 | 0.29 | -3.11 | 1.87E-03 | 2.81E-02 |
| Tgm2 | 10596.82 | -0.89 | 0.16 | -5.74 | 9.57E-09 | 4.44E-07 |
| Rap2b | 299.20 | -0.89 | 0.22 | -3.98 | 6.87E-05 | 1.68E-03 |
| Dcp2 | 746.23 | -0.90 | 0.29 | -3.09 | 1.99E-03 | 2.96E-02 |
| Heatr1 | 532.71 | -0.90 | 0.20 | -4.42 | 9.74E-06 | 2.89E-04 |
| Cry1 | 361.34 | -0.90 | 0.26 | -3.46 | 5.47E-04 | 1.01E-02 |
| Rasgef1b | 1271.90 | -0.90 | 0.21 | -4.26 | 2.04E-05 | 5.74E-04 |
| Klf7 | 1622.29 | -0.90 | 0.21 | -4.26 | 2.06E-05 | 5.79E-04 |
| Peli1 | 1125.60 | -0.91 | 0.22 | -4.05 | 5.09E-05 | 1.29E-03 |
| Trpv4 | 372.41 | -0.91 | 0.22 | -4.13 | 3.62E-05 | 9.61E-04 |
| Fam241a | 117.40 | -0.91 | 0.32 | -2.84 | 4.56E-03 | 5.75E-02 |
| Ptpn2 | 630.26 | -0.91 | 0.23 | -3.98 | 7.01E-05 | 1.71E-03 |
| Fcgr3 | 379.61 | -0.91 | 0.22 | -4.10 | 4.10E-05 | 1.06E-03 |
| Ankrd33b | 1796.84 | -0.92 | 0.21 | -4.33 | 1.46E-05 | 4.21E-04 |
| Ube2l6 | 253.37 | -0.92 | 0.25 | -3.70 | 2.16E-04 | 4.62E-03 |
| Fam43a | 1557.09 | -0.92 | 0.25 | -3.64 | 2.71E-04 | 5.59E-03 |
| Tlnrd1 | 905.82 | -0.92 | 0.26 | -3.53 | 4.12E-04 | 7.96E-03 |
| Slc45a3 | 162.04 | -0.93 | 0.29 | -3.14 | 1.70E-03 | 2.63E-02 |
| Rab20 | 813.50 | -0.93 | 0.22 | -4.19 | 2.80E-05 | 7.64E-04 |
| B4galt3 | 570.92 | -0.93 | 0.24 | -3.88 | 1.05E-04 | 2.47E-03 |
| Wsb1 | 1923.28 | -0.93 | 0.29 | -3.24 | 1.20E-03 | 1.97E-02 |
| Flnb | 4214.96 | -0.93 | 0.27 | -3.46 | 5.43E-04 | 1.01E-02 |
| Ccnl1 | 2794.60 | -0.94 | 0.26 | -3.57 | 3.61E-04 | 7.10E-03 |
| Marchf3 | 170.33 | -0.94 | 0.28 | -3.28 | 1.02E-03 | 1.72E-02 |
| Clcn5 | 132.07 | -0.94 | 0.36 | -2.60 | 9.27E-03 | 9.82E-02 |
| Loxl2 | 1264.22 | -0.94 | 0.21 | -4.50 | 6.94E-06 | 2.12E-04 |
| Marcks | 1758.92 | -0.94 | 0.17 | -5.47 | 4.43E-08 | 1.85E-06 |
| Elmsan1 | 1436.78 | -0.94 | 0.20 | -4.74 | 2.17E-06 | 7.18E-05 |
| Katna1 | 582.09 | -0.95 | 0.24 | -3.95 | 7.78E-05 | 1.88E-03 |
| Slc9a3r1 | 201.75 | -0.95 | 0.32 | -2.94 | 3.28E-03 | 4.42E-02 |
| Il10ra | 229.28 | -0.96 | 0.28 | -3.36 | 7.77E-04 | 1.37E-02 |
| Resf1 | 984.19 | -0.96 | 0.28 | -3.42 | 6.19E-04 | 1.12E-02 |
| Pirb | 311.66 | -0.96 | 0.32 | -2.96 | 3.09E-03 | 4.22E-02 |
| Rin3 | 789.22 | -0.96 | 0.23 | -4.17 | 3.02E-05 | 8.14E-04 |
| Map3k14 | 441.10 | -0.96 | 0.31 | -3.14 | 1.67E-03 | 2.59E-02 |
| 1810055G02Rik | 259.91 | -0.96 | 0.26 | -3.73 | 1.94E-04 | 4.19E-03 |
| Ramp3 | 244.90 | -0.96 | 0.31 | -3.08 | 2.10E-03 | 3.09E-02 |
| Cyth4 | 290.00 | -0.96 | 0.23 | -4.20 | 2.63E-05 | 7.21E-04 |
| Nol10 | 420.45 | -0.96 | 0.29 | -3.31 | 9.37E-04 | 1.60E-02 |
| Col27a1 | 393.78 | -0.96 | 0.27 | -3.51 | 4.43E-04 | 8.51E-03 |
| Atp10a | 131.17 | -0.97 | 0.33 | -2.90 | 3.73E-03 | 4.89E-02 |
| Atp2b4 | 1366.34 | -0.97 | 0.26 | -3.77 | 1.63E-04 | 3.61E-03 |
| Zfp954 | 205.46 | -0.97 | 0.33 | -2.94 | 3.25E-03 | 4.40E-02 |
| Tle3 | 778.73 | -0.97 | 0.17 | -5.57 | 2.52E-08 | 1.10E-06 |
| Epha2 | 929.44 | -0.97 | 0.25 | -3.88 | 1.05E-04 | 2.47E-03 |
| Crem | 562.12 | -0.97 | 0.36 | -2.71 | 6.81E-03 | 7.95E-02 |
| Tnfrsf23 | 122.41 | -0.98 | 0.37 | -2.66 | 7.72E-03 | 8.67E-02 |
| Ctla2a | 834.09 | -0.98 | 0.36 | -2.74 | 6.22E-03 | 7.37E-02 |
| Adamts1 | 6798.60 | -0.98 | 0.21 | -4.61 | 3.99E-06 | 1.29E-04 |
| Pgm2 | 424.57 | -0.99 | 0.38 | -2.60 | 9.32E-03 | 9.85E-02 |
| Fbn1 | 3920.39 | -0.99 | 0.18 | -5.37 | 8.04E-08 | 3.22E-06 |
| Clic4 | 17017.39 | -0.99 | 0.14 | -7.07 | 1.60E-12 | 1.11E-10 |
| Cnn3 | 1813.95 | -0.99 | 0.18 | -5.53 | 3.14E-08 | 1.34E-06 |
| Plekhg2 | 1568.33 | -0.99 | 0.26 | -3.86 | 1.12E-04 | 2.60E-03 |
| Ier3 | 4417.46 | -0.99 | 0.25 | -4.01 | 6.16E-05 | 1.51E-03 |
| Med11 | 166.33 | -1.00 | 0.32 | -3.16 | 1.60E-03 | 2.50E-02 |
| Mitd1 | 124.40 | -1.00 | 0.34 | -2.97 | 2.99E-03 | 4.10E-02 |
| Bcl2l11 | 782.86 | -1.00 | 0.19 | -5.32 | 1.01E-07 | 3.99E-06 |
| Themis2 | 131.65 | -1.01 | 0.38 | -2.65 | 8.08E-03 | 8.94E-02 |
| Trim56 | 1650.16 | -1.01 | 0.18 | -5.47 | 4.63E-08 | 1.92E-06 |
| Cd74 | 1513.08 | -1.01 | 0.25 | -4.04 | 5.36E-05 | 1.35E-03 |
| Plagl2 | 202.04 | -1.01 | 0.27 | -3.71 | 2.10E-04 | 4.52E-03 |
| Irf5 | 210.38 | -1.02 | 0.28 | -3.65 | 2.57E-04 | 5.35E-03 |
| Pxdc1 | 989.80 | -1.02 | 0.34 | -2.96 | 3.07E-03 | 4.20E-02 |
| Rhog | 598.96 | -1.02 | 0.23 | -4.38 | 1.21E-05 | 3.55E-04 |
| Foxc1 | 224.40 | -1.02 | 0.29 | -3.50 | 4.57E-04 | 8.74E-03 |
| Glipr2 | 168.06 | -1.02 | 0.28 | -3.62 | 3.00E-04 | 6.11E-03 |
| Col4a1 | 22465.25 | -1.03 | 0.17 | -5.97 | 2.32E-09 | 1.15E-07 |
| Lrrc32 | 1475.90 | -1.03 | 0.28 | -3.69 | 2.20E-04 | 4.70E-03 |
| 1500009L16Rik | 100.47 | -1.03 | 0.39 | -2.64 | 8.29E-03 | 9.10E-02 |
| Klhl25 | 271.88 | -1.03 | 0.24 | -4.29 | 1.81E-05 | 5.17E-04 |
| Kctd12 | 2064.82 | -1.03 | 0.23 | -4.46 | 8.07E-06 | 2.44E-04 |
| Fam49a | 354.28 | -1.03 | 0.24 | -4.37 | 1.22E-05 | 3.58E-04 |
| Msx1 | 224.49 | -1.03 | 0.37 | -2.79 | 5.23E-03 | 6.44E-02 |
| Pvr | 1736.32 | -1.04 | 0.25 | -4.22 | 2.48E-05 | 6.84E-04 |
| Tinagl1 | 2571.11 | -1.04 | 0.22 | -4.70 | 2.59E-06 | 8.48E-05 |
| Tnfrsf11a | 131.30 | -1.04 | 0.34 | -3.08 | 2.04E-03 | 3.02E-02 |
| Fam107b | 318.37 | -1.05 | 0.32 | -3.28 | 1.03E-03 | 1.73E-02 |
| Ncf1 | 266.37 | -1.05 | 0.34 | -3.08 | 2.09E-03 | 3.09E-02 |
| 9930111J21Rik1 | 121.87 | -1.05 | 0.38 | -2.74 | 6.10E-03 | 7.25E-02 |
| Prr5l | 82.72 | -1.05 | 0.38 | -2.76 | 5.71E-03 | 6.90E-02 |
| Fgl2 | 3691.36 | -1.05 | 0.26 | -4.04 | 5.41E-05 | 1.35E-03 |
| Lrrc8b | 317.17 | -1.05 | 0.30 | -3.56 | 3.76E-04 | 7.32E-03 |
| Bst2 | 654.63 | -1.06 | 0.19 | -5.70 | 1.22E-08 | 5.58E-07 |
| Steap4 | 1684.61 | -1.06 | 0.22 | -4.83 | 1.34E-06 | 4.56E-05 |
| Ugdh | 955.76 | -1.06 | 0.34 | -3.11 | 1.89E-03 | 2.84E-02 |
| Zfp36 | 14734.93 | -1.06 | 0.26 | -4.14 | 3.40E-05 | 9.10E-04 |
| Alpk1 | 270.70 | -1.07 | 0.29 | -3.68 | 2.29E-04 | 4.85E-03 |
| Prkd2 | 1582.82 | -1.07 | 0.28 | -3.87 | 1.07E-04 | 2.50E-03 |
| Rnf24 | 390.06 | -1.07 | 0.24 | -4.55 | 5.46E-06 | 1.73E-04 |
| Ptprc | 370.80 | -1.07 | 0.24 | -4.46 | 8.29E-06 | 2.49E-04 |
| Il1b | 842.71 | -1.07 | 0.34 | -3.13 | 1.72E-03 | 2.65E-02 |
| Kazn | 206.43 | -1.08 | 0.39 | -2.76 | 5.83E-03 | 7.01E-02 |
| Lgals9 | 913.00 | -1.08 | 0.18 | -6.13 | 8.81E-10 | 4.58E-08 |
| Cdc42ep4 | 906.31 | -1.08 | 0.31 | -3.44 | 5.88E-04 | 1.08E-02 |
| Piezo1 | 2207.38 | -1.09 | 0.19 | -5.58 | 2.34E-08 | 1.03E-06 |
| N4bp1 | 1331.76 | -1.09 | 0.16 | -6.78 | 1.23E-11 | 7.75E-10 |
| Dab2 | 2077.75 | -1.09 | 0.26 | -4.26 | 2.02E-05 | 5.71E-04 |
| Srgn | 1236.24 | -1.09 | 0.27 | -3.99 | 6.61E-05 | 1.62E-03 |
| Gm6548 | 170.86 | -1.09 | 0.30 | -3.65 | 2.58E-04 | 5.35E-03 |
| Mt1 | 6597.47 | -1.10 | 0.20 | -5.62 | 1.93E-08 | 8.59E-07 |
| Parp10 | 772.34 | -1.10 | 0.17 | -6.37 | 1.87E-10 | 1.04E-08 |
| B2m | 6658.47 | -1.10 | 0.16 | -7.07 | 1.57E-12 | 1.10E-10 |
| Hdc | 116.36 | -1.10 | 0.39 | -2.82 | 4.73E-03 | 5.92E-02 |
| Gm15542 | 297.03 | -1.10 | 0.36 | -3.10 | 1.94E-03 | 2.91E-02 |
| Sema4a | 192.34 | -1.10 | 0.35 | -3.14 | 1.71E-03 | 2.63E-02 |
| Sash1 | 2309.97 | -1.11 | 0.17 | -6.48 | 9.04E-11 | 5.21E-09 |
| Arsi | 66.04 | -1.11 | 0.42 | -2.63 | 8.57E-03 | 9.31E-02 |
| Zfp516 | 553.76 | -1.11 | 0.21 | -5.35 | 8.97E-08 | 3.56E-06 |
| Pak4 | 293.90 | -1.11 | 0.25 | -4.38 | 1.20E-05 | 3.52E-04 |
| Ccl6 | 447.77 | -1.12 | 0.37 | -2.99 | 2.80E-03 | 3.90E-02 |
| Mpp2 | 290.22 | -1.12 | 0.26 | -4.25 | 2.16E-05 | 6.01E-04 |
| Eef1e1 | 302.61 | -1.12 | 0.27 | -4.15 | 3.28E-05 | 8.80E-04 |
| Aacs | 235.87 | -1.12 | 0.27 | -4.13 | 3.57E-05 | 9.49E-04 |
| Pkhd1l1 | 123.83 | -1.12 | 0.33 | -3.35 | 8.03E-04 | 1.41E-02 |
| Ms4a6c | 204.77 | -1.12 | 0.28 | -4.07 | 4.79E-05 | 1.22E-03 |
| Ralb | 1126.87 | -1.12 | 0.19 | -5.81 | 6.39E-09 | 3.00E-07 |
| Timeless | 1054.32 | -1.13 | 0.24 | -4.66 | 3.19E-06 | 1.04E-04 |
| Gm27029 | 71.95 | -1.13 | 0.40 | -2.86 | 4.21E-03 | 5.42E-02 |
| Fam107a | 270.08 | -1.13 | 0.25 | -4.61 | 3.95E-06 | 1.28E-04 |
| Dse | 321.04 | -1.14 | 0.30 | -3.80 | 1.47E-04 | 3.30E-03 |
| Noct | 12592.45 | -1.14 | 0.18 | -6.17 | 6.90E-10 | 3.63E-08 |
| 9930111J21Rik2 | 773.45 | -1.14 | 0.25 | -4.57 | 4.87E-06 | 1.55E-04 |
| Tnfsf10 | 562.54 | -1.15 | 0.35 | -3.26 | 1.12E-03 | 1.85E-02 |
| H2-D1 | 9420.07 | -1.15 | 0.22 | -5.28 | 1.30E-07 | 5.00E-06 |
| Rell1 | 607.47 | -1.15 | 0.21 | -5.54 | 2.99E-08 | 1.28E-06 |
| Gna13 | 1865.88 | -1.15 | 0.18 | -6.34 | 2.23E-10 | 1.23E-08 |
| Lgals3bp | 1422.30 | -1.16 | 0.20 | -5.86 | 4.74E-09 | 2.27E-07 |
| Tox2 | 72.30 | -1.16 | 0.39 | -2.94 | 3.28E-03 | 4.42E-02 |
| Odc1 | 3923.45 | -1.16 | 0.23 | -5.08 | 3.72E-07 | 1.37E-05 |
| Cilp | 663.75 | -1.16 | 0.32 | -3.57 | 3.57E-04 | 7.07E-03 |
| Psmb8 | 290.87 | -1.16 | 0.24 | -4.85 | 1.21E-06 | 4.18E-05 |
| Fscn1 | 1480.02 | -1.16 | 0.22 | -5.20 | 2.04E-07 | 7.75E-06 |
| Trim16 | 366.22 | -1.16 | 0.32 | -3.69 | 2.25E-04 | 4.78E-03 |
| Gabpb1 | 519.57 | -1.17 | 0.22 | -5.38 | 7.39E-08 | 2.98E-06 |
| Rasd1 | 442.18 | -1.17 | 0.21 | -5.55 | 2.87E-08 | 1.24E-06 |
| Etv6 | 600.33 | -1.17 | 0.25 | -4.74 | 2.10E-06 | 7.00E-05 |
| Txnrd1 | 2922.73 | -1.17 | 0.23 | -5.02 | 5.25E-07 | 1.88E-05 |
| Ifitm2 | 1581.28 | -1.18 | 0.15 | -7.64 | 2.09E-14 | 1.84E-12 |
| Myo1g | 186.37 | -1.18 | 0.29 | -4.11 | 4.00E-05 | 1.04E-03 |
| Tnfrsf10b | 703.66 | -1.19 | 0.32 | -3.66 | 2.55E-04 | 5.32E-03 |
| Znfx1 | 2258.57 | -1.19 | 0.21 | -5.64 | 1.69E-08 | 7.60E-07 |
| Mthfd2 | 198.30 | -1.19 | 0.43 | -2.77 | 5.68E-03 | 6.87E-02 |
| Cdr2l | 261.71 | -1.19 | 0.32 | -3.69 | 2.24E-04 | 4.75E-03 |
| Adgrg6 | 72.61 | -1.20 | 0.43 | -2.77 | 5.54E-03 | 6.73E-02 |
| Snx10 | 861.93 | -1.20 | 0.20 | -6.06 | 1.39E-09 | 7.17E-08 |
| Psd4 | 61.13 | -1.20 | 0.46 | -2.61 | 9.13E-03 | 9.76E-02 |
| Rnf19b | 1465.23 | -1.20 | 0.21 | -5.62 | 1.89E-08 | 8.45E-07 |
| Lilr4b | 1094.36 | -1.21 | 0.29 | -4.13 | 3.71E-05 | 9.79E-04 |
| Bach1 | 2199.25 | -1.21 | 0.18 | -6.65 | 2.95E-11 | 1.79E-09 |
| Thbs4 | 168.27 | -1.21 | 0.40 | -3.05 | 2.28E-03 | 3.32E-02 |
| Tpm3 | 3608.31 | -1.21 | 0.29 | -4.13 | 3.66E-05 | 9.71E-04 |
| Gnai3 | 1475.84 | -1.22 | 0.20 | -6.19 | 6.00E-10 | 3.17E-08 |
| Rasip1 | 3460.42 | -1.23 | 0.26 | -4.79 | 1.68E-06 | 5.63E-05 |
| Fcer1g | 217.36 | -1.24 | 0.26 | -4.75 | 1.99E-06 | 6.63E-05 |
| Micall2 | 239.68 | -1.24 | 0.26 | -4.84 | 1.30E-06 | 4.43E-05 |
| Dusp5 | 1101.95 | -1.24 | 0.29 | -4.29 | 1.79E-05 | 5.13E-04 |
| Ugcg | 798.87 | -1.25 | 0.27 | -4.71 | 2.51E-06 | 8.26E-05 |
| Acta1 | 14945.66 | -1.25 | 0.28 | -4.53 | 5.83E-06 | 1.81E-04 |
| Cd52 | 71.61 | -1.25 | 0.41 | -3.05 | 2.33E-03 | 3.37E-02 |
| Neto2 | 48.13 | -1.26 | 0.48 | -2.61 | 8.96E-03 | 9.67E-02 |
| Junb | 14852.73 | -1.26 | 0.28 | -4.53 | 6.03E-06 | 1.87E-04 |
| Creb5 | 780.11 | -1.26 | 0.24 | -5.18 | 2.20E-07 | 8.34E-06 |
| Rab32 | 101.98 | -1.26 | 0.38 | -3.34 | 8.37E-04 | 1.45E-02 |
| Vcan | 818.00 | -1.26 | 0.18 | -6.95 | 3.55E-12 | 2.39E-10 |
| Dbn1 | 450.69 | -1.26 | 0.21 | -6.00 | 1.97E-09 | 9.88E-08 |
| Hcls1 | 453.66 | -1.27 | 0.22 | -5.73 | 9.99E-09 | 4.62E-07 |
| Parp9 | 898.69 | -1.27 | 0.24 | -5.28 | 1.27E-07 | 4.91E-06 |
| Fzd5 | 367.25 | -1.27 | 0.28 | -4.52 | 6.10E-06 | 1.89E-04 |
| Skil | 1573.70 | -1.27 | 0.18 | -7.06 | 1.72E-12 | 1.18E-10 |
| Irak3 | 384.81 | -1.27 | 0.25 | -5.04 | 4.78E-07 | 1.73E-05 |
| Lacc1 | 324.02 | -1.28 | 0.26 | -4.95 | 7.37E-07 | 2.60E-05 |
| Gramd1a | 2419.33 | -1.28 | 0.16 | -8.19 | 2.62E-16 | 2.84E-14 |
| Ptpn1 | 967.56 | -1.28 | 0.25 | -5.22 | 1.80E-07 | 6.89E-06 |
| Spred1 | 1238.95 | -1.28 | 0.20 | -6.51 | 7.53E-11 | 4.38E-09 |
| Ppp1r15a | 5085.85 | -1.29 | 0.22 | -5.91 | 3.33E-09 | 1.61E-07 |
| Slc2a3 | 127.48 | -1.29 | 0.41 | -3.17 | 1.54E-03 | 2.43E-02 |
| Ifi203 | 2435.79 | -1.29 | 0.23 | -5.50 | 3.73E-08 | 1.58E-06 |
| H3f3b | 7202.30 | -1.29 | 0.27 | -4.82 | 1.46E-06 | 4.92E-05 |
| Serpine1 | 7869.17 | -1.29 | 0.16 | -7.91 | 2.64E-15 | 2.59E-13 |
| Trib1 | 1800.73 | -1.30 | 0.23 | -5.62 | 1.87E-08 | 8.35E-07 |
| Cflar | 2724.86 | -1.30 | 0.18 | -7.04 | 1.92E-12 | 1.31E-10 |
| Aldh1a2 | 250.56 | -1.30 | 0.32 | -4.03 | 5.56E-05 | 1.38E-03 |
| Mlkl | 191.44 | -1.30 | 0.28 | -4.68 | 2.94E-06 | 9.58E-05 |
| Zmynd15 | 212.63 | -1.30 | 0.31 | -4.19 | 2.73E-05 | 7.48E-04 |
| Ldlr | 449.61 | -1.30 | 0.29 | -4.52 | 6.25E-06 | 1.92E-04 |
| Pakap | 10674.53 | -1.31 | 0.17 | -7.61 | 2.83E-14 | 2.42E-12 |
| Foxp4 | 753.50 | -1.31 | 0.23 | -5.80 | 6.75E-09 | 3.15E-07 |
| Rhou | 278.13 | -1.31 | 0.25 | -5.14 | 2.72E-07 | 1.02E-05 |
| Pawr | 109.86 | -1.31 | 0.35 | -3.78 | 1.54E-04 | 3.44E-03 |
| Vash1 | 560.80 | -1.32 | 0.27 | -4.95 | 7.26E-07 | 2.57E-05 |
| Ulbp1 | 146.61 | -1.32 | 0.47 | -2.79 | 5.35E-03 | 6.55E-02 |
| Lbp | 332.53 | -1.32 | 0.23 | -5.77 | 7.76E-09 | 3.61E-07 |
| Plekho2 | 1685.74 | -1.33 | 0.30 | -4.43 | 9.25E-06 | 2.76E-04 |
| Nek6 | 194.71 | -1.33 | 0.36 | -3.74 | 1.86E-04 | 4.06E-03 |
| Sp110 | 397.46 | -1.33 | 0.27 | -5.00 | 5.73E-07 | 2.04E-05 |
| Sh3bp2 | 198.93 | -1.33 | 0.39 | -3.41 | 6.52E-04 | 1.18E-02 |
| Hsp90aa1 | 13914.91 | -1.34 | 0.23 | -5.92 | 3.18E-09 | 1.55E-07 |
| Gm10160 | 157.91 | -1.34 | 0.45 | -2.97 | 2.97E-03 | 4.08E-02 |
| Kcne4 | 333.58 | -1.34 | 0.30 | -4.53 | 5.82E-06 | 1.81E-04 |
| Tgfb1 | 1467.84 | -1.34 | 0.21 | -6.39 | 1.62E-10 | 9.07E-09 |
| Ubtd2 | 65.45 | -1.34 | 0.43 | -3.12 | 1.79E-03 | 2.72E-02 |
| Ell2 | 874.07 | -1.34 | 0.20 | -6.74 | 1.61E-11 | 1.01E-09 |
| Xaf1 | 580.23 | -1.35 | 0.27 | -5.07 | 3.92E-07 | 1.44E-05 |
| Adgrd1 | 449.46 | -1.35 | 0.32 | -4.26 | 2.08E-05 | 5.82E-04 |
| Tent5c | 97.42 | -1.35 | 0.51 | -2.65 | 8.09E-03 | 8.94E-02 |
| Samd9l | 979.45 | -1.35 | 0.21 | -6.40 | 1.59E-10 | 8.97E-09 |
| Gdf15 | 218.19 | -1.35 | 0.36 | -3.73 | 1.90E-04 | 4.13E-03 |
| Zfp57 | 102.25 | -1.36 | 0.45 | -3.00 | 2.71E-03 | 3.80E-02 |
| Slc7a7 | 120.20 | -1.36 | 0.33 | -4.09 | 4.33E-05 | 1.11E-03 |
| Lox | 167.70 | -1.36 | 0.37 | -3.63 | 2.83E-04 | 5.80E-03 |
| Arc | 810.75 | -1.36 | 0.26 | -5.30 | 1.15E-07 | 4.52E-06 |
| Daxx | 98.92 | -1.36 | 0.38 | -3.59 | 3.32E-04 | 6.63E-03 |
| Mndal | 363.19 | -1.36 | 0.26 | -5.28 | 1.27E-07 | 4.92E-06 |
| Rela | 2553.97 | -1.36 | 0.25 | -5.37 | 7.92E-08 | 3.18E-06 |
| Cebpb | 3211.66 | -1.37 | 0.21 | -6.50 | 7.87E-11 | 4.55E-09 |
| Taf4b | 239.31 | -1.38 | 0.25 | -5.51 | 3.61E-08 | 1.53E-06 |
| Agpat4 | 256.61 | -1.38 | 0.39 | -3.57 | 3.60E-04 | 7.09E-03 |
| Creb3l1 | 241.92 | -1.38 | 0.28 | -4.95 | 7.49E-07 | 2.64E-05 |
| Pgf | 548.77 | -1.38 | 0.33 | -4.24 | 2.27E-05 | 6.31E-04 |
| Trim21 | 285.89 | -1.38 | 0.30 | -4.54 | 5.65E-06 | 1.77E-04 |
| Kcna5 | 1023.61 | -1.38 | 0.26 | -5.22 | 1.82E-07 | 6.93E-06 |
| Lcp1 | 1225.40 | -1.39 | 0.22 | -6.19 | 5.86E-10 | 3.11E-08 |
| Rab11fip1 | 70.40 | -1.39 | 0.46 | -3.00 | 2.72E-03 | 3.80E-02 |
| Ddx58 | 987.62 | -1.39 | 0.22 | -6.22 | 5.03E-10 | 2.70E-08 |
| Sema3f | 1303.50 | -1.40 | 0.20 | -6.82 | 8.97E-12 | 5.71E-10 |
| Snai1 | 95.94 | -1.40 | 0.42 | -3.35 | 8.10E-04 | 1.42E-02 |
| Slfn5 | 6283.02 | -1.40 | 0.18 | -7.62 | 2.51E-14 | 2.17E-12 |
| Zfp593 | 237.49 | -1.41 | 0.37 | -3.82 | 1.34E-04 | 3.06E-03 |
| Slc16a13 | 363.86 | -1.41 | 0.37 | -3.86 | 1.13E-04 | 2.62E-03 |
| Egfr | 531.89 | -1.41 | 0.27 | -5.24 | 1.60E-07 | 6.17E-06 |
| Inhba | 292.56 | -1.41 | 0.44 | -3.22 | 1.29E-03 | 2.11E-02 |
| Etv3 | 1787.72 | -1.42 | 0.15 | -9.45 | 3.35E-21 | 5.40E-19 |
| Igf2bp2 | 236.82 | -1.42 | 0.31 | -4.56 | 5.14E-06 | 1.63E-04 |
| Nfil3 | 1300.51 | -1.43 | 0.26 | -5.49 | 3.91E-08 | 1.64E-06 |
| Nfkb1 | 3050.20 | -1.43 | 0.18 | -7.89 | 2.90E-15 | 2.82E-13 |
| Rhoc | 3502.73 | -1.44 | 0.26 | -5.50 | 3.84E-08 | 1.62E-06 |
| Apaf1 | 590.50 | -1.45 | 0.19 | -7.50 | 6.63E-14 | 5.46E-12 |
| Ikzf1 | 110.92 | -1.45 | 0.36 | -3.97 | 7.10E-05 | 1.72E-03 |
| Fignl2 | 198.75 | -1.45 | 0.32 | -4.54 | 5.60E-06 | 1.76E-04 |
| Gpr132 | 119.73 | -1.45 | 0.49 | -2.95 | 3.21E-03 | 4.36E-02 |
| Uap1 | 2819.13 | -1.45 | 0.23 | -6.29 | 3.14E-10 | 1.72E-08 |
| Plat | 1320.84 | -1.46 | 0.24 | -6.03 | 1.68E-09 | 8.49E-08 |
| Clec4n | 128.40 | -1.47 | 0.47 | -3.12 | 1.83E-03 | 2.76E-02 |
| Aoah | 73.80 | -1.47 | 0.41 | -3.59 | 3.26E-04 | 6.54E-03 |
| Vav1 | 140.50 | -1.47 | 0.35 | -4.21 | 2.57E-05 | 7.08E-04 |
| Traf3ip2 | 161.13 | -1.47 | 0.42 | -3.46 | 5.35E-04 | 1.00E-02 |
| Trim30d | 169.68 | -1.47 | 0.30 | -4.83 | 1.34E-06 | 4.56E-05 |
| Ngfr | 40.77 | -1.47 | 0.56 | -2.62 | 8.83E-03 | 9.55E-02 |
| Plekha4 | 200.95 | -1.48 | 0.36 | -4.12 | 3.78E-05 | 9.95E-04 |
| Gvin1 | 2890.03 | -1.48 | 0.18 | -8.05 | 8.02E-16 | 8.32E-14 |
| Pde4b | 3854.32 | -1.48 | 0.25 | -5.82 | 5.94E-09 | 2.81E-07 |
| Syt12 | 163.79 | -1.48 | 0.29 | -5.04 | 4.60E-07 | 1.68E-05 |
| Pde12 | 827.53 | -1.49 | 0.30 | -5.03 | 4.99E-07 | 1.80E-05 |
| Ikbke | 131.46 | -1.49 | 0.32 | -4.65 | 3.40E-06 | 1.10E-04 |
| Irf9 | 1179.93 | -1.49 | 0.23 | -6.41 | 1.47E-10 | 8.34E-09 |
| Asb4 | 345.60 | -1.49 | 0.40 | -3.73 | 1.93E-04 | 4.18E-03 |
| Igsf6 | 123.74 | -1.50 | 0.36 | -4.10 | 4.13E-05 | 1.07E-03 |
| Tgif1 | 476.86 | -1.50 | 0.32 | -4.71 | 2.44E-06 | 8.07E-05 |
| Lonrf3 | 74.11 | -1.50 | 0.55 | -2.75 | 5.98E-03 | 7.15E-02 |
| Ppp1r18 | 1738.47 | -1.50 | 0.20 | -7.62 | 2.49E-14 | 2.17E-12 |
| Emp1 | 4833.34 | -1.50 | 0.54 | -2.79 | 5.34E-03 | 6.54E-02 |
| Crtc2 | 1431.03 | -1.52 | 0.19 | -7.88 | 3.18E-15 | 3.05E-13 |
| Rab8b | 882.98 | -1.52 | 0.24 | -6.37 | 1.94E-10 | 1.07E-08 |
| Ifrd1 | 5845.78 | -1.52 | 0.26 | -5.82 | 5.89E-09 | 2.79E-07 |
| Stat2 | 1452.31 | -1.53 | 0.19 | -7.83 | 4.82E-15 | 4.59E-13 |
| Col12a1 | 93.37 | -1.53 | 0.48 | -3.18 | 1.47E-03 | 2.33E-02 |
| Arrdc2 | 580.68 | -1.53 | 0.31 | -4.95 | 7.51E-07 | 2.64E-05 |
| Tsku | 164.50 | -1.54 | 0.34 | -4.46 | 8.13E-06 | 2.45E-04 |
| Nop58 | 1231.43 | -1.54 | 0.27 | -5.65 | 1.63E-08 | 7.35E-07 |
| Cd53 | 265.73 | -1.54 | 0.30 | -5.09 | 3.51E-07 | 1.30E-05 |
| Plscr1 | 841.16 | -1.54 | 0.22 | -6.89 | 5.47E-12 | 3.58E-10 |
| C5ar1 | 509.03 | -1.54 | 0.28 | -5.60 | 2.14E-08 | 9.47E-07 |
| Mmp14 | 814.11 | -1.55 | 0.21 | -7.20 | 6.01E-13 | 4.34E-11 |
| Fcgr1 | 76.95 | -1.55 | 0.52 | -2.97 | 2.98E-03 | 4.09E-02 |
| Tor3a | 612.24 | -1.55 | 0.24 | -6.40 | 1.51E-10 | 8.54E-09 |
| Alkal2 | 47.10 | -1.55 | 0.59 | -2.61 | 8.98E-03 | 9.67E-02 |
| Gbp7 | 1216.13 | -1.55 | 0.17 | -9.03 | 1.70E-19 | 2.33E-17 |
| Eif1a | 512.80 | -1.56 | 0.28 | -5.50 | 3.89E-08 | 1.64E-06 |
| Errfi1 | 3216.23 | -1.56 | 0.23 | -6.69 | 2.30E-11 | 1.41E-09 |
| Dnajb1 | 4948.41 | -1.57 | 0.22 | -6.99 | 2.82E-12 | 1.91E-10 |
| Oas2 | 764.62 | -1.57 | 0.22 | -7.25 | 4.28E-13 | 3.16E-11 |
| Spon2 | 219.64 | -1.57 | 0.44 | -3.56 | 3.67E-04 | 7.19E-03 |
| Ift122 | 670.64 | -1.58 | 0.33 | -4.78 | 1.75E-06 | 5.86E-05 |
| Gcc1 | 761.69 | -1.58 | 0.31 | -5.08 | 3.70E-07 | 1.36E-05 |
| Gm21596 | 94.86 | -1.59 | 0.51 | -3.12 | 1.79E-03 | 2.72E-02 |
| Ifitm3 | 3027.76 | -1.59 | 0.21 | -7.57 | 3.73E-14 | 3.17E-12 |
| Slamf7 | 121.23 | -1.59 | 0.44 | -3.66 | 2.55E-04 | 5.32E-03 |
| Mapk6 | 1813.56 | -1.59 | 0.17 | -9.30 | 1.47E-20 | 2.25E-18 |
| Trim25 | 1801.99 | -1.60 | 0.22 | -7.19 | 6.30E-13 | 4.50E-11 |
| Oas1b | 215.58 | -1.60 | 0.32 | -4.99 | 6.08E-07 | 2.16E-05 |
| Pml | 699.48 | -1.60 | 0.22 | -7.32 | 2.57E-13 | 1.95E-11 |
| H2-K1 | 2317.54 | -1.60 | 0.20 | -7.98 | 1.43E-15 | 1.44E-13 |
| Ripk1 | 981.78 | -1.60 | 0.32 | -5.04 | 4.67E-07 | 1.69E-05 |
| Dnaja1 | 6435.66 | -1.61 | 0.21 | -7.72 | 1.17E-14 | 1.05E-12 |
| Slc25a25 | 1507.56 | -1.61 | 0.33 | -4.88 | 1.07E-06 | 3.69E-05 |
| Hsph1 | 5986.94 | -1.61 | 0.20 | -8.03 | 9.93E-16 | 1.02E-13 |
| Bcat1 | 45.79 | -1.61 | 0.57 | -2.84 | 4.50E-03 | 5.69E-02 |
| Gas7 | 689.26 | -1.61 | 0.22 | -7.25 | 4.11E-13 | 3.05E-11 |
| Fosl2 | 6752.69 | -1.62 | 0.24 | -6.61 | 3.94E-11 | 2.37E-09 |
| Jak2 | 2170.22 | -1.62 | 0.23 | -7.13 | 1.01E-12 | 7.17E-11 |
| Izumo1 | 56.93 | -1.62 | 0.50 | -3.27 | 1.07E-03 | 1.79E-02 |
| Eda2r | 242.97 | -1.62 | 0.37 | -4.44 | 8.86E-06 | 2.65E-04 |
| Slc1a1 | 284.31 | -1.63 | 0.25 | -6.60 | 4.00E-11 | 2.39E-09 |
| Avpr1a | 53.78 | -1.63 | 0.58 | -2.83 | 4.66E-03 | 5.85E-02 |
| Gbp9 | 1664.42 | -1.63 | 0.19 | -8.59 | 8.58E-18 | 1.05E-15 |
| Tnfrsf1b | 765.25 | -1.63 | 0.22 | -7.49 | 7.11E-14 | 5.76E-12 |
| Cd86 | 250.65 | -1.63 | 0.30 | -5.49 | 3.97E-08 | 1.66E-06 |
| Snx20 | 74.57 | -1.64 | 0.50 | -3.30 | 9.51E-04 | 1.62E-02 |
| Adamts9 | 2395.26 | -1.64 | 0.23 | -7.06 | 1.72E-12 | 1.18E-10 |
| Baz1a | 653.17 | -1.64 | 0.20 | -8.30 | 1.03E-16 | 1.16E-14 |
| Prnd | 193.15 | -1.65 | 0.53 | -3.09 | 1.99E-03 | 2.97E-02 |
| Basp1 | 123.33 | -1.65 | 0.37 | -4.41 | 1.01E-05 | 3.00E-04 |
| Rdh10 | 403.95 | -1.65 | 0.31 | -5.34 | 9.52E-08 | 3.76E-06 |
| Dtx3l | 1005.01 | -1.66 | 0.24 | -6.91 | 4.78E-12 | 3.17E-10 |
| Apobec3 | 512.30 | -1.66 | 0.25 | -6.53 | 6.64E-11 | 3.89E-09 |
| Ptprj | 660.81 | -1.66 | 0.24 | -6.83 | 8.73E-12 | 5.61E-10 |
| Enc1 | 993.99 | -1.67 | 0.23 | -7.22 | 5.36E-13 | 3.89E-11 |
| Gm38431 | 57.53 | -1.68 | 0.60 | -2.81 | 4.90E-03 | 6.09E-02 |
| Sp140 | 180.19 | -1.68 | 0.31 | -5.45 | 5.11E-08 | 2.09E-06 |
| Adm | 576.58 | -1.68 | 0.19 | -8.77 | 1.73E-18 | 2.27E-16 |
| Map3k8 | 113.58 | -1.68 | 0.37 | -4.57 | 4.86E-06 | 1.55E-04 |
| Map3k6 | 1305.06 | -1.68 | 0.23 | -7.22 | 5.04E-13 | 3.69E-11 |
| Psmb9 | 82.02 | -1.69 | 0.41 | -4.12 | 3.80E-05 | 9.97E-04 |
| Spi1 | 249.24 | -1.69 | 0.30 | -5.56 | 2.69E-08 | 1.16E-06 |
| Orai2 | 122.22 | -1.70 | 0.39 | -4.37 | 1.24E-05 | 3.62E-04 |
| Mob3c | 546.01 | -1.70 | 0.33 | -5.15 | 2.54E-07 | 9.58E-06 |
| Fcgr2b | 620.26 | -1.70 | 0.31 | -5.45 | 5.11E-08 | 2.09E-06 |
| Il1r1 | 1125.25 | -1.70 | 0.29 | -5.96 | 2.53E-09 | 1.25E-07 |
| Eif2ak2 | 833.00 | -1.71 | 0.26 | -6.70 | 2.14E-11 | 1.33E-09 |
| Meox1 | 668.35 | -1.71 | 0.32 | -5.42 | 5.85E-08 | 2.38E-06 |
| Stc1 | 411.08 | -1.73 | 0.28 | -6.14 | 8.06E-10 | 4.21E-08 |
| Eid3 | 39.82 | -1.75 | 0.64 | -2.72 | 6.57E-03 | 7.73E-02 |
| Nlrc5 | 417.83 | -1.76 | 0.26 | -6.70 | 2.11E-11 | 1.32E-09 |
| Ptges | 74.76 | -1.76 | 0.45 | -3.91 | 9.33E-05 | 2.21E-03 |
| Mmp9 | 37.36 | -1.76 | 0.65 | -2.70 | 6.98E-03 | 8.08E-02 |
| Mmp3 | 211.26 | -1.76 | 0.35 | -5.05 | 4.35E-07 | 1.59E-05 |
| Zfp46 | 2718.15 | -1.76 | 0.53 | -3.35 | 8.10E-04 | 1.42E-02 |
| Zc3hav1 | 1503.08 | -1.76 | 0.21 | -8.55 | 1.27E-17 | 1.52E-15 |
| Jdp2 | 585.90 | -1.76 | 0.21 | -8.26 | 1.42E-16 | 1.56E-14 |
| Akap12 | 2816.25 | -1.77 | 0.23 | -7.68 | 1.63E-14 | 1.44E-12 |
| Nfkbiz | 4582.44 | -1.77 | 0.62 | -2.83 | 4.69E-03 | 5.88E-02 |
| Plau | 906.24 | -1.77 | 0.66 | -2.68 | 7.30E-03 | 8.36E-02 |
| Pou2f2 | 146.89 | -1.77 | 0.32 | -5.61 | 2.08E-08 | 9.21E-07 |
| Msr1 | 257.27 | -1.77 | 0.34 | -5.29 | 1.23E-07 | 4.80E-06 |
| Rgs1 | 85.72 | -1.77 | 0.47 | -3.74 | 1.84E-04 | 4.02E-03 |
| Icosl | 785.51 | -1.78 | 0.30 | -6.03 | 1.59E-09 | 8.10E-08 |
| Exoc3l4 | 201.50 | -1.78 | 0.33 | -5.47 | 4.51E-08 | 1.87E-06 |
| Slc7a5 | 377.16 | -1.78 | 0.41 | -4.36 | 1.29E-05 | 3.75E-04 |
| Tapbp | 121.76 | -1.79 | 0.43 | -4.16 | 3.13E-05 | 8.43E-04 |
| Rel | 1536.61 | -1.79 | 0.23 | -7.77 | 7.90E-15 | 7.27E-13 |
| Npas2 | 172.78 | -1.79 | 0.51 | -3.50 | 4.63E-04 | 8.82E-03 |
| Rtn4rl2 | 159.47 | -1.79 | 0.35 | -5.14 | 2.80E-07 | 1.05E-05 |
| Gch1 | 722.86 | -1.80 | 0.25 | -7.29 | 3.02E-13 | 2.26E-11 |
| Rhbdf2 | 1415.19 | -1.81 | 0.19 | -9.75 | 1.80E-22 | 3.32E-20 |
| Ptafr | 334.48 | -1.81 | 0.36 | -5.04 | 4.61E-07 | 1.68E-05 |
| Ncf4 | 75.00 | -1.81 | 0.44 | -4.16 | 3.18E-05 | 8.55E-04 |
| Stk36 | 34.91 | -1.81 | 0.63 | -2.90 | 3.72E-03 | 4.89E-02 |
| Peg10 | 96.54 | -1.82 | 0.43 | -4.18 | 2.86E-05 | 7.77E-04 |
| Slc7a2 | 390.72 | -1.82 | 0.36 | -5.10 | 3.32E-07 | 1.24E-05 |
| Phf11d | 602.66 | -1.83 | 0.24 | -7.55 | 4.50E-14 | 3.78E-12 |
| Grrp1 | 339.46 | -1.83 | 0.42 | -4.33 | 1.50E-05 | 4.32E-04 |
| Gbp10 | 99.27 | -1.83 | 0.48 | -3.80 | 1.44E-04 | 3.24E-03 |
| Hdx | 65.57 | -1.83 | 0.45 | -4.04 | 5.46E-05 | 1.36E-03 |
| Cybb | 1049.66 | -1.83 | 0.31 | -5.99 | 2.14E-09 | 1.07E-07 |
| Gprc5a | 144.96 | -1.83 | 0.53 | -3.46 | 5.38E-04 | 1.00E-02 |
| Gm614 | 46.22 | -1.83 | 0.66 | -2.77 | 5.65E-03 | 6.85E-02 |
| Hilpda | 282.26 | -1.84 | 0.46 | -4.01 | 5.95E-05 | 1.47E-03 |
| Gbp6 | 3267.94 | -1.84 | 0.18 | -10.09 | 6.16E-24 | 1.33E-21 |
| Il2rg | 1000.79 | -1.84 | 0.27 | -6.82 | 8.94E-12 | 5.71E-10 |
| Kdm6b | 5461.91 | -1.85 | 0.25 | -7.41 | 1.27E-13 | 1.01E-11 |
| Sntb2 | 1485.48 | -1.85 | 0.27 | -6.84 | 8.14E-12 | 5.25E-10 |
| Mmp12 | 60.33 | -1.85 | 0.53 | -3.48 | 5.01E-04 | 9.39E-03 |
| Rasgef1a | 28.55 | -1.86 | 0.66 | -2.82 | 4.82E-03 | 6.01E-02 |
| H2-Q7 | 2704.83 | -1.86 | 0.19 | -9.76 | 1.66E-22 | 3.11E-20 |
| Irgm2 | 1514.95 | -1.86 | 0.26 | -7.07 | 1.55E-12 | 1.09E-10 |
| Hmox1 | 1517.58 | -1.87 | 0.57 | -3.27 | 1.08E-03 | 1.79E-02 |
| Milr1 | 30.27 | -1.88 | 0.65 | -2.90 | 3.76E-03 | 4.93E-02 |
| Rnf213 | 4453.84 | -1.88 | 0.20 | -9.49 | 2.27E-21 | 3.74E-19 |
| Nuak2 | 533.10 | -1.89 | 0.30 | -6.37 | 1.85E-10 | 1.03E-08 |
| Gm5431 | 54.74 | -1.89 | 0.56 | -3.37 | 7.60E-04 | 1.34E-02 |
| Tnf | 1389.17 | -1.90 | 0.72 | -2.65 | 8.13E-03 | 8.97E-02 |
| Gadd45g | 3765.76 | -1.90 | 0.20 | -9.64 | 5.41E-22 | 9.59E-20 |
| Cd80 | 40.83 | -1.91 | 0.59 | -3.24 | 1.21E-03 | 1.99E-02 |
| Trim30a | 839.03 | -1.92 | 0.26 | -7.51 | 5.91E-14 | 4.89E-12 |
| Elovl6 | 81.08 | -1.92 | 0.42 | -4.58 | 4.64E-06 | 1.49E-04 |
| Gm3636 | 57.14 | -1.93 | 0.60 | -3.21 | 1.31E-03 | 2.12E-02 |
| Irak2 | 876.26 | -1.93 | 0.25 | -7.82 | 5.41E-15 | 5.08E-13 |
| Ifi208 | 149.28 | -1.93 | 0.36 | -5.36 | 8.19E-08 | 3.27E-06 |
| Cx3cl1 | 729.68 | -1.93 | 0.26 | -7.49 | 7.02E-14 | 5.71E-12 |
| Wfdc17 | 205.38 | -1.93 | 0.35 | -5.57 | 2.53E-08 | 1.10E-06 |
| Spdl1 | 26.45 | -1.94 | 0.73 | -2.64 | 8.29E-03 | 9.10E-02 |
| Cd44 | 727.60 | -1.94 | 0.25 | -7.83 | 4.86E-15 | 4.60E-13 |
| Cfap69 | 120.16 | -1.95 | 0.57 | -3.44 | 5.78E-04 | 1.06E-02 |
| Rnf122 | 449.95 | -1.95 | 0.35 | -5.57 | 2.50E-08 | 1.09E-06 |
| Kif1a | 43.82 | -1.95 | 0.53 | -3.68 | 2.30E-04 | 4.86E-03 |
| Il33 | 284.84 | -1.95 | 0.75 | -2.61 | 9.01E-03 | 9.68E-02 |
| Tnfaip8l1 | 165.04 | -1.96 | 0.35 | -5.54 | 3.03E-08 | 1.30E-06 |
| Zfp729a | 1923.86 | -1.97 | 0.19 | -10.34 | 4.84E-25 | 1.17E-22 |
| Phf11b | 62.84 | -1.98 | 0.49 | -4.04 | 5.24E-05 | 1.32E-03 |
| Tiparp | 3376.61 | -1.98 | 0.30 | -6.66 | 2.77E-11 | 1.69E-09 |
| Ccrl2 | 962.20 | -1.98 | 0.25 | -7.93 | 2.21E-15 | 2.20E-13 |
| Arrdc4 | 1401.01 | -1.98 | 0.23 | -8.56 | 1.10E-17 | 1.32E-15 |
| Asns | 219.97 | -1.98 | 0.48 | -4.15 | 3.39E-05 | 9.07E-04 |
| Sema7a | 3017.30 | -1.99 | 0.16 | -12.23 | 2.09E-34 | 1.17E-31 |
| Cxcl16 | 1101.43 | -1.99 | 0.24 | -8.46 | 2.78E-17 | 3.27E-15 |
| Tpbg | 57.47 | -1.99 | 0.56 | -3.56 | 3.76E-04 | 7.32E-03 |
| Fyb | 222.96 | -2.00 | 0.46 | -4.31 | 1.62E-05 | 4.66E-04 |
| Gbp4 | 1657.44 | -2.01 | 0.23 | -8.60 | 7.85E-18 | 9.82E-16 |
| Dennd4a | 1010.07 | -2.01 | 0.19 | -10.32 | 5.62E-25 | 1.33E-22 |
| Nrip3 | 22.79 | -2.01 | 0.77 | -2.60 | 9.30E-03 | 9.85E-02 |
| Pilra | 43.59 | -2.01 | 0.56 | -3.61 | 3.05E-04 | 6.19E-03 |
| Myd88 | 1125.03 | -2.01 | 0.26 | -7.72 | 1.12E-14 | 1.02E-12 |
| Ifi44 | 300.53 | -2.02 | 0.29 | -6.93 | 4.19E-12 | 2.79E-10 |
| Ptpre | 736.80 | -2.02 | 0.28 | -7.31 | 2.74E-13 | 2.06E-11 |
| Myc | 1523.25 | -2.02 | 0.26 | -7.61 | 2.65E-14 | 2.28E-12 |
| Gm4070 | 2174.60 | -2.02 | 0.23 | -8.60 | 7.74E-18 | 9.77E-16 |
| Sh3pxd2b | 673.47 | -2.02 | 0.19 | -10.66 | 1.60E-26 | 4.66E-24 |
| Adam8 | 61.56 | -2.02 | 0.58 | -3.52 | 4.32E-04 | 8.34E-03 |
| Arntl | 479.45 | -2.03 | 0.27 | -7.55 | 4.43E-14 | 3.74E-12 |
| Ccno | 55.31 | -2.03 | 0.64 | -3.16 | 1.59E-03 | 2.49E-02 |
| Pou3f1 | 29.83 | -2.03 | 0.64 | -3.16 | 1.55E-03 | 2.44E-02 |
| Bmp2 | 192.57 | -2.04 | 0.33 | -6.17 | 7.04E-10 | 3.69E-08 |
| Ada | 45.44 | -2.06 | 0.63 | -3.26 | 1.13E-03 | 1.86E-02 |
| Ms4a6d | 172.77 | -2.06 | 0.31 | -6.65 | 2.95E-11 | 1.79E-09 |
| H2-Q4 | 4183.97 | -2.07 | 0.23 | -9.17 | 4.57E-20 | 6.60E-18 |
| Itpkc | 703.51 | -2.07 | 0.28 | -7.37 | 1.76E-13 | 1.36E-11 |
| Saa3 | 434.75 | -2.08 | 0.27 | -7.79 | 6.47E-15 | 6.04E-13 |
| Lcn2 | 161.05 | -2.08 | 0.32 | -6.53 | 6.64E-11 | 3.89E-09 |
| Csf2rb | 176.14 | -2.08 | 0.34 | -6.08 | 1.20E-09 | 6.22E-08 |
| Ccl9 | 708.24 | -2.08 | 0.23 | -8.90 | 5.48E-19 | 7.37E-17 |
| H2-Q5 | 699.09 | -2.09 | 0.23 | -9.06 | 1.32E-19 | 1.83E-17 |
| Gm6377 | 230.26 | -2.09 | 0.49 | -4.26 | 2.08E-05 | 5.81E-04 |
| Gfpt2 | 2728.39 | -2.09 | 0.24 | -8.60 | 8.26E-18 | 1.01E-15 |
| Ddit4 | 2096.99 | -2.11 | 0.54 | -3.91 | 9.29E-05 | 2.21E-03 |
| Pnp | 1317.17 | -2.11 | 0.27 | -7.78 | 7.34E-15 | 6.81E-13 |
| Ralgds | 1370.76 | -2.12 | 0.17 | -12.15 | 6.05E-34 | 3.26E-31 |
| Hspa1a | 20241.84 | -2.13 | 0.81 | -2.64 | 8.35E-03 | 9.13E-02 |
| Ifi213 | 115.20 | -2.13 | 0.37 | -5.72 | 1.09E-08 | 5.02E-07 |
| Igtp | 1109.19 | -2.14 | 0.33 | -6.56 | 5.32E-11 | 3.15E-09 |
| Trex1 | 715.24 | -2.14 | 0.28 | -7.75 | 9.13E-15 | 8.35E-13 |
| Batf3 | 21.47 | -2.14 | 0.80 | -2.68 | 7.31E-03 | 8.36E-02 |
| Slfn3 | 298.35 | -2.15 | 0.26 | -8.27 | 1.32E-16 | 1.47E-14 |
| Ncoa7 | 313.50 | -2.15 | 0.38 | -5.67 | 1.46E-08 | 6.62E-07 |
| H2-Q10 | 430.31 | -2.17 | 0.26 | -8.33 | 8.41E-17 | 9.57E-15 |
| Tnip1 | 3098.02 | -2.18 | 0.16 | -13.58 | 5.12E-42 | 4.78E-39 |
| Irf7 | 978.34 | -2.18 | 0.27 | -7.98 | 1.46E-15 | 1.46E-13 |
| Oas1a | 300.15 | -2.20 | 0.27 | -8.09 | 5.75E-16 | 6.01E-14 |
| Osmr | 2424.71 | -2.21 | 0.26 | -8.65 | 5.28E-18 | 6.72E-16 |
| Oasl2 | 1000.96 | -2.21 | 0.29 | -7.54 | 4.70E-14 | 3.92E-12 |
| Olfr56 | 39.90 | -2.21 | 0.58 | -3.81 | 1.39E-04 | 3.16E-03 |
| Ccl11 | 206.00 | -2.21 | 0.37 | -6.01 | 1.83E-09 | 9.20E-08 |
| Socs3 | 8192.75 | -2.22 | 0.50 | -4.46 | 8.20E-06 | 2.47E-04 |
| Foxf1 | 40.92 | -2.22 | 0.56 | -3.94 | 8.12E-05 | 1.95E-03 |
| Fam124b | 42.85 | -2.23 | 0.62 | -3.58 | 3.49E-04 | 6.93E-03 |
| Ngf | 744.89 | -2.23 | 0.32 | -6.96 | 3.32E-12 | 2.24E-10 |
| Nlrp3 | 572.02 | -2.23 | 0.23 | -9.75 | 1.94E-22 | 3.52E-20 |
| Tlr2 | 992.61 | -2.24 | 0.36 | -6.22 | 4.91E-10 | 2.64E-08 |
| Il1a | 314.24 | -2.24 | 0.41 | -5.45 | 5.04E-08 | 2.07E-06 |
| Gbp3 | 1305.65 | -2.24 | 0.22 | -10.13 | 4.14E-24 | 9.21E-22 |
| Hp | 44.80 | -2.24 | 0.62 | -3.60 | 3.21E-04 | 6.47E-03 |
| Nfkbia | 7170.52 | -2.24 | 0.23 | -9.63 | 5.87E-22 | 1.03E-19 |
| Spp1 | 24.65 | -2.24 | 0.84 | -2.67 | 7.58E-03 | 8.54E-02 |
| Ccl12 | 178.42 | -2.26 | 0.50 | -4.54 | 5.70E-06 | 1.78E-04 |
| Helz2 | 4802.12 | -2.26 | 0.24 | -9.35 | 8.62E-21 | 1.36E-18 |
| Upp1 | 314.52 | -2.26 | 0.27 | -8.34 | 7.18E-17 | 8.31E-15 |
| Traf1 | 217.00 | -2.27 | 0.39 | -5.82 | 5.99E-09 | 2.83E-07 |
| Litaf | 2269.00 | -2.28 | 0.23 | -9.77 | 1.59E-22 | 3.01E-20 |
| Tmem132e | 32.97 | -2.28 | 0.66 | -3.45 | 5.58E-04 | 1.03E-02 |
| Gm49392 | 53.45 | -2.30 | 0.54 | -4.28 | 1.91E-05 | 5.41E-04 |
| Panx1 | 161.57 | -2.30 | 0.31 | -7.49 | 6.80E-14 | 5.57E-12 |
| Plek | 1590.09 | -2.30 | 0.31 | -7.47 | 7.75E-14 | 6.24E-12 |
| Inhbb | 218.58 | -2.32 | 0.39 | -5.91 | 3.48E-09 | 1.67E-07 |
| Ifi207 | 1356.65 | -2.32 | 0.31 | -7.40 | 1.33E-13 | 1.05E-11 |
| Pdpn | 331.92 | -2.32 | 0.36 | -6.48 | 9.11E-11 | 5.23E-09 |
| Usp18 | 354.00 | -2.32 | 0.31 | -7.40 | 1.39E-13 | 1.10E-11 |
| Tgif2 | 219.76 | -2.32 | 0.47 | -4.97 | 6.80E-07 | 2.41E-05 |
| Herc6 | 570.64 | -2.33 | 0.20 | -11.56 | 6.38E-31 | 2.35E-28 |
| Lhfpl2 | 361.98 | -2.34 | 0.22 | -10.54 | 5.71E-26 | 1.63E-23 |
| Fgr | 32.58 | -2.34 | 0.85 | -2.74 | 6.07E-03 | 7.24E-02 |
| Relb | 1177.76 | -2.34 | 0.22 | -10.52 | 6.71E-26 | 1.88E-23 |
| Oas1g | 112.66 | -2.34 | 0.42 | -5.53 | 3.24E-08 | 1.38E-06 |
| Bdkrb2 | 142.35 | -2.34 | 0.41 | -5.70 | 1.22E-08 | 5.58E-07 |
| H2-Q6 | 2540.80 | -2.35 | 0.17 | -13.90 | 6.49E-44 | 6.99E-41 |
| Dll1 | 496.19 | -2.36 | 0.36 | -6.62 | 3.52E-11 | 2.13E-09 |
| Irgm1 | 1607.42 | -2.37 | 0.27 | -8.91 | 4.94E-19 | 6.72E-17 |
| Sowahc | 434.00 | -2.38 | 0.26 | -9.23 | 2.59E-20 | 3.82E-18 |
| Rgs16 | 858.69 | -2.39 | 0.62 | -3.82 | 1.31E-04 | 3.00E-03 |
| Cyp1b1 | 1126.86 | -2.39 | 0.30 | -8.02 | 1.04E-15 | 1.06E-13 |
| Ctrl | 16.94 | -2.39 | 0.90 | -2.65 | 8.13E-03 | 8.97E-02 |
| Zc3h12a | 1302.50 | -2.39 | 0.57 | -4.22 | 2.48E-05 | 6.84E-04 |
| Sema4c | 2342.81 | -2.40 | 0.22 | -10.90 | 1.18E-27 | 3.85E-25 |
| Il11 | 32.16 | -2.42 | 0.81 | -3.00 | 2.66E-03 | 3.75E-02 |
| Sh2b2 | 74.51 | -2.42 | 0.43 | -5.60 | 2.18E-08 | 9.59E-07 |
| Serpina3n | 1018.12 | -2.42 | 0.62 | -3.93 | 8.53E-05 | 2.04E-03 |
| Tnfaip2 | 6930.34 | -2.42 | 0.19 | -12.94 | 2.82E-38 | 2.32E-35 |
| Areg | 46.23 | -2.43 | 0.72 | -3.37 | 7.54E-04 | 1.33E-02 |
| Il17ra | 681.40 | -2.43 | 0.27 | -9.13 | 6.80E-20 | 9.62E-18 |
| Has2 | 271.07 | -2.45 | 0.35 | -6.95 | 3.64E-12 | 2.44E-10 |
| Phf11a | 17.46 | -2.46 | 0.88 | -2.79 | 5.21E-03 | 6.43E-02 |
| Dhx58 | 193.18 | -2.48 | 0.34 | -7.20 | 6.14E-13 | 4.41E-11 |
| B4galt5 | 1285.02 | -2.49 | 0.21 | -11.68 | 1.64E-31 | 6.57E-29 |
| Nptx1 | 23.52 | -2.49 | 0.84 | -2.96 | 3.08E-03 | 4.21E-02 |
| Trim30b | 33.58 | -2.50 | 0.64 | -3.89 | 1.02E-04 | 2.41E-03 |
| Ifi209 | 132.93 | -2.50 | 0.38 | -6.59 | 4.31E-11 | 2.57E-09 |
| Il4ra | 2208.10 | -2.51 | 0.28 | -9.08 | 1.05E-19 | 1.47E-17 |
| Gadd45b | 3835.45 | -2.51 | 0.26 | -9.51 | 1.89E-21 | 3.14E-19 |
| Gm4951 | 86.58 | -2.52 | 0.47 | -5.30 | 1.16E-07 | 4.54E-06 |
| Oas3 | 56.28 | -2.53 | 0.60 | -4.18 | 2.95E-05 | 7.98E-04 |
| Csf3r | 140.42 | -2.53 | 0.48 | -5.32 | 1.06E-07 | 4.17E-06 |
| Gem | 1714.27 | -2.54 | 0.24 | -10.74 | 6.37E-27 | 1.98E-24 |
| Slc41a2 | 109.56 | -2.54 | 0.40 | -6.29 | 3.09E-10 | 1.70E-08 |
| Lcp2 | 385.11 | -2.54 | 0.27 | -9.28 | 1.71E-20 | 2.58E-18 |
| Csrnp1 | 3618.52 | -2.54 | 0.27 | -9.33 | 1.09E-20 | 1.69E-18 |
| Slfn9 | 351.26 | -2.56 | 0.35 | -7.38 | 1.57E-13 | 1.23E-11 |
| Parp14 | 3660.57 | -2.56 | 0.24 | -10.89 | 1.26E-27 | 4.00E-25 |
| Bcl2a1b | 219.24 | -2.57 | 0.37 | -6.87 | 6.60E-12 | 4.28E-10 |
| Gbp2 | 2630.16 | -2.58 | 0.23 | -11.03 | 2.70E-28 | 9.00E-26 |
| Ripk2 | 725.55 | -2.60 | 0.31 | -8.30 | 1.06E-16 | 1.19E-14 |
| Irf8 | 774.23 | -2.60 | 0.34 | -7.69 | 1.49E-14 | 1.33E-12 |
| Birc3 | 2089.86 | -2.61 | 0.32 | -8.18 | 2.89E-16 | 3.08E-14 |
| Sdc4 | 2583.33 | -2.61 | 0.23 | -11.47 | 1.82E-30 | 6.38E-28 |
| Rtp4 | 629.11 | -2.62 | 0.28 | -9.24 | 2.58E-20 | 3.82E-18 |
| Isg20 | 218.71 | -2.62 | 0.31 | -8.33 | 7.98E-17 | 9.16E-15 |
| Runx1 | 245.98 | -2.63 | 0.27 | -9.70 | 2.94E-22 | 5.28E-20 |
| Cmpk2 | 1095.08 | -2.63 | 0.25 | -10.66 | 1.59E-26 | 4.66E-24 |
| Ccl3 | 952.98 | -2.63 | 0.35 | -7.63 | 2.29E-14 | 2.01E-12 |
| Ccl8 | 43.64 | -2.64 | 0.72 | -3.68 | 2.30E-04 | 4.86E-03 |
| Ifih1 | 1365.79 | -2.67 | 0.25 | -10.46 | 1.26E-25 | 3.46E-23 |
| Acp5 | 89.20 | -2.67 | 0.45 | -5.91 | 3.41E-09 | 1.64E-07 |
| Slc10a6 | 388.52 | -2.67 | 0.26 | -10.15 | 3.37E-24 | 7.73E-22 |
| Tgtp2 | 1371.61 | -2.69 | 0.30 | -8.87 | 7.63E-19 | 1.01E-16 |
| Dusp2 | 217.42 | -2.70 | 0.36 | -7.46 | 8.54E-14 | 6.83E-12 |
| Cd274 | 1230.19 | -2.70 | 0.27 | -9.98 | 1.77E-23 | 3.65E-21 |
| Slfn10-ps | 21.32 | -2.71 | 0.87 | -3.12 | 1.83E-03 | 2.77E-02 |
| Ifi206 | 56.19 | -2.72 | 0.50 | -5.45 | 5.02E-08 | 2.07E-06 |
| Cebpd | 2735.69 | -2.72 | 0.23 | -11.60 | 4.19E-31 | 1.59E-28 |
| Gm49339 | 301.36 | -2.73 | 0.29 | -9.29 | 1.52E-20 | 2.32E-18 |
| Ifi204 | 1012.55 | -2.73 | 0.35 | -7.89 | 3.10E-15 | 2.99E-13 |
| Gm12185 | 93.53 | -2.74 | 0.51 | -5.40 | 6.59E-08 | 2.68E-06 |
| Slfn2 | 1032.14 | -2.75 | 0.24 | -11.35 | 7.18E-30 | 2.45E-27 |
| Ackr1 | 17.49 | -2.75 | 0.91 | -3.01 | 2.64E-03 | 3.72E-02 |
| Cxcl2 | 3766.85 | -2.76 | 0.32 | -8.71 | 2.92E-18 | 3.75E-16 |
| Csf1 | 9083.75 | -2.76 | 0.23 | -11.84 | 2.36E-32 | 1.06E-29 |
| Neurl3 | 4240.04 | -2.76 | 0.65 | -4.23 | 2.36E-05 | 6.55E-04 |
| AC109138.2 | 2374.72 | -2.79 | 0.16 | -17.19 | 3.21E-66 | 6.41E-63 |
| Vcam1 | 5525.57 | -2.79 | 0.24 | -11.76 | 6.27E-32 | 2.58E-29 |
| Slc2a6 | 147.05 | -2.80 | 0.48 | -5.89 | 3.95E-09 | 1.89E-07 |
| Il4i1 | 44.90 | -2.81 | 0.55 | -5.10 | 3.41E-07 | 1.26E-05 |
| Gm18853 | 287.16 | -2.82 | 0.47 | -6.05 | 1.46E-09 | 7.49E-08 |
| Mt2 | 4403.46 | -2.82 | 0.50 | -5.69 | 1.24E-08 | 5.64E-07 |
| Thbs1 | 13182.74 | -2.84 | 0.29 | -9.95 | 2.43E-23 | 4.92E-21 |
| Slfn4 | 58.83 | -2.85 | 0.56 | -5.12 | 3.07E-07 | 1.15E-05 |
| Foxj1 | 32.95 | -2.85 | 0.94 | -3.02 | 2.51E-03 | 3.59E-02 |
| Irf1 | 7395.79 | -2.86 | 0.28 | -10.22 | 1.66E-24 | 3.88E-22 |
| Cfb | 173.51 | -2.86 | 0.35 | -8.27 | 1.35E-16 | 1.48E-14 |
| Cgas | 175.30 | -2.87 | 0.36 | -8.04 | 9.24E-16 | 9.51E-14 |
| Tifa | 523.66 | -2.88 | 0.37 | -7.74 | 9.60E-15 | 8.73E-13 |
| Tap1 | 1310.55 | -2.88 | 0.22 | -12.90 | 4.42E-38 | 3.27E-35 |
| Slc15a3 | 698.67 | -2.91 | 0.23 | -12.42 | 1.96E-35 | 1.14E-32 |
| Olfr1033 | 3661.24 | -2.91 | 0.23 | -12.53 | 5.26E-36 | 3.20E-33 |
| Ccl22 | 47.23 | -2.92 | 0.73 | -3.98 | 6.96E-05 | 1.69E-03 |
| Gm45551 | 1789.66 | -2.93 | 0.19 | -15.22 | 2.44E-52 | 3.11E-49 |
| Cd40 | 437.52 | -2.93 | 0.31 | -9.43 | 4.29E-21 | 6.83E-19 |
| Adora2b | 107.67 | -2.93 | 0.40 | -7.34 | 2.15E-13 | 1.64E-11 |
| Slc4a11 | 18.25 | -2.95 | 1.10 | -2.67 | 7.52E-03 | 8.50E-02 |
| Ifit3b | 450.44 | -2.95 | 0.31 | -9.62 | 6.31E-22 | 1.09E-19 |
| Casp4 | 989.08 | -2.95 | 0.61 | -4.84 | 1.32E-06 | 4.48E-05 |
| Hck | 167.04 | -2.96 | 0.50 | -5.93 | 3.11E-09 | 1.52E-07 |
| Asprv1 | 14.37 | -2.96 | 1.11 | -2.68 | 7.36E-03 | 8.40E-02 |
| Ccl7 | 2435.30 | -2.97 | 0.59 | -5.03 | 5.03E-07 | 1.81E-05 |
| Sbno2 | 5602.25 | -2.97 | 0.51 | -5.80 | 6.71E-09 | 3.14E-07 |
| Nod2 | 840.02 | -2.98 | 0.23 | -12.90 | 4.43E-38 | 3.27E-35 |
| Egr4 | 66.48 | -2.98 | 0.58 | -5.17 | 2.32E-07 | 8.78E-06 |
| Loxl4 | 213.52 | -2.98 | 0.49 | -6.03 | 1.59E-09 | 8.10E-08 |
| Ifi205 | 2309.32 | -3.00 | 0.58 | -5.15 | 2.60E-07 | 9.77E-06 |
| Gbp11 | 11.82 | -3.01 | 1.10 | -2.74 | 6.14E-03 | 7.29E-02 |
| Ptgs2 | 2466.02 | -3.04 | 0.24 | -12.65 | 1.08E-36 | 7.18E-34 |
| Slfn8 | 435.56 | -3.06 | 0.33 | -9.22 | 2.97E-20 | 4.33E-18 |
| Zbp1 | 139.78 | -3.06 | 0.38 | -8.16 | 3.41E-16 | 3.62E-14 |
| Bcl2a1d | 93.87 | -3.07 | 0.48 | -6.42 | 1.32E-10 | 7.51E-09 |
| Fjx1 | 104.27 | -3.08 | 0.52 | -5.94 | 2.87E-09 | 1.40E-07 |
| Bcl3 | 1281.70 | -3.09 | 0.29 | -10.70 | 9.92E-27 | 3.02E-24 |
| Rsad2 | 3481.66 | -3.09 | 0.26 | -11.77 | 5.52E-32 | 2.34E-29 |
| Sgms2 | 207.06 | -3.09 | 0.47 | -6.53 | 6.71E-11 | 3.91E-09 |
| Batf2 | 88.04 | -3.10 | 0.46 | -6.81 | 9.48E-12 | 6.00E-10 |
| Tnfaip3 | 7815.14 | -3.10 | 0.64 | -4.85 | 1.24E-06 | 4.24E-05 |
| Gm49342 | 295.02 | -3.11 | 0.30 | -10.38 | 3.10E-25 | 7.90E-23 |
| Sphk1 | 336.98 | -3.11 | 0.77 | -4.05 | 5.06E-05 | 1.29E-03 |
| Marcksl1 | 2146.77 | -3.12 | 0.30 | -10.39 | 2.80E-25 | 7.27E-23 |
| Tgtp1 | 1081.03 | -3.21 | 0.31 | -10.42 | 2.02E-25 | 5.33E-23 |
| Gm8818 | 16.22 | -3.22 | 1.08 | -2.99 | 2.83E-03 | 3.93E-02 |
| Gm13889 | 1248.15 | -3.22 | 0.33 | -9.80 | 1.17E-22 | 2.24E-20 |
| Ifi211 | 1286.83 | -3.23 | 0.58 | -5.57 | 2.57E-08 | 1.12E-06 |
| B3gnt7 | 10.56 | -3.24 | 1.25 | -2.61 | 9.18E-03 | 9.79E-02 |
| Sec1 | 23.48 | -3.25 | 0.91 | -3.56 | 3.76E-04 | 7.32E-03 |
| Cxcl1 | 9827.68 | -3.25 | 0.26 | -12.55 | 3.98E-36 | 2.53E-33 |
| Rnd1 | 5005.15 | -3.25 | 0.64 | -5.09 | 3.53E-07 | 1.30E-05 |
| Tnfaip6 | 1216.38 | -3.26 | 0.27 | -12.14 | 6.55E-34 | 3.40E-31 |
| Pcdh10 | 16.30 | -3.27 | 1.02 | -3.21 | 1.31E-03 | 2.12E-02 |
| Adamts8 | 177.35 | -3.27 | 0.40 | -8.18 | 2.77E-16 | 2.99E-14 |
| Clec4e | 201.65 | -3.28 | 0.33 | -10.08 | 6.79E-24 | 1.44E-21 |
| AA467197 | 12.92 | -3.29 | 1.19 | -2.75 | 5.88E-03 | 7.05E-02 |
| Tnc | 256.17 | -3.29 | 0.88 | -3.73 | 1.90E-04 | 4.13E-03 |
| Ifit3 | 1802.23 | -3.31 | 0.32 | -10.43 | 1.74E-25 | 4.68E-23 |
| Fam83a | 24.31 | -3.31 | 0.94 | -3.51 | 4.53E-04 | 8.68E-03 |
| Cd69 | 31.05 | -3.31 | 0.97 | -3.43 | 5.97E-04 | 1.09E-02 |
| Prr7 | 210.43 | -3.32 | 0.37 | -8.89 | 6.24E-19 | 8.33E-17 |
| Ccl19 | 77.21 | -3.34 | 0.46 | -7.29 | 3.20E-13 | 2.38E-11 |
| Batf | 123.74 | -3.34 | 0.40 | -8.44 | 3.18E-17 | 3.71E-15 |
| Procr | 325.20 | -3.35 | 0.37 | -9.14 | 6.46E-20 | 9.23E-18 |
| Slfn1 | 30.95 | -3.39 | 0.75 | -4.54 | 5.66E-06 | 1.77E-04 |
| Ifit2 | 2999.77 | -3.42 | 0.54 | -6.29 | 3.24E-10 | 1.77E-08 |
| Tfec | 52.35 | -3.44 | 0.58 | -5.95 | 2.63E-09 | 1.30E-07 |
| Ifi47 | 454.00 | -3.46 | 0.29 | -11.90 | 1.16E-32 | 5.58E-30 |
| Clec4d | 93.03 | -3.46 | 0.53 | -6.54 | 6.27E-11 | 3.70E-09 |
| Gbp5 | 2623.48 | -3.46 | 0.27 | -12.71 | 4.89E-37 | 3.43E-34 |
| Lrguk | 9.62 | -3.47 | 1.18 | -2.93 | 3.37E-03 | 4.52E-02 |
| Mx1 | 279.47 | -3.48 | 0.80 | -4.36 | 1.33E-05 | 3.85E-04 |
| Tnip3 | 331.06 | -3.50 | 0.29 | -11.89 | 1.32E-32 | 6.18E-30 |
| Tfpi2 | 241.58 | -3.50 | 0.47 | -7.38 | 1.60E-13 | 1.24E-11 |
| Ccl2 | 5754.16 | -3.55 | 0.31 | -11.62 | 3.36E-31 | 1.31E-28 |
| Bcl2a1a | 41.70 | -3.58 | 0.66 | -5.38 | 7.51E-08 | 3.02E-06 |
| Car13 | 133.80 | -3.61 | 0.38 | -9.55 | 1.25E-21 | 2.14E-19 |
| Serpina3i | 24.87 | -3.62 | 0.84 | -4.28 | 1.86E-05 | 5.29E-04 |
| Mmp13 | 607.11 | -3.62 | 0.37 | -9.80 | 1.13E-22 | 2.21E-20 |
| Olr1 | 171.36 | -3.63 | 0.42 | -8.60 | 7.99E-18 | 9.89E-16 |
| Ereg | 49.94 | -3.64 | 0.58 | -6.21 | 5.15E-10 | 2.75E-08 |
| Mx2 | 767.86 | -3.66 | 0.35 | -10.37 | 3.51E-25 | 8.78E-23 |
| Ccl4 | 1204.39 | -3.66 | 0.32 | -11.55 | 7.47E-31 | 2.68E-28 |
| Pik3r5 | 483.91 | -3.67 | 0.22 | -16.59 | 8.70E-62 | 1.35E-58 |
| Pfkfb3 | 4469.70 | -3.69 | 0.20 | -18.51 | 1.67E-76 | 7.77E-73 |
| Ikzf4 | 259.05 | -3.74 | 0.81 | -4.63 | 3.67E-06 | 1.19E-04 |
| Cxcl5 | 734.12 | -3.78 | 0.87 | -4.36 | 1.32E-05 | 3.83E-04 |
| Plaur | 2089.53 | -3.79 | 0.64 | -5.95 | 2.66E-09 | 1.31E-07 |
| Olfr961 | 23.15 | -3.81 | 0.93 | -4.09 | 4.28E-05 | 1.10E-03 |
| Iigp1 | 264.26 | -3.82 | 0.39 | -9.85 | 7.02E-23 | 1.40E-20 |
| Icam1 | 23418.22 | -3.84 | 0.57 | -6.69 | 2.19E-11 | 1.35E-09 |
| Vgf | 15.19 | -3.85 | 1.21 | -3.19 | 1.42E-03 | 2.26E-02 |
| Timp1 | 512.89 | -3.88 | 0.41 | -9.52 | 1.78E-21 | 3.00E-19 |
| Samsn1 | 145.33 | -3.90 | 0.45 | -8.74 | 2.39E-18 | 3.10E-16 |
| Gm5970 | 28.86 | -3.90 | 0.80 | -4.89 | 1.03E-06 | 3.55E-05 |
| Il10 | 26.49 | -3.91 | 0.90 | -4.36 | 1.32E-05 | 3.83E-04 |
| Hcar2 | 34.22 | -3.95 | 0.82 | -4.82 | 1.47E-06 | 4.93E-05 |
| Lif | 512.78 | -4.03 | 0.34 | -11.84 | 2.42E-32 | 1.06E-29 |
| Hbb-bs | 847.59 | -4.04 | 1.55 | -2.61 | 9.18E-03 | 9.79E-02 |
| Fpr1 | 8.16 | -4.10 | 1.49 | -2.76 | 5.87E-03 | 7.05E-02 |
| Serpinb2 | 33.83 | -4.16 | 0.95 | -4.40 | 1.08E-05 | 3.18E-04 |
| Oasl1 | 936.49 | -4.19 | 0.31 | -13.65 | 1.94E-42 | 1.94E-39 |
| A730049H05Rik | 56.28 | -4.19 | 0.78 | -5.35 | 8.87E-08 | 3.53E-06 |
| Stx11 | 1131.27 | -4.20 | 0.57 | -7.34 | 2.14E-13 | 1.64E-11 |
| Ifit1 | 2680.58 | -4.20 | 0.59 | -7.07 | 1.59E-12 | 1.10E-10 |
| Wfdc18 | 8.08 | -4.24 | 1.47 | -2.89 | 3.85E-03 | 5.04E-02 |
| Trim30c | 62.55 | -4.27 | 0.62 | -6.91 | 4.92E-12 | 3.24E-10 |
| Ifnb1 | 65.44 | -4.28 | 0.75 | -5.70 | 1.18E-08 | 5.42E-07 |
| Hbb-bt | 111.12 | -4.28 | 1.49 | -2.88 | 4.02E-03 | 5.21E-02 |
| Sell | 9.26 | -4.29 | 1.28 | -3.34 | 8.29E-04 | 1.44E-02 |
| Chil1 | 11.11 | -4.37 | 1.38 | -3.16 | 1.58E-03 | 2.48E-02 |
| Csf2 | 89.48 | -4.52 | 0.75 | -6.03 | 1.62E-09 | 8.23E-08 |
| Cxcl3 | 117.35 | -4.53 | 1.11 | -4.09 | 4.30E-05 | 1.11E-03 |
| Selp | 2632.62 | -4.53 | 0.26 | -17.23 | 1.65E-66 | 4.63E-63 |
| Sele | 6058.28 | -4.62 | 0.26 | -17.56 | 5.19E-69 | 1.82E-65 |
| Ubd | 14.05 | -4.62 | 1.27 | -3.65 | 2.59E-04 | 5.36E-03 |
| Gm12250 | 163.23 | -4.63 | 0.46 | -10.14 | 3.62E-24 | 8.18E-22 |
| Serpina3f | 195.73 | -4.68 | 0.48 | -9.84 | 7.42E-23 | 1.46E-20 |
| Fpr2 | 40.05 | -4.75 | 0.82 | -5.82 | 5.87E-09 | 2.79E-07 |
| Gm5796 | 24.68 | -4.90 | 1.26 | -3.88 | 1.06E-04 | 2.48E-03 |
| Cxcl10 | 3485.10 | -4.93 | 0.82 | -6.00 | 2.00E-09 | 1.00E-07 |
| Mab21l3 | 14.33 | -4.93 | 1.63 | -3.03 | 2.44E-03 | 3.51E-02 |
| Gm8752 | 23.54 | -4.96 | 1.11 | -4.49 | 7.24E-06 | 2.21E-04 |
| Cxcl9 | 1770.01 | -4.98 | 0.31 | -16.01 | 1.17E-57 | 1.64E-54 |
| Acod1 | 311.44 | -5.03 | 0.81 | -6.24 | 4.37E-10 | 2.37E-08 |
| Cxcl11 | 64.40 | -5.08 | 0.62 | -8.15 | 3.53E-16 | 3.72E-14 |
| Il12b | 12.55 | -5.09 | 1.69 | -3.01 | 2.60E-03 | 3.68E-02 |
| Ptx3 | 5910.66 | -5.11 | 0.30 | -16.85 | 1.04E-63 | 1.83E-60 |
| Sh2d5 | 93.66 | -5.14 | 0.71 | -7.22 | 5.13E-13 | 3.74E-11 |
| Ccl5 | 282.32 | -5.19 | 0.39 | -13.24 | 5.48E-40 | 4.79E-37 |
| Csf3 | 222.38 | -5.31 | 0.44 | -11.99 | 4.11E-33 | 2.05E-30 |
| Il6 | 3330.75 | -5.34 | 0.53 | -10.11 | 4.79E-24 | 1.05E-21 |
| Fgf23 | 49.83 | -5.38 | 0.86 | -6.23 | 4.59E-10 | 2.48E-08 |
| Serpina3g | 14.64 | -5.39 | 1.36 | -3.96 | 7.60E-05 | 1.83E-03 |
| Nipal1 | 10.98 | -5.43 | 1.70 | -3.19 | 1.40E-03 | 2.24E-02 |
| Adamts4 | 6764.22 | -5.51 | 0.53 | -10.34 | 4.67E-25 | 1.15E-22 |
| Lipg | 98.38 | -5.58 | 0.65 | -8.54 | 1.37E-17 | 1.63E-15 |
| Il1rn | 506.42 | -5.65 | 0.33 | -17.21 | 2.25E-66 | 5.25E-63 |
| S100a9 | 42.17 | -5.74 | 1.02 | -5.63 | 1.84E-08 | 8.27E-07 |
| Calhm6 | 51.56 | -5.88 | 0.85 | -6.91 | 4.93E-12 | 3.24E-10 |
| Mcoln2 | 163.25 | -6.01 | 0.63 | -9.48 | 2.48E-21 | 4.04E-19 |
| Slc6a12 | 8.48 | -6.03 | 1.69 | -3.57 | 3.60E-04 | 7.09E-03 |
| Kctd20 | 10.26 | -6.25 | 1.79 | -3.50 | 4.73E-04 | 8.96E-03 |
| Acat3 | 9.74 | -6.76 | 1.88 | -3.60 | 3.19E-04 | 6.44E-03 |
| Rpl7-ps8 | 8.93 | -6.78 | 1.78 | -3.81 | 1.37E-04 | 3.11E-03 |
| Gm10240 | 30.09 | -6.89 | 1.64 | -4.19 | 2.75E-05 | 7.52E-04 |
| Gm13370 | 30.09 | -6.89 | 1.64 | -4.19 | 2.75E-05 | 7.52E-04 |
| Cbx3-ps6 | 25.01 | -7.00 | 1.81 | -3.87 | 1.10E-04 | 2.56E-03 |
| Alas2 | 15.84 | -7.37 | 1.96 | -3.76 | 1.71E-04 | 3.76E-03 |
| S100a8 | 21.75 | -8.50 | 1.69 | -5.03 | 5.03E-07 | 1.81E-05 |
| Gm50241 | 76.90 | -22.16 | 4.79 | -4.63 | 3.63E-06 | 1.18E-04 |

RV4h vs RV0h DEGs

|  | baseMean | log2FoldChange | lfcSE | stat | pvalue | padj |
| --- | --- | --- | --- | --- | --- | --- |
| Gm28439 | 1394.53 | 27.74 | 4.78 | 5.80 | 6.75E-09 | 2.33E-07 |
| Gm49320 | 6.51 | 6.38 | 2.21 | 2.89 | 3.84E-03 | 3.39E-02 |
| Tll2 | 7.16 | 6.02 | 2.20 | 2.74 | 6.10E-03 | 4.90E-02 |
| Gm3696 | 6.35 | 5.83 | 1.93 | 3.02 | 2.56E-03 | 2.44E-02 |
| Gm47103 | 11.42 | 5.67 | 1.78 | 3.18 | 1.49E-03 | 1.58E-02 |
| Gm9522 | 13.63 | 5.09 | 1.69 | 3.01 | 2.64E-03 | 2.50E-02 |
| 1700030C10Rik | 11.89 | 4.89 | 1.75 | 2.79 | 5.21E-03 | 4.33E-02 |
| Gask1a | 10.68 | 4.02 | 1.40 | 2.87 | 4.17E-03 | 3.63E-02 |
| Kifc1 | 7.99 | 4.01 | 1.54 | 2.60 | 9.27E-03 | 6.91E-02 |
| Gm49378 | 21.33 | 3.38 | 1.16 | 2.90 | 3.69E-03 | 3.27E-02 |
| Tmem240 | 8.31 | 3.30 | 1.32 | 2.51 | 1.22E-02 | 8.45E-02 |
| Gm8189 | 14.29 | 2.86 | 1.14 | 2.50 | 1.24E-02 | 8.55E-02 |
| Cfap100 | 14.03 | 2.84 | 1.11 | 2.56 | 1.06E-02 | 7.63E-02 |
| Vps52 | 17.44 | 2.79 | 0.87 | 3.22 | 1.30E-03 | 1.40E-02 |
| Dbp | 1319.85 | 2.78 | 0.25 | 11.14 | 7.73E-29 | 1.87E-26 |
| Per3 | 625.45 | 2.68 | 0.27 | 9.75 | 1.81E-22 | 2.70E-20 |
| Gpr34 | 17.85 | 2.67 | 0.86 | 3.13 | 1.78E-03 | 1.82E-02 |
| Gm12258 | 17.93 | 2.66 | 0.99 | 2.70 | 6.92E-03 | 5.42E-02 |
| Zfp354c | 47.00 | 2.66 | 0.55 | 4.82 | 1.40E-06 | 3.22E-05 |
| Aplnr | 92.56 | 2.54 | 0.57 | 4.43 | 9.52E-06 | 1.89E-04 |
| Sall2 | 27.83 | 2.47 | 0.66 | 3.72 | 1.99E-04 | 2.77E-03 |
| Flt3l | 123.98 | 2.42 | 0.40 | 6.08 | 1.21E-09 | 4.48E-08 |
| Kcnt1 | 32.29 | 2.32 | 0.60 | 3.84 | 1.22E-04 | 1.80E-03 |
| Gstt2 | 150.03 | 2.26 | 0.41 | 5.49 | 4.10E-08 | 1.26E-06 |
| Myct1 | 216.53 | 2.24 | 0.33 | 6.86 | 7.04E-12 | 3.57E-10 |
| Izumo4 | 53.96 | 2.21 | 0.57 | 3.90 | 9.60E-05 | 1.45E-03 |
| Gng4 | 27.45 | 2.17 | 0.76 | 2.84 | 4.50E-03 | 3.86E-02 |
| Alox8 | 31.13 | 2.15 | 0.73 | 2.94 | 3.32E-03 | 3.00E-02 |
| Clec14a | 185.76 | 2.14 | 0.34 | 6.26 | 3.84E-10 | 1.52E-08 |
| AI854703 | 21.74 | 2.14 | 0.83 | 2.58 | 9.89E-03 | 7.25E-02 |
| Ddit4l | 48.88 | 2.13 | 0.50 | 4.25 | 2.14E-05 | 3.90E-04 |
| B3gnt8 | 32.82 | 2.12 | 0.60 | 3.54 | 3.99E-04 | 5.06E-03 |
| Zfp72 | 43.73 | 2.09 | 0.61 | 3.44 | 5.89E-04 | 7.13E-03 |
| Tceanc | 24.72 | 2.04 | 0.81 | 2.52 | 1.17E-02 | 8.25E-02 |
| Depp1 | 634.37 | 2.04 | 0.30 | 6.84 | 8.04E-12 | 4.01E-10 |
| Phf21b | 74.35 | 2.01 | 0.45 | 4.46 | 8.26E-06 | 1.66E-04 |
| Sv2a | 78.29 | 2.00 | 0.43 | 4.64 | 3.49E-06 | 7.55E-05 |
| Sema6b | 40.96 | 1.99 | 0.72 | 2.76 | 5.77E-03 | 4.69E-02 |
| Zfp128 | 44.16 | 1.98 | 0.61 | 3.26 | 1.12E-03 | 1.24E-02 |
| AW146154 | 67.24 | 1.97 | 0.47 | 4.16 | 3.14E-05 | 5.44E-04 |
| Akr1c19 | 22.88 | 1.94 | 0.74 | 2.61 | 9.10E-03 | 6.80E-02 |
| Hrk | 20.21 | 1.93 | 0.76 | 2.55 | 1.09E-02 | 7.81E-02 |
| Calhm2 | 51.52 | 1.89 | 0.49 | 3.85 | 1.20E-04 | 1.78E-03 |
| Zfp61 | 40.61 | 1.89 | 0.57 | 3.31 | 9.17E-04 | 1.04E-02 |
| Pbld1 | 36.98 | 1.88 | 0.59 | 3.18 | 1.46E-03 | 1.55E-02 |
| Nr2f2 | 236.86 | 1.87 | 0.35 | 5.36 | 8.43E-08 | 2.43E-06 |
| Npy1r | 23.65 | 1.87 | 0.77 | 2.43 | 1.53E-02 | 9.98E-02 |
| Ankrd63 | 51.71 | 1.85 | 0.49 | 3.80 | 1.47E-04 | 2.11E-03 |
| Lrrc70 | 24.79 | 1.84 | 0.70 | 2.63 | 8.47E-03 | 6.40E-02 |
| Egfl7 | 416.45 | 1.84 | 0.26 | 7.02 | 2.19E-12 | 1.23E-10 |
| Kantr | 35.46 | 1.83 | 0.70 | 2.61 | 8.94E-03 | 6.70E-02 |
| Dcdc2b | 30.70 | 1.82 | 0.65 | 2.81 | 4.93E-03 | 4.14E-02 |
| Fut10 | 48.95 | 1.82 | 0.53 | 3.45 | 5.66E-04 | 6.92E-03 |
| Il2ra | 64.18 | 1.81 | 0.46 | 3.94 | 8.16E-05 | 1.27E-03 |
| Nynrin | 96.99 | 1.80 | 0.39 | 4.61 | 4.06E-06 | 8.67E-05 |
| Gm14440 | 321.28 | 1.78 | 0.42 | 4.20 | 2.70E-05 | 4.76E-04 |
| Gimap1 | 74.68 | 1.78 | 0.42 | 4.22 | 2.45E-05 | 4.36E-04 |
| Zfp60 | 178.70 | 1.77 | 0.38 | 4.72 | 2.37E-06 | 5.29E-05 |
| Cys1 | 104.85 | 1.77 | 0.45 | 3.94 | 8.17E-05 | 1.27E-03 |
| Sox4 | 1365.40 | 1.76 | 0.20 | 8.89 | 6.28E-19 | 7.06E-17 |
| Shpk | 37.35 | 1.75 | 0.57 | 3.07 | 2.16E-03 | 2.13E-02 |
| Kctd12b | 129.44 | 1.75 | 0.34 | 5.07 | 3.88E-07 | 1.01E-05 |
| Zfp964 | 28.94 | 1.74 | 0.65 | 2.70 | 7.01E-03 | 5.48E-02 |
| Zfp759 | 63.12 | 1.74 | 0.48 | 3.58 | 3.39E-04 | 4.41E-03 |
| Zfp433 | 58.51 | 1.73 | 0.57 | 3.02 | 2.53E-03 | 2.42E-02 |
| Gal3st2b | 25.41 | 1.73 | 0.69 | 2.52 | 1.18E-02 | 8.30E-02 |
| Zfp607b | 24.94 | 1.72 | 0.66 | 2.59 | 9.50E-03 | 7.03E-02 |
| Chst12 | 47.20 | 1.70 | 0.56 | 3.02 | 2.56E-03 | 2.44E-02 |
| Rufy2 | 159.99 | 1.69 | 0.43 | 3.94 | 8.17E-05 | 1.27E-03 |
| Neurl1b | 91.92 | 1.69 | 0.40 | 4.18 | 2.86E-05 | 5.02E-04 |
| Stk38 | 74.32 | 1.69 | 0.42 | 3.99 | 6.57E-05 | 1.04E-03 |
| D630003M21Rik | 144.02 | 1.68 | 0.33 | 5.03 | 4.85E-07 | 1.23E-05 |
| Lmntd1 | 54.65 | 1.68 | 0.67 | 2.52 | 1.19E-02 | 8.32E-02 |
| Zfp280b | 117.10 | 1.67 | 0.36 | 4.59 | 4.47E-06 | 9.44E-05 |
| Zfp606 | 139.93 | 1.67 | 0.34 | 4.87 | 1.10E-06 | 2.57E-05 |
| Exog | 30.19 | 1.67 | 0.68 | 2.46 | 1.38E-02 | 9.28E-02 |
| Tmem204 | 464.69 | 1.67 | 0.24 | 6.96 | 3.45E-12 | 1.86E-10 |
| Gstt3 | 56.76 | 1.67 | 0.51 | 3.29 | 1.00E-03 | 1.13E-02 |
| Pirt | 176.00 | 1.65 | 0.35 | 4.75 | 2.00E-06 | 4.48E-05 |
| Zfp810 | 94.12 | 1.64 | 0.39 | 4.25 | 2.17E-05 | 3.94E-04 |
| Zfp763 | 36.22 | 1.63 | 0.59 | 2.77 | 5.66E-03 | 4.62E-02 |
| Pik3ip1 | 1091.23 | 1.63 | 0.20 | 8.22 | 1.97E-16 | 1.74E-14 |
| Cbx2 | 52.42 | 1.63 | 0.50 | 3.28 | 1.04E-03 | 1.17E-02 |
| A430033K04Rik | 50.58 | 1.63 | 0.54 | 3.03 | 2.42E-03 | 2.33E-02 |
| Casp2 | 125.10 | 1.63 | 0.34 | 4.78 | 1.75E-06 | 3.97E-05 |
| Fibin | 179.33 | 1.63 | 0.38 | 4.24 | 2.26E-05 | 4.06E-04 |
| Bcl11b | 30.66 | 1.62 | 0.64 | 2.53 | 1.14E-02 | 8.08E-02 |
| Dvl3 | 40.77 | 1.62 | 0.53 | 3.04 | 2.36E-03 | 2.30E-02 |
| Zfp661 | 43.84 | 1.62 | 0.55 | 2.97 | 2.98E-03 | 2.74E-02 |
| 1700029J07Rik | 43.21 | 1.62 | 0.57 | 2.84 | 4.56E-03 | 3.90E-02 |
| Slc35d2 | 31.49 | 1.61 | 0.62 | 2.61 | 8.99E-03 | 6.74E-02 |
| Hs3st5 | 44.51 | 1.61 | 0.64 | 2.52 | 1.18E-02 | 8.29E-02 |
| 1700030K09Rik | 108.01 | 1.59 | 0.38 | 4.14 | 3.47E-05 | 5.93E-04 |
| Dynlt1c | 116.73 | 1.58 | 0.35 | 4.53 | 6.02E-06 | 1.24E-04 |
| Ntf3 | 35.45 | 1.58 | 0.64 | 2.49 | 1.29E-02 | 8.84E-02 |
| Zfp760 | 33.41 | 1.58 | 0.61 | 2.61 | 9.02E-03 | 6.75E-02 |
| Slx1b | 122.43 | 1.57 | 0.36 | 4.34 | 1.42E-05 | 2.70E-04 |
| Zkscan8 | 159.91 | 1.55 | 0.44 | 3.55 | 3.91E-04 | 4.98E-03 |
| Gm42688 | 51.03 | 1.54 | 0.53 | 2.88 | 4.03E-03 | 3.52E-02 |
| Rasd2 | 70.72 | 1.53 | 0.48 | 3.22 | 1.30E-03 | 1.40E-02 |
| Card6 | 125.13 | 1.53 | 0.41 | 3.68 | 2.35E-04 | 3.19E-03 |
| Proser3 | 74.04 | 1.53 | 0.43 | 3.57 | 3.53E-04 | 4.55E-03 |
| Tnfrsf25 | 57.80 | 1.52 | 0.53 | 2.85 | 4.32E-03 | 3.74E-02 |
| Zfp862-ps | 108.62 | 1.51 | 0.36 | 4.15 | 3.26E-05 | 5.62E-04 |
| Zfp963 | 52.21 | 1.51 | 0.49 | 3.10 | 1.95E-03 | 1.96E-02 |
| Ccdc8 | 83.12 | 1.51 | 0.48 | 3.16 | 1.57E-03 | 1.64E-02 |
| Angptl7 | 52.07 | 1.51 | 0.62 | 2.43 | 1.50E-02 | 9.88E-02 |
| Aatk | 144.01 | 1.51 | 0.36 | 4.16 | 3.15E-05 | 5.45E-04 |
| Smad9 | 41.57 | 1.50 | 0.60 | 2.49 | 1.30E-02 | 8.85E-02 |
| Saraf | 57.28 | 1.50 | 0.56 | 2.69 | 7.09E-03 | 5.53E-02 |
| Zfp39 | 57.94 | 1.49 | 0.53 | 2.82 | 4.75E-03 | 4.02E-02 |
| Zfp605 | 40.64 | 1.49 | 0.59 | 2.51 | 1.22E-02 | 8.45E-02 |
| Hspa12b | 547.51 | 1.46 | 0.22 | 6.76 | 1.36E-11 | 6.58E-10 |
| AC149090.1 | 654.02 | 1.45 | 0.21 | 6.94 | 4.04E-12 | 2.12E-10 |
| Rnf152 | 61.34 | 1.45 | 0.50 | 2.91 | 3.65E-03 | 3.24E-02 |
| Slc25a45 | 46.50 | 1.44 | 0.51 | 2.84 | 4.48E-03 | 3.84E-02 |
| Prickle1 | 374.60 | 1.44 | 0.33 | 4.34 | 1.44E-05 | 2.72E-04 |
| St6galnac2 | 163.67 | 1.44 | 0.35 | 4.05 | 5.03E-05 | 8.19E-04 |
| Lysmd1 | 95.00 | 1.44 | 0.37 | 3.84 | 1.21E-04 | 1.79E-03 |
| Aldh1l1 | 138.99 | 1.43 | 0.34 | 4.24 | 2.25E-05 | 4.05E-04 |
| Mansc1 | 37.72 | 1.42 | 0.57 | 2.49 | 1.27E-02 | 8.72E-02 |
| Calcoco1 | 379.28 | 1.42 | 0.35 | 4.11 | 3.93E-05 | 6.57E-04 |
| Hlf | 635.89 | 1.42 | 0.26 | 5.53 | 3.27E-08 | 1.01E-06 |
| Zfp169 | 45.06 | 1.41 | 0.57 | 2.48 | 1.30E-02 | 8.89E-02 |
| Acvr2b | 61.71 | 1.41 | 0.46 | 3.10 | 1.91E-03 | 1.93E-02 |
| Trib2 | 499.65 | 1.39 | 0.22 | 6.28 | 3.32E-10 | 1.32E-08 |
| Tbx2 | 147.89 | 1.39 | 0.34 | 4.13 | 3.65E-05 | 6.19E-04 |
| Palm | 680.53 | 1.39 | 0.30 | 4.68 | 2.92E-06 | 6.38E-05 |
| Kcna2 | 96.89 | 1.39 | 0.43 | 3.26 | 1.12E-03 | 1.24E-02 |
| Tril | 88.30 | 1.39 | 0.43 | 3.24 | 1.21E-03 | 1.32E-02 |
| Zfp668 | 86.18 | 1.38 | 0.43 | 3.25 | 1.14E-03 | 1.25E-02 |
| Dffb | 141.40 | 1.38 | 0.34 | 4.12 | 3.77E-05 | 6.34E-04 |
| Ciart | 154.80 | 1.38 | 0.45 | 3.10 | 1.96E-03 | 1.96E-02 |
| Plag1 | 64.46 | 1.38 | 0.44 | 3.11 | 1.87E-03 | 1.90E-02 |
| Slc33a1 | 191.97 | 1.37 | 0.31 | 4.42 | 9.76E-06 | 1.93E-04 |
| Ccdc28b | 123.70 | 1.37 | 0.36 | 3.83 | 1.30E-04 | 1.90E-03 |
| Inmt | 281.20 | 1.37 | 0.35 | 3.90 | 9.48E-05 | 1.44E-03 |
| Haus4 | 55.24 | 1.36 | 0.48 | 2.83 | 4.61E-03 | 3.93E-02 |
| Pik3r2 | 457.45 | 1.36 | 0.24 | 5.69 | 1.25E-08 | 4.11E-07 |
| Pdp2 | 841.85 | 1.35 | 0.29 | 4.59 | 4.43E-06 | 9.37E-05 |
| Glb1l | 66.09 | 1.35 | 0.44 | 3.06 | 2.19E-03 | 2.15E-02 |
| Zfp93 | 68.55 | 1.35 | 0.53 | 2.55 | 1.07E-02 | 7.67E-02 |
| Plscr4 | 246.39 | 1.35 | 0.27 | 5.04 | 4.71E-07 | 1.20E-05 |
| Fn3krp | 77.88 | 1.35 | 0.42 | 3.17 | 1.53E-03 | 1.61E-02 |
| Tef | 1689.28 | 1.34 | 0.22 | 6.19 | 5.98E-10 | 2.33E-08 |
| Tesmin | 71.29 | 1.34 | 0.47 | 2.86 | 4.19E-03 | 3.65E-02 |
| Ahr | 159.09 | 1.34 | 0.31 | 4.32 | 1.55E-05 | 2.91E-04 |
| Gask1b | 516.92 | 1.33 | 0.27 | 4.90 | 9.82E-07 | 2.34E-05 |
| Il16 | 88.47 | 1.33 | 0.41 | 3.23 | 1.26E-03 | 1.36E-02 |
| Cdc25b | 73.83 | 1.32 | 0.44 | 2.99 | 2.80E-03 | 2.61E-02 |
| Zfp961 | 79.15 | 1.32 | 0.50 | 2.63 | 8.60E-03 | 6.49E-02 |
| Heyl | 481.32 | 1.31 | 0.24 | 5.39 | 7.10E-08 | 2.08E-06 |
| Zfp952 | 94.94 | 1.31 | 0.54 | 2.43 | 1.50E-02 | 9.88E-02 |
| Mamdc4 | 86.45 | 1.31 | 0.44 | 3.01 | 2.58E-03 | 2.45E-02 |
| Rasl12 | 155.30 | 1.31 | 0.32 | 4.07 | 4.67E-05 | 7.69E-04 |
| Zfp738 | 148.08 | 1.31 | 0.42 | 3.15 | 1.65E-03 | 1.71E-02 |
| Mmp28 | 90.09 | 1.31 | 0.41 | 3.18 | 1.47E-03 | 1.56E-02 |
| 9130023H24Rik | 49.85 | 1.30 | 0.51 | 2.57 | 1.03E-02 | 7.46E-02 |
| Zfp112 | 75.34 | 1.30 | 0.46 | 2.80 | 5.09E-03 | 4.25E-02 |
| Hpgd | 105.10 | 1.28 | 0.43 | 3.00 | 2.70E-03 | 2.53E-02 |
| Patz1 | 339.56 | 1.28 | 0.29 | 4.37 | 1.25E-05 | 2.39E-04 |
| Pi16 | 442.63 | 1.28 | 0.30 | 4.24 | 2.19E-05 | 3.97E-04 |
| Aspa | 56.95 | 1.28 | 0.47 | 2.69 | 7.14E-03 | 5.55E-02 |
| Plp1 | 207.27 | 1.27 | 0.28 | 4.53 | 5.76E-06 | 1.20E-04 |
| Sertad3 | 48.88 | 1.27 | 0.50 | 2.53 | 1.15E-02 | 8.15E-02 |
| Hey1 | 143.92 | 1.27 | 0.46 | 2.74 | 6.10E-03 | 4.90E-02 |
| Sybu | 127.69 | 1.26 | 0.35 | 3.66 | 2.56E-04 | 3.44E-03 |
| Kcna1 | 54.07 | 1.26 | 0.51 | 2.47 | 1.35E-02 | 9.10E-02 |
| Zfp866 | 288.38 | 1.26 | 0.28 | 4.53 | 5.97E-06 | 1.23E-04 |
| Zfp526 | 126.38 | 1.26 | 0.38 | 3.28 | 1.04E-03 | 1.17E-02 |
| Iffo1 | 217.87 | 1.25 | 0.27 | 4.60 | 4.18E-06 | 8.90E-05 |
| Pfkfb1 | 502.88 | 1.25 | 0.31 | 4.06 | 4.93E-05 | 8.05E-04 |
| Hic1 | 196.60 | 1.25 | 0.40 | 3.13 | 1.73E-03 | 1.78E-02 |
| Golga1 | 358.92 | 1.25 | 0.30 | 4.23 | 2.37E-05 | 4.23E-04 |
| Zfp949 | 151.97 | 1.25 | 0.39 | 3.21 | 1.33E-03 | 1.44E-02 |
| Klf15 | 1157.33 | 1.24 | 0.29 | 4.35 | 1.36E-05 | 2.58E-04 |
| Inpp5b | 376.19 | 1.24 | 0.26 | 4.72 | 2.34E-06 | 5.22E-05 |
| Hemk1 | 423.27 | 1.24 | 0.24 | 5.08 | 3.75E-07 | 9.75E-06 |
| Arhgef15 | 978.02 | 1.23 | 0.24 | 5.22 | 1.80E-07 | 4.96E-06 |
| Nr2f1 | 90.86 | 1.23 | 0.40 | 3.06 | 2.19E-03 | 2.16E-02 |
| Dixdc1 | 270.05 | 1.23 | 0.40 | 3.11 | 1.84E-03 | 1.88E-02 |
| Igfbp3 | 733.60 | 1.23 | 0.28 | 4.41 | 1.05E-05 | 2.06E-04 |
| Zfp944 | 106.65 | 1.23 | 0.39 | 3.11 | 1.86E-03 | 1.89E-02 |
| Stc2 | 268.77 | 1.22 | 0.33 | 3.77 | 1.65E-04 | 2.34E-03 |
| Zfp518b | 148.50 | 1.22 | 0.40 | 3.03 | 2.48E-03 | 2.38E-02 |
| Podn | 469.00 | 1.22 | 0.30 | 4.04 | 5.40E-05 | 8.74E-04 |
| Adprm | 145.91 | 1.21 | 0.32 | 3.84 | 1.22E-04 | 1.80E-03 |
| Thap3 | 168.09 | 1.21 | 0.30 | 4.01 | 6.12E-05 | 9.79E-04 |
| Shld1 | 457.42 | 1.21 | 0.24 | 4.95 | 7.51E-07 | 1.84E-05 |
| Dipk2b | 88.79 | 1.21 | 0.44 | 2.73 | 6.34E-03 | 5.07E-02 |
| Ttf1 | 255.08 | 1.20 | 0.29 | 4.16 | 3.21E-05 | 5.55E-04 |
| Rab3il1 | 80.26 | 1.20 | 0.46 | 2.60 | 9.45E-03 | 7.00E-02 |
| Capn10 | 105.12 | 1.20 | 0.40 | 2.97 | 2.94E-03 | 2.71E-02 |
| Snx33 | 169.38 | 1.19 | 0.31 | 3.81 | 1.41E-04 | 2.05E-03 |
| Ttc30b | 273.80 | 1.19 | 0.37 | 3.19 | 1.43E-03 | 1.53E-02 |
| Slc38a1 | 141.26 | 1.19 | 0.43 | 2.80 | 5.06E-03 | 4.23E-02 |
| Zfp612 | 448.92 | 1.19 | 0.28 | 4.31 | 1.62E-05 | 3.03E-04 |
| Ccdc163 | 93.51 | 1.19 | 0.43 | 2.76 | 5.87E-03 | 4.75E-02 |
| Gm10644 | 63.79 | 1.19 | 0.47 | 2.51 | 1.22E-02 | 8.45E-02 |
| Cep97 | 146.82 | 1.19 | 0.32 | 3.72 | 2.00E-04 | 2.78E-03 |
| Zfp395 | 278.02 | 1.19 | 0.33 | 3.65 | 2.61E-04 | 3.50E-03 |
| 4833420G17Rik | 355.24 | 1.19 | 0.26 | 4.62 | 3.87E-06 | 8.33E-05 |
| Acad10 | 209.04 | 1.19 | 0.31 | 3.82 | 1.33E-04 | 1.94E-03 |
| Rassf9 | 141.30 | 1.19 | 0.37 | 3.24 | 1.20E-03 | 1.31E-02 |
| H2-T24 | 126.94 | 1.19 | 0.33 | 3.60 | 3.17E-04 | 4.18E-03 |
| Ajuba | 120.53 | 1.18 | 0.34 | 3.50 | 4.60E-04 | 5.71E-03 |
| Asb14 | 320.26 | 1.18 | 0.26 | 4.52 | 6.26E-06 | 1.29E-04 |
| C1qtnf2 | 58.90 | 1.18 | 0.47 | 2.51 | 1.22E-02 | 8.45E-02 |
| Tmem164 | 901.65 | 1.18 | 0.24 | 5.02 | 5.10E-07 | 1.29E-05 |
| Ccdc71 | 322.43 | 1.18 | 0.27 | 4.37 | 1.27E-05 | 2.43E-04 |
| Trarg1 | 54.51 | 1.17 | 0.47 | 2.49 | 1.27E-02 | 8.71E-02 |
| Gm48348 | 91.28 | 1.17 | 0.39 | 2.99 | 2.80E-03 | 2.61E-02 |
| Zfp512b | 439.32 | 1.17 | 0.28 | 4.20 | 2.64E-05 | 4.67E-04 |
| Asb7 | 163.12 | 1.17 | 0.34 | 3.42 | 6.30E-04 | 7.54E-03 |
| Cracr2b | 254.28 | 1.17 | 0.26 | 4.48 | 7.38E-06 | 1.50E-04 |
| Xndc1 | 183.34 | 1.17 | 0.33 | 3.48 | 5.04E-04 | 6.20E-03 |
| Art5 | 549.85 | 1.16 | 0.22 | 5.20 | 1.98E-07 | 5.38E-06 |
| Bmf | 142.67 | 1.16 | 0.47 | 2.44 | 1.48E-02 | 9.79E-02 |
| Ap5z1 | 230.78 | 1.16 | 0.30 | 3.92 | 9.03E-05 | 1.38E-03 |
| Chst14 | 94.18 | 1.16 | 0.38 | 3.06 | 2.22E-03 | 2.18E-02 |
| Rgma | 784.37 | 1.15 | 0.21 | 5.56 | 2.68E-08 | 8.43E-07 |
| Meox2 | 369.27 | 1.15 | 0.29 | 3.91 | 9.36E-05 | 1.43E-03 |
| Thrb | 565.50 | 1.15 | 0.41 | 2.78 | 5.41E-03 | 4.46E-02 |
| Evc | 200.08 | 1.15 | 0.30 | 3.83 | 1.28E-04 | 1.87E-03 |
| Gsta3 | 142.16 | 1.14 | 0.36 | 3.17 | 1.53E-03 | 1.60E-02 |
| Zfp799 | 133.56 | 1.14 | 0.32 | 3.61 | 3.08E-04 | 4.07E-03 |
| Bahcc1 | 448.42 | 1.14 | 0.30 | 3.80 | 1.44E-04 | 2.07E-03 |
| Tnfrsf19 | 111.78 | 1.14 | 0.43 | 2.66 | 7.81E-03 | 5.98E-02 |
| Slc25a47 | 104.93 | 1.14 | 0.39 | 2.90 | 3.78E-03 | 3.35E-02 |
| Stard9 | 121.04 | 1.14 | 0.39 | 2.88 | 4.02E-03 | 3.52E-02 |
| Zfp65 | 101.28 | 1.13 | 0.41 | 2.76 | 5.85E-03 | 4.74E-02 |
| Gal3st2c | 158.82 | 1.13 | 0.33 | 3.42 | 6.24E-04 | 7.48E-03 |
| Klhl33 | 1094.91 | 1.13 | 0.30 | 3.80 | 1.44E-04 | 2.08E-03 |
| Zfp780b | 114.90 | 1.13 | 0.36 | 3.13 | 1.73E-03 | 1.79E-02 |
| Per2 | 673.99 | 1.12 | 0.30 | 3.76 | 1.70E-04 | 2.40E-03 |
| Clcn1 | 252.00 | 1.12 | 0.30 | 3.73 | 1.94E-04 | 2.71E-03 |
| Wdr6 | 329.04 | 1.12 | 0.27 | 4.19 | 2.77E-05 | 4.88E-04 |
| Iqce | 114.84 | 1.12 | 0.37 | 3.01 | 2.64E-03 | 2.49E-02 |
| Zfp329 | 130.69 | 1.12 | 0.37 | 3.01 | 2.61E-03 | 2.48E-02 |
| Gpr182 | 115.29 | 1.12 | 0.35 | 3.18 | 1.49E-03 | 1.58E-02 |
| Tcea2 | 98.81 | 1.11 | 0.39 | 2.84 | 4.58E-03 | 3.92E-02 |
| Fam13c | 166.87 | 1.11 | 0.32 | 3.51 | 4.53E-04 | 5.65E-03 |
| Tbc1d13 | 367.51 | 1.11 | 0.28 | 3.96 | 7.45E-05 | 1.17E-03 |
| Adal | 179.71 | 1.10 | 0.30 | 3.71 | 2.07E-04 | 2.87E-03 |
| Wdr81 | 343.01 | 1.10 | 0.26 | 4.21 | 2.56E-05 | 4.54E-04 |
| Rnf26 | 76.72 | 1.10 | 0.40 | 2.79 | 5.24E-03 | 4.35E-02 |
| Gipc3 | 91.91 | 1.10 | 0.39 | 2.82 | 4.81E-03 | 4.06E-02 |
| Acbd4 | 304.20 | 1.10 | 0.28 | 3.93 | 8.52E-05 | 1.31E-03 |
| Cxcl12 | 2403.99 | 1.10 | 0.21 | 5.22 | 1.83E-07 | 5.02E-06 |
| Cbx6 | 635.75 | 1.09 | 0.21 | 5.12 | 3.04E-07 | 8.03E-06 |
| Chd6 | 1746.77 | 1.09 | 0.18 | 6.06 | 1.36E-09 | 4.99E-08 |
| Thsd1 | 100.77 | 1.09 | 0.42 | 2.60 | 9.29E-03 | 6.91E-02 |
| Zfp523 | 142.09 | 1.08 | 0.39 | 2.80 | 5.06E-03 | 4.23E-02 |
| Abca8a | 2282.23 | 1.08 | 0.22 | 4.95 | 7.47E-07 | 1.83E-05 |
| Tlcd5 | 187.22 | 1.08 | 0.35 | 3.08 | 2.07E-03 | 2.06E-02 |
| 9430015G10Rik | 92.62 | 1.08 | 0.37 | 2.93 | 3.37E-03 | 3.04E-02 |
| Zfp74 | 118.51 | 1.08 | 0.40 | 2.70 | 6.88E-03 | 5.40E-02 |
| Tchp | 172.08 | 1.08 | 0.30 | 3.60 | 3.24E-04 | 4.25E-03 |
| Pcx | 159.80 | 1.08 | 0.41 | 2.64 | 8.22E-03 | 6.25E-02 |
| Zfp120 | 160.62 | 1.07 | 0.35 | 3.08 | 2.07E-03 | 2.06E-02 |
| Rtp3 | 70.77 | 1.07 | 0.42 | 2.57 | 1.03E-02 | 7.46E-02 |
| Kmt5c | 250.68 | 1.07 | 0.27 | 4.01 | 5.96E-05 | 9.57E-04 |
| Cpt1c | 87.93 | 1.07 | 0.40 | 2.69 | 7.10E-03 | 5.53E-02 |
| Adamts7 | 884.08 | 1.07 | 0.26 | 4.14 | 3.50E-05 | 5.97E-04 |
| Sertad4 | 78.97 | 1.07 | 0.43 | 2.50 | 1.25E-02 | 8.63E-02 |
| Rasgrp2 | 752.15 | 1.06 | 0.24 | 4.39 | 1.11E-05 | 2.17E-04 |
| Pth1r | 117.63 | 1.06 | 0.36 | 2.97 | 2.94E-03 | 2.71E-02 |
| Lysmd4 | 251.96 | 1.06 | 0.32 | 3.33 | 8.79E-04 | 1.01E-02 |
| 2210407C18Rik | 207.61 | 1.06 | 0.35 | 3.04 | 2.37E-03 | 2.30E-02 |
| Rnf219 | 68.94 | 1.06 | 0.44 | 2.43 | 1.52E-02 | 9.94E-02 |
| Adcy6 | 6971.13 | 1.06 | 0.20 | 5.27 | 1.39E-07 | 3.87E-06 |
| Adcyap1r1 | 165.68 | 1.06 | 0.34 | 3.09 | 2.03E-03 | 2.03E-02 |
| Ky | 295.59 | 1.06 | 0.33 | 3.23 | 1.23E-03 | 1.34E-02 |
| Adamts10 | 761.28 | 1.05 | 0.22 | 4.76 | 1.96E-06 | 4.42E-05 |
| Sema6c | 204.53 | 1.05 | 0.32 | 3.27 | 1.07E-03 | 1.19E-02 |
| Dpysl2 | 304.73 | 1.05 | 0.28 | 3.69 | 2.22E-04 | 3.04E-03 |
| Setd1b | 612.57 | 1.05 | 0.25 | 4.13 | 3.70E-05 | 6.24E-04 |
| Pcdh18 | 102.78 | 1.05 | 0.40 | 2.65 | 8.00E-03 | 6.10E-02 |
| Rbm10 | 556.63 | 1.05 | 0.21 | 4.90 | 9.51E-07 | 2.27E-05 |
| Smtnl2 | 263.84 | 1.04 | 0.26 | 4.04 | 5.29E-05 | 8.58E-04 |
| Krba1 | 824.90 | 1.04 | 0.23 | 4.62 | 3.78E-06 | 8.15E-05 |
| Zfp579 | 214.55 | 1.04 | 0.31 | 3.40 | 6.65E-04 | 7.89E-03 |
| Zfp933 | 197.86 | 1.04 | 0.33 | 3.11 | 1.87E-03 | 1.90E-02 |
| Slc26a10 | 668.90 | 1.04 | 0.27 | 3.86 | 1.14E-04 | 1.70E-03 |
| Dclre1a | 179.68 | 1.04 | 0.36 | 2.85 | 4.33E-03 | 3.74E-02 |
| Map3k12 | 112.00 | 1.04 | 0.36 | 2.90 | 3.69E-03 | 3.27E-02 |
| Zfp189 | 89.29 | 1.03 | 0.42 | 2.45 | 1.44E-02 | 9.58E-02 |
| Zfp788 | 173.26 | 1.03 | 0.31 | 3.37 | 7.41E-04 | 8.68E-03 |
| Bcl9 | 801.51 | 1.03 | 0.29 | 3.58 | 3.38E-04 | 4.40E-03 |
| Rbak | 102.78 | 1.03 | 0.38 | 2.68 | 7.44E-03 | 5.74E-02 |
| Cdk20 | 83.61 | 1.03 | 0.39 | 2.60 | 9.32E-03 | 6.93E-02 |
| Rap1gap | 413.35 | 1.03 | 0.24 | 4.21 | 2.51E-05 | 4.45E-04 |
| Gm5113 | 264.47 | 1.02 | 0.27 | 3.78 | 1.58E-04 | 2.25E-03 |
| Adck5 | 101.97 | 1.02 | 0.36 | 2.83 | 4.72E-03 | 4.00E-02 |
| Fbf1 | 442.48 | 1.01 | 0.24 | 4.15 | 3.35E-05 | 5.76E-04 |
| Htra3 | 2286.56 | 1.01 | 0.19 | 5.22 | 1.82E-07 | 5.00E-06 |
| Golga2 | 177.12 | 1.01 | 0.33 | 3.08 | 2.09E-03 | 2.08E-02 |
| Zfp950 | 355.69 | 1.01 | 0.29 | 3.44 | 5.78E-04 | 7.01E-03 |
| C1qtnf1 | 350.46 | 1.01 | 0.26 | 3.90 | 9.49E-05 | 1.44E-03 |
| Zfp446 | 179.24 | 1.00 | 0.29 | 3.50 | 4.72E-04 | 5.85E-03 |
| Zfp646 | 392.18 | 1.00 | 0.30 | 3.36 | 7.89E-04 | 9.18E-03 |
| Mcf2l | 1155.10 | 1.00 | 0.21 | 4.83 | 1.37E-06 | 3.15E-05 |
| Ccdc102a | 101.47 | 1.00 | 0.35 | 2.84 | 4.55E-03 | 3.90E-02 |
| Mtmr9 | 305.60 | 1.00 | 0.28 | 3.60 | 3.24E-04 | 4.25E-03 |
| Cbx7 | 538.52 | 0.99 | 0.24 | 4.10 | 4.08E-05 | 6.78E-04 |
| Zfp113 | 94.92 | 0.98 | 0.38 | 2.60 | 9.45E-03 | 7.00E-02 |
| Map10 | 111.21 | 0.98 | 0.36 | 2.74 | 6.21E-03 | 4.97E-02 |
| Pla2g4e | 169.00 | 0.98 | 0.35 | 2.83 | 4.64E-03 | 3.96E-02 |
| Fbxl12 | 249.80 | 0.98 | 0.26 | 3.80 | 1.45E-04 | 2.09E-03 |
| Tgfbr3l | 77.24 | 0.98 | 0.40 | 2.43 | 1.50E-02 | 9.88E-02 |
| Zfp3 | 89.26 | 0.98 | 0.40 | 2.47 | 1.34E-02 | 9.09E-02 |
| Tspan13 | 707.43 | 0.98 | 0.21 | 4.57 | 4.98E-06 | 1.04E-04 |
| Bcl9l | 1094.29 | 0.98 | 0.23 | 4.22 | 2.40E-05 | 4.27E-04 |
| Exoc8 | 287.46 | 0.97 | 0.32 | 3.08 | 2.04E-03 | 2.03E-02 |
| Taok2 | 1157.34 | 0.97 | 0.19 | 5.25 | 1.51E-07 | 4.18E-06 |
| Ahnak2 | 360.09 | 0.97 | 0.28 | 3.44 | 5.73E-04 | 6.96E-03 |
| B3galt2 | 193.30 | 0.97 | 0.32 | 3.08 | 2.08E-03 | 2.07E-02 |
| Hey2 | 164.45 | 0.97 | 0.33 | 2.98 | 2.88E-03 | 2.67E-02 |
| Syde2 | 645.31 | 0.97 | 0.27 | 3.62 | 2.97E-04 | 3.95E-03 |
| Gucy1a1 | 411.03 | 0.97 | 0.23 | 4.22 | 2.49E-05 | 4.42E-04 |
| Recql5 | 243.12 | 0.97 | 0.29 | 3.28 | 1.05E-03 | 1.17E-02 |
| Lims2 | 1011.32 | 0.96 | 0.27 | 3.59 | 3.31E-04 | 4.33E-03 |
| Hsf4 | 98.03 | 0.96 | 0.37 | 2.57 | 1.03E-02 | 7.46E-02 |
| Grap | 161.06 | 0.96 | 0.33 | 2.87 | 4.11E-03 | 3.60E-02 |
| Kank3 | 792.73 | 0.96 | 0.27 | 3.58 | 3.40E-04 | 4.42E-03 |
| Card10 | 612.38 | 0.96 | 0.24 | 4.06 | 4.84E-05 | 7.93E-04 |
| Pm20d1 | 387.84 | 0.96 | 0.25 | 3.81 | 1.37E-04 | 1.99E-03 |
| Fmo1 | 795.16 | 0.96 | 0.22 | 4.30 | 1.69E-05 | 3.15E-04 |
| Dnal1 | 185.87 | 0.96 | 0.31 | 3.10 | 1.96E-03 | 1.96E-02 |
| Cngb3 | 130.03 | 0.95 | 0.39 | 2.48 | 1.32E-02 | 9.01E-02 |
| Adamts6 | 113.00 | 0.95 | 0.38 | 2.51 | 1.20E-02 | 8.38E-02 |
| Snrk | 2174.32 | 0.95 | 0.17 | 5.55 | 2.88E-08 | 9.04E-07 |
| Afdn | 1737.88 | 0.95 | 0.20 | 4.66 | 3.22E-06 | 6.99E-05 |
| A130010J15Rik | 103.09 | 0.95 | 0.38 | 2.48 | 1.30E-02 | 8.85E-02 |
| Caskin2 | 1440.27 | 0.95 | 0.18 | 5.28 | 1.31E-07 | 3.67E-06 |
| Zscan12 | 107.29 | 0.95 | 0.38 | 2.47 | 1.34E-02 | 9.08E-02 |
| Ston1 | 157.02 | 0.95 | 0.34 | 2.79 | 5.29E-03 | 4.39E-02 |
| Zfp629 | 592.12 | 0.94 | 0.28 | 3.41 | 6.54E-04 | 7.78E-03 |
| Zfp687 | 308.93 | 0.94 | 0.37 | 2.56 | 1.05E-02 | 7.57E-02 |
| Abca6 | 249.69 | 0.94 | 0.33 | 2.85 | 4.34E-03 | 3.75E-02 |
| 2610021A01Rik | 183.41 | 0.94 | 0.34 | 2.74 | 6.19E-03 | 4.96E-02 |
| Fam219b | 255.81 | 0.94 | 0.30 | 3.19 | 1.40E-03 | 1.50E-02 |
| Zfyve19 | 168.65 | 0.94 | 0.29 | 3.19 | 1.40E-03 | 1.50E-02 |
| Rhobtb2 | 531.45 | 0.94 | 0.26 | 3.60 | 3.19E-04 | 4.20E-03 |
| Fmo2 | 783.66 | 0.94 | 0.33 | 2.86 | 4.23E-03 | 3.67E-02 |
| Mxra8 | 745.74 | 0.94 | 0.24 | 3.87 | 1.11E-04 | 1.65E-03 |
| Slc25a29 | 164.27 | 0.94 | 0.31 | 3.02 | 2.53E-03 | 2.42E-02 |
| Ppara | 669.00 | 0.93 | 0.29 | 3.16 | 1.60E-03 | 1.66E-02 |
| Cep250 | 373.98 | 0.93 | 0.26 | 3.58 | 3.48E-04 | 4.51E-03 |
| Slc46a3 | 208.20 | 0.93 | 0.27 | 3.38 | 7.13E-04 | 8.40E-03 |
| Crebl2 | 325.42 | 0.92 | 0.27 | 3.45 | 5.69E-04 | 6.93E-03 |
| 2010315B03Rik | 164.33 | 0.92 | 0.34 | 2.73 | 6.40E-03 | 5.10E-02 |
| Prr12 | 555.99 | 0.92 | 0.21 | 4.38 | 1.21E-05 | 2.34E-04 |
| Kcnh2 | 2191.01 | 0.92 | 0.17 | 5.43 | 5.76E-08 | 1.71E-06 |
| Kdm6a | 615.64 | 0.92 | 0.26 | 3.50 | 4.59E-04 | 5.71E-03 |
| Atxn7l2 | 122.22 | 0.92 | 0.36 | 2.58 | 1.00E-02 | 7.32E-02 |
| Foxo4 | 1365.53 | 0.91 | 0.17 | 5.34 | 9.07E-08 | 2.60E-06 |
| Thbs3 | 312.93 | 0.91 | 0.25 | 3.71 | 2.09E-04 | 2.89E-03 |
| Phf12 | 653.08 | 0.91 | 0.23 | 3.92 | 9.03E-05 | 1.38E-03 |
| Rnf207 | 2221.29 | 0.91 | 0.18 | 5.17 | 2.31E-07 | 6.22E-06 |
| G0s2 | 600.09 | 0.91 | 0.27 | 3.39 | 6.91E-04 | 8.16E-03 |
| Sfxn5 | 110.99 | 0.91 | 0.36 | 2.53 | 1.15E-02 | 8.17E-02 |
| Pot1a | 234.89 | 0.91 | 0.26 | 3.49 | 4.75E-04 | 5.88E-03 |
| Sephs1 | 287.47 | 0.90 | 0.30 | 3.02 | 2.55E-03 | 2.43E-02 |
| Ndor1 | 300.09 | 0.90 | 0.28 | 3.23 | 1.24E-03 | 1.35E-02 |
| Lgals4 | 1638.01 | 0.90 | 0.24 | 3.70 | 2.14E-04 | 2.95E-03 |
| Ncam1 | 187.99 | 0.90 | 0.37 | 2.45 | 1.44E-02 | 9.59E-02 |
| Six5 | 300.24 | 0.90 | 0.24 | 3.80 | 1.47E-04 | 2.11E-03 |
| Pgpep1 | 531.23 | 0.90 | 0.24 | 3.75 | 1.76E-04 | 2.48E-03 |
| Tcf15 | 300.44 | 0.90 | 0.37 | 2.44 | 1.48E-02 | 9.79E-02 |
| Slc27a1 | 6616.45 | 0.90 | 0.17 | 5.41 | 6.35E-08 | 1.87E-06 |
| Tada2a | 170.21 | 0.89 | 0.30 | 3.00 | 2.69E-03 | 2.53E-02 |
| Klf13 | 1330.24 | 0.89 | 0.18 | 4.91 | 9.27E-07 | 2.23E-05 |
| Fat4 | 222.99 | 0.89 | 0.30 | 2.99 | 2.76E-03 | 2.58E-02 |
| Fbxo9 | 525.96 | 0.89 | 0.24 | 3.77 | 1.62E-04 | 2.31E-03 |
| Abhd4 | 319.28 | 0.88 | 0.27 | 3.31 | 9.41E-04 | 1.07E-02 |
| Fan1 | 128.07 | 0.88 | 0.35 | 2.51 | 1.20E-02 | 8.40E-02 |
| Cnot9 | 261.20 | 0.88 | 0.26 | 3.33 | 8.73E-04 | 1.00E-02 |
| Pex1 | 283.89 | 0.88 | 0.29 | 3.03 | 2.44E-03 | 2.35E-02 |
| 3425401B19Rik | 2508.81 | 0.88 | 0.34 | 2.61 | 9.04E-03 | 6.76E-02 |
| Pcolce2 | 486.96 | 0.88 | 0.26 | 3.42 | 6.34E-04 | 7.57E-03 |
| Hand2 | 973.76 | 0.88 | 0.28 | 3.10 | 1.93E-03 | 1.95E-02 |
| Pwwp3a | 384.48 | 0.88 | 0.26 | 3.42 | 6.35E-04 | 7.57E-03 |
| Zfp641 | 147.45 | 0.88 | 0.35 | 2.53 | 1.13E-02 | 8.03E-02 |
| Cdh19 | 201.86 | 0.88 | 0.36 | 2.43 | 1.49E-02 | 9.87E-02 |
| Casp9 | 217.42 | 0.87 | 0.31 | 2.79 | 5.20E-03 | 4.33E-02 |
| Pcmtd2 | 1374.52 | 0.87 | 0.26 | 3.31 | 9.42E-04 | 1.07E-02 |
| Tubgcp5 | 218.53 | 0.87 | 0.31 | 2.83 | 4.65E-03 | 3.96E-02 |
| Gja4 | 194.06 | 0.87 | 0.34 | 2.56 | 1.05E-02 | 7.58E-02 |
| Usp11 | 216.21 | 0.87 | 0.29 | 3.03 | 2.45E-03 | 2.35E-02 |
| Spta1 | 584.67 | 0.87 | 0.21 | 4.15 | 3.36E-05 | 5.77E-04 |
| Cep131 | 241.52 | 0.86 | 0.26 | 3.38 | 7.14E-04 | 8.40E-03 |
| Rbm20 | 1218.33 | 0.86 | 0.24 | 3.54 | 3.93E-04 | 4.99E-03 |
| Adh1 | 176.89 | 0.86 | 0.35 | 2.45 | 1.41E-02 | 9.42E-02 |
| Stx2 | 291.26 | 0.86 | 0.24 | 3.57 | 3.52E-04 | 4.55E-03 |
| Pla2g15 | 166.38 | 0.86 | 0.29 | 2.99 | 2.80E-03 | 2.61E-02 |
| Prickle3 | 511.41 | 0.86 | 0.23 | 3.78 | 1.57E-04 | 2.24E-03 |
| Lrrc61 | 233.28 | 0.86 | 0.29 | 2.95 | 3.16E-03 | 2.87E-02 |
| Kcnj8 | 706.36 | 0.86 | 0.19 | 4.40 | 1.10E-05 | 2.16E-04 |
| Zscan20 | 138.38 | 0.85 | 0.35 | 2.46 | 1.40E-02 | 9.37E-02 |
| Syde1 | 386.80 | 0.85 | 0.26 | 3.27 | 1.07E-03 | 1.20E-02 |
| Pfas | 587.80 | 0.85 | 0.21 | 4.02 | 5.81E-05 | 9.35E-04 |
| Rab29 | 112.61 | 0.85 | 0.34 | 2.51 | 1.19E-02 | 8.35E-02 |
| Lrrc14 | 211.01 | 0.85 | 0.28 | 3.07 | 2.14E-03 | 2.11E-02 |
| Ankzf1 | 400.19 | 0.85 | 0.22 | 3.88 | 1.06E-04 | 1.59E-03 |
| Nr2c2 | 656.69 | 0.85 | 0.26 | 3.28 | 1.04E-03 | 1.17E-02 |
| Bcl7a | 326.46 | 0.85 | 0.27 | 3.20 | 1.40E-03 | 1.50E-02 |
| Pik3c2b | 742.93 | 0.85 | 0.23 | 3.68 | 2.35E-04 | 3.19E-03 |
| Prox1 | 1254.82 | 0.84 | 0.28 | 2.97 | 2.98E-03 | 2.73E-02 |
| Letm2 | 175.44 | 0.84 | 0.32 | 2.62 | 8.73E-03 | 6.58E-02 |
| Nfic | 2889.91 | 0.84 | 0.17 | 5.04 | 4.62E-07 | 1.18E-05 |
| Uvssa | 227.83 | 0.84 | 0.33 | 2.57 | 1.01E-02 | 7.39E-02 |
| Anpep | 316.20 | 0.84 | 0.32 | 2.66 | 7.84E-03 | 6.00E-02 |
| Mrm1 | 367.10 | 0.84 | 0.24 | 3.45 | 5.69E-04 | 6.93E-03 |
| Ska2 | 189.89 | 0.84 | 0.34 | 2.44 | 1.46E-02 | 9.69E-02 |
| Zfp362 | 217.51 | 0.84 | 0.31 | 2.67 | 7.63E-03 | 5.87E-02 |
| Ppp1r3b | 547.75 | 0.84 | 0.25 | 3.36 | 7.80E-04 | 9.08E-03 |
| Tmem18 | 233.43 | 0.84 | 0.30 | 2.82 | 4.75E-03 | 4.02E-02 |
| Nfyc | 1036.26 | 0.83 | 0.21 | 4.07 | 4.73E-05 | 7.77E-04 |
| Rrnad1 | 220.69 | 0.83 | 0.28 | 2.99 | 2.77E-03 | 2.59E-02 |
| Zfp507 | 286.35 | 0.83 | 0.25 | 3.28 | 1.04E-03 | 1.16E-02 |
| Rgp1 | 1057.23 | 0.83 | 0.21 | 3.95 | 7.66E-05 | 1.19E-03 |
| Ccbe1 | 277.83 | 0.83 | 0.34 | 2.46 | 1.38E-02 | 9.31E-02 |
| Cygb | 1195.35 | 0.83 | 0.20 | 4.11 | 3.99E-05 | 6.67E-04 |
| Nrbp2 | 711.63 | 0.83 | 0.24 | 3.52 | 4.32E-04 | 5.42E-03 |
| Mtx3 | 312.69 | 0.83 | 0.27 | 3.06 | 2.23E-03 | 2.19E-02 |
| Ephx1 | 899.00 | 0.83 | 0.33 | 2.50 | 1.23E-02 | 8.49E-02 |
| Nfatc4 | 214.57 | 0.83 | 0.31 | 2.64 | 8.35E-03 | 6.34E-02 |
| Etv1 | 355.08 | 0.83 | 0.30 | 2.77 | 5.54E-03 | 4.55E-02 |
| Dcbld1 | 117.53 | 0.82 | 0.34 | 2.43 | 1.49E-02 | 9.88E-02 |
| Slc2a4rg-ps | 147.15 | 0.82 | 0.30 | 2.71 | 6.77E-03 | 5.32E-02 |
| Zmym3 | 542.67 | 0.82 | 0.29 | 2.84 | 4.56E-03 | 3.91E-02 |
| Ctns | 192.14 | 0.82 | 0.30 | 2.72 | 6.54E-03 | 5.18E-02 |
| Carns1 | 1284.03 | 0.82 | 0.21 | 3.89 | 9.84E-05 | 1.49E-03 |
| Zfp715 | 468.84 | 0.81 | 0.22 | 3.67 | 2.45E-04 | 3.31E-03 |
| Lrrc45 | 238.08 | 0.81 | 0.26 | 3.10 | 1.95E-03 | 1.96E-02 |
| Zfp839 | 315.47 | 0.81 | 0.32 | 2.58 | 9.91E-03 | 7.25E-02 |
| Cnpy2 | 431.54 | 0.81 | 0.23 | 3.45 | 5.64E-04 | 6.89E-03 |
| Lum | 1701.40 | 0.81 | 0.28 | 2.94 | 3.29E-03 | 2.98E-02 |
| Cep295 | 217.73 | 0.81 | 0.32 | 2.54 | 1.09E-02 | 7.86E-02 |
| 0610030E20Rik | 280.65 | 0.81 | 0.26 | 3.15 | 1.66E-03 | 1.72E-02 |
| Col14a1 | 676.88 | 0.81 | 0.24 | 3.35 | 7.96E-04 | 9.22E-03 |
| Zfp825 | 157.87 | 0.81 | 0.32 | 2.53 | 1.14E-02 | 8.12E-02 |
| Cc2d2a | 347.24 | 0.81 | 0.27 | 2.96 | 3.11E-03 | 2.84E-02 |
| Ypel3 | 955.34 | 0.81 | 0.18 | 4.39 | 1.14E-05 | 2.21E-04 |
| Cdkn2c | 197.57 | 0.80 | 0.28 | 2.86 | 4.24E-03 | 3.68E-02 |
| Zfp366 | 338.12 | 0.80 | 0.28 | 2.82 | 4.73E-03 | 4.02E-02 |
| Fam217b | 269.56 | 0.80 | 0.31 | 2.62 | 8.70E-03 | 6.56E-02 |
| Ranbp6 | 303.69 | 0.80 | 0.26 | 3.04 | 2.39E-03 | 2.31E-02 |
| Septin8 | 1233.33 | 0.80 | 0.18 | 4.38 | 1.21E-05 | 2.33E-04 |
| Inka2 | 511.35 | 0.80 | 0.25 | 3.20 | 1.36E-03 | 1.46E-02 |
| L3mbtl3 | 208.31 | 0.80 | 0.32 | 2.48 | 1.33E-02 | 9.03E-02 |
| Miip | 234.07 | 0.80 | 0.31 | 2.53 | 1.16E-02 | 8.17E-02 |
| Zfp292 | 460.45 | 0.80 | 0.29 | 2.73 | 6.42E-03 | 5.11E-02 |
| Shprh | 296.27 | 0.79 | 0.32 | 2.49 | 1.26E-02 | 8.69E-02 |
| Adamtsl4 | 1677.19 | 0.79 | 0.19 | 4.15 | 3.34E-05 | 5.74E-04 |
| Wdr24 | 257.81 | 0.79 | 0.25 | 3.12 | 1.81E-03 | 1.85E-02 |
| Nf2 | 2403.35 | 0.79 | 0.17 | 4.60 | 4.23E-06 | 8.99E-05 |
| Plekhh3 | 352.77 | 0.79 | 0.26 | 3.04 | 2.40E-03 | 2.32E-02 |
| Plekha6 | 3121.12 | 0.79 | 0.20 | 3.86 | 1.14E-04 | 1.70E-03 |
| Zfp319 | 280.30 | 0.79 | 0.25 | 3.21 | 1.34E-03 | 1.44E-02 |
| Tmpo | 897.85 | 0.79 | 0.24 | 3.32 | 8.90E-04 | 1.02E-02 |
| Gigyf1 | 1118.82 | 0.79 | 0.22 | 3.50 | 4.58E-04 | 5.70E-03 |
| Tle2 | 332.87 | 0.78 | 0.23 | 3.43 | 6.07E-04 | 7.30E-03 |
| Pnisr | 1467.34 | 0.78 | 0.22 | 3.50 | 4.59E-04 | 5.70E-03 |
| St8sia4 | 218.54 | 0.78 | 0.31 | 2.51 | 1.22E-02 | 8.45E-02 |
| Kcnj12 | 516.62 | 0.78 | 0.23 | 3.45 | 5.57E-04 | 6.82E-03 |
| Gm49396 | 191.47 | 0.78 | 0.28 | 2.79 | 5.31E-03 | 4.40E-02 |
| Lrrc14b | 1733.26 | 0.78 | 0.24 | 3.21 | 1.33E-03 | 1.43E-02 |
| Zfp740 | 929.13 | 0.78 | 0.21 | 3.71 | 2.05E-04 | 2.85E-03 |
| Trim65 | 176.58 | 0.78 | 0.29 | 2.65 | 8.11E-03 | 6.18E-02 |
| Plcg1 | 1660.54 | 0.78 | 0.19 | 3.98 | 6.90E-05 | 1.09E-03 |
| Zfp316 | 220.53 | 0.77 | 0.27 | 2.91 | 3.65E-03 | 3.24E-02 |
| Tmem44 | 241.10 | 0.77 | 0.30 | 2.62 | 8.78E-03 | 6.60E-02 |
| Ushbp1 | 1452.04 | 0.77 | 0.18 | 4.41 | 1.05E-05 | 2.06E-04 |
| Tbc1d17 | 719.11 | 0.77 | 0.20 | 3.91 | 9.25E-05 | 1.41E-03 |
| Usp2 | 2827.98 | 0.77 | 0.25 | 3.13 | 1.76E-03 | 1.80E-02 |
| Gatd1 | 176.23 | 0.77 | 0.30 | 2.55 | 1.07E-02 | 7.70E-02 |
| Kcnj3 | 2408.08 | 0.77 | 0.22 | 3.43 | 5.95E-04 | 7.19E-03 |
| Gba2 | 426.92 | 0.76 | 0.22 | 3.45 | 5.71E-04 | 6.94E-03 |
| Hmgcs2 | 700.09 | 0.76 | 0.26 | 2.98 | 2.93E-03 | 2.70E-02 |
| Clasrp | 601.37 | 0.76 | 0.23 | 3.30 | 9.59E-04 | 1.08E-02 |
| Lrsam1 | 343.25 | 0.76 | 0.25 | 3.04 | 2.36E-03 | 2.29E-02 |
| Exoc1 | 359.08 | 0.76 | 0.27 | 2.81 | 4.99E-03 | 4.19E-02 |
| Rcor3 | 538.00 | 0.76 | 0.27 | 2.81 | 5.02E-03 | 4.20E-02 |
| Ap4m1 | 271.00 | 0.76 | 0.29 | 2.60 | 9.21E-03 | 6.87E-02 |
| Cbfa2t3 | 555.57 | 0.76 | 0.20 | 3.70 | 2.16E-04 | 2.97E-03 |
| Pdzd2 | 2420.87 | 0.76 | 0.21 | 3.65 | 2.59E-04 | 3.49E-03 |
| Rtkn | 162.80 | 0.75 | 0.30 | 2.52 | 1.16E-02 | 8.18E-02 |
| Rab6b | 392.35 | 0.75 | 0.22 | 3.35 | 7.94E-04 | 9.21E-03 |
| Sema6a | 350.53 | 0.75 | 0.29 | 2.60 | 9.38E-03 | 6.97E-02 |
| 2310011J03Rik | 300.81 | 0.75 | 0.25 | 3.05 | 2.27E-03 | 2.22E-02 |
| Hdac7 | 1202.98 | 0.75 | 0.29 | 2.57 | 1.01E-02 | 7.35E-02 |
| Inpp5e | 603.34 | 0.74 | 0.26 | 2.91 | 3.58E-03 | 3.19E-02 |
| Btbd3 | 417.38 | 0.74 | 0.24 | 3.13 | 1.77E-03 | 1.82E-02 |
| Foxk1 | 572.14 | 0.74 | 0.21 | 3.60 | 3.24E-04 | 4.25E-03 |
| Adamtsl3 | 275.93 | 0.74 | 0.29 | 2.58 | 9.90E-03 | 7.25E-02 |
| Gpr157 | 1196.23 | 0.74 | 0.22 | 3.38 | 7.26E-04 | 8.52E-03 |
| Rere | 1554.40 | 0.74 | 0.21 | 3.53 | 4.08E-04 | 5.16E-03 |
| Ago1 | 439.69 | 0.74 | 0.29 | 2.57 | 1.02E-02 | 7.42E-02 |
| Pomgnt2 | 295.33 | 0.73 | 0.27 | 2.73 | 6.26E-03 | 5.01E-02 |
| C1qtnf9 | 596.94 | 0.73 | 0.24 | 3.00 | 2.66E-03 | 2.51E-02 |
| Usf1 | 388.55 | 0.73 | 0.28 | 2.59 | 9.56E-03 | 7.06E-02 |
| Maf1 | 1643.92 | 0.73 | 0.19 | 3.91 | 9.35E-05 | 1.43E-03 |
| Slc38a7 | 333.95 | 0.73 | 0.26 | 2.81 | 5.02E-03 | 4.20E-02 |
| Tmem86a | 357.97 | 0.73 | 0.29 | 2.47 | 1.34E-02 | 9.08E-02 |
| Zscan26 | 887.72 | 0.73 | 0.22 | 3.26 | 1.12E-03 | 1.24E-02 |
| Trim11 | 389.53 | 0.73 | 0.24 | 3.07 | 2.11E-03 | 2.09E-02 |
| 2610008E11Rik | 396.81 | 0.73 | 0.26 | 2.78 | 5.36E-03 | 4.43E-02 |
| Zfp24 | 609.42 | 0.72 | 0.22 | 3.25 | 1.14E-03 | 1.26E-02 |
| Ermard | 260.33 | 0.72 | 0.26 | 2.83 | 4.71E-03 | 4.00E-02 |
| Zfp637 | 220.03 | 0.72 | 0.27 | 2.72 | 6.48E-03 | 5.14E-02 |
| Cavin2 | 3134.14 | 0.72 | 0.20 | 3.54 | 4.00E-04 | 5.07E-03 |
| Erbb3 | 318.69 | 0.72 | 0.25 | 2.90 | 3.76E-03 | 3.33E-02 |
| Sbk1 | 2382.28 | 0.72 | 0.22 | 3.19 | 1.41E-03 | 1.51E-02 |
| Arhgef25 | 382.89 | 0.72 | 0.24 | 3.02 | 2.57E-03 | 2.44E-02 |
| Mrtfb | 801.98 | 0.72 | 0.21 | 3.37 | 7.56E-04 | 8.83E-03 |
| Lgi4 | 207.58 | 0.72 | 0.29 | 2.47 | 1.34E-02 | 9.07E-02 |
| Plxnb1 | 1741.57 | 0.72 | 0.24 | 2.93 | 3.44E-03 | 3.08E-02 |
| Gga3 | 468.64 | 0.71 | 0.25 | 2.83 | 4.61E-03 | 3.93E-02 |
| Il15ra | 510.01 | 0.71 | 0.21 | 3.48 | 5.04E-04 | 6.20E-03 |
| Zfp27 | 201.16 | 0.71 | 0.29 | 2.46 | 1.39E-02 | 9.32E-02 |
| Cnnm3 | 430.14 | 0.71 | 0.26 | 2.71 | 6.72E-03 | 5.30E-02 |
| Abtb1 | 503.11 | 0.71 | 0.21 | 3.37 | 7.42E-04 | 8.68E-03 |
| Mtmr10 | 417.29 | 0.71 | 0.29 | 2.46 | 1.38E-02 | 9.30E-02 |
| Tmem104 | 261.01 | 0.71 | 0.25 | 2.79 | 5.23E-03 | 4.34E-02 |
| Maz | 1074.69 | 0.71 | 0.18 | 3.97 | 7.07E-05 | 1.11E-03 |
| Ccdc9b | 990.58 | 0.71 | 0.20 | 3.53 | 4.11E-04 | 5.18E-03 |
| Zer1 | 917.06 | 0.71 | 0.22 | 3.29 | 1.01E-03 | 1.14E-02 |
| Cers4 | 1284.24 | 0.71 | 0.19 | 3.75 | 1.76E-04 | 2.48E-03 |
| Ankrd16 | 312.01 | 0.71 | 0.25 | 2.88 | 4.02E-03 | 3.52E-02 |
| Hdac9 | 484.75 | 0.70 | 0.22 | 3.15 | 1.65E-03 | 1.71E-02 |
| Dennd6a | 404.61 | 0.70 | 0.24 | 2.95 | 3.22E-03 | 2.92E-02 |
| Rsrp1 | 5112.03 | 0.70 | 0.17 | 4.24 | 2.24E-05 | 4.05E-04 |
| Nsun5 | 239.74 | 0.70 | 0.27 | 2.57 | 1.02E-02 | 7.41E-02 |
| Kcnj5 | 1619.23 | 0.70 | 0.17 | 4.10 | 4.15E-05 | 6.90E-04 |
| Mmd | 507.04 | 0.70 | 0.24 | 2.98 | 2.92E-03 | 2.69E-02 |
| Klhl3 | 209.97 | 0.70 | 0.28 | 2.49 | 1.28E-02 | 8.79E-02 |
| Zfp317 | 431.31 | 0.70 | 0.22 | 3.13 | 1.74E-03 | 1.79E-02 |
| Ring1 | 487.43 | 0.70 | 0.21 | 3.35 | 8.05E-04 | 9.31E-03 |
| Ttc5 | 228.84 | 0.70 | 0.28 | 2.46 | 1.38E-02 | 9.27E-02 |
| Usp54 | 867.02 | 0.70 | 0.26 | 2.70 | 6.86E-03 | 5.39E-02 |
| Mib2 | 790.90 | 0.69 | 0.20 | 3.53 | 4.23E-04 | 5.31E-03 |
| Slc5a6 | 363.07 | 0.69 | 0.25 | 2.80 | 5.14E-03 | 4.28E-02 |
| Rbck1 | 941.61 | 0.69 | 0.19 | 3.69 | 2.27E-04 | 3.11E-03 |
| Dgkz | 1768.95 | 0.69 | 0.17 | 3.95 | 7.69E-05 | 1.20E-03 |
| Angptl2 | 1929.36 | 0.69 | 0.20 | 3.47 | 5.15E-04 | 6.33E-03 |
| Ciz1 | 790.65 | 0.69 | 0.20 | 3.51 | 4.43E-04 | 5.54E-03 |
| Cd300lg | 1679.88 | 0.69 | 0.22 | 3.12 | 1.78E-03 | 1.82E-02 |
| Tmem184c | 232.25 | 0.69 | 0.27 | 2.51 | 1.21E-02 | 8.42E-02 |
| Speg | 8569.42 | 0.68 | 0.16 | 4.29 | 1.76E-05 | 3.27E-04 |
| Mapk1ip1 | 539.17 | 0.68 | 0.25 | 2.73 | 6.36E-03 | 5.08E-02 |
| Lsp1 | 796.27 | 0.68 | 0.20 | 3.42 | 6.31E-04 | 7.54E-03 |
| Aqp7 | 233.89 | 0.68 | 0.27 | 2.51 | 1.22E-02 | 8.45E-02 |
| Trim41 | 759.95 | 0.68 | 0.22 | 3.03 | 2.41E-03 | 2.33E-02 |
| Rreb1 | 1247.95 | 0.68 | 0.23 | 2.92 | 3.51E-03 | 3.14E-02 |
| Rsad1 | 391.00 | 0.68 | 0.22 | 3.02 | 2.51E-03 | 2.40E-02 |
| Mturn | 528.36 | 0.67 | 0.21 | 3.14 | 1.70E-03 | 1.75E-02 |
| Mrc2 | 828.39 | 0.67 | 0.24 | 2.78 | 5.41E-03 | 4.46E-02 |
| Ezh1 | 1079.99 | 0.67 | 0.20 | 3.41 | 6.47E-04 | 7.71E-03 |
| Esrrb | 691.79 | 0.67 | 0.25 | 2.69 | 7.05E-03 | 5.51E-02 |
| Kcng2 | 2866.62 | 0.67 | 0.20 | 3.32 | 9.03E-04 | 1.03E-02 |
| Ttc33 | 517.31 | 0.67 | 0.26 | 2.60 | 9.20E-03 | 6.87E-02 |
| Fktn | 699.45 | 0.67 | 0.22 | 3.12 | 1.82E-03 | 1.86E-02 |
| Kcnn2 | 214.99 | 0.67 | 0.27 | 2.51 | 1.22E-02 | 8.45E-02 |
| Zfyve27 | 615.04 | 0.67 | 0.20 | 3.43 | 6.04E-04 | 7.28E-03 |
| Grk6 | 514.42 | 0.67 | 0.21 | 3.19 | 1.42E-03 | 1.52E-02 |
| Rnf14 | 286.37 | 0.67 | 0.27 | 2.48 | 1.33E-02 | 9.03E-02 |
| Abl1 | 1357.70 | 0.66 | 0.18 | 3.70 | 2.18E-04 | 2.99E-03 |
| Fgf13 | 1056.14 | 0.66 | 0.25 | 2.62 | 8.82E-03 | 6.63E-02 |
| Tia1 | 513.89 | 0.66 | 0.27 | 2.49 | 1.29E-02 | 8.84E-02 |
| Mllt6 | 3160.84 | 0.66 | 0.17 | 3.96 | 7.58E-05 | 1.19E-03 |
| Zbed3 | 233.85 | 0.66 | 0.27 | 2.44 | 1.49E-02 | 9.83E-02 |
| Gal3st3 | 308.22 | 0.66 | 0.26 | 2.53 | 1.15E-02 | 8.17E-02 |
| Wipf3 | 1226.42 | 0.66 | 0.21 | 3.08 | 2.09E-03 | 2.08E-02 |
| Adora1 | 350.47 | 0.65 | 0.27 | 2.46 | 1.39E-02 | 9.33E-02 |
| Rfwd3 | 563.69 | 0.65 | 0.22 | 2.94 | 3.26E-03 | 2.95E-02 |
| Fbxl20 | 715.00 | 0.65 | 0.21 | 3.07 | 2.17E-03 | 2.14E-02 |
| Ednra | 678.77 | 0.65 | 0.22 | 2.91 | 3.59E-03 | 3.20E-02 |
| Ints6l | 353.21 | 0.65 | 0.26 | 2.51 | 1.22E-02 | 8.45E-02 |
| Pde4c | 279.99 | 0.65 | 0.26 | 2.47 | 1.34E-02 | 9.08E-02 |
| Uri1 | 407.12 | 0.65 | 0.23 | 2.78 | 5.43E-03 | 4.47E-02 |
| Adck2 | 259.91 | 0.65 | 0.26 | 2.52 | 1.19E-02 | 8.32E-02 |
| Zfp809 | 305.31 | 0.65 | 0.27 | 2.43 | 1.53E-02 | 1.00E-01 |
| Sema3g | 539.23 | 0.65 | 0.23 | 2.88 | 4.02E-03 | 3.52E-02 |
| Mlh3 | 300.19 | 0.65 | 0.27 | 2.43 | 1.51E-02 | 9.91E-02 |
| Rin2 | 696.59 | 0.65 | 0.22 | 2.96 | 3.12E-03 | 2.84E-02 |
| Arl6ip4 | 437.82 | 0.64 | 0.23 | 2.78 | 5.52E-03 | 4.53E-02 |
| Rnf145 | 337.53 | 0.64 | 0.25 | 2.59 | 9.65E-03 | 7.10E-02 |
| Pcgf3 | 585.60 | 0.64 | 0.23 | 2.82 | 4.83E-03 | 4.08E-02 |
| Cnot3 | 528.39 | 0.64 | 0.21 | 3.11 | 1.84E-03 | 1.88E-02 |
| Szt2 | 863.54 | 0.64 | 0.20 | 3.24 | 1.17E-03 | 1.29E-02 |
| Slc25a42 | 1917.36 | 0.64 | 0.22 | 2.93 | 3.40E-03 | 3.06E-02 |
| Pdp1 | 898.96 | 0.64 | 0.22 | 2.96 | 3.12E-03 | 2.84E-02 |
| Kalrn | 425.67 | 0.64 | 0.23 | 2.76 | 5.75E-03 | 4.68E-02 |
| Elk3 | 627.01 | 0.64 | 0.26 | 2.44 | 1.48E-02 | 9.79E-02 |
| Fbxo8 | 758.81 | 0.63 | 0.20 | 3.09 | 1.99E-03 | 2.00E-02 |
| Pkd1 | 2689.93 | 0.63 | 0.17 | 3.77 | 1.61E-04 | 2.30E-03 |
| Efnb3 | 1929.54 | 0.63 | 0.26 | 2.46 | 1.38E-02 | 9.30E-02 |
| Gle1 | 678.88 | 0.63 | 0.20 | 3.11 | 1.89E-03 | 1.92E-02 |
| Stk16 | 484.07 | 0.63 | 0.26 | 2.47 | 1.35E-02 | 9.11E-02 |
| Itpr3 | 355.54 | 0.63 | 0.23 | 2.72 | 6.51E-03 | 5.17E-02 |
| Mitf | 1083.77 | 0.63 | 0.22 | 2.84 | 4.50E-03 | 3.86E-02 |
| Kat14 | 552.43 | 0.63 | 0.25 | 2.52 | 1.18E-02 | 8.28E-02 |
| Vipas39 | 506.13 | 0.63 | 0.21 | 3.01 | 2.65E-03 | 2.50E-02 |
| Tecpr1 | 1193.81 | 0.63 | 0.19 | 3.37 | 7.52E-04 | 8.79E-03 |
| Vps33a | 645.60 | 0.63 | 0.21 | 2.93 | 3.35E-03 | 3.02E-02 |
| Nagpa | 295.21 | 0.62 | 0.25 | 2.52 | 1.18E-02 | 8.29E-02 |
| Zmynd8 | 1294.65 | 0.62 | 0.18 | 3.53 | 4.17E-04 | 5.25E-03 |
| Pde7b | 551.65 | 0.62 | 0.23 | 2.64 | 8.39E-03 | 6.36E-02 |
| Cdkn1b | 1019.90 | 0.62 | 0.22 | 2.84 | 4.47E-03 | 3.84E-02 |
| Fmo5 | 396.28 | 0.62 | 0.25 | 2.43 | 1.50E-02 | 9.90E-02 |
| Pde2a | 1489.19 | 0.62 | 0.25 | 2.45 | 1.44E-02 | 9.58E-02 |
| Ing4 | 458.76 | 0.62 | 0.22 | 2.87 | 4.14E-03 | 3.62E-02 |
| Zkscan3 | 986.90 | 0.62 | 0.25 | 2.48 | 1.31E-02 | 8.94E-02 |
| Doc2g | 493.70 | 0.61 | 0.23 | 2.66 | 7.75E-03 | 5.95E-02 |
| Tmem106b | 2592.38 | 0.61 | 0.23 | 2.61 | 8.93E-03 | 6.70E-02 |
| Coro6 | 8498.86 | 0.61 | 0.20 | 3.01 | 2.62E-03 | 2.48E-02 |
| Gdi1 | 1957.72 | 0.61 | 0.23 | 2.64 | 8.26E-03 | 6.28E-02 |
| Unk | 415.86 | 0.60 | 0.24 | 2.47 | 1.33E-02 | 9.05E-02 |
| Cir1 | 494.61 | 0.60 | 0.22 | 2.79 | 5.20E-03 | 4.33E-02 |
| Eif2s3x | 2843.62 | 0.60 | 0.16 | 3.82 | 1.33E-04 | 1.94E-03 |
| Zfp397 | 589.94 | 0.60 | 0.20 | 3.00 | 2.66E-03 | 2.51E-02 |
| Ldb1 | 1105.96 | 0.60 | 0.20 | 3.07 | 2.11E-03 | 2.08E-02 |
| Rnf167 | 978.23 | 0.60 | 0.18 | 3.27 | 1.07E-03 | 1.20E-02 |
| Acot11 | 762.17 | 0.60 | 0.21 | 2.85 | 4.43E-03 | 3.81E-02 |
| Slc4a3 | 9093.27 | 0.59 | 0.17 | 3.58 | 3.49E-04 | 4.52E-03 |
| Nbeal2 | 467.76 | 0.59 | 0.24 | 2.50 | 1.23E-02 | 8.49E-02 |
| Flcn | 1955.16 | 0.59 | 0.20 | 2.98 | 2.90E-03 | 2.68E-02 |
| Meis1 | 499.98 | 0.59 | 0.21 | 2.82 | 4.80E-03 | 4.06E-02 |
| Zdhhc1 | 597.79 | 0.59 | 0.20 | 2.97 | 2.96E-03 | 2.72E-02 |
| Ecm2 | 691.84 | 0.59 | 0.22 | 2.73 | 6.26E-03 | 5.01E-02 |
| Tmem94 | 2857.45 | 0.59 | 0.21 | 2.85 | 4.39E-03 | 3.78E-02 |
| Zcchc8 | 482.10 | 0.59 | 0.23 | 2.59 | 9.47E-03 | 7.01E-02 |
| Mxd4 | 1305.23 | 0.59 | 0.18 | 3.30 | 9.61E-04 | 1.08E-02 |
| Cant1 | 557.30 | 0.59 | 0.21 | 2.76 | 5.84E-03 | 4.73E-02 |
| Slu7 | 684.49 | 0.59 | 0.21 | 2.82 | 4.74E-03 | 4.02E-02 |
| Cspg4 | 636.08 | 0.59 | 0.23 | 2.58 | 9.80E-03 | 7.20E-02 |
| Wbp1l | 1546.85 | 0.59 | 0.22 | 2.71 | 6.75E-03 | 5.31E-02 |
| Mef2c | 1290.78 | 0.59 | 0.22 | 2.71 | 6.71E-03 | 5.30E-02 |
| Arfip2 | 365.64 | 0.58 | 0.23 | 2.50 | 1.26E-02 | 8.65E-02 |
| Ncoa6 | 760.29 | 0.58 | 0.21 | 2.83 | 4.59E-03 | 3.92E-02 |
| Pitpnm2 | 2003.78 | 0.58 | 0.21 | 2.76 | 5.76E-03 | 4.68E-02 |
| 2310002L09Rik | 1102.21 | 0.58 | 0.23 | 2.54 | 1.09E-02 | 7.85E-02 |
| Fam53a | 523.54 | 0.57 | 0.23 | 2.54 | 1.12E-02 | 8.01E-02 |
| Fam32a | 554.37 | 0.57 | 0.22 | 2.62 | 8.90E-03 | 6.68E-02 |
| Scara5 | 1046.18 | 0.57 | 0.19 | 2.97 | 3.02E-03 | 2.76E-02 |
| Kansl3 | 1909.98 | 0.57 | 0.19 | 2.96 | 3.09E-03 | 2.82E-02 |
| Arhgef17 | 2422.38 | 0.57 | 0.21 | 2.68 | 7.45E-03 | 5.75E-02 |
| Zfyve21 | 774.26 | 0.56 | 0.21 | 2.72 | 6.53E-03 | 5.18E-02 |
| Atp13a2 | 543.20 | 0.56 | 0.21 | 2.71 | 6.70E-03 | 5.28E-02 |
| Mmp15 | 2569.44 | 0.56 | 0.23 | 2.49 | 1.29E-02 | 8.81E-02 |
| Aldh9a1 | 1251.18 | 0.55 | 0.21 | 2.63 | 8.46E-03 | 6.40E-02 |
| Mcoln1 | 633.65 | 0.55 | 0.21 | 2.60 | 9.29E-03 | 6.91E-02 |
| AW549877 | 986.32 | 0.55 | 0.19 | 2.88 | 3.99E-03 | 3.50E-02 |
| Capn15 | 477.35 | 0.55 | 0.22 | 2.53 | 1.13E-02 | 8.06E-02 |
| Obsl1 | 3184.22 | 0.55 | 0.17 | 3.16 | 1.56E-03 | 1.63E-02 |
| Ip6k1 | 1677.82 | 0.55 | 0.21 | 2.63 | 8.65E-03 | 6.53E-02 |
| Mrs2 | 1580.11 | 0.54 | 0.22 | 2.51 | 1.22E-02 | 8.45E-02 |
| Marchf8 | 1187.99 | 0.54 | 0.19 | 2.82 | 4.74E-03 | 4.02E-02 |
| Mff | 2112.80 | 0.54 | 0.18 | 3.02 | 2.52E-03 | 2.41E-02 |
| Gpsm1 | 2814.87 | 0.54 | 0.20 | 2.73 | 6.31E-03 | 5.05E-02 |
| Tcap | 29776.76 | 0.53 | 0.16 | 3.30 | 9.63E-04 | 1.09E-02 |
| Zfp219 | 707.70 | 0.53 | 0.21 | 2.53 | 1.13E-02 | 8.07E-02 |
| Leng8 | 3356.47 | 0.53 | 0.20 | 2.65 | 8.15E-03 | 6.20E-02 |
| Dgcr2 | 1220.59 | 0.53 | 0.19 | 2.76 | 5.70E-03 | 4.65E-02 |
| 2700081O15Rik | 812.35 | 0.53 | 0.19 | 2.77 | 5.58E-03 | 4.57E-02 |
| Wrnip1 | 653.75 | 0.53 | 0.20 | 2.59 | 9.48E-03 | 7.01E-02 |
| Kdm5c | 1597.96 | 0.53 | 0.19 | 2.74 | 6.19E-03 | 4.96E-02 |
| Stard3 | 515.58 | 0.52 | 0.21 | 2.50 | 1.23E-02 | 8.49E-02 |
| Dock6 | 1753.87 | 0.52 | 0.20 | 2.55 | 1.07E-02 | 7.67E-02 |
| Rit1 | 1257.07 | 0.52 | 0.18 | 2.93 | 3.41E-03 | 3.06E-02 |
| Dnajc14 | 760.99 | 0.52 | 0.21 | 2.49 | 1.29E-02 | 8.81E-02 |
| Ctdsp2 | 3757.77 | 0.52 | 0.17 | 2.99 | 2.78E-03 | 2.60E-02 |
| Sh3bp5l | 587.03 | 0.52 | 0.21 | 2.48 | 1.32E-02 | 9.01E-02 |
| Nisch | 5239.66 | 0.51 | 0.17 | 3.00 | 2.73E-03 | 2.56E-02 |
| Trpm4 | 776.67 | 0.51 | 0.20 | 2.64 | 8.33E-03 | 6.33E-02 |
| Gypc | 818.07 | 0.51 | 0.20 | 2.58 | 9.78E-03 | 7.19E-02 |
| Eef2k | 802.23 | 0.51 | 0.20 | 2.57 | 1.03E-02 | 7.46E-02 |
| Secisbp2 | 640.65 | 0.51 | 0.20 | 2.51 | 1.21E-02 | 8.44E-02 |
| Tnrc18 | 1461.95 | 0.50 | 0.17 | 2.89 | 3.90E-03 | 3.44E-02 |
| Pde4dip | 48813.39 | 0.50 | 0.18 | 2.70 | 6.97E-03 | 5.46E-02 |
| Trim3 | 651.78 | 0.50 | 0.20 | 2.53 | 1.13E-02 | 8.07E-02 |
| Pdgfrb | 1801.24 | 0.49 | 0.19 | 2.61 | 8.94E-03 | 6.70E-02 |
| Selenbp1 | 3055.78 | 0.48 | 0.18 | 2.60 | 9.21E-03 | 6.87E-02 |
| Max | 1035.56 | 0.48 | 0.18 | 2.59 | 9.57E-03 | 7.07E-02 |
| Gtf2i | 4671.03 | 0.48 | 0.20 | 2.43 | 1.52E-02 | 9.94E-02 |
| Limd1 | 1807.75 | 0.48 | 0.19 | 2.44 | 1.46E-02 | 9.67E-02 |
| Plekhm2 | 3849.13 | 0.47 | 0.19 | 2.56 | 1.03E-02 | 7.49E-02 |
| Pcp4l1 | 5186.25 | 0.47 | 0.18 | 2.58 | 9.83E-03 | 7.22E-02 |
| Mat2b | 1714.47 | 0.47 | 0.19 | 2.49 | 1.27E-02 | 8.72E-02 |
| Cds2 | 4145.54 | 0.46 | 0.16 | 2.95 | 3.13E-03 | 2.85E-02 |
| Synpo2l | 2625.29 | 0.45 | 0.17 | 2.58 | 9.84E-03 | 7.22E-02 |
| Kcnip2 | 4310.64 | 0.44 | 0.17 | 2.59 | 9.64E-03 | 7.10E-02 |
| Zbtb4 | 1427.78 | 0.42 | 0.17 | 2.47 | 1.37E-02 | 9.21E-02 |
| Prrc2a | 6564.53 | -0.38 | 0.15 | -2.51 | 1.21E-02 | 8.44E-02 |
| Ipo5 | 5334.26 | -0.39 | 0.16 | -2.48 | 1.32E-02 | 8.98E-02 |
| Iars | 2042.78 | -0.40 | 0.17 | -2.44 | 1.46E-02 | 9.69E-02 |
| Chd4 | 6488.50 | -0.40 | 0.16 | -2.51 | 1.20E-02 | 8.38E-02 |
| Kif5b | 4390.15 | -0.41 | 0.17 | -2.48 | 1.31E-02 | 8.93E-02 |
| Trip12 | 3992.75 | -0.42 | 0.17 | -2.52 | 1.19E-02 | 8.34E-02 |
| Pdcd6ip | 2748.70 | -0.42 | 0.16 | -2.57 | 1.01E-02 | 7.39E-02 |
| Myl12a | 13128.91 | -0.43 | 0.16 | -2.63 | 8.47E-03 | 6.40E-02 |
| Cyfip1 | 1522.74 | -0.43 | 0.17 | -2.47 | 1.35E-02 | 9.12E-02 |
| Prdm2 | 1551.67 | -0.43 | 0.18 | -2.43 | 1.52E-02 | 9.96E-02 |
| Slc35f5 | 2523.07 | -0.44 | 0.17 | -2.54 | 1.10E-02 | 7.90E-02 |
| Smg1 | 2319.01 | -0.44 | 0.17 | -2.53 | 1.15E-02 | 8.13E-02 |
| G3bp1 | 3206.87 | -0.44 | 0.18 | -2.51 | 1.22E-02 | 8.45E-02 |
| Rab3gap2 | 997.23 | -0.44 | 0.18 | -2.46 | 1.41E-02 | 9.40E-02 |
| Vcl | 4519.73 | -0.44 | 0.16 | -2.81 | 4.88E-03 | 4.11E-02 |
| Lgals1 | 2849.19 | -0.44 | 0.18 | -2.48 | 1.31E-02 | 8.91E-02 |
| Gripap1 | 1145.84 | -0.45 | 0.18 | -2.46 | 1.40E-02 | 9.38E-02 |
| Degs1 | 1535.25 | -0.45 | 0.18 | -2.52 | 1.18E-02 | 8.32E-02 |
| Aebp2 | 1342.72 | -0.45 | 0.18 | -2.44 | 1.45E-02 | 9.64E-02 |
| mt-Nd1 | 643475.44 | -0.45 | 0.18 | -2.57 | 1.02E-02 | 7.40E-02 |
| Furin | 3169.00 | -0.45 | 0.18 | -2.45 | 1.45E-02 | 9.63E-02 |
| Cited2 | 4052.90 | -0.45 | 0.18 | -2.48 | 1.33E-02 | 9.04E-02 |
| Lrrc59 | 1288.88 | -0.46 | 0.18 | -2.58 | 9.99E-03 | 7.30E-02 |
| Akap13 | 3464.18 | -0.46 | 0.16 | -2.79 | 5.23E-03 | 4.34E-02 |
| Csnk1a1 | 6130.10 | -0.46 | 0.17 | -2.70 | 6.91E-03 | 5.42E-02 |
| Hfe | 1201.45 | -0.46 | 0.18 | -2.57 | 1.02E-02 | 7.42E-02 |
| Arpc2 | 2940.90 | -0.46 | 0.19 | -2.45 | 1.45E-02 | 9.63E-02 |
| Mapkapk2 | 10659.57 | -0.46 | 0.15 | -3.03 | 2.48E-03 | 2.38E-02 |
| Cdc42bpb | 3144.64 | -0.47 | 0.17 | -2.77 | 5.58E-03 | 4.57E-02 |
| Gnb1 | 3257.01 | -0.47 | 0.19 | -2.46 | 1.40E-02 | 9.35E-02 |
| Pgam2 | 8932.59 | -0.47 | 0.19 | -2.47 | 1.34E-02 | 9.08E-02 |
| Ttn | 11925.91 | -0.47 | 0.18 | -2.56 | 1.05E-02 | 7.56E-02 |
| Rbm3 | 1734.31 | -0.47 | 0.18 | -2.61 | 8.99E-03 | 6.74E-02 |
| Ube2z | 1595.73 | -0.47 | 0.17 | -2.74 | 6.12E-03 | 4.92E-02 |
| Dstn | 3866.62 | -0.47 | 0.18 | -2.57 | 1.01E-02 | 7.35E-02 |
| Ccnd1 | 1026.85 | -0.48 | 0.19 | -2.52 | 1.18E-02 | 8.29E-02 |
| Hspa5 | 24044.75 | -0.48 | 0.20 | -2.45 | 1.44E-02 | 9.58E-02 |
| Ino80 | 726.56 | -0.48 | 0.19 | -2.51 | 1.19E-02 | 8.36E-02 |
| Kcmf1 | 2562.44 | -0.48 | 0.16 | -2.92 | 3.55E-03 | 3.17E-02 |
| Tspan9 | 1432.69 | -0.48 | 0.17 | -2.83 | 4.64E-03 | 3.95E-02 |
| Ptges3 | 2063.06 | -0.48 | 0.17 | -2.80 | 5.11E-03 | 4.26E-02 |
| Hsp90ab1 | 41724.52 | -0.48 | 0.18 | -2.70 | 6.96E-03 | 5.45E-02 |
| Zc3h7a | 1333.69 | -0.48 | 0.18 | -2.69 | 7.17E-03 | 5.56E-02 |
| Pak2 | 1576.99 | -0.48 | 0.17 | -2.87 | 4.10E-03 | 3.58E-02 |
| Smarca5 | 2270.92 | -0.48 | 0.17 | -2.82 | 4.84E-03 | 4.08E-02 |
| Hspb6 | 18272.56 | -0.49 | 0.17 | -2.83 | 4.58E-03 | 3.92E-02 |
| Ube2j1 | 947.88 | -0.49 | 0.19 | -2.62 | 8.77E-03 | 6.60E-02 |
| Retreg1 | 9986.05 | -0.49 | 0.17 | -2.97 | 3.00E-03 | 2.75E-02 |
| Xirp2 | 10731.86 | -0.49 | 0.17 | -2.95 | 3.20E-03 | 2.90E-02 |
| Ctsl | 4873.12 | -0.49 | 0.15 | -3.18 | 1.46E-03 | 1.55E-02 |
| Tpp2 | 1687.45 | -0.49 | 0.18 | -2.76 | 5.82E-03 | 4.72E-02 |
| Mtdh | 2150.87 | -0.49 | 0.17 | -2.88 | 3.99E-03 | 3.50E-02 |
| Pcnx | 2429.14 | -0.50 | 0.20 | -2.43 | 1.51E-02 | 9.91E-02 |
| Spsb1 | 1307.94 | -0.50 | 0.17 | -2.86 | 4.27E-03 | 3.70E-02 |
| Ube2d3 | 9578.13 | -0.50 | 0.16 | -3.11 | 1.84E-03 | 1.88E-02 |
| Mob1a | 1081.99 | -0.50 | 0.18 | -2.81 | 4.89E-03 | 4.11E-02 |
| Rab43 | 536.16 | -0.50 | 0.20 | -2.46 | 1.39E-02 | 9.33E-02 |
| Rasa3 | 962.90 | -0.50 | 0.19 | -2.57 | 1.03E-02 | 7.46E-02 |
| Rnf2 | 587.38 | -0.50 | 0.20 | -2.53 | 1.16E-02 | 8.17E-02 |
| Itpr1 | 1295.36 | -0.50 | 0.20 | -2.46 | 1.40E-02 | 9.39E-02 |
| Tmem123 | 1762.38 | -0.50 | 0.18 | -2.80 | 5.10E-03 | 4.25E-02 |
| Far1 | 788.75 | -0.51 | 0.20 | -2.59 | 9.65E-03 | 7.10E-02 |
| Ipo7 | 4255.01 | -0.51 | 0.17 | -3.03 | 2.49E-03 | 2.39E-02 |
| mt-Co2 | 856608.21 | -0.51 | 0.20 | -2.54 | 1.12E-02 | 8.01E-02 |
| Nufip2 | 1151.23 | -0.51 | 0.18 | -2.75 | 6.00E-03 | 4.84E-02 |
| Srsf10 | 1527.26 | -0.51 | 0.19 | -2.75 | 6.02E-03 | 4.85E-02 |
| Fus | 8474.44 | -0.51 | 0.16 | -3.10 | 1.92E-03 | 1.94E-02 |
| Vamp3 | 1344.57 | -0.51 | 0.20 | -2.54 | 1.10E-02 | 7.87E-02 |
| Nus1 | 1605.36 | -0.51 | 0.18 | -2.78 | 5.37E-03 | 4.44E-02 |
| Kras | 1110.49 | -0.52 | 0.18 | -2.82 | 4.74E-03 | 4.02E-02 |
| Nab1 | 1177.41 | -0.52 | 0.21 | -2.46 | 1.39E-02 | 9.32E-02 |
| Gnl2 | 1273.50 | -0.52 | 0.21 | -2.52 | 1.19E-02 | 8.34E-02 |
| Hdac5 | 4845.12 | -0.52 | 0.20 | -2.60 | 9.42E-03 | 6.99E-02 |
| Cryab | 33650.01 | -0.52 | 0.16 | -3.26 | 1.11E-03 | 1.23E-02 |
| N4bp2l2 | 744.24 | -0.52 | 0.19 | -2.69 | 7.08E-03 | 5.53E-02 |
| Taf1 | 887.50 | -0.53 | 0.20 | -2.65 | 7.98E-03 | 6.10E-02 |
| F11r | 1443.76 | -0.53 | 0.18 | -2.92 | 3.49E-03 | 3.12E-02 |
| mt-Co3 | 969778.42 | -0.53 | 0.18 | -3.00 | 2.69E-03 | 2.53E-02 |
| Prkch | 696.01 | -0.53 | 0.21 | -2.57 | 1.02E-02 | 7.41E-02 |
| Stk17b | 442.12 | -0.54 | 0.22 | -2.43 | 1.50E-02 | 9.90E-02 |
| Ranbp2 | 2647.35 | -0.54 | 0.18 | -2.92 | 3.46E-03 | 3.11E-02 |
| Adar | 1003.21 | -0.54 | 0.20 | -2.74 | 6.13E-03 | 4.92E-02 |
| Fam98b | 818.61 | -0.54 | 0.20 | -2.76 | 5.77E-03 | 4.69E-02 |
| Gbp7 | 1161.11 | -0.54 | 0.22 | -2.47 | 1.34E-02 | 9.08E-02 |
| Aen | 998.27 | -0.54 | 0.20 | -2.76 | 5.79E-03 | 4.70E-02 |
| Sf3b1 | 5755.92 | -0.54 | 0.18 | -3.03 | 2.41E-03 | 2.33E-02 |
| Tgfbr1 | 689.54 | -0.54 | 0.20 | -2.69 | 7.15E-03 | 5.55E-02 |
| Wdr26 | 1236.36 | -0.55 | 0.20 | -2.68 | 7.42E-03 | 5.73E-02 |
| Cmpk1 | 804.83 | -0.55 | 0.22 | -2.45 | 1.45E-02 | 9.63E-02 |
| Slc23a2 | 974.00 | -0.55 | 0.22 | -2.52 | 1.19E-02 | 8.34E-02 |
| Wdr1 | 2920.94 | -0.55 | 0.18 | -3.05 | 2.31E-03 | 2.25E-02 |
| Tax1bp3 | 805.62 | -0.55 | 0.20 | -2.68 | 7.34E-03 | 5.68E-02 |
| Frmd4a | 1234.47 | -0.55 | 0.18 | -2.98 | 2.91E-03 | 2.69E-02 |
| Map4k4 | 4533.52 | -0.55 | 0.16 | -3.36 | 7.75E-04 | 9.05E-03 |
| Htt | 979.52 | -0.55 | 0.20 | -2.75 | 5.94E-03 | 4.79E-02 |
| Fam91a1 | 735.18 | -0.55 | 0.21 | -2.60 | 9.43E-03 | 6.99E-02 |
| Ifnar2 | 1003.14 | -0.55 | 0.20 | -2.75 | 6.02E-03 | 4.85E-02 |
| Zfp622 | 814.10 | -0.55 | 0.19 | -2.90 | 3.67E-03 | 3.26E-02 |
| Erf | 1239.92 | -0.55 | 0.22 | -2.48 | 1.32E-02 | 9.01E-02 |
| Uaca | 2720.68 | -0.55 | 0.17 | -3.23 | 1.24E-03 | 1.35E-02 |
| Med13l | 1404.08 | -0.55 | 0.18 | -3.05 | 2.29E-03 | 2.24E-02 |
| Il13ra1 | 1141.05 | -0.55 | 0.18 | -3.05 | 2.27E-03 | 2.22E-02 |
| Calm2 | 3062.04 | -0.55 | 0.18 | -3.03 | 2.43E-03 | 2.34E-02 |
| Zc3h15 | 1422.75 | -0.56 | 0.20 | -2.84 | 4.46E-03 | 3.84E-02 |
| Cd83 | 1835.98 | -0.56 | 0.23 | -2.46 | 1.39E-02 | 9.32E-02 |
| Zyx | 1967.74 | -0.56 | 0.21 | -2.73 | 6.35E-03 | 5.07E-02 |
| Pdlim3 | 769.89 | -0.56 | 0.22 | -2.50 | 1.26E-02 | 8.66E-02 |
| Diaph1 | 1281.22 | -0.56 | 0.20 | -2.87 | 4.17E-03 | 3.63E-02 |
| Atp6v1h | 639.57 | -0.56 | 0.22 | -2.54 | 1.12E-02 | 7.99E-02 |
| Hnrnpc | 3563.72 | -0.56 | 0.16 | -3.42 | 6.19E-04 | 7.42E-03 |
| Dnajb6 | 2506.59 | -0.56 | 0.18 | -3.16 | 1.55E-03 | 1.62E-02 |
| Cdh5 | 5632.25 | -0.56 | 0.20 | -2.85 | 4.38E-03 | 3.78E-02 |
| Chmp4b | 3422.46 | -0.56 | 0.21 | -2.67 | 7.69E-03 | 5.92E-02 |
| Braf | 685.87 | -0.56 | 0.21 | -2.69 | 7.14E-03 | 5.55E-02 |
| Zfp330 | 580.89 | -0.56 | 0.23 | -2.43 | 1.51E-02 | 9.91E-02 |
| Bag3 | 7953.44 | -0.57 | 0.20 | -2.89 | 3.84E-03 | 3.39E-02 |
| Kdm7a | 938.02 | -0.57 | 0.21 | -2.74 | 6.08E-03 | 4.90E-02 |
| Mrc1 | 1482.46 | -0.57 | 0.19 | -2.98 | 2.90E-03 | 2.68E-02 |
| Sdad1 | 582.45 | -0.57 | 0.23 | -2.51 | 1.21E-02 | 8.44E-02 |
| Wdr43 | 771.92 | -0.57 | 0.21 | -2.74 | 6.16E-03 | 4.94E-02 |
| Smox | 675.59 | -0.57 | 0.20 | -2.88 | 3.94E-03 | 3.47E-02 |
| Fnbp1l | 1137.20 | -0.57 | 0.21 | -2.78 | 5.38E-03 | 4.44E-02 |
| Slc11a2 | 1071.80 | -0.57 | 0.18 | -3.10 | 1.92E-03 | 1.94E-02 |
| Kpna4 | 3190.88 | -0.57 | 0.17 | -3.42 | 6.33E-04 | 7.56E-03 |
| Zfp131 | 951.95 | -0.57 | 0.19 | -3.08 | 2.10E-03 | 2.08E-02 |
| Gabarapl1 | 3399.07 | -0.58 | 0.17 | -3.49 | 4.92E-04 | 6.08E-03 |
| Zfp335 | 812.07 | -0.58 | 0.19 | -3.05 | 2.28E-03 | 2.23E-02 |
| Por | 1970.74 | -0.58 | 0.22 | -2.64 | 8.30E-03 | 6.31E-02 |
| B4galt1 | 2649.19 | -0.58 | 0.19 | -3.07 | 2.11E-03 | 2.09E-02 |
| Sbds | 1345.89 | -0.58 | 0.22 | -2.59 | 9.53E-03 | 7.04E-02 |
| Etf1 | 3948.59 | -0.58 | 0.18 | -3.32 | 9.09E-04 | 1.03E-02 |
| C1s1 | 1419.64 | -0.58 | 0.22 | -2.68 | 7.34E-03 | 5.68E-02 |
| Ubb-ps | 1628.84 | -0.58 | 0.21 | -2.72 | 6.44E-03 | 5.12E-02 |
| Mvp | 2360.46 | -0.58 | 0.18 | -3.31 | 9.20E-04 | 1.04E-02 |
| Galnt18 | 366.09 | -0.58 | 0.24 | -2.44 | 1.45E-02 | 9.64E-02 |
| Cd93 | 3809.45 | -0.59 | 0.18 | -3.34 | 8.48E-04 | 9.75E-03 |
| Pde8a | 605.38 | -0.59 | 0.22 | -2.72 | 6.56E-03 | 5.19E-02 |
| Gls | 2543.54 | -0.59 | 0.19 | -3.14 | 1.72E-03 | 1.77E-02 |
| Sin3a | 1257.63 | -0.59 | 0.19 | -3.16 | 1.57E-03 | 1.64E-02 |
| Hs6st1 | 1122.14 | -0.59 | 0.18 | -3.19 | 1.42E-03 | 1.52E-02 |
| Pdcd10 | 500.79 | -0.59 | 0.23 | -2.58 | 9.98E-03 | 7.30E-02 |
| Capza1 | 1276.06 | -0.59 | 0.19 | -3.18 | 1.46E-03 | 1.55E-02 |
| Micu1 | 410.62 | -0.59 | 0.22 | -2.63 | 8.47E-03 | 6.40E-02 |
| Ube2v2 | 384.30 | -0.59 | 0.24 | -2.51 | 1.21E-02 | 8.45E-02 |
| Dync1li1 | 1508.24 | -0.59 | 0.19 | -3.08 | 2.09E-03 | 2.07E-02 |
| Tbrg1 | 1063.51 | -0.59 | 0.20 | -3.01 | 2.63E-03 | 2.49E-02 |
| Lrrfip1 | 2843.16 | -0.59 | 0.18 | -3.21 | 1.34E-03 | 1.44E-02 |
| Hnrnpf | 4749.97 | -0.59 | 0.21 | -2.80 | 5.15E-03 | 4.29E-02 |
| Atxn7 | 986.10 | -0.59 | 0.20 | -3.04 | 2.40E-03 | 2.32E-02 |
| Slfn5 | 6297.06 | -0.59 | 0.19 | -3.06 | 2.22E-03 | 2.18E-02 |
| Twf2 | 5463.37 | -0.59 | 0.19 | -3.04 | 2.34E-03 | 2.28E-02 |
| Cebpg | 1129.79 | -0.59 | 0.22 | -2.70 | 6.89E-03 | 5.41E-02 |
| Rapgef1 | 5045.92 | -0.59 | 0.16 | -3.62 | 2.91E-04 | 3.88E-03 |
| Tmem33 | 1062.77 | -0.59 | 0.18 | -3.25 | 1.17E-03 | 1.28E-02 |
| Tsc22d1 | 9229.24 | -0.59 | 0.21 | -2.78 | 5.38E-03 | 4.44E-02 |
| Eif3j2 | 1416.93 | -0.60 | 0.19 | -3.12 | 1.80E-03 | 1.85E-02 |
| Mcam | 1558.04 | -0.60 | 0.22 | -2.68 | 7.46E-03 | 5.75E-02 |
| Trim63 | 7530.89 | -0.60 | 0.20 | -2.96 | 3.03E-03 | 2.78E-02 |
| Mgst1 | 921.26 | -0.60 | 0.20 | -2.94 | 3.24E-03 | 2.94E-02 |
| Cald1 | 2099.44 | -0.60 | 0.18 | -3.30 | 9.66E-04 | 1.09E-02 |
| Ppfibp1 | 2278.24 | -0.60 | 0.21 | -2.80 | 5.07E-03 | 4.23E-02 |
| Ankrd28 | 1138.19 | -0.60 | 0.18 | -3.36 | 7.78E-04 | 9.07E-03 |
| Smad1 | 533.67 | -0.60 | 0.23 | -2.66 | 7.77E-03 | 5.95E-02 |
| Prpf38a | 431.54 | -0.60 | 0.23 | -2.65 | 8.07E-03 | 6.15E-02 |
| Eml1 | 2550.15 | -0.61 | 0.18 | -3.42 | 6.15E-04 | 7.38E-03 |
| Myo1d | 443.91 | -0.61 | 0.22 | -2.70 | 7.02E-03 | 5.48E-02 |
| Pcf11 | 1357.17 | -0.61 | 0.19 | -3.26 | 1.11E-03 | 1.23E-02 |
| Itpripl2 | 1369.74 | -0.61 | 0.22 | -2.82 | 4.81E-03 | 4.06E-02 |
| Nop14 | 750.14 | -0.61 | 0.23 | -2.63 | 8.47E-03 | 6.40E-02 |
| Ccdc86 | 298.95 | -0.61 | 0.24 | -2.53 | 1.14E-02 | 8.11E-02 |
| Impdh2 | 1738.92 | -0.61 | 0.24 | -2.50 | 1.26E-02 | 8.67E-02 |
| Optn | 2397.97 | -0.61 | 0.21 | -2.86 | 4.17E-03 | 3.63E-02 |
| Nt5dc3 | 2578.32 | -0.61 | 0.17 | -3.54 | 4.00E-04 | 5.07E-03 |
| Msn | 6144.59 | -0.62 | 0.17 | -3.67 | 2.45E-04 | 3.30E-03 |
| Atp6v1a | 998.43 | -0.62 | 0.21 | -3.02 | 2.57E-03 | 2.44E-02 |
| Tbk1 | 878.73 | -0.62 | 0.23 | -2.73 | 6.38E-03 | 5.09E-02 |
| N4bp1 | 1450.49 | -0.62 | 0.18 | -3.55 | 3.80E-04 | 4.86E-03 |
| Hnrnpu | 7961.17 | -0.63 | 0.17 | -3.72 | 1.98E-04 | 2.76E-03 |
| Elmo1 | 339.64 | -0.63 | 0.25 | -2.51 | 1.20E-02 | 8.37E-02 |
| Zfp281 | 575.78 | -0.63 | 0.21 | -2.98 | 2.90E-03 | 2.68E-02 |
| Naf1 | 337.54 | -0.63 | 0.23 | -2.68 | 7.41E-03 | 5.73E-02 |
| Adss | 509.55 | -0.63 | 0.26 | -2.46 | 1.41E-02 | 9.40E-02 |
| Ppp4r2 | 1733.34 | -0.63 | 0.21 | -2.98 | 2.85E-03 | 2.65E-02 |
| Wsb2 | 2957.21 | -0.63 | 0.17 | -3.71 | 2.08E-04 | 2.87E-03 |
| Pid1 | 339.92 | -0.63 | 0.24 | -2.58 | 9.93E-03 | 7.27E-02 |
| Rbm4b | 320.86 | -0.63 | 0.25 | -2.47 | 1.34E-02 | 9.08E-02 |
| Cmtm6 | 1025.96 | -0.63 | 0.26 | -2.43 | 1.51E-02 | 9.91E-02 |
| Raf1 | 3647.38 | -0.63 | 0.17 | -3.73 | 1.91E-04 | 2.68E-03 |
| Rbm7 | 679.59 | -0.63 | 0.21 | -3.04 | 2.39E-03 | 2.31E-02 |
| Cggbp1 | 910.09 | -0.63 | 0.20 | -3.17 | 1.50E-03 | 1.59E-02 |
| Cavin4 | 2672.43 | -0.63 | 0.17 | -3.68 | 2.35E-04 | 3.19E-03 |
| Klhl41 | 2582.33 | -0.63 | 0.21 | -3.04 | 2.34E-03 | 2.28E-02 |
| Serpine2 | 925.17 | -0.63 | 0.23 | -2.72 | 6.62E-03 | 5.23E-02 |
| Zbtb16 | 4559.83 | -0.63 | 0.26 | -2.43 | 1.51E-02 | 9.90E-02 |
| Aff4 | 2284.84 | -0.63 | 0.20 | -3.16 | 1.58E-03 | 1.65E-02 |
| Rapgef5 | 1585.27 | -0.64 | 0.20 | -3.14 | 1.67E-03 | 1.72E-02 |
| Hspa8 | 64078.21 | -0.64 | 0.20 | -3.17 | 1.51E-03 | 1.59E-02 |
| Crk | 3458.39 | -0.64 | 0.19 | -3.44 | 5.72E-04 | 6.95E-03 |
| Asap2 | 837.97 | -0.64 | 0.20 | -3.22 | 1.27E-03 | 1.37E-02 |
| Cdkn2aip | 408.96 | -0.64 | 0.23 | -2.81 | 4.90E-03 | 4.12E-02 |
| Pgs1 | 840.38 | -0.64 | 0.22 | -2.92 | 3.53E-03 | 3.15E-02 |
| Gpx1 | 1467.68 | -0.64 | 0.24 | -2.68 | 7.42E-03 | 5.73E-02 |
| Senp2 | 868.17 | -0.64 | 0.21 | -3.10 | 1.96E-03 | 1.97E-02 |
| Samd4b | 1266.34 | -0.64 | 0.19 | -3.40 | 6.76E-04 | 8.01E-03 |
| Trp53 | 586.95 | -0.65 | 0.24 | -2.69 | 7.18E-03 | 5.57E-02 |
| Arih2 | 1661.18 | -0.65 | 0.19 | -3.32 | 8.95E-04 | 1.02E-02 |
| Des | 56982.68 | -0.65 | 0.15 | -4.42 | 1.00E-05 | 1.98E-04 |
| Lysmd3 | 587.02 | -0.65 | 0.22 | -2.98 | 2.87E-03 | 2.67E-02 |
| Mindy3 | 331.01 | -0.65 | 0.23 | -2.77 | 5.66E-03 | 4.62E-02 |
| Entpd1 | 602.91 | -0.65 | 0.25 | -2.57 | 1.02E-02 | 7.42E-02 |
| Pxn | 2055.72 | -0.65 | 0.21 | -3.11 | 1.90E-03 | 1.93E-02 |
| Scyl2 | 876.86 | -0.65 | 0.20 | -3.24 | 1.18E-03 | 1.30E-02 |
| Usp16 | 1441.51 | -0.65 | 0.17 | -3.85 | 1.16E-04 | 1.73E-03 |
| Bzw1 | 2912.11 | -0.66 | 0.17 | -3.96 | 7.65E-05 | 1.19E-03 |
| Camkk2 | 493.14 | -0.66 | 0.24 | -2.72 | 6.61E-03 | 5.23E-02 |
| Heatr1 | 507.36 | -0.66 | 0.26 | -2.56 | 1.06E-02 | 7.62E-02 |
| Anxa3 | 823.71 | -0.66 | 0.21 | -3.08 | 2.09E-03 | 2.07E-02 |
| Arpc5 | 1061.88 | -0.66 | 0.23 | -2.89 | 3.89E-03 | 3.43E-02 |
| Eif2s2 | 2566.27 | -0.66 | 0.21 | -3.16 | 1.60E-03 | 1.66E-02 |
| Lgals9 | 857.90 | -0.66 | 0.23 | -2.83 | 4.71E-03 | 4.00E-02 |
| Cd82 | 370.19 | -0.66 | 0.22 | -2.99 | 2.83E-03 | 2.64E-02 |
| Ptma | 7555.61 | -0.66 | 0.21 | -3.11 | 1.85E-03 | 1.88E-02 |
| Csf2ra | 414.83 | -0.67 | 0.24 | -2.83 | 4.66E-03 | 3.97E-02 |
| Bcor | 1557.11 | -0.67 | 0.20 | -3.35 | 7.95E-04 | 9.21E-03 |
| Tmx4 | 2274.27 | -0.67 | 0.21 | -3.18 | 1.45E-03 | 1.54E-02 |
| Rbm19 | 467.82 | -0.67 | 0.21 | -3.16 | 1.57E-03 | 1.64E-02 |
| Dusp16 | 1115.41 | -0.67 | 0.23 | -2.96 | 3.09E-03 | 2.83E-02 |
| Polr2a | 3106.98 | -0.67 | 0.16 | -4.19 | 2.82E-05 | 4.95E-04 |
| Med10 | 346.26 | -0.67 | 0.27 | -2.44 | 1.46E-02 | 9.69E-02 |
| Traf2 | 544.41 | -0.67 | 0.24 | -2.80 | 5.03E-03 | 4.21E-02 |
| Rbm12 | 510.65 | -0.67 | 0.24 | -2.84 | 4.46E-03 | 3.84E-02 |
| Nras | 1196.75 | -0.67 | 0.25 | -2.70 | 6.95E-03 | 5.44E-02 |
| Cyth3 | 1539.87 | -0.67 | 0.23 | -2.97 | 2.96E-03 | 2.72E-02 |
| Dynll1 | 830.97 | -0.67 | 0.27 | -2.50 | 1.24E-02 | 8.54E-02 |
| Mapk1ip1l | 1270.24 | -0.67 | 0.18 | -3.68 | 2.38E-04 | 3.22E-03 |
| Actn1 | 1253.84 | -0.67 | 0.23 | -2.98 | 2.88E-03 | 2.67E-02 |
| Fam129a | 906.64 | -0.67 | 0.18 | -3.67 | 2.42E-04 | 3.27E-03 |
| Phldb1 | 8121.96 | -0.67 | 0.15 | -4.42 | 9.77E-06 | 1.93E-04 |
| Snx7 | 225.33 | -0.67 | 0.27 | -2.49 | 1.26E-02 | 8.67E-02 |
| Acly | 1336.32 | -0.68 | 0.20 | -3.33 | 8.74E-04 | 1.00E-02 |
| Adnp2 | 593.46 | -0.68 | 0.26 | -2.57 | 1.03E-02 | 7.47E-02 |
| B3gnt2 | 530.32 | -0.68 | 0.25 | -2.75 | 5.88E-03 | 4.75E-02 |
| Chac1 | 285.40 | -0.68 | 0.26 | -2.58 | 9.89E-03 | 7.25E-02 |
| Rfx1 | 909.78 | -0.68 | 0.19 | -3.51 | 4.46E-04 | 5.57E-03 |
| 2410002F23Rik | 644.56 | -0.68 | 0.24 | -2.82 | 4.82E-03 | 4.06E-02 |
| Spata6 | 250.06 | -0.68 | 0.26 | -2.60 | 9.39E-03 | 6.97E-02 |
| Arsa | 591.63 | -0.68 | 0.27 | -2.54 | 1.11E-02 | 7.92E-02 |
| Soga1 | 886.69 | -0.68 | 0.22 | -3.10 | 1.96E-03 | 1.96E-02 |
| Traf3 | 687.06 | -0.68 | 0.24 | -2.86 | 4.30E-03 | 3.72E-02 |
| Morf4l2 | 2395.73 | -0.68 | 0.21 | -3.21 | 1.32E-03 | 1.43E-02 |
| Birc2 | 965.55 | -0.68 | 0.18 | -3.74 | 1.83E-04 | 2.57E-03 |
| Arpc3 | 1687.14 | -0.68 | 0.23 | -2.96 | 3.07E-03 | 2.81E-02 |
| Dip2b | 405.74 | -0.68 | 0.25 | -2.76 | 5.86E-03 | 4.74E-02 |
| Oser1 | 649.60 | -0.68 | 0.20 | -3.50 | 4.72E-04 | 5.85E-03 |
| Cpeb4 | 2470.87 | -0.69 | 0.17 | -3.93 | 8.45E-05 | 1.30E-03 |
| Prnp | 3562.58 | -0.69 | 0.17 | -4.12 | 3.73E-05 | 6.28E-04 |
| Nlrc5 | 448.95 | -0.69 | 0.26 | -2.68 | 7.43E-03 | 5.74E-02 |
| Rbpj | 1453.42 | -0.69 | 0.18 | -3.81 | 1.38E-04 | 2.01E-03 |
| Phc2 | 1128.34 | -0.69 | 0.21 | -3.35 | 8.15E-04 | 9.41E-03 |
| Acsl6 | 523.66 | -0.69 | 0.28 | -2.47 | 1.33E-02 | 9.05E-02 |
| Ankrd10 | 1092.30 | -0.69 | 0.19 | -3.68 | 2.29E-04 | 3.13E-03 |
| Lima1 | 1698.63 | -0.70 | 0.24 | -2.94 | 3.30E-03 | 2.98E-02 |
| Slc7a6 | 502.19 | -0.70 | 0.22 | -3.09 | 1.97E-03 | 1.97E-02 |
| Iws1 | 966.89 | -0.70 | 0.19 | -3.66 | 2.54E-04 | 3.42E-03 |
| Eef1a1 | 27508.39 | -0.70 | 0.24 | -2.92 | 3.49E-03 | 3.12E-02 |
| Ppig | 1288.96 | -0.70 | 0.22 | -3.17 | 1.51E-03 | 1.59E-02 |
| Itgav | 902.17 | -0.70 | 0.23 | -3.01 | 2.57E-03 | 2.45E-02 |
| Fndc3a | 930.73 | -0.70 | 0.23 | -3.11 | 1.88E-03 | 1.91E-02 |
| Sec23b | 604.16 | -0.70 | 0.23 | -3.09 | 1.97E-03 | 1.97E-02 |
| Tma16 | 355.62 | -0.70 | 0.24 | -2.88 | 3.92E-03 | 3.45E-02 |
| Svep1 | 677.10 | -0.70 | 0.20 | -3.51 | 4.51E-04 | 5.62E-03 |
| Atp6v1b2 | 2493.93 | -0.70 | 0.19 | -3.81 | 1.40E-04 | 2.02E-03 |
| Slc7a6os | 453.00 | -0.71 | 0.25 | -2.81 | 4.94E-03 | 4.15E-02 |
| Cfl1 | 3471.58 | -0.71 | 0.28 | -2.57 | 1.02E-02 | 7.44E-02 |
| Fmr1 | 675.38 | -0.71 | 0.25 | -2.86 | 4.22E-03 | 3.66E-02 |
| Ccdc50 | 1099.57 | -0.71 | 0.20 | -3.55 | 3.81E-04 | 4.86E-03 |
| Calm1 | 5603.74 | -0.71 | 0.18 | -3.96 | 7.46E-05 | 1.17E-03 |
| Tfe3 | 762.62 | -0.71 | 0.20 | -3.56 | 3.66E-04 | 4.71E-03 |
| Taf1d | 517.02 | -0.72 | 0.25 | -2.85 | 4.39E-03 | 3.78E-02 |
| Gar1 | 251.86 | -0.72 | 0.27 | -2.65 | 7.97E-03 | 6.09E-02 |
| Rnf149 | 315.69 | -0.72 | 0.28 | -2.53 | 1.15E-02 | 8.15E-02 |
| Mcl1 | 12338.28 | -0.72 | 0.19 | -3.75 | 1.80E-04 | 2.53E-03 |
| Tmsb10 | 1020.93 | -0.72 | 0.25 | -2.86 | 4.18E-03 | 3.64E-02 |
| Mak16 | 422.40 | -0.72 | 0.27 | -2.69 | 7.06E-03 | 5.51E-02 |
| Septin11 | 1101.15 | -0.72 | 0.20 | -3.69 | 2.26E-04 | 3.09E-03 |
| Ptpn1 | 1053.25 | -0.72 | 0.20 | -3.61 | 3.07E-04 | 4.06E-03 |
| Akna | 379.12 | -0.72 | 0.26 | -2.74 | 6.13E-03 | 4.92E-02 |
| Adam17 | 1355.79 | -0.72 | 0.19 | -3.72 | 1.99E-04 | 2.77E-03 |
| Hnrnph1 | 7446.16 | -0.73 | 0.18 | -4.06 | 4.88E-05 | 7.98E-04 |
| Sec24b | 1221.44 | -0.73 | 0.22 | -3.35 | 7.96E-04 | 9.22E-03 |
| Spata5 | 256.80 | -0.73 | 0.30 | -2.46 | 1.38E-02 | 9.27E-02 |
| Ubc | 61023.46 | -0.73 | 0.17 | -4.36 | 1.28E-05 | 2.44E-04 |
| Aars | 2473.03 | -0.73 | 0.24 | -3.02 | 2.53E-03 | 2.42E-02 |
| Efemp1 | 329.10 | -0.73 | 0.27 | -2.71 | 6.74E-03 | 5.31E-02 |
| B2m | 6333.35 | -0.74 | 0.17 | -4.26 | 2.00E-05 | 3.67E-04 |
| Mafb | 840.02 | -0.74 | 0.27 | -2.76 | 5.72E-03 | 4.66E-02 |
| Lpar1 | 459.75 | -0.74 | 0.23 | -3.22 | 1.27E-03 | 1.37E-02 |
| Nfatc2 | 804.76 | -0.74 | 0.27 | -2.69 | 7.11E-03 | 5.54E-02 |
| Tank | 686.03 | -0.74 | 0.21 | -3.56 | 3.67E-04 | 4.72E-03 |
| Sgpl1 | 913.05 | -0.74 | 0.20 | -3.80 | 1.46E-04 | 2.10E-03 |
| Dot1l | 3883.85 | -0.75 | 0.20 | -3.69 | 2.24E-04 | 3.07E-03 |
| Btaf1 | 1064.81 | -0.75 | 0.21 | -3.51 | 4.48E-04 | 5.60E-03 |
| Arpc1b | 1813.43 | -0.75 | 0.27 | -2.81 | 4.96E-03 | 4.16E-02 |
| Homer1 | 1626.23 | -0.75 | 0.19 | -3.90 | 9.57E-05 | 1.45E-03 |
| Nfat5 | 1738.87 | -0.75 | 0.22 | -3.45 | 5.55E-04 | 6.80E-03 |
| Clu | 16420.56 | -0.75 | 0.15 | -4.92 | 8.60E-07 | 2.08E-05 |
| F2r | 1335.86 | -0.75 | 0.20 | -3.77 | 1.62E-04 | 2.30E-03 |
| Rhou | 274.25 | -0.75 | 0.29 | -2.65 | 8.11E-03 | 6.18E-02 |
| Osgin2 | 296.03 | -0.76 | 0.27 | -2.81 | 4.94E-03 | 4.15E-02 |
| Tnks1bp1 | 2365.35 | -0.76 | 0.18 | -4.23 | 2.36E-05 | 4.23E-04 |
| Usp37 | 434.06 | -0.76 | 0.29 | -2.66 | 7.74E-03 | 5.94E-02 |
| Ubb | 22679.77 | -0.76 | 0.18 | -4.13 | 3.65E-05 | 6.19E-04 |
| Maml1 | 864.80 | -0.76 | 0.21 | -3.57 | 3.54E-04 | 4.56E-03 |
| Cldnd1 | 957.04 | -0.76 | 0.23 | -3.40 | 6.85E-04 | 8.10E-03 |
| Pik3r3 | 429.09 | -0.77 | 0.23 | -3.26 | 1.10E-03 | 1.22E-02 |
| Lrrc8c | 1106.32 | -0.77 | 0.19 | -4.12 | 3.80E-05 | 6.37E-04 |
| Spag9 | 5901.18 | -0.77 | 0.16 | -4.72 | 2.39E-06 | 5.32E-05 |
| Zfp516 | 707.94 | -0.77 | 0.23 | -3.39 | 7.07E-04 | 8.35E-03 |
| Tnrc6b | 1337.44 | -0.77 | 0.18 | -4.38 | 1.17E-05 | 2.27E-04 |
| P4ha1 | 1892.19 | -0.77 | 0.25 | -3.03 | 2.46E-03 | 2.36E-02 |
| Gm49273 | 820.35 | -0.77 | 0.28 | -2.76 | 5.73E-03 | 4.66E-02 |
| Mast4 | 1396.93 | -0.77 | 0.21 | -3.61 | 3.02E-04 | 4.01E-03 |
| Coq8b | 310.68 | -0.77 | 0.26 | -2.91 | 3.61E-03 | 3.21E-02 |
| Prkx | 1129.17 | -0.77 | 0.19 | -4.09 | 4.23E-05 | 7.01E-04 |
| Atp13a3 | 1665.89 | -0.77 | 0.18 | -4.18 | 2.92E-05 | 5.11E-04 |
| Nedd9 | 1546.10 | -0.77 | 0.24 | -3.19 | 1.41E-03 | 1.50E-02 |
| Slc25a30 | 415.44 | -0.77 | 0.26 | -2.99 | 2.80E-03 | 2.61E-02 |
| Ccnt1 | 1192.05 | -0.78 | 0.18 | -4.41 | 1.03E-05 | 2.03E-04 |
| S1pr3 | 962.34 | -0.78 | 0.31 | -2.46 | 1.38E-02 | 9.27E-02 |
| Kitl | 2832.84 | -0.78 | 0.16 | -4.91 | 8.95E-07 | 2.16E-05 |
| Lrrc8a | 1379.57 | -0.78 | 0.19 | -3.98 | 6.77E-05 | 1.07E-03 |
| Nop56 | 1277.99 | -0.78 | 0.26 | -2.99 | 2.83E-03 | 2.63E-02 |
| Cmip | 492.55 | -0.78 | 0.26 | -3.02 | 2.49E-03 | 2.39E-02 |
| Edc3 | 378.82 | -0.78 | 0.26 | -2.95 | 3.17E-03 | 2.88E-02 |
| Col18a1 | 541.99 | -0.78 | 0.30 | -2.56 | 1.04E-02 | 7.51E-02 |
| Gprc5b | 628.39 | -0.78 | 0.24 | -3.23 | 1.22E-03 | 1.34E-02 |
| Ampd2 | 416.47 | -0.78 | 0.24 | -3.27 | 1.08E-03 | 1.20E-02 |
| Rell1 | 631.15 | -0.78 | 0.27 | -2.86 | 4.24E-03 | 3.68E-02 |
| Cd9 | 1184.48 | -0.78 | 0.29 | -2.66 | 7.76E-03 | 5.95E-02 |
| Ackr3 | 3660.46 | -0.78 | 0.18 | -4.37 | 1.22E-05 | 2.34E-04 |
| Rapgef2 | 1556.08 | -0.78 | 0.23 | -3.42 | 6.26E-04 | 7.50E-03 |
| Zbtb21 | 261.92 | -0.78 | 0.29 | -2.72 | 6.46E-03 | 5.14E-02 |
| Zbtb2 | 345.92 | -0.79 | 0.24 | -3.23 | 1.25E-03 | 1.36E-02 |
| Jmjd1c | 2251.49 | -0.79 | 0.27 | -2.88 | 3.99E-03 | 3.50E-02 |
| Fzd1 | 362.14 | -0.79 | 0.25 | -3.17 | 1.55E-03 | 1.62E-02 |
| Dlgap4 | 2487.34 | -0.79 | 0.18 | -4.27 | 2.00E-05 | 3.67E-04 |
| Slc2a1 | 872.22 | -0.79 | 0.32 | -2.49 | 1.29E-02 | 8.81E-02 |
| Anxa1 | 1751.04 | -0.79 | 0.26 | -3.01 | 2.59E-03 | 2.46E-02 |
| Arih1 | 3047.52 | -0.79 | 0.18 | -4.50 | 6.86E-06 | 1.40E-04 |
| Vav3 | 233.51 | -0.79 | 0.29 | -2.76 | 5.79E-03 | 4.70E-02 |
| Pros1 | 999.78 | -0.79 | 0.27 | -2.92 | 3.52E-03 | 3.14E-02 |
| Rybp | 753.79 | -0.79 | 0.20 | -3.88 | 1.03E-04 | 1.55E-03 |
| Bhlhe40 | 6864.96 | -0.79 | 0.27 | -2.99 | 2.82E-03 | 2.63E-02 |
| Tra2a | 1638.08 | -0.79 | 0.18 | -4.39 | 1.11E-05 | 2.17E-04 |
| Pnrc1 | 5076.17 | -0.79 | 0.19 | -4.14 | 3.43E-05 | 5.88E-04 |
| Ror1 | 197.86 | -0.79 | 0.30 | -2.65 | 8.10E-03 | 6.18E-02 |
| Nolc1 | 1074.20 | -0.79 | 0.23 | -3.41 | 6.55E-04 | 7.79E-03 |
| Ckap4 | 776.03 | -0.79 | 0.23 | -3.45 | 5.68E-04 | 6.93E-03 |
| Tra2b | 1710.23 | -0.80 | 0.17 | -4.70 | 2.58E-06 | 5.70E-05 |
| Tmem251 | 228.94 | -0.80 | 0.33 | -2.43 | 1.51E-02 | 9.91E-02 |
| Actr3 | 3932.73 | -0.80 | 0.18 | -4.46 | 8.32E-06 | 1.67E-04 |
| Edn1 | 428.03 | -0.80 | 0.24 | -3.33 | 8.62E-04 | 9.90E-03 |
| Pisd | 1018.11 | -0.80 | 0.19 | -4.18 | 2.90E-05 | 5.08E-04 |
| Picalm | 6642.03 | -0.80 | 0.17 | -4.84 | 1.31E-06 | 3.03E-05 |
| Nfatc1 | 833.97 | -0.80 | 0.26 | -3.04 | 2.33E-03 | 2.27E-02 |
| Mal | 400.75 | -0.80 | 0.27 | -2.99 | 2.77E-03 | 2.59E-02 |
| Nlgn2 | 542.97 | -0.80 | 0.27 | -2.94 | 3.33E-03 | 3.01E-02 |
| Bcl6b | 2612.48 | -0.80 | 0.17 | -4.85 | 1.22E-06 | 2.85E-05 |
| Pabpc1 | 4384.76 | -0.80 | 0.23 | -3.51 | 4.41E-04 | 5.51E-03 |
| Cd74 | 1721.75 | -0.81 | 0.21 | -3.86 | 1.13E-04 | 1.68E-03 |
| Ninj1 | 1394.03 | -0.81 | 0.21 | -3.78 | 1.55E-04 | 2.23E-03 |
| Zbtb10 | 888.36 | -0.81 | 0.19 | -4.16 | 3.23E-05 | 5.58E-04 |
| Man2a1 | 1574.71 | -0.81 | 0.19 | -4.30 | 1.69E-05 | 3.15E-04 |
| Idi1 | 302.22 | -0.81 | 0.25 | -3.20 | 1.38E-03 | 1.48E-02 |
| Col4a2 | 12756.72 | -0.81 | 0.15 | -5.36 | 8.11E-08 | 2.35E-06 |
| Arl4d | 572.15 | -0.81 | 0.28 | -2.93 | 3.36E-03 | 3.03E-02 |
| Plcg2 | 253.14 | -0.81 | 0.27 | -3.05 | 2.28E-03 | 2.23E-02 |
| Rrad | 22300.63 | -0.81 | 0.28 | -2.89 | 3.88E-03 | 3.42E-02 |
| Dennd5b | 895.39 | -0.81 | 0.19 | -4.36 | 1.32E-05 | 2.50E-04 |
| Slc7a1 | 3233.86 | -0.81 | 0.17 | -4.71 | 2.50E-06 | 5.54E-05 |
| Ogfr | 1608.12 | -0.81 | 0.20 | -4.11 | 4.04E-05 | 6.73E-04 |
| Dgat1 | 690.76 | -0.81 | 0.29 | -2.84 | 4.47E-03 | 3.84E-02 |
| Ptgir | 268.83 | -0.81 | 0.33 | -2.44 | 1.48E-02 | 9.79E-02 |
| Xylt1 | 154.58 | -0.81 | 0.32 | -2.53 | 1.14E-02 | 8.11E-02 |
| Gpr132 | 162.29 | -0.82 | 0.31 | -2.60 | 9.45E-03 | 7.00E-02 |
| Slc9a3r1 | 288.28 | -0.82 | 0.28 | -2.90 | 3.73E-03 | 3.31E-02 |
| Fmnl2 | 478.25 | -0.82 | 0.27 | -2.98 | 2.91E-03 | 2.68E-02 |
| Eif2ak2 | 884.12 | -0.82 | 0.33 | -2.51 | 1.19E-02 | 8.34E-02 |
| Alyref | 552.35 | -0.82 | 0.30 | -2.71 | 6.69E-03 | 5.28E-02 |
| Leo1 | 495.72 | -0.82 | 0.28 | -2.98 | 2.90E-03 | 2.68E-02 |
| Ubxn4 | 2274.20 | -0.82 | 0.23 | -3.55 | 3.83E-04 | 4.88E-03 |
| Med13 | 1465.61 | -0.82 | 0.24 | -3.38 | 7.25E-04 | 8.52E-03 |
| Ssh1 | 711.61 | -0.83 | 0.28 | -2.91 | 3.58E-03 | 3.19E-02 |
| Prr13 | 761.02 | -0.83 | 0.26 | -3.20 | 1.36E-03 | 1.46E-02 |
| Hexim1 | 1860.83 | -0.83 | 0.18 | -4.62 | 3.93E-06 | 8.41E-05 |
| Arf4 | 2406.53 | -0.83 | 0.19 | -4.33 | 1.52E-05 | 2.86E-04 |
| Mapre1 | 2302.49 | -0.83 | 0.17 | -4.78 | 1.79E-06 | 4.05E-05 |
| Hes1 | 1734.64 | -0.83 | 0.27 | -3.13 | 1.76E-03 | 1.80E-02 |
| H2ax | 289.86 | -0.83 | 0.27 | -3.09 | 2.02E-03 | 2.02E-02 |
| Polr3d | 569.78 | -0.83 | 0.25 | -3.27 | 1.08E-03 | 1.20E-02 |
| Ippk | 382.54 | -0.83 | 0.34 | -2.43 | 1.52E-02 | 9.94E-02 |
| Ifi35 | 618.19 | -0.83 | 0.23 | -3.64 | 2.67E-04 | 3.59E-03 |
| Fbxo34 | 443.67 | -0.83 | 0.21 | -3.90 | 9.76E-05 | 1.47E-03 |
| Steap4 | 2119.04 | -0.83 | 0.23 | -3.63 | 2.87E-04 | 3.83E-03 |
| C4b | 993.67 | -0.83 | 0.34 | -2.49 | 1.29E-02 | 8.81E-02 |
| Erbin | 2458.58 | -0.83 | 0.20 | -4.26 | 2.03E-05 | 3.73E-04 |
| Acot9 | 808.91 | -0.84 | 0.24 | -3.54 | 4.00E-04 | 5.07E-03 |
| Mcm3 | 259.35 | -0.84 | 0.30 | -2.79 | 5.31E-03 | 4.40E-02 |
| Slc1a5 | 1189.25 | -0.84 | 0.32 | -2.62 | 8.74E-03 | 6.58E-02 |
| Urb1 | 532.65 | -0.84 | 0.25 | -3.38 | 7.33E-04 | 8.59E-03 |
| Parp9 | 932.86 | -0.84 | 0.28 | -3.04 | 2.36E-03 | 2.29E-02 |
| Coq10b | 1714.04 | -0.84 | 0.22 | -3.90 | 9.55E-05 | 1.45E-03 |
| Gvin1 | 3083.41 | -0.84 | 0.20 | -4.22 | 2.49E-05 | 4.42E-04 |
| Rasa4 | 289.36 | -0.84 | 0.27 | -3.10 | 1.90E-03 | 1.93E-02 |
| Spsb4 | 136.83 | -0.85 | 0.34 | -2.50 | 1.24E-02 | 8.59E-02 |
| Zfand5 | 472.57 | -0.85 | 0.31 | -2.77 | 5.66E-03 | 4.62E-02 |
| Arf6 | 963.66 | -0.85 | 0.27 | -3.09 | 2.00E-03 | 2.00E-02 |
| Prkab2 | 1931.31 | -0.85 | 0.18 | -4.75 | 2.06E-06 | 4.62E-05 |
| Dnaja4 | 4895.32 | -0.85 | 0.19 | -4.48 | 7.61E-06 | 1.54E-04 |
| Sertad2 | 1293.68 | -0.85 | 0.22 | -3.94 | 8.27E-05 | 1.28E-03 |
| Ttyh2 | 158.21 | -0.85 | 0.35 | -2.45 | 1.43E-02 | 9.52E-02 |
| Acsl5 | 687.09 | -0.85 | 0.24 | -3.49 | 4.80E-04 | 5.93E-03 |
| Tgoln1 | 4531.90 | -0.85 | 0.21 | -4.04 | 5.31E-05 | 8.61E-04 |
| Slc25a33 | 1243.72 | -0.85 | 0.27 | -3.11 | 1.88E-03 | 1.91E-02 |
| Tars | 1037.00 | -0.85 | 0.24 | -3.54 | 4.07E-04 | 5.14E-03 |
| Tlnrd1 | 918.89 | -0.85 | 0.26 | -3.29 | 9.88E-04 | 1.11E-02 |
| Acsl3 | 287.02 | -0.85 | 0.27 | -3.14 | 1.70E-03 | 1.75E-02 |
| Klf7 | 1845.63 | -0.85 | 0.18 | -4.80 | 1.56E-06 | 3.57E-05 |
| Sqstm1 | 17261.41 | -0.85 | 0.17 | -4.97 | 6.60E-07 | 1.63E-05 |
| Nasp | 875.62 | -0.85 | 0.21 | -4.13 | 3.58E-05 | 6.09E-04 |
| Rgcc | 1011.86 | -0.85 | 0.31 | -2.77 | 5.64E-03 | 4.61E-02 |
| Tubb2a | 1310.15 | -0.86 | 0.26 | -3.33 | 8.65E-04 | 9.94E-03 |
| Cmtm8 | 352.47 | -0.86 | 0.27 | -3.15 | 1.65E-03 | 1.71E-02 |
| Lpin2 | 457.54 | -0.86 | 0.23 | -3.72 | 1.98E-04 | 2.76E-03 |
| Ccn3 | 238.10 | -0.86 | 0.34 | -2.53 | 1.12E-02 | 8.03E-02 |
| Timeless | 1142.34 | -0.86 | 0.30 | -2.82 | 4.84E-03 | 4.08E-02 |
| Bclaf3 | 159.02 | -0.86 | 0.33 | -2.62 | 8.70E-03 | 6.56E-02 |
| Pogk | 954.04 | -0.86 | 0.20 | -4.37 | 1.24E-05 | 2.38E-04 |
| D1Ertd622e | 204.75 | -0.86 | 0.30 | -2.86 | 4.21E-03 | 3.66E-02 |
| Vasp | 1178.81 | -0.86 | 0.22 | -3.87 | 1.10E-04 | 1.64E-03 |
| Dram1 | 295.21 | -0.86 | 0.26 | -3.36 | 7.77E-04 | 9.06E-03 |
| Serpinh1 | 8271.39 | -0.86 | 0.23 | -3.80 | 1.46E-04 | 2.10E-03 |
| Timd4 | 133.28 | -0.86 | 0.32 | -2.66 | 7.75E-03 | 5.95E-02 |
| Rrp15 | 213.68 | -0.86 | 0.31 | -2.77 | 5.62E-03 | 4.60E-02 |
| Parp16 | 120.67 | -0.86 | 0.35 | -2.49 | 1.28E-02 | 8.80E-02 |
| Tut7 | 2785.90 | -0.87 | 0.19 | -4.46 | 8.29E-06 | 1.66E-04 |
| Csrp1 | 1778.49 | -0.87 | 0.20 | -4.24 | 2.22E-05 | 4.02E-04 |
| Tgm2 | 10332.11 | -0.87 | 0.17 | -4.98 | 6.45E-07 | 1.60E-05 |
| Cotl1 | 375.09 | -0.87 | 0.32 | -2.69 | 7.24E-03 | 5.61E-02 |
| Clp1 | 138.26 | -0.87 | 0.34 | -2.54 | 1.10E-02 | 7.87E-02 |
| Gck | 393.77 | -0.87 | 0.26 | -3.36 | 7.87E-04 | 9.16E-03 |
| Rpl3 | 391.31 | -0.87 | 0.30 | -2.95 | 3.22E-03 | 2.92E-02 |
| Flt4 | 766.38 | -0.87 | 0.22 | -4.04 | 5.40E-05 | 8.74E-04 |
| Cited4 | 652.22 | -0.87 | 0.27 | -3.23 | 1.23E-03 | 1.34E-02 |
| Nkapd1 | 468.94 | -0.87 | 0.27 | -3.19 | 1.43E-03 | 1.52E-02 |
| E2f5 | 136.10 | -0.87 | 0.34 | -2.55 | 1.06E-02 | 7.66E-02 |
| Gas2l1 | 936.45 | -0.87 | 0.21 | -4.16 | 3.14E-05 | 5.44E-04 |
| Ube2f | 994.24 | -0.87 | 0.21 | -4.26 | 2.07E-05 | 3.78E-04 |
| Ywhaz | 4980.68 | -0.87 | 0.20 | -4.43 | 9.47E-06 | 1.88E-04 |
| Rpsa-ps10 | 156.76 | -0.87 | 0.32 | -2.78 | 5.50E-03 | 4.52E-02 |
| Nxf1 | 827.04 | -0.88 | 0.26 | -3.40 | 6.78E-04 | 8.02E-03 |
| Sfpq | 3367.84 | -0.88 | 0.20 | -4.40 | 1.06E-05 | 2.08E-04 |
| Gcc1 | 901.78 | -0.88 | 0.31 | -2.86 | 4.19E-03 | 3.64E-02 |
| Tex30 | 123.68 | -0.88 | 0.33 | -2.69 | 7.13E-03 | 5.55E-02 |
| Cttnbp2nl | 1216.90 | -0.88 | 0.18 | -4.91 | 9.17E-07 | 2.21E-05 |
| Map1b | 432.02 | -0.88 | 0.29 | -3.04 | 2.37E-03 | 2.30E-02 |
| Skap2 | 338.68 | -0.88 | 0.30 | -2.93 | 3.41E-03 | 3.06E-02 |
| Anxa7 | 3195.69 | -0.88 | 0.24 | -3.73 | 1.88E-04 | 2.64E-03 |
| Ninl | 168.21 | -0.88 | 0.36 | -2.49 | 1.29E-02 | 8.81E-02 |
| Fnip2 | 992.69 | -0.89 | 0.19 | -4.58 | 4.75E-06 | 1.00E-04 |
| Rhbdf1 | 1404.76 | -0.89 | 0.17 | -5.21 | 1.91E-07 | 5.21E-06 |
| Chic2 | 564.97 | -0.89 | 0.27 | -3.24 | 1.18E-03 | 1.30E-02 |
| Ptbp1 | 2111.64 | -0.89 | 0.21 | -4.24 | 2.19E-05 | 3.97E-04 |
| Rps6ka3 | 1965.51 | -0.89 | 0.18 | -4.91 | 9.28E-07 | 2.23E-05 |
| Nes | 2222.45 | -0.89 | 0.21 | -4.27 | 1.96E-05 | 3.60E-04 |
| Atp10a | 123.49 | -0.89 | 0.35 | -2.53 | 1.15E-02 | 8.13E-02 |
| Fstl1 | 3505.55 | -0.89 | 0.21 | -4.25 | 2.12E-05 | 3.87E-04 |
| Rtn4 | 2638.32 | -0.89 | 0.26 | -3.40 | 6.78E-04 | 8.02E-03 |
| C3 | 10376.52 | -0.90 | 0.23 | -3.89 | 1.01E-04 | 1.52E-03 |
| Anxa5 | 3259.62 | -0.90 | 0.25 | -3.59 | 3.25E-04 | 4.26E-03 |
| Rad52 | 259.67 | -0.90 | 0.29 | -3.07 | 2.13E-03 | 2.10E-02 |
| Cry1 | 400.92 | -0.90 | 0.30 | -2.98 | 2.89E-03 | 2.68E-02 |
| Cp | 3611.88 | -0.90 | 0.20 | -4.52 | 6.06E-06 | 1.25E-04 |
| 1810055G02Rik | 280.53 | -0.90 | 0.25 | -3.53 | 4.20E-04 | 5.28E-03 |
| Parp10 | 817.25 | -0.90 | 0.26 | -3.44 | 5.85E-04 | 7.08E-03 |
| Zranb1 | 1861.49 | -0.90 | 0.20 | -4.47 | 7.72E-06 | 1.56E-04 |
| Ywhaq | 198.10 | -0.90 | 0.31 | -2.87 | 4.12E-03 | 3.60E-02 |
| Rassf4 | 702.80 | -0.90 | 0.25 | -3.59 | 3.28E-04 | 4.29E-03 |
| Ccnyl1 | 769.89 | -0.90 | 0.21 | -4.25 | 2.16E-05 | 3.93E-04 |
| Slc10a6 | 393.90 | -0.90 | 0.30 | -3.00 | 2.69E-03 | 2.53E-02 |
| Efhd2 | 1017.82 | -0.90 | 0.30 | -3.03 | 2.41E-03 | 2.33E-02 |
| Slc41a3 | 1861.63 | -0.91 | 0.27 | -3.36 | 7.93E-04 | 9.21E-03 |
| Fem1b | 1136.32 | -0.91 | 0.22 | -4.09 | 4.23E-05 | 7.01E-04 |
| Cdk17 | 835.99 | -0.91 | 0.19 | -4.83 | 1.34E-06 | 3.09E-05 |
| Pak4 | 297.27 | -0.91 | 0.27 | -3.32 | 8.85E-04 | 1.01E-02 |
| 1110002E22Rik | 2383.73 | -0.91 | 0.19 | -4.69 | 2.73E-06 | 6.00E-05 |
| Ier5l | 639.94 | -0.91 | 0.30 | -3.02 | 2.53E-03 | 2.42E-02 |
| Foxo1 | 1181.39 | -0.92 | 0.19 | -4.90 | 9.63E-07 | 2.29E-05 |
| Nfe2l2 | 3284.09 | -0.92 | 0.18 | -5.16 | 2.49E-07 | 6.69E-06 |
| Etv5 | 479.42 | -0.92 | 0.29 | -3.20 | 1.39E-03 | 1.49E-02 |
| Slc22a15 | 225.87 | -0.92 | 0.29 | -3.14 | 1.66E-03 | 1.72E-02 |
| Ercc1 | 743.62 | -0.92 | 0.25 | -3.63 | 2.79E-04 | 3.73E-03 |
| Metrnl | 686.98 | -0.92 | 0.32 | -2.88 | 4.01E-03 | 3.52E-02 |
| Tcim | 2681.79 | -0.92 | 0.25 | -3.61 | 3.04E-04 | 4.03E-03 |
| Plekhg2 | 1520.35 | -0.92 | 0.23 | -3.96 | 7.65E-05 | 1.19E-03 |
| Shb | 751.22 | -0.92 | 0.24 | -3.90 | 9.60E-05 | 1.45E-03 |
| Adcy4 | 1504.05 | -0.92 | 0.24 | -3.81 | 1.38E-04 | 2.01E-03 |
| Mob3a | 417.15 | -0.92 | 0.29 | -3.23 | 1.24E-03 | 1.35E-02 |
| Gtpbp4 | 1411.08 | -0.92 | 0.21 | -4.46 | 8.35E-06 | 1.67E-04 |
| Slc39a1 | 2947.23 | -0.92 | 0.23 | -4.09 | 4.40E-05 | 7.26E-04 |
| Mt1 | 7300.53 | -0.92 | 0.19 | -4.89 | 1.00E-06 | 2.37E-05 |
| Marcks | 1751.73 | -0.93 | 0.20 | -4.53 | 5.94E-06 | 1.23E-04 |
| Lgals3bp | 1354.95 | -0.93 | 0.18 | -5.09 | 3.53E-07 | 9.24E-06 |
| Tnfrsf1a | 1692.28 | -0.93 | 0.25 | -3.73 | 1.92E-04 | 2.69E-03 |
| S100a11 | 709.65 | -0.93 | 0.26 | -3.59 | 3.37E-04 | 4.40E-03 |
| AW822252 | 333.56 | -0.93 | 0.38 | -2.47 | 1.34E-02 | 9.08E-02 |
| Tinagl1 | 2470.78 | -0.93 | 0.25 | -3.74 | 1.83E-04 | 2.57E-03 |
| Tle3 | 811.85 | -0.93 | 0.20 | -4.55 | 5.48E-06 | 1.14E-04 |
| Arl4a | 679.54 | -0.93 | 0.26 | -3.53 | 4.09E-04 | 5.16E-03 |
| H2-D1 | 10205.28 | -0.93 | 0.24 | -3.95 | 7.72E-05 | 1.20E-03 |
| Apobec3 | 500.81 | -0.93 | 0.24 | -3.89 | 9.98E-05 | 1.51E-03 |
| Zfp568 | 694.78 | -0.93 | 0.23 | -4.14 | 3.48E-05 | 5.94E-04 |
| Nup54 | 516.85 | -0.94 | 0.23 | -4.02 | 5.80E-05 | 9.34E-04 |
| Ppan | 361.70 | -0.94 | 0.35 | -2.72 | 6.63E-03 | 5.24E-02 |
| Medag | 894.78 | -0.94 | 0.35 | -2.65 | 7.99E-03 | 6.10E-02 |
| Klhl34 | 119.35 | -0.94 | 0.36 | -2.63 | 8.44E-03 | 6.39E-02 |
| Nfkbib | 916.05 | -0.94 | 0.23 | -4.03 | 5.54E-05 | 8.96E-04 |
| Ezh2 | 216.62 | -0.94 | 0.33 | -2.85 | 4.37E-03 | 3.77E-02 |
| Rab20 | 975.17 | -0.94 | 0.27 | -3.55 | 3.85E-04 | 4.91E-03 |
| Med11 | 197.76 | -0.94 | 0.35 | -2.72 | 6.54E-03 | 5.19E-02 |
| Tgtp2 | 1448.87 | -0.94 | 0.24 | -3.97 | 7.22E-05 | 1.14E-03 |
| Enah | 3857.34 | -0.95 | 0.21 | -4.43 | 9.22E-06 | 1.84E-04 |
| 4930523C07Rik | 936.69 | -0.95 | 0.21 | -4.57 | 4.81E-06 | 1.01E-04 |
| Tlr4 | 420.43 | -0.95 | 0.23 | -4.06 | 5.01E-05 | 8.17E-04 |
| Zfp217 | 637.22 | -0.95 | 0.24 | -3.99 | 6.53E-05 | 1.04E-03 |
| Vash1 | 412.14 | -0.95 | 0.26 | -3.63 | 2.84E-04 | 3.80E-03 |
| Ggct | 538.18 | -0.95 | 0.26 | -3.69 | 2.26E-04 | 3.09E-03 |
| Lamc2 | 274.96 | -0.95 | 0.37 | -2.59 | 9.72E-03 | 7.16E-02 |
| Ppp1r2 | 4407.80 | -0.95 | 0.17 | -5.47 | 4.59E-08 | 1.40E-06 |
| Ifitm2 | 1609.29 | -0.95 | 0.20 | -4.68 | 2.80E-06 | 6.15E-05 |
| Ctps | 1635.14 | -0.95 | 0.24 | -3.97 | 7.05E-05 | 1.11E-03 |
| Zfp800 | 339.70 | -0.95 | 0.23 | -4.07 | 4.73E-05 | 7.77E-04 |
| Brd2 | 3008.64 | -0.95 | 0.22 | -4.25 | 2.12E-05 | 3.87E-04 |
| Man1a | 1753.15 | -0.96 | 0.21 | -4.46 | 8.37E-06 | 1.67E-04 |
| Papss2 | 799.88 | -0.96 | 0.20 | -4.90 | 9.50E-07 | 2.27E-05 |
| Arf2 | 1026.22 | -0.96 | 0.21 | -4.47 | 7.73E-06 | 1.56E-04 |
| Ttpal | 782.82 | -0.96 | 0.25 | -3.82 | 1.35E-04 | 1.97E-03 |
| Prdm1 | 217.50 | -0.96 | 0.32 | -2.98 | 2.86E-03 | 2.65E-02 |
| Slc35e4 | 396.17 | -0.96 | 0.38 | -2.54 | 1.10E-02 | 7.86E-02 |
| Top1 | 2583.12 | -0.96 | 0.19 | -5.18 | 2.22E-07 | 5.99E-06 |
| Wapl | 1450.11 | -0.97 | 0.17 | -5.68 | 1.31E-08 | 4.30E-07 |
| Fignl2 | 216.00 | -0.97 | 0.38 | -2.56 | 1.04E-02 | 7.49E-02 |
| Jun | 18954.62 | -0.97 | 0.23 | -4.23 | 2.38E-05 | 4.25E-04 |
| Tagln2 | 3442.22 | -0.97 | 0.22 | -4.38 | 1.19E-05 | 2.31E-04 |
| Iffo2 | 438.37 | -0.97 | 0.23 | -4.13 | 3.67E-05 | 6.20E-04 |
| Mall | 187.30 | -0.97 | 0.35 | -2.78 | 5.48E-03 | 4.51E-02 |
| Slbp | 543.43 | -0.97 | 0.25 | -3.85 | 1.18E-04 | 1.75E-03 |
| Glipr2 | 168.88 | -0.97 | 0.30 | -3.21 | 1.34E-03 | 1.44E-02 |
| Mlkl | 213.60 | -0.97 | 0.32 | -3.00 | 2.66E-03 | 2.51E-02 |
| Ptbp3 | 1389.85 | -0.97 | 0.19 | -5.05 | 4.45E-07 | 1.14E-05 |
| Cyth4 | 291.28 | -0.97 | 0.28 | -3.53 | 4.10E-04 | 5.17E-03 |
| Ctdp1 | 567.37 | -0.97 | 0.31 | -3.18 | 1.46E-03 | 1.55E-02 |
| Pcna | 1639.23 | -0.97 | 0.24 | -4.13 | 3.68E-05 | 6.21E-04 |
| Clic4 | 20749.20 | -0.98 | 0.16 | -6.14 | 8.47E-10 | 3.18E-08 |
| Hmga1 | 176.43 | -0.98 | 0.37 | -2.61 | 9.00E-03 | 6.74E-02 |
| Zfp503 | 136.09 | -0.98 | 0.39 | -2.52 | 1.18E-02 | 8.29E-02 |
| Trim25 | 1885.42 | -0.98 | 0.18 | -5.46 | 4.68E-08 | 1.43E-06 |
| Sh3bp2 | 177.85 | -0.98 | 0.35 | -2.83 | 4.67E-03 | 3.98E-02 |
| Cemip2 | 377.76 | -0.98 | 0.32 | -3.08 | 2.07E-03 | 2.06E-02 |
| Csnk1d | 2661.19 | -0.98 | 0.20 | -4.92 | 8.49E-07 | 2.06E-05 |
| Elf4 | 631.08 | -0.98 | 0.25 | -3.87 | 1.11E-04 | 1.66E-03 |
| Stat2 | 1687.69 | -0.98 | 0.21 | -4.77 | 1.80E-06 | 4.07E-05 |
| Vmp1 | 1833.76 | -0.98 | 0.25 | -3.93 | 8.43E-05 | 1.30E-03 |
| Hivep2 | 1654.22 | -0.98 | 0.19 | -5.15 | 2.67E-07 | 7.13E-06 |
| Oas2 | 659.15 | -0.98 | 0.24 | -4.17 | 3.05E-05 | 5.32E-04 |
| Eif4a1 | 6368.61 | -0.99 | 0.18 | -5.53 | 3.25E-08 | 1.01E-06 |
| Acvrl1 | 1633.12 | -0.99 | 0.24 | -4.19 | 2.81E-05 | 4.95E-04 |
| Taf7 | 555.76 | -0.99 | 0.25 | -3.93 | 8.35E-05 | 1.29E-03 |
| Hspa1l | 240.87 | -0.99 | 0.27 | -3.60 | 3.19E-04 | 4.20E-03 |
| Dnajb4 | 6175.85 | -0.99 | 0.21 | -4.63 | 3.62E-06 | 7.81E-05 |
| Spry2 | 1010.18 | -0.99 | 0.30 | -3.27 | 1.07E-03 | 1.20E-02 |
| Slc7a7 | 131.74 | -0.99 | 0.39 | -2.52 | 1.16E-02 | 8.18E-02 |
| Nip7 | 352.69 | -0.99 | 0.31 | -3.18 | 1.49E-03 | 1.57E-02 |
| Tmbim1 | 2773.00 | -0.99 | 0.28 | -3.52 | 4.37E-04 | 5.48E-03 |
| Col5a3 | 1411.47 | -0.99 | 0.30 | -3.33 | 8.70E-04 | 9.99E-03 |
| Ero1l | 779.00 | -0.99 | 0.22 | -4.54 | 5.54E-06 | 1.15E-04 |
| Ddit3 | 1396.85 | -0.99 | 0.29 | -3.44 | 5.85E-04 | 7.08E-03 |
| Fcgr2b | 713.15 | -0.99 | 0.30 | -3.27 | 1.09E-03 | 1.21E-02 |
| Col4a1 | 17754.72 | -1.00 | 0.18 | -5.53 | 3.27E-08 | 1.01E-06 |
| Pirb | 353.50 | -1.00 | 0.28 | -3.57 | 3.53E-04 | 4.55E-03 |
| Tjp2 | 1053.66 | -1.00 | 0.22 | -4.43 | 9.50E-06 | 1.89E-04 |
| Gda | 911.09 | -1.00 | 0.19 | -5.11 | 3.14E-07 | 8.24E-06 |
| Rlim | 1423.53 | -1.00 | 0.19 | -5.19 | 2.09E-07 | 5.68E-06 |
| Foxs1 | 124.44 | -1.00 | 0.32 | -3.08 | 2.08E-03 | 2.07E-02 |
| Etv6 | 633.19 | -1.00 | 0.26 | -3.82 | 1.34E-04 | 1.95E-03 |
| Agt | 166.09 | -1.00 | 0.33 | -3.01 | 2.62E-03 | 2.48E-02 |
| Slc7a8 | 163.35 | -1.00 | 0.34 | -2.95 | 3.17E-03 | 2.88E-02 |
| Coro1a | 269.03 | -1.00 | 0.35 | -2.87 | 4.14E-03 | 3.61E-02 |
| Marchf3 | 210.22 | -1.00 | 0.28 | -3.57 | 3.53E-04 | 4.55E-03 |
| Ep400 | 1573.57 | -1.00 | 0.27 | -3.68 | 2.33E-04 | 3.18E-03 |
| Plec | 16812.50 | -1.01 | 0.15 | -6.67 | 2.48E-11 | 1.16E-09 |
| Irgm2 | 2103.59 | -1.01 | 0.22 | -4.48 | 7.33E-06 | 1.49E-04 |
| Bach1 | 2677.30 | -1.01 | 0.17 | -5.82 | 6.02E-09 | 2.09E-07 |
| Il10ra | 285.25 | -1.01 | 0.25 | -3.98 | 6.79E-05 | 1.07E-03 |
| Lilrb4a | 1607.96 | -1.01 | 0.24 | -4.15 | 3.31E-05 | 5.70E-04 |
| Gm10160 | 183.18 | -1.01 | 0.41 | -2.45 | 1.41E-02 | 9.42E-02 |
| Adgrg1 | 2555.22 | -1.01 | 0.19 | -5.46 | 4.70E-08 | 1.43E-06 |
| Mmp3 | 292.51 | -1.01 | 0.36 | -2.78 | 5.37E-03 | 4.44E-02 |
| Cstf3 | 501.47 | -1.02 | 0.29 | -3.48 | 5.10E-04 | 6.28E-03 |
| Samd9l | 971.91 | -1.02 | 0.29 | -3.45 | 5.56E-04 | 6.81E-03 |
| Ppp1r15b | 1655.27 | -1.02 | 0.20 | -5.15 | 2.56E-07 | 6.84E-06 |
| Shank3 | 719.50 | -1.02 | 0.28 | -3.65 | 2.60E-04 | 3.49E-03 |
| Nup98 | 1904.51 | -1.02 | 0.18 | -5.58 | 2.38E-08 | 7.62E-07 |
| Tob1 | 2630.31 | -1.02 | 0.27 | -3.74 | 1.82E-04 | 2.56E-03 |
| Foxc2 | 112.34 | -1.02 | 0.35 | -2.89 | 3.84E-03 | 3.39E-02 |
| Gnl3 | 991.48 | -1.02 | 0.25 | -4.13 | 3.67E-05 | 6.20E-04 |
| Sptlc2 | 876.95 | -1.03 | 0.26 | -3.93 | 8.32E-05 | 1.29E-03 |
| Igtp | 1638.65 | -1.03 | 0.27 | -3.77 | 1.63E-04 | 2.31E-03 |
| St3gal1 | 1733.66 | -1.03 | 0.22 | -4.79 | 1.66E-06 | 3.77E-05 |
| Abi1 | 1188.37 | -1.03 | 0.21 | -4.89 | 1.00E-06 | 2.37E-05 |
| Lgi1 | 121.90 | -1.03 | 0.35 | -2.97 | 3.00E-03 | 2.75E-02 |
| Fcer1g | 255.31 | -1.03 | 0.33 | -3.17 | 1.52E-03 | 1.59E-02 |
| Rabgef1 | 1131.90 | -1.03 | 0.20 | -5.09 | 3.60E-07 | 9.40E-06 |
| Fam110a | 89.72 | -1.03 | 0.41 | -2.50 | 1.25E-02 | 8.63E-02 |
| Ltv1 | 586.96 | -1.04 | 0.22 | -4.69 | 2.68E-06 | 5.90E-05 |
| Eif5 | 6223.78 | -1.04 | 0.17 | -6.10 | 1.04E-09 | 3.87E-08 |
| Sdcbp | 3783.31 | -1.04 | 0.21 | -5.01 | 5.31E-07 | 1.34E-05 |
| Cenpa | 1424.26 | -1.04 | 0.19 | -5.49 | 3.97E-08 | 1.22E-06 |
| Tubb4a | 155.32 | -1.04 | 0.38 | -2.72 | 6.47E-03 | 5.14E-02 |
| Plxna2 | 2336.40 | -1.04 | 0.19 | -5.58 | 2.45E-08 | 7.81E-07 |
| Hectd2 | 134.29 | -1.05 | 0.36 | -2.88 | 3.97E-03 | 3.49E-02 |
| Il3ra | 158.67 | -1.05 | 0.33 | -3.14 | 1.71E-03 | 1.77E-02 |
| Irf2bp2 | 2511.52 | -1.05 | 0.24 | -4.46 | 8.15E-06 | 1.64E-04 |
| Myh9 | 8056.40 | -1.05 | 0.15 | -6.96 | 3.39E-12 | 1.83E-10 |
| Daxx | 96.10 | -1.05 | 0.42 | -2.52 | 1.16E-02 | 8.19E-02 |
| Ugcg | 851.34 | -1.05 | 0.22 | -4.71 | 2.50E-06 | 5.54E-05 |
| Xiap | 2010.05 | -1.05 | 0.21 | -5.03 | 4.94E-07 | 1.25E-05 |
| Ppp1r9b | 2077.83 | -1.05 | 0.25 | -4.24 | 2.24E-05 | 4.04E-04 |
| Loxl2 | 1172.79 | -1.05 | 0.23 | -4.67 | 2.95E-06 | 6.44E-05 |
| Srpx | 197.21 | -1.05 | 0.43 | -2.43 | 1.50E-02 | 9.88E-02 |
| Tm4sf1 | 2984.39 | -1.05 | 0.33 | -3.21 | 1.31E-03 | 1.42E-02 |
| Kif21a | 205.02 | -1.05 | 0.34 | -3.13 | 1.75E-03 | 1.80E-02 |
| Ddx21 | 2407.88 | -1.06 | 0.19 | -5.45 | 5.18E-08 | 1.56E-06 |
| Zbtb11 | 786.94 | -1.06 | 0.20 | -5.42 | 5.87E-08 | 1.74E-06 |
| Gbp3 | 1559.31 | -1.06 | 0.29 | -3.70 | 2.16E-04 | 2.98E-03 |
| Pawr | 170.25 | -1.06 | 0.31 | -3.43 | 6.04E-04 | 7.28E-03 |
| Vcan | 781.58 | -1.06 | 0.28 | -3.82 | 1.32E-04 | 1.94E-03 |
| Thrsp | 434.75 | -1.06 | 0.23 | -4.68 | 2.83E-06 | 6.20E-05 |
| Dtx3l | 1004.84 | -1.06 | 0.28 | -3.80 | 1.42E-04 | 2.05E-03 |
| Pgm2 | 504.77 | -1.06 | 0.32 | -3.38 | 7.32E-04 | 8.59E-03 |
| C3ar1 | 402.55 | -1.06 | 0.34 | -3.17 | 1.51E-03 | 1.59E-02 |
| Ms4a6c | 187.99 | -1.07 | 0.30 | -3.56 | 3.65E-04 | 4.69E-03 |
| Paqr8 | 116.55 | -1.07 | 0.41 | -2.60 | 9.34E-03 | 6.94E-02 |
| Myot | 571.37 | -1.07 | 0.28 | -3.87 | 1.10E-04 | 1.65E-03 |
| Cachd1 | 215.07 | -1.07 | 0.29 | -3.67 | 2.42E-04 | 3.27E-03 |
| Sde2 | 1117.52 | -1.07 | 0.27 | -4.02 | 5.94E-05 | 9.56E-04 |
| Ahrr | 115.77 | -1.08 | 0.43 | -2.52 | 1.18E-02 | 8.29E-02 |
| Msx1 | 253.90 | -1.08 | 0.31 | -3.43 | 6.04E-04 | 7.28E-03 |
| Iqgap1 | 3047.56 | -1.08 | 0.20 | -5.31 | 1.09E-07 | 3.08E-06 |
| Clic1 | 1321.16 | -1.08 | 0.33 | -3.23 | 1.25E-03 | 1.36E-02 |
| Stx6 | 726.17 | -1.08 | 0.23 | -4.68 | 2.91E-06 | 6.38E-05 |
| Kit | 185.84 | -1.08 | 0.35 | -3.06 | 2.21E-03 | 2.17E-02 |
| Dennd3 | 796.06 | -1.08 | 0.30 | -3.62 | 2.95E-04 | 3.92E-03 |
| Ace | 1831.50 | -1.08 | 0.22 | -4.95 | 7.34E-07 | 1.80E-05 |
| Pspc1 | 484.11 | -1.08 | 0.27 | -3.99 | 6.56E-05 | 1.04E-03 |
| Pml | 873.23 | -1.09 | 0.27 | -4.06 | 4.96E-05 | 8.10E-04 |
| Btg1 | 3009.01 | -1.09 | 0.26 | -4.14 | 3.47E-05 | 5.93E-04 |
| Arhgap23 | 1495.62 | -1.09 | 0.31 | -3.56 | 3.77E-04 | 4.82E-03 |
| Lrrc8d | 362.18 | -1.10 | 0.30 | -3.68 | 2.33E-04 | 3.18E-03 |
| Stat3 | 8865.60 | -1.10 | 0.19 | -5.71 | 1.12E-08 | 3.74E-07 |
| Zc3h12c | 376.35 | -1.10 | 0.26 | -4.19 | 2.82E-05 | 4.96E-04 |
| Casp3 | 158.72 | -1.10 | 0.35 | -3.12 | 1.83E-03 | 1.87E-02 |
| Fbn1 | 2838.19 | -1.10 | 0.19 | -5.66 | 1.49E-08 | 4.88E-07 |
| H2-K1 | 2610.39 | -1.10 | 0.22 | -4.93 | 8.29E-07 | 2.02E-05 |
| Nhsl2 | 292.43 | -1.11 | 0.33 | -3.35 | 8.01E-04 | 9.27E-03 |
| Trim30d | 196.30 | -1.11 | 0.42 | -2.66 | 7.71E-03 | 5.92E-02 |
| Rai14 | 251.20 | -1.11 | 0.26 | -4.31 | 1.66E-05 | 3.10E-04 |
| Mex3c | 723.31 | -1.11 | 0.22 | -5.15 | 2.54E-07 | 6.81E-06 |
| Ncoa7 | 368.60 | -1.11 | 0.42 | -2.67 | 7.53E-03 | 5.80E-02 |
| Gm29216 | 203898.04 | -1.12 | 0.21 | -5.43 | 5.70E-08 | 1.70E-06 |
| Trim30a | 912.92 | -1.12 | 0.29 | -3.84 | 1.25E-04 | 1.84E-03 |
| Vav1 | 144.66 | -1.12 | 0.34 | -3.26 | 1.13E-03 | 1.24E-02 |
| Creb1 | 994.08 | -1.12 | 0.20 | -5.61 | 2.07E-08 | 6.70E-07 |
| Pde1b | 112.48 | -1.12 | 0.36 | -3.10 | 1.93E-03 | 1.95E-02 |
| Ikbke | 180.54 | -1.12 | 0.41 | -2.71 | 6.69E-03 | 5.28E-02 |
| Ehd1 | 4748.64 | -1.12 | 0.19 | -5.76 | 8.26E-09 | 2.78E-07 |
| Phf11d | 630.61 | -1.12 | 0.30 | -3.77 | 1.62E-04 | 2.31E-03 |
| Cbx4 | 735.76 | -1.13 | 0.22 | -5.01 | 5.39E-07 | 1.35E-05 |
| Edem1 | 687.24 | -1.13 | 0.23 | -4.86 | 1.20E-06 | 2.80E-05 |
| Dusp8 | 2415.29 | -1.13 | 0.19 | -5.90 | 3.55E-09 | 1.26E-07 |
| Nkain1 | 140.36 | -1.13 | 0.45 | -2.53 | 1.13E-02 | 8.06E-02 |
| Slc20a1 | 1867.37 | -1.13 | 0.25 | -4.53 | 6.00E-06 | 1.24E-04 |
| Acsl4 | 882.87 | -1.14 | 0.25 | -4.52 | 6.16E-06 | 1.27E-04 |
| Zfp703 | 1249.52 | -1.14 | 0.18 | -6.16 | 7.16E-10 | 2.76E-08 |
| Kcne4 | 314.41 | -1.14 | 0.41 | -2.77 | 5.54E-03 | 4.55E-02 |
| N4bp3 | 537.34 | -1.14 | 0.30 | -3.84 | 1.23E-04 | 1.81E-03 |
| Spi1 | 300.45 | -1.14 | 0.30 | -3.85 | 1.20E-04 | 1.78E-03 |
| Dyrk2 | 2220.05 | -1.15 | 0.21 | -5.46 | 4.80E-08 | 1.45E-06 |
| Nfya | 1093.00 | -1.15 | 0.18 | -6.29 | 3.15E-10 | 1.27E-08 |
| Fbxo30 | 1465.44 | -1.15 | 0.17 | -6.61 | 3.73E-11 | 1.71E-09 |
| Psat1 | 95.30 | -1.15 | 0.45 | -2.55 | 1.06E-02 | 7.67E-02 |
| Gm8995 | 1786.33 | -1.15 | 0.31 | -3.77 | 1.62E-04 | 2.30E-03 |
| Ets1 | 3015.43 | -1.15 | 0.26 | -4.42 | 9.86E-06 | 1.95E-04 |
| Egr3 | 3118.50 | -1.15 | 0.26 | -4.49 | 7.04E-06 | 1.43E-04 |
| Apaf1 | 725.04 | -1.16 | 0.24 | -4.89 | 1.02E-06 | 2.39E-05 |
| Dcun1d3 | 500.49 | -1.16 | 0.29 | -3.95 | 7.74E-05 | 1.20E-03 |
| Irf9 | 1414.03 | -1.16 | 0.19 | -6.10 | 1.09E-09 | 4.06E-08 |
| Eif4a-ps4 | 1279.68 | -1.16 | 0.26 | -4.47 | 7.78E-06 | 1.57E-04 |
| Rap1b | 3803.14 | -1.16 | 0.21 | -5.58 | 2.44E-08 | 7.79E-07 |
| Cdr2 | 589.76 | -1.16 | 0.22 | -5.27 | 1.37E-07 | 3.82E-06 |
| Xaf1 | 608.05 | -1.16 | 0.27 | -4.25 | 2.17E-05 | 3.94E-04 |
| Spred1 | 1498.81 | -1.16 | 0.23 | -5.00 | 5.72E-07 | 1.43E-05 |
| Mxd1 | 620.13 | -1.17 | 0.23 | -4.98 | 6.26E-07 | 1.55E-05 |
| Ptpn23 | 1670.08 | -1.17 | 0.23 | -5.05 | 4.47E-07 | 1.15E-05 |
| Bcl2l11 | 1132.24 | -1.17 | 0.18 | -6.55 | 5.85E-11 | 2.59E-09 |
| Pwwp3b | 99.80 | -1.18 | 0.36 | -3.26 | 1.10E-03 | 1.23E-02 |
| Nupr1 | 425.09 | -1.18 | 0.33 | -3.56 | 3.76E-04 | 4.82E-03 |
| Slc25a36 | 2123.16 | -1.18 | 0.18 | -6.59 | 4.36E-11 | 1.97E-09 |
| Rnf19b | 1811.92 | -1.18 | 0.20 | -5.86 | 4.67E-09 | 1.63E-07 |
| Rhoj | 1749.44 | -1.18 | 0.27 | -4.43 | 9.45E-06 | 1.88E-04 |
| Yrdc | 529.21 | -1.18 | 0.25 | -4.68 | 2.93E-06 | 6.40E-05 |
| Col12a1 | 92.70 | -1.18 | 0.49 | -2.43 | 1.50E-02 | 9.90E-02 |
| Actb | 17515.21 | -1.19 | 0.20 | -5.88 | 4.20E-09 | 1.48E-07 |
| Srgn | 1347.02 | -1.19 | 0.30 | -4.01 | 6.17E-05 | 9.87E-04 |
| Ptgds | 3361.12 | -1.20 | 0.22 | -5.46 | 4.86E-08 | 1.47E-06 |
| Carmil1 | 156.00 | -1.20 | 0.32 | -3.71 | 2.09E-04 | 2.89E-03 |
| Dbn1 | 390.07 | -1.20 | 0.27 | -4.37 | 1.26E-05 | 2.41E-04 |
| Gngt2 | 328.21 | -1.20 | 0.46 | -2.60 | 9.29E-03 | 6.91E-02 |
| Ptprc | 445.56 | -1.20 | 0.25 | -4.77 | 1.88E-06 | 4.24E-05 |
| Gabpb1 | 621.06 | -1.20 | 0.23 | -5.12 | 3.09E-07 | 8.13E-06 |
| Ccrl2 | 1191.47 | -1.20 | 0.28 | -4.27 | 1.95E-05 | 3.60E-04 |
| Gm4070 | 2659.70 | -1.20 | 0.29 | -4.13 | 3.64E-05 | 6.18E-04 |
| Tanc2 | 385.56 | -1.20 | 0.29 | -4.08 | 4.42E-05 | 7.29E-04 |
| Mgat4a | 692.70 | -1.20 | 0.22 | -5.43 | 5.67E-08 | 1.70E-06 |
| H2-Q7 | 3098.22 | -1.21 | 0.18 | -6.58 | 4.72E-11 | 2.13E-09 |
| S100a10 | 1618.53 | -1.21 | 0.28 | -4.39 | 1.14E-05 | 2.22E-04 |
| Etv3 | 2123.42 | -1.21 | 0.17 | -7.18 | 7.07E-13 | 4.19E-11 |
| Ldlr | 535.42 | -1.21 | 0.25 | -4.93 | 8.02E-07 | 1.96E-05 |
| Dusp4 | 178.42 | -1.22 | 0.46 | -2.63 | 8.59E-03 | 6.49E-02 |
| Kif23 | 86.98 | -1.22 | 0.44 | -2.78 | 5.50E-03 | 4.52E-02 |
| Herc6 | 711.19 | -1.22 | 0.37 | -3.30 | 9.59E-04 | 1.08E-02 |
| Prkcd | 725.29 | -1.22 | 0.26 | -4.71 | 2.48E-06 | 5.50E-05 |
| Skil | 1709.97 | -1.22 | 0.20 | -6.11 | 1.01E-09 | 3.77E-08 |
| Fam111a | 347.43 | -1.22 | 0.38 | -3.24 | 1.18E-03 | 1.30E-02 |
| Cflar | 3332.97 | -1.22 | 0.17 | -7.08 | 1.42E-12 | 8.16E-11 |
| Ppard | 87.04 | -1.22 | 0.46 | -2.66 | 7.70E-03 | 5.92E-02 |
| Rin3 | 770.76 | -1.23 | 0.25 | -4.93 | 8.07E-07 | 1.97E-05 |
| Fam222a | 126.35 | -1.23 | 0.33 | -3.68 | 2.34E-04 | 3.19E-03 |
| Osgin1 | 375.52 | -1.23 | 0.32 | -3.79 | 1.50E-04 | 2.15E-03 |
| Chd1 | 1274.23 | -1.23 | 0.18 | -6.70 | 2.04E-11 | 9.63E-10 |
| Hmcn2 | 1617.79 | -1.23 | 0.25 | -4.89 | 1.03E-06 | 2.42E-05 |
| Trim16 | 406.34 | -1.23 | 0.31 | -3.92 | 8.81E-05 | 1.35E-03 |
| Rhob | 12210.91 | -1.23 | 0.21 | -5.87 | 4.37E-09 | 1.54E-07 |
| Foxm1 | 48.96 | -1.23 | 0.51 | -2.43 | 1.50E-02 | 9.90E-02 |
| Alpk1 | 304.10 | -1.24 | 0.28 | -4.39 | 1.15E-05 | 2.23E-04 |
| Nuak1 | 1255.68 | -1.24 | 0.19 | -6.56 | 5.47E-11 | 2.44E-09 |
| Ifi203 | 2609.47 | -1.25 | 0.23 | -5.44 | 5.41E-08 | 1.62E-06 |
| Rtn4rl2 | 192.44 | -1.25 | 0.39 | -3.17 | 1.54E-03 | 1.61E-02 |
| Rcl1 | 231.84 | -1.25 | 0.32 | -3.96 | 7.58E-05 | 1.19E-03 |
| Sec24a | 732.15 | -1.25 | 0.20 | -6.29 | 3.21E-10 | 1.28E-08 |
| Jund | 13029.05 | -1.25 | 0.21 | -6.10 | 1.07E-09 | 3.98E-08 |
| Irf5 | 253.01 | -1.26 | 0.27 | -4.61 | 3.99E-06 | 8.54E-05 |
| Sat1 | 3542.80 | -1.26 | 0.30 | -4.23 | 2.35E-05 | 4.21E-04 |
| Hcls1 | 382.46 | -1.26 | 0.28 | -4.53 | 5.90E-06 | 1.23E-04 |
| Crlf2 | 359.76 | -1.26 | 0.29 | -4.36 | 1.32E-05 | 2.50E-04 |
| Elf1 | 1458.33 | -1.26 | 0.23 | -5.40 | 6.55E-08 | 1.93E-06 |
| Cdt1 | 88.76 | -1.26 | 0.48 | -2.61 | 9.07E-03 | 6.78E-02 |
| Hsd11b1 | 326.74 | -1.26 | 0.29 | -4.36 | 1.31E-05 | 2.50E-04 |
| Nos3 | 2513.15 | -1.26 | 0.29 | -4.33 | 1.51E-05 | 2.84E-04 |
| Plk3 | 1180.05 | -1.26 | 0.23 | -5.41 | 6.17E-08 | 1.82E-06 |
| Plekhg1 | 603.47 | -1.27 | 0.24 | -5.19 | 2.15E-07 | 5.81E-06 |
| Tmem106a | 242.23 | -1.27 | 0.29 | -4.36 | 1.32E-05 | 2.50E-04 |
| Icosl | 886.02 | -1.27 | 0.25 | -5.04 | 4.75E-07 | 1.21E-05 |
| Zfp36l1 | 8011.95 | -1.27 | 0.26 | -4.88 | 1.04E-06 | 2.43E-05 |
| Tsku | 160.30 | -1.27 | 0.36 | -3.49 | 4.86E-04 | 6.01E-03 |
| Gas2l3 | 90.26 | -1.27 | 0.49 | -2.58 | 9.86E-03 | 7.23E-02 |
| Aoc2 | 116.09 | -1.28 | 0.39 | -3.31 | 9.17E-04 | 1.04E-02 |
| Cnn3 | 1991.92 | -1.28 | 0.19 | -6.81 | 1.00E-11 | 4.94E-10 |
| Chka | 488.68 | -1.28 | 0.21 | -5.96 | 2.51E-09 | 9.03E-08 |
| Ddx58 | 1123.64 | -1.28 | 0.21 | -6.14 | 8.31E-10 | 3.15E-08 |
| Itgb3 | 131.90 | -1.28 | 0.37 | -3.48 | 4.93E-04 | 6.08E-03 |
| Zfand2a | 937.43 | -1.28 | 0.26 | -4.96 | 7.06E-07 | 1.74E-05 |
| Cnnm4 | 448.86 | -1.28 | 0.22 | -5.87 | 4.40E-09 | 1.55E-07 |
| Plagl1 | 481.85 | -1.28 | 0.24 | -5.34 | 9.09E-08 | 2.60E-06 |
| Dusp1 | 7654.18 | -1.28 | 0.24 | -5.30 | 1.14E-07 | 3.21E-06 |
| Gna13 | 2198.48 | -1.28 | 0.18 | -7.20 | 6.16E-13 | 3.68E-11 |
| Mmp14 | 912.37 | -1.29 | 0.25 | -5.19 | 2.10E-07 | 5.69E-06 |
| Ptafr | 393.18 | -1.29 | 0.29 | -4.50 | 6.65E-06 | 1.36E-04 |
| Meox1 | 400.42 | -1.29 | 0.26 | -4.90 | 9.36E-07 | 2.25E-05 |
| Slc3a2 | 1959.34 | -1.29 | 0.28 | -4.57 | 4.95E-06 | 1.04E-04 |
| P2ry10b | 55.32 | -1.29 | 0.51 | -2.51 | 1.20E-02 | 8.40E-02 |
| Sash1 | 2828.74 | -1.29 | 0.19 | -6.94 | 3.91E-12 | 2.08E-10 |
| Apold1 | 4954.27 | -1.30 | 0.27 | -4.76 | 1.92E-06 | 4.32E-05 |
| Nrp2 | 2232.31 | -1.30 | 0.21 | -6.31 | 2.82E-10 | 1.15E-08 |
| Ankrd33b | 2020.15 | -1.30 | 0.17 | -7.46 | 8.98E-14 | 5.88E-12 |
| Fam107b | 430.50 | -1.30 | 0.33 | -3.99 | 6.60E-05 | 1.05E-03 |
| Lbp | 359.22 | -1.30 | 0.31 | -4.24 | 2.19E-05 | 3.97E-04 |
| Rc3h1 | 1544.96 | -1.30 | 0.21 | -6.09 | 1.12E-09 | 4.16E-08 |
| Utp14b | 129.47 | -1.30 | 0.36 | -3.58 | 3.37E-04 | 4.40E-03 |
| Swap70 | 1316.04 | -1.31 | 0.25 | -5.25 | 1.52E-07 | 4.21E-06 |
| Sirt1 | 1361.82 | -1.31 | 0.21 | -6.31 | 2.83E-10 | 1.15E-08 |
| Btg2 | 15821.00 | -1.32 | 0.29 | -4.53 | 5.82E-06 | 1.21E-04 |
| Nrn1 | 89.04 | -1.32 | 0.39 | -3.34 | 8.37E-04 | 9.64E-03 |
| Pdgfa | 1224.43 | -1.32 | 0.19 | -6.94 | 3.95E-12 | 2.09E-10 |
| Tfrc | 2008.65 | -1.32 | 0.29 | -4.64 | 3.55E-06 | 7.66E-05 |
| Slc66a2 | 1104.35 | -1.33 | 0.27 | -4.99 | 6.17E-07 | 1.54E-05 |
| Rap2b | 423.31 | -1.33 | 0.23 | -5.80 | 6.75E-09 | 2.33E-07 |
| Sgk1 | 3244.98 | -1.33 | 0.27 | -4.95 | 7.40E-07 | 1.82E-05 |
| Dbf4 | 74.77 | -1.33 | 0.45 | -2.93 | 3.38E-03 | 3.04E-02 |
| Rpl10a | 325.69 | -1.33 | 0.36 | -3.73 | 1.91E-04 | 2.67E-03 |
| Rassf1 | 1906.31 | -1.33 | 0.27 | -4.83 | 1.33E-06 | 3.09E-05 |
| Pstpip1 | 59.16 | -1.33 | 0.48 | -2.77 | 5.57E-03 | 4.57E-02 |
| Ncf4 | 85.62 | -1.33 | 0.44 | -3.02 | 2.49E-03 | 2.39E-02 |
| Gbp6 | 3206.66 | -1.34 | 0.26 | -5.13 | 2.88E-07 | 7.65E-06 |
| Hmgcr | 484.30 | -1.34 | 0.21 | -6.24 | 4.46E-10 | 1.75E-08 |
| Uhrf1 | 74.21 | -1.34 | 0.46 | -2.92 | 3.49E-03 | 3.12E-02 |
| Dpep2 | 59.06 | -1.34 | 0.54 | -2.49 | 1.29E-02 | 8.81E-02 |
| Noct | 15937.74 | -1.35 | 0.15 | -8.71 | 2.98E-18 | 3.02E-16 |
| Ednrb | 1317.82 | -1.35 | 0.26 | -5.25 | 1.52E-07 | 4.21E-06 |
| Hivep1 | 1666.03 | -1.35 | 0.17 | -7.82 | 5.24E-15 | 4.00E-13 |
| Ttc9 | 81.41 | -1.35 | 0.50 | -2.68 | 7.25E-03 | 5.62E-02 |
| Rab11fip1 | 133.47 | -1.35 | 0.39 | -3.45 | 5.66E-04 | 6.92E-03 |
| Pprc1 | 1112.90 | -1.36 | 0.21 | -6.61 | 3.93E-11 | 1.79E-09 |
| Elmsan1 | 1811.12 | -1.36 | 0.24 | -5.63 | 1.81E-08 | 5.89E-07 |
| Mmp12 | 65.21 | -1.36 | 0.53 | -2.58 | 9.85E-03 | 7.23E-02 |
| Ccnl1 | 3673.76 | -1.36 | 0.22 | -6.14 | 8.46E-10 | 3.18E-08 |
| Ralb | 1205.25 | -1.36 | 0.19 | -7.29 | 3.02E-13 | 1.88E-11 |
| Snx10 | 969.15 | -1.37 | 0.20 | -6.82 | 9.23E-12 | 4.58E-10 |
| Osbpl10 | 53.44 | -1.37 | 0.49 | -2.78 | 5.51E-03 | 4.53E-02 |
| Klf6 | 7737.38 | -1.38 | 0.17 | -8.15 | 3.72E-16 | 3.18E-14 |
| Slfn3 | 315.45 | -1.38 | 0.31 | -4.50 | 6.74E-06 | 1.38E-04 |
| Magt1 | 307.02 | -1.38 | 0.35 | -3.91 | 9.08E-05 | 1.39E-03 |
| Ntrk2 | 68.78 | -1.38 | 0.53 | -2.59 | 9.63E-03 | 7.10E-02 |
| Lcat | 87.45 | -1.38 | 0.42 | -3.30 | 9.79E-04 | 1.10E-02 |
| Inhbb | 215.61 | -1.38 | 0.43 | -3.18 | 1.47E-03 | 1.55E-02 |
| Usp27x | 104.86 | -1.40 | 0.46 | -3.02 | 2.50E-03 | 2.39E-02 |
| Pvr | 2001.19 | -1.40 | 0.24 | -5.78 | 7.31E-09 | 2.51E-07 |
| Slfn9 | 369.11 | -1.40 | 0.42 | -3.34 | 8.36E-04 | 9.64E-03 |
| Pdlim1 | 2642.99 | -1.40 | 0.22 | -6.52 | 7.18E-11 | 3.14E-09 |
| Fbxo33 | 487.95 | -1.41 | 0.25 | -5.61 | 2.05E-08 | 6.62E-07 |
| Plscr1 | 862.48 | -1.41 | 0.26 | -5.42 | 5.96E-08 | 1.77E-06 |
| Abcb1b | 460.56 | -1.41 | 0.28 | -4.98 | 6.21E-07 | 1.54E-05 |
| Katna1 | 753.55 | -1.41 | 0.23 | -6.16 | 7.30E-10 | 2.80E-08 |
| Igsf6 | 154.27 | -1.41 | 0.38 | -3.77 | 1.66E-04 | 2.34E-03 |
| Psd4 | 63.81 | -1.41 | 0.56 | -2.50 | 1.23E-02 | 8.53E-02 |
| Sox7 | 1661.40 | -1.41 | 0.20 | -7.13 | 1.02E-12 | 5.96E-11 |
| Kctd12 | 2773.48 | -1.41 | 0.17 | -8.56 | 1.12E-17 | 1.08E-15 |
| Rgs2 | 1797.31 | -1.42 | 0.24 | -5.79 | 7.11E-09 | 2.45E-07 |
| C5ar1 | 853.97 | -1.42 | 0.20 | -6.99 | 2.80E-12 | 1.53E-10 |
| Tsc22d2 | 1787.31 | -1.42 | 0.25 | -5.76 | 8.20E-09 | 2.77E-07 |
| Ugdh | 1652.37 | -1.42 | 0.36 | -3.97 | 7.07E-05 | 1.11E-03 |
| Ptpn12 | 1984.82 | -1.42 | 0.19 | -7.55 | 4.34E-14 | 2.95E-12 |
| Gas7 | 920.69 | -1.42 | 0.21 | -6.71 | 1.96E-11 | 9.26E-10 |
| H2-Q10 | 505.20 | -1.42 | 0.24 | -6.03 | 1.63E-09 | 5.98E-08 |
| Tbc1d9 | 329.08 | -1.43 | 0.28 | -5.08 | 3.70E-07 | 9.62E-06 |
| Trpv4 | 364.50 | -1.43 | 0.26 | -5.53 | 3.14E-08 | 9.83E-07 |
| Bcar1 | 1655.10 | -1.43 | 0.22 | -6.54 | 6.03E-11 | 2.66E-09 |
| Rhog | 726.31 | -1.43 | 0.26 | -5.43 | 5.69E-08 | 1.70E-06 |
| Rnf122 | 545.16 | -1.43 | 0.39 | -3.70 | 2.18E-04 | 2.99E-03 |
| Hspb1 | 17334.98 | -1.43 | 0.24 | -6.01 | 1.87E-09 | 6.79E-08 |
| Eda2r | 246.27 | -1.43 | 0.32 | -4.50 | 6.80E-06 | 1.39E-04 |
| Rnf213 | 3829.68 | -1.44 | 0.24 | -6.00 | 2.00E-09 | 7.21E-08 |
| Hspa2 | 460.48 | -1.44 | 0.31 | -4.70 | 2.58E-06 | 5.69E-05 |
| Snx20 | 90.85 | -1.44 | 0.41 | -3.47 | 5.25E-04 | 6.44E-03 |
| Ipmk | 1049.92 | -1.44 | 0.24 | -6.01 | 1.90E-09 | 6.90E-08 |
| Stk40 | 2850.47 | -1.44 | 0.21 | -7.00 | 2.58E-12 | 1.43E-10 |
| Myd88 | 1457.81 | -1.44 | 0.28 | -5.18 | 2.22E-07 | 5.99E-06 |
| Gale | 43.01 | -1.44 | 0.53 | -2.70 | 6.96E-03 | 5.45E-02 |
| Lrrc8b | 355.78 | -1.44 | 0.29 | -4.98 | 6.23E-07 | 1.55E-05 |
| Pdk4 | 31593.28 | -1.44 | 0.21 | -6.77 | 1.32E-11 | 6.43E-10 |
| Ptpn2 | 773.71 | -1.44 | 0.26 | -5.63 | 1.77E-08 | 5.78E-07 |
| Rasl11b | 1677.22 | -1.44 | 0.23 | -6.16 | 7.24E-10 | 2.79E-08 |
| Arhgap8 | 50.23 | -1.44 | 0.59 | -2.43 | 1.51E-02 | 9.91E-02 |
| Siglece | 61.95 | -1.44 | 0.54 | -2.69 | 7.16E-03 | 5.56E-02 |
| Chrnb1 | 217.81 | -1.45 | 0.29 | -5.03 | 4.86E-07 | 1.23E-05 |
| Simc1 | 243.79 | -1.45 | 0.29 | -5.06 | 4.26E-07 | 1.10E-05 |
| Pdlim7 | 2036.69 | -1.45 | 0.24 | -6.14 | 8.41E-10 | 3.18E-08 |
| Cdc42ep4 | 1012.69 | -1.45 | 0.34 | -4.28 | 1.85E-05 | 3.42E-04 |
| Ier3 | 6974.27 | -1.45 | 0.26 | -5.57 | 2.50E-08 | 7.89E-07 |
| Tnfrsf23 | 115.60 | -1.45 | 0.40 | -3.68 | 2.35E-04 | 3.20E-03 |
| Map2k3 | 3207.41 | -1.46 | 0.22 | -6.57 | 4.87E-11 | 2.19E-09 |
| Upk3b | 99.02 | -1.46 | 0.53 | -2.73 | 6.40E-03 | 5.10E-02 |
| Gadd45a | 1558.19 | -1.46 | 0.28 | -5.27 | 1.39E-07 | 3.86E-06 |
| Rasd1 | 608.26 | -1.46 | 0.29 | -5.02 | 5.11E-07 | 1.29E-05 |
| Frmd6 | 2136.41 | -1.46 | 0.22 | -6.50 | 8.20E-11 | 3.54E-09 |
| Dse | 394.77 | -1.46 | 0.36 | -4.09 | 4.41E-05 | 7.27E-04 |
| Exoc3l4 | 240.80 | -1.46 | 0.36 | -4.04 | 5.30E-05 | 8.59E-04 |
| Irf7 | 1152.67 | -1.46 | 0.27 | -5.51 | 3.60E-08 | 1.11E-06 |
| Ikzf1 | 144.27 | -1.47 | 0.43 | -3.41 | 6.60E-04 | 7.84E-03 |
| Abi3 | 421.49 | -1.47 | 0.35 | -4.24 | 2.27E-05 | 4.06E-04 |
| Nol10 | 537.23 | -1.47 | 0.34 | -4.36 | 1.31E-05 | 2.50E-04 |
| Spty2d1 | 1135.91 | -1.47 | 0.19 | -7.82 | 5.44E-15 | 4.13E-13 |
| Sh2b3 | 2343.60 | -1.47 | 0.25 | -5.90 | 3.66E-09 | 1.30E-07 |
| Ints6 | 850.38 | -1.47 | 0.31 | -4.78 | 1.71E-06 | 3.89E-05 |
| Eif6 | 1590.66 | -1.47 | 0.33 | -4.49 | 7.07E-06 | 1.44E-04 |
| Enc1 | 1205.03 | -1.48 | 0.27 | -5.38 | 7.27E-08 | 2.13E-06 |
| Lcp1 | 1419.53 | -1.48 | 0.19 | -7.58 | 3.41E-14 | 2.35E-12 |
| Uck2 | 1382.30 | -1.48 | 0.29 | -5.04 | 4.57E-07 | 1.17E-05 |
| Zfp36 | 21266.52 | -1.48 | 0.26 | -5.77 | 7.91E-09 | 2.69E-07 |
| H3f3b | 9901.44 | -1.48 | 0.25 | -5.91 | 3.46E-09 | 1.23E-07 |
| Zc3hav1l | 115.57 | -1.48 | 0.37 | -4.01 | 5.95E-05 | 9.56E-04 |
| Gnai3 | 1935.75 | -1.48 | 0.19 | -8.00 | 1.29E-15 | 1.04E-13 |
| Txnrd1 | 3916.18 | -1.48 | 0.25 | -6.01 | 1.86E-09 | 6.78E-08 |
| Itgb2 | 284.18 | -1.49 | 0.40 | -3.71 | 2.06E-04 | 2.86E-03 |
| Fzd5 | 554.98 | -1.49 | 0.25 | -6.05 | 1.46E-09 | 5.37E-08 |
| Eno2 | 101.25 | -1.49 | 0.55 | -2.73 | 6.37E-03 | 5.08E-02 |
| Foxp4 | 1076.85 | -1.49 | 0.21 | -6.99 | 2.83E-12 | 1.53E-10 |
| Npas2 | 135.13 | -1.49 | 0.41 | -3.62 | 2.90E-04 | 3.87E-03 |
| Cebpb | 3590.86 | -1.49 | 0.24 | -6.21 | 5.29E-10 | 2.06E-08 |
| Alkal2 | 80.66 | -1.50 | 0.44 | -3.40 | 6.69E-04 | 7.93E-03 |
| Kcnd1 | 41.12 | -1.50 | 0.56 | -2.69 | 7.10E-03 | 5.53E-02 |
| Bcl10 | 1321.50 | -1.50 | 0.22 | -6.84 | 7.74E-12 | 3.89E-10 |
| Nnmt | 158.21 | -1.50 | 0.51 | -2.96 | 3.09E-03 | 2.82E-02 |
| Ifitm3 | 3576.70 | -1.50 | 0.23 | -6.56 | 5.29E-11 | 2.37E-09 |
| Lmna | 10538.84 | -1.50 | 0.24 | -6.17 | 6.77E-10 | 2.62E-08 |
| Siah2 | 546.49 | -1.51 | 0.24 | -6.25 | 4.10E-10 | 1.61E-08 |
| Fosl2 | 9197.96 | -1.51 | 0.24 | -6.18 | 6.39E-10 | 2.48E-08 |
| Pakap | 12645.57 | -1.51 | 0.16 | -9.49 | 2.34E-21 | 3.03E-19 |
| Sowahc | 561.40 | -1.51 | 0.24 | -6.33 | 2.52E-10 | 1.03E-08 |
| Gramd1a | 2825.38 | -1.52 | 0.21 | -7.31 | 2.71E-13 | 1.70E-11 |
| Cytip | 47.22 | -1.52 | 0.55 | -2.75 | 6.01E-03 | 4.85E-02 |
| Malt1 | 80.39 | -1.52 | 0.48 | -3.16 | 1.57E-03 | 1.64E-02 |
| Slc4a7 | 271.13 | -1.52 | 0.27 | -5.59 | 2.25E-08 | 7.25E-07 |
| Egr2 | 2404.42 | -1.53 | 0.31 | -4.99 | 6.02E-07 | 1.50E-05 |
| Klhl25 | 358.37 | -1.53 | 0.27 | -5.72 | 1.09E-08 | 3.64E-07 |
| Ubald1 | 2200.84 | -1.53 | 0.27 | -5.59 | 2.31E-08 | 7.41E-07 |
| Myo1g | 210.11 | -1.53 | 0.32 | -4.83 | 1.38E-06 | 3.18E-05 |
| Ripk1 | 1340.78 | -1.53 | 0.27 | -5.77 | 8.16E-09 | 2.76E-07 |
| Mafk | 4353.99 | -1.53 | 0.22 | -6.86 | 7.12E-12 | 3.59E-10 |
| Tpm3 | 4748.56 | -1.54 | 0.29 | -5.27 | 1.35E-07 | 3.77E-06 |
| Midn | 6484.85 | -1.54 | 0.25 | -6.14 | 8.12E-10 | 3.09E-08 |
| Uap1 | 3846.04 | -1.54 | 0.31 | -4.92 | 8.78E-07 | 2.13E-05 |
| Fgl2 | 5509.58 | -1.54 | 0.32 | -4.81 | 1.53E-06 | 3.51E-05 |
| Gpr4 | 729.17 | -1.54 | 0.30 | -5.13 | 2.97E-07 | 7.86E-06 |
| Nuak2 | 703.56 | -1.55 | 0.22 | -7.17 | 7.48E-13 | 4.39E-11 |
| Cdh4 | 88.38 | -1.55 | 0.47 | -3.29 | 9.91E-04 | 1.11E-02 |
| Bcl6 | 1512.78 | -1.55 | 0.18 | -8.42 | 3.72E-17 | 3.41E-15 |
| Flnc | 22112.11 | -1.55 | 0.20 | -7.63 | 2.39E-14 | 1.68E-12 |
| Cd53 | 359.16 | -1.56 | 0.24 | -6.40 | 1.58E-10 | 6.51E-09 |
| Actg1 | 25747.85 | -1.56 | 0.24 | -6.37 | 1.86E-10 | 7.62E-09 |
| Creb3l1 | 258.96 | -1.56 | 0.36 | -4.30 | 1.69E-05 | 3.15E-04 |
| Flvcr1 | 453.24 | -1.56 | 0.24 | -6.61 | 3.83E-11 | 1.75E-09 |
| Rffl | 366.98 | -1.56 | 0.29 | -5.31 | 1.09E-07 | 3.08E-06 |
| Arid5b | 2804.67 | -1.56 | 0.31 | -5.12 | 3.06E-07 | 8.07E-06 |
| Tnfrsf10b | 983.82 | -1.57 | 0.30 | -5.28 | 1.29E-07 | 3.62E-06 |
| Tubb4b | 10209.69 | -1.57 | 0.18 | -8.76 | 2.04E-18 | 2.09E-16 |
| Zfp57 | 105.28 | -1.57 | 0.44 | -3.59 | 3.37E-04 | 4.40E-03 |
| Junb | 20622.48 | -1.57 | 0.29 | -5.36 | 8.32E-08 | 2.41E-06 |
| Mapk6 | 2202.64 | -1.58 | 0.18 | -8.84 | 9.39E-19 | 1.02E-16 |
| Mpp2 | 441.62 | -1.58 | 0.24 | -6.47 | 9.77E-11 | 4.15E-09 |
| Pde12 | 1106.56 | -1.58 | 0.31 | -5.14 | 2.81E-07 | 7.46E-06 |
| Neto2 | 67.55 | -1.58 | 0.44 | -3.62 | 2.91E-04 | 3.88E-03 |
| Adamts1 | 7984.47 | -1.58 | 0.28 | -5.67 | 1.42E-08 | 4.64E-07 |
| A3galt2 | 30.55 | -1.58 | 0.64 | -2.46 | 1.39E-02 | 9.32E-02 |
| Nfkbiz | 6861.24 | -1.58 | 0.19 | -8.48 | 2.22E-17 | 2.10E-15 |
| Anxa2 | 3319.67 | -1.58 | 0.32 | -4.89 | 1.02E-06 | 2.39E-05 |
| Nr4a3 | 6091.45 | -1.58 | 0.25 | -6.26 | 3.96E-10 | 1.56E-08 |
| Fhl3 | 355.38 | -1.59 | 0.26 | -6.12 | 9.54E-10 | 3.58E-08 |
| Ralgds | 1809.73 | -1.59 | 0.22 | -7.23 | 4.89E-13 | 2.96E-11 |
| Zswim4 | 1305.86 | -1.59 | 0.20 | -7.95 | 1.89E-15 | 1.50E-13 |
| Sh3bgrl2 | 277.18 | -1.59 | 0.41 | -3.87 | 1.07E-04 | 1.61E-03 |
| Apobr | 84.84 | -1.59 | 0.49 | -3.24 | 1.18E-03 | 1.29E-02 |
| Fam129b | 1825.14 | -1.60 | 0.33 | -4.90 | 9.56E-07 | 2.28E-05 |
| Ttyh1 | 61.67 | -1.60 | 0.53 | -3.01 | 2.62E-03 | 2.48E-02 |
| Flnb | 4429.01 | -1.60 | 0.23 | -6.82 | 9.23E-12 | 4.58E-10 |
| Erdr1 | 39.61 | -1.60 | 0.59 | -2.70 | 6.89E-03 | 5.41E-02 |
| Irgm1 | 2068.50 | -1.60 | 0.24 | -6.55 | 5.59E-11 | 2.48E-09 |
| Akap12 | 3545.60 | -1.61 | 0.21 | -7.67 | 1.75E-14 | 1.26E-12 |
| Prkcg | 1242.44 | -1.61 | 0.21 | -7.77 | 7.70E-15 | 5.76E-13 |
| Fscn1 | 1338.00 | -1.61 | 0.21 | -7.62 | 2.45E-14 | 1.72E-12 |
| Tgtp1 | 1299.59 | -1.61 | 0.30 | -5.37 | 7.68E-08 | 2.24E-06 |
| Fzd9 | 28.94 | -1.61 | 0.64 | -2.51 | 1.22E-02 | 8.46E-02 |
| Tap1 | 1542.04 | -1.61 | 0.27 | -5.95 | 2.65E-09 | 9.48E-08 |
| Gm18853 | 464.53 | -1.63 | 0.35 | -4.60 | 4.27E-06 | 9.07E-05 |
| Peg10 | 168.63 | -1.63 | 0.38 | -4.28 | 1.84E-05 | 3.42E-04 |
| Mki67 | 206.05 | -1.64 | 0.43 | -3.81 | 1.41E-04 | 2.05E-03 |
| Vasn | 982.71 | -1.65 | 0.31 | -5.34 | 9.33E-08 | 2.66E-06 |
| Scd4 | 336.27 | -1.65 | 0.30 | -5.46 | 4.70E-08 | 1.43E-06 |
| Tox2 | 76.16 | -1.65 | 0.43 | -3.87 | 1.11E-04 | 1.65E-03 |
| Myo1e | 772.14 | -1.65 | 0.21 | -8.01 | 1.16E-15 | 9.50E-14 |
| Lrrc32 | 1768.83 | -1.65 | 0.26 | -6.44 | 1.22E-10 | 5.12E-09 |
| Yeats2 | 110.73 | -1.65 | 0.36 | -4.60 | 4.18E-06 | 8.90E-05 |
| Rab8b | 1229.53 | -1.65 | 0.23 | -7.28 | 3.37E-13 | 2.08E-11 |
| Ier5 | 6119.03 | -1.65 | 0.28 | -5.90 | 3.73E-09 | 1.32E-07 |
| Fyb | 225.51 | -1.66 | 0.31 | -5.28 | 1.31E-07 | 3.67E-06 |
| Nfkbid | 536.54 | -1.66 | 0.22 | -7.43 | 1.12E-13 | 7.28E-12 |
| Rdh10 | 525.43 | -1.66 | 0.36 | -4.67 | 3.01E-06 | 6.55E-05 |
| Lilr4b | 1704.71 | -1.66 | 0.26 | -6.50 | 8.24E-11 | 3.54E-09 |
| Lratd1 | 77.61 | -1.67 | 0.42 | -3.96 | 7.63E-05 | 1.19E-03 |
| Prkd2 | 1974.59 | -1.67 | 0.18 | -9.35 | 8.49E-21 | 1.07E-18 |
| Oasl2 | 1160.38 | -1.67 | 0.28 | -6.03 | 1.64E-09 | 5.98E-08 |
| Crem | 717.91 | -1.68 | 0.39 | -4.31 | 1.61E-05 | 3.03E-04 |
| H2-Q4 | 5425.31 | -1.68 | 0.23 | -7.39 | 1.43E-13 | 9.30E-12 |
| Tlr2 | 1372.70 | -1.68 | 0.22 | -7.53 | 5.12E-14 | 3.42E-12 |
| Ccno | 120.88 | -1.68 | 0.41 | -4.10 | 4.21E-05 | 6.99E-04 |
| Rela | 3640.56 | -1.69 | 0.17 | -9.74 | 2.07E-22 | 3.01E-20 |
| Pde4b | 5774.48 | -1.69 | 0.25 | -6.66 | 2.68E-11 | 1.24E-09 |
| H2-Q5 | 836.76 | -1.69 | 0.23 | -7.26 | 3.95E-13 | 2.41E-11 |
| Plekho2 | 2417.50 | -1.69 | 0.27 | -6.31 | 2.79E-10 | 1.14E-08 |
| Sntb2 | 1661.15 | -1.69 | 0.23 | -7.39 | 1.49E-13 | 9.62E-12 |
| Plk2 | 3278.14 | -1.69 | 0.17 | -9.91 | 3.59E-23 | 5.82E-21 |
| Itga5 | 4678.44 | -1.69 | 0.25 | -6.79 | 1.10E-11 | 5.40E-10 |
| Cd44 | 1108.58 | -1.70 | 0.24 | -7.01 | 2.44E-12 | 1.36E-10 |
| Map3k14 | 611.45 | -1.70 | 0.20 | -8.37 | 5.76E-17 | 5.20E-15 |
| Panx1 | 174.18 | -1.70 | 0.41 | -4.17 | 3.10E-05 | 5.39E-04 |
| Wsb1 | 2768.55 | -1.70 | 0.20 | -8.49 | 2.15E-17 | 2.04E-15 |
| Bcr | 1255.82 | -1.70 | 0.61 | -2.78 | 5.46E-03 | 4.50E-02 |
| Sox9 | 166.10 | -1.71 | 0.41 | -4.12 | 3.73E-05 | 6.28E-04 |
| Irak2 | 963.38 | -1.71 | 0.19 | -8.86 | 8.12E-19 | 9.02E-17 |
| Irf1 | 9854.61 | -1.71 | 0.23 | -7.35 | 1.98E-13 | 1.26E-11 |
| Col7a1 | 72.29 | -1.72 | 0.49 | -3.51 | 4.40E-04 | 5.51E-03 |
| Hcar2 | 40.79 | -1.72 | 0.70 | -2.46 | 1.40E-02 | 9.38E-02 |
| Hsp90aa1 | 13197.84 | -1.72 | 0.18 | -9.34 | 9.28E-21 | 1.16E-18 |
| Nek6 | 244.72 | -1.72 | 0.36 | -4.82 | 1.47E-06 | 3.36E-05 |
| Foxf1 | 45.10 | -1.72 | 0.62 | -2.79 | 5.24E-03 | 4.35E-02 |
| Ell2 | 1076.61 | -1.72 | 0.21 | -8.19 | 2.69E-16 | 2.36E-14 |
| Fos | 17612.39 | -1.73 | 0.32 | -5.32 | 1.06E-07 | 3.00E-06 |
| Sema7a | 2796.98 | -1.73 | 0.21 | -8.14 | 4.02E-16 | 3.42E-14 |
| Zbtb46 | 1067.19 | -1.73 | 0.20 | -8.46 | 2.60E-17 | 2.39E-15 |
| Dnaja1 | 7647.31 | -1.73 | 0.24 | -7.17 | 7.34E-13 | 4.33E-11 |
| Nmrk2 | 388.77 | -1.74 | 0.43 | -4.01 | 6.20E-05 | 9.90E-04 |
| Irs2 | 6543.54 | -1.74 | 0.23 | -7.71 | 1.21E-14 | 8.83E-13 |
| Plat | 1455.77 | -1.74 | 0.26 | -6.69 | 2.21E-11 | 1.04E-09 |
| Hk3 | 99.54 | -1.74 | 0.48 | -3.67 | 2.44E-04 | 3.29E-03 |
| Adam8 | 75.35 | -1.75 | 0.46 | -3.77 | 1.66E-04 | 2.35E-03 |
| Crtc2 | 1779.36 | -1.75 | 0.26 | -6.63 | 3.32E-11 | 1.53E-09 |
| Spon2 | 153.77 | -1.75 | 0.49 | -3.54 | 3.93E-04 | 4.99E-03 |
| Aacs | 284.20 | -1.75 | 0.32 | -5.52 | 3.31E-08 | 1.02E-06 |
| Msr1 | 346.82 | -1.75 | 0.29 | -6.00 | 1.98E-09 | 7.15E-08 |
| Pxdc1 | 1360.58 | -1.75 | 0.28 | -6.15 | 7.57E-10 | 2.90E-08 |
| Jak2 | 2686.03 | -1.76 | 0.24 | -7.26 | 3.75E-13 | 2.30E-11 |
| Cx3cl1 | 731.90 | -1.76 | 0.27 | -6.41 | 1.48E-10 | 6.12E-09 |
| Isg20 | 293.25 | -1.76 | 0.33 | -5.37 | 7.98E-08 | 2.32E-06 |
| Map3k6 | 1480.86 | -1.76 | 0.28 | -6.40 | 1.51E-10 | 6.26E-09 |
| Zc3hav1 | 1953.13 | -1.76 | 0.19 | -9.21 | 3.14E-20 | 3.77E-18 |
| Nphp4 | 29.44 | -1.77 | 0.67 | -2.66 | 7.91E-03 | 6.05E-02 |
| Il1r1 | 1571.25 | -1.77 | 0.36 | -4.89 | 9.89E-07 | 2.35E-05 |
| Mob3c | 677.72 | -1.77 | 0.25 | -7.00 | 2.49E-12 | 1.39E-10 |
| Nop58 | 1619.12 | -1.77 | 0.30 | -5.81 | 6.07E-09 | 2.10E-07 |
| Zfp593 | 309.83 | -1.77 | 0.37 | -4.74 | 2.18E-06 | 4.88E-05 |
| Usp18 | 421.35 | -1.78 | 0.37 | -4.82 | 1.42E-06 | 3.25E-05 |
| Bmp2 | 306.53 | -1.78 | 0.27 | -6.53 | 6.71E-11 | 2.94E-09 |
| Slc39a6 | 318.03 | -1.78 | 0.27 | -6.53 | 6.67E-11 | 2.93E-09 |
| Cnksr1 | 149.36 | -1.78 | 0.53 | -3.38 | 7.15E-04 | 8.41E-03 |
| Lacc1 | 413.21 | -1.79 | 0.25 | -7.28 | 3.34E-13 | 2.07E-11 |
| Sesn2 | 428.89 | -1.79 | 0.35 | -5.09 | 3.64E-07 | 9.48E-06 |
| Gch1 | 1070.65 | -1.79 | 0.21 | -8.47 | 2.38E-17 | 2.21E-15 |
| Rasip1 | 4390.33 | -1.79 | 0.19 | -9.26 | 2.02E-20 | 2.46E-18 |
| Slc7a2 | 564.02 | -1.79 | 0.34 | -5.35 | 8.86E-08 | 2.55E-06 |
| Dnajb1 | 5820.67 | -1.80 | 0.25 | -7.20 | 6.19E-13 | 3.68E-11 |
| Frat2 | 531.90 | -1.80 | 0.33 | -5.40 | 6.71E-08 | 1.97E-06 |
| Cpxm2 | 388.57 | -1.80 | 0.31 | -5.73 | 1.00E-08 | 3.36E-07 |
| Dusp10 | 592.53 | -1.80 | 0.22 | -8.27 | 1.29E-16 | 1.15E-14 |
| Rab32 | 155.05 | -1.80 | 0.36 | -5.06 | 4.18E-07 | 1.08E-05 |
| Pilra | 46.68 | -1.80 | 0.65 | -2.76 | 5.79E-03 | 4.70E-02 |
| Tnfrsf11a | 158.96 | -1.81 | 0.35 | -5.12 | 3.12E-07 | 8.19E-06 |
| Helz2 | 5952.55 | -1.81 | 0.20 | -8.93 | 4.33E-19 | 4.94E-17 |
| Egr1 | 33080.74 | -1.81 | 0.24 | -7.52 | 5.47E-14 | 3.62E-12 |
| Mid1 | 470.16 | -1.81 | 0.27 | -6.64 | 3.14E-11 | 1.45E-09 |
| Ifi213 | 97.41 | -1.81 | 0.55 | -3.31 | 9.30E-04 | 1.05E-02 |
| Ccl19 | 126.10 | -1.81 | 0.44 | -4.12 | 3.79E-05 | 6.37E-04 |
| Rhbdf2 | 1746.56 | -1.82 | 0.17 | -10.52 | 6.93E-26 | 1.39E-23 |
| Cgas | 199.83 | -1.82 | 0.33 | -5.57 | 2.55E-08 | 8.05E-07 |
| Relt | 148.54 | -1.82 | 0.37 | -4.86 | 1.16E-06 | 2.71E-05 |
| Trim6 | 41.48 | -1.82 | 0.70 | -2.59 | 9.60E-03 | 7.08E-02 |
| Osm | 43.67 | -1.82 | 0.56 | -3.26 | 1.11E-03 | 1.23E-02 |
| Ms4a6d | 213.35 | -1.82 | 0.32 | -5.71 | 1.16E-08 | 3.84E-07 |
| Igf2bp2 | 227.83 | -1.83 | 0.36 | -5.04 | 4.65E-07 | 1.19E-05 |
| Baiap2 | 634.92 | -1.83 | 0.27 | -6.76 | 1.37E-11 | 6.59E-10 |
| Ifi207 | 1798.38 | -1.83 | 0.23 | -7.94 | 1.94E-15 | 1.53E-13 |
| Rnd3 | 1590.84 | -1.83 | 0.21 | -8.71 | 3.09E-18 | 3.11E-16 |
| Ubash3b | 297.27 | -1.84 | 0.45 | -4.09 | 4.39E-05 | 7.26E-04 |
| Cybb | 1448.15 | -1.84 | 0.25 | -7.27 | 3.56E-13 | 2.19E-11 |
| Flrt3 | 56.24 | -1.84 | 0.52 | -3.58 | 3.42E-04 | 4.43E-03 |
| Slc1a1 | 353.81 | -1.85 | 0.27 | -6.95 | 3.59E-12 | 1.91E-10 |
| Tnip3 | 410.55 | -1.85 | 0.24 | -7.55 | 4.40E-14 | 2.98E-12 |
| Ccn2 | 8540.15 | -1.85 | 0.23 | -8.13 | 4.46E-16 | 3.77E-14 |
| Ppp1r18 | 2359.19 | -1.85 | 0.20 | -9.32 | 1.19E-20 | 1.48E-18 |
| Dusp5 | 1929.53 | -1.86 | 0.23 | -8.16 | 3.38E-16 | 2.93E-14 |
| Nlrp3 | 1051.86 | -1.86 | 0.27 | -6.99 | 2.72E-12 | 1.49E-10 |
| Tgif1 | 756.90 | -1.86 | 0.28 | -6.67 | 2.54E-11 | 1.18E-09 |
| Arid5a | 4621.23 | -1.86 | 0.24 | -7.69 | 1.43E-14 | 1.04E-12 |
| Oas1a | 343.14 | -1.87 | 0.28 | -6.76 | 1.40E-11 | 6.72E-10 |
| Oas1b | 240.90 | -1.87 | 0.34 | -5.46 | 4.87E-08 | 1.47E-06 |
| Socs3 | 11226.21 | -1.87 | 0.32 | -5.77 | 7.75E-09 | 2.65E-07 |
| Tent5c | 143.50 | -1.87 | 0.35 | -5.34 | 9.31E-08 | 2.66E-06 |
| Ccn1 | 14793.66 | -1.87 | 0.23 | -7.98 | 1.46E-15 | 1.17E-13 |
| H2-Q6 | 3325.59 | -1.88 | 0.17 | -11.31 | 1.12E-29 | 2.90E-27 |
| Nts | 335.45 | -1.88 | 0.39 | -4.83 | 1.33E-06 | 3.09E-05 |
| Smad7 | 1291.97 | -1.88 | 0.24 | -7.69 | 1.52E-14 | 1.09E-12 |
| Cnmd | 40.90 | -1.88 | 0.57 | -3.31 | 9.17E-04 | 1.04E-02 |
| Gpr85 | 25.78 | -1.88 | 0.77 | -2.45 | 1.45E-02 | 9.63E-02 |
| Itpkc | 868.50 | -1.88 | 0.29 | -6.50 | 8.00E-11 | 3.47E-09 |
| Baz1a | 932.43 | -1.89 | 0.21 | -9.03 | 1.79E-19 | 2.08E-17 |
| Gm18852 | 385.34 | -1.89 | 0.38 | -5.00 | 5.62E-07 | 1.41E-05 |
| Dhx58 | 239.42 | -1.89 | 0.34 | -5.48 | 4.18E-08 | 1.28E-06 |
| Rel | 2175.16 | -1.89 | 0.18 | -10.24 | 1.26E-24 | 2.30E-22 |
| Kdm6b | 8223.37 | -1.89 | 0.19 | -9.90 | 4.16E-23 | 6.67E-21 |
| Gm9973 | 69.05 | -1.90 | 0.55 | -3.43 | 6.02E-04 | 7.27E-03 |
| Tgfb1 | 1616.14 | -1.90 | 0.23 | -8.10 | 5.37E-16 | 4.51E-14 |
| Gbp2 | 4010.95 | -1.90 | 0.24 | -8.01 | 1.15E-15 | 9.46E-14 |
| Basp1 | 119.24 | -1.90 | 0.40 | -4.78 | 1.71E-06 | 3.89E-05 |
| Srxn1 | 1350.11 | -1.90 | 0.30 | -6.28 | 3.46E-10 | 1.37E-08 |
| Jdp2 | 607.51 | -1.90 | 0.28 | -6.86 | 7.02E-12 | 3.57E-10 |
| Eif1a | 710.91 | -1.91 | 0.27 | -6.95 | 3.53E-12 | 1.89E-10 |
| Hsph1 | 4824.35 | -1.91 | 0.27 | -6.96 | 3.50E-12 | 1.88E-10 |
| Agpat4 | 384.37 | -1.91 | 0.34 | -5.59 | 2.25E-08 | 7.25E-07 |
| Rnf24 | 428.35 | -1.92 | 0.27 | -7.16 | 8.17E-13 | 4.78E-11 |
| Cmpk2 | 1235.04 | -1.92 | 0.64 | -2.98 | 2.85E-03 | 2.65E-02 |
| Gm8281 | 49.28 | -1.92 | 0.60 | -3.17 | 1.50E-03 | 1.59E-02 |
| Aldh1a2 | 331.94 | -1.92 | 0.30 | -6.44 | 1.17E-10 | 4.93E-09 |
| Phlda1 | 3211.82 | -1.92 | 0.33 | -5.85 | 4.85E-09 | 1.69E-07 |
| Piezo1 | 2632.44 | -1.93 | 0.27 | -7.09 | 1.37E-12 | 7.94E-11 |
| Cars | 906.21 | -1.93 | 0.31 | -6.15 | 7.79E-10 | 2.97E-08 |
| Slc5a3 | 311.61 | -1.93 | 0.26 | -7.54 | 4.64E-14 | 3.12E-12 |
| Odc1 | 5160.52 | -1.93 | 0.27 | -7.29 | 3.02E-13 | 1.88E-11 |
| Ankrd23 | 14126.70 | -1.93 | 0.23 | -8.34 | 7.63E-17 | 6.85E-15 |
| Oas3 | 57.73 | -1.94 | 0.51 | -3.82 | 1.33E-04 | 1.94E-03 |
| Tchh | 71.11 | -1.94 | 0.56 | -3.47 | 5.15E-04 | 6.33E-03 |
| Ngfr | 65.65 | -1.95 | 0.53 | -3.67 | 2.42E-04 | 3.27E-03 |
| Sertad1 | 1925.22 | -1.95 | 0.28 | -7.06 | 1.63E-12 | 9.29E-11 |
| Izumo1 | 82.25 | -1.95 | 0.44 | -4.39 | 1.11E-05 | 2.17E-04 |
| Nptx1 | 44.46 | -1.95 | 0.77 | -2.52 | 1.16E-02 | 8.19E-02 |
| Cebpd | 2444.19 | -1.95 | 0.33 | -5.87 | 4.26E-09 | 1.50E-07 |
| Upp1 | 324.62 | -1.95 | 0.29 | -6.84 | 7.91E-12 | 3.96E-10 |
| Parp14 | 4148.79 | -1.96 | 0.27 | -7.20 | 5.85E-13 | 3.52E-11 |
| Dennd4a | 1426.93 | -1.96 | 0.19 | -10.08 | 6.66E-24 | 1.13E-21 |
| Errfi1 | 5012.66 | -1.96 | 0.29 | -6.79 | 1.10E-11 | 5.40E-10 |
| Taf4b | 300.87 | -1.97 | 0.32 | -6.14 | 8.18E-10 | 3.11E-08 |
| Zmynd15 | 311.82 | -1.97 | 0.29 | -6.86 | 6.93E-12 | 3.53E-10 |
| Rbm47 | 66.06 | -1.97 | 0.47 | -4.20 | 2.69E-05 | 4.75E-04 |
| Wfdc17 | 298.87 | -1.97 | 0.35 | -5.71 | 1.14E-08 | 3.78E-07 |
| Slfn4 | 51.33 | -1.97 | 0.67 | -2.95 | 3.15E-03 | 2.87E-02 |
| Stap1 | 73.43 | -1.97 | 0.47 | -4.18 | 2.92E-05 | 5.11E-04 |
| Cd86 | 370.60 | -1.97 | 0.29 | -6.77 | 1.26E-11 | 6.14E-10 |
| Pnp | 1984.94 | -1.98 | 0.28 | -7.13 | 1.03E-12 | 5.97E-11 |
| Gem | 2488.46 | -1.98 | 0.26 | -7.62 | 2.51E-14 | 1.75E-12 |
| F2rl1 | 28.17 | -1.99 | 0.65 | -3.07 | 2.12E-03 | 2.10E-02 |
| Saa3 | 670.79 | -1.99 | 0.23 | -8.67 | 4.23E-18 | 4.20E-16 |
| Egfr | 807.80 | -1.99 | 0.29 | -6.87 | 6.58E-12 | 3.37E-10 |
| Rhoc | 4297.92 | -1.99 | 0.27 | -7.52 | 5.40E-14 | 3.59E-12 |
| Csf2rb | 237.34 | -2.00 | 0.34 | -5.85 | 4.94E-09 | 1.72E-07 |
| Nfkbia | 10300.25 | -2.00 | 0.20 | -9.81 | 1.04E-22 | 1.56E-20 |
| Sema3f | 1241.79 | -2.01 | 0.23 | -8.84 | 9.53E-19 | 1.03E-16 |
| H4f16 | 50.18 | -2.01 | 0.78 | -2.57 | 1.01E-02 | 7.37E-02 |
| Rgs16 | 1026.70 | -2.01 | 0.30 | -6.72 | 1.84E-11 | 8.77E-10 |
| Peli1 | 1896.23 | -2.01 | 0.19 | -10.53 | 6.27E-26 | 1.30E-23 |
| Lmcd1 | 2344.09 | -2.01 | 0.26 | -7.85 | 4.22E-15 | 3.24E-13 |
| Ptges | 111.81 | -2.01 | 0.38 | -5.25 | 1.53E-07 | 4.21E-06 |
| Gclc | 1275.23 | -2.02 | 0.19 | -10.73 | 7.10E-27 | 1.51E-24 |
| Gdf15 | 188.66 | -2.02 | 0.35 | -5.71 | 1.16E-08 | 3.84E-07 |
| Ppp1r15a | 7680.56 | -2.02 | 0.27 | -7.58 | 3.45E-14 | 2.37E-12 |
| Sema4c | 2874.90 | -2.02 | 0.23 | -8.71 | 2.93E-18 | 2.99E-16 |
| Dab2 | 2893.25 | -2.03 | 0.20 | -9.97 | 1.98E-23 | 3.24E-21 |
| Trib3 | 150.18 | -2.03 | 0.57 | -3.58 | 3.41E-04 | 4.43E-03 |
| Fosb | 24226.73 | -2.03 | 0.26 | -7.88 | 3.16E-15 | 2.44E-13 |
| Slc16a13 | 622.45 | -2.04 | 0.27 | -7.54 | 4.88E-14 | 3.27E-12 |
| 1500009L16Rik | 70.05 | -2.04 | 0.52 | -3.90 | 9.53E-05 | 1.45E-03 |
| Zfp954 | 334.57 | -2.05 | 0.35 | -5.77 | 7.83E-09 | 2.67E-07 |
| Ypel2 | 1248.26 | -2.05 | 0.32 | -6.43 | 1.29E-10 | 5.38E-09 |
| Irf8 | 865.14 | -2.05 | 0.32 | -6.50 | 8.13E-11 | 3.52E-09 |
| Azin1 | 4902.92 | -2.05 | 0.20 | -10.04 | 1.03E-23 | 1.73E-21 |
| Gm4202 | 65.85 | -2.05 | 0.65 | -3.15 | 1.62E-03 | 1.68E-02 |
| Clec4n | 155.88 | -2.05 | 0.39 | -5.34 | 9.50E-08 | 2.70E-06 |
| Cfb | 246.02 | -2.05 | 0.37 | -5.57 | 2.49E-08 | 7.88E-07 |
| Irak3 | 667.48 | -2.06 | 0.26 | -7.95 | 1.88E-15 | 1.50E-13 |
| Glis3 | 53.33 | -2.06 | 0.50 | -4.14 | 3.51E-05 | 5.98E-04 |
| Nfkb1 | 4128.54 | -2.07 | 0.22 | -9.60 | 7.84E-22 | 1.07E-19 |
| Slc16a3 | 339.16 | -2.07 | 0.45 | -4.59 | 4.39E-06 | 9.31E-05 |
| Lhfpl2 | 418.63 | -2.07 | 0.27 | -7.75 | 8.98E-15 | 6.64E-13 |
| Tnfaip2 | 8888.51 | -2.07 | 0.18 | -11.67 | 1.81E-31 | 4.97E-29 |
| F10 | 33.38 | -2.07 | 0.78 | -2.67 | 7.56E-03 | 5.82E-02 |
| Bcl2a1b | 295.70 | -2.07 | 0.32 | -6.46 | 1.02E-10 | 4.33E-09 |
| Xirp1 | 22308.32 | -2.08 | 0.23 | -9.17 | 4.57E-20 | 5.44E-18 |
| Sh3pxd2b | 822.20 | -2.09 | 0.24 | -8.81 | 1.30E-18 | 1.40E-16 |
| Bcl2a1a | 53.34 | -2.09 | 0.65 | -3.21 | 1.32E-03 | 1.43E-02 |
| Marcksl1 | 3140.46 | -2.10 | 0.18 | -11.75 | 7.14E-32 | 2.04E-29 |
| Adgrd1 | 739.18 | -2.10 | 0.25 | -8.39 | 4.77E-17 | 4.34E-15 |
| Emp1 | 6706.89 | -2.10 | 0.32 | -6.54 | 6.25E-11 | 2.75E-09 |
| Slfn2 | 1475.41 | -2.10 | 0.21 | -10.24 | 1.29E-24 | 2.34E-22 |
| Bcl3 | 1516.79 | -2.11 | 0.22 | -9.52 | 1.66E-21 | 2.21E-19 |
| Ifit3 | 2089.45 | -2.11 | 0.69 | -3.05 | 2.29E-03 | 2.24E-02 |
| Atf3 | 21240.57 | -2.12 | 0.27 | -7.90 | 2.83E-15 | 2.19E-13 |
| Tfec | 68.21 | -2.12 | 0.53 | -4.00 | 6.41E-05 | 1.02E-03 |
| Trib1 | 2662.41 | -2.13 | 0.25 | -8.48 | 2.26E-17 | 2.12E-15 |
| Runx1 | 363.76 | -2.14 | 0.28 | -7.63 | 2.34E-14 | 1.65E-12 |
| Trex1 | 1101.68 | -2.14 | 0.23 | -9.50 | 2.01E-21 | 2.66E-19 |
| Gm12250 | 165.18 | -2.15 | 0.49 | -4.38 | 1.20E-05 | 2.32E-04 |
| Sdc4 | 3588.36 | -2.15 | 0.27 | -7.91 | 2.60E-15 | 2.03E-13 |
| Acp5 | 126.93 | -2.16 | 0.34 | -6.39 | 1.62E-10 | 6.67E-09 |
| Klrg2 | 18.92 | -2.16 | 0.89 | -2.43 | 1.51E-02 | 9.91E-02 |
| Plekhh1 | 89.49 | -2.16 | 0.42 | -5.21 | 1.89E-07 | 5.15E-06 |
| Il4i1 | 51.27 | -2.17 | 0.61 | -3.58 | 3.41E-04 | 4.43E-03 |
| Rasgef1b | 2024.93 | -2.18 | 0.17 | -12.56 | 3.33E-36 | 1.10E-33 |
| Cd80 | 55.90 | -2.18 | 0.50 | -4.32 | 1.53E-05 | 2.88E-04 |
| Creb5 | 1057.41 | -2.19 | 0.31 | -6.99 | 2.75E-12 | 1.50E-10 |
| Ada | 54.00 | -2.19 | 0.57 | -3.84 | 1.25E-04 | 1.83E-03 |
| Prr5l | 125.67 | -2.20 | 0.51 | -4.29 | 1.76E-05 | 3.26E-04 |
| 2200002D01Rik | 59.64 | -2.20 | 0.53 | -4.18 | 2.98E-05 | 5.20E-04 |
| Batf2 | 102.67 | -2.20 | 0.51 | -4.30 | 1.74E-05 | 3.23E-04 |
| Ankrd42 | 34.03 | -2.21 | 0.69 | -3.19 | 1.44E-03 | 1.53E-02 |
| Fut2 | 36.32 | -2.21 | 0.64 | -3.45 | 5.69E-04 | 6.93E-03 |
| Fgr | 42.36 | -2.21 | 0.67 | -3.32 | 9.04E-04 | 1.03E-02 |
| Dusp6 | 1786.57 | -2.21 | 0.29 | -7.59 | 3.21E-14 | 2.22E-12 |
| Gm28661 | 9452.56 | -2.21 | 0.73 | -3.04 | 2.36E-03 | 2.29E-02 |
| Plek | 3042.47 | -2.22 | 0.16 | -13.50 | 1.65E-41 | 7.84E-39 |
| Acta1 | 3036.07 | -2.22 | 0.57 | -3.86 | 1.12E-04 | 1.66E-03 |
| Ramp3 | 216.30 | -2.22 | 0.34 | -6.49 | 8.42E-11 | 3.61E-09 |
| Star | 42.26 | -2.22 | 0.65 | -3.43 | 5.99E-04 | 7.24E-03 |
| 2010300C02Rik | 60.41 | -2.23 | 0.57 | -3.91 | 9.40E-05 | 1.43E-03 |
| Slamf7 | 182.06 | -2.23 | 0.46 | -4.87 | 1.10E-06 | 2.58E-05 |
| Zfp729a | 3008.46 | -2.23 | 0.18 | -12.21 | 2.87E-34 | 9.11E-32 |
| Pdpn | 539.21 | -2.24 | 0.39 | -5.77 | 8.08E-09 | 2.74E-07 |
| Orai2 | 170.04 | -2.24 | 0.38 | -5.96 | 2.55E-09 | 9.15E-08 |
| Vgll3 | 212.12 | -2.25 | 0.33 | -6.78 | 1.23E-11 | 6.02E-10 |
| Il1b | 1466.92 | -2.26 | 0.23 | -9.86 | 6.15E-23 | 9.44E-21 |
| Arrdc4 | 1626.70 | -2.26 | 0.31 | -7.39 | 1.50E-13 | 9.65E-12 |
| Gbp5 | 3208.35 | -2.26 | 0.28 | -8.16 | 3.43E-16 | 2.95E-14 |
| Tpbg | 101.91 | -2.27 | 0.50 | -4.51 | 6.57E-06 | 1.35E-04 |
| Gfpt2 | 4945.91 | -2.27 | 0.31 | -7.38 | 1.61E-13 | 1.03E-11 |
| Sp140 | 254.64 | -2.28 | 0.29 | -7.78 | 7.38E-15 | 5.54E-13 |
| Inhba | 412.12 | -2.28 | 0.82 | -2.76 | 5.70E-03 | 4.65E-02 |
| Acr | 30.54 | -2.28 | 0.67 | -3.43 | 6.09E-04 | 7.32E-03 |
| Rtp4 | 812.54 | -2.29 | 0.25 | -9.07 | 1.19E-19 | 1.40E-17 |
| Rgs1 | 111.40 | -2.29 | 0.49 | -4.65 | 3.39E-06 | 7.35E-05 |
| Mt2 | 6051.23 | -2.29 | 0.24 | -9.71 | 2.71E-22 | 3.91E-20 |
| Pgf | 585.37 | -2.29 | 0.33 | -6.91 | 4.72E-12 | 2.47E-10 |
| Osmr | 3562.83 | -2.29 | 0.23 | -9.90 | 4.27E-23 | 6.71E-21 |
| Areg | 100.03 | -2.30 | 0.43 | -5.38 | 7.57E-08 | 2.21E-06 |
| Epha2 | 1350.20 | -2.31 | 0.27 | -8.64 | 5.73E-18 | 5.60E-16 |
| Otud1 | 5415.98 | -2.31 | 0.26 | -8.77 | 1.79E-18 | 1.87E-16 |
| Ccr1 | 82.04 | -2.31 | 0.46 | -5.05 | 4.33E-07 | 1.11E-05 |
| Gm38431 | 62.91 | -2.31 | 0.50 | -4.58 | 4.56E-06 | 9.62E-05 |
| Sh2b2 | 79.86 | -2.31 | 0.58 | -3.99 | 6.64E-05 | 1.05E-03 |
| Tnf | 2737.78 | -2.31 | 0.18 | -13.10 | 3.16E-39 | 1.33E-36 |
| Pou3f1 | 44.84 | -2.32 | 0.58 | -4.02 | 5.75E-05 | 9.28E-04 |
| Ptprj | 913.69 | -2.32 | 0.26 | -8.84 | 9.34E-19 | 1.02E-16 |
| Wt1 | 889.21 | -2.33 | 0.21 | -11.28 | 1.58E-29 | 4.03E-27 |
| Zc3h12a | 2130.01 | -2.33 | 0.24 | -9.75 | 1.85E-22 | 2.73E-20 |
| Slc41a2 | 160.12 | -2.34 | 0.41 | -5.72 | 1.07E-08 | 3.56E-07 |
| Pou2f2 | 149.89 | -2.34 | 0.44 | -5.36 | 8.17E-08 | 2.37E-06 |
| Slc25a25 | 2370.11 | -2.34 | 0.19 | -12.08 | 1.27E-33 | 3.86E-31 |
| Cxcl16 | 1662.30 | -2.35 | 0.21 | -11.28 | 1.67E-29 | 4.19E-27 |
| Slfn8 | 534.23 | -2.36 | 0.34 | -6.91 | 4.82E-12 | 2.51E-10 |
| Serpine1 | 10647.87 | -2.36 | 0.22 | -10.88 | 1.37E-27 | 3.02E-25 |
| Lox | 190.77 | -2.36 | 0.38 | -6.22 | 4.90E-10 | 1.92E-08 |
| Klf5 | 76.55 | -2.37 | 0.43 | -5.55 | 2.92E-08 | 9.15E-07 |
| Gm42417 | 90.83 | -2.38 | 0.67 | -3.56 | 3.75E-04 | 4.80E-03 |
| Cxcl9 | 1750.43 | -2.38 | 0.35 | -6.88 | 5.87E-12 | 3.02E-10 |
| Oas1g | 123.60 | -2.39 | 0.41 | -5.78 | 7.69E-09 | 2.63E-07 |
| Gm49342 | 421.51 | -2.39 | 0.30 | -8.04 | 9.09E-16 | 7.50E-14 |
| Adamts9 | 2138.70 | -2.40 | 0.23 | -10.36 | 3.76E-25 | 7.25E-23 |
| Slfn10-ps | 27.93 | -2.40 | 0.74 | -3.24 | 1.19E-03 | 1.30E-02 |
| Vcam1 | 8656.19 | -2.41 | 0.17 | -14.06 | 6.85E-45 | 3.76E-42 |
| Plau | 1241.88 | -2.41 | 0.61 | -3.92 | 8.95E-05 | 1.37E-03 |
| Adgrg3 | 235.95 | -2.41 | 0.38 | -6.30 | 2.93E-10 | 1.18E-08 |
| Tnfrsf1b | 1130.97 | -2.42 | 0.27 | -8.86 | 8.15E-19 | 9.02E-17 |
| Il11 | 40.48 | -2.42 | 0.77 | -3.13 | 1.77E-03 | 1.82E-02 |
| Il4ra | 2816.27 | -2.43 | 0.27 | -8.92 | 4.67E-19 | 5.30E-17 |
| Hspa1b | 12721.47 | -2.43 | 0.23 | -10.43 | 1.75E-25 | 3.42E-23 |
| Slc44a5 | 51.94 | -2.43 | 0.58 | -4.17 | 3.10E-05 | 5.39E-04 |
| Dusp2 | 313.07 | -2.43 | 0.28 | -8.58 | 9.34E-18 | 9.07E-16 |
| Tnip1 | 3802.60 | -2.44 | 0.18 | -13.37 | 8.76E-41 | 3.91E-38 |
| Gm6377 | 461.03 | -2.45 | 0.33 | -7.31 | 2.61E-13 | 1.65E-11 |
| Tmem132e | 44.21 | -2.45 | 0.74 | -3.32 | 9.06E-04 | 1.03E-02 |
| Hspa1a | 15954.43 | -2.45 | 0.20 | -12.17 | 4.52E-34 | 1.40E-31 |
| Litaf | 3426.52 | -2.45 | 0.22 | -10.91 | 9.87E-28 | 2.20E-25 |
| Bcl2a1d | 121.99 | -2.46 | 0.61 | -4.01 | 6.04E-05 | 9.68E-04 |
| Tifa | 798.35 | -2.46 | 0.24 | -10.08 | 6.59E-24 | 1.13E-21 |
| Lcn2 | 205.94 | -2.48 | 0.33 | -7.48 | 7.19E-14 | 4.73E-12 |
| Nrip3 | 24.70 | -2.49 | 0.80 | -3.09 | 2.01E-03 | 2.01E-02 |
| Birc3 | 2936.26 | -2.50 | 0.18 | -14.21 | 8.39E-46 | 5.21E-43 |
| Bdkrb2 | 158.41 | -2.51 | 0.36 | -7.01 | 2.41E-12 | 1.35E-10 |
| Gadd45g | 4714.23 | -2.52 | 0.61 | -4.11 | 3.90E-05 | 6.52E-04 |
| Il17ra | 998.56 | -2.52 | 0.25 | -10.26 | 1.06E-24 | 1.96E-22 |
| Serpina3n | 1254.55 | -2.52 | 0.31 | -8.08 | 6.33E-16 | 5.29E-14 |
| Arntl | 415.16 | -2.52 | 0.36 | -7.09 | 1.38E-12 | 7.94E-11 |
| Ifi47 | 526.35 | -2.53 | 0.32 | -7.91 | 2.56E-15 | 2.01E-13 |
| Csf1 | 13135.81 | -2.53 | 0.18 | -13.92 | 4.58E-44 | 2.42E-41 |
| Gm49339 | 608.40 | -2.53 | 0.25 | -9.98 | 1.86E-23 | 3.09E-21 |
| Traf1 | 292.10 | -2.54 | 0.33 | -7.75 | 8.85E-15 | 6.58E-13 |
| Ccl7 | 3654.04 | -2.54 | 0.27 | -9.50 | 2.15E-21 | 2.82E-19 |
| Gm8752 | 45.65 | -2.54 | 0.61 | -4.16 | 3.13E-05 | 5.43E-04 |
| Cd40 | 511.44 | -2.54 | 0.24 | -10.53 | 6.39E-26 | 1.30E-23 |
| Rsad2 | 4022.96 | -2.55 | 0.59 | -4.29 | 1.76E-05 | 3.27E-04 |
| B4galt5 | 1795.01 | -2.55 | 0.23 | -11.16 | 6.67E-29 | 1.64E-26 |
| Slc15a3 | 848.94 | -2.56 | 0.25 | -10.18 | 2.42E-24 | 4.21E-22 |
| Ifih1 | 1808.68 | -2.56 | 0.27 | -9.44 | 3.89E-21 | 4.95E-19 |
| Gm8818 | 35.33 | -2.56 | 0.73 | -3.52 | 4.30E-04 | 5.40E-03 |
| Sphkap | 23.65 | -2.57 | 0.84 | -3.07 | 2.15E-03 | 2.12E-02 |
| Ccl11 | 426.05 | -2.58 | 0.37 | -7.06 | 1.69E-12 | 9.56E-11 |
| Ngf | 1287.78 | -2.58 | 0.27 | -9.69 | 3.38E-22 | 4.82E-20 |
| Gadd45b | 6046.56 | -2.58 | 0.27 | -9.68 | 3.62E-22 | 5.12E-20 |
| Relb | 1591.84 | -2.58 | 0.20 | -13.12 | 2.40E-39 | 1.04E-36 |
| Arl13b | 1831.83 | -2.59 | 0.25 | -10.34 | 4.73E-25 | 9.01E-23 |
| Adm | 732.61 | -2.59 | 0.24 | -10.99 | 4.50E-28 | 1.05E-25 |
| Dll1 | 564.84 | -2.60 | 0.34 | -7.73 | 1.07E-14 | 7.87E-13 |
| Ifi209 | 162.95 | -2.60 | 0.38 | -6.89 | 5.72E-12 | 2.95E-10 |
| Hmox1 | 2449.70 | -2.60 | 0.65 | -3.97 | 7.12E-05 | 1.12E-03 |
| Dyrk3 | 13.81 | -2.60 | 1.03 | -2.54 | 1.11E-02 | 7.94E-02 |
| Ccl12 | 192.35 | -2.62 | 0.40 | -6.60 | 4.05E-11 | 1.84E-09 |
| Ano5 | 81.47 | -2.62 | 0.64 | -4.11 | 4.02E-05 | 6.71E-04 |
| Samsn1 | 225.28 | -2.63 | 0.41 | -6.47 | 9.74E-11 | 4.15E-09 |
| Ccl9 | 1168.92 | -2.63 | 0.27 | -9.64 | 5.49E-22 | 7.61E-20 |
| Nppb | 2826.72 | -2.63 | 0.60 | -4.39 | 1.11E-05 | 2.17E-04 |
| Lcp2 | 533.08 | -2.64 | 0.26 | -10.19 | 2.24E-24 | 4.00E-22 |
| Trim30b | 32.43 | -2.64 | 0.81 | -3.25 | 1.14E-03 | 1.26E-02 |
| Myc | 2112.51 | -2.64 | 0.28 | -9.59 | 8.89E-22 | 1.20E-19 |
| Ccl8 | 63.21 | -2.64 | 0.62 | -4.24 | 2.26E-05 | 4.06E-04 |
| Csrnp1 | 5344.40 | -2.65 | 0.26 | -10.19 | 2.28E-24 | 4.03E-22 |
| Il2rg | 1332.01 | -2.65 | 0.18 | -14.39 | 6.20E-47 | 4.22E-44 |
| Gm45551 | 2851.70 | -2.66 | 0.22 | -11.92 | 9.50E-33 | 2.83E-30 |
| Fam83g | 49.33 | -2.66 | 0.69 | -3.85 | 1.18E-04 | 1.75E-03 |
| Klhl40 | 1731.09 | -2.66 | 0.32 | -8.21 | 2.31E-16 | 2.03E-14 |
| Tnfaip8l1 | 174.90 | -2.68 | 0.43 | -6.27 | 3.65E-10 | 1.45E-08 |
| Ptgdr | 15.73 | -2.69 | 0.92 | -2.91 | 3.59E-03 | 3.20E-02 |
| Ripk2 | 882.28 | -2.69 | 0.26 | -10.54 | 5.94E-26 | 1.25E-23 |
| Slc2a6 | 318.20 | -2.70 | 0.34 | -8.06 | 7.56E-16 | 6.27E-14 |
| Spdl1 | 27.22 | -2.70 | 0.76 | -3.58 | 3.49E-04 | 4.52E-03 |
| Ifi204 | 1459.55 | -2.71 | 0.23 | -11.69 | 1.52E-31 | 4.25E-29 |
| Pfkfb3 | 5713.60 | -2.71 | 0.16 | -16.73 | 8.26E-63 | 9.82E-60 |
| Kcnn4 | 29.32 | -2.71 | 0.75 | -3.61 | 3.01E-04 | 4.00E-03 |
| Il27 | 16.55 | -2.72 | 1.04 | -2.62 | 8.77E-03 | 6.60E-02 |
| Zbp1 | 153.56 | -2.72 | 0.41 | -6.60 | 4.12E-11 | 1.87E-09 |
| Car13 | 182.82 | -2.73 | 0.35 | -7.73 | 1.06E-14 | 7.79E-13 |
| Ccl22 | 55.60 | -2.74 | 0.67 | -4.07 | 4.68E-05 | 7.70E-04 |
| Hilpda | 396.86 | -2.74 | 0.87 | -3.16 | 1.60E-03 | 1.66E-02 |
| Prr7 | 230.57 | -2.74 | 0.36 | -7.55 | 4.22E-14 | 2.89E-12 |
| Sbno2 | 7917.65 | -2.74 | 0.30 | -9.24 | 2.39E-20 | 2.89E-18 |
| Pcdh10 | 16.11 | -2.74 | 0.95 | -2.90 | 3.79E-03 | 3.35E-02 |
| Micall2 | 254.27 | -2.75 | 0.30 | -9.27 | 1.87E-20 | 2.30E-18 |
| Cxcl2 | 6923.78 | -2.76 | 0.16 | -17.02 | 5.83E-65 | 8.32E-62 |
| Nfil3 | 1587.92 | -2.76 | 0.31 | -9.03 | 1.67E-19 | 1.96E-17 |
| Lonrf3 | 117.17 | -2.76 | 0.43 | -6.42 | 1.37E-10 | 5.72E-09 |
| Gprc5a | 237.18 | -2.77 | 0.41 | -6.71 | 1.92E-11 | 9.08E-10 |
| Mx2 | 994.79 | -2.77 | 0.68 | -4.05 | 5.15E-05 | 8.38E-04 |
| Olfr1033 | 5229.21 | -2.77 | 0.26 | -10.82 | 2.75E-27 | 5.94E-25 |
| Ddit4 | 2114.34 | -2.79 | 0.31 | -9.01 | 2.00E-19 | 2.31E-17 |
| Cxcl1 | 14546.18 | -2.80 | 0.19 | -14.48 | 1.62E-47 | 1.22E-44 |
| AC109138.2 | 3551.09 | -2.80 | 0.20 | -14.16 | 1.55E-45 | 9.21E-43 |
| Phf11b | 80.64 | -2.80 | 0.52 | -5.35 | 8.73E-08 | 2.51E-06 |
| Mthfd2 | 292.59 | -2.80 | 0.45 | -6.29 | 3.16E-10 | 1.27E-08 |
| Csf3r | 193.67 | -2.81 | 0.42 | -6.74 | 1.60E-11 | 7.64E-10 |
| Has2 | 413.06 | -2.83 | 0.40 | -7.06 | 1.70E-12 | 9.60E-11 |
| Adamts8 | 204.55 | -2.84 | 0.50 | -5.69 | 1.30E-08 | 4.29E-07 |
| Ifrd1 | 10640.46 | -2.85 | 0.23 | -12.36 | 4.46E-35 | 1.45E-32 |
| Syt5 | 29.47 | -2.85 | 0.70 | -4.06 | 4.87E-05 | 7.98E-04 |
| Pik3r5 | 646.54 | -2.87 | 0.25 | -11.36 | 6.30E-30 | 1.67E-27 |
| Ikzf4 | 373.01 | -2.89 | 0.42 | -6.89 | 5.67E-12 | 2.93E-10 |
| Batf | 173.60 | -2.89 | 0.39 | -7.33 | 2.33E-13 | 1.48E-11 |
| Mab21l3 | 21.33 | -2.89 | 0.86 | -3.35 | 8.11E-04 | 9.37E-03 |
| Ifi206 | 72.67 | -2.90 | 0.58 | -4.97 | 6.79E-07 | 1.67E-05 |
| Tgif2 | 374.00 | -2.92 | 0.28 | -10.27 | 1.00E-24 | 1.88E-22 |
| Casp4 | 1598.15 | -2.92 | 0.21 | -14.10 | 3.97E-45 | 2.27E-42 |
| Loxl4 | 288.66 | -2.94 | 0.47 | -6.29 | 3.12E-10 | 1.26E-08 |
| Kif11 | 159.70 | -2.95 | 0.39 | -7.66 | 1.88E-14 | 1.34E-12 |
| Gm5970 | 38.68 | -2.95 | 0.83 | -3.56 | 3.68E-04 | 4.73E-03 |
| Sec1 | 27.51 | -2.96 | 1.00 | -2.95 | 3.13E-03 | 2.85E-02 |
| Tfpi2 | 358.43 | -2.97 | 0.78 | -3.81 | 1.38E-04 | 2.01E-03 |
| Tnfaip3 | 12380.76 | -2.99 | 0.16 | -19.24 | 1.62E-82 | 3.86E-79 |
| Ripk3 | 492.93 | -3.00 | 0.35 | -8.55 | 1.25E-17 | 1.19E-15 |
| Eid3 | 48.66 | -3.01 | 0.73 | -4.12 | 3.82E-05 | 6.41E-04 |
| Gm18787 | 18.17 | -3.01 | 1.05 | -2.86 | 4.27E-03 | 3.70E-02 |
| Thbs1 | 18626.97 | -3.04 | 0.55 | -5.53 | 3.23E-08 | 1.01E-06 |
| Adora2b | 158.89 | -3.05 | 0.44 | -6.94 | 3.98E-12 | 2.10E-10 |
| Neurl3 | 7029.46 | -3.09 | 0.21 | -14.81 | 1.31E-49 | 1.25E-46 |
| Hck | 206.64 | -3.09 | 0.35 | -8.76 | 1.89E-18 | 1.96E-16 |
| Arc | 1047.98 | -3.10 | 0.32 | -9.82 | 9.12E-23 | 1.39E-20 |
| Clec4d | 157.15 | -3.10 | 0.48 | -6.47 | 9.77E-11 | 4.15E-09 |
| Slc7a5 | 602.34 | -3.10 | 0.35 | -8.79 | 1.47E-18 | 1.57E-16 |
| Gm13889 | 1212.24 | -3.11 | 0.73 | -4.27 | 1.99E-05 | 3.66E-04 |
| A730049H05Rik | 56.26 | -3.12 | 0.61 | -5.14 | 2.76E-07 | 7.35E-06 |
| Icam1 | 36584.91 | -3.12 | 0.16 | -19.48 | 1.71E-84 | 4.89E-81 |
| Calhm6 | 44.88 | -3.13 | 0.73 | -4.27 | 1.95E-05 | 3.60E-04 |
| Tnc | 259.35 | -3.13 | 0.87 | -3.60 | 3.14E-04 | 4.15E-03 |
| Stc1 | 413.28 | -3.17 | 0.33 | -9.65 | 4.82E-22 | 6.75E-20 |
| Tnfaip6 | 2527.91 | -3.18 | 0.30 | -10.48 | 1.07E-25 | 2.11E-23 |
| Chil1 | 25.28 | -3.18 | 0.95 | -3.34 | 8.44E-04 | 9.72E-03 |
| Rnd1 | 8984.52 | -3.19 | 0.21 | -15.26 | 1.51E-52 | 1.54E-49 |
| C7 | 640.69 | -3.19 | 0.34 | -9.46 | 3.07E-21 | 3.95E-19 |
| Gm614 | 75.88 | -3.19 | 0.49 | -6.45 | 1.13E-10 | 4.78E-09 |
| Kif17 | 11.55 | -3.20 | 1.21 | -2.64 | 8.38E-03 | 6.36E-02 |
| Tiparp | 6072.24 | -3.21 | 0.24 | -13.61 | 3.31E-42 | 1.63E-39 |
| Nptx2 | 16.80 | -3.21 | 1.09 | -2.96 | 3.07E-03 | 2.81E-02 |
| CR974586.4 | 12.78 | -3.25 | 1.28 | -2.54 | 1.11E-02 | 7.92E-02 |
| Cyp1b1 | 2017.54 | -3.25 | 0.28 | -11.76 | 6.25E-32 | 1.82E-29 |
| Cxcl10 | 4244.36 | -3.26 | 0.33 | -9.88 | 5.02E-23 | 7.79E-21 |
| Steap1 | 13.37 | -3.27 | 1.17 | -2.79 | 5.22E-03 | 4.34E-02 |
| Edn2 | 21.75 | -3.28 | 0.87 | -3.77 | 1.64E-04 | 2.33E-03 |
| Fpr2 | 56.22 | -3.28 | 0.67 | -4.87 | 1.14E-06 | 2.66E-05 |
| Ifi211 | 1755.34 | -3.29 | 0.22 | -14.73 | 3.94E-49 | 3.52E-46 |
| Ccl2 | 8874.72 | -3.29 | 0.23 | -14.36 | 8.72E-47 | 5.66E-44 |
| Clec4e | 412.25 | -3.30 | 0.25 | -13.02 | 9.05E-39 | 3.69E-36 |
| Arl5b | 25.38 | -3.30 | 1.22 | -2.71 | 6.75E-03 | 5.32E-02 |
| Ptpre | 964.25 | -3.32 | 0.23 | -14.40 | 5.03E-47 | 3.59E-44 |
| Ifit1 | 3645.38 | -3.32 | 0.62 | -5.32 | 1.03E-07 | 2.92E-06 |
| Gm5796 | 27.03 | -3.35 | 0.90 | -3.70 | 2.14E-04 | 2.95E-03 |
| Gm11843 | 28.39 | -3.37 | 0.78 | -4.32 | 1.55E-05 | 2.91E-04 |
| Il1a | 866.15 | -3.37 | 0.31 | -11.04 | 2.47E-28 | 5.87E-26 |
| Lipg | 110.20 | -3.39 | 0.59 | -5.75 | 9.13E-09 | 3.07E-07 |
| Ccl3 | 1737.10 | -3.39 | 0.22 | -15.52 | 2.70E-54 | 2.96E-51 |
| Ptgs2 | 4388.59 | -3.41 | 0.20 | -17.17 | 4.23E-66 | 6.71E-63 |
| Asns | 368.60 | -3.44 | 0.41 | -8.48 | 2.33E-17 | 2.17E-15 |
| Procr | 438.79 | -3.45 | 0.76 | -4.55 | 5.37E-06 | 1.12E-04 |
| Vgf | 24.46 | -3.48 | 0.96 | -3.61 | 3.04E-04 | 4.02E-03 |
| Il12b | 17.19 | -3.48 | 1.06 | -3.28 | 1.04E-03 | 1.16E-02 |
| Timp1 | 778.11 | -3.52 | 0.70 | -5.04 | 4.76E-07 | 1.21E-05 |
| Fjx1 | 129.23 | -3.52 | 0.49 | -7.25 | 4.26E-13 | 2.59E-11 |
| Mmp13 | 1100.10 | -3.54 | 0.24 | -14.66 | 1.11E-48 | 9.33E-46 |
| Cfap69 | 157.30 | -3.57 | 0.36 | -9.90 | 4.28E-23 | 6.71E-21 |
| Sgms2 | 325.63 | -3.57 | 0.37 | -9.62 | 6.59E-22 | 9.04E-20 |
| Kdm5d | 264.36 | -3.58 | 0.28 | -12.57 | 3.07E-36 | 1.04E-33 |
| Dpep3 | 9.10 | -3.60 | 1.31 | -2.75 | 6.00E-03 | 4.84E-02 |
| Egr4 | 137.08 | -3.61 | 0.42 | -8.66 | 4.54E-18 | 4.47E-16 |
| Sphk1 | 403.59 | -3.63 | 0.47 | -7.78 | 7.25E-15 | 5.48E-13 |
| Oasl1 | 1341.52 | -3.63 | 0.28 | -12.85 | 8.71E-38 | 3.19E-35 |
| Zbtb32 | 12.90 | -3.63 | 1.14 | -3.17 | 1.51E-03 | 1.59E-02 |
| Ccl4 | 1862.31 | -3.68 | 0.21 | -17.35 | 2.07E-67 | 3.69E-64 |
| Gm21748 | 46.59 | -3.69 | 0.66 | -5.57 | 2.49E-08 | 7.88E-07 |
| Gm21860 | 46.59 | -3.69 | 0.66 | -5.57 | 2.49E-08 | 7.88E-07 |
| Nod2 | 1213.74 | -3.73 | 0.20 | -18.65 | 1.20E-77 | 2.45E-74 |
| Ifi205 | 3605.88 | -3.74 | 0.26 | -14.59 | 3.17E-48 | 2.51E-45 |
| Cxcl11 | 62.45 | -3.75 | 0.84 | -4.47 | 7.65E-06 | 1.55E-04 |
| Fam83a | 55.81 | -3.76 | 0.81 | -4.62 | 3.88E-06 | 8.33E-05 |
| Ereg | 95.78 | -3.78 | 0.55 | -6.89 | 5.49E-12 | 2.85E-10 |
| Hdx | 91.52 | -3.81 | 0.54 | -7.00 | 2.60E-12 | 1.43E-10 |
| AA467197 | 21.53 | -3.82 | 1.08 | -3.55 | 3.80E-04 | 4.86E-03 |
| Olfr961 | 43.33 | -3.85 | 0.68 | -5.63 | 1.84E-08 | 5.96E-07 |
| Lman1l | 17.69 | -3.85 | 1.31 | -2.93 | 3.39E-03 | 3.05E-02 |
| Mcoln2 | 186.88 | -3.87 | 0.54 | -7.20 | 6.00E-13 | 3.60E-11 |
| Plaur | 3795.02 | -3.92 | 0.30 | -12.99 | 1.46E-38 | 5.78E-36 |
| Plet1 | 25.03 | -3.99 | 0.86 | -4.66 | 3.13E-06 | 6.79E-05 |
| Stx11 | 1550.07 | -4.00 | 0.29 | -13.69 | 1.10E-42 | 5.61E-40 |
| Il10 | 31.99 | -4.05 | 0.89 | -4.55 | 5.36E-06 | 1.12E-04 |
| Mx1 | 308.94 | -4.06 | 0.37 | -10.93 | 8.36E-28 | 1.89E-25 |
| Serpinb2 | 85.75 | -4.09 | 0.87 | -4.71 | 2.45E-06 | 5.44E-05 |
| Uty | 313.44 | -4.10 | 0.36 | -11.47 | 1.79E-30 | 4.83E-28 |
| Serpina3f | 232.69 | -4.12 | 0.50 | -8.16 | 3.26E-16 | 2.84E-14 |
| Asprv1 | 24.50 | -4.12 | 1.07 | -3.86 | 1.13E-04 | 1.68E-03 |
| Il6 | 4413.93 | -4.20 | 0.63 | -6.70 | 2.10E-11 | 9.86E-10 |
| Cxcl5 | 1362.54 | -4.21 | 0.77 | -5.45 | 5.18E-08 | 1.56E-06 |
| Ccl5 | 417.78 | -4.21 | 0.33 | -12.58 | 2.63E-36 | 9.15E-34 |
| Il1rn | 773.10 | -4.29 | 0.33 | -12.95 | 2.40E-38 | 9.02E-36 |
| Rsph4a | 11.15 | -4.32 | 1.47 | -2.94 | 3.32E-03 | 2.99E-02 |
| Sele | 8807.35 | -4.33 | 0.20 | -22.12 | 2.24E-108 | 1.60E-104 |
| Gm47283 | 642.41 | -4.34 | 0.33 | -12.96 | 2.19E-38 | 8.45E-36 |
| Fst | 18.24 | -4.35 | 1.20 | -3.63 | 2.81E-04 | 3.76E-03 |
| Psors1c2 | 15.28 | -4.36 | 1.35 | -3.23 | 1.23E-03 | 1.34E-02 |
| Cyp1a1 | 3529.29 | -4.40 | 0.68 | -6.50 | 7.91E-11 | 3.44E-09 |
| Acod1 | 594.00 | -4.42 | 0.33 | -13.42 | 4.83E-41 | 2.22E-38 |
| Gm8488 | 7.47 | -4.57 | 1.81 | -2.53 | 1.14E-02 | 8.12E-02 |
| Trim30c | 98.64 | -4.60 | 0.60 | -7.63 | 2.28E-14 | 1.62E-12 |
| Prss46 | 13.83 | -4.67 | 1.30 | -3.59 | 3.32E-04 | 4.34E-03 |
| Gm18445 | 14.52 | -4.71 | 1.57 | -3.00 | 2.67E-03 | 2.52E-02 |
| Sh2d5 | 91.67 | -4.75 | 0.67 | -7.07 | 1.58E-12 | 9.01E-11 |
| Olr1 | 403.34 | -4.77 | 0.38 | -12.67 | 8.59E-37 | 3.07E-34 |
| Sprr1a | 36.95 | -4.79 | 0.94 | -5.10 | 3.33E-07 | 8.73E-06 |
| B3gnt7 | 10.88 | -4.80 | 1.70 | -2.82 | 4.77E-03 | 4.04E-02 |
| Lif | 806.47 | -4.81 | 0.29 | -16.76 | 4.52E-63 | 5.86E-60 |
| Selp | 5916.24 | -4.95 | 0.24 | -20.71 | 2.67E-95 | 1.27E-91 |
| Hba-a1 | 94.82 | -4.97 | 1.91 | -2.60 | 9.23E-03 | 6.88E-02 |
| Adamts4 | 10018.85 | -4.98 | 0.62 | -8.00 | 1.23E-15 | 1.00E-13 |
| Samd11 | 29.60 | -5.00 | 0.96 | -5.21 | 1.86E-07 | 5.08E-06 |
| S100a8 | 7.66 | -5.12 | 2.00 | -2.56 | 1.05E-02 | 7.56E-02 |
| Ifnb1 | 61.05 | -5.12 | 0.76 | -6.76 | 1.34E-11 | 6.48E-10 |
| Fgf23 | 179.65 | -5.20 | 0.59 | -8.79 | 1.53E-18 | 1.62E-16 |
| Sprr2b | 6.71 | -5.21 | 2.07 | -2.52 | 1.17E-02 | 8.24E-02 |
| Ptx3 | 11707.76 | -5.24 | 0.60 | -8.79 | 1.55E-18 | 1.63E-16 |
| Ifitm7 | 5.73 | -5.31 | 2.09 | -2.54 | 1.10E-02 | 7.87E-02 |
| Slc6a14 | 11.26 | -5.32 | 1.71 | -3.11 | 1.86E-03 | 1.89E-02 |
| Foxj1 | 60.78 | -5.36 | 0.83 | -6.44 | 1.22E-10 | 5.12E-09 |
| Cox5b-ps | 5.79 | -5.38 | 2.04 | -2.64 | 8.38E-03 | 6.36E-02 |
| Cxcl3 | 221.80 | -5.43 | 1.18 | -4.61 | 4.05E-06 | 8.65E-05 |
| Gm3752 | 7.00 | -5.44 | 1.97 | -2.75 | 5.89E-03 | 4.76E-02 |
| Csf2 | 160.66 | -5.45 | 0.63 | -8.70 | 3.41E-18 | 3.40E-16 |
| Csf3 | 579.06 | -5.48 | 0.50 | -10.98 | 4.65E-28 | 1.07E-25 |
| Eif2s3y | 976.20 | -5.66 | 0.28 | -20.49 | 2.85E-93 | 1.02E-89 |
| Slfn1 | 35.56 | -5.76 | 1.20 | -4.80 | 1.55E-06 | 3.55E-05 |
| Ddx3y | 2056.56 | -5.82 | 0.24 | -24.51 | 1.19E-132 | 1.70E-128 |
| Gm3667 | 12.52 | -5.84 | 1.72 | -3.39 | 7.10E-04 | 8.37E-03 |
| G530012D18Rik | 5.94 | -5.90 | 1.97 | -3.00 | 2.74E-03 | 2.56E-02 |
| Colca2 | 11.21 | -6.14 | 1.80 | -3.41 | 6.43E-04 | 7.66E-03 |
| Hmgb1-ps2 | 7.39 | -6.23 | 2.01 | -3.10 | 1.94E-03 | 1.96E-02 |
| Il13ra2 | 7.33 | -6.27 | 1.90 | -3.31 | 9.32E-04 | 1.06E-02 |
| Pphln1 | 7.26 | -6.51 | 2.17 | -3.00 | 2.68E-03 | 2.52E-02 |
| Nipal1 | 10.43 | -6.56 | 1.81 | -3.62 | 2.91E-04 | 3.88E-03 |
| Gm15710 | 12.36 | -6.62 | 1.75 | -3.78 | 1.57E-04 | 2.24E-03 |
| C5ar2 | 24.91 | -7.76 | 1.59 | -4.89 | 9.90E-07 | 2.35E-05 |

RV8h vs RV0h DEGs

|  | baseMean | log2FoldChange | lfcSE | stat | pvalue | padj |
| --- | --- | --- | --- | --- | --- | --- |
| Gm28439 | 1394.53 | 27.43 | 4.78 | 5.73 | 9.85E-09 | 2.61E-07 |
| Alpk2 | 40.88 | 7.82 | 1.60 | 4.89 | 1.02E-06 | 2.01E-05 |
| Gpr22 | 17.11 | 7.80 | 3.01 | 2.60 | 9.42E-03 | 6.16E-02 |
| Gm49356 | 6.76 | 5.81 | 1.89 | 3.07 | 2.17E-03 | 1.87E-02 |
| BC049715 | 11.82 | 4.66 | 1.32 | 3.54 | 3.97E-04 | 4.37E-03 |
| Gm5141 | 9.73 | 3.31 | 1.32 | 2.50 | 1.23E-02 | 7.51E-02 |
| Dbp | 1319.85 | 2.96 | 0.25 | 11.79 | 4.57E-32 | 7.89E-30 |
| Per3 | 625.45 | 2.89 | 0.28 | 10.41 | 2.30E-25 | 2.37E-23 |
| AI854703 | 21.74 | 2.77 | 0.83 | 3.35 | 8.16E-04 | 8.04E-03 |
| Gpr34 | 17.85 | 2.73 | 0.88 | 3.12 | 1.79E-03 | 1.57E-02 |
| Egfl7 | 416.45 | 2.50 | 0.27 | 9.31 | 1.33E-20 | 9.83E-19 |
| Gm29609 | 35.06 | 2.50 | 0.87 | 2.88 | 3.98E-03 | 3.10E-02 |
| Snurf | 21.70 | 2.46 | 0.98 | 2.50 | 1.24E-02 | 7.56E-02 |
| Zfp985 | 18.96 | 2.29 | 0.96 | 2.38 | 1.74E-02 | 9.74E-02 |
| Sertad4 | 78.97 | 2.27 | 0.45 | 5.03 | 4.99E-07 | 1.04E-05 |
| Kctd12b | 129.44 | 2.21 | 0.36 | 6.18 | 6.60E-10 | 1.99E-08 |
| Myct1 | 216.53 | 2.18 | 0.33 | 6.59 | 4.28E-11 | 1.47E-09 |
| Sox4 | 1365.40 | 2.14 | 0.20 | 10.71 | 8.90E-27 | 1.07E-24 |
| B3gnt8 | 32.82 | 2.08 | 0.61 | 3.39 | 7.00E-04 | 7.06E-03 |
| Sv2a | 78.29 | 2.04 | 0.44 | 4.67 | 2.97E-06 | 5.44E-05 |
| Gng4 | 27.45 | 2.04 | 0.77 | 2.65 | 7.95E-03 | 5.40E-02 |
| Nynrin | 96.99 | 2.04 | 0.40 | 5.06 | 4.10E-07 | 8.77E-06 |
| Flt3l | 123.98 | 2.03 | 0.40 | 5.13 | 2.97E-07 | 6.49E-06 |
| Gstt2 | 150.03 | 1.99 | 0.41 | 4.82 | 1.45E-06 | 2.81E-05 |
| Aplnr | 92.56 | 1.98 | 0.57 | 3.47 | 5.25E-04 | 5.53E-03 |
| Clec14a | 185.76 | 1.97 | 0.34 | 5.74 | 9.50E-09 | 2.53E-07 |
| Mief1 | 33.96 | 1.97 | 0.77 | 2.56 | 1.04E-02 | 6.64E-02 |
| Calhm2 | 51.52 | 1.96 | 0.50 | 3.90 | 9.66E-05 | 1.25E-03 |
| Zfp760 | 33.41 | 1.88 | 0.62 | 3.02 | 2.57E-03 | 2.15E-02 |
| Chst12 | 47.20 | 1.85 | 0.57 | 3.24 | 1.20E-03 | 1.13E-02 |
| Heyl | 481.32 | 1.85 | 0.25 | 7.44 | 9.98E-14 | 4.27E-12 |
| Mmp28 | 90.09 | 1.82 | 0.42 | 4.31 | 1.60E-05 | 2.53E-04 |
| Gstt3 | 56.76 | 1.81 | 0.51 | 3.53 | 4.15E-04 | 4.54E-03 |
| Izumo4 | 53.96 | 1.80 | 0.57 | 3.19 | 1.42E-03 | 1.30E-02 |
| Zfp607b | 24.94 | 1.77 | 0.67 | 2.63 | 8.52E-03 | 5.70E-02 |
| Rtl5 | 44.93 | 1.77 | 0.70 | 2.52 | 1.18E-02 | 7.30E-02 |
| Camsap3 | 24.24 | 1.77 | 0.74 | 2.40 | 1.64E-02 | 9.30E-02 |
| Calcoco1 | 379.28 | 1.76 | 0.35 | 5.05 | 4.49E-07 | 9.50E-06 |
| Slc38a1 | 141.26 | 1.76 | 0.43 | 4.05 | 5.02E-05 | 7.00E-04 |
| Inka1 | 42.98 | 1.73 | 0.59 | 2.92 | 3.47E-03 | 2.77E-02 |
| Aph1c | 40.06 | 1.73 | 0.72 | 2.40 | 1.64E-02 | 9.32E-02 |
| Smad9 | 41.57 | 1.72 | 0.62 | 2.79 | 5.28E-03 | 3.92E-02 |
| Sall2 | 27.83 | 1.71 | 0.64 | 2.66 | 7.71E-03 | 5.28E-02 |
| Snai3 | 51.01 | 1.69 | 0.67 | 2.50 | 1.24E-02 | 7.58E-02 |
| Wscd1 | 79.87 | 1.66 | 0.50 | 3.36 | 7.89E-04 | 7.82E-03 |
| Meox2 | 369.27 | 1.66 | 0.30 | 5.57 | 2.56E-08 | 6.36E-07 |
| Fibin | 179.33 | 1.66 | 0.39 | 4.29 | 1.76E-05 | 2.75E-04 |
| D630003M21Rik | 144.02 | 1.66 | 0.34 | 4.91 | 9.20E-07 | 1.82E-05 |
| Phf21b | 74.35 | 1.66 | 0.45 | 3.66 | 2.56E-04 | 2.94E-03 |
| Neurl1b | 91.92 | 1.65 | 0.41 | 4.00 | 6.39E-05 | 8.64E-04 |
| Ddit4l | 48.88 | 1.64 | 0.50 | 3.27 | 1.08E-03 | 1.03E-02 |
| Gm21981 | 27.06 | 1.64 | 0.67 | 2.46 | 1.39E-02 | 8.26E-02 |
| Kcna2 | 96.89 | 1.63 | 0.43 | 3.76 | 1.68E-04 | 2.03E-03 |
| Dna2 | 35.42 | 1.61 | 0.65 | 2.47 | 1.35E-02 | 8.06E-02 |
| Cbx2 | 52.42 | 1.60 | 0.50 | 3.21 | 1.34E-03 | 1.23E-02 |
| Rufy2 | 159.99 | 1.59 | 0.43 | 3.67 | 2.43E-04 | 2.80E-03 |
| Zfp882 | 33.82 | 1.58 | 0.61 | 2.60 | 9.35E-03 | 6.13E-02 |
| Hspa12b | 547.51 | 1.58 | 0.22 | 7.18 | 6.91E-13 | 2.75E-11 |
| Thsd1 | 100.77 | 1.57 | 0.43 | 3.68 | 2.36E-04 | 2.74E-03 |
| Aatk | 144.01 | 1.57 | 0.37 | 4.28 | 1.83E-05 | 2.85E-04 |
| Zfp60 | 178.70 | 1.57 | 0.38 | 4.18 | 2.98E-05 | 4.33E-04 |
| Ccdc8 | 83.12 | 1.55 | 0.48 | 3.21 | 1.32E-03 | 1.21E-02 |
| Palm | 680.53 | 1.55 | 0.30 | 5.19 | 2.07E-07 | 4.61E-06 |
| St6galnac2 | 163.67 | 1.54 | 0.36 | 4.27 | 1.95E-05 | 3.00E-04 |
| Mansc1 | 37.72 | 1.52 | 0.58 | 2.62 | 8.92E-03 | 5.92E-02 |
| Zfp963 | 52.21 | 1.51 | 0.49 | 3.05 | 2.29E-03 | 1.96E-02 |
| Tef | 1689.28 | 1.51 | 0.22 | 6.91 | 4.94E-12 | 1.84E-10 |
| Tnfrsf25 | 57.80 | 1.50 | 0.54 | 2.78 | 5.38E-03 | 3.97E-02 |
| Gm42688 | 51.03 | 1.49 | 0.54 | 2.76 | 5.81E-03 | 4.22E-02 |
| Zfp169 | 45.06 | 1.48 | 0.57 | 2.60 | 9.31E-03 | 6.12E-02 |
| Zfp72 | 43.73 | 1.48 | 0.58 | 2.53 | 1.15E-02 | 7.13E-02 |
| Klhl33 | 1094.91 | 1.47 | 0.30 | 4.94 | 7.66E-07 | 1.54E-05 |
| Pirt | 176.00 | 1.45 | 0.35 | 4.19 | 2.78E-05 | 4.09E-04 |
| Gask1b | 516.92 | 1.45 | 0.27 | 5.32 | 1.02E-07 | 2.33E-06 |
| Hey1 | 143.92 | 1.45 | 0.47 | 3.10 | 1.90E-03 | 1.66E-02 |
| Cyren | 68.17 | 1.42 | 0.50 | 2.87 | 4.07E-03 | 3.16E-02 |
| Fut10 | 48.95 | 1.42 | 0.53 | 2.69 | 7.15E-03 | 4.98E-02 |
| Per2 | 673.99 | 1.42 | 0.30 | 4.71 | 2.42E-06 | 4.53E-05 |
| Hlf | 635.89 | 1.41 | 0.26 | 5.47 | 4.60E-08 | 1.10E-06 |
| Slc25a45 | 46.50 | 1.41 | 0.51 | 2.78 | 5.37E-03 | 3.97E-02 |
| Zfp354c | 47.00 | 1.41 | 0.52 | 2.70 | 7.01E-03 | 4.90E-02 |
| Snx33 | 169.38 | 1.40 | 0.32 | 4.40 | 1.11E-05 | 1.81E-04 |
| Zbtb8a | 48.91 | 1.40 | 0.55 | 2.57 | 1.02E-02 | 6.56E-02 |
| Lca5 | 63.91 | 1.39 | 0.58 | 2.40 | 1.65E-02 | 9.35E-02 |
| Ciart | 154.80 | 1.39 | 0.45 | 3.11 | 1.89E-03 | 1.65E-02 |
| Tor4a | 63.13 | 1.39 | 0.47 | 2.98 | 2.87E-03 | 2.37E-02 |
| Dynlt1c | 116.73 | 1.38 | 0.35 | 4.00 | 6.47E-05 | 8.71E-04 |
| Rasl12 | 155.30 | 1.36 | 0.33 | 4.18 | 2.91E-05 | 4.25E-04 |
| Iffo1 | 217.87 | 1.35 | 0.28 | 4.90 | 9.48E-07 | 1.87E-05 |
| Clcn1 | 252.00 | 1.35 | 0.30 | 4.44 | 9.02E-06 | 1.49E-04 |
| Zfp526 | 126.38 | 1.34 | 0.39 | 3.45 | 5.70E-04 | 5.94E-03 |
| Zkscan8 | 159.91 | 1.34 | 0.44 | 3.07 | 2.15E-03 | 1.85E-02 |
| Xndc1 | 183.34 | 1.33 | 0.34 | 3.94 | 8.08E-05 | 1.07E-03 |
| Shprh | 296.27 | 1.33 | 0.32 | 4.13 | 3.59E-05 | 5.13E-04 |
| Cdc25b | 73.83 | 1.33 | 0.45 | 2.97 | 2.99E-03 | 2.45E-02 |
| Golga1 | 358.92 | 1.32 | 0.30 | 4.45 | 8.65E-06 | 1.44E-04 |
| Mmgt2 | 80.76 | 1.31 | 0.51 | 2.55 | 1.09E-02 | 6.87E-02 |
| 9430015G10Rik | 92.62 | 1.31 | 0.37 | 3.51 | 4.40E-04 | 4.77E-03 |
| Spaar | 62.12 | 1.30 | 0.50 | 2.57 | 1.02E-02 | 6.55E-02 |
| Iqce | 114.84 | 1.29 | 0.38 | 3.40 | 6.80E-04 | 6.87E-03 |
| Rgp1 | 1057.23 | 1.29 | 0.21 | 6.11 | 1.03E-09 | 3.03E-08 |
| Stard9 | 121.04 | 1.29 | 0.40 | 3.24 | 1.21E-03 | 1.13E-02 |
| Dixdc1 | 270.05 | 1.29 | 0.40 | 3.26 | 1.12E-03 | 1.05E-02 |
| Adamts7 | 884.08 | 1.28 | 0.26 | 4.96 | 7.11E-07 | 1.44E-05 |
| Ahnak2 | 360.09 | 1.28 | 0.29 | 4.48 | 7.32E-06 | 1.24E-04 |
| Pik3ip1 | 1091.23 | 1.27 | 0.20 | 6.40 | 1.57E-10 | 5.06E-09 |
| Plscr4 | 246.39 | 1.27 | 0.27 | 4.69 | 2.72E-06 | 5.03E-05 |
| Tril | 88.30 | 1.27 | 0.43 | 2.94 | 3.30E-03 | 2.66E-02 |
| Sertad3 | 48.88 | 1.26 | 0.51 | 2.47 | 1.33E-02 | 7.99E-02 |
| Stk38 | 74.32 | 1.25 | 0.42 | 3.01 | 2.59E-03 | 2.16E-02 |
| Lims2 | 1011.32 | 1.25 | 0.27 | 4.66 | 3.11E-06 | 5.66E-05 |
| Sema6c | 204.53 | 1.24 | 0.33 | 3.82 | 1.36E-04 | 1.68E-03 |
| Amt | 89.93 | 1.24 | 0.43 | 2.92 | 3.47E-03 | 2.77E-02 |
| Slc27a1 | 6616.45 | 1.24 | 0.17 | 7.49 | 7.11E-14 | 3.09E-12 |
| Galnt17 | 235.16 | 1.24 | 0.27 | 4.63 | 3.70E-06 | 6.61E-05 |
| Zfp518b | 148.50 | 1.23 | 0.40 | 3.05 | 2.29E-03 | 1.96E-02 |
| Ddc | 182.75 | 1.23 | 0.29 | 4.22 | 2.42E-05 | 3.62E-04 |
| Il2ra | 64.18 | 1.23 | 0.45 | 2.72 | 6.58E-03 | 4.66E-02 |
| 1700030K09Rik | 108.01 | 1.22 | 0.38 | 3.18 | 1.47E-03 | 1.33E-02 |
| Hic1 | 196.60 | 1.22 | 0.40 | 3.04 | 2.40E-03 | 2.04E-02 |
| Cacng6 | 75.04 | 1.22 | 0.51 | 2.39 | 1.67E-02 | 9.43E-02 |
| Asb14 | 320.26 | 1.21 | 0.27 | 4.57 | 4.86E-06 | 8.50E-05 |
| Gimap1 | 74.68 | 1.20 | 0.42 | 2.86 | 4.26E-03 | 3.28E-02 |
| Card6 | 125.13 | 1.19 | 0.41 | 2.88 | 3.92E-03 | 3.06E-02 |
| Plag1 | 64.46 | 1.19 | 0.45 | 2.68 | 7.42E-03 | 5.13E-02 |
| Cep295 | 217.73 | 1.19 | 0.32 | 3.71 | 2.07E-04 | 2.43E-03 |
| Nr2f1 | 90.86 | 1.18 | 0.41 | 2.90 | 3.79E-03 | 2.98E-02 |
| Rasd2 | 70.72 | 1.18 | 0.48 | 2.48 | 1.30E-02 | 7.84E-02 |
| Capn10 | 105.12 | 1.18 | 0.40 | 2.94 | 3.28E-03 | 2.65E-02 |
| Cth | 75.78 | 1.18 | 0.42 | 2.82 | 4.88E-03 | 3.67E-02 |
| Inpp5b | 376.19 | 1.18 | 0.26 | 4.46 | 8.37E-06 | 1.40E-04 |
| Ccdc167 | 94.42 | 1.18 | 0.39 | 3.02 | 2.49E-03 | 2.09E-02 |
| Alox12 | 71.81 | 1.17 | 0.48 | 2.45 | 1.44E-02 | 8.45E-02 |
| Tmem164 | 901.65 | 1.17 | 0.24 | 4.94 | 7.90E-07 | 1.58E-05 |
| Ccdc71 | 322.43 | 1.16 | 0.27 | 4.29 | 1.79E-05 | 2.79E-04 |
| Acad10 | 209.04 | 1.16 | 0.31 | 3.73 | 1.92E-04 | 2.27E-03 |
| Nr2f2 | 236.86 | 1.16 | 0.35 | 3.34 | 8.46E-04 | 8.29E-03 |
| 2210407C18Rik | 207.61 | 1.16 | 0.35 | 3.30 | 9.56E-04 | 9.25E-03 |
| Pi16 | 442.63 | 1.16 | 0.30 | 3.84 | 1.25E-04 | 1.56E-03 |
| Zfp113 | 94.92 | 1.15 | 0.38 | 3.02 | 2.49E-03 | 2.09E-02 |
| Zfp866 | 288.38 | 1.15 | 0.28 | 4.11 | 4.03E-05 | 5.71E-04 |
| Pik3r2 | 457.45 | 1.15 | 0.24 | 4.80 | 1.61E-06 | 3.09E-05 |
| Snrk | 2174.32 | 1.14 | 0.17 | 6.61 | 3.81E-11 | 1.31E-09 |
| Abca8a | 2282.23 | 1.13 | 0.22 | 5.18 | 2.22E-07 | 4.93E-06 |
| Fubp3 | 299.47 | 1.13 | 0.28 | 4.00 | 6.40E-05 | 8.64E-04 |
| Taok2 | 1157.34 | 1.13 | 0.19 | 6.06 | 1.35E-09 | 3.94E-08 |
| Tmem220 | 64.75 | 1.13 | 0.46 | 2.43 | 1.51E-02 | 8.78E-02 |
| Kcnj8 | 706.36 | 1.13 | 0.20 | 5.73 | 1.02E-08 | 2.69E-07 |
| Tmem204 | 464.69 | 1.12 | 0.24 | 4.72 | 2.38E-06 | 4.46E-05 |
| Proser3 | 74.04 | 1.12 | 0.42 | 2.64 | 8.27E-03 | 5.58E-02 |
| Gsta3 | 142.16 | 1.12 | 0.36 | 3.07 | 2.12E-03 | 1.83E-02 |
| Pnmal2 | 104.33 | 1.11 | 0.40 | 2.78 | 5.46E-03 | 4.02E-02 |
| Kdm6a | 615.64 | 1.11 | 0.26 | 4.22 | 2.46E-05 | 3.66E-04 |
| Zfp606 | 139.93 | 1.11 | 0.34 | 3.26 | 1.12E-03 | 1.06E-02 |
| Ky | 295.59 | 1.11 | 0.33 | 3.38 | 7.16E-04 | 7.19E-03 |
| Gm48348 | 91.28 | 1.10 | 0.40 | 2.79 | 5.26E-03 | 3.91E-02 |
| Tnfrsf19 | 111.78 | 1.10 | 0.43 | 2.57 | 1.01E-02 | 6.48E-02 |
| Hpgd | 105.10 | 1.10 | 0.43 | 2.57 | 1.02E-02 | 6.56E-02 |
| Pias3 | 229.38 | 1.10 | 0.27 | 4.03 | 5.59E-05 | 7.67E-04 |
| Nrxn2 | 79.69 | 1.10 | 0.44 | 2.52 | 1.19E-02 | 7.31E-02 |
| Dffb | 141.40 | 1.09 | 0.33 | 3.27 | 1.07E-03 | 1.02E-02 |
| Cbx7 | 538.52 | 1.09 | 0.24 | 4.51 | 6.37E-06 | 1.09E-04 |
| Frmd8 | 207.45 | 1.08 | 0.32 | 3.40 | 6.70E-04 | 6.80E-03 |
| Fzd2 | 73.42 | 1.08 | 0.43 | 2.55 | 1.08E-02 | 6.84E-02 |
| Rgma | 784.37 | 1.08 | 0.21 | 5.20 | 1.96E-07 | 4.40E-06 |
| Zfp395 | 278.02 | 1.08 | 0.33 | 3.29 | 1.00E-03 | 9.62E-03 |
| Gm14416 | 78.11 | 1.08 | 0.44 | 2.44 | 1.48E-02 | 8.64E-02 |
| Adprm | 145.91 | 1.07 | 0.32 | 3.38 | 7.37E-04 | 7.37E-03 |
| Lysmd1 | 95.00 | 1.07 | 0.37 | 2.87 | 4.10E-03 | 3.17E-02 |
| Ctns | 192.14 | 1.07 | 0.30 | 3.51 | 4.43E-04 | 4.80E-03 |
| Aasdh | 366.85 | 1.07 | 0.32 | 3.34 | 8.44E-04 | 8.28E-03 |
| Abca12 | 161.49 | 1.06 | 0.38 | 2.78 | 5.50E-03 | 4.03E-02 |
| Ston1 | 157.02 | 1.06 | 0.34 | 3.09 | 2.00E-03 | 1.74E-02 |
| Pfkfb1 | 502.88 | 1.06 | 0.31 | 3.42 | 6.24E-04 | 6.40E-03 |
| Dok4 | 214.91 | 1.06 | 0.30 | 3.54 | 4.00E-04 | 4.40E-03 |
| Pcdh18 | 102.78 | 1.06 | 0.40 | 2.61 | 9.05E-03 | 5.98E-02 |
| Thap3 | 168.09 | 1.05 | 0.30 | 3.47 | 5.27E-04 | 5.55E-03 |
| Atxn7l2 | 122.22 | 1.05 | 0.36 | 2.92 | 3.46E-03 | 2.77E-02 |
| Tmc8 | 96.87 | 1.05 | 0.42 | 2.48 | 1.30E-02 | 7.83E-02 |
| Ttf1 | 255.08 | 1.05 | 0.29 | 3.59 | 3.37E-04 | 3.78E-03 |
| Ap5z1 | 230.78 | 1.05 | 0.30 | 3.50 | 4.61E-04 | 4.95E-03 |
| Cavin2 | 3134.14 | 1.05 | 0.20 | 5.12 | 3.12E-07 | 6.79E-06 |
| Sybu | 127.69 | 1.04 | 0.35 | 3.01 | 2.62E-03 | 2.18E-02 |
| Zfp862-ps | 108.62 | 1.04 | 0.36 | 2.89 | 3.86E-03 | 3.02E-02 |
| Inka2 | 511.35 | 1.04 | 0.25 | 4.15 | 3.33E-05 | 4.79E-04 |
| Pitpnm2 | 2003.78 | 1.04 | 0.21 | 4.92 | 8.80E-07 | 1.75E-05 |
| Bcl9 | 801.51 | 1.04 | 0.29 | 3.61 | 3.02E-04 | 3.44E-03 |
| AC149090.1 | 654.02 | 1.04 | 0.21 | 4.97 | 6.80E-07 | 1.39E-05 |
| Pm20d1 | 387.84 | 1.03 | 0.25 | 4.09 | 4.30E-05 | 6.06E-04 |
| Zfp280c | 159.39 | 1.02 | 0.40 | 2.56 | 1.06E-02 | 6.73E-02 |
| Gm10359 | 4934.54 | 1.02 | 0.34 | 3.02 | 2.57E-03 | 2.15E-02 |
| Gm12671 | 4934.54 | 1.02 | 0.34 | 3.02 | 2.57E-03 | 2.15E-02 |
| Cbfa2t3 | 555.57 | 1.02 | 0.21 | 4.93 | 8.43E-07 | 1.68E-05 |
| Setd1b | 612.57 | 1.02 | 0.26 | 3.98 | 6.99E-05 | 9.32E-04 |
| Tgfbr3l | 77.24 | 1.02 | 0.41 | 2.48 | 1.31E-02 | 7.87E-02 |
| Speg | 8569.42 | 1.02 | 0.16 | 6.36 | 1.99E-10 | 6.40E-09 |
| Dcaf17 | 109.97 | 1.02 | 0.37 | 2.71 | 6.73E-03 | 4.74E-02 |
| Clasrp | 601.37 | 1.01 | 0.23 | 4.34 | 1.39E-05 | 2.23E-04 |
| Ide | 2918.67 | 1.01 | 0.20 | 5.11 | 3.24E-07 | 7.03E-06 |
| Tctn1 | 188.66 | 1.01 | 0.37 | 2.75 | 6.01E-03 | 4.34E-02 |
| Depp1 | 634.37 | 1.00 | 0.30 | 3.40 | 6.83E-04 | 6.91E-03 |
| Shld2 | 117.18 | 1.00 | 0.38 | 2.66 | 7.71E-03 | 5.28E-02 |
| Gigyf1 | 1118.82 | 1.00 | 0.23 | 4.45 | 8.79E-06 | 1.46E-04 |
| Tbc1d13 | 367.51 | 1.00 | 0.28 | 3.57 | 3.58E-04 | 3.99E-03 |
| Pcmtd2 | 1374.52 | 0.99 | 0.26 | 3.76 | 1.70E-04 | 2.04E-03 |
| Trib2 | 499.65 | 0.99 | 0.22 | 4.49 | 7.28E-06 | 1.23E-04 |
| Fam13c | 166.87 | 0.99 | 0.32 | 3.12 | 1.79E-03 | 1.58E-02 |
| Ccdc102a | 101.47 | 0.99 | 0.36 | 2.79 | 5.35E-03 | 3.96E-02 |
| Brat1 | 182.42 | 0.99 | 0.36 | 2.77 | 5.54E-03 | 4.06E-02 |
| Usp54 | 867.02 | 0.99 | 0.26 | 3.83 | 1.26E-04 | 1.57E-03 |
| Plxnb1 | 1741.57 | 0.99 | 0.25 | 4.03 | 5.54E-05 | 7.62E-04 |
| Caskin2 | 1440.27 | 0.99 | 0.18 | 5.48 | 4.29E-08 | 1.03E-06 |
| Slc25a29 | 164.27 | 0.99 | 0.31 | 3.15 | 1.61E-03 | 1.43E-02 |
| Kank3 | 792.73 | 0.99 | 0.27 | 3.68 | 2.31E-04 | 2.69E-03 |
| Rp1 | 196.14 | 0.99 | 0.36 | 2.77 | 5.59E-03 | 4.09E-02 |
| Cep250 | 373.98 | 0.99 | 0.26 | 3.78 | 1.55E-04 | 1.88E-03 |
| Hdac7 | 1202.98 | 0.98 | 0.29 | 3.38 | 7.34E-04 | 7.35E-03 |
| Lysmd4 | 251.96 | 0.98 | 0.32 | 3.07 | 2.11E-03 | 1.82E-02 |
| Casp9 | 217.42 | 0.98 | 0.31 | 3.11 | 1.84E-03 | 1.61E-02 |
| Adamts6 | 113.00 | 0.98 | 0.38 | 2.57 | 1.02E-02 | 6.56E-02 |
| Rreb1 | 1247.95 | 0.98 | 0.23 | 4.18 | 2.87E-05 | 4.19E-04 |
| Afdn | 1737.88 | 0.98 | 0.20 | 4.78 | 1.78E-06 | 3.40E-05 |
| Adal | 179.71 | 0.97 | 0.30 | 3.26 | 1.12E-03 | 1.05E-02 |
| Igsf9b | 177.28 | 0.97 | 0.31 | 3.19 | 1.43E-03 | 1.30E-02 |
| Ccdc141 | 3529.06 | 0.97 | 0.20 | 4.78 | 1.72E-06 | 3.28E-05 |
| Sbk1 | 2382.28 | 0.97 | 0.23 | 4.30 | 1.68E-05 | 2.63E-04 |
| Gpcpd1 | 7457.26 | 0.97 | 0.26 | 3.72 | 2.01E-04 | 2.37E-03 |
| Cers4 | 1284.24 | 0.97 | 0.19 | 5.10 | 3.39E-07 | 7.31E-06 |
| Hmgcs2 | 700.09 | 0.96 | 0.26 | 3.74 | 1.86E-04 | 2.21E-03 |
| Zfp523 | 142.09 | 0.96 | 0.39 | 2.46 | 1.38E-02 | 8.19E-02 |
| Zfp784 | 93.62 | 0.96 | 0.40 | 2.42 | 1.56E-02 | 8.98E-02 |
| Zfp467 | 97.62 | 0.95 | 0.37 | 2.60 | 9.42E-03 | 6.16E-02 |
| Esrrb | 691.79 | 0.95 | 0.25 | 3.79 | 1.48E-04 | 1.82E-03 |
| Art5 | 549.85 | 0.95 | 0.22 | 4.25 | 2.16E-05 | 3.27E-04 |
| Cpt1c | 87.93 | 0.95 | 0.40 | 2.37 | 1.76E-02 | 9.83E-02 |
| Card10 | 612.38 | 0.95 | 0.24 | 3.99 | 6.53E-05 | 8.79E-04 |
| 2610008E11Rik | 396.81 | 0.94 | 0.26 | 3.60 | 3.18E-04 | 3.59E-03 |
| Yeats2 | 110.73 | 0.94 | 0.40 | 2.38 | 1.73E-02 | 9.74E-02 |
| Adcy6 | 6971.13 | 0.94 | 0.20 | 4.69 | 2.70E-06 | 5.01E-05 |
| Dclre1a | 179.68 | 0.94 | 0.36 | 2.58 | 9.81E-03 | 6.36E-02 |
| Aqp7 | 233.89 | 0.94 | 0.28 | 3.40 | 6.76E-04 | 6.84E-03 |
| Cracr2b | 254.28 | 0.94 | 0.26 | 3.57 | 3.59E-04 | 4.00E-03 |
| Fam219b | 255.81 | 0.94 | 0.30 | 3.16 | 1.56E-03 | 1.40E-02 |
| Top3a | 133.38 | 0.94 | 0.35 | 2.65 | 8.12E-03 | 5.49E-02 |
| Fbxo9 | 525.96 | 0.94 | 0.23 | 3.98 | 6.78E-05 | 9.05E-04 |
| Uvssa | 227.83 | 0.93 | 0.33 | 2.84 | 4.48E-03 | 3.42E-02 |
| Rnf207 | 2221.29 | 0.93 | 0.18 | 5.30 | 1.14E-07 | 2.60E-06 |
| Tmem104 | 261.01 | 0.93 | 0.26 | 3.61 | 3.04E-04 | 3.46E-03 |
| Scrn1 | 298.54 | 0.93 | 0.25 | 3.75 | 1.74E-04 | 2.09E-03 |
| Patz1 | 339.56 | 0.93 | 0.29 | 3.18 | 1.50E-03 | 1.35E-02 |
| Cnnm3 | 430.14 | 0.93 | 0.26 | 3.53 | 4.19E-04 | 4.58E-03 |
| Yipf6 | 400.45 | 0.93 | 0.26 | 3.58 | 3.41E-04 | 3.82E-03 |
| Jag2 | 1230.64 | 0.93 | 0.17 | 5.34 | 9.18E-08 | 2.11E-06 |
| Plcg1 | 1660.54 | 0.93 | 0.20 | 4.74 | 2.13E-06 | 4.00E-05 |
| Nbeal2 | 467.76 | 0.93 | 0.24 | 3.87 | 1.07E-04 | 1.35E-03 |
| Hey2 | 164.45 | 0.92 | 0.33 | 2.82 | 4.88E-03 | 3.67E-02 |
| Mtus2 | 4168.59 | 0.92 | 0.25 | 3.76 | 1.71E-04 | 2.06E-03 |
| Zfp949 | 151.97 | 0.92 | 0.39 | 2.38 | 1.74E-02 | 9.78E-02 |
| Car11 | 91.65 | 0.92 | 0.37 | 2.46 | 1.40E-02 | 8.28E-02 |
| Nr2c2 | 656.69 | 0.91 | 0.26 | 3.52 | 4.33E-04 | 4.71E-03 |
| Mfng | 113.61 | 0.91 | 0.39 | 2.37 | 1.79E-02 | 1.00E-01 |
| Plekhh3 | 352.77 | 0.91 | 0.26 | 3.47 | 5.15E-04 | 5.43E-03 |
| Irx5 | 128.75 | 0.91 | 0.35 | 2.60 | 9.30E-03 | 6.11E-02 |
| Evc | 200.08 | 0.91 | 0.30 | 3.02 | 2.49E-03 | 2.09E-02 |
| Krba1 | 824.90 | 0.91 | 0.23 | 4.02 | 5.90E-05 | 8.02E-04 |
| Plekha6 | 3121.12 | 0.91 | 0.20 | 4.45 | 8.62E-06 | 1.44E-04 |
| 3425401B19Rik | 2508.81 | 0.91 | 0.34 | 2.69 | 7.09E-03 | 4.95E-02 |
| Map6 | 132.81 | 0.91 | 0.37 | 2.48 | 1.30E-02 | 7.84E-02 |
| Ip6k1 | 1677.82 | 0.90 | 0.21 | 4.33 | 1.47E-05 | 2.34E-04 |
| Gba2 | 426.92 | 0.90 | 0.22 | 4.03 | 5.63E-05 | 7.70E-04 |
| Phf12 | 653.08 | 0.90 | 0.23 | 3.86 | 1.12E-04 | 1.41E-03 |
| Rem1 | 137.24 | 0.90 | 0.33 | 2.72 | 6.49E-03 | 4.62E-02 |
| Ttc5 | 228.84 | 0.90 | 0.28 | 3.16 | 1.59E-03 | 1.42E-02 |
| Sox12 | 232.78 | 0.90 | 0.33 | 2.75 | 6.01E-03 | 4.34E-02 |
| Slx1b | 122.43 | 0.90 | 0.36 | 2.50 | 1.23E-02 | 7.52E-02 |
| Pitpnm3 | 155.29 | 0.90 | 0.35 | 2.57 | 1.02E-02 | 6.53E-02 |
| Tbx2 | 147.89 | 0.89 | 0.33 | 2.67 | 7.51E-03 | 5.17E-02 |
| Pdp2 | 841.85 | 0.89 | 0.29 | 3.03 | 2.41E-03 | 2.04E-02 |
| Pomgnt2 | 295.33 | 0.89 | 0.27 | 3.30 | 9.73E-04 | 9.39E-03 |
| Cbx6 | 635.75 | 0.89 | 0.21 | 4.18 | 2.96E-05 | 4.32E-04 |
| Ablim2 | 753.50 | 0.89 | 0.26 | 3.38 | 7.12E-04 | 7.16E-03 |
| Cep97 | 146.82 | 0.89 | 0.32 | 2.75 | 5.90E-03 | 4.28E-02 |
| Slc4a3 | 9093.27 | 0.89 | 0.17 | 5.36 | 8.45E-08 | 1.95E-06 |
| Kcnj3 | 2408.08 | 0.89 | 0.22 | 3.99 | 6.68E-05 | 8.94E-04 |
| Rhobtb2 | 531.45 | 0.89 | 0.26 | 3.39 | 7.07E-04 | 7.12E-03 |
| Slc26a10 | 668.90 | 0.89 | 0.27 | 3.29 | 1.01E-03 | 9.67E-03 |
| Pik3c2b | 742.93 | 0.89 | 0.23 | 3.84 | 1.24E-04 | 1.55E-03 |
| AW549877 | 986.32 | 0.89 | 0.19 | 4.59 | 4.42E-06 | 7.81E-05 |
| Asb7 | 163.12 | 0.89 | 0.34 | 2.61 | 9.17E-03 | 6.05E-02 |
| Ncoa6 | 760.29 | 0.89 | 0.21 | 4.28 | 1.83E-05 | 2.85E-04 |
| Eml3 | 263.54 | 0.88 | 0.28 | 3.12 | 1.83E-03 | 1.60E-02 |
| Rtkn | 162.80 | 0.88 | 0.30 | 2.96 | 3.10E-03 | 2.52E-02 |
| Lrrc61 | 233.28 | 0.88 | 0.29 | 3.04 | 2.40E-03 | 2.04E-02 |
| Carns1 | 1284.03 | 0.88 | 0.21 | 4.20 | 2.63E-05 | 3.88E-04 |
| Spice1 | 174.30 | 0.88 | 0.36 | 2.47 | 1.37E-02 | 8.14E-02 |
| Pla2g4e | 169.00 | 0.88 | 0.35 | 2.52 | 1.19E-02 | 7.32E-02 |
| Nadsyn1 | 107.37 | 0.87 | 0.35 | 2.49 | 1.28E-02 | 7.76E-02 |
| Stx2 | 291.26 | 0.87 | 0.24 | 3.59 | 3.28E-04 | 3.70E-03 |
| Usp11 | 216.21 | 0.87 | 0.29 | 3.03 | 2.42E-03 | 2.05E-02 |
| Slc17a5 | 147.91 | 0.87 | 0.33 | 2.61 | 8.95E-03 | 5.93E-02 |
| Slc5a6 | 363.07 | 0.87 | 0.25 | 3.51 | 4.53E-04 | 4.89E-03 |
| Bcl9l | 1094.29 | 0.87 | 0.23 | 3.77 | 1.65E-04 | 1.99E-03 |
| Pot1a | 234.89 | 0.87 | 0.26 | 3.33 | 8.72E-04 | 8.50E-03 |
| Fbxo10 | 401.61 | 0.87 | 0.29 | 2.97 | 2.96E-03 | 2.43E-02 |
| Zfp612 | 448.92 | 0.87 | 0.28 | 3.16 | 1.60E-03 | 1.43E-02 |
| Acap3 | 461.85 | 0.87 | 0.23 | 3.78 | 1.55E-04 | 1.88E-03 |
| Lrsam1 | 343.25 | 0.87 | 0.25 | 3.44 | 5.90E-04 | 6.11E-03 |
| Nckap5 | 186.91 | 0.87 | 0.31 | 2.77 | 5.52E-03 | 4.05E-02 |
| Dvl2 | 142.05 | 0.87 | 0.35 | 2.45 | 1.44E-02 | 8.45E-02 |
| Crebl2 | 325.42 | 0.86 | 0.27 | 3.22 | 1.28E-03 | 1.18E-02 |
| Gal3st2c | 158.82 | 0.86 | 0.33 | 2.60 | 9.39E-03 | 6.14E-02 |
| Ezh1 | 1079.99 | 0.86 | 0.20 | 4.32 | 1.59E-05 | 2.51E-04 |
| Nfic | 2889.91 | 0.86 | 0.17 | 5.13 | 2.89E-07 | 6.34E-06 |
| Cep164 | 252.36 | 0.86 | 0.35 | 2.45 | 1.42E-02 | 8.36E-02 |
| Szt2 | 863.54 | 0.86 | 0.20 | 4.31 | 1.63E-05 | 2.57E-04 |
| Bdh1 | 1471.38 | 0.86 | 0.24 | 3.56 | 3.70E-04 | 4.10E-03 |
| Klhdc8b | 255.02 | 0.85 | 0.30 | 2.84 | 4.46E-03 | 3.40E-02 |
| Fgf13 | 1056.14 | 0.85 | 0.25 | 3.38 | 7.35E-04 | 7.35E-03 |
| Ajuba | 120.53 | 0.85 | 0.34 | 2.52 | 1.18E-02 | 7.29E-02 |
| Rbm20 | 1218.33 | 0.85 | 0.24 | 3.50 | 4.61E-04 | 4.95E-03 |
| Hinfp | 254.42 | 0.85 | 0.32 | 2.64 | 8.38E-03 | 5.63E-02 |
| Ankzf1 | 400.19 | 0.85 | 0.22 | 3.87 | 1.09E-04 | 1.38E-03 |
| Tbc1d17 | 719.11 | 0.85 | 0.20 | 4.30 | 1.70E-05 | 2.67E-04 |
| Adamts10 | 761.28 | 0.85 | 0.22 | 3.84 | 1.22E-04 | 1.53E-03 |
| Pwwp3a | 384.48 | 0.85 | 0.26 | 3.28 | 1.05E-03 | 1.00E-02 |
| Acbd4 | 304.20 | 0.85 | 0.28 | 3.03 | 2.41E-03 | 2.05E-02 |
| Rasgrp2 | 752.15 | 0.85 | 0.24 | 3.51 | 4.53E-04 | 4.89E-03 |
| Nt5c1a | 295.67 | 0.85 | 0.32 | 2.63 | 8.46E-03 | 5.67E-02 |
| Pde7a | 1035.61 | 0.85 | 0.32 | 2.64 | 8.30E-03 | 5.59E-02 |
| Dact1 | 276.83 | 0.85 | 0.26 | 3.23 | 1.25E-03 | 1.16E-02 |
| Smtnl2 | 263.84 | 0.85 | 0.26 | 3.27 | 1.07E-03 | 1.02E-02 |
| Zfp646 | 392.18 | 0.85 | 0.30 | 2.82 | 4.76E-03 | 3.60E-02 |
| Gal3st3 | 308.22 | 0.84 | 0.26 | 3.19 | 1.44E-03 | 1.31E-02 |
| Mettl17 | 307.75 | 0.84 | 0.24 | 3.42 | 6.20E-04 | 6.37E-03 |
| Crocc | 301.97 | 0.84 | 0.31 | 2.71 | 6.73E-03 | 4.74E-02 |
| Madd | 294.12 | 0.83 | 0.32 | 2.60 | 9.23E-03 | 6.08E-02 |
| Cdan1 | 325.78 | 0.83 | 0.30 | 2.77 | 5.58E-03 | 4.08E-02 |
| Prrg3 | 177.04 | 0.83 | 0.33 | 2.51 | 1.22E-02 | 7.47E-02 |
| Abhd18 | 1734.84 | 0.83 | 0.19 | 4.39 | 1.11E-05 | 1.82E-04 |
| Pfas | 587.80 | 0.83 | 0.21 | 3.87 | 1.11E-04 | 1.39E-03 |
| Dgkq | 319.18 | 0.82 | 0.27 | 3.09 | 2.03E-03 | 1.75E-02 |
| Zer1 | 917.06 | 0.82 | 0.22 | 3.79 | 1.52E-04 | 1.85E-03 |
| Efnb3 | 1929.54 | 0.82 | 0.26 | 3.19 | 1.43E-03 | 1.30E-02 |
| Hars2 | 574.70 | 0.82 | 0.25 | 3.26 | 1.11E-03 | 1.04E-02 |
| Abtb1 | 503.11 | 0.82 | 0.21 | 3.87 | 1.11E-04 | 1.39E-03 |
| Slc2a4rg-ps | 147.15 | 0.82 | 0.31 | 2.67 | 7.70E-03 | 5.27E-02 |
| Cc2d2a | 347.24 | 0.82 | 0.27 | 2.98 | 2.85E-03 | 2.36E-02 |
| Fbxl18 | 240.11 | 0.82 | 0.30 | 2.74 | 6.10E-03 | 4.39E-02 |
| Aldh1l1 | 138.99 | 0.82 | 0.34 | 2.41 | 1.60E-02 | 9.13E-02 |
| Rere | 1554.40 | 0.81 | 0.21 | 3.90 | 9.64E-05 | 1.24E-03 |
| Podn | 469.00 | 0.81 | 0.30 | 2.71 | 6.82E-03 | 4.79E-02 |
| Pdp1 | 898.96 | 0.81 | 0.22 | 3.75 | 1.79E-04 | 2.14E-03 |
| Eps8l1 | 240.22 | 0.81 | 0.34 | 2.43 | 1.53E-02 | 8.83E-02 |
| Klhl3 | 209.97 | 0.81 | 0.28 | 2.86 | 4.27E-03 | 3.28E-02 |
| Gpam | 3791.17 | 0.81 | 0.30 | 2.74 | 6.21E-03 | 4.45E-02 |
| Foxo4 | 1365.53 | 0.81 | 0.17 | 4.75 | 2.05E-06 | 3.87E-05 |
| Ldb1 | 1105.96 | 0.81 | 0.20 | 4.13 | 3.55E-05 | 5.09E-04 |
| Fuz | 318.56 | 0.81 | 0.24 | 3.32 | 9.12E-04 | 8.85E-03 |
| Kansl3 | 1909.98 | 0.81 | 0.19 | 4.21 | 2.50E-05 | 3.71E-04 |
| Fyco1 | 12481.95 | 0.81 | 0.31 | 2.64 | 8.33E-03 | 5.61E-02 |
| Spta1 | 584.67 | 0.81 | 0.21 | 3.85 | 1.20E-04 | 1.50E-03 |
| Zfp579 | 214.55 | 0.81 | 0.31 | 2.64 | 8.38E-03 | 5.63E-02 |
| B3galt2 | 193.30 | 0.81 | 0.32 | 2.54 | 1.10E-02 | 6.89E-02 |
| Hemk1 | 423.27 | 0.81 | 0.25 | 3.29 | 1.02E-03 | 9.73E-03 |
| Klf15 | 1157.33 | 0.81 | 0.29 | 2.82 | 4.84E-03 | 3.65E-02 |
| Fmo5 | 396.28 | 0.80 | 0.26 | 3.15 | 1.65E-03 | 1.46E-02 |
| Bicra | 407.64 | 0.80 | 0.31 | 2.63 | 8.43E-03 | 5.65E-02 |
| Lclat1 | 2754.32 | 0.80 | 0.22 | 3.62 | 2.90E-04 | 3.32E-03 |
| Agtr1a | 689.51 | 0.80 | 0.30 | 2.71 | 6.64E-03 | 4.69E-02 |
| Abca6 | 249.69 | 0.80 | 0.33 | 2.42 | 1.54E-02 | 8.88E-02 |
| Nfix | 3748.42 | 0.80 | 0.21 | 3.89 | 1.01E-04 | 1.29E-03 |
| Ivd | 11839.86 | 0.80 | 0.22 | 3.60 | 3.15E-04 | 3.56E-03 |
| Cables1 | 141.62 | 0.80 | 0.33 | 2.40 | 1.62E-02 | 9.24E-02 |
| Lgals4 | 1638.01 | 0.80 | 0.24 | 3.28 | 1.05E-03 | 1.00E-02 |
| Nfatc4 | 214.57 | 0.80 | 0.32 | 2.53 | 1.14E-02 | 7.10E-02 |
| Gm10698 | 159.03 | 0.80 | 0.32 | 2.48 | 1.32E-02 | 7.95E-02 |
| Zbtb12 | 150.91 | 0.80 | 0.30 | 2.62 | 8.90E-03 | 5.91E-02 |
| Pde2a | 1489.19 | 0.80 | 0.25 | 3.14 | 1.70E-03 | 1.50E-02 |
| Zfp292 | 460.45 | 0.79 | 0.29 | 2.71 | 6.80E-03 | 4.78E-02 |
| Zfp346 | 658.68 | 0.79 | 0.22 | 3.53 | 4.13E-04 | 4.53E-03 |
| Map2k7 | 1631.15 | 0.79 | 0.21 | 3.79 | 1.52E-04 | 1.85E-03 |
| Slc25a42 | 1917.36 | 0.79 | 0.22 | 3.61 | 3.10E-04 | 3.51E-03 |
| Zfp317 | 431.31 | 0.79 | 0.23 | 3.50 | 4.73E-04 | 5.05E-03 |
| C1qtnf1 | 350.46 | 0.79 | 0.26 | 3.05 | 2.29E-03 | 1.96E-02 |
| Tnrc6c | 1653.84 | 0.78 | 0.25 | 3.14 | 1.71E-03 | 1.51E-02 |
| Ypel3 | 955.34 | 0.78 | 0.18 | 4.25 | 2.18E-05 | 3.29E-04 |
| Kcnj12 | 516.62 | 0.78 | 0.23 | 3.44 | 5.89E-04 | 6.11E-03 |
| Slc16a7 | 174.53 | 0.78 | 0.29 | 2.66 | 7.77E-03 | 5.30E-02 |
| Ganc | 505.61 | 0.78 | 0.27 | 2.94 | 3.31E-03 | 2.67E-02 |
| Angptl2 | 1929.36 | 0.78 | 0.20 | 3.91 | 9.10E-05 | 1.19E-03 |
| Six5 | 300.24 | 0.78 | 0.24 | 3.26 | 1.10E-03 | 1.04E-02 |
| Lrwd1 | 213.73 | 0.78 | 0.27 | 2.86 | 4.20E-03 | 3.24E-02 |
| Ehd2 | 2088.52 | 0.78 | 0.18 | 4.27 | 1.96E-05 | 3.00E-04 |
| Hdac9 | 484.75 | 0.78 | 0.23 | 3.44 | 5.78E-04 | 6.01E-03 |
| Rhobtb1 | 3907.23 | 0.78 | 0.28 | 2.77 | 5.66E-03 | 4.14E-02 |
| Gja3 | 443.56 | 0.77 | 0.24 | 3.17 | 1.54E-03 | 1.39E-02 |
| Zfyve27 | 615.04 | 0.77 | 0.20 | 3.93 | 8.42E-05 | 1.11E-03 |
| Ttc13 | 310.58 | 0.77 | 0.31 | 2.48 | 1.31E-02 | 7.89E-02 |
| Ebf2 | 233.60 | 0.77 | 0.28 | 2.75 | 5.93E-03 | 4.30E-02 |
| Shld1 | 457.42 | 0.77 | 0.24 | 3.15 | 1.64E-03 | 1.46E-02 |
| Bcl7a | 326.46 | 0.76 | 0.27 | 2.87 | 4.14E-03 | 3.20E-02 |
| Dgcr8 | 292.99 | 0.76 | 0.28 | 2.76 | 5.71E-03 | 4.16E-02 |
| Zbtb40 | 310.90 | 0.76 | 0.29 | 2.66 | 7.90E-03 | 5.38E-02 |
| Kcnj5 | 1619.23 | 0.76 | 0.17 | 4.43 | 9.38E-06 | 1.55E-04 |
| Tmem44 | 241.10 | 0.76 | 0.30 | 2.56 | 1.05E-02 | 6.69E-02 |
| Mbd6 | 830.82 | 0.76 | 0.21 | 3.68 | 2.29E-04 | 2.67E-03 |
| Appl2 | 1606.05 | 0.76 | 0.29 | 2.59 | 9.53E-03 | 6.20E-02 |
| Wdr6 | 329.04 | 0.76 | 0.27 | 2.83 | 4.66E-03 | 3.54E-02 |
| Pold1 | 321.05 | 0.75 | 0.27 | 2.81 | 5.03E-03 | 3.77E-02 |
| Abhd4 | 319.28 | 0.75 | 0.27 | 2.82 | 4.85E-03 | 3.65E-02 |
| Maf1 | 1643.92 | 0.75 | 0.19 | 4.03 | 5.63E-05 | 7.70E-04 |
| Vps33a | 645.60 | 0.75 | 0.21 | 3.51 | 4.46E-04 | 4.83E-03 |
| Neurl4 | 1413.72 | 0.75 | 0.24 | 3.13 | 1.73E-03 | 1.53E-02 |
| Celsr2 | 253.39 | 0.75 | 0.28 | 2.67 | 7.52E-03 | 5.18E-02 |
| Dgke | 539.30 | 0.75 | 0.24 | 3.16 | 1.59E-03 | 1.42E-02 |
| Tmpo | 897.85 | 0.75 | 0.24 | 3.15 | 1.61E-03 | 1.44E-02 |
| Selenbp1 | 3055.78 | 0.75 | 0.18 | 4.05 | 5.21E-05 | 7.23E-04 |
| LTO1 | 274.06 | 0.75 | 0.29 | 2.55 | 1.07E-02 | 6.79E-02 |
| Bcorl1 | 212.07 | 0.75 | 0.29 | 2.56 | 1.06E-02 | 6.73E-02 |
| Epha7 | 174.38 | 0.75 | 0.29 | 2.54 | 1.10E-02 | 6.89E-02 |
| Dyrk1b | 427.77 | 0.75 | 0.27 | 2.81 | 5.02E-03 | 3.76E-02 |
| Arhgef17 | 2422.38 | 0.75 | 0.21 | 3.52 | 4.32E-04 | 4.71E-03 |
| Zbtb4 | 1427.78 | 0.74 | 0.17 | 4.35 | 1.36E-05 | 2.17E-04 |
| Zfp710 | 532.58 | 0.74 | 0.23 | 3.18 | 1.46E-03 | 1.32E-02 |
| Rsad1 | 391.00 | 0.74 | 0.23 | 3.28 | 1.05E-03 | 1.00E-02 |
| Ago1 | 439.69 | 0.74 | 0.29 | 2.58 | 9.95E-03 | 6.43E-02 |
| Wipf3 | 1226.42 | 0.74 | 0.21 | 3.46 | 5.33E-04 | 5.59E-03 |
| Pde4c | 279.99 | 0.74 | 0.27 | 2.79 | 5.31E-03 | 3.93E-02 |
| Armc2 | 887.66 | 0.74 | 0.26 | 2.88 | 4.01E-03 | 3.12E-02 |
| Cenpf | 1231.36 | 0.74 | 0.29 | 2.51 | 1.20E-02 | 7.39E-02 |
| Tchp | 172.08 | 0.74 | 0.30 | 2.45 | 1.42E-02 | 8.38E-02 |
| Rrnad1 | 220.69 | 0.74 | 0.28 | 2.64 | 8.19E-03 | 5.53E-02 |
| Ttc33 | 517.31 | 0.74 | 0.26 | 2.85 | 4.40E-03 | 3.37E-02 |
| Pde4dip | 48813.39 | 0.74 | 0.18 | 4.00 | 6.41E-05 | 8.65E-04 |
| Trim68 | 328.79 | 0.74 | 0.24 | 3.06 | 2.24E-03 | 1.92E-02 |
| Fbf1 | 442.48 | 0.73 | 0.24 | 3.00 | 2.68E-03 | 2.22E-02 |
| Ogt | 2294.93 | 0.73 | 0.22 | 3.29 | 9.96E-04 | 9.58E-03 |
| Bahcc1 | 448.42 | 0.73 | 0.30 | 2.44 | 1.47E-02 | 8.58E-02 |
| Rbm10 | 556.63 | 0.73 | 0.21 | 3.42 | 6.21E-04 | 6.38E-03 |
| Wdr81 | 343.01 | 0.73 | 0.26 | 2.79 | 5.30E-03 | 3.93E-02 |
| Kcnd3 | 418.06 | 0.73 | 0.29 | 2.50 | 1.24E-02 | 7.57E-02 |
| Rprd2 | 807.70 | 0.73 | 0.21 | 3.40 | 6.73E-04 | 6.81E-03 |
| Plekhg5 | 575.08 | 0.73 | 0.21 | 3.53 | 4.17E-04 | 4.56E-03 |
| Asb15 | 2116.13 | 0.73 | 0.26 | 2.78 | 5.39E-03 | 3.98E-02 |
| Ndor1 | 300.09 | 0.73 | 0.28 | 2.59 | 9.46E-03 | 6.17E-02 |
| Gnpda2 | 245.64 | 0.73 | 0.28 | 2.59 | 9.72E-03 | 6.31E-02 |
| Kmt5c | 250.68 | 0.72 | 0.27 | 2.70 | 7.04E-03 | 4.92E-02 |
| Mitf | 1083.77 | 0.72 | 0.22 | 3.25 | 1.17E-03 | 1.10E-02 |
| Wee1 | 390.66 | 0.72 | 0.26 | 2.74 | 6.11E-03 | 4.39E-02 |
| Cpeb3 | 2699.47 | 0.72 | 0.27 | 2.66 | 7.76E-03 | 5.30E-02 |
| 0610030E20Rik | 280.65 | 0.72 | 0.26 | 2.79 | 5.34E-03 | 3.95E-02 |
| Mtx3 | 312.69 | 0.72 | 0.27 | 2.64 | 8.20E-03 | 5.54E-02 |
| Gm5113 | 264.47 | 0.72 | 0.27 | 2.66 | 7.74E-03 | 5.29E-02 |
| Nnt | 12040.43 | 0.72 | 0.20 | 3.52 | 4.35E-04 | 4.73E-03 |
| Gle1 | 678.88 | 0.72 | 0.20 | 3.51 | 4.47E-04 | 4.84E-03 |
| Trim41 | 759.95 | 0.72 | 0.23 | 3.19 | 1.43E-03 | 1.30E-02 |
| Dhrs11 | 1449.09 | 0.72 | 0.22 | 3.29 | 1.01E-03 | 9.67E-03 |
| Lamb3 | 449.03 | 0.72 | 0.26 | 2.80 | 5.07E-03 | 3.79E-02 |
| Rcor3 | 538.00 | 0.71 | 0.27 | 2.63 | 8.49E-03 | 5.68E-02 |
| Ptpdc1 | 224.32 | 0.71 | 0.29 | 2.46 | 1.38E-02 | 8.21E-02 |
| Irx4 | 464.28 | 0.71 | 0.29 | 2.46 | 1.38E-02 | 8.19E-02 |
| Myom2 | 22291.02 | 0.71 | 0.25 | 2.81 | 4.88E-03 | 3.67E-02 |
| Foxk1 | 572.14 | 0.71 | 0.21 | 3.43 | 5.96E-04 | 6.16E-03 |
| Ctc1 | 415.15 | 0.71 | 0.23 | 3.03 | 2.42E-03 | 2.05E-02 |
| Wnk2 | 2915.79 | 0.71 | 0.26 | 2.70 | 6.89E-03 | 4.83E-02 |
| Npc1 | 1502.39 | 0.71 | 0.23 | 3.06 | 2.22E-03 | 1.90E-02 |
| Ppp6r2 | 1355.08 | 0.71 | 0.25 | 2.78 | 5.42E-03 | 4.00E-02 |
| Zscan26 | 887.72 | 0.71 | 0.22 | 3.17 | 1.54E-03 | 1.39E-02 |
| Mef2c | 1290.78 | 0.71 | 0.22 | 3.27 | 1.09E-03 | 1.04E-02 |
| Pla2g5 | 1278.57 | 0.71 | 0.25 | 2.81 | 5.02E-03 | 3.76E-02 |
| Zfp692 | 222.38 | 0.71 | 0.30 | 2.38 | 1.75E-02 | 9.78E-02 |
| Tfpi | 1601.48 | 0.71 | 0.19 | 3.67 | 2.40E-04 | 2.78E-03 |
| Ushbp1 | 1452.04 | 0.70 | 0.18 | 4.00 | 6.25E-05 | 8.48E-04 |
| Rab11fip2 | 210.53 | 0.70 | 0.29 | 2.45 | 1.43E-02 | 8.41E-02 |
| Thbs3 | 312.93 | 0.70 | 0.25 | 2.84 | 4.45E-03 | 3.40E-02 |
| Cep85l | 726.04 | 0.70 | 0.26 | 2.68 | 7.38E-03 | 5.11E-02 |
| Zfp740 | 929.13 | 0.70 | 0.21 | 3.33 | 8.62E-04 | 8.42E-03 |
| Traf2 | 544.41 | -0.70 | 0.24 | -2.92 | 3.45E-03 | 2.76E-02 |
| Rybp | 753.79 | -0.70 | 0.20 | -3.43 | 5.94E-04 | 6.14E-03 |
| Chd9 | 2074.07 | -0.70 | 0.23 | -3.09 | 1.98E-03 | 1.72E-02 |
| Cd82 | 370.19 | -0.70 | 0.22 | -3.16 | 1.55E-03 | 1.40E-02 |
| Adam17 | 1355.79 | -0.71 | 0.19 | -3.62 | 2.97E-04 | 3.39E-03 |
| Ddr2 | 1366.61 | -0.71 | 0.28 | -2.51 | 1.21E-02 | 7.43E-02 |
| Dennd2a | 492.55 | -0.71 | 0.29 | -2.42 | 1.55E-02 | 8.95E-02 |
| Oser1 | 649.60 | -0.71 | 0.20 | -3.60 | 3.16E-04 | 3.57E-03 |
| Tra2b | 1710.23 | -0.71 | 0.17 | -4.18 | 2.98E-05 | 4.33E-04 |
| Pcf11 | 1357.17 | -0.71 | 0.19 | -3.81 | 1.41E-04 | 1.74E-03 |
| Nfatc2 | 804.76 | -0.71 | 0.28 | -2.57 | 1.00E-02 | 6.47E-02 |
| Ablim3 | 585.47 | -0.71 | 0.21 | -3.37 | 7.42E-04 | 7.41E-03 |
| Ipmk | 1049.92 | -0.71 | 0.24 | -2.95 | 3.19E-03 | 2.58E-02 |
| Rbpj | 1453.42 | -0.71 | 0.18 | -3.92 | 8.87E-05 | 1.16E-03 |
| Cers6 | 215.68 | -0.71 | 0.29 | -2.42 | 1.56E-02 | 8.98E-02 |
| Nuak2 | 703.56 | -0.71 | 0.22 | -3.25 | 1.15E-03 | 1.08E-02 |
| Scyl2 | 876.86 | -0.71 | 0.20 | -3.53 | 4.21E-04 | 4.59E-03 |
| Tra2a | 1638.08 | -0.71 | 0.18 | -3.94 | 8.25E-05 | 1.09E-03 |
| Adam9 | 2099.90 | -0.71 | 0.18 | -3.93 | 8.54E-05 | 1.12E-03 |
| Spata6 | 250.06 | -0.71 | 0.26 | -2.72 | 6.53E-03 | 4.63E-02 |
| Naf1 | 337.54 | -0.71 | 0.23 | -3.04 | 2.34E-03 | 1.99E-02 |
| Hnrnpf | 4749.97 | -0.71 | 0.21 | -3.38 | 7.25E-04 | 7.27E-03 |
| mt-Nd4 | 755904.06 | -0.72 | 0.21 | -3.41 | 6.49E-04 | 6.61E-03 |
| Trafd1 | 2399.18 | -0.72 | 0.24 | -3.03 | 2.43E-03 | 2.05E-02 |
| Hcn4 | 383.06 | -0.72 | 0.28 | -2.60 | 9.33E-03 | 6.12E-02 |
| Coq10b | 1714.04 | -0.72 | 0.22 | -3.31 | 9.17E-04 | 8.88E-03 |
| Rbm19 | 467.82 | -0.72 | 0.21 | -3.38 | 7.12E-04 | 7.16E-03 |
| Atp6v1a | 998.43 | -0.72 | 0.21 | -3.48 | 4.97E-04 | 5.28E-03 |
| Mast4 | 1396.93 | -0.72 | 0.21 | -3.37 | 7.42E-04 | 7.41E-03 |
| Rbm18 | 1330.45 | -0.72 | 0.28 | -2.56 | 1.04E-02 | 6.63E-02 |
| Tanc2 | 385.56 | -0.72 | 0.30 | -2.44 | 1.48E-02 | 8.61E-02 |
| Ccnd1 | 1026.85 | -0.72 | 0.19 | -3.82 | 1.34E-04 | 1.66E-03 |
| Plxnd1 | 3497.07 | -0.72 | 0.22 | -3.26 | 1.13E-03 | 1.06E-02 |
| Shisa5 | 1049.70 | -0.72 | 0.20 | -3.58 | 3.48E-04 | 3.89E-03 |
| Hecw2 | 544.14 | -0.73 | 0.30 | -2.43 | 1.49E-02 | 8.67E-02 |
| Frmd4b | 909.60 | -0.73 | 0.25 | -2.97 | 2.98E-03 | 2.45E-02 |
| Spsb1 | 1307.94 | -0.73 | 0.17 | -4.19 | 2.80E-05 | 4.11E-04 |
| Slc23a2 | 974.00 | -0.73 | 0.22 | -3.35 | 7.98E-04 | 7.89E-03 |
| Tspan6 | 148.59 | -0.73 | 0.31 | -2.37 | 1.77E-02 | 9.90E-02 |
| Cct3 | 3374.21 | -0.73 | 0.24 | -2.99 | 2.81E-03 | 2.33E-02 |
| Ubb-ps | 1628.84 | -0.73 | 0.21 | -3.42 | 6.28E-04 | 6.44E-03 |
| Med7 | 364.65 | -0.73 | 0.27 | -2.71 | 6.75E-03 | 4.75E-02 |
| Fn1 | 2471.30 | -0.73 | 0.22 | -3.26 | 1.11E-03 | 1.05E-02 |
| Tor1aip2 | 1713.14 | -0.73 | 0.20 | -3.58 | 3.38E-04 | 3.80E-03 |
| Galnt16 | 190.61 | -0.73 | 0.30 | -2.45 | 1.43E-02 | 8.43E-02 |
| Erbin | 2458.58 | -0.73 | 0.20 | -3.74 | 1.86E-04 | 2.21E-03 |
| Plod3 | 1248.66 | -0.73 | 0.26 | -2.78 | 5.48E-03 | 4.03E-02 |
| Tnrc6b | 1337.44 | -0.73 | 0.18 | -4.17 | 3.08E-05 | 4.46E-04 |
| Ppp1r2 | 4407.80 | -0.73 | 0.17 | -4.22 | 2.43E-05 | 3.62E-04 |
| mt-Atp8 | 65192.25 | -0.74 | 0.20 | -3.61 | 3.09E-04 | 3.50E-03 |
| Eif2s2 | 2566.27 | -0.74 | 0.21 | -3.51 | 4.56E-04 | 4.90E-03 |
| Samhd1 | 1136.05 | -0.74 | 0.22 | -3.36 | 7.87E-04 | 7.80E-03 |
| Zbtb11 | 786.94 | -0.74 | 0.20 | -3.75 | 1.78E-04 | 2.13E-03 |
| Chmp4b | 3422.46 | -0.74 | 0.21 | -3.48 | 5.01E-04 | 5.31E-03 |
| Dnttip2 | 851.72 | -0.74 | 0.21 | -3.50 | 4.62E-04 | 4.96E-03 |
| Loxl1 | 1134.16 | -0.74 | 0.26 | -2.87 | 4.06E-03 | 3.16E-02 |
| Tmem128 | 295.05 | -0.74 | 0.26 | -2.88 | 4.03E-03 | 3.14E-02 |
| Zfp568 | 694.78 | -0.74 | 0.23 | -3.28 | 1.05E-03 | 1.00E-02 |
| mt-Nd3 | 62309.24 | -0.74 | 0.21 | -3.55 | 3.90E-04 | 4.30E-03 |
| Osgin2 | 296.03 | -0.74 | 0.27 | -2.75 | 5.97E-03 | 4.32E-02 |
| Sod3 | 1147.02 | -0.74 | 0.24 | -3.05 | 2.31E-03 | 1.97E-02 |
| Mindy3 | 331.01 | -0.75 | 0.24 | -3.16 | 1.59E-03 | 1.42E-02 |
| Actr3 | 3932.73 | -0.75 | 0.18 | -4.17 | 3.09E-05 | 4.47E-04 |
| Maml1 | 864.80 | -0.75 | 0.21 | -3.48 | 4.99E-04 | 5.29E-03 |
| Tmem243 | 249.54 | -0.75 | 0.26 | -2.92 | 3.48E-03 | 2.77E-02 |
| Pisd | 1018.11 | -0.75 | 0.19 | -3.89 | 9.85E-05 | 1.27E-03 |
| 4930453N24Rik | 538.14 | -0.75 | 0.23 | -3.27 | 1.06E-03 | 1.01E-02 |
| Aen | 998.27 | -0.75 | 0.20 | -3.80 | 1.42E-04 | 1.74E-03 |
| mt-Cytb | 891170.08 | -0.75 | 0.18 | -4.27 | 1.93E-05 | 2.98E-04 |
| Zfp217 | 637.22 | -0.75 | 0.24 | -3.16 | 1.57E-03 | 1.41E-02 |
| Rapgef5 | 1585.27 | -0.75 | 0.20 | -3.73 | 1.95E-04 | 2.30E-03 |
| Etnk1 | 1771.91 | -0.76 | 0.24 | -3.19 | 1.45E-03 | 1.32E-02 |
| Ror1 | 197.86 | -0.76 | 0.30 | -2.51 | 1.20E-02 | 7.38E-02 |
| Mvp | 2360.46 | -0.76 | 0.18 | -4.30 | 1.72E-05 | 2.70E-04 |
| Tirap | 250.12 | -0.76 | 0.28 | -2.68 | 7.34E-03 | 5.09E-02 |
| Fancc | 171.49 | -0.76 | 0.30 | -2.52 | 1.19E-02 | 7.31E-02 |
| Commd1b | 190.36 | -0.76 | 0.31 | -2.43 | 1.49E-02 | 8.69E-02 |
| Cd163 | 1126.49 | -0.76 | 0.24 | -3.21 | 1.33E-03 | 1.22E-02 |
| Capza1 | 1276.06 | -0.76 | 0.19 | -4.10 | 4.04E-05 | 5.72E-04 |
| Uaca | 2720.68 | -0.76 | 0.17 | -4.44 | 8.82E-06 | 1.47E-04 |
| Stat5a | 1017.66 | -0.76 | 0.20 | -3.88 | 1.06E-04 | 1.34E-03 |
| Fam98b | 818.61 | -0.77 | 0.20 | -3.91 | 9.06E-05 | 1.18E-03 |
| mt-Co1 | 2830746.01 | -0.77 | 0.18 | -4.25 | 2.12E-05 | 3.21E-04 |
| Plpp3 | 3505.37 | -0.77 | 0.20 | -3.84 | 1.22E-04 | 1.53E-03 |
| Bmp2k | 353.69 | -0.77 | 0.26 | -3.01 | 2.65E-03 | 2.20E-02 |
| Mustn1 | 236.22 | -0.77 | 0.32 | -2.39 | 1.70E-02 | 9.61E-02 |
| Myl1 | 734.96 | -0.77 | 0.26 | -2.92 | 3.52E-03 | 2.81E-02 |
| Lpin2 | 457.54 | -0.77 | 0.23 | -3.33 | 8.63E-04 | 8.43E-03 |
| Tlnrd1 | 918.89 | -0.77 | 0.26 | -2.97 | 2.99E-03 | 2.45E-02 |
| Tubb2a | 1310.15 | -0.77 | 0.26 | -3.00 | 2.73E-03 | 2.26E-02 |
| C1qc | 933.71 | -0.77 | 0.28 | -2.76 | 5.85E-03 | 4.24E-02 |
| Psmb10 | 757.31 | -0.77 | 0.22 | -3.56 | 3.68E-04 | 4.09E-03 |
| Adss | 509.55 | -0.77 | 0.26 | -3.03 | 2.48E-03 | 2.09E-02 |
| Dgat1 | 690.76 | -0.78 | 0.29 | -2.71 | 6.68E-03 | 4.72E-02 |
| 2410002F23Rik | 644.56 | -0.78 | 0.24 | -3.22 | 1.27E-03 | 1.17E-02 |
| Col1a2 | 5200.49 | -0.78 | 0.27 | -2.90 | 3.72E-03 | 2.94E-02 |
| Pid1 | 339.92 | -0.78 | 0.24 | -3.18 | 1.49E-03 | 1.35E-02 |
| Plagl2 | 236.02 | -0.78 | 0.31 | -2.52 | 1.16E-02 | 7.21E-02 |
| Gm49273 | 820.35 | -0.78 | 0.28 | -2.79 | 5.20E-03 | 3.88E-02 |
| Nup54 | 516.85 | -0.78 | 0.23 | -3.35 | 8.13E-04 | 8.01E-03 |
| Eps8 | 695.37 | -0.78 | 0.32 | -2.48 | 1.31E-02 | 7.90E-02 |
| Kcna5 | 519.91 | -0.78 | 0.25 | -3.12 | 1.81E-03 | 1.58E-02 |
| mt-Atp6 | 658746.49 | -0.78 | 0.18 | -4.38 | 1.21E-05 | 1.96E-04 |
| S1pr1 | 2044.94 | -0.78 | 0.19 | -4.21 | 2.59E-05 | 3.82E-04 |
| Nadk | 1053.01 | -0.79 | 0.18 | -4.26 | 2.09E-05 | 3.16E-04 |
| Cntn2 | 376.31 | -0.79 | 0.27 | -2.94 | 3.28E-03 | 2.65E-02 |
| Pla2g7 | 216.36 | -0.79 | 0.32 | -2.49 | 1.29E-02 | 7.79E-02 |
| Atp13a3 | 1665.89 | -0.79 | 0.19 | -4.26 | 2.04E-05 | 3.11E-04 |
| Septin11 | 1101.15 | -0.79 | 0.20 | -4.03 | 5.64E-05 | 7.71E-04 |
| Fzd1 | 362.14 | -0.79 | 0.25 | -3.17 | 1.54E-03 | 1.39E-02 |
| Foxo1 | 1181.39 | -0.79 | 0.19 | -4.22 | 2.42E-05 | 3.62E-04 |
| Lnx2 | 471.40 | -0.79 | 0.24 | -3.28 | 1.03E-03 | 9.90E-03 |
| Arl4c | 355.01 | -0.79 | 0.28 | -2.87 | 4.08E-03 | 3.16E-02 |
| Picalm | 6642.03 | -0.80 | 0.17 | -4.81 | 1.52E-06 | 2.93E-05 |
| Pikfyve | 368.84 | -0.80 | 0.25 | -3.14 | 1.69E-03 | 1.50E-02 |
| Rbm7 | 679.59 | -0.80 | 0.21 | -3.83 | 1.28E-04 | 1.59E-03 |
| Xylt1 | 154.58 | -0.80 | 0.32 | -2.47 | 1.37E-02 | 8.17E-02 |
| Tmsb4x | 5224.78 | -0.80 | 0.28 | -2.84 | 4.52E-03 | 3.44E-02 |
| Irf5 | 253.01 | -0.80 | 0.27 | -2.91 | 3.62E-03 | 2.88E-02 |
| Rgs4 | 501.41 | -0.80 | 0.26 | -3.13 | 1.75E-03 | 1.54E-02 |
| Rrs1 | 719.73 | -0.80 | 0.23 | -3.43 | 6.02E-04 | 6.20E-03 |
| Col16a1 | 425.26 | -0.80 | 0.33 | -2.39 | 1.68E-02 | 9.48E-02 |
| Cyth3 | 1539.87 | -0.80 | 0.23 | -3.55 | 3.88E-04 | 4.30E-03 |
| Shb | 751.22 | -0.80 | 0.24 | -3.39 | 7.06E-04 | 7.12E-03 |
| Tmem123 | 1762.38 | -0.80 | 0.18 | -4.49 | 7.09E-06 | 1.21E-04 |
| Tmbim1 | 2773.00 | -0.80 | 0.28 | -2.86 | 4.30E-03 | 3.30E-02 |
| Wdr43 | 771.92 | -0.80 | 0.21 | -3.87 | 1.09E-04 | 1.38E-03 |
| Ubb | 22679.77 | -0.81 | 0.18 | -4.38 | 1.20E-05 | 1.94E-04 |
| Ckap4 | 776.03 | -0.81 | 0.23 | -3.50 | 4.58E-04 | 4.92E-03 |
| Fmr1 | 675.38 | -0.81 | 0.25 | -3.26 | 1.10E-03 | 1.04E-02 |
| Arpc3 | 1687.14 | -0.81 | 0.23 | -3.51 | 4.55E-04 | 4.90E-03 |
| Klf11 | 540.97 | -0.81 | 0.22 | -3.68 | 2.30E-04 | 2.67E-03 |
| Bgn | 5968.94 | -0.81 | 0.25 | -3.26 | 1.11E-03 | 1.04E-02 |
| Zc3h7a | 1333.69 | -0.81 | 0.18 | -4.51 | 6.49E-06 | 1.11E-04 |
| Ptma | 7555.61 | -0.81 | 0.21 | -3.81 | 1.39E-04 | 1.71E-03 |
| Hspa8 | 64078.21 | -0.81 | 0.20 | -4.04 | 5.35E-05 | 7.39E-04 |
| Impdh2 | 1738.92 | -0.81 | 0.24 | -3.32 | 8.88E-04 | 8.64E-03 |
| Cnnm4 | 448.86 | -0.81 | 0.22 | -3.67 | 2.39E-04 | 2.77E-03 |
| Edem1 | 687.24 | -0.81 | 0.23 | -3.48 | 4.98E-04 | 5.28E-03 |
| Laptm5 | 497.82 | -0.81 | 0.25 | -3.30 | 9.81E-04 | 9.47E-03 |
| Col1a1 | 3517.43 | -0.81 | 0.32 | -2.58 | 9.82E-03 | 6.36E-02 |
| Gpatch4 | 331.97 | -0.81 | 0.30 | -2.69 | 7.23E-03 | 5.02E-02 |
| Arid5b | 2804.67 | -0.81 | 0.31 | -2.66 | 7.75E-03 | 5.29E-02 |
| Slc22a15 | 225.87 | -0.81 | 0.29 | -2.78 | 5.37E-03 | 3.97E-02 |
| Ninj1 | 1394.03 | -0.82 | 0.21 | -3.82 | 1.36E-04 | 1.68E-03 |
| Bbc3 | 172.22 | -0.82 | 0.31 | -2.66 | 7.83E-03 | 5.34E-02 |
| Snx7 | 225.33 | -0.82 | 0.27 | -3.00 | 2.68E-03 | 2.23E-02 |
| Brix1 | 502.05 | -0.82 | 0.28 | -2.87 | 4.12E-03 | 3.19E-02 |
| Cstf3 | 501.47 | -0.82 | 0.29 | -2.78 | 5.37E-03 | 3.97E-02 |
| Nip7 | 352.69 | -0.82 | 0.31 | -2.61 | 9.00E-03 | 5.96E-02 |
| Serping1 | 3417.25 | -0.82 | 0.22 | -3.73 | 1.89E-04 | 2.24E-03 |
| Sgpl1 | 913.05 | -0.82 | 0.20 | -4.17 | 3.06E-05 | 4.44E-04 |
| Rgcc | 1011.86 | -0.82 | 0.31 | -2.65 | 8.04E-03 | 5.45E-02 |
| Carmil1 | 156.00 | -0.82 | 0.32 | -2.54 | 1.12E-02 | 7.02E-02 |
| Tnks1bp1 | 2365.35 | -0.82 | 0.18 | -4.58 | 4.60E-06 | 8.06E-05 |
| Nfkbid | 536.54 | -0.82 | 0.23 | -3.64 | 2.74E-04 | 3.15E-03 |
| Crybg1 | 209.96 | -0.82 | 0.34 | -2.42 | 1.56E-02 | 8.99E-02 |
| Igfbp7 | 4414.13 | -0.82 | 0.23 | -3.60 | 3.24E-04 | 3.65E-03 |
| Mapre1 | 2302.49 | -0.82 | 0.17 | -4.74 | 2.16E-06 | 4.06E-05 |
| Dlgap4 | 2487.34 | -0.83 | 0.18 | -4.47 | 7.95E-06 | 1.34E-04 |
| Shank3 | 719.50 | -0.83 | 0.28 | -2.96 | 3.07E-03 | 2.50E-02 |
| Pxn | 2055.72 | -0.83 | 0.21 | -3.96 | 7.56E-05 | 1.01E-03 |
| Morf4l2 | 2395.73 | -0.83 | 0.21 | -3.90 | 9.47E-05 | 1.23E-03 |
| Timm8a1 | 355.47 | -0.83 | 0.29 | -2.90 | 3.68E-03 | 2.91E-02 |
| Ccn3 | 238.10 | -0.83 | 0.34 | -2.45 | 1.43E-02 | 8.40E-02 |
| Smc1a | 2133.78 | -0.83 | 0.26 | -3.16 | 1.55E-03 | 1.40E-02 |
| Yes1 | 695.89 | -0.83 | 0.20 | -4.09 | 4.40E-05 | 6.19E-04 |
| Trp53inp1 | 480.36 | -0.83 | 0.33 | -2.53 | 1.13E-02 | 7.08E-02 |
| Sfpq | 3367.84 | -0.83 | 0.20 | -4.18 | 2.96E-05 | 4.32E-04 |
| Arf2 | 1026.22 | -0.83 | 0.22 | -3.87 | 1.10E-04 | 1.39E-03 |
| Fam91a1 | 735.18 | -0.83 | 0.21 | -3.92 | 8.94E-05 | 1.17E-03 |
| Csf2ra | 414.83 | -0.83 | 0.24 | -3.53 | 4.09E-04 | 4.49E-03 |
| Zfp719 | 204.54 | -0.83 | 0.31 | -2.67 | 7.64E-03 | 5.24E-02 |
| B4galt3 | 485.86 | -0.83 | 0.24 | -3.44 | 5.89E-04 | 6.11E-03 |
| H2-T22 | 527.88 | -0.83 | 0.24 | -3.42 | 6.22E-04 | 6.38E-03 |
| Dusp8 | 2415.29 | -0.84 | 0.19 | -4.37 | 1.26E-05 | 2.04E-04 |
| Nme1 | 341.12 | -0.84 | 0.29 | -2.91 | 3.65E-03 | 2.89E-02 |
| Rps12 | 342.81 | -0.84 | 0.29 | -2.90 | 3.77E-03 | 2.97E-02 |
| Nhp2 | 369.90 | -0.84 | 0.30 | -2.79 | 5.20E-03 | 3.88E-02 |
| Tbrg1 | 1063.51 | -0.84 | 0.20 | -4.27 | 1.95E-05 | 3.00E-04 |
| Ctsz | 874.18 | -0.84 | 0.30 | -2.78 | 5.36E-03 | 3.96E-02 |
| Pdcd7 | 532.92 | -0.84 | 0.32 | -2.59 | 9.50E-03 | 6.19E-02 |
| Chic2 | 564.97 | -0.84 | 0.27 | -3.08 | 2.10E-03 | 1.81E-02 |
| Anxa5 | 3259.62 | -0.84 | 0.25 | -3.37 | 7.41E-04 | 7.40E-03 |
| mt-Nd2 | 496834.02 | -0.84 | 0.20 | -4.27 | 1.95E-05 | 3.00E-04 |
| Col5a3 | 1411.47 | -0.84 | 0.30 | -2.83 | 4.63E-03 | 3.52E-02 |
| mt-Nd1 | 643475.44 | -0.84 | 0.18 | -4.81 | 1.51E-06 | 2.92E-05 |
| Tmem33 | 1062.77 | -0.84 | 0.18 | -4.62 | 3.86E-06 | 6.89E-05 |
| H2-T23 | 863.17 | -0.85 | 0.24 | -3.49 | 4.81E-04 | 5.12E-03 |
| Slit3 | 643.56 | -0.85 | 0.23 | -3.65 | 2.62E-04 | 3.01E-03 |
| Tjp2 | 1053.66 | -0.85 | 0.23 | -3.77 | 1.66E-04 | 2.00E-03 |
| Srpx2 | 169.13 | -0.85 | 0.32 | -2.66 | 7.79E-03 | 5.31E-02 |
| Top1 | 2583.12 | -0.85 | 0.19 | -4.57 | 4.88E-06 | 8.51E-05 |
| Rbm12 | 510.65 | -0.85 | 0.24 | -3.61 | 3.09E-04 | 3.50E-03 |
| Tex30 | 123.68 | -0.85 | 0.33 | -2.60 | 9.26E-03 | 6.09E-02 |
| Nepro | 179.21 | -0.86 | 0.35 | -2.43 | 1.53E-02 | 8.83E-02 |
| Xirp1 | 22308.32 | -0.86 | 0.23 | -3.78 | 1.56E-04 | 1.90E-03 |
| Hivep3 | 372.28 | -0.86 | 0.34 | -2.55 | 1.09E-02 | 6.85E-02 |
| Sec24a | 732.15 | -0.86 | 0.20 | -4.27 | 1.92E-05 | 2.97E-04 |
| Ero1l | 779.00 | -0.86 | 0.22 | -3.92 | 8.87E-05 | 1.16E-03 |
| Tbk1 | 878.73 | -0.86 | 0.23 | -3.75 | 1.73E-04 | 2.08E-03 |
| mt-Co2 | 856608.21 | -0.86 | 0.20 | -4.29 | 1.77E-05 | 2.77E-04 |
| Prkx | 1129.17 | -0.86 | 0.19 | -4.56 | 5.21E-06 | 9.05E-05 |
| Ccnyl1 | 769.89 | -0.86 | 0.21 | -4.04 | 5.38E-05 | 7.43E-04 |
| Sulf1 | 955.42 | -0.86 | 0.27 | -3.24 | 1.21E-03 | 1.13E-02 |
| Gpkow | 915.53 | -0.86 | 0.18 | -4.66 | 3.09E-06 | 5.64E-05 |
| Kri1 | 632.88 | -0.86 | 0.26 | -3.35 | 8.14E-04 | 8.02E-03 |
| Chka | 488.68 | -0.86 | 0.22 | -3.99 | 6.70E-05 | 8.96E-04 |
| Arl6ip1 | 764.79 | -0.86 | 0.27 | -3.18 | 1.49E-03 | 1.35E-02 |
| Slc2a1 | 872.22 | -0.86 | 0.32 | -2.72 | 6.58E-03 | 4.66E-02 |
| Slc45a3 | 140.51 | -0.86 | 0.31 | -2.76 | 5.74E-03 | 4.18E-02 |
| Dusp16 | 1115.41 | -0.86 | 0.23 | -3.82 | 1.35E-04 | 1.66E-03 |
| Cfl1 | 3471.58 | -0.86 | 0.28 | -3.14 | 1.69E-03 | 1.50E-02 |
| Nos3 | 2513.15 | -0.86 | 0.29 | -2.96 | 3.08E-03 | 2.51E-02 |
| Fnbp1l | 1137.20 | -0.86 | 0.21 | -4.20 | 2.63E-05 | 3.88E-04 |
| Nr4a3 | 6091.45 | -0.86 | 0.25 | -3.41 | 6.48E-04 | 6.61E-03 |
| Sh2b3 | 2343.60 | -0.87 | 0.25 | -3.46 | 5.34E-04 | 5.59E-03 |
| Nfya | 1093.00 | -0.87 | 0.18 | -4.74 | 2.19E-06 | 4.10E-05 |
| Nasp | 875.62 | -0.87 | 0.21 | -4.20 | 2.68E-05 | 3.95E-04 |
| Arf6 | 963.66 | -0.87 | 0.27 | -3.18 | 1.47E-03 | 1.33E-02 |
| Fam49a | 353.27 | -0.87 | 0.23 | -3.83 | 1.29E-04 | 1.60E-03 |
| Arl4d | 572.15 | -0.87 | 0.28 | -3.16 | 1.57E-03 | 1.41E-02 |
| Tasor2 | 644.98 | -0.87 | 0.24 | -3.61 | 3.04E-04 | 3.46E-03 |
| Dimt1 | 192.81 | -0.87 | 0.28 | -3.10 | 1.90E-03 | 1.66E-02 |
| Cdk17 | 835.99 | -0.87 | 0.19 | -4.63 | 3.68E-06 | 6.59E-05 |
| Nfatc1 | 833.97 | -0.88 | 0.26 | -3.32 | 8.93E-04 | 8.68E-03 |
| Cldn12 | 776.43 | -0.88 | 0.23 | -3.81 | 1.38E-04 | 1.70E-03 |
| Ubxn4 | 2274.20 | -0.88 | 0.23 | -3.79 | 1.51E-04 | 1.84E-03 |
| Mxd1 | 620.13 | -0.88 | 0.24 | -3.72 | 2.02E-04 | 2.37E-03 |
| Pnrc1 | 5076.17 | -0.88 | 0.19 | -4.57 | 4.79E-06 | 8.38E-05 |
| C3ar1 | 402.55 | -0.88 | 0.34 | -2.61 | 9.19E-03 | 6.05E-02 |
| Bmper | 195.89 | -0.88 | 0.35 | -2.48 | 1.33E-02 | 7.97E-02 |
| C1ra | 826.27 | -0.88 | 0.23 | -3.78 | 1.54E-04 | 1.88E-03 |
| Erf | 1239.92 | -0.88 | 0.22 | -3.94 | 8.21E-05 | 1.09E-03 |
| Spred2 | 945.06 | -0.88 | 0.23 | -3.86 | 1.14E-04 | 1.43E-03 |
| Stat3 | 8865.60 | -0.88 | 0.19 | -4.57 | 4.80E-06 | 8.40E-05 |
| P4ha1 | 1892.19 | -0.88 | 0.25 | -3.47 | 5.26E-04 | 5.54E-03 |
| Ms4a6b | 108.57 | -0.88 | 0.37 | -2.38 | 1.73E-02 | 9.74E-02 |
| Lmcd1 | 2344.09 | -0.88 | 0.26 | -3.43 | 5.96E-04 | 6.16E-03 |
| Lrrc8c | 1106.32 | -0.88 | 0.19 | -4.75 | 2.05E-06 | 3.87E-05 |
| Gm13340 | 622.37 | -0.88 | 0.21 | -4.30 | 1.73E-05 | 2.70E-04 |
| Pogk | 954.04 | -0.89 | 0.20 | -4.52 | 6.21E-06 | 1.07E-04 |
| C1qtnf6 | 163.44 | -0.89 | 0.33 | -2.73 | 6.37E-03 | 4.54E-02 |
| Sdad1 | 582.45 | -0.89 | 0.23 | -3.91 | 9.19E-05 | 1.20E-03 |
| Xiap | 2010.05 | -0.89 | 0.21 | -4.24 | 2.20E-05 | 3.32E-04 |
| Nhsl2 | 292.43 | -0.89 | 0.33 | -2.68 | 7.28E-03 | 5.06E-02 |
| Grwd1 | 342.11 | -0.89 | 0.26 | -3.45 | 5.66E-04 | 5.90E-03 |
| Sirt1 | 1361.82 | -0.89 | 0.21 | -4.27 | 1.99E-05 | 3.04E-04 |
| Gm13341 | 107.44 | -0.89 | 0.37 | -2.43 | 1.52E-02 | 8.82E-02 |
| Pi4k2b | 152.98 | -0.90 | 0.31 | -2.89 | 3.82E-03 | 3.01E-02 |
| Cmtm6 | 1025.96 | -0.90 | 0.26 | -3.46 | 5.40E-04 | 5.65E-03 |
| Tead2 | 162.13 | -0.90 | 0.31 | -2.86 | 4.20E-03 | 3.24E-02 |
| Creb1 | 994.08 | -0.90 | 0.20 | -4.50 | 6.81E-06 | 1.16E-04 |
| Ctdp1 | 567.37 | -0.90 | 0.31 | -2.93 | 3.36E-03 | 2.70E-02 |
| H2ax | 289.86 | -0.90 | 0.27 | -3.34 | 8.42E-04 | 8.26E-03 |
| Ltv1 | 586.96 | -0.90 | 0.22 | -4.07 | 4.63E-05 | 6.48E-04 |
| Noc4l | 357.78 | -0.91 | 0.24 | -3.85 | 1.19E-04 | 1.49E-03 |
| Pi15 | 180.31 | -0.91 | 0.35 | -2.57 | 1.00E-02 | 6.47E-02 |
| Gm28438 | 38187.61 | -0.91 | 0.30 | -3.06 | 2.25E-03 | 1.93E-02 |
| Mcm3 | 259.35 | -0.91 | 0.30 | -3.00 | 2.72E-03 | 2.26E-02 |
| Lyn | 633.32 | -0.91 | 0.20 | -4.60 | 4.18E-06 | 7.42E-05 |
| Nlgn2 | 542.97 | -0.91 | 0.27 | -3.33 | 8.70E-04 | 8.49E-03 |
| Tsr1 | 686.93 | -0.91 | 0.25 | -3.67 | 2.39E-04 | 2.77E-03 |
| Gpr132 | 162.29 | -0.91 | 0.32 | -2.89 | 3.89E-03 | 3.04E-02 |
| Dpep1 | 2015.12 | -0.91 | 0.24 | -3.75 | 1.74E-04 | 2.09E-03 |
| Bhlhe40 | 6864.96 | -0.91 | 0.27 | -3.44 | 5.91E-04 | 6.12E-03 |
| Aftph | 890.44 | -0.91 | 0.21 | -4.28 | 1.85E-05 | 2.86E-04 |
| Tgoln1 | 4531.90 | -0.91 | 0.21 | -4.32 | 1.55E-05 | 2.47E-04 |
| Abi1 | 1188.37 | -0.91 | 0.21 | -4.32 | 1.54E-05 | 2.45E-04 |
| Cbr2 | 592.22 | -0.91 | 0.38 | -2.43 | 1.53E-02 | 8.83E-02 |
| Nras | 1196.75 | -0.91 | 0.25 | -3.68 | 2.33E-04 | 2.70E-03 |
| Tank | 686.03 | -0.92 | 0.21 | -4.39 | 1.13E-05 | 1.84E-04 |
| Akna | 379.12 | -0.92 | 0.26 | -3.46 | 5.31E-04 | 5.57E-03 |
| Parp16 | 120.67 | -0.92 | 0.35 | -2.63 | 8.55E-03 | 5.71E-02 |
| Nfe2l2 | 3284.09 | -0.92 | 0.18 | -5.16 | 2.47E-07 | 5.47E-06 |
| Tlr4 | 420.43 | -0.92 | 0.24 | -3.91 | 9.38E-05 | 1.22E-03 |
| F2r | 1335.86 | -0.92 | 0.20 | -4.60 | 4.31E-06 | 7.64E-05 |
| mt-Co3 | 969778.42 | -0.92 | 0.18 | -5.22 | 1.82E-07 | 4.12E-06 |
| Pcna | 1639.23 | -0.92 | 0.24 | -3.90 | 9.68E-05 | 1.25E-03 |
| Fndc3a | 930.73 | -0.92 | 0.23 | -4.09 | 4.26E-05 | 6.01E-04 |
| Cdca4 | 195.25 | -0.92 | 0.31 | -2.99 | 2.81E-03 | 2.33E-02 |
| Flt4 | 766.38 | -0.92 | 0.22 | -4.27 | 1.94E-05 | 2.99E-04 |
| Serpinh1 | 8271.39 | -0.92 | 0.23 | -4.06 | 4.82E-05 | 6.74E-04 |
| Zfp800 | 339.70 | -0.92 | 0.24 | -3.93 | 8.58E-05 | 1.13E-03 |
| Slc11a2 | 1071.80 | -0.92 | 0.18 | -5.01 | 5.41E-07 | 1.13E-05 |
| Prkag3 | 95.23 | -0.92 | 0.38 | -2.42 | 1.57E-02 | 9.02E-02 |
| Ecscr | 315.45 | -0.93 | 0.31 | -2.98 | 2.86E-03 | 2.36E-02 |
| Usp16 | 1441.51 | -0.93 | 0.17 | -5.46 | 4.87E-08 | 1.16E-06 |
| Arhgap23 | 1495.62 | -0.93 | 0.31 | -3.01 | 2.62E-03 | 2.18E-02 |
| Sertad2 | 1293.68 | -0.93 | 0.22 | -4.29 | 1.79E-05 | 2.79E-04 |
| Nup62 | 753.93 | -0.93 | 0.25 | -3.71 | 2.06E-04 | 2.42E-03 |
| Rasa3 | 962.90 | -0.93 | 0.19 | -4.77 | 1.81E-06 | 3.43E-05 |
| Cd38 | 316.10 | -0.93 | 0.28 | -3.32 | 9.14E-04 | 8.86E-03 |
| Tent5a | 860.02 | -0.93 | 0.21 | -4.45 | 8.75E-06 | 1.46E-04 |
| Ccdc86 | 298.95 | -0.93 | 0.24 | -3.87 | 1.08E-04 | 1.37E-03 |
| Pgs1 | 840.38 | -0.93 | 0.22 | -4.25 | 2.16E-05 | 3.27E-04 |
| Csnk1d | 2661.19 | -0.93 | 0.20 | -4.68 | 2.83E-06 | 5.21E-05 |
| Sdc1 | 187.79 | -0.93 | 0.35 | -2.66 | 7.78E-03 | 5.30E-02 |
| Ccn2 | 8540.15 | -0.93 | 0.23 | -4.11 | 4.01E-05 | 5.68E-04 |
| Nudcd1 | 244.74 | -0.93 | 0.28 | -3.29 | 1.02E-03 | 9.73E-03 |
| Leo1 | 495.72 | -0.94 | 0.28 | -3.39 | 6.92E-04 | 6.98E-03 |
| Map1b | 432.02 | -0.94 | 0.29 | -3.22 | 1.27E-03 | 1.17E-02 |
| Casp8 | 260.44 | -0.94 | 0.30 | -3.13 | 1.72E-03 | 1.52E-02 |
| Tap2 | 1260.39 | -0.94 | 0.20 | -4.59 | 4.49E-06 | 7.92E-05 |
| Clcn5 | 140.89 | -0.94 | 0.35 | -2.69 | 7.20E-03 | 5.01E-02 |
| Klf10 | 1362.41 | -0.94 | 0.30 | -3.16 | 1.58E-03 | 1.41E-02 |
| Cmip | 492.55 | -0.94 | 0.26 | -3.65 | 2.65E-04 | 3.04E-03 |
| Gja4 | 194.06 | -0.94 | 0.33 | -2.86 | 4.21E-03 | 3.24E-02 |
| Csrp1 | 1778.49 | -0.94 | 0.20 | -4.60 | 4.13E-06 | 7.35E-05 |
| Sec23b | 604.16 | -0.94 | 0.23 | -4.16 | 3.18E-05 | 4.59E-04 |
| Bcar1 | 1655.10 | -0.94 | 0.22 | -4.30 | 1.74E-05 | 2.72E-04 |
| Ube2f | 994.24 | -0.94 | 0.21 | -4.58 | 4.57E-06 | 8.02E-05 |
| Eef1a1 | 27508.39 | -0.95 | 0.24 | -3.96 | 7.43E-05 | 9.89E-04 |
| Igf1 | 266.19 | -0.95 | 0.33 | -2.88 | 3.96E-03 | 3.09E-02 |
| Anxa7 | 3195.69 | -0.95 | 0.24 | -4.00 | 6.31E-05 | 8.53E-04 |
| Rtn4 | 2638.32 | -0.95 | 0.26 | -3.61 | 3.07E-04 | 3.49E-03 |
| Cntfr | 130.54 | -0.95 | 0.32 | -2.96 | 3.05E-03 | 2.49E-02 |
| Col4a2 | 12756.72 | -0.95 | 0.15 | -6.32 | 2.70E-10 | 8.45E-09 |
| Tulp1 | 292.75 | -0.95 | 0.27 | -3.49 | 4.77E-04 | 5.09E-03 |
| Acvrl1 | 1633.12 | -0.95 | 0.24 | -4.04 | 5.34E-05 | 7.39E-04 |
| Mex3c | 723.31 | -0.96 | 0.22 | -4.43 | 9.60E-06 | 1.59E-04 |
| Nop56 | 1277.99 | -0.96 | 0.26 | -3.67 | 2.40E-04 | 2.77E-03 |
| Efr3b | 639.55 | -0.96 | 0.25 | -3.90 | 9.68E-05 | 1.25E-03 |
| Socs2 | 1353.79 | -0.96 | 0.24 | -3.93 | 8.33E-05 | 1.10E-03 |
| Fbxo30 | 1465.44 | -0.96 | 0.17 | -5.49 | 3.95E-08 | 9.53E-07 |
| Mob3a | 417.15 | -0.96 | 0.29 | -3.34 | 8.32E-04 | 8.17E-03 |
| Dnajb4 | 6175.85 | -0.96 | 0.21 | -4.50 | 6.94E-06 | 1.18E-04 |
| Esyt1 | 1478.43 | -0.96 | 0.26 | -3.66 | 2.48E-04 | 2.86E-03 |
| Cstb | 324.27 | -0.96 | 0.32 | -2.99 | 2.76E-03 | 2.29E-02 |
| Plk3 | 1180.05 | -0.96 | 0.23 | -4.11 | 4.01E-05 | 5.68E-04 |
| Ecm1 | 1357.79 | -0.96 | 0.28 | -3.44 | 5.90E-04 | 6.11E-03 |
| Plekhg1 | 603.47 | -0.96 | 0.25 | -3.93 | 8.51E-05 | 1.12E-03 |
| Il1rap | 192.85 | -0.96 | 0.28 | -3.40 | 6.70E-04 | 6.80E-03 |
| Ppp1r9b | 2077.83 | -0.97 | 0.25 | -3.89 | 1.02E-04 | 1.30E-03 |
| Arpc5 | 1061.88 | -0.97 | 0.23 | -4.22 | 2.39E-05 | 3.58E-04 |
| Acer3 | 291.76 | -0.97 | 0.25 | -3.91 | 9.26E-05 | 1.20E-03 |
| Slc30a7 | 392.11 | -0.97 | 0.40 | -2.42 | 1.56E-02 | 8.96E-02 |
| Ccdc88b | 89.15 | -0.97 | 0.39 | -2.46 | 1.38E-02 | 8.19E-02 |
| Tcf21 | 416.03 | -0.97 | 0.34 | -2.81 | 4.91E-03 | 3.69E-02 |
| Fam241a | 93.82 | -0.97 | 0.41 | -2.40 | 1.64E-02 | 9.30E-02 |
| Nxf1 | 827.04 | -0.98 | 0.26 | -3.79 | 1.53E-04 | 1.86E-03 |
| Acot9 | 808.91 | -0.98 | 0.24 | -4.13 | 3.65E-05 | 5.20E-04 |
| H2-M3 | 138.67 | -0.98 | 0.34 | -2.84 | 4.45E-03 | 3.40E-02 |
| Rai14 | 251.20 | -0.98 | 0.26 | -3.75 | 1.74E-04 | 2.09E-03 |
| Col3a1 | 7042.77 | -0.98 | 0.24 | -4.15 | 3.30E-05 | 4.75E-04 |
| Rpl12 | 392.77 | -0.98 | 0.32 | -3.09 | 1.97E-03 | 1.71E-02 |
| C1s1 | 1419.64 | -0.99 | 0.22 | -4.55 | 5.43E-06 | 9.39E-05 |
| Enpp3 | 290.30 | -0.99 | 0.38 | -2.61 | 8.93E-03 | 5.92E-02 |
| Tinagl1 | 2470.78 | -0.99 | 0.25 | -3.99 | 6.71E-05 | 8.97E-04 |
| Eva1a | 124.43 | -0.99 | 0.41 | -2.44 | 1.45E-02 | 8.49E-02 |
| Tob1 | 2630.31 | -1.00 | 0.27 | -3.65 | 2.60E-04 | 2.99E-03 |
| Itgam | 328.85 | -1.00 | 0.31 | -3.25 | 1.15E-03 | 1.08E-02 |
| Nmi | 260.73 | -1.00 | 0.26 | -3.79 | 1.51E-04 | 1.85E-03 |
| Myh9 | 8056.40 | -1.00 | 0.15 | -6.61 | 3.80E-11 | 1.31E-09 |
| Ggct | 538.18 | -1.00 | 0.26 | -3.87 | 1.10E-04 | 1.39E-03 |
| Eif4a1 | 6368.61 | -1.00 | 0.18 | -5.60 | 2.18E-08 | 5.49E-07 |
| Man1a | 1753.15 | -1.00 | 0.21 | -4.65 | 3.27E-06 | 5.91E-05 |
| Tdrd7 | 660.26 | -1.00 | 0.26 | -3.83 | 1.26E-04 | 1.56E-03 |
| Iqgap1 | 3047.56 | -1.00 | 0.20 | -4.93 | 8.32E-07 | 1.66E-05 |
| Ift122 | 363.01 | -1.00 | 0.29 | -3.42 | 6.28E-04 | 6.44E-03 |
| Gtpbp4 | 1411.08 | -1.00 | 0.21 | -4.84 | 1.28E-06 | 2.49E-05 |
| Ackr3 | 3660.46 | -1.00 | 0.18 | -5.61 | 2.04E-08 | 5.17E-07 |
| Tlr7 | 153.11 | -1.00 | 0.40 | -2.49 | 1.29E-02 | 7.80E-02 |
| Tma16 | 355.62 | -1.01 | 0.24 | -4.13 | 3.65E-05 | 5.20E-04 |
| Top2a | 98.33 | -1.01 | 0.43 | -2.37 | 1.79E-02 | 1.00E-01 |
| Svep1 | 677.10 | -1.01 | 0.20 | -5.03 | 4.86E-07 | 1.02E-05 |
| Ywhaz | 4980.68 | -1.01 | 0.20 | -5.10 | 3.31E-07 | 7.17E-06 |
| Fem1b | 1136.32 | -1.01 | 0.22 | -4.54 | 5.64E-06 | 9.74E-05 |
| Pf4 | 449.45 | -1.01 | 0.34 | -2.95 | 3.16E-03 | 2.57E-02 |
| Nckap5l | 350.31 | -1.01 | 0.32 | -3.17 | 1.54E-03 | 1.39E-02 |
| Nup98 | 1904.51 | -1.01 | 0.18 | -5.55 | 2.89E-08 | 7.11E-07 |
| Taf1d | 517.02 | -1.01 | 0.25 | -4.01 | 5.96E-05 | 8.11E-04 |
| Tut7 | 2785.90 | -1.01 | 0.19 | -5.21 | 1.85E-07 | 4.17E-06 |
| Tyrobp | 158.55 | -1.01 | 0.42 | -2.44 | 1.47E-02 | 8.58E-02 |
| Tnip2 | 586.17 | -1.01 | 0.30 | -3.41 | 6.41E-04 | 6.55E-03 |
| Eef1e1 | 249.29 | -1.02 | 0.31 | -3.25 | 1.15E-03 | 1.08E-02 |
| Rabgef1 | 1131.90 | -1.02 | 0.20 | -5.00 | 5.86E-07 | 1.21E-05 |
| Clic1 | 1321.16 | -1.02 | 0.33 | -3.04 | 2.37E-03 | 2.01E-02 |
| Mak16 | 422.40 | -1.02 | 0.27 | -3.80 | 1.45E-04 | 1.77E-03 |
| Spon1 | 916.25 | -1.02 | 0.26 | -3.87 | 1.11E-04 | 1.40E-03 |
| Zfp703 | 1249.52 | -1.02 | 0.19 | -5.51 | 3.56E-08 | 8.62E-07 |
| Ubald1 | 2200.84 | -1.02 | 0.27 | -3.73 | 1.90E-04 | 2.25E-03 |
| Plekhg2 | 1520.35 | -1.03 | 0.23 | -4.40 | 1.07E-05 | 1.76E-04 |
| Ctss | 456.96 | -1.03 | 0.33 | -3.15 | 1.64E-03 | 1.46E-02 |
| Cpne8 | 427.76 | -1.03 | 0.27 | -3.87 | 1.07E-04 | 1.36E-03 |
| Ptgis | 543.12 | -1.03 | 0.34 | -2.99 | 2.79E-03 | 2.31E-02 |
| Nolc1 | 1074.20 | -1.03 | 0.23 | -4.41 | 1.01E-05 | 1.66E-04 |
| Col5a2 | 1483.08 | -1.03 | 0.31 | -3.35 | 8.01E-04 | 7.91E-03 |
| Zfp46 | 1911.16 | -1.03 | 0.19 | -5.29 | 1.21E-07 | 2.75E-06 |
| Mgat4a | 692.70 | -1.03 | 0.22 | -4.63 | 3.59E-06 | 6.46E-05 |
| Zfp960 | 222.58 | -1.03 | 0.39 | -2.63 | 8.60E-03 | 5.74E-02 |
| Rnf149 | 315.69 | -1.03 | 0.28 | -3.64 | 2.73E-04 | 3.13E-03 |
| Plxna2 | 2336.40 | -1.03 | 0.19 | -5.53 | 3.23E-08 | 7.90E-07 |
| Efhd2 | 1017.82 | -1.03 | 0.30 | -3.46 | 5.31E-04 | 5.57E-03 |
| Pabpc1 | 4384.76 | -1.03 | 0.23 | -4.52 | 6.04E-06 | 1.04E-04 |
| Slbp | 543.43 | -1.04 | 0.25 | -4.10 | 4.09E-05 | 5.78E-04 |
| Slc35e4 | 396.17 | -1.04 | 0.38 | -2.73 | 6.28E-03 | 4.49E-02 |
| Slc39a1 | 2947.23 | -1.04 | 0.23 | -4.59 | 4.52E-06 | 7.95E-05 |
| Aebp1 | 684.67 | -1.04 | 0.31 | -3.34 | 8.29E-04 | 8.15E-03 |
| Rassf4 | 702.80 | -1.04 | 0.25 | -4.14 | 3.43E-05 | 4.92E-04 |
| Rhob | 12210.91 | -1.04 | 0.21 | -4.95 | 7.29E-07 | 1.47E-05 |
| Mest | 149.33 | -1.04 | 0.41 | -2.53 | 1.13E-02 | 7.08E-02 |
| Ppm1j | 616.61 | -1.04 | 0.31 | -3.36 | 7.72E-04 | 7.67E-03 |
| Chd1 | 1274.23 | -1.04 | 0.18 | -5.66 | 1.51E-08 | 3.90E-07 |
| Trmt10a | 99.19 | -1.04 | 0.41 | -2.52 | 1.16E-02 | 7.17E-02 |
| Chrnb1 | 217.81 | -1.04 | 0.29 | -3.60 | 3.24E-04 | 3.65E-03 |
| Otud1 | 5415.98 | -1.05 | 0.26 | -3.96 | 7.37E-05 | 9.81E-04 |
| Ninl | 168.21 | -1.05 | 0.36 | -2.93 | 3.39E-03 | 2.72E-02 |
| Rhou | 274.25 | -1.05 | 0.29 | -3.67 | 2.41E-04 | 2.78E-03 |
| Bcl6 | 1512.78 | -1.05 | 0.19 | -5.66 | 1.50E-08 | 3.86E-07 |
| Itgb3 | 131.90 | -1.05 | 0.37 | -2.83 | 4.69E-03 | 3.55E-02 |
| Smad1 | 533.67 | -1.05 | 0.23 | -4.63 | 3.62E-06 | 6.49E-05 |
| Pros1 | 999.78 | -1.05 | 0.27 | -3.87 | 1.07E-04 | 1.36E-03 |
| D1Ertd622e | 204.75 | -1.05 | 0.30 | -3.51 | 4.54E-04 | 4.89E-03 |
| Thap2 | 264.68 | -1.05 | 0.31 | -3.43 | 6.04E-04 | 6.22E-03 |
| Rasl11b | 1677.22 | -1.05 | 0.23 | -4.48 | 7.39E-06 | 1.25E-04 |
| Eif4a-ps4 | 1279.68 | -1.05 | 0.26 | -4.06 | 4.83E-05 | 6.75E-04 |
| Lima1 | 1698.63 | -1.05 | 0.24 | -4.45 | 8.46E-06 | 1.42E-04 |
| Fam111a | 347.43 | -1.05 | 0.38 | -2.79 | 5.23E-03 | 3.89E-02 |
| Klf7 | 1845.63 | -1.06 | 0.18 | -5.94 | 2.91E-09 | 8.22E-08 |
| Capg | 400.41 | -1.06 | 0.41 | -2.59 | 9.59E-03 | 6.23E-02 |
| Slc2a3 | 127.91 | -1.06 | 0.41 | -2.58 | 9.99E-03 | 6.44E-02 |
| B3gnt3 | 345.20 | -1.06 | 0.25 | -4.24 | 2.22E-05 | 3.34E-04 |
| Cchcr1 | 233.14 | -1.06 | 0.40 | -2.62 | 8.76E-03 | 5.83E-02 |
| Adgrg1 | 2555.22 | -1.06 | 0.19 | -5.71 | 1.13E-08 | 2.97E-07 |
| Tlr3 | 315.61 | -1.06 | 0.35 | -3.03 | 2.45E-03 | 2.06E-02 |
| Actn1 | 1253.84 | -1.06 | 0.23 | -4.70 | 2.56E-06 | 4.76E-05 |
| Fam43a | 1544.79 | -1.06 | 0.27 | -3.89 | 9.91E-05 | 1.27E-03 |
| Cdr2 | 589.76 | -1.06 | 0.22 | -4.80 | 1.58E-06 | 3.04E-05 |
| Ptbp1 | 2111.64 | -1.06 | 0.21 | -5.07 | 3.94E-07 | 8.47E-06 |
| Agt | 166.09 | -1.06 | 0.33 | -3.18 | 1.46E-03 | 1.32E-02 |
| Dph5 | 166.49 | -1.07 | 0.38 | -2.81 | 4.97E-03 | 3.73E-02 |
| Rasa4 | 289.36 | -1.07 | 0.27 | -3.93 | 8.65E-05 | 1.14E-03 |
| Arl4a | 679.54 | -1.07 | 0.26 | -4.04 | 5.33E-05 | 7.39E-04 |
| Sav1 | 1023.45 | -1.07 | 0.18 | -5.99 | 2.15E-09 | 6.13E-08 |
| Ddit3 | 1396.85 | -1.07 | 0.29 | -3.69 | 2.23E-04 | 2.60E-03 |
| Yrdc | 529.21 | -1.07 | 0.25 | -4.21 | 2.56E-05 | 3.78E-04 |
| Tbc1d9 | 329.08 | -1.07 | 0.28 | -3.77 | 1.64E-04 | 1.98E-03 |
| Snhg11 | 483.87 | -1.07 | 0.31 | -3.50 | 4.65E-04 | 4.97E-03 |
| Arf4 | 2406.53 | -1.07 | 0.19 | -5.58 | 2.37E-08 | 5.94E-07 |
| Zcchc2 | 607.52 | -1.07 | 0.25 | -4.27 | 1.96E-05 | 3.01E-04 |
| Rnf31 | 1367.87 | -1.07 | 0.20 | -5.42 | 6.03E-08 | 1.41E-06 |
| Lilrb4a | 1607.96 | -1.07 | 0.24 | -4.40 | 1.06E-05 | 1.74E-04 |
| Pdk4 | 31593.28 | -1.08 | 0.21 | -5.05 | 4.38E-07 | 9.31E-06 |
| Ccl21b | 179.57 | -1.08 | 0.43 | -2.53 | 1.15E-02 | 7.14E-02 |
| Slc20a1 | 1867.37 | -1.08 | 0.25 | -4.31 | 1.66E-05 | 2.62E-04 |
| Uck2 | 1382.30 | -1.08 | 0.29 | -3.68 | 2.33E-04 | 2.71E-03 |
| Hmgcr | 484.30 | -1.08 | 0.21 | -5.05 | 4.48E-07 | 9.49E-06 |
| Foxc1 | 263.97 | -1.08 | 0.32 | -3.41 | 6.60E-04 | 6.72E-03 |
| Gstcd | 79.47 | -1.09 | 0.40 | -2.71 | 6.77E-03 | 4.76E-02 |
| Btg2 | 15821.00 | -1.09 | 0.29 | -3.74 | 1.81E-04 | 2.16E-03 |
| Acsl5 | 687.09 | -1.09 | 0.24 | -4.46 | 8.14E-06 | 1.37E-04 |
| Cpxm2 | 388.57 | -1.09 | 0.32 | -3.44 | 5.90E-04 | 6.11E-03 |
| Sox7 | 1661.40 | -1.09 | 0.20 | -5.47 | 4.49E-08 | 1.08E-06 |
| C1qb | 1293.01 | -1.09 | 0.27 | -3.99 | 6.64E-05 | 8.90E-04 |
| Cadm3 | 130.25 | -1.09 | 0.39 | -2.82 | 4.79E-03 | 3.62E-02 |
| Nle1 | 404.21 | -1.10 | 0.29 | -3.74 | 1.85E-04 | 2.19E-03 |
| Ptpn6 | 152.37 | -1.10 | 0.35 | -3.10 | 1.93E-03 | 1.68E-02 |
| Jund | 13029.05 | -1.10 | 0.21 | -5.36 | 8.43E-08 | 1.95E-06 |
| Mst1r | 133.58 | -1.10 | 0.38 | -2.88 | 3.94E-03 | 3.07E-02 |
| Zfp503 | 136.09 | -1.11 | 0.39 | -2.84 | 4.44E-03 | 3.40E-02 |
| Slc25a30 | 415.44 | -1.11 | 0.26 | -4.27 | 1.92E-05 | 2.96E-04 |
| Elf4 | 631.08 | -1.11 | 0.25 | -4.36 | 1.29E-05 | 2.08E-04 |
| Hivep2 | 1654.22 | -1.11 | 0.19 | -5.80 | 6.74E-09 | 1.81E-07 |
| Trpv4 | 364.50 | -1.11 | 0.26 | -4.28 | 1.88E-05 | 2.91E-04 |
| Dclk2 | 70.42 | -1.11 | 0.47 | -2.38 | 1.71E-02 | 9.64E-02 |
| Rrm2 | 63.63 | -1.11 | 0.46 | -2.40 | 1.65E-02 | 9.35E-02 |
| Prr13 | 761.02 | -1.11 | 0.26 | -4.31 | 1.63E-05 | 2.58E-04 |
| Slc1a5 | 1189.25 | -1.12 | 0.32 | -3.48 | 5.00E-04 | 5.30E-03 |
| Klf2 | 5274.35 | -1.12 | 0.33 | -3.38 | 7.30E-04 | 7.32E-03 |
| Dusp10 | 592.53 | -1.12 | 0.22 | -5.08 | 3.82E-07 | 8.23E-06 |
| Jmjd1c | 2251.49 | -1.12 | 0.27 | -4.10 | 4.17E-05 | 5.89E-04 |
| Ppp1r15b | 1655.27 | -1.12 | 0.20 | -5.67 | 1.39E-08 | 3.61E-07 |
| Slc4a8 | 153.62 | -1.12 | 0.45 | -2.49 | 1.28E-02 | 7.74E-02 |
| Ptpn23 | 1670.08 | -1.12 | 0.23 | -4.84 | 1.32E-06 | 2.57E-05 |
| Tapbpl | 254.79 | -1.12 | 0.25 | -4.47 | 7.81E-06 | 1.32E-04 |
| Syt12 | 139.56 | -1.12 | 0.41 | -2.72 | 6.51E-03 | 4.62E-02 |
| E2f5 | 136.10 | -1.12 | 0.34 | -3.27 | 1.06E-03 | 1.01E-02 |
| Hectd2 | 134.29 | -1.12 | 0.37 | -3.07 | 2.17E-03 | 1.87E-02 |
| Sla | 54.31 | -1.12 | 0.47 | -2.41 | 1.57E-02 | 9.02E-02 |
| Ms4a7 | 115.99 | -1.13 | 0.44 | -2.58 | 9.84E-03 | 6.37E-02 |
| Trim26 | 719.04 | -1.13 | 0.26 | -4.26 | 2.09E-05 | 3.16E-04 |
| Nfkbib | 916.05 | -1.13 | 0.23 | -4.82 | 1.47E-06 | 2.84E-05 |
| Topors | 602.82 | -1.13 | 0.24 | -4.74 | 2.12E-06 | 3.99E-05 |
| Fhl3 | 355.38 | -1.13 | 0.26 | -4.32 | 1.58E-05 | 2.51E-04 |
| Fbn1 | 2838.19 | -1.13 | 0.19 | -5.80 | 6.46E-09 | 1.73E-07 |
| Arrdc2 | 506.43 | -1.13 | 0.30 | -3.78 | 1.54E-04 | 1.87E-03 |
| Postn | 1424.61 | -1.13 | 0.27 | -4.18 | 2.87E-05 | 4.19E-04 |
| Clec5a | 57.50 | -1.13 | 0.45 | -2.49 | 1.26E-02 | 7.70E-02 |
| Actb | 17515.21 | -1.13 | 0.20 | -5.61 | 2.04E-08 | 5.17E-07 |
| Ddx21 | 2407.88 | -1.14 | 0.19 | -5.85 | 4.80E-09 | 1.30E-07 |
| Gck | 393.77 | -1.14 | 0.26 | -4.38 | 1.17E-05 | 1.89E-04 |
| Arpc1b | 1813.43 | -1.14 | 0.27 | -4.27 | 1.93E-05 | 2.97E-04 |
| Ehd1 | 4748.64 | -1.14 | 0.19 | -5.86 | 4.63E-09 | 1.26E-07 |
| Tagln2 | 3442.22 | -1.14 | 0.22 | -5.15 | 2.65E-07 | 5.83E-06 |
| Prkcd | 725.29 | -1.14 | 0.26 | -4.40 | 1.10E-05 | 1.81E-04 |
| Atp10a | 123.49 | -1.14 | 0.35 | -3.23 | 1.22E-03 | 1.14E-02 |
| Metrnl | 686.98 | -1.14 | 0.32 | -3.58 | 3.49E-04 | 3.89E-03 |
| Clmp | 202.28 | -1.15 | 0.34 | -3.42 | 6.31E-04 | 6.45E-03 |
| Gngt2 | 328.21 | -1.15 | 0.46 | -2.48 | 1.31E-02 | 7.87E-02 |
| Foxc2 | 112.34 | -1.15 | 0.36 | -3.23 | 1.25E-03 | 1.16E-02 |
| Fmnl2 | 478.25 | -1.15 | 0.27 | -4.18 | 2.94E-05 | 4.29E-04 |
| Tsc22d2 | 1787.31 | -1.15 | 0.25 | -4.65 | 3.34E-06 | 6.01E-05 |
| P2ry6 | 91.89 | -1.15 | 0.45 | -2.54 | 1.12E-02 | 7.03E-02 |
| Stk40 | 2850.47 | -1.15 | 0.21 | -5.60 | 2.18E-08 | 5.50E-07 |
| Ccn5 | 72.24 | -1.15 | 0.42 | -2.75 | 6.04E-03 | 4.35E-02 |
| Coq8b | 310.68 | -1.15 | 0.26 | -4.36 | 1.31E-05 | 2.11E-04 |
| Msx1 | 253.90 | -1.16 | 0.32 | -3.68 | 2.37E-04 | 2.75E-03 |
| Dok2 | 101.34 | -1.16 | 0.48 | -2.42 | 1.55E-02 | 8.92E-02 |
| Fxyd5 | 412.32 | -1.16 | 0.34 | -3.45 | 5.64E-04 | 5.88E-03 |
| Pik3r6 | 259.67 | -1.16 | 0.28 | -4.11 | 3.94E-05 | 5.60E-04 |
| Malt1 | 80.39 | -1.16 | 0.48 | -2.41 | 1.61E-02 | 9.19E-02 |
| Mafb | 840.02 | -1.16 | 0.27 | -4.37 | 1.27E-05 | 2.04E-04 |
| Eif5 | 6223.78 | -1.17 | 0.17 | -6.84 | 7.81E-12 | 2.82E-10 |
| Klhl40 | 1731.09 | -1.17 | 0.33 | -3.58 | 3.44E-04 | 3.85E-03 |
| Casp3 | 158.72 | -1.17 | 0.35 | -3.29 | 1.00E-03 | 9.65E-03 |
| Tcirg1 | 579.89 | -1.17 | 0.21 | -5.59 | 2.30E-08 | 5.78E-07 |
| Fcna | 132.83 | -1.17 | 0.44 | -2.64 | 8.35E-03 | 5.62E-02 |
| Trim16 | 406.34 | -1.17 | 0.32 | -3.71 | 2.08E-04 | 2.44E-03 |
| Zfand2a | 937.43 | -1.17 | 0.26 | -4.51 | 6.45E-06 | 1.11E-04 |
| Timd4 | 133.28 | -1.17 | 0.32 | -3.61 | 3.08E-04 | 3.49E-03 |
| Mmp19 | 154.02 | -1.17 | 0.34 | -3.41 | 6.58E-04 | 6.70E-03 |
| Fam107b | 430.50 | -1.17 | 0.33 | -3.59 | 3.33E-04 | 3.74E-03 |
| Ptbp3 | 1389.85 | -1.17 | 0.19 | -6.08 | 1.23E-09 | 3.60E-08 |
| Resf1 | 792.55 | -1.17 | 0.27 | -4.32 | 1.54E-05 | 2.45E-04 |
| Loxl2 | 1172.79 | -1.17 | 0.23 | -5.21 | 1.90E-07 | 4.28E-06 |
| Prdm1 | 217.50 | -1.17 | 0.32 | -3.63 | 2.88E-04 | 3.29E-03 |
| Man2a1 | 1574.71 | -1.17 | 0.19 | -6.27 | 3.72E-10 | 1.14E-08 |
| Irf2bp2 | 2511.52 | -1.17 | 0.24 | -5.00 | 5.80E-07 | 1.20E-05 |
| Tmsb10 | 1020.93 | -1.18 | 0.25 | -4.68 | 2.84E-06 | 5.23E-05 |
| Ier5l | 639.94 | -1.18 | 0.30 | -3.89 | 9.95E-05 | 1.27E-03 |
| Zfp281 | 575.78 | -1.18 | 0.21 | -5.63 | 1.78E-08 | 4.53E-07 |
| Sde2 | 1117.52 | -1.18 | 0.27 | -4.42 | 9.91E-06 | 1.63E-04 |
| Fam107a | 250.99 | -1.18 | 0.32 | -3.74 | 1.84E-04 | 2.19E-03 |
| Ddx60 | 150.27 | -1.18 | 0.40 | -2.98 | 2.84E-03 | 2.35E-02 |
| Spry2 | 1010.18 | -1.18 | 0.30 | -3.91 | 9.06E-05 | 1.18E-03 |
| Btg1 | 3009.01 | -1.19 | 0.26 | -4.51 | 6.61E-06 | 1.13E-04 |
| Poldip3 | 116.12 | -1.19 | 0.45 | -2.65 | 8.05E-03 | 5.45E-02 |
| Setdb2 | 174.29 | -1.19 | 0.37 | -3.22 | 1.28E-03 | 1.18E-02 |
| 1810055G02Rik | 280.53 | -1.19 | 0.25 | -4.66 | 3.16E-06 | 5.75E-05 |
| Icosl | 886.02 | -1.19 | 0.25 | -4.72 | 2.39E-06 | 4.47E-05 |
| Rgs2 | 1797.31 | -1.19 | 0.24 | -4.86 | 1.20E-06 | 2.34E-05 |
| Itgbl1 | 251.02 | -1.19 | 0.34 | -3.55 | 3.88E-04 | 4.30E-03 |
| Alkbh1 | 237.71 | -1.19 | 0.36 | -3.29 | 9.95E-04 | 9.58E-03 |
| Cyb561 | 518.66 | -1.20 | 0.30 | -3.92 | 8.77E-05 | 1.15E-03 |
| Gadd45a | 1558.19 | -1.20 | 0.28 | -4.32 | 1.59E-05 | 2.52E-04 |
| Fstl1 | 3505.55 | -1.20 | 0.21 | -5.71 | 1.13E-08 | 2.98E-07 |
| Pak4 | 297.27 | -1.20 | 0.27 | -4.37 | 1.23E-05 | 1.99E-04 |
| Il3ra | 158.67 | -1.20 | 0.33 | -3.61 | 3.06E-04 | 3.48E-03 |
| Cd24a | 96.35 | -1.20 | 0.39 | -3.09 | 1.99E-03 | 1.73E-02 |
| Gm48216 | 79.19 | -1.20 | 0.44 | -2.73 | 6.29E-03 | 4.49E-02 |
| Abi3 | 421.49 | -1.21 | 0.35 | -3.46 | 5.32E-04 | 5.58E-03 |
| Ppard | 87.04 | -1.21 | 0.46 | -2.60 | 9.25E-03 | 6.08E-02 |
| Heatr1 | 507.36 | -1.21 | 0.26 | -4.69 | 2.71E-06 | 5.01E-05 |
| Swap70 | 1316.04 | -1.21 | 0.25 | -4.85 | 1.24E-06 | 2.43E-05 |
| Mafk | 4353.99 | -1.21 | 0.22 | -5.41 | 6.46E-08 | 1.50E-06 |
| Map2k3 | 3207.41 | -1.21 | 0.22 | -5.47 | 4.60E-08 | 1.10E-06 |
| Ctps | 1635.14 | -1.21 | 0.24 | -5.07 | 4.07E-07 | 8.72E-06 |
| Gas2l3 | 90.26 | -1.21 | 0.50 | -2.44 | 1.49E-02 | 8.67E-02 |
| AW822252 | 333.56 | -1.21 | 0.38 | -3.23 | 1.24E-03 | 1.15E-02 |
| Rhog | 726.31 | -1.21 | 0.26 | -4.59 | 4.49E-06 | 7.92E-05 |
| Tle3 | 811.85 | -1.22 | 0.20 | -5.93 | 3.00E-09 | 8.43E-08 |
| S100a11 | 709.65 | -1.22 | 0.26 | -4.70 | 2.61E-06 | 4.85E-05 |
| Il1rl2 | 170.60 | -1.22 | 0.44 | -2.76 | 5.70E-03 | 4.15E-02 |
| Apold1 | 4954.27 | -1.22 | 0.27 | -4.47 | 7.85E-06 | 1.32E-04 |
| Cfp | 398.57 | -1.22 | 0.34 | -3.64 | 2.76E-04 | 3.16E-03 |
| Slc4a7 | 271.13 | -1.22 | 0.28 | -4.42 | 9.75E-06 | 1.61E-04 |
| Hes1 | 1734.64 | -1.22 | 0.27 | -4.60 | 4.26E-06 | 7.56E-05 |
| Gcnt2 | 389.24 | -1.22 | 0.37 | -3.33 | 8.56E-04 | 8.37E-03 |
| Cbx4 | 735.76 | -1.22 | 0.23 | -5.43 | 5.66E-08 | 1.33E-06 |
| Mcm4 | 334.57 | -1.22 | 0.32 | -3.80 | 1.45E-04 | 1.78E-03 |
| Clec4a1 | 102.39 | -1.22 | 0.46 | -2.68 | 7.38E-03 | 5.11E-02 |
| Stk19 | 375.65 | -1.22 | 0.27 | -4.55 | 5.29E-06 | 9.17E-05 |
| Cotl1 | 375.09 | -1.22 | 0.32 | -3.79 | 1.53E-04 | 1.86E-03 |
| Cachd1 | 215.07 | -1.23 | 0.29 | -4.20 | 2.69E-05 | 3.96E-04 |
| Prkd2 | 1974.59 | -1.23 | 0.18 | -6.85 | 7.40E-12 | 2.69E-10 |
| Ltbp2 | 129.60 | -1.23 | 0.50 | -2.45 | 1.44E-02 | 8.44E-02 |
| Skap2 | 338.68 | -1.23 | 0.30 | -4.07 | 4.61E-05 | 6.46E-04 |
| Lgi1 | 121.90 | -1.23 | 0.35 | -3.52 | 4.37E-04 | 4.74E-03 |
| Birc2 | 965.55 | -1.23 | 0.18 | -6.77 | 1.33E-11 | 4.74E-10 |
| Tnfrsf1a | 1692.28 | -1.23 | 0.25 | -4.97 | 6.84E-07 | 1.39E-05 |
| Zfp429 | 71.06 | -1.23 | 0.47 | -2.61 | 8.93E-03 | 5.92E-02 |
| Map3k8 | 115.71 | -1.24 | 0.38 | -3.23 | 1.24E-03 | 1.15E-02 |
| Adar | 1003.21 | -1.24 | 0.20 | -6.29 | 3.12E-10 | 9.71E-09 |
| Ankrd33b | 2020.15 | -1.24 | 0.17 | -7.11 | 1.15E-12 | 4.48E-11 |
| Tfrc | 2008.65 | -1.24 | 0.29 | -4.34 | 1.42E-05 | 2.27E-04 |
| Sgk1 | 3244.98 | -1.24 | 0.27 | -4.63 | 3.60E-06 | 6.48E-05 |
| Cd68 | 267.41 | -1.24 | 0.41 | -3.02 | 2.56E-03 | 2.14E-02 |
| Actg1 | 25747.85 | -1.25 | 0.24 | -5.10 | 3.35E-07 | 7.24E-06 |
| Tcf7l2 | 121.98 | -1.25 | 0.43 | -2.91 | 3.63E-03 | 2.88E-02 |
| Coro1a | 269.03 | -1.25 | 0.35 | -3.56 | 3.67E-04 | 4.07E-03 |
| Thbd | 3591.48 | -1.25 | 0.33 | -3.75 | 1.76E-04 | 2.11E-03 |
| Mt1 | 7300.53 | -1.25 | 0.19 | -6.62 | 3.67E-11 | 1.27E-09 |
| Fbxo33 | 487.95 | -1.25 | 0.25 | -4.96 | 6.93E-07 | 1.41E-05 |
| Aoc2 | 116.09 | -1.25 | 0.39 | -3.23 | 1.25E-03 | 1.16E-02 |
| Cd74 | 1721.75 | -1.25 | 0.21 | -6.01 | 1.81E-09 | 5.19E-08 |
| Slc11a1 | 180.06 | -1.25 | 0.43 | -2.92 | 3.47E-03 | 2.77E-02 |
| Itga5 | 4678.44 | -1.26 | 0.25 | -5.03 | 4.99E-07 | 1.04E-05 |
| Klhl25 | 358.37 | -1.26 | 0.27 | -4.67 | 3.02E-06 | 5.51E-05 |
| Efemp1 | 329.10 | -1.26 | 0.27 | -4.66 | 3.17E-06 | 5.76E-05 |
| Gpnmb | 89.35 | -1.26 | 0.51 | -2.49 | 1.28E-02 | 7.74E-02 |
| Trim56 | 1662.03 | -1.26 | 0.23 | -5.61 | 1.98E-08 | 5.04E-07 |
| Clec2d | 630.62 | -1.27 | 0.23 | -5.46 | 4.86E-08 | 1.15E-06 |
| Spata5 | 256.80 | -1.27 | 0.30 | -4.26 | 2.04E-05 | 3.11E-04 |
| Ube2l6 | 230.46 | -1.27 | 0.34 | -3.77 | 1.64E-04 | 1.98E-03 |
| Clp1 | 138.26 | -1.27 | 0.34 | -3.72 | 2.01E-04 | 2.37E-03 |
| N4bp1 | 1450.49 | -1.27 | 0.18 | -7.24 | 4.52E-13 | 1.83E-11 |
| Ccn1 | 14793.66 | -1.27 | 0.23 | -5.42 | 5.99E-08 | 1.41E-06 |
| Zc3h12c | 376.35 | -1.27 | 0.26 | -4.84 | 1.28E-06 | 2.49E-05 |
| 3110082I17Rik | 186.97 | -1.27 | 0.48 | -2.63 | 8.53E-03 | 5.70E-02 |
| Prnd | 84.04 | -1.28 | 0.53 | -2.40 | 1.63E-02 | 9.29E-02 |
| Magt1 | 307.02 | -1.28 | 0.35 | -3.62 | 3.00E-04 | 3.42E-03 |
| Pdlim7 | 2036.69 | -1.28 | 0.24 | -5.39 | 7.06E-08 | 1.64E-06 |
| Trps1 | 290.58 | -1.28 | 0.34 | -3.80 | 1.42E-04 | 1.74E-03 |
| Snai1 | 63.16 | -1.28 | 0.47 | -2.72 | 6.56E-03 | 4.65E-02 |
| Col27a1 | 495.97 | -1.28 | 0.29 | -4.42 | 9.69E-06 | 1.60E-04 |
| Pde1b | 112.48 | -1.28 | 0.36 | -3.56 | 3.77E-04 | 4.18E-03 |
| Sdcbp | 3783.31 | -1.28 | 0.21 | -6.19 | 6.20E-10 | 1.87E-08 |
| Parp12 | 954.80 | -1.28 | 0.27 | -4.71 | 2.46E-06 | 4.58E-05 |
| Dcun1d3 | 500.49 | -1.29 | 0.29 | -4.39 | 1.14E-05 | 1.86E-04 |
| Stx6 | 726.17 | -1.29 | 0.23 | -5.57 | 2.57E-08 | 6.37E-07 |
| Elf1 | 1458.33 | -1.29 | 0.23 | -5.52 | 3.36E-08 | 8.19E-07 |
| Trim12a | 385.23 | -1.29 | 0.24 | -5.42 | 5.98E-08 | 1.41E-06 |
| Fmod | 87.03 | -1.29 | 0.47 | -2.76 | 5.69E-03 | 4.15E-02 |
| Col4a1 | 17754.72 | -1.29 | 0.18 | -7.16 | 8.00E-13 | 3.15E-11 |
| Tgm2 | 10332.11 | -1.29 | 0.17 | -7.42 | 1.17E-13 | 4.93E-12 |
| Ednrb | 1317.82 | -1.29 | 0.26 | -5.03 | 4.99E-07 | 1.04E-05 |
| Hspb1 | 17334.98 | -1.29 | 0.24 | -5.42 | 6.07E-08 | 1.42E-06 |
| Rfx5 | 318.38 | -1.29 | 0.26 | -5.01 | 5.54E-07 | 1.15E-05 |
| Dram1 | 295.21 | -1.29 | 0.26 | -5.07 | 3.97E-07 | 8.52E-06 |
| Il27ra | 103.15 | -1.30 | 0.38 | -3.37 | 7.49E-04 | 7.47E-03 |
| Slc7a6 | 502.19 | -1.30 | 0.22 | -5.78 | 7.29E-09 | 1.95E-07 |
| Fam110a | 89.72 | -1.30 | 0.41 | -3.14 | 1.69E-03 | 1.50E-02 |
| Hmcn2 | 1617.79 | -1.30 | 0.25 | -5.15 | 2.65E-07 | 5.83E-06 |
| Rlim | 1423.53 | -1.30 | 0.19 | -6.75 | 1.45E-11 | 5.14E-10 |
| Zswim4 | 1305.86 | -1.30 | 0.20 | -6.48 | 9.11E-11 | 3.00E-09 |
| Gm29216 | 203898.04 | -1.30 | 0.21 | -6.34 | 2.25E-10 | 7.14E-09 |
| Ampd2 | 416.47 | -1.31 | 0.24 | -5.47 | 4.52E-08 | 1.08E-06 |
| Foxm1 | 48.96 | -1.31 | 0.51 | -2.57 | 1.02E-02 | 6.55E-02 |
| Lag3 | 79.67 | -1.31 | 0.51 | -2.58 | 9.83E-03 | 6.37E-02 |
| Pdgfa | 1224.43 | -1.31 | 0.19 | -6.86 | 6.67E-12 | 2.44E-10 |
| Zbtb21 | 261.92 | -1.31 | 0.29 | -4.56 | 5.02E-06 | 8.73E-05 |
| Papss2 | 799.88 | -1.31 | 0.20 | -6.72 | 1.84E-11 | 6.41E-10 |
| Rad52 | 259.67 | -1.31 | 0.29 | -4.48 | 7.33E-06 | 1.24E-04 |
| Hspa1l | 240.87 | -1.32 | 0.27 | -4.79 | 1.64E-06 | 3.14E-05 |
| Fam129b | 1825.14 | -1.32 | 0.33 | -4.04 | 5.38E-05 | 7.43E-04 |
| Dcp2 | 669.33 | -1.32 | 0.29 | -4.46 | 8.01E-06 | 1.35E-04 |
| Marchf3 | 210.22 | -1.32 | 0.28 | -4.69 | 2.68E-06 | 4.98E-05 |
| Ptpn12 | 1984.82 | -1.32 | 0.19 | -6.98 | 2.96E-12 | 1.11E-10 |
| Rrp15 | 213.68 | -1.32 | 0.31 | -4.23 | 2.35E-05 | 3.53E-04 |
| Gli1 | 91.11 | -1.32 | 0.43 | -3.09 | 1.97E-03 | 1.71E-02 |
| Clic4 | 20749.20 | -1.32 | 0.16 | -8.28 | 1.20E-16 | 6.40E-15 |
| Bcl2l11 | 1132.24 | -1.32 | 0.18 | -7.36 | 1.85E-13 | 7.65E-12 |
| Adamts14 | 89.11 | -1.32 | 0.40 | -3.26 | 1.10E-03 | 1.04E-02 |
| Cd9 | 1184.48 | -1.32 | 0.29 | -4.50 | 6.92E-06 | 1.18E-04 |
| Gsap | 142.89 | -1.32 | 0.32 | -4.16 | 3.16E-05 | 4.56E-04 |
| Flvcr1 | 453.24 | -1.32 | 0.24 | -5.58 | 2.45E-08 | 6.10E-07 |
| Atp2b4 | 1382.41 | -1.33 | 0.24 | -5.52 | 3.45E-08 | 8.38E-07 |
| Ogfr | 1608.12 | -1.33 | 0.20 | -6.72 | 1.82E-11 | 6.34E-10 |
| Rin3 | 770.76 | -1.33 | 0.25 | -5.33 | 9.81E-08 | 2.25E-06 |
| Lpar1 | 459.75 | -1.33 | 0.23 | -5.81 | 6.18E-09 | 1.66E-07 |
| Slc3a2 | 1959.34 | -1.33 | 0.28 | -4.69 | 2.72E-06 | 5.03E-05 |
| S1pr3 | 962.34 | -1.33 | 0.31 | -4.22 | 2.39E-05 | 3.58E-04 |
| Hs3st3b1 | 57.96 | -1.33 | 0.49 | -2.74 | 6.15E-03 | 4.42E-02 |
| Mpp2 | 441.62 | -1.33 | 0.25 | -5.44 | 5.43E-08 | 1.28E-06 |
| Dbn1 | 390.07 | -1.34 | 0.27 | -4.87 | 1.09E-06 | 2.14E-05 |
| Ppan | 361.70 | -1.34 | 0.35 | -3.88 | 1.05E-04 | 1.33E-03 |
| Tubb4b | 10209.69 | -1.34 | 0.18 | -7.47 | 8.24E-14 | 3.56E-12 |
| Dennd3 | 796.06 | -1.34 | 0.30 | -4.50 | 6.94E-06 | 1.18E-04 |
| Slc66a2 | 1104.35 | -1.34 | 0.27 | -5.05 | 4.31E-07 | 9.18E-06 |
| Mrc1 | 1482.46 | -1.35 | 0.19 | -7.06 | 1.64E-12 | 6.27E-11 |
| Slc7a7 | 131.74 | -1.35 | 0.39 | -3.44 | 5.77E-04 | 6.01E-03 |
| Jun | 18954.62 | -1.35 | 0.23 | -5.92 | 3.32E-09 | 9.27E-08 |
| Dyrk2 | 2220.05 | -1.35 | 0.21 | -6.44 | 1.23E-10 | 4.01E-09 |
| 4930523C07Rik | 936.69 | -1.35 | 0.21 | -6.55 | 5.57E-11 | 1.88E-09 |
| Sh3bp2 | 177.85 | -1.35 | 0.35 | -3.91 | 9.36E-05 | 1.21E-03 |
| Plk2 | 3278.14 | -1.36 | 0.17 | -7.92 | 2.34E-15 | 1.14E-13 |
| Pgm2 | 504.77 | -1.36 | 0.32 | -4.30 | 1.71E-05 | 2.68E-04 |
| Kcnd1 | 41.12 | -1.36 | 0.56 | -2.42 | 1.57E-02 | 9.01E-02 |
| St3gal1 | 1733.66 | -1.36 | 0.22 | -6.31 | 2.81E-10 | 8.78E-09 |
| Tcim | 2681.79 | -1.36 | 0.25 | -5.34 | 9.06E-08 | 2.08E-06 |
| Elmsan1 | 1811.12 | -1.37 | 0.24 | -5.65 | 1.61E-08 | 4.14E-07 |
| Vcan | 781.58 | -1.37 | 0.28 | -4.92 | 8.59E-07 | 1.71E-05 |
| Ptpn1 | 1053.25 | -1.37 | 0.20 | -6.85 | 7.55E-12 | 2.74E-10 |
| Tubb4a | 155.32 | -1.37 | 0.38 | -3.58 | 3.45E-04 | 3.86E-03 |
| Tnfrsf23 | 115.60 | -1.37 | 0.40 | -3.44 | 5.90E-04 | 6.11E-03 |
| Paqr8 | 116.55 | -1.37 | 0.41 | -3.34 | 8.48E-04 | 8.30E-03 |
| S100a10 | 1618.53 | -1.37 | 0.28 | -4.99 | 6.02E-07 | 1.24E-05 |
| Ralb | 1205.25 | -1.37 | 0.19 | -7.34 | 2.12E-13 | 8.75E-12 |
| Hspa2 | 460.48 | -1.37 | 0.31 | -4.49 | 7.23E-06 | 1.23E-04 |
| Siah2 | 546.49 | -1.37 | 0.24 | -5.68 | 1.36E-08 | 3.55E-07 |
| Neto2 | 67.55 | -1.38 | 0.44 | -3.12 | 1.80E-03 | 1.58E-02 |
| Psat1 | 95.30 | -1.38 | 0.45 | -3.05 | 2.32E-03 | 1.98E-02 |
| Hdc | 122.85 | -1.38 | 0.41 | -3.35 | 8.03E-04 | 7.92E-03 |
| Peli1 | 1896.23 | -1.38 | 0.19 | -7.19 | 6.59E-13 | 2.63E-11 |
| Slc16a13 | 622.45 | -1.38 | 0.27 | -5.06 | 4.18E-07 | 8.92E-06 |
| Nrp2 | 2232.31 | -1.38 | 0.21 | -6.73 | 1.69E-11 | 5.93E-10 |
| Thsd7a | 279.34 | -1.38 | 0.42 | -3.33 | 8.54E-04 | 8.36E-03 |
| B3gnt2 | 530.32 | -1.38 | 0.24 | -5.67 | 1.41E-08 | 3.66E-07 |
| Tnfsf10 | 426.18 | -1.39 | 0.42 | -3.27 | 1.07E-03 | 1.01E-02 |
| Gm10160 | 183.18 | -1.39 | 0.41 | -3.37 | 7.49E-04 | 7.47E-03 |
| Rhoj | 1749.44 | -1.39 | 0.27 | -5.21 | 1.88E-07 | 4.23E-06 |
| Klf6 | 7737.38 | -1.39 | 0.17 | -8.22 | 1.99E-16 | 1.05E-14 |
| Avpr1a | 48.19 | -1.39 | 0.52 | -2.66 | 7.74E-03 | 5.29E-02 |
| Smad7 | 1291.97 | -1.39 | 0.25 | -5.67 | 1.41E-08 | 3.65E-07 |
| Tyms | 90.82 | -1.40 | 0.42 | -3.37 | 7.54E-04 | 7.51E-03 |
| Zfp516 | 707.94 | -1.40 | 0.23 | -6.20 | 5.49E-10 | 1.67E-08 |
| Midn | 6484.85 | -1.40 | 0.25 | -5.60 | 2.17E-08 | 5.48E-07 |
| Ccl6 | 535.00 | -1.40 | 0.42 | -3.36 | 7.76E-04 | 7.69E-03 |
| Rell1 | 631.15 | -1.40 | 0.27 | -5.15 | 2.64E-07 | 5.82E-06 |
| Hivep1 | 1666.03 | -1.40 | 0.17 | -8.11 | 5.17E-16 | 2.65E-14 |
| Bst2 | 572.49 | -1.41 | 0.22 | -6.46 | 1.08E-10 | 3.53E-09 |
| Hmga1 | 176.43 | -1.41 | 0.37 | -3.79 | 1.50E-04 | 1.84E-03 |
| Mmp9 | 67.98 | -1.42 | 0.52 | -2.73 | 6.35E-03 | 4.53E-02 |
| Tmem88b | 121.64 | -1.42 | 0.52 | -2.72 | 6.52E-03 | 4.63E-02 |
| Sp100 | 1228.60 | -1.42 | 0.26 | -5.44 | 5.36E-08 | 1.27E-06 |
| Noct | 15937.74 | -1.42 | 0.15 | -9.19 | 3.86E-20 | 2.81E-18 |
| Ccl21a | 452.93 | -1.42 | 0.32 | -4.45 | 8.68E-06 | 1.45E-04 |
| Brd2 | 3008.64 | -1.42 | 0.22 | -6.35 | 2.17E-10 | 6.94E-09 |
| Utp14b | 129.47 | -1.43 | 0.36 | -3.91 | 9.39E-05 | 1.22E-03 |
| Il10ra | 285.25 | -1.43 | 0.25 | -5.63 | 1.75E-08 | 4.48E-07 |
| Cd302 | 332.89 | -1.43 | 0.31 | -4.66 | 3.19E-06 | 5.79E-05 |
| Irs2 | 6543.54 | -1.43 | 0.23 | -6.32 | 2.69E-10 | 8.44E-09 |
| Ace | 1831.50 | -1.43 | 0.22 | -6.53 | 6.55E-11 | 2.17E-09 |
| Cd52 | 52.63 | -1.43 | 0.50 | -2.86 | 4.17E-03 | 3.22E-02 |
| Adgrg2 | 61.47 | -1.43 | 0.48 | -2.96 | 3.09E-03 | 2.52E-02 |
| Gna13 | 2198.48 | -1.44 | 0.18 | -8.04 | 9.30E-16 | 4.67E-14 |
| Bach1 | 2677.30 | -1.44 | 0.17 | -8.31 | 9.93E-17 | 5.34E-15 |
| Ets1 | 3015.43 | -1.44 | 0.26 | -5.51 | 3.56E-08 | 8.62E-07 |
| Id2 | 352.34 | -1.44 | 0.34 | -4.17 | 2.98E-05 | 4.33E-04 |
| Flnb | 4429.01 | -1.44 | 0.23 | -6.15 | 7.64E-10 | 2.29E-08 |
| Rap1b | 3803.14 | -1.44 | 0.21 | -6.93 | 4.23E-12 | 1.58E-10 |
| Rab20 | 975.17 | -1.44 | 0.27 | -5.44 | 5.37E-08 | 1.27E-06 |
| Hgf | 40.42 | -1.45 | 0.58 | -2.49 | 1.28E-02 | 7.77E-02 |
| Abcb1b | 460.56 | -1.45 | 0.28 | -5.10 | 3.37E-07 | 7.28E-06 |
| Crlf2 | 359.76 | -1.45 | 0.29 | -5.01 | 5.48E-07 | 1.14E-05 |
| Nacad | 46.30 | -1.45 | 0.55 | -2.63 | 8.52E-03 | 5.70E-02 |
| Clec12a | 104.91 | -1.45 | 0.38 | -3.78 | 1.59E-04 | 1.93E-03 |
| Sh3bgrl2 | 277.18 | -1.45 | 0.41 | -3.51 | 4.48E-04 | 4.84E-03 |
| Vmp1 | 1833.76 | -1.45 | 0.25 | -5.80 | 6.76E-09 | 1.81E-07 |
| Cars | 906.21 | -1.45 | 0.32 | -4.61 | 4.10E-06 | 7.30E-05 |
| Fcgr3 | 377.46 | -1.46 | 0.30 | -4.87 | 1.09E-06 | 2.14E-05 |
| Zbtb46 | 1067.19 | -1.46 | 0.21 | -7.09 | 1.35E-12 | 5.20E-11 |
| Ttc9 | 81.41 | -1.46 | 0.51 | -2.88 | 3.93E-03 | 3.07E-02 |
| Hap1 | 66.67 | -1.46 | 0.48 | -3.04 | 2.37E-03 | 2.01E-02 |
| Rffl | 366.98 | -1.46 | 0.29 | -4.95 | 7.40E-07 | 1.50E-05 |
| Gnl3 | 991.48 | -1.46 | 0.25 | -5.89 | 3.81E-09 | 1.06E-07 |
| Gpr4 | 729.17 | -1.46 | 0.30 | -4.84 | 1.28E-06 | 2.49E-05 |
| Slco2a1 | 263.07 | -1.46 | 0.41 | -3.60 | 3.14E-04 | 3.56E-03 |
| Nupr1 | 425.09 | -1.46 | 0.33 | -4.42 | 1.00E-05 | 1.65E-04 |
| Gdf15 | 188.66 | -1.46 | 0.36 | -4.08 | 4.51E-05 | 6.33E-04 |
| Cp | 3611.88 | -1.47 | 0.20 | -7.39 | 1.48E-13 | 6.20E-12 |
| Col20a1 | 119.10 | -1.47 | 0.34 | -4.33 | 1.52E-05 | 2.42E-04 |
| Klf5 | 76.55 | -1.47 | 0.44 | -3.34 | 8.29E-04 | 8.15E-03 |
| Ugcg | 851.34 | -1.47 | 0.22 | -6.59 | 4.42E-11 | 1.51E-09 |
| Cflar | 3332.97 | -1.47 | 0.17 | -8.51 | 1.68E-17 | 9.73E-16 |
| Katna1 | 753.55 | -1.47 | 0.23 | -6.42 | 1.38E-10 | 4.47E-09 |
| Bcl10 | 1321.50 | -1.48 | 0.22 | -6.73 | 1.66E-11 | 5.85E-10 |
| Dusp1 | 7654.18 | -1.48 | 0.24 | -6.10 | 1.04E-09 | 3.07E-08 |
| Simc1 | 243.79 | -1.48 | 0.29 | -5.13 | 2.93E-07 | 6.41E-06 |
| Dse | 394.77 | -1.48 | 0.36 | -4.15 | 3.37E-05 | 4.85E-04 |
| Ly6a | 2221.15 | -1.48 | 0.25 | -5.91 | 3.35E-09 | 9.32E-08 |
| Cxcl14 | 716.71 | -1.49 | 0.37 | -4.00 | 6.33E-05 | 8.56E-04 |
| Pdlim1 | 2642.99 | -1.49 | 0.22 | -6.89 | 5.65E-12 | 2.09E-10 |
| Pvr | 2001.19 | -1.49 | 0.24 | -6.13 | 8.82E-10 | 2.63E-08 |
| Cnn3 | 1991.92 | -1.49 | 0.19 | -7.92 | 2.46E-15 | 1.19E-13 |
| Rassf1 | 1906.31 | -1.49 | 0.28 | -5.41 | 6.29E-08 | 1.47E-06 |
| Kit | 185.84 | -1.49 | 0.35 | -4.22 | 2.47E-05 | 3.67E-04 |
| C3 | 10376.52 | -1.49 | 0.23 | -6.47 | 1.00E-10 | 3.29E-09 |
| Lcat | 87.45 | -1.49 | 0.42 | -3.55 | 3.86E-04 | 4.27E-03 |
| Lrrc32 | 1768.83 | -1.50 | 0.26 | -5.83 | 5.40E-09 | 1.46E-07 |
| Rap2b | 423.31 | -1.50 | 0.23 | -6.54 | 6.29E-11 | 2.09E-09 |
| Gm48493 | 231.52 | -1.50 | 0.36 | -4.16 | 3.19E-05 | 4.60E-04 |
| Taf7 | 555.76 | -1.50 | 0.25 | -5.99 | 2.10E-09 | 6.00E-08 |
| Cilp | 479.80 | -1.50 | 0.38 | -3.93 | 8.41E-05 | 1.11E-03 |
| 3110001I22Rik | 66.70 | -1.50 | 0.45 | -3.35 | 7.95E-04 | 7.87E-03 |
| Srxn1 | 1350.11 | -1.51 | 0.30 | -4.97 | 6.59E-07 | 1.35E-05 |
| Lmna | 10538.84 | -1.51 | 0.24 | -6.19 | 5.84E-10 | 1.77E-08 |
| Marcks | 1751.73 | -1.51 | 0.20 | -7.40 | 1.32E-13 | 5.55E-12 |
| Eif6 | 1590.66 | -1.51 | 0.33 | -4.61 | 4.10E-06 | 7.30E-05 |
| Medag | 894.78 | -1.51 | 0.35 | -4.27 | 1.98E-05 | 3.03E-04 |
| Rps6ka3 | 1965.51 | -1.51 | 0.18 | -8.35 | 6.59E-17 | 3.61E-15 |
| B2m | 6333.35 | -1.52 | 0.17 | -8.80 | 1.32E-18 | 8.37E-17 |
| Nlrp3 | 1051.86 | -1.52 | 0.27 | -5.70 | 1.20E-08 | 3.14E-07 |
| Crem | 717.91 | -1.52 | 0.39 | -3.91 | 9.10E-05 | 1.19E-03 |
| Gabpb1 | 621.06 | -1.52 | 0.23 | -6.51 | 7.64E-11 | 2.52E-09 |
| Skil | 1709.97 | -1.52 | 0.20 | -7.63 | 2.42E-14 | 1.08E-12 |
| Cnksr1 | 149.36 | -1.52 | 0.53 | -2.89 | 3.91E-03 | 3.05E-02 |
| Vash1 | 412.14 | -1.53 | 0.26 | -5.87 | 4.44E-09 | 1.22E-07 |
| Upk3b | 99.02 | -1.53 | 0.54 | -2.85 | 4.31E-03 | 3.30E-02 |
| Tac1 | 88.67 | -1.53 | 0.57 | -2.67 | 7.55E-03 | 5.19E-02 |
| Sash1 | 2828.74 | -1.53 | 0.19 | -8.22 | 2.10E-16 | 1.10E-14 |
| Lyz1 | 48.18 | -1.53 | 0.64 | -2.41 | 1.61E-02 | 9.19E-02 |
| Acsl4 | 882.87 | -1.53 | 0.25 | -6.10 | 1.04E-09 | 3.06E-08 |
| Slc9a3r1 | 288.28 | -1.53 | 0.28 | -5.49 | 4.01E-08 | 9.67E-07 |
| Ier3 | 6974.27 | -1.54 | 0.26 | -5.88 | 4.04E-09 | 1.11E-07 |
| Baiap2 | 634.92 | -1.55 | 0.27 | -5.69 | 1.24E-08 | 3.25E-07 |
| Igf2bp2 | 227.83 | -1.55 | 0.36 | -4.26 | 2.08E-05 | 3.16E-04 |
| Iffo2 | 438.37 | -1.55 | 0.23 | -6.62 | 3.67E-11 | 1.27E-09 |
| Anxa2 | 3319.67 | -1.55 | 0.32 | -4.78 | 1.71E-06 | 3.27E-05 |
| Steap4 | 2119.04 | -1.55 | 0.23 | -6.75 | 1.49E-11 | 5.25E-10 |
| Lrrc8b | 355.78 | -1.55 | 0.29 | -5.36 | 8.24E-08 | 1.91E-06 |
| Gda | 911.09 | -1.55 | 0.19 | -7.99 | 1.34E-15 | 6.64E-14 |
| Ifi35 | 618.19 | -1.56 | 0.23 | -6.84 | 8.00E-12 | 2.88E-10 |
| Ano5 | 81.47 | -1.56 | 0.65 | -2.41 | 1.58E-02 | 9.07E-02 |
| C5ar1 | 853.97 | -1.56 | 0.20 | -7.67 | 1.72E-14 | 7.75E-13 |
| Rcl1 | 231.84 | -1.56 | 0.32 | -4.93 | 8.13E-07 | 1.63E-05 |
| Itgb2 | 284.18 | -1.56 | 0.40 | -3.90 | 9.61E-05 | 1.24E-03 |
| Zfp36l1 | 8011.95 | -1.56 | 0.26 | -6.00 | 1.96E-09 | 5.64E-08 |
| Gm3636 | 51.61 | -1.56 | 0.53 | -2.96 | 3.06E-03 | 2.50E-02 |
| Acp5 | 126.93 | -1.57 | 0.34 | -4.56 | 5.11E-06 | 8.88E-05 |
| Ssh1 | 711.61 | -1.57 | 0.28 | -5.54 | 3.07E-08 | 7.53E-07 |
| Slc44a5 | 51.94 | -1.57 | 0.59 | -2.65 | 7.93E-03 | 5.39E-02 |
| Fkbp5 | 126.70 | -1.57 | 0.33 | -4.78 | 1.80E-06 | 3.41E-05 |
| Wsb1 | 2768.55 | -1.57 | 0.20 | -7.83 | 4.76E-15 | 2.23E-13 |
| Pprc1 | 1112.90 | -1.58 | 0.21 | -7.67 | 1.76E-14 | 7.90E-13 |
| Agpat4 | 384.37 | -1.58 | 0.34 | -4.59 | 4.40E-06 | 7.79E-05 |
| Spty2d1 | 1135.91 | -1.58 | 0.19 | -8.37 | 5.72E-17 | 3.15E-15 |
| Zc3hav1l | 115.57 | -1.58 | 0.37 | -4.27 | 1.99E-05 | 3.04E-04 |
| Zup1 | 324.67 | -1.58 | 0.31 | -5.05 | 4.40E-07 | 9.33E-06 |
| Ifitm2 | 1609.29 | -1.59 | 0.20 | -7.83 | 4.70E-15 | 2.21E-13 |
| Gclc | 1275.23 | -1.59 | 0.19 | -8.40 | 4.34E-17 | 2.42E-15 |
| Sptlc2 | 876.95 | -1.59 | 0.26 | -6.09 | 1.13E-09 | 3.33E-08 |
| Pappa | 52.06 | -1.60 | 0.51 | -3.10 | 1.92E-03 | 1.68E-02 |
| Klf4 | 6692.86 | -1.60 | 0.55 | -2.89 | 3.85E-03 | 3.02E-02 |
| Rab32 | 155.05 | -1.60 | 0.36 | -4.45 | 8.57E-06 | 1.43E-04 |
| Rpsa-ps10 | 156.76 | -1.60 | 0.31 | -5.13 | 2.94E-07 | 6.44E-06 |
| Rnf19b | 1811.92 | -1.60 | 0.20 | -7.95 | 1.82E-15 | 8.97E-14 |
| Etv6 | 633.19 | -1.60 | 0.26 | -6.13 | 8.89E-10 | 2.64E-08 |
| H2-D1 | 10205.28 | -1.60 | 0.24 | -6.79 | 1.09E-11 | 3.90E-10 |
| Fyb | 225.51 | -1.60 | 0.31 | -5.12 | 3.08E-07 | 6.71E-06 |
| Hsd11b1 | 326.74 | -1.60 | 0.29 | -5.55 | 2.87E-08 | 7.06E-07 |
| Zfp36 | 21266.52 | -1.61 | 0.26 | -6.27 | 3.67E-10 | 1.13E-08 |
| Stk36 | 43.78 | -1.61 | 0.58 | -2.79 | 5.27E-03 | 3.91E-02 |
| Ptprc | 445.56 | -1.61 | 0.25 | -6.42 | 1.38E-10 | 4.47E-09 |
| Fzd5 | 554.98 | -1.61 | 0.25 | -6.55 | 5.71E-11 | 1.91E-09 |
| Azin1 | 4902.92 | -1.62 | 0.20 | -7.90 | 2.81E-15 | 1.34E-13 |
| Kcne4 | 314.41 | -1.62 | 0.41 | -3.94 | 8.30E-05 | 1.10E-03 |
| Ramp3 | 216.30 | -1.62 | 0.35 | -4.68 | 2.85E-06 | 5.24E-05 |
| Sertad1 | 1925.22 | -1.62 | 0.28 | -5.87 | 4.33E-09 | 1.19E-07 |
| Aoah | 85.39 | -1.62 | 0.40 | -4.05 | 5.22E-05 | 7.25E-04 |
| Fgl2 | 5509.58 | -1.62 | 0.32 | -5.06 | 4.25E-07 | 9.06E-06 |
| Spi1 | 300.45 | -1.62 | 0.30 | -5.46 | 4.73E-08 | 1.13E-06 |
| Psmb8 | 288.67 | -1.62 | 0.26 | -6.25 | 4.17E-10 | 1.28E-08 |
| Znfx1 | 2495.69 | -1.62 | 0.31 | -5.30 | 1.17E-07 | 2.66E-06 |
| Stra6 | 36.71 | -1.62 | 0.68 | -2.38 | 1.73E-02 | 9.74E-02 |
| Zfp711 | 42.36 | -1.63 | 0.60 | -2.73 | 6.37E-03 | 4.54E-02 |
| Cdc42ep4 | 1012.69 | -1.63 | 0.34 | -4.79 | 1.64E-06 | 3.13E-05 |
| Ccnl1 | 3673.76 | -1.63 | 0.22 | -7.34 | 2.08E-13 | 8.61E-12 |
| Glis3 | 53.33 | -1.63 | 0.50 | -3.28 | 1.03E-03 | 9.85E-03 |
| Vasn | 982.71 | -1.63 | 0.31 | -5.29 | 1.25E-07 | 2.84E-06 |
| Adamtsl1 | 85.64 | -1.63 | 0.57 | -2.85 | 4.31E-03 | 3.30E-02 |
| Gm4202 | 65.85 | -1.64 | 0.66 | -2.50 | 1.26E-02 | 7.66E-02 |
| Ms4a6c | 187.99 | -1.64 | 0.30 | -5.52 | 3.38E-08 | 8.22E-07 |
| Traf3ip2 | 211.25 | -1.64 | 0.38 | -4.28 | 1.90E-05 | 2.95E-04 |
| Rgs17 | 46.04 | -1.64 | 0.56 | -2.94 | 3.32E-03 | 2.67E-02 |
| Rasip1 | 4390.33 | -1.64 | 0.19 | -8.47 | 2.46E-17 | 1.41E-15 |
| Cdc6 | 51.43 | -1.64 | 0.68 | -2.40 | 1.63E-02 | 9.28E-02 |
| Creb3l1 | 258.96 | -1.64 | 0.36 | -4.53 | 6.00E-06 | 1.03E-04 |
| Wt1 | 889.21 | -1.64 | 0.21 | -7.90 | 2.81E-15 | 1.34E-13 |
| Bcr | 1255.82 | -1.65 | 0.61 | -2.69 | 7.23E-03 | 5.02E-02 |
| Sesn2 | 428.89 | -1.65 | 0.35 | -4.68 | 2.93E-06 | 5.37E-05 |
| Aacs | 284.20 | -1.65 | 0.32 | -5.19 | 2.06E-07 | 4.59E-06 |
| Plekha4 | 259.49 | -1.65 | 0.33 | -5.03 | 5.02E-07 | 1.05E-05 |
| Tapbp | 134.53 | -1.65 | 0.33 | -4.98 | 6.45E-07 | 1.32E-05 |
| Slc39a6 | 318.03 | -1.65 | 0.27 | -6.02 | 1.71E-09 | 4.95E-08 |
| Sat1 | 3542.80 | -1.65 | 0.30 | -5.55 | 2.79E-08 | 6.89E-07 |
| Ankrd23 | 14126.70 | -1.65 | 0.23 | -7.11 | 1.14E-12 | 4.45E-11 |
| Arl13b | 1831.83 | -1.65 | 0.25 | -6.57 | 4.99E-11 | 1.69E-09 |
| Sema4a | 199.96 | -1.65 | 0.28 | -5.83 | 5.41E-09 | 1.46E-07 |
| Lgals3bp | 1354.95 | -1.65 | 0.18 | -9.13 | 7.15E-20 | 5.12E-18 |
| A3galt2 | 30.55 | -1.65 | 0.65 | -2.56 | 1.05E-02 | 6.70E-02 |
| Nfkbiz | 6861.24 | -1.66 | 0.19 | -8.87 | 7.17E-19 | 4.63E-17 |
| Tgfb1 | 1616.14 | -1.66 | 0.23 | -7.07 | 1.55E-12 | 5.97E-11 |
| Dennd2d | 26.73 | -1.66 | 0.65 | -2.54 | 1.10E-02 | 6.93E-02 |
| Ppp1r18 | 2359.19 | -1.67 | 0.20 | -8.38 | 5.42E-17 | 3.00E-15 |
| Nol10 | 537.23 | -1.67 | 0.34 | -4.95 | 7.41E-07 | 1.50E-05 |
| Dpep2 | 59.06 | -1.67 | 0.54 | -3.11 | 1.85E-03 | 1.62E-02 |
| Kif11 | 159.70 | -1.67 | 0.39 | -4.24 | 2.19E-05 | 3.31E-04 |
| Fosl2 | 9197.96 | -1.67 | 0.24 | -6.86 | 6.91E-12 | 2.52E-10 |
| Tnfrsf10b | 983.82 | -1.68 | 0.30 | -5.64 | 1.68E-08 | 4.32E-07 |
| Srpx | 197.21 | -1.68 | 0.43 | -3.88 | 1.03E-04 | 1.31E-03 |
| Plekhh1 | 89.49 | -1.68 | 0.42 | -3.99 | 6.53E-05 | 8.79E-04 |
| Piezo1 | 2632.44 | -1.68 | 0.27 | -6.18 | 6.60E-10 | 1.99E-08 |
| Gnai3 | 1935.75 | -1.68 | 0.19 | -9.05 | 1.47E-19 | 1.01E-17 |
| Trib3 | 150.18 | -1.68 | 0.57 | -2.96 | 3.07E-03 | 2.50E-02 |
| Vgll3 | 212.12 | -1.68 | 0.34 | -5.01 | 5.42E-07 | 1.13E-05 |
| Rasgrp1 | 33.83 | -1.68 | 0.61 | -2.77 | 5.61E-03 | 4.10E-02 |
| Osgin1 | 375.52 | -1.68 | 0.32 | -5.20 | 1.97E-07 | 4.41E-06 |
| Etv3 | 2123.42 | -1.68 | 0.17 | -9.98 | 1.83E-23 | 1.64E-21 |
| Alpk1 | 304.10 | -1.69 | 0.28 | -6.02 | 1.77E-09 | 5.09E-08 |
| Mob3c | 677.72 | -1.69 | 0.25 | -6.69 | 2.27E-11 | 7.88E-10 |
| Rela | 3640.56 | -1.70 | 0.17 | -9.79 | 1.20E-22 | 1.00E-20 |
| Irak2 | 963.38 | -1.70 | 0.19 | -8.75 | 2.18E-18 | 1.35E-16 |
| Tm4sf1 | 2984.39 | -1.70 | 0.33 | -5.18 | 2.27E-07 | 5.04E-06 |
| Npas2 | 135.13 | -1.70 | 0.41 | -4.12 | 3.76E-05 | 5.34E-04 |
| C4b | 993.67 | -1.70 | 0.33 | -5.09 | 3.58E-07 | 7.71E-06 |
| Glipr2 | 168.88 | -1.70 | 0.30 | -5.69 | 1.28E-08 | 3.35E-07 |
| Ptafr | 393.18 | -1.71 | 0.29 | -5.97 | 2.40E-09 | 6.83E-08 |
| Cpne7 | 42.02 | -1.71 | 0.61 | -2.79 | 5.21E-03 | 3.88E-02 |
| Itih4 | 79.24 | -1.71 | 0.49 | -3.47 | 5.17E-04 | 5.45E-03 |
| Cx3cl1 | 731.90 | -1.72 | 0.27 | -6.25 | 4.23E-10 | 1.29E-08 |
| Ngfr | 65.65 | -1.72 | 0.53 | -3.22 | 1.27E-03 | 1.17E-02 |
| Gas7 | 920.69 | -1.73 | 0.21 | -8.16 | 3.43E-16 | 1.77E-14 |
| Prpf40a | 49.15 | -1.73 | 0.57 | -3.03 | 2.45E-03 | 2.06E-02 |
| Frat2 | 531.90 | -1.73 | 0.33 | -5.18 | 2.16E-07 | 4.82E-06 |
| Ikbke | 180.54 | -1.74 | 0.41 | -4.22 | 2.41E-05 | 3.61E-04 |
| Rnf24 | 428.35 | -1.74 | 0.27 | -6.46 | 1.03E-10 | 3.36E-09 |
| 2200002D01Rik | 59.64 | -1.74 | 0.53 | -3.26 | 1.12E-03 | 1.06E-02 |
| Pxdc1 | 1360.58 | -1.74 | 0.29 | -6.10 | 1.03E-09 | 3.04E-08 |
| Cebpb | 3590.86 | -1.74 | 0.24 | -7.24 | 4.34E-13 | 1.76E-11 |
| Myo1e | 772.14 | -1.74 | 0.21 | -8.45 | 2.88E-17 | 1.63E-15 |
| Kctd12 | 2773.48 | -1.74 | 0.17 | -10.55 | 4.89E-26 | 5.52E-24 |
| Ldlr | 535.42 | -1.75 | 0.25 | -7.11 | 1.16E-12 | 4.51E-11 |
| Snx20 | 90.85 | -1.75 | 0.41 | -4.22 | 2.43E-05 | 3.62E-04 |
| Igsf6 | 154.27 | -1.75 | 0.37 | -4.67 | 3.01E-06 | 5.50E-05 |
| Epha2 | 1350.20 | -1.75 | 0.27 | -6.55 | 5.71E-11 | 1.91E-09 |
| Ugdh | 1652.37 | -1.76 | 0.36 | -4.91 | 9.01E-07 | 1.79E-05 |
| Acr | 30.54 | -1.76 | 0.68 | -2.59 | 9.47E-03 | 6.17E-02 |
| Rhbdf2 | 1746.56 | -1.76 | 0.17 | -10.15 | 3.16E-24 | 3.01E-22 |
| Txnrd1 | 3916.18 | -1.76 | 0.25 | -7.13 | 9.97E-13 | 3.90E-11 |
| Ptpn2 | 773.71 | -1.76 | 0.26 | -6.89 | 5.59E-12 | 2.07E-10 |
| Sema3f | 1241.79 | -1.76 | 0.23 | -7.76 | 8.49E-15 | 3.89E-13 |
| Rasd1 | 608.26 | -1.77 | 0.29 | -6.09 | 1.16E-09 | 3.40E-08 |
| Anxa1 | 1751.04 | -1.77 | 0.26 | -6.77 | 1.31E-11 | 4.69E-10 |
| Ncf4 | 85.62 | -1.77 | 0.44 | -4.03 | 5.51E-05 | 7.59E-04 |
| Sp110 | 419.71 | -1.77 | 0.32 | -5.59 | 2.32E-08 | 5.82E-07 |
| Aldh1a3 | 68.11 | -1.77 | 0.50 | -3.55 | 3.90E-04 | 4.30E-03 |
| Meox1 | 400.42 | -1.77 | 0.26 | -6.74 | 1.53E-11 | 5.41E-10 |
| Susd1 | 37.75 | -1.77 | 0.60 | -2.94 | 3.23E-03 | 2.61E-02 |
| Junb | 20622.48 | -1.78 | 0.29 | -6.04 | 1.51E-09 | 4.37E-08 |
| Gramd1a | 2825.38 | -1.78 | 0.21 | -8.56 | 1.09E-17 | 6.40E-16 |
| Cdr2l | 309.46 | -1.78 | 0.36 | -4.99 | 6.16E-07 | 1.27E-05 |
| Themis2 | 109.24 | -1.78 | 0.36 | -4.99 | 6.08E-07 | 1.25E-05 |
| Fcer1g | 255.31 | -1.78 | 0.32 | -5.50 | 3.76E-08 | 9.09E-07 |
| Tnf | 2737.78 | -1.78 | 0.18 | -10.07 | 7.36E-24 | 6.75E-22 |
| Tlr2 | 1372.70 | -1.78 | 0.22 | -8.00 | 1.22E-15 | 6.07E-14 |
| Mmp14 | 912.37 | -1.79 | 0.25 | -7.21 | 5.50E-13 | 2.21E-11 |
| Egr2 | 2404.42 | -1.79 | 0.31 | -5.84 | 5.18E-09 | 1.40E-07 |
| Pakap | 12645.57 | -1.79 | 0.16 | -11.22 | 3.14E-29 | 4.43E-27 |
| Egr1 | 33080.74 | -1.79 | 0.24 | -7.44 | 1.03E-13 | 4.37E-12 |
| Baz1a | 932.43 | -1.79 | 0.21 | -8.55 | 1.28E-17 | 7.48E-16 |
| Exoc3l4 | 240.80 | -1.79 | 0.36 | -4.95 | 7.52E-07 | 1.52E-05 |
| Gcc1 | 901.78 | -1.80 | 0.31 | -5.89 | 3.97E-09 | 1.10E-07 |
| Cyth4 | 291.28 | -1.80 | 0.27 | -6.58 | 4.59E-11 | 1.56E-09 |
| Slc16a3 | 339.16 | -1.80 | 0.45 | -3.99 | 6.54E-05 | 8.79E-04 |
| Pawr | 170.25 | -1.80 | 0.31 | -5.88 | 4.00E-09 | 1.10E-07 |
| Nppb | 2826.72 | -1.81 | 0.60 | -3.02 | 2.53E-03 | 2.12E-02 |
| Ulbp1 | 100.99 | -1.81 | 0.49 | -3.74 | 1.88E-04 | 2.22E-03 |
| Cd69 | 27.62 | -1.82 | 0.73 | -2.49 | 1.27E-02 | 7.73E-02 |
| Zmynd15 | 311.82 | -1.82 | 0.29 | -6.32 | 2.54E-10 | 8.01E-09 |
| Izumo1 | 82.25 | -1.82 | 0.45 | -4.08 | 4.54E-05 | 6.37E-04 |
| Phlda1 | 3211.82 | -1.82 | 0.33 | -5.54 | 3.03E-08 | 7.44E-07 |
| Map3k14 | 611.45 | -1.82 | 0.20 | -8.96 | 3.12E-19 | 2.08E-17 |
| Ankrd42 | 34.03 | -1.82 | 0.70 | -2.60 | 9.24E-03 | 6.08E-02 |
| Tmem106a | 242.23 | -1.83 | 0.29 | -6.32 | 2.66E-10 | 8.35E-09 |
| Fignl2 | 216.00 | -1.83 | 0.37 | -4.88 | 1.04E-06 | 2.04E-05 |
| Timeless | 1142.34 | -1.83 | 0.30 | -6.03 | 1.62E-09 | 4.69E-08 |
| Vmn1r47 | 23.35 | -1.83 | 0.71 | -2.59 | 9.64E-03 | 6.26E-02 |
| H2-K1 | 2610.39 | -1.83 | 0.22 | -8.20 | 2.41E-16 | 1.26E-14 |
| Lgals9 | 857.90 | -1.83 | 0.23 | -7.88 | 3.27E-15 | 1.55E-13 |
| Rab11fip1 | 133.47 | -1.83 | 0.39 | -4.68 | 2.86E-06 | 5.24E-05 |
| Spred1 | 1498.81 | -1.84 | 0.23 | -7.90 | 2.71E-15 | 1.30E-13 |
| Gm6548 | 180.52 | -1.84 | 0.34 | -5.44 | 5.30E-08 | 1.26E-06 |
| Mndal | 317.55 | -1.84 | 0.32 | -5.71 | 1.10E-08 | 2.92E-07 |
| Ier5 | 6119.03 | -1.84 | 0.28 | -6.56 | 5.46E-11 | 1.85E-09 |
| Dusp5 | 1929.53 | -1.85 | 0.23 | -8.09 | 6.05E-16 | 3.08E-14 |
| Kcnk13 | 26.23 | -1.85 | 0.71 | -2.61 | 9.16E-03 | 6.04E-02 |
| Pstpip1 | 59.16 | -1.85 | 0.47 | -3.92 | 8.86E-05 | 1.16E-03 |
| Nup210l | 30.72 | -1.85 | 0.63 | -2.94 | 3.32E-03 | 2.67E-02 |
| Srgn | 1347.02 | -1.85 | 0.30 | -6.23 | 4.53E-10 | 1.38E-08 |
| Zfp593 | 309.83 | -1.85 | 0.37 | -4.95 | 7.57E-07 | 1.52E-05 |
| Ell2 | 1076.61 | -1.85 | 0.21 | -8.79 | 1.44E-18 | 9.08E-17 |
| Plekho2 | 2417.50 | -1.86 | 0.27 | -6.93 | 4.08E-12 | 1.53E-10 |
| Relt | 148.54 | -1.86 | 0.38 | -4.94 | 7.67E-07 | 1.54E-05 |
| Parvg | 31.53 | -1.86 | 0.76 | -2.44 | 1.49E-02 | 8.67E-02 |
| Slamf1 | 33.40 | -1.86 | 0.63 | -2.93 | 3.36E-03 | 2.70E-02 |
| Lilr4b | 1704.71 | -1.86 | 0.26 | -7.26 | 3.81E-13 | 1.55E-11 |
| Hcls1 | 382.46 | -1.86 | 0.28 | -6.73 | 1.68E-11 | 5.91E-10 |
| Rasgef1b | 2024.93 | -1.86 | 0.17 | -10.71 | 9.39E-27 | 1.11E-24 |
| Mitd1 | 119.31 | -1.86 | 0.39 | -4.81 | 1.52E-06 | 2.93E-05 |
| Fscn1 | 1338.00 | -1.86 | 0.21 | -8.83 | 1.05E-18 | 6.75E-17 |
| Stat2 | 1687.69 | -1.86 | 0.21 | -9.09 | 9.93E-20 | 7.00E-18 |
| 9130008F23Rik | 36.52 | -1.87 | 0.70 | -2.65 | 7.96E-03 | 5.40E-02 |
| Grrp1 | 318.29 | -1.87 | 0.46 | -4.05 | 5.21E-05 | 7.23E-04 |
| Akap12 | 3545.60 | -1.87 | 0.21 | -8.91 | 5.26E-19 | 3.46E-17 |
| Ntrk2 | 68.78 | -1.87 | 0.53 | -3.53 | 4.15E-04 | 4.54E-03 |
| Mcm10 | 28.54 | -1.88 | 0.78 | -2.39 | 1.68E-02 | 9.48E-02 |
| Myo1g | 210.11 | -1.88 | 0.32 | -5.93 | 2.95E-09 | 8.32E-08 |
| Eda2r | 246.27 | -1.88 | 0.32 | -5.92 | 3.27E-09 | 9.15E-08 |
| Rnd3 | 1590.84 | -1.88 | 0.21 | -8.94 | 4.03E-19 | 2.66E-17 |
| Tchh | 71.11 | -1.88 | 0.56 | -3.36 | 7.69E-04 | 7.64E-03 |
| Star | 42.26 | -1.89 | 0.65 | -2.93 | 3.34E-03 | 2.68E-02 |
| Jdp2 | 607.51 | -1.91 | 0.28 | -6.86 | 6.83E-12 | 2.50E-10 |
| Rel | 2175.16 | -1.91 | 0.18 | -10.32 | 5.52E-25 | 5.58E-23 |
| Eif2b5 | 33.87 | -1.91 | 0.75 | -2.56 | 1.04E-02 | 6.64E-02 |
| Crtc2 | 1779.36 | -1.92 | 0.26 | -7.27 | 3.63E-13 | 1.48E-11 |
| Peg10 | 168.63 | -1.92 | 0.38 | -5.04 | 4.74E-07 | 9.99E-06 |
| Fos | 17612.39 | -1.92 | 0.32 | -5.91 | 3.33E-09 | 9.29E-08 |
| Rpl10a | 325.69 | -1.92 | 0.36 | -5.41 | 6.40E-08 | 1.49E-06 |
| Parp10 | 817.25 | -1.92 | 0.26 | -7.39 | 1.46E-13 | 6.13E-12 |
| Alkal2 | 80.66 | -1.92 | 0.44 | -4.39 | 1.15E-05 | 1.87E-04 |
| 9930111J21Rik2 | 648.36 | -1.92 | 0.34 | -5.71 | 1.12E-08 | 2.96E-07 |
| Rbm47 | 66.06 | -1.92 | 0.48 | -4.05 | 5.12E-05 | 7.12E-04 |
| Slfn5 | 6297.06 | -1.93 | 0.19 | -9.96 | 2.29E-23 | 2.00E-21 |
| Inhbb | 215.61 | -1.93 | 0.43 | -4.44 | 8.88E-06 | 1.47E-04 |
| Lbp | 359.22 | -1.93 | 0.30 | -6.33 | 2.44E-10 | 7.71E-09 |
| Hk3 | 99.54 | -1.93 | 0.48 | -4.06 | 4.94E-05 | 6.89E-04 |
| Sox9 | 166.10 | -1.94 | 0.41 | -4.68 | 2.81E-06 | 5.18E-05 |
| 5430427O19Rik | 25.87 | -1.94 | 0.73 | -2.67 | 7.59E-03 | 5.22E-02 |
| Lcp1 | 1419.53 | -1.94 | 0.19 | -9.98 | 1.85E-23 | 1.65E-21 |
| Tor3a | 554.31 | -1.94 | 0.30 | -6.58 | 4.58E-11 | 1.56E-09 |
| Bdkrb2 | 158.41 | -1.95 | 0.36 | -5.36 | 8.16E-08 | 1.89E-06 |
| Pirb | 353.50 | -1.95 | 0.28 | -7.05 | 1.76E-12 | 6.74E-11 |
| Fgfr2 | 24.96 | -1.95 | 0.72 | -2.73 | 6.29E-03 | 4.49E-02 |
| Mid1 | 470.16 | -1.96 | 0.27 | -7.17 | 7.75E-13 | 3.06E-11 |
| H3f3b | 9901.44 | -1.96 | 0.25 | -7.80 | 6.09E-15 | 2.82E-13 |
| Adamts1 | 7984.47 | -1.96 | 0.28 | -7.03 | 2.07E-12 | 7.88E-11 |
| Col12a1 | 92.70 | -1.96 | 0.49 | -4.04 | 5.43E-05 | 7.48E-04 |
| Slc1a1 | 353.81 | -1.96 | 0.27 | -7.37 | 1.65E-13 | 6.88E-12 |
| Gpr176 | 18.26 | -1.97 | 0.80 | -2.45 | 1.44E-02 | 8.44E-02 |
| Cenpl | 43.50 | -1.97 | 0.66 | -2.97 | 3.00E-03 | 2.46E-02 |
| Trim25 | 1885.42 | -1.97 | 0.18 | -11.07 | 1.73E-28 | 2.27E-26 |
| Plau | 1241.88 | -1.98 | 0.62 | -3.22 | 1.30E-03 | 1.20E-02 |
| Tpm3 | 4748.56 | -1.98 | 0.29 | -6.80 | 1.05E-11 | 3.78E-10 |
| Map3k6 | 1480.86 | -1.98 | 0.28 | -7.21 | 5.54E-13 | 2.22E-11 |
| Arid5a | 4621.23 | -1.99 | 0.24 | -8.20 | 2.47E-16 | 1.29E-14 |
| Gm8281 | 49.28 | -1.99 | 0.60 | -3.30 | 9.68E-04 | 9.35E-03 |
| Fut2 | 36.32 | -1.99 | 0.64 | -3.10 | 1.93E-03 | 1.68E-02 |
| Tox2 | 76.16 | -1.99 | 0.42 | -4.71 | 2.43E-06 | 4.53E-05 |
| Xlr3b | 19.72 | -1.99 | 0.83 | -2.39 | 1.69E-02 | 9.55E-02 |
| Irf1 | 9854.61 | -1.99 | 0.23 | -8.54 | 1.32E-17 | 7.68E-16 |
| Irf9 | 1414.03 | -1.99 | 0.19 | -10.54 | 5.84E-26 | 6.44E-24 |
| Mapk6 | 2202.64 | -2.00 | 0.18 | -11.22 | 3.17E-29 | 4.43E-27 |
| Mmp12 | 65.21 | -2.00 | 0.52 | -3.86 | 1.12E-04 | 1.41E-03 |
| Mki67 | 206.05 | -2.00 | 0.43 | -4.66 | 3.22E-06 | 5.82E-05 |
| Basp1 | 119.24 | -2.01 | 0.40 | -5.04 | 4.54E-07 | 9.59E-06 |
| Kdm6b | 8223.37 | -2.01 | 0.19 | -10.51 | 8.19E-26 | 8.84E-24 |
| Bcl2a1b | 295.70 | -2.01 | 0.32 | -6.26 | 3.97E-10 | 1.22E-08 |
| Rab8b | 1229.53 | -2.01 | 0.23 | -8.87 | 7.01E-19 | 4.55E-17 |
| Il1b | 1466.92 | -2.02 | 0.23 | -8.79 | 1.52E-18 | 9.46E-17 |
| Atf3 | 21240.57 | -2.02 | 0.27 | -7.56 | 4.09E-14 | 1.80E-12 |
| Irgm2 | 2103.59 | -2.03 | 0.22 | -9.06 | 1.27E-19 | 8.85E-18 |
| Slamf8 | 28.53 | -2.03 | 0.72 | -2.83 | 4.66E-03 | 3.54E-02 |
| Slc25a25 | 2370.11 | -2.03 | 0.19 | -10.45 | 1.42E-25 | 1.49E-23 |
| Daxx | 96.10 | -2.03 | 0.41 | -4.96 | 7.22E-07 | 1.46E-05 |
| Slc13a3 | 25.51 | -2.04 | 0.75 | -2.72 | 6.61E-03 | 4.68E-02 |
| Ubash3b | 297.27 | -2.04 | 0.45 | -4.53 | 5.90E-06 | 1.02E-04 |
| Ube2c | 15.72 | -2.04 | 0.86 | -2.38 | 1.73E-02 | 9.71E-02 |
| Enc1 | 1205.03 | -2.04 | 0.27 | -7.46 | 8.78E-14 | 3.79E-12 |
| Oas2 | 659.15 | -2.04 | 0.23 | -8.71 | 2.97E-18 | 1.82E-16 |
| Psme2b | 51.82 | -2.04 | 0.53 | -3.84 | 1.23E-04 | 1.53E-03 |
| Bcat1 | 53.49 | -2.04 | 0.71 | -2.87 | 4.16E-03 | 3.22E-02 |
| Cd3d | 20.08 | -2.05 | 0.80 | -2.57 | 1.02E-02 | 6.55E-02 |
| Uap1 | 3846.04 | -2.05 | 0.31 | -6.55 | 5.67E-11 | 1.91E-09 |
| Fcgr1 | 72.35 | -2.05 | 0.42 | -4.87 | 1.10E-06 | 2.15E-05 |
| Ppp1r15a | 7680.56 | -2.06 | 0.27 | -7.73 | 1.11E-14 | 5.06E-13 |
| Gprc5a | 237.18 | -2.07 | 0.42 | -4.96 | 6.90E-07 | 1.40E-05 |
| Foxp4 | 1076.85 | -2.07 | 0.21 | -9.70 | 3.13E-22 | 2.52E-20 |
| Hsp90aa1 | 13197.84 | -2.07 | 0.18 | -11.24 | 2.55E-29 | 3.67E-27 |
| Kif22 | 20.82 | -2.07 | 0.84 | -2.46 | 1.37E-02 | 8.18E-02 |
| Micall2 | 254.27 | -2.07 | 0.30 | -6.89 | 5.75E-12 | 2.12E-10 |
| Stap1 | 73.43 | -2.07 | 0.47 | -4.39 | 1.13E-05 | 1.84E-04 |
| Gvin1 | 3083.41 | -2.07 | 0.20 | -10.38 | 3.10E-25 | 3.18E-23 |
| Rhoc | 4297.92 | -2.07 | 0.27 | -7.82 | 5.39E-15 | 2.51E-13 |
| Bmp2 | 306.53 | -2.07 | 0.27 | -7.61 | 2.80E-14 | 1.24E-12 |
| Tgm1 | 18.29 | -2.08 | 0.83 | -2.51 | 1.21E-02 | 7.44E-02 |
| Rtn4rl2 | 192.44 | -2.08 | 0.39 | -5.30 | 1.18E-07 | 2.68E-06 |
| Pde4b | 5774.48 | -2.08 | 0.25 | -8.22 | 2.07E-16 | 1.09E-14 |
| Gbp7 | 1161.11 | -2.08 | 0.22 | -9.59 | 9.17E-22 | 7.31E-20 |
| Tent5c | 143.50 | -2.08 | 0.35 | -5.95 | 2.75E-09 | 7.79E-08 |
| Cytip | 47.22 | -2.09 | 0.55 | -3.80 | 1.45E-04 | 1.77E-03 |
| Gbp9 | 1667.16 | -2.09 | 0.29 | -7.23 | 4.68E-13 | 1.89E-11 |
| Jak2 | 2686.03 | -2.09 | 0.24 | -8.63 | 6.20E-18 | 3.70E-16 |
| H2-Q10 | 505.20 | -2.09 | 0.24 | -8.88 | 6.93E-19 | 4.52E-17 |
| Nfkbia | 10300.25 | -2.09 | 0.20 | -10.25 | 1.13E-24 | 1.09E-22 |
| Tlr1 | 73.65 | -2.10 | 0.43 | -4.86 | 1.17E-06 | 2.29E-05 |
| Rab15 | 61.52 | -2.10 | 0.52 | -4.00 | 6.29E-05 | 8.52E-04 |
| Usp27x | 104.86 | -2.10 | 0.46 | -4.58 | 4.54E-06 | 7.98E-05 |
| Ripk3 | 492.93 | -2.10 | 0.35 | -5.97 | 2.43E-09 | 6.92E-08 |
| Ripk1 | 1340.78 | -2.10 | 0.27 | -7.92 | 2.41E-15 | 1.17E-13 |
| Zfp473 | 30.85 | -2.11 | 0.85 | -2.49 | 1.28E-02 | 7.77E-02 |
| Tgif1 | 756.90 | -2.12 | 0.28 | -7.57 | 3.70E-14 | 1.63E-12 |
| Oas3 | 57.73 | -2.12 | 0.51 | -4.20 | 2.69E-05 | 3.96E-04 |
| H2-Q7 | 3098.22 | -2.13 | 0.18 | -11.63 | 2.90E-31 | 4.73E-29 |
| Tnfaip2 | 8888.51 | -2.13 | 0.18 | -11.99 | 3.84E-33 | 6.86E-31 |
| Vav1 | 144.66 | -2.13 | 0.34 | -6.29 | 3.11E-10 | 9.70E-09 |
| Gch1 | 1070.65 | -2.14 | 0.21 | -10.11 | 4.91E-24 | 4.58E-22 |
| Bcl2a1d | 121.99 | -2.14 | 0.62 | -3.47 | 5.14E-04 | 5.43E-03 |
| Aldh1a2 | 331.94 | -2.14 | 0.30 | -7.17 | 7.33E-13 | 2.91E-11 |
| Rgs1 | 111.40 | -2.14 | 0.50 | -4.33 | 1.51E-05 | 2.41E-04 |
| Nts | 335.45 | -2.15 | 0.39 | -5.52 | 3.37E-08 | 8.21E-07 |
| Lacc1 | 413.21 | -2.15 | 0.25 | -8.75 | 2.05E-18 | 1.27E-16 |
| Ccrl2 | 1191.47 | -2.15 | 0.28 | -7.66 | 1.82E-14 | 8.17E-13 |
| Cd44 | 1108.58 | -2.15 | 0.24 | -8.89 | 6.20E-19 | 4.06E-17 |
| Orai2 | 170.04 | -2.16 | 0.38 | -5.73 | 9.84E-09 | 2.61E-07 |
| Col9a3 | 35.60 | -2.16 | 0.73 | -2.95 | 3.20E-03 | 2.59E-02 |
| Adgrg3 | 235.95 | -2.16 | 0.38 | -5.62 | 1.93E-08 | 4.90E-07 |
| Gm42417 | 90.83 | -2.17 | 0.67 | -3.23 | 1.25E-03 | 1.16E-02 |
| Snx10 | 969.15 | -2.17 | 0.20 | -10.84 | 2.11E-27 | 2.69E-25 |
| H2-T10 | 74.65 | -2.17 | 0.46 | -4.67 | 3.00E-06 | 5.49E-05 |
| 1500009L16Rik | 70.05 | -2.18 | 0.52 | -4.15 | 3.26E-05 | 4.70E-04 |
| Fam83g | 49.33 | -2.18 | 0.70 | -3.12 | 1.79E-03 | 1.57E-02 |
| Nrip3 | 24.70 | -2.18 | 0.81 | -2.69 | 7.08E-03 | 4.95E-02 |
| Slc5a3 | 311.61 | -2.18 | 0.26 | -8.49 | 2.02E-17 | 1.16E-15 |
| Prkcg | 1242.44 | -2.18 | 0.21 | -10.55 | 5.24E-26 | 5.82E-24 |
| Cyp1a1 | 3529.29 | -2.18 | 0.68 | -3.22 | 1.27E-03 | 1.17E-02 |
| 2010300C02Rik | 60.41 | -2.19 | 0.57 | -3.81 | 1.37E-04 | 1.69E-03 |
| Cybb | 1448.15 | -2.19 | 0.25 | -8.65 | 5.31E-18 | 3.19E-16 |
| D16Ertd472e | 52.36 | -2.19 | 0.61 | -3.57 | 3.60E-04 | 4.01E-03 |
| Dab2 | 2893.25 | -2.20 | 0.20 | -10.81 | 3.00E-27 | 3.73E-25 |
| Apobec3 | 500.81 | -2.20 | 0.24 | -9.27 | 1.84E-20 | 1.36E-18 |
| Lax1 | 17.66 | -2.20 | 0.86 | -2.55 | 1.09E-02 | 6.86E-02 |
| Nop58 | 1619.12 | -2.20 | 0.30 | -7.23 | 4.89E-13 | 1.97E-11 |
| Gpr68 | 40.50 | -2.20 | 0.82 | -2.69 | 7.11E-03 | 4.96E-02 |
| Sema7a | 2796.98 | -2.21 | 0.21 | -10.37 | 3.25E-25 | 3.31E-23 |
| Psd4 | 63.81 | -2.21 | 0.57 | -3.89 | 9.94E-05 | 1.27E-03 |
| Adam8 | 75.35 | -2.21 | 0.46 | -4.78 | 1.79E-06 | 3.40E-05 |
| Parp9 | 932.86 | -2.22 | 0.28 | -8.04 | 9.14E-16 | 4.61E-14 |
| Mmp3 | 292.51 | -2.22 | 0.36 | -6.15 | 7.95E-10 | 2.37E-08 |
| Ifitm3 | 3576.70 | -2.22 | 0.23 | -9.71 | 2.66E-22 | 2.16E-20 |
| Bcl2a1a | 53.34 | -2.22 | 0.65 | -3.40 | 6.71E-04 | 6.81E-03 |
| Il11 | 40.48 | -2.23 | 0.78 | -2.87 | 4.10E-03 | 3.18E-02 |
| Gm8995 | 1786.33 | -2.23 | 0.30 | -7.33 | 2.30E-13 | 9.46E-12 |
| Odc1 | 5160.52 | -2.23 | 0.27 | -8.43 | 3.61E-17 | 2.03E-15 |
| Taf4b | 300.87 | -2.24 | 0.32 | -6.98 | 2.93E-12 | 1.10E-10 |
| Nfkb1 | 4128.54 | -2.24 | 0.22 | -10.41 | 2.23E-25 | 2.32E-23 |
| Ddx58 | 1123.64 | -2.24 | 0.21 | -10.79 | 3.87E-27 | 4.78E-25 |
| Acta1 | 3036.07 | -2.25 | 0.57 | -3.91 | 9.24E-05 | 1.20E-03 |
| Gm49037 | 39.59 | -2.25 | 0.85 | -2.64 | 8.37E-03 | 5.63E-02 |
| Fcgr2b | 713.15 | -2.25 | 0.30 | -7.43 | 1.10E-13 | 4.67E-12 |
| Dtx3l | 1004.84 | -2.25 | 0.28 | -8.10 | 5.59E-16 | 2.86E-14 |
| Zfp954 | 334.57 | -2.25 | 0.35 | -6.35 | 2.21E-10 | 7.03E-09 |
| Itpkc | 868.50 | -2.27 | 0.29 | -7.82 | 5.49E-15 | 2.55E-13 |
| Pde12 | 1106.56 | -2.27 | 0.31 | -7.40 | 1.40E-13 | 5.89E-12 |
| Milr1 | 41.56 | -2.27 | 0.55 | -4.13 | 3.59E-05 | 5.13E-04 |
| Mlkl | 213.60 | -2.27 | 0.32 | -7.12 | 1.06E-12 | 4.14E-11 |
| Trim21 | 292.49 | -2.27 | 0.36 | -6.28 | 3.42E-10 | 1.06E-08 |
| Dnaja1 | 7647.31 | -2.28 | 0.24 | -9.44 | 3.87E-21 | 2.99E-19 |
| Spon2 | 153.77 | -2.28 | 0.49 | -4.62 | 3.84E-06 | 6.87E-05 |
| Asb4 | 615.66 | -2.28 | 0.78 | -2.91 | 3.61E-03 | 2.87E-02 |
| Pgf | 585.37 | -2.28 | 0.33 | -6.88 | 6.11E-12 | 2.24E-10 |
| Samd9l | 971.91 | -2.29 | 0.29 | -7.82 | 5.30E-15 | 2.48E-13 |
| Plat | 1455.77 | -2.29 | 0.26 | -8.81 | 1.20E-18 | 7.64E-17 |
| Nptx1 | 44.46 | -2.30 | 0.77 | -2.98 | 2.93E-03 | 2.41E-02 |
| Fosb | 24226.73 | -2.31 | 0.26 | -8.97 | 3.03E-19 | 2.03E-17 |
| Eif2ak2 | 884.12 | -2.31 | 0.32 | -7.14 | 9.57E-13 | 3.77E-11 |
| Osr2 | 19.90 | -2.31 | 0.82 | -2.82 | 4.82E-03 | 3.64E-02 |
| Trib1 | 2662.41 | -2.32 | 0.25 | -9.20 | 3.49E-20 | 2.55E-18 |
| Tnfrsf11a | 158.96 | -2.32 | 0.35 | -6.59 | 4.35E-11 | 1.49E-09 |
| Lratd1 | 77.61 | -2.33 | 0.42 | -5.56 | 2.65E-08 | 6.56E-07 |
| Adgrd1 | 739.18 | -2.33 | 0.25 | -9.32 | 1.13E-20 | 8.44E-19 |
| Tgif2 | 374.00 | -2.33 | 0.29 | -8.16 | 3.38E-16 | 1.75E-14 |
| Pou3f1 | 44.84 | -2.33 | 0.58 | -4.03 | 5.63E-05 | 7.70E-04 |
| Csf1 | 13135.81 | -2.34 | 0.18 | -12.86 | 7.14E-38 | 1.54E-35 |
| Ripor3 | 88.09 | -2.34 | 0.54 | -4.35 | 1.37E-05 | 2.20E-04 |
| Adamts9 | 2138.70 | -2.34 | 0.23 | -10.11 | 5.09E-24 | 4.73E-22 |
| H2-Q5 | 836.76 | -2.35 | 0.23 | -10.12 | 4.65E-24 | 4.37E-22 |
| Vcam1 | 8656.19 | -2.35 | 0.17 | -13.72 | 8.16E-43 | 2.14E-40 |
| Slamf7 | 182.06 | -2.35 | 0.46 | -5.13 | 2.90E-07 | 6.37E-06 |
| Ifi203 | 2609.47 | -2.36 | 0.23 | -10.29 | 7.79E-25 | 7.67E-23 |
| Cd86 | 370.60 | -2.37 | 0.29 | -8.12 | 4.64E-16 | 2.39E-14 |
| Plscr1 | 862.48 | -2.37 | 0.26 | -9.12 | 7.54E-20 | 5.34E-18 |
| Hamp | 53.04 | -2.37 | 0.64 | -3.73 | 1.94E-04 | 2.30E-03 |
| Apaf1 | 725.04 | -2.37 | 0.23 | -10.08 | 6.90E-24 | 6.37E-22 |
| Gm11427 | 61.07 | -2.37 | 0.50 | -4.79 | 1.70E-06 | 3.24E-05 |
| Ikzf1 | 144.27 | -2.38 | 0.43 | -5.57 | 2.49E-08 | 6.19E-07 |
| Nlrc5 | 448.95 | -2.39 | 0.25 | -9.45 | 3.51E-21 | 2.73E-19 |
| Xaf1 | 608.05 | -2.39 | 0.27 | -8.79 | 1.47E-18 | 9.21E-17 |
| Slc7a2 | 564.02 | -2.39 | 0.34 | -7.13 | 9.79E-13 | 3.84E-11 |
| Hells | 46.44 | -2.39 | 0.58 | -4.14 | 3.53E-05 | 5.06E-04 |
| Sema4c | 2874.90 | -2.39 | 0.23 | -10.30 | 7.01E-25 | 6.99E-23 |
| Mthfd2 | 292.59 | -2.39 | 0.45 | -5.36 | 8.42E-08 | 1.94E-06 |
| Ptges | 111.81 | -2.39 | 0.38 | -6.33 | 2.53E-10 | 7.97E-09 |
| Ccr1 | 82.04 | -2.40 | 0.46 | -5.22 | 1.78E-07 | 4.03E-06 |
| Eif1a | 710.91 | -2.40 | 0.27 | -8.75 | 2.09E-18 | 1.29E-16 |
| Rgs16 | 1026.70 | -2.40 | 0.30 | -8.02 | 1.07E-15 | 5.34E-14 |
| Areg | 100.03 | -2.40 | 0.43 | -5.59 | 2.23E-08 | 5.62E-07 |
| Rdh10 | 525.43 | -2.40 | 0.36 | -6.75 | 1.46E-11 | 5.19E-10 |
| Cnmd | 40.90 | -2.41 | 0.56 | -4.26 | 2.00E-05 | 3.06E-04 |
| Sntb2 | 1661.15 | -2.42 | 0.23 | -10.58 | 3.73E-26 | 4.24E-24 |
| Sowahc | 561.40 | -2.42 | 0.24 | -10.15 | 3.21E-24 | 3.04E-22 |
| Cd53 | 359.16 | -2.42 | 0.24 | -10.04 | 1.00E-23 | 9.09E-22 |
| Arntl | 415.16 | -2.42 | 0.36 | -6.79 | 1.16E-11 | 4.13E-10 |
| Arhgap8 | 50.23 | -2.42 | 0.59 | -4.10 | 4.06E-05 | 5.74E-04 |
| Cd80 | 55.90 | -2.42 | 0.50 | -4.80 | 1.57E-06 | 3.02E-05 |
| Trim30d | 196.30 | -2.42 | 0.41 | -5.89 | 3.84E-09 | 1.06E-07 |
| Gem | 2488.46 | -2.43 | 0.26 | -9.34 | 9.59E-21 | 7.33E-19 |
| Ifrd1 | 10640.46 | -2.43 | 0.23 | -10.53 | 6.07E-26 | 6.65E-24 |
| Hsph1 | 4824.35 | -2.43 | 0.27 | -8.86 | 7.84E-19 | 5.04E-17 |
| Nfil3 | 1587.92 | -2.43 | 0.31 | -7.96 | 1.71E-15 | 8.44E-14 |
| Tnfaip8l1 | 174.90 | -2.44 | 0.43 | -5.69 | 1.30E-08 | 3.38E-07 |
| Apobr | 84.84 | -2.44 | 0.49 | -5.00 | 5.64E-07 | 1.17E-05 |
| Rnf122 | 545.16 | -2.45 | 0.39 | -6.34 | 2.27E-10 | 7.20E-09 |
| Ralgds | 1809.73 | -2.46 | 0.22 | -11.22 | 3.35E-29 | 4.63E-27 |
| 9930111J21Rik1 | 62.34 | -2.47 | 0.61 | -4.03 | 5.67E-05 | 7.74E-04 |
| Sdc4 | 3588.36 | -2.47 | 0.27 | -9.08 | 1.04E-19 | 7.31E-18 |
| Ccno | 120.88 | -2.47 | 0.41 | -6.06 | 1.40E-09 | 4.06E-08 |
| Errfi1 | 5012.66 | -2.48 | 0.29 | -8.58 | 9.86E-18 | 5.83E-16 |
| Siglece | 61.95 | -2.48 | 0.53 | -4.65 | 3.28E-06 | 5.91E-05 |
| Msr1 | 346.82 | -2.48 | 0.29 | -8.56 | 1.12E-17 | 6.54E-16 |
| Zc3hav1 | 1953.13 | -2.49 | 0.19 | -13.00 | 1.15E-38 | 2.61E-36 |
| Socs3 | 11226.21 | -2.49 | 0.32 | -7.69 | 1.51E-14 | 6.85E-13 |
| Ccl22 | 55.60 | -2.50 | 0.68 | -3.69 | 2.22E-04 | 2.60E-03 |
| Cebpd | 2444.19 | -2.50 | 0.33 | -7.53 | 5.15E-14 | 2.26E-12 |
| Egfr | 807.80 | -2.51 | 0.29 | -8.64 | 5.75E-18 | 3.44E-16 |
| Traf1 | 292.10 | -2.51 | 0.33 | -7.65 | 1.96E-14 | 8.76E-13 |
| Osm | 43.67 | -2.51 | 0.55 | -4.53 | 5.78E-06 | 9.97E-05 |
| Serpine1 | 10647.87 | -2.51 | 0.22 | -11.58 | 5.09E-31 | 8.21E-29 |
| Ptprj | 913.69 | -2.51 | 0.26 | -9.57 | 1.08E-21 | 8.52E-20 |
| Slc10a6 | 393.90 | -2.51 | 0.30 | -8.46 | 2.64E-17 | 1.51E-15 |
| Gbp10 | 81.32 | -2.52 | 0.50 | -5.04 | 4.69E-07 | 9.88E-06 |
| Bcl3 | 1516.79 | -2.52 | 0.22 | -11.39 | 4.87E-30 | 7.29E-28 |
| Il1r1 | 1571.25 | -2.53 | 0.36 | -7.00 | 2.60E-12 | 9.82E-11 |
| Myd88 | 1457.81 | -2.53 | 0.28 | -9.12 | 7.39E-20 | 5.27E-18 |
| Irak3 | 667.48 | -2.53 | 0.26 | -9.78 | 1.41E-22 | 1.17E-20 |
| Lck | 14.32 | -2.53 | 0.96 | -2.64 | 8.31E-03 | 5.60E-02 |
| Tsku | 160.30 | -2.53 | 0.36 | -7.04 | 1.87E-12 | 7.12E-11 |
| Ido1 | 16.58 | -2.53 | 1.00 | -2.53 | 1.13E-02 | 7.08E-02 |
| Pou2f2 | 149.89 | -2.53 | 0.44 | -5.81 | 6.20E-09 | 1.66E-07 |
| Gm28661 | 9452.56 | -2.53 | 0.73 | -3.48 | 4.97E-04 | 5.28E-03 |
| Emp1 | 6706.89 | -2.53 | 0.32 | -7.88 | 3.36E-15 | 1.59E-13 |
| Marcksl1 | 3140.46 | -2.54 | 0.18 | -14.23 | 6.24E-46 | 1.99E-43 |
| Spag5 | 24.34 | -2.55 | 0.93 | -2.73 | 6.25E-03 | 4.48E-02 |
| Arc | 1047.98 | -2.55 | 0.32 | -8.06 | 7.46E-16 | 3.79E-14 |
| Zc3h12a | 2130.01 | -2.55 | 0.24 | -10.66 | 1.59E-26 | 1.85E-24 |
[truncated: 31,830 more chars]
